# Catalytic enantioselective nucleophilic desymmetrization of phosphonate esters

**DOI:** 10.1038/s41557-023-01165-6

**Published:** 2023-05-01

**Authors:** Michele Formica, Tatiana Rogova, Heyao Shi, Naoto Sahara, Branislav Ferko, Alistair J. M. Farley, Kirsten E. Christensen, Fernanda Duarte, Ken Yamazaki, Darren J. Dixon

**Affiliations:** 1 Chemistry Research Laboratory, Department of Chemistry, University of Oxford, Oxford, UK; 2 Graduate School of Engineering, Nagoya University, Nagoya, Japan; 3 Division of Applied Chemistry, Okayama University, Okayama, Japan

**Keywords:** Organocatalysis, Synthetic chemistry methodology, Asymmetric catalysis, Synthetic chemistry methodology

## Abstract

Molecules that contain a stereogenic phosphorus atom are crucial to medicine, agrochemistry and catalysis. While methods are available for the selective construction of various chiral organophosphorus compounds, catalytic enantioselective approaches for their synthesis are far less common. Given the vastness of possible substituent combinations around a phosphorus atom, protocols for their preparation should also be divergent, providing facile access not only to one but to many classes of phosphorus compounds. Here we introduce a catalytic and enantioselective strategy for the preparation of an enantioenriched phosphorus(V) centre that can be diversified enantiospecifically to a wide range of biologically relevant phosphorus(V) compounds. The process, which involves an enantioselective nucleophilic substitution catalysed by a superbasic bifunctional iminophosphorane catalyst, can accommodate a wide range of carbon substituents at phosphorus. The resulting stable, yet versatile, synthetic intermediates can be combined with a multitude of medicinally relevant O-, N- and S-based nucleophiles.

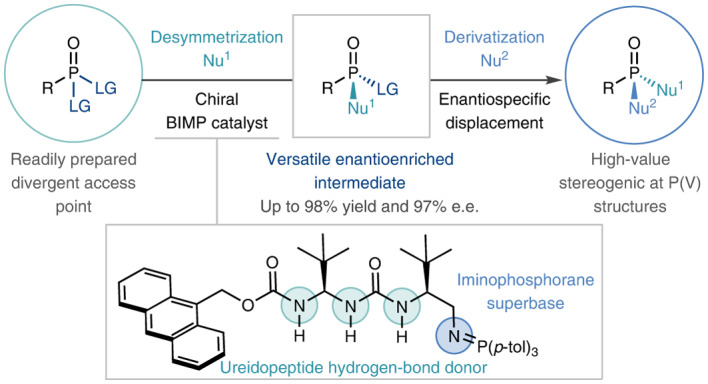

## Abstract (web summary)

Catalytic enantioselective approaches to access chiral organophosphorus compounds are rare. A two-stage catalytic strategy for the synthesis of diverse enantioenriched P(V) compounds has now been developed: a bifunctional iminophosphorane superbase enables enantioselective nucleophilic desymmetrization, followed by downstream enantiospecific diversification of the resulting intermediate by substitution with medicinally relevant O-, N- and S-based nucleophiles.

## Main

Compounds containing one or more stereogenic phosphorus atoms in the P(V) oxidation state are important to chemistry, biology and medicine^1^. These include marketed antiviral drugs such as Tenofovir alafenamide and Remdesivir^2^, an effective treatment for Ebola that has also recently been approved for use against SARS-CoV-2. Existing approaches for the stereoselective synthesis of P-stereogenic centres, while elegant, remain mostly diastereoselective, with catalytic enantioselective approaches being limited in application. Accordingly, divergent, broad-scope, catalytic strategies for the efficient stereoselective synthesis of diverse stereogenic P(V)-containing compounds remain essential^3,4^.

To this end, we describe an enantioselective two-stage strategy, exploiting a catalytic and highly enantioselective^5–7^ desymmetrization^8^ of phosphonate esters. Pivoting on a stereocontrolled, sequential nucleophilic substitution of enantiotopic leaving groups from readily accessible prochiral P(V) precursors, a bifunctional iminophosphorane (BIMP)^9–11^ superbase catalyst was found to be essential in delivering reactive desymmetrized intermediates capable of downstream enantiospecific substitution. This uniquely modular, catalytic platform allows broad-scope, stereoselective access to a diverse library of chiral P(V) compounds including those with O, N and S linkages.

Over the past few years, substantial progress has been made in improving the synthesis of P(V) chiral compounds. In 2017, Merck reported a diastereoselective dynamic kinetic asymmetric transformation (DYKAT) to synthesize MK-3682, a lead ProTide (ref. ^12^) antiviral compound as well as other analogues^13^. The process was reliant on a chiral C2 symmetric nucleophilic organocatalyst, which provided the desired prodrug with exquisite diastereocontrol from racemic P(V) starting material.

Recently, the Baran group reported a highly versatile chiral P(V) reagent: PSI (ψ), derived from limonene, for the diastereoselective synthesis of phosphorothioate oligonucleotides and of cyclic dinucleotides (CDNs)^14,15^. Building upon previous chiral auxiliary approaches^16–21^, this work was also expanded towards the synthesis of P-chiral phosphines, as well as methylphosphonate oligonucleotides (MPOs)^22^.

**Fig. 1:**
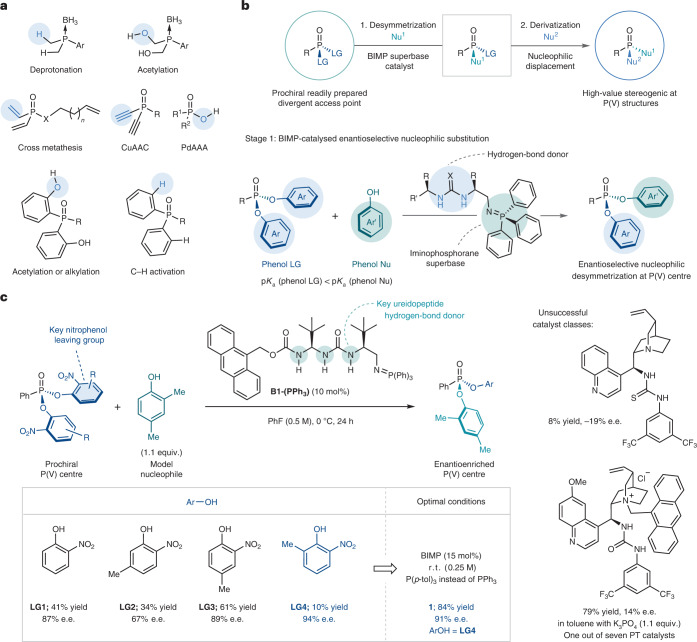
Previous work, reaction design and optimisation. **a**, State-of-the-art in catalytic enantioselective desymmetrization of P(V) compounds. **b**, A two-phase strategy for the construction of diverse enantioenriched P(V) compounds consisting of a bifunctional iminophosphorane-catalysed enantioselective nucleophilic substitution and downstream enantiospecific modification of the resulting enantioenriched intermediate. **c**, Effect of the nature of the leaving group on reaction efficiency and selectivity (left) with representative unsuccessful catalyst classes (right). CuAAC, copper-catalysed azide–alkyne cycloaddition; PdAAA, palladium-catalysed asymmetric allylic alkylation; LG, leaving group; Nu, nucleophile; r. t., room temperature; PT, phase transfer.

While these elegant processes towards P-chiral compounds are effective, they remain diastereoselective, where the absolute stereochemical configuration at the phosphorus centre is controlled either through a matched combination of catalyst and a carefully tailored starting material or by employing a stoichiometric chiral leaving group. Notwithstanding sophisticated, contemporary enantioselective arylation^23,24^ and allylation^25^ protocols of secondary phosphine oxides (limited to all carbon substituents on phosphorus), a few notable reports have also been seen where a P-stereogenic centre is generated indirectly through the functionalization of enantiotopic P-bound groups. However, this approach inherently limits the scope and application of these protocols (Fig. 1a)^26–36^. Despite these impressive advances, to date, no catalytic enantioselective desymmetrization protocols have been established with reactivity occurring directly at the phosphorus atom^37^ (following the disclosure of this work as a preprint and while this paper was under revision, an elegant and complementary catalytic enantioselective nucleophilic desymmetrization of phosphoryl dichlorides was published by the Jacobsen group^38^).

We envisioned a two-stage desymmetrization–derivatization strategy by which enantiotopic phenolic leaving groups on a prochiral phosphonate ester are enantiodiscriminated by a suitable nucleophile under the control of a chiral Brønsted base catalyst^39^, generating a new P−O bond. The resulting enantioenriched intermediate would retain suitable reactivity for sequential substitution of the remaining leaving group. With appropriate stereocontrol, this uniquely modular approach would overcome the key restrictions of previously developed protocols. Given the tunability and high basicity of our bifunctional iminophosphorane catalysts^9–11^, we expected that they would provide sufficient pronucleophile/substrate activation and could be suitably adapted to obtain high levels of enantiopurity in the products (Fig. 1b).

## Results and discussion

To explore the proposed reaction design, we began our investigations by identifying an appropriate model system containing a phosphonyl dichloride mimic, utilizing 2,4-dimethylphenol as the nucleophile and a BIMP superbase catalyst. The leaving group had to possess key characteristics based on the dichotomy between leaving group ability/basicity and stability of the P(V) precursor to allow for reactivity and catalyst turnover. After a comprehensive investigation of potential leaving groups, *para*-nitrophenol was identified to possess good reactivity as well as crucially permitting catalyst turnover^40^ (Supplementary Scheme 1). Following this key breakthrough, a survey of nitrophenol isomers resulted in *ortho*-nitrophenol (**LG1**) being identified as optimal for enantioselectivity. The development of a new BIMP catalyst bearing a ureidopeptide hydrogen-bond donor motif (**B1**), inspired by the work of Palomo^41–43^, and tuning of the reaction conditions led to a first lead result of 41% yield and 87% e.e. (Fig. 1c). To augment catalyst turnover, the p*K*_a_ of the leaving group was carefully adjusted by introducing a methyl-group at different positions around the aromatic ring of the nitrophenol (**LG2**–**LG4**). While the installation of a methyl group at the 4-position (**LG3**) gave the best yield (61%), a methyl group at the 6-position (**LG4**) gave optimal enantioselectivity (94% e.e.) albeit with a drop in reactivity. By conducting the reaction at room temperature and fine-tuning the basicity of the catalyst (exchanging PPh_3_ with P(*p*-tol)_3_ for the generation of the iminophosphorane), the yield of product **1** was increased to 84% and its e.e. was found to be 91%. For comparison, it is worthy of note that both Cinchona-derived bifunctional organocatalysts and phase-transfer catalysis were ineffective in providing the desired product in high yield and enantioselectivity (Supplementary Schemes 2 and 3).

With the optimized conditions in hand, we proceeded to explore the scope of this enantioselective nucleophilic desymmetrization (Table 1). We were pleased to find that a variety of phenols possessing *ortho*-substituents (**2**–**6**) were well tolerated and high yields and enantioselectivities were maintained. Compound **6** (98% yield, 91% e.e.) is of particular interest as a precursor to cycloSal-type prodrugs^44^. 2,3-Disubstituted phenols were also found to be effective nucleophiles (**7**). The introduction of electron-withdrawing groups (**8**) was also possible, despite a slight reduction in enantioselectivity. Bulky, naturally occurring phenols totarol and thymol were also competent nucleophiles for delivering the desymmetrized P(V) products (**9, 10**) in good yield and high enantioselectivity. Importantly, when totarol was reacted with **P1**, catalysed by achiral base BEMP (2-*tert*-butylimino-2-diethylamino-1,3-dimethylperhydro-1,3,2-diazaphosphorine), no diastereoselectivity was observed, demonstrating that the stereoselective reaction was under complete catalyst control. Using carvacrol as a nucleophile (**11**) resulted in diminished enantioselectivity. Nucleophiles bearing no *ortho*-substituent were found to afford the corresponding products in good yields but poor enantioselectivity, confirming that the *ortho*-substituent was a key discriminating feature in the transition structures governing the stereoselectivity of the reaction. Additionally, heterocyclic phenols or alkyl alcohols were found to be unreactive under the optimized conditions (Supplementary Scheme 8). The reaction was also successful on a gram scale (3 mmol) with no loss in yield or enantioselectivity. Desymmetrized product **1** was recrystallized to >99.9% e.e. and the absolute stereochemical configuration was assigned as (*R*) by single crystal X-ray diffraction analysis (see Supplementary Data 1–2). Having probed the scope of the nucleophilic partner, we proceeded to vary the P-linked carbon substituent on the phosphonate ester electrophile. Such changes were broadly tolerated with aryl (**12**), methyl (**13**), higher alkyl (**14**, **16**) and β-branched (**17**) substituents maintaining high reaction efficiency and enantioselectivities. Substrates containing α-branched substituents (**15**) were less reactive, resulting in low yield of product and diminished enantioselectivity, most probably due to the increased steric hindrance around the P atom. Benzyl substituents (**18**–**20**) were also tolerated, albeit with a slight decrease in selectivity. Finally, we were pleased to find that thiophene (**21**)- and (thio)ether (**22**, **23**)-substituted prochiral phosphorus electrophiles were also competent substrates for our methodology. Once again, increasing the steric demand around the P atom by installing a *tert*-butyldiphenylsilyl (TBDPS) silyl ether (**24**) resulted in diminished reactivity. However, in this case, good enantioselectivity was maintained.

**Table 1:**
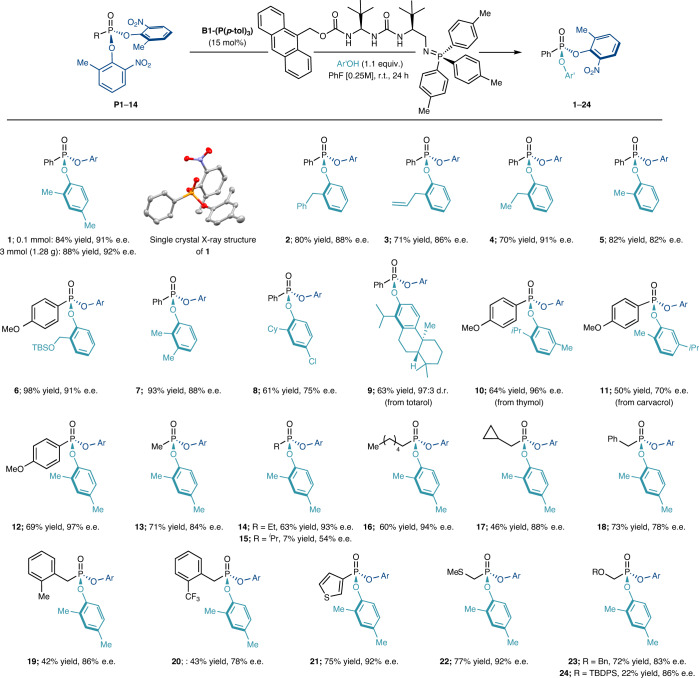
Scope of the desymmetrization reaction with respect to the phenol nucleophile and electrophilic P(V) substrate

Bn, benzyl; Cy, cyclohexyl; TBS, *tert*-butyldimethylsilyl; TBDPS, *tert*-butyldiphenylsilyl; r. t., room temperature.

Having established the scope of the desymmetrization for both the nucleophilic and electrophilic components, we proceeded to assess the crucial second stage of the strategy: the enantiospecific nucleophilic substitution of the remaining nitrophenol leaving group (Table 2). After a preliminary investigation into the applicability of existing protocols, we found that Lewis acid activation of the P(V) species, pioneered by Merck^45^, was highly efficient in promoting the second nucleophilic displacement. Indeed, treating compound **1** with 1.5 equiv. each of ^*t*^BuMgCl and benzyl alcohol in THF at room temperature smoothly afforded mixed phosphonate ester **25** in almost quantitative yield and 100% enantiospecificity (e.s.). Encouraged by this success, we proceeded to use more complex and biologically relevant alcohols as nucleophiles. All four DNA nucleosides (**26**–**29**), hepatitis C treatment sofosbuvir (**30**) as well as acetal-protected uridine (**31**) and adenosine (**32**) were successfully phosphorylated at the 5′-OH with moderate to good yields and >95:5 d.r. Acetal-protected d-glucose (**33**) and deoxythymidine (**34**) were both phosphorylated at the more hindered secondary 3′-OH in excellent yields and diastereoselectivity. Having established reactivity with alcohol nucleophiles we proceeded to investigate the stereocontrolled formation of alternative P-heteroatom linkages. However, when using *n*-propanethiol as the nucleophile, no desired product was obtained when using ^*t*^BuMgCl to promote the reaction. The main by-product observed was (2-methyl-6-nitrophenyl)(propyl)thioether, obtained via an S_N_Ar mechanism. This obstacle was overcome by switching the promoter to 3 equiv. of DBU (1,8-diazabicyclo(5.4.0)undec-7-ene), which gave the desired thiophosphonate ester **35** in 59% yield and 98% e.s.. We then turned our attention to N-centred nucleophiles and were thrilled to find that when using *N*-Boc benzylamine (Boc, *tert*-butyloxycarbonyl) as the nucleophile we could obtain phosphonamidite ester **36** in 84% yield and 100% e.s. Finally, *tert*-butyldimethylsilyl (TBS)-protected phosphonate ester **6** was deprotected with trifluoroacetic acid (TFA) and cyclized with ^*t*^BuMgCl to give cycloSal derivative **37** in 67% yield and 92% e.s. over two steps. Upon recrystallization to enantiopurity and single crystal X-ray diffraction analysis (see Supplementary Data 1–2), the absolute configuration of **37** was determined to be (*R*), showing inversion of configuration^21,46–48^ with respect to the starting material. To confirm the importance of controlling stereochemical configuration at all substitution steps, acetal-protected uridine and d-glucose were also reacted with racemic **1**. Both compounds **31** and **33** were obtained as a 1:1 mixture of diastereoisomers, thus highlighting the importance of deploying enantioenriched **1** in the second nucleophilic substitution to obtain a single diastereomeric product (see NMR data for compounds **31** and **33** in the Supplementary Information for details).

**Table 2:**
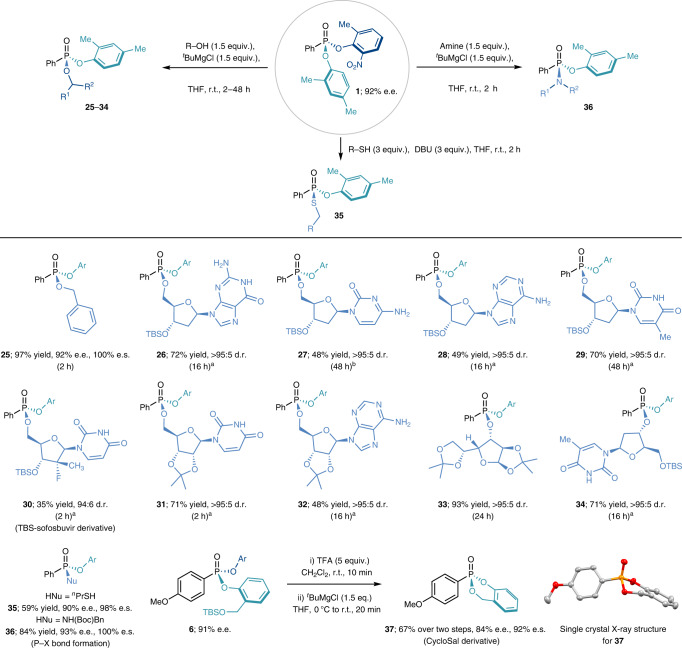
Derivatization of the desymmetrized enantioenriched P(V) products

P−O bond formation, P−S and P−N bond formation and synthesis of a cycloSal prodrug derivative. ^a^ ^*t*^BuMgCl (3 equiv.) was used. ^b^ ^*t*^BuMgCl (4 equiv.) was used. DBU, 1,8-diazabicyclo(5.4.0)undec-7-ene; TBS, *tert*-butyldimethylsilyl; TFA, trifluoroacetic acid; Bn, benzyl; Boc, *tert*-butyloxycarbonyl; r.t., room temperature.

**Fig. 2:**
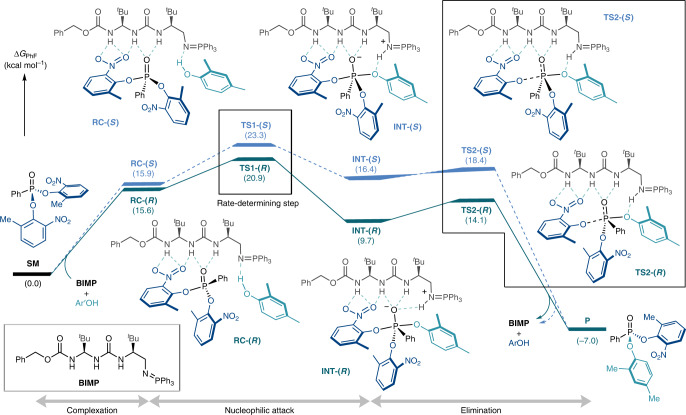
DFT study. Potential energy surface [Δ*G* (kcal mol^−1^)] for the enantioselective nucleophilic desymmetrization of the P(V) compound **P1** with 2-methyl-6-nitrophenol using the model BIMP catalyst **B12-P(Ph)**_**3**_ computed at the SMD(fluorobenzene)/M06-2X/def2TZVP//SMD(fluorobenzene)/M06-2X/def2SVP level of theory. The reaction was found to proceed in three stages: complexation, nucleophilic attack and elimination from a metastable pentacoordinate intermediate. The rate- (and enantio-) determining step was found to be nucleophilic addition of the phenol to **P1** with the TS leading to the (*R*)-product (**TS1-(*R*)**) being more favourable than that leading to the (*S*)-product (**TS1-(*S*)**) by 2.4 kcal mol^−1^. Gibbs free energies (kcal mol^−1^) are reported in parenthesis. ArOH, 2-methyl-6-nitrophenol; Ar′OH, 2,4-dimethylphenol; SM, starting material.

To further probe the mechanism and establish the origins of enantioselectivity, a computational density functional theory (DFT) study was undertaken^47–50^. The catalysed reaction was modelled with **B12-P(Ph)**_**3**_ containing a benzyloxycarbonyl (Cbz) carbamate and a triphenylphosphine-derived iminophosphorane moiety (Fig. 2). The computed potential energy surfaces indicated that the initial nucleophilic attack for the formation of the pentacoordinate intermediate is the rate- and enantio-determining step. Pleasingly, the favoured transition state (TS) **TS1-(*R*)** leads to the desymmetrized product with experimentally confirmed absolute stereochemical configuration by single crystal X-ray diffraction studies (ΔΔ*G*^‡^ = 2.4 kcal mol^−1^), and the predicted enantioselectivity of 97% e.e. is in good agreement with the experimental selectivity of 92% e.e. Once the pentacoordinate intermediate **INT-(*R*)** is obtained, the carbamate-assisted facile elimination of the nitrophenol group occurs through **TS2-(*R*)** to furnish the desymmetrized product.

**Fig. 3:**
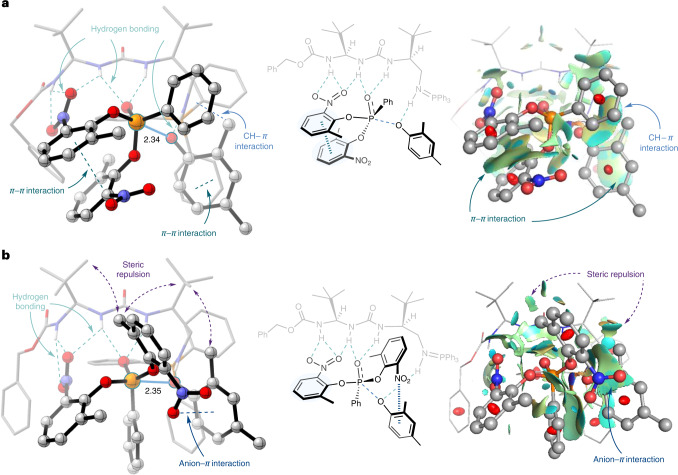
Rate-determining nucleophilic attack TS structures and the visualization of the NCI surfaces. **a**, Three-dimensional structures of **TS1-(*R*)** for the formation of the (*R*)-product. **b**, Three-dimensional structures of **TS1-(*S*)** for the formation of the (*S*)-product. Bond lengths of the TS geometries are given in Å. NCI surfaces are represented as the coloured regions in the figure. Multiple stabilising inter- and intra-molecular NCIs such as hydrogen bonding, *π*–*π* and CH–*π* interactions are observed in the favoured TS **TS1-(*R*)**, while the destabilizing steric repulsion between the substrate, nucleophile and catalyst exists in the unfavoured TS **TS1-(*S*)**.

To obtain further insight into the origin of enantioselectivity with the optimized BIMP catalyst, the TS structures for the initial nucleophilic attack were analysed (Fig. 3). The favoured TS conformation in **TS1-(*R*)** engages in several stabilizing non-covalent interactions (NCIs) such as hydrogen bonding, *π*–*π* and CH–*π* interactions (Fig. 3a). The notable characteristic feature is that the two nitrophenol aromatic rings are tightly bound by a *π*–*π* interaction, which, in the absence of significant steric repulsions, stabilises the TS. On the other hand, the disfavoured TS **TS1-(*S*)** does not display such an interaction. Instead, steric repulsion between the substrate, nucleophile and catalyst can be observed that could contribute to destabilizing the TS (Fig. 3b). A qualitative analysis of the NCI surfaces provided further evidence of the existence of the above-mentioned interactions for the origin of enantioselectivity^51^. Furthermore, the specific roles of substituents in the optimized catalyst and substrates were examined by calculating the key nucleophilic attack TS structures with different catalyst backbones, which were later verified experimentally (Supplementary Fig. 1). Specifically, the *t*-Bu groups on the catalyst and the *ortho*-methyl group of the nucleophile contribute to destabilizing the disfavoured TS structure due to steric repulsion. On the other hand, the *ortho*-nitro group of the leaving group is required for the substrate to bind to the catalyst tightly via hydrogen-bond interactions. The insights gained from both experiments and computation demonstrate that key NCIs arising from the tunable nature of the catalyst and leaving group are vital for the observed catalytic and enantioselective efficiency.

In conclusion, a two-stage strategy for the synthesis of stereogenic P(V) compounds through an unprecedented enantioselective nucleophilic desymmetrization and subsequent enantiospecific derivatization, was developed. A BIMP catalyst bearing a ureidopeptide hydrogen-bond donor moiety provided a unique chiral environment and sufficient pronucleophile/substrate activation to allow the desymmetrization to proceed with excellent yield and enantioselectivity. Through judicious choice of leaving group, facile downstream diversification of the desymmetrized P(V) ester with a multitude of medicinally relevant nucleophiles with very high enantiospecificity was allowed. This study represents a clear strategic departure from previously established catalytic methods that rely on substrate engineering and do not easily allow for facile downstream modification to medicinally attractive molecules. Finally, DFT studies were carried out to elucidate an energetically plausible reaction pathway for the two enantiomeric products and to uncover the main attractive NCIs responsible for the high levels of enantioselectivity observed for the major enantiomer. This conceptually novel, two-stage desymmetrization/derivatization approach has enabled modular catalytic enantioselective access to stereogenic P(V) compounds by a strategy that we hope will unlock new opportunities and inspire further developments in the field.

## Methods

To the corresponding organoazide (0.015 mmol) and tris-*para*-tolylphosphine (0.015 mmol) under an argon atmosphere was added THF (0.40 ml). Then the reaction mixture was stirred for 24 h at room temperature. The formation of the organocatalysts was monitored by thin-layer chromatography. Upon completion volatiles were removed under a stream of N_2_, yielding the expected iminiphosphorane (**B1-(P(*p*-tol)**_**3**_) that was used without further purification.

To the corresponding phosphonate (0.10 mmol) and BIMP organocatalyst (0.015 mmol) under an argon atmosphere was added PhF (0.40 ml) and phenol (0.11 mmol, 1.1 equiv.). The reaction mixture was stirred at room temperature for 24 h. Then it was loaded directly onto silica gel and purified by flash column chromatography to afford pure desymmetrized phosphonate ester.

### Reporting summary

Further information on research design is available in the Nature Portfolio Reporting Summary linked to this article.

## Online content

Any methods, additional references, Nature Portfolio reporting summaries, source data, extended data, supplementary information, acknowledgements, peer review information; details of author contributions and competing interests; and statements of data and code availability are available at 10.1038/s41557-023-01165-6.

## Supplementary information

Supplementary Information Supplementary Schemes 1–9, Figs. 1–4, Table 1, synthetic procedures, NMR spectra, HPLC traces, X-ray data and additional computational data.

Reporting Summary

Supplementary Data 1 Crystallographic data for compound 1; CCDC reference 2097043.

Supplementary Data 2 Structure factors file for compound 1; CCDC reference 2097043.

Supplementary Data 3 Crystallographic data for compound 37; CCDC reference 2097044.

Supplementary Data 4 Structure factors file for compound 37; CCDC reference 2097044.

Supplementary Data 5 XYZ coordinates for: the substrate, product, BIMP catalyst, *R* and *S* pentacoordinate intermediates, *R* and *S* transition states, *R* and *S* reaction coordinates, the phenol nucleophile and the nitrophenol leaving group.

## Data Availability

Crystallographic data are available free of charge from the Cambridge Crystallographic Data Centre under references CCDC 2097043 (**1**) and CCDC 2097044 (**37**). Additional optimization data, full synthetic methods and characterization data are available in the Supplementary Information.
