# Supplementary material for: Catalytic enantioselective nucleophilic desymmetrization of phosphonate esters
Source: Nat Chem. 2023 May 1;15(5):714–21. doi: 10.1038/s41557-023-01165-6 (PMC10159838; doi:10.1038/s41557-023-01165-6)
Supplement: Supplementary file 1 — Supplementary Schemes 1–9, Figs. 1–4, Table 1, synthetic procedures, NMR spectra, HPLC traces, X-ray data and additional computational data. [file 41557_2023_1165_MOESM1_ESM.pdf]

nature chemistry

Article https://doi.org/10.1038/s41557-023-01165-6

Catalytic enantioselective nucleophilic desymmetrization of phosphonate esters

In the format provided by the authors and unedited

Supporting Information for "Catalytic Enantioselective Nucleophilic Desymmetrisation of Phosphonate Esters"

Authors: Michele Formica[1], Tatiana Rogova[1], Heyao Shi[1], Naoto Sahara[1,2], Branislav Ferko[1], Alistair J. M. Farley[1], Kirsten E. Christensen[1], Fernanda Duarte[1], Ken Yamazaki[3]* & Darren J. Dixon[1]*

Affiliation: [1]Department of Chemistry, University of Oxford, Chemical Research Laboratory, 12 Mansfield Road, Oxford, OX1 3TA, UK; [2]Graduate School of Engineering, Nagoya University, B2-3(611), Furo-cho, Chikusa, Nagoya 464-8603, Japan; [3]Division of Applied Chemistry, Okayama University, Tsushimanaka, Okayama 700-8530, Japan

Contents

General Information 3
Optimisation: 4
Computational Studies 13
Synthesis of Catalyst B1: 17
Synthesis of Catalyst B12: 19
Synthesis of Catalyst B13: 20
Substrate Synthesis: 21
General Procedure I: Synthesis of P(V) desymmetrisation substrates 21
Synthesis of Starting Materials 22
General Procedure II: Enantioselective Desymmetrisation of Phosphonate esters 40
General Procedure III: Racemic Desymmetrisation of Phosphonate esters 40
Products: Leaving Group Variation 41
Products: Phenol Scope 46
Products: R group Scope 56
General Procedure IV: Second Nucleophilic Displacement 69
Products: Derivatisation 70
NMR Spectra 83
HPLC Traces: 206
Single Crystal X-Ray Diffraction Data 237
Compound 1 (CCDC: 2097043) 237
Compound 37 (CCDC: 2097044) 239

General Information

Reactions were carried out under a nitrogen atmosphere in oven-dried glassware at room temperature (22 °C) unless stated otherwise. Standard inert atmosphere techniques were used in handling all air and moisture sensitive reagents. Thin-layer chromatography (TLC) was performed using Merck aluminium backed sheets coated with Merck Kieselgel 60 F254 (230-400 mesh) fluorescent treated silica, which were visualised under UV light (λmax= 254 or 365 nm). Flash column chromatography was performed using Merck Kieselgel (230-400 mesh). All ^{1}H,^{13}C,^{19}F and ^{31}P NMR spectra were recorded using a Bruker 500 MHz and Bruker 400 MHz spectrometers and are quoted in ppm for measurement against a tetramethylsilane (TMS) or residual solvent peak internal standard. Coupling constants (*J*) are reported in hertz (Hz). Two-dimensional spectroscopy (COSY, HSQC and HMBC) was used to assist in the assignment and the data is not reported. IR spectra were recorded on a Bruker Tensor 27 FT-IR spectrometer deposited as a thin film. Melting points were recorded using a Leica Galen III hot-stage microscope apparatus and are reported uncorrected in degrees Celsius (°C). Low resolution mass spectra were recorded on a Waters LCT premier XE Micromass spectrometer (ESI). High resolution mass spectra (ESI) were recorded on a Bruker MicroTof mass spectrometer. Optical rotations were recorded using a Perkin Elmer 341 polarimeter; $[\alpha]_D$ T values are reported in 10^{-1} deg·cm^{2} g^{-1} ; concentrations (c) are quoted in g/100 mL; D refers to the D-line of sodium (589 nm); temperatures (T) are given in degrees Celsius (°C). (+) and (–) compound number prefixes indicate the sign of the optical rotation. The enantiomeric excesses were determined by HPLC analysis on an Agilent 1200 Series instrument employing a chiral stationary phase column specified in the individual experiment and by comparing the samples with the appropriate racemic mixtures. Alternatively, enantiomeric excesses were deteremined using chiral SFC (supercritical fluid chromatography) separations were conducted on a Waters Acquity UPC2 system using Waters Empower Software. Chiralpak© columns (150x3 mm, particle size 3 μm) were used as specified in the text. Solvents used were of HPLC grade (Fischer Scientific, Sigma-Aldrich or Rathburn). Concentration under reduced pressure was performed by rotary evaporation at the appropriate pressure and temperature. Reagents used were obtained from commercial suppliers or purified according to standard procedures. Petroleum ether refers to distilled light petroleum of fraction 30 - 40 °C. Anhydrous toluene, tetrahydrofuran, dichloromethane and diethyl ether were dried by filtration through activated alumina (powder ~150 mesh, pore size 58 Å, basic, Sigma-Aldrich) columns. Dimethyl sulfoxide and dimethylformamide were used as supplied. Deuterated solvents were used as supplied.

Optimisation:

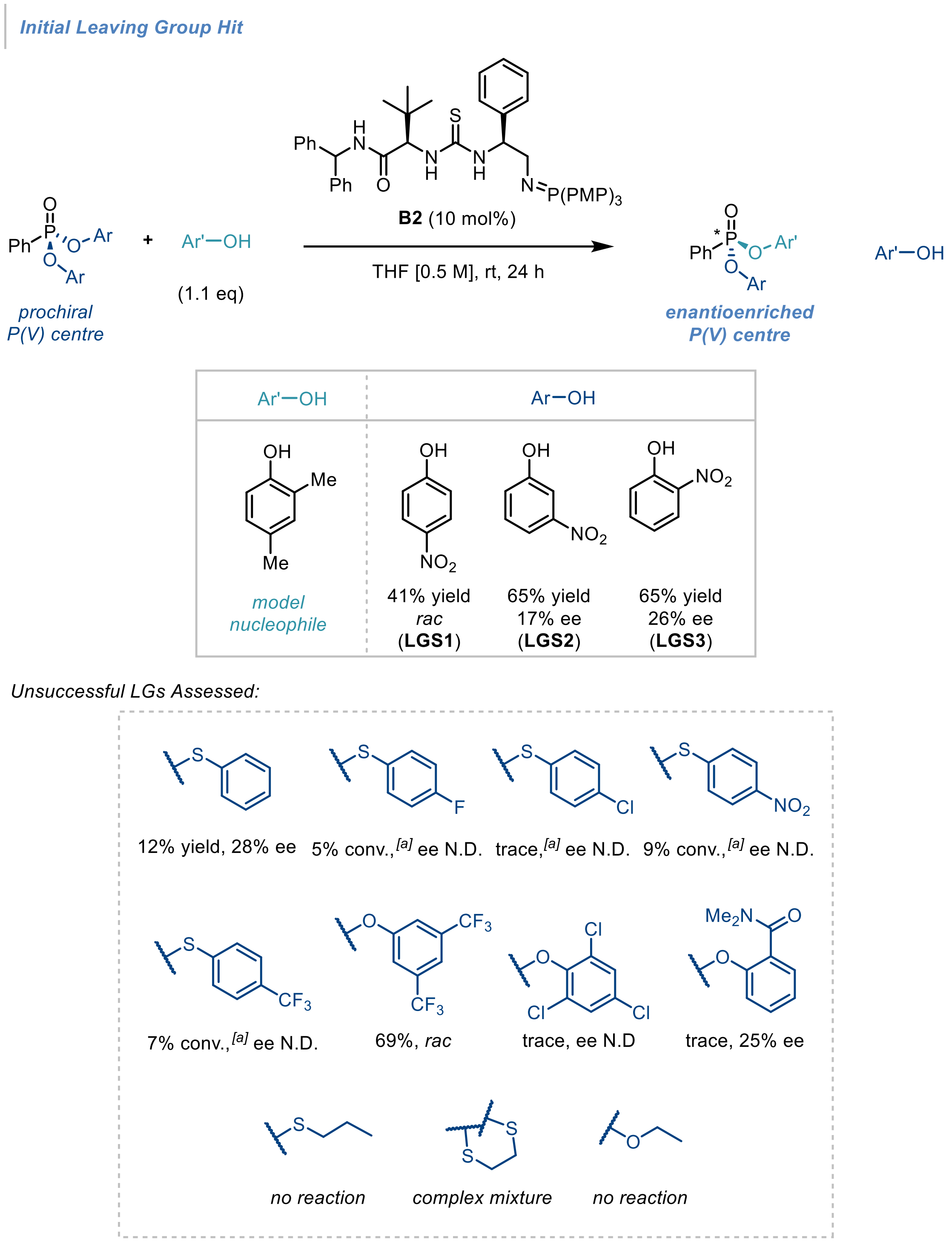

Scheme S1: Survey of possible leaving groups. [a] Conversion determined by ^{31}P NMR.

Preliminary Catalyst Screen

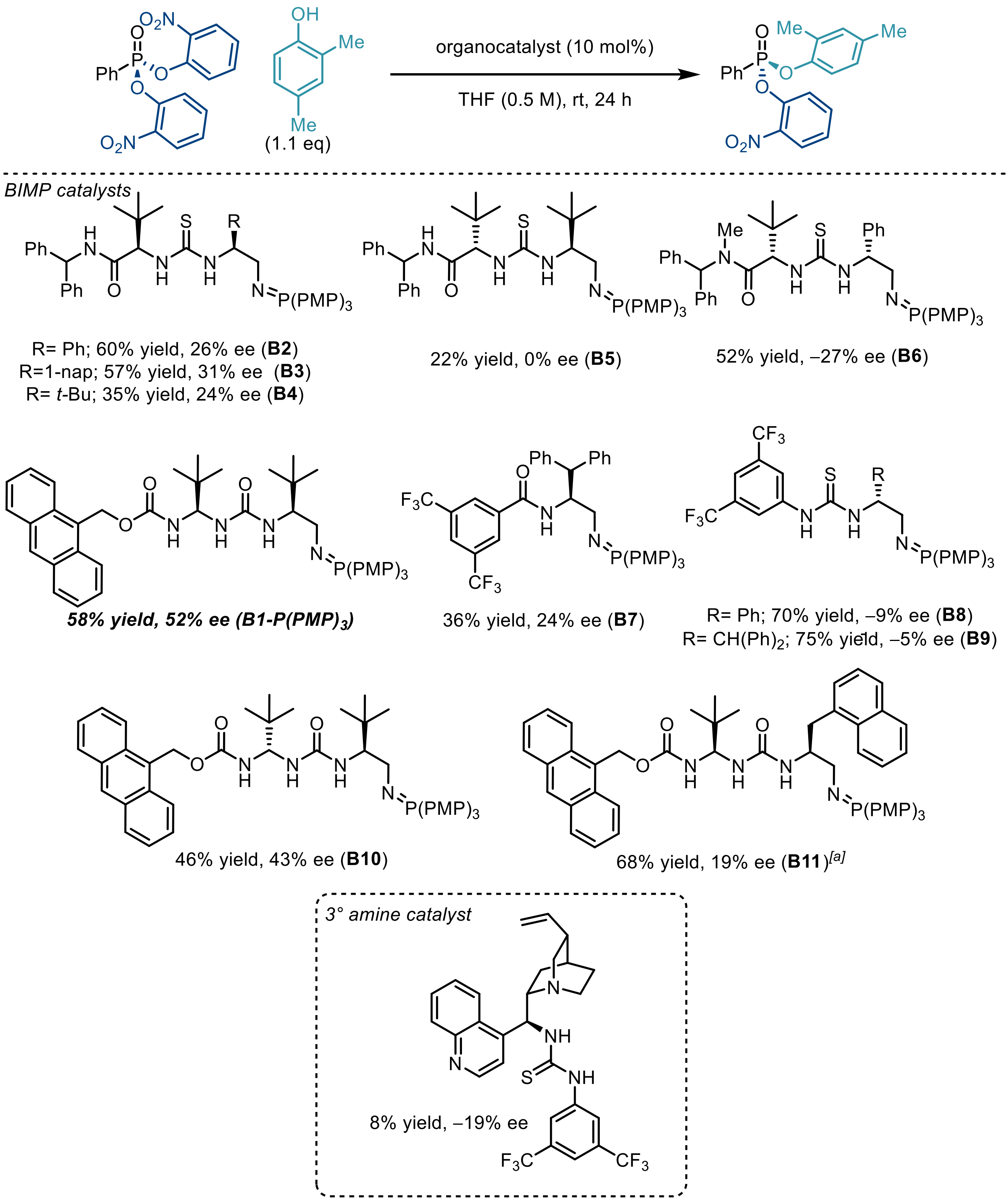

Scheme S2: Screening of catalysts. [a] Using PhF as solvent.

Phase Transfer Catalyst Screen

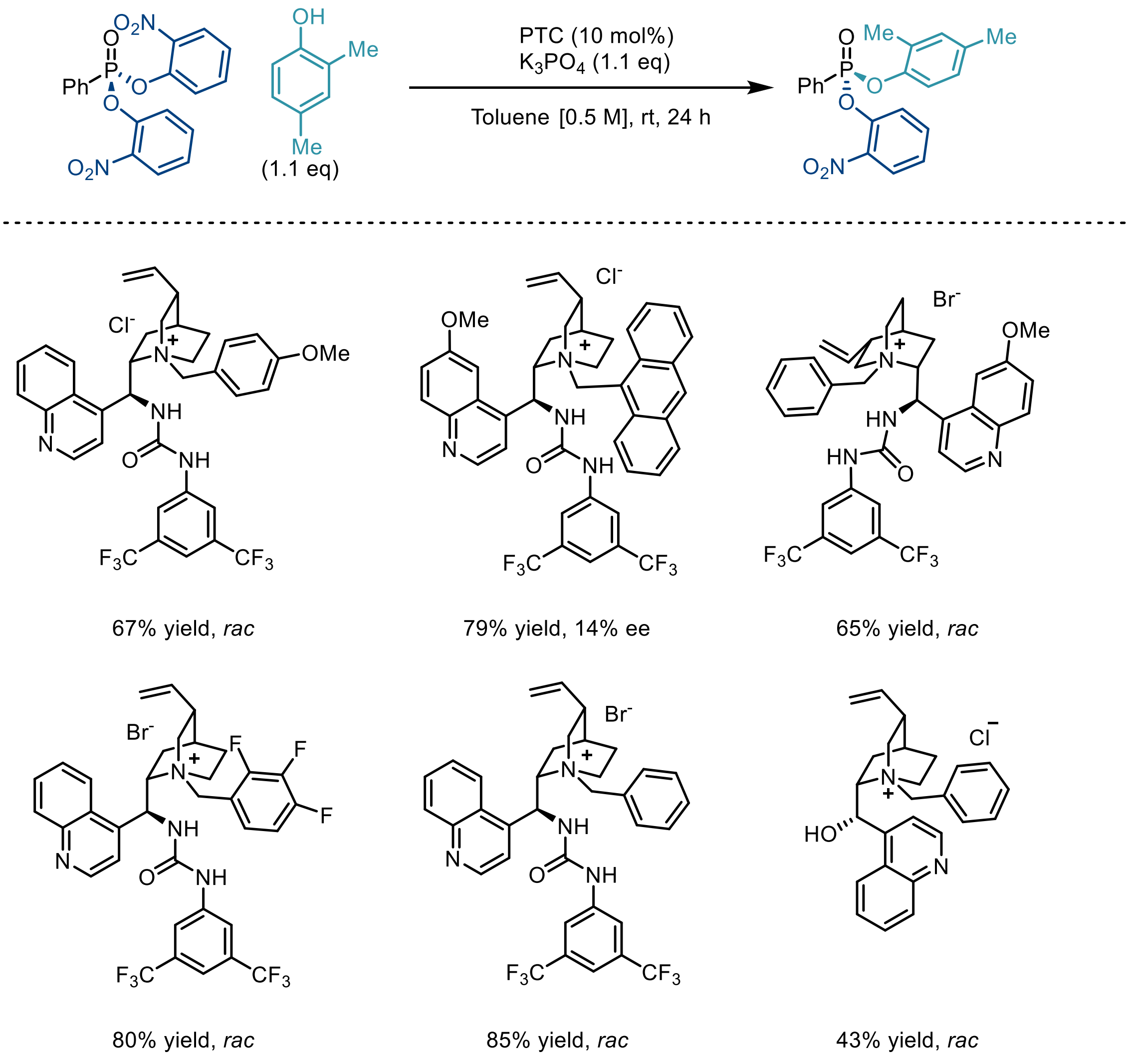

Scheme S3: Screening of phase transfer catalysts.

Solvent Screen

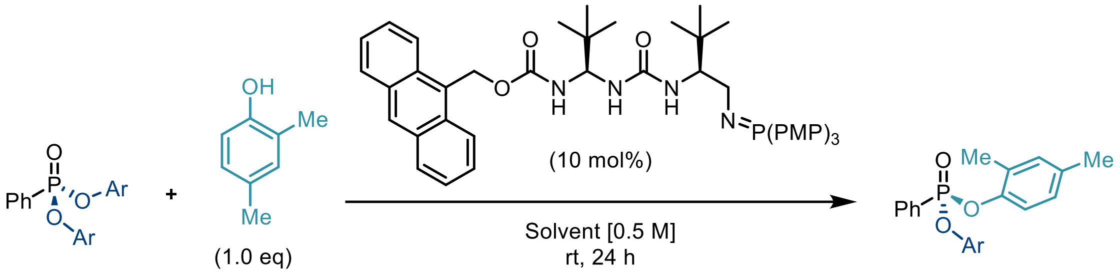

Entry	Solvent	Yield (%)	ee (%)
1	Toluene	52	60
2	PhCl	58	61
3	CPME	60	50
4	1,4-Dioxane	24	44
5	Et_2O	32	59
6	***PhF***	***63***	***67***
7	Mesitylene	60	58
8	1,2-Dichlorobenzene	78	61
9	1,2-Difluorobenzene	60	67
10	MeCN	53	3

Scheme S4: Screening of solvents with BIMP catalyst **B1-P(PMP)$_3$**. Ar= 2-$NO_2C_6H_4$.

Phosphine Screen

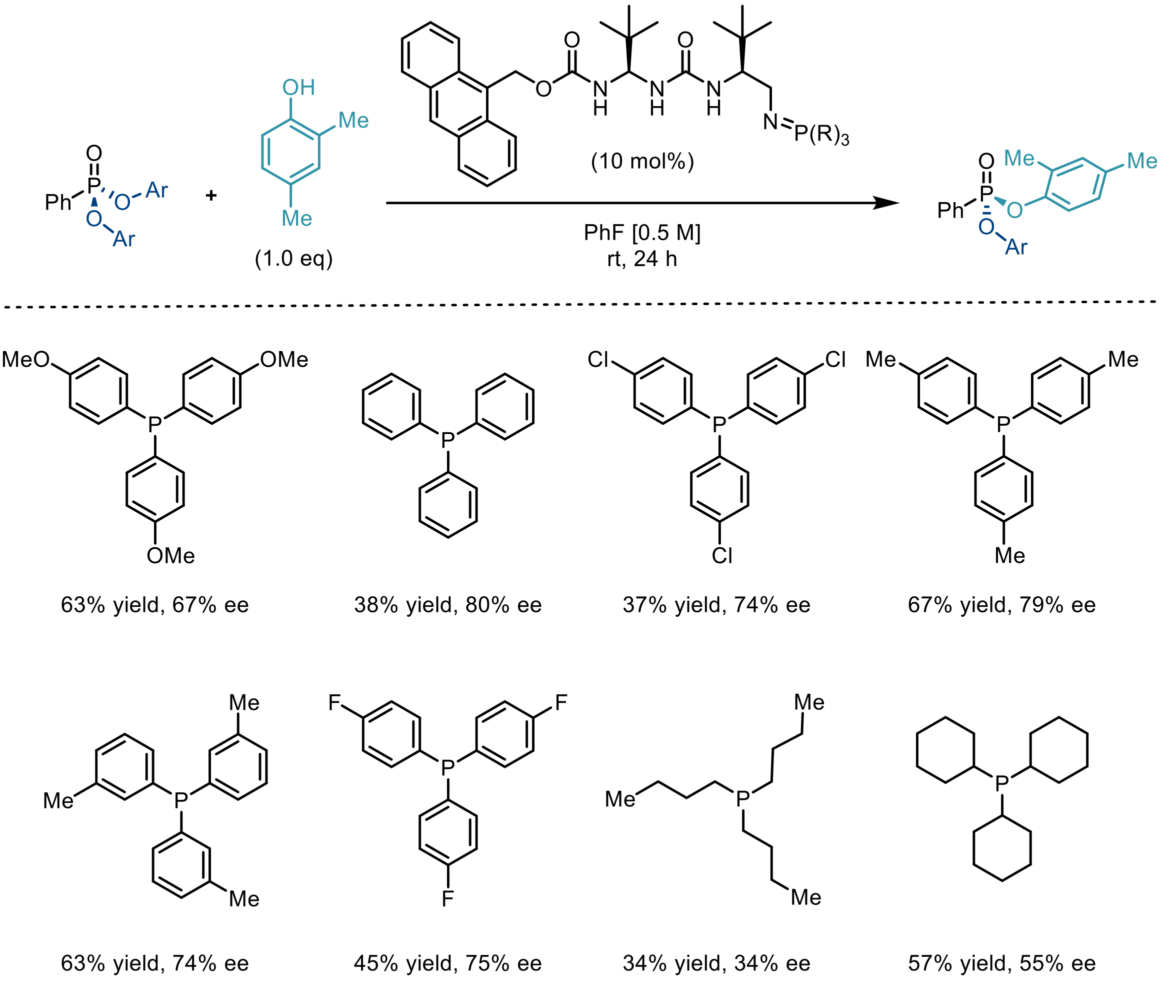

Scheme S5: Screening of phosphines to generate the iminophosphorane superbase. Ar= 2-NO_2-C_6H_4.

Miscellaneous Variables Screen

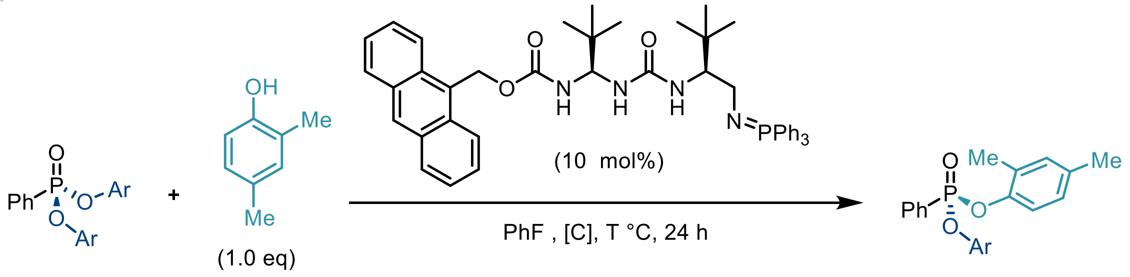

Entry	[C]	T (°C)	Additive	Yield (%)	ee (%)
1	0.5 M	rt	Li_2CO_3 (1 eq)	49	81
2	0.5 M	rt	$NaHCO_3$ (1 eq)	48	81
3	0.06 M	rt	-	54	84
4	***0.5 M***	***0***	***-***	***41***	***87***
5	0.06 M	0	-	36	86

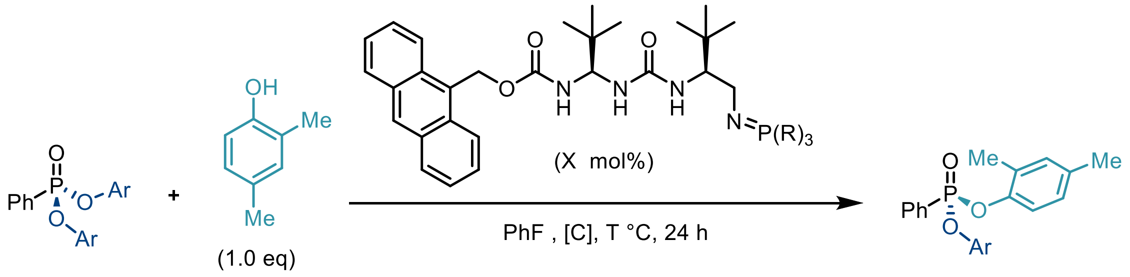

Entry	Cat. Loading	T (°C)	[C]	Phosphine	Yield (%)	ee (%)
1	10 mol%	0	0.5 M	P(*p*-tol)$_3$	52	85
2	10 mol%	-15	0.5 M	P(*p*-tol)$_3$	14	90
3	15 mol%	-15	0.5 M	P(*p*-tol)$_3$	22	89
4	10 mol%	-15	0.5 M	PPh_3	13	88
5	15 mol%	-15	0.5 M	PPh_3	19	88
6	10 mol%	-15	0.25 M	P(*p*-tol)$_3$	16	89
7	10 mol%	-15	0.25 M	PPh_3	15	88

Scheme S6: Further optimisation of reaction conditions. Ar= 2-NO_2-C_6H_4.

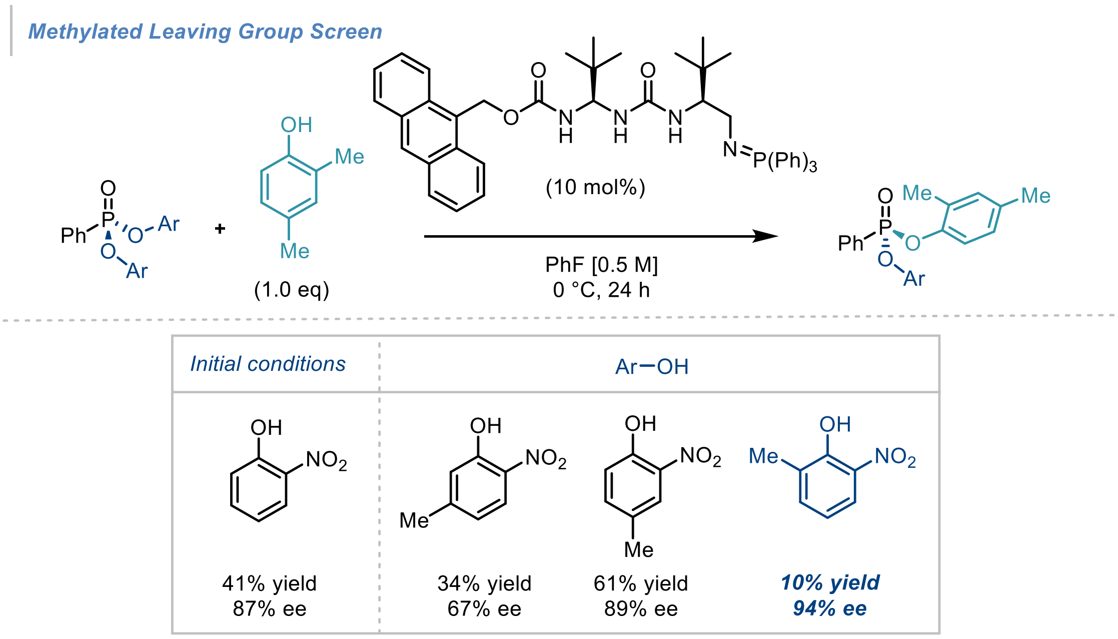

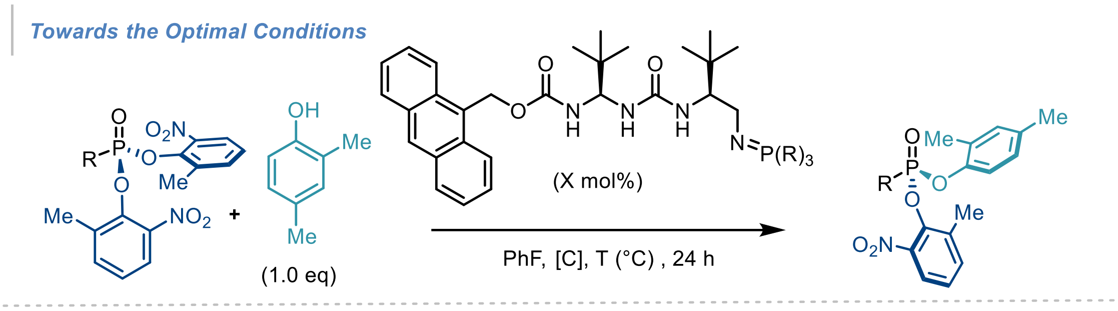

Entry	Cat. Loading	T (°C)	[C]	Phosphine	Yield (%)	ee (%)
1	10 mol%	0	0.5 M	PPh_3	10	94
2	10 mol%	rt	0.25 M	P(*p*-tol)$_3$	56	93
3	10 mol%	rt	0.5 M	PPh_3	46	92
4	10 mol%	rt	0.25 M	PPh_3	46	92
5	15 mol%	rt	0.25 M	P(*p*-tol)$_3$	66	92.5
6	15 mol% (1.5 eq Nuc)	rt	0.25 M	P(*p*-tol)$_3$	66	92.5
7	15 mol%	rt	0.16 M	P(*p*-tol)$_3$	59	92
8	15 mol% (PhF over 4 Å MS)	rt	0.25 M	P(*p*-tol)$_3$	***84***	***91***

Scheme S7: Survey of methylated leaving groups (top) and final optimisation of reaction conditions (bottom).

Unsuccessful Nucleophiles

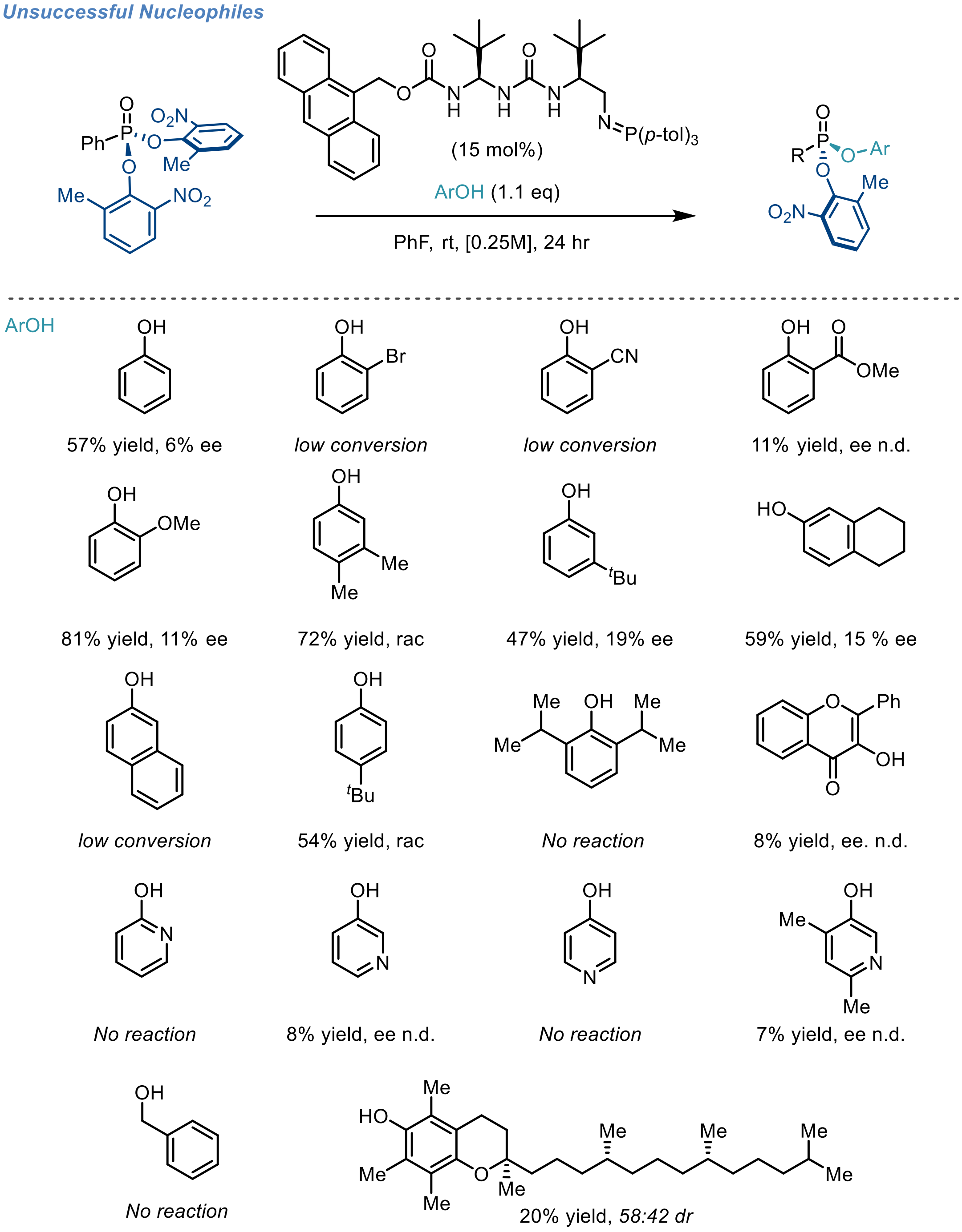

Scheme S8: Unsuccessful alcohol nucleophiles.

Further Unsuccessful Electrophiles

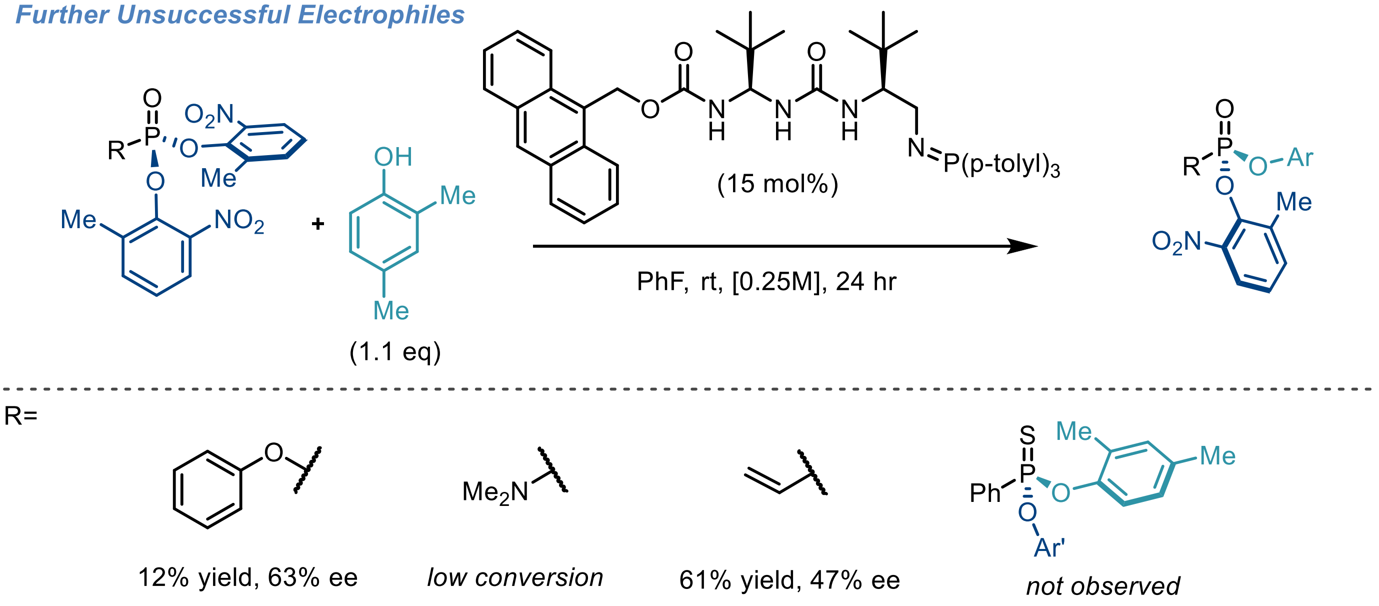

Scheme S9: Further unsuccessful P(V) electrophiles. Ar= 2,4-dimethyl phenol. Ar'= 2-(NO_2),4-(CH_3)-C_6H_3

Computational Studies

General Computational Methods

Calculations were performed using Gaussian 16 A.03.[1] Geometry optimisations of all structures were carried out with the M06-2X meta-generalized gradient approximation (GGA) functional in combination with the def2SVP basis set ("loose" optimisation criteria).[2,3] The effect of fluorobenzene solvent was evaluated using the SMD implicit solvent model.[4] Harmonic vibrational frequencies at the same level of theory were calculated to characterize stationary points as either minima or transition state (TS) structures and to calculate the zero-point vibrational energy and thermal corrections. Free energies were evaluated at 25 °C and have been corrected to a standard liquid state of 1 mol/L. In all cases, vibrational entropies were obtained using a quasi-harmonic approximation, treating vibrational modes below 100 cm^{-1} as free rotors and as rigid rotors above this cut off, as first proposed by Grimme[5] and implemented in Python.[6] Single point energies were evaluated at the SMD(fluorobenzene)-M06-2X/def2TZVP level of theory. Molecular graphics were generated with CYLview20 and PyMol.[7,8] The NCIPLOT4 was used to visualise the non-covalent interactions.[9,10,11]

---

[1] Gaussian 16, Revision A.03, M. J. Frisch, G. W. Trucks, H. B. Schlegel, G. E. Scuseria, M. A. Robb, J. R. Cheeseman, G. Scalmani, V. Barone, G. A. Petersson, H. Nakatsuji, X. Li, M. Caricato, A. V. Marenich, J. Bloino, B. G. Janesko, R. Gomperts, B. Mennucci, H. P. Hratchian, J. V. Ortiz, A. F. Izmaylov, J. L. Sonnenberg, D. Williams-Young, F. Ding, F. Lipparini, F. Egidi, J. Goings, B. Peng, A. Petrone, T. Henderson, D. Ranasinghe, V. G. Zakrzewski, J. Gao, N. Rega, G. Zheng, W. Liang, M. Hada, M. Ehara, K. Toyota, R. Fukuda, J. Hasegawa, M. Ishida, T. Nakajima, Y. Honda, O. Kitao, H. Nakai, T. Vreven, K. Throssell, J. A. Montgomery, Jr., J. E. Peralta, F. Ogliaro, M. J. Bearpark, J. J. Heyd, E. N. Brothers, K. N. Kudin, V. N. Staroverov, T. A. Keith, R. Kobayashi, J. Normand, K. Raghavachari, A. P. Rendell, J. C. Burant, S. S. Iyengar, J. Tomasi, M. Cossi, J. M. Millam, M. Klene, C. Adamo, R. Cammi, J. W. Ochterski, R. L. Martin, K. Morokuma, O. Farkas, J. B. Foresman, and D. J. Fox, Gaussian, Inc., Wallingford CT, 2016.

[2] Zhao, Y.; Truhlar, D.G. *Theor. Chem. Acc.*, **2008**, *120*, 215-41.

[3] Weigend, F.; Ahlrichs, R. Phys. *Chem. Chem. Phys.* **2005**, *7*, 3297.

[4] Marenich, A. V.; Cramer, C. J.; Truhlar, D. G *J. Phys. Chem. B* **2009**, *113*, 6378–6396.

[5] Grimme, S. *Chem. Eur. J.* **2012**, *18*, 9955–9964.

[6] Funes-Ardoiz, I.; Paton, R. S. GoodVibes v2.0.2 DOI: 10.5281/zenodo.595246.

[7] CYLview20; Legault, C. Y., Unversité de Sherbrooke: Sherbrooke, **2020** (http://www.cylview.org).

[8] Schrödinger, L., & DeLano, W. (2020). PyMOL. Retrieved from http://www.pymol.org/pymol.

[9] Boto, R. A.; Peccati, F.; Laplaza, R.; Quan, C.; Carbone, A.; Piquemal, J.-P.; Maday, Y.; Contreras-García, J. *J. Chem. Theory Comput.* **2020**, *16*, 4150–4158.

[10] Johnson, E. R.; Keinan, S.; Mori-Sanchez, P.; Contreras-Garcia, J.; Cohen, A. J.; Yang, W. *J. Am. Chem. Soc.* **2010**, *132*, 6498–6506.

[11] Contreras-Garcia, J.; Johnson, E. R.; Keinan, S.; Chaudret, R.; Piquemal, J.-P.; Beratan, D. N.; Yang. W. *J. Chem. Theory Comput.* **2011**, *7*, 625–632.

A structurally simplified version of the BIMP catalyst **B12-P(Ph)₃**, for which enantioselectivity has been measured experimentally, was used to construct the catalytic reaction profile (**Figure S1**). Given that the chiral backbone, the basic iminophosphorane site, and the *N*-substituent on the distal portion of the BIMP structure were essential for achieving the high reactivity and enantioselectivity in the P(V) desymmetrisation step, these components were conserved in BIMP **B12-P(Ph)$_3$**. In this catalyst, the anthracenyl motif was replaced with a phenyl ring and the phenyl groups instead of tolyl groups were used on the iminophosphorane moiety. The energy differences of the TSs for nucleophilic attack with other structurally simplified BIMP catalysts were also computed. Subsequently these catalysts were synthesised and tested experimentally and the empirical enantioselectivity data were in keeping with the computational trend. The transition states with **B12-P(*p*-tol)$_3$** and **B12-P(Bu)$_3$** are not computed due to the structural similarity with **B12-P(Ph)$_3$** and **B12-P(Me)$_3$**, respectively.

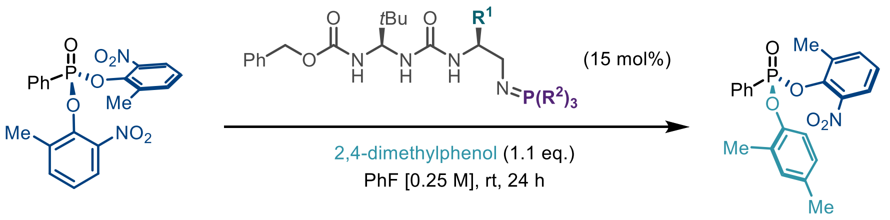

BIMP catalyst	R^1	R^2	yield (%)	ee (%)	ΔΔG‡ (kcal mol^{-1})
**B12-P(*p*-tol)$_3$**	^{t}Bu	*p*-tolyl	79	92	---
**B12-P(Ph)$_3$**	^{t}Bu	Ph	76	92	2.4
**B12-P(Bu)$_3$**	^{t}Bu	Bu	60	75	---
**B12-P(Me)$_3$**	^{t}Bu	Me	20	47	1.8
**B13-P(Ph)$_3$**	Me	Ph	82	3	0.6

$\Delta\Delta G^{‡} = \Delta G^{‡}_{\text{TS1-}(R)} - \Delta G^{‡}_{\text{TS1-}(R)}$

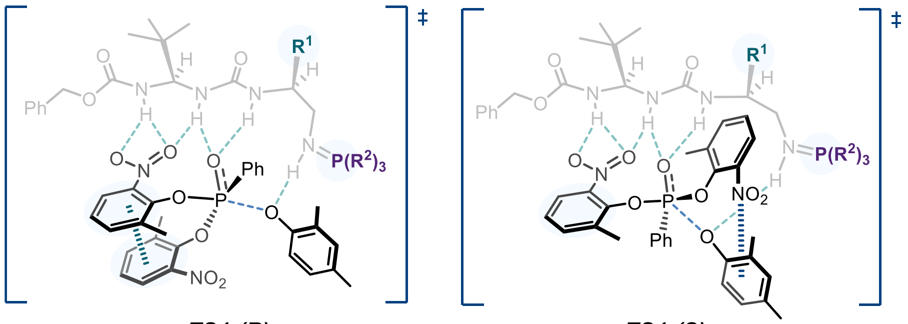

TS1-(R) ***TS1-(S)***

Figure S1. Comparison of empirical and computed enantioselectivity between different BIMP catalysts.

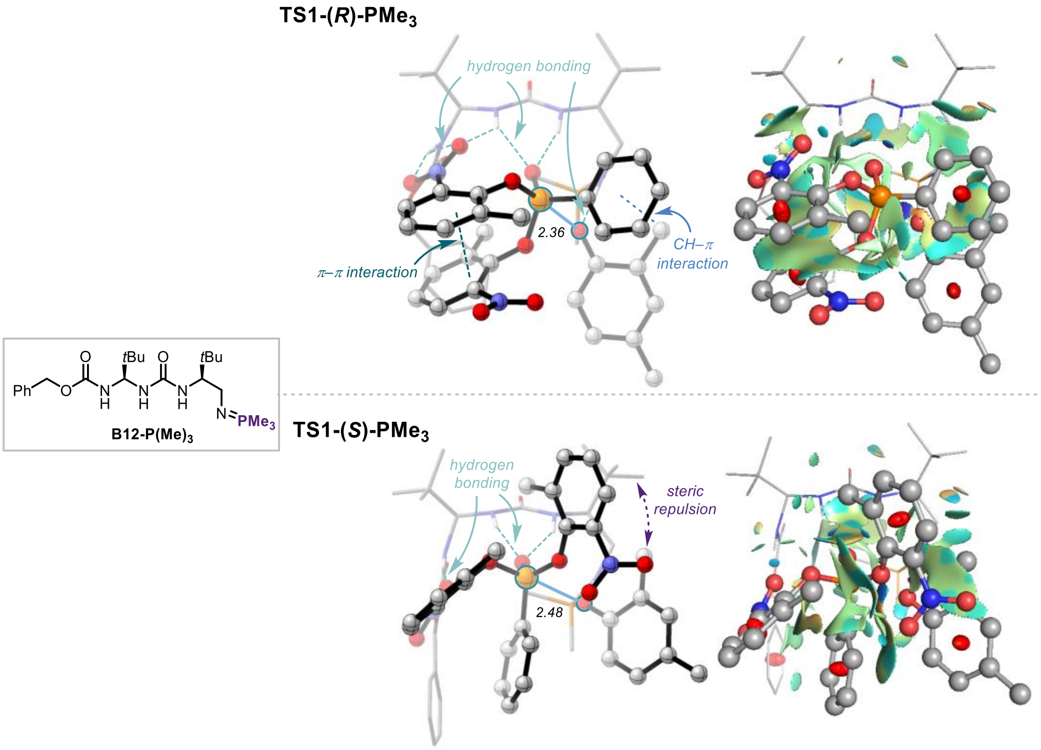

Figure S2. Nucleophilic attack transition state structures and the NCI surfaces with **B12-P(Me)3**. Bond lengths (Å) of the TS geometries are provided in the insert.

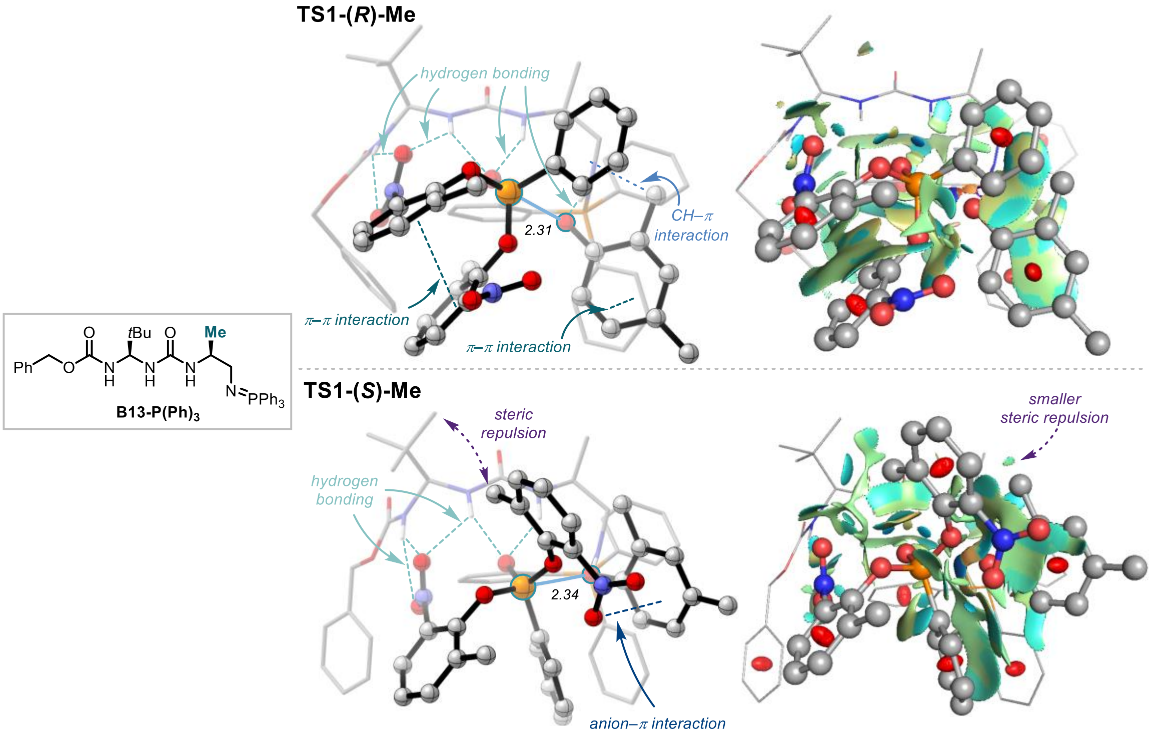

Figure S3. Nucleophilic attack transition state structures and the NCI surfaces with **B13-P(Ph)3**. Bond lengths (Å) of the TS geometries are provided in the insert.

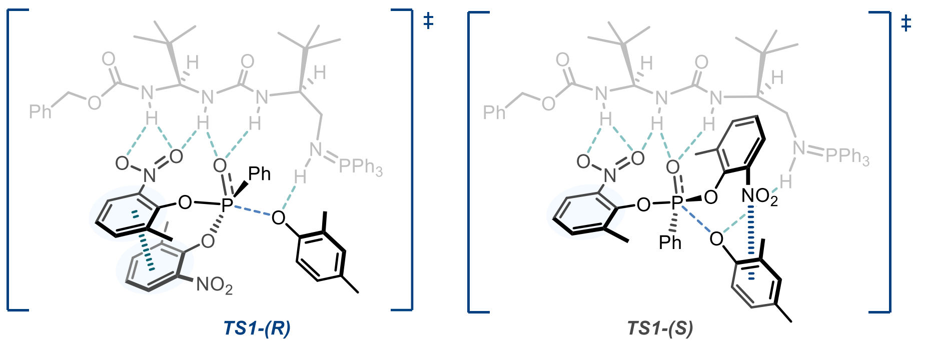

TS1-(R) *TS1-(S)*

calculations	ΔΔ$G^{‡}$ (kcal mol^{-1})
SMD(fluorobenzene)-M06-2X/def2TZVP//SMD(fluorobenzene)-M06-2X/def2SVP	2.4
SMD(fluorobenzene)-BLYP-D3/def2TZVP//SMD(fluorobenzene)-M06-2X/def2SVP	2.6
SMD(fluorobenzene)-B3LYP-D3BJ/def2TZVP//SMD(fluorobenzene)-M06-2X/def2SVP	1.9
SMD(fluorobenzene)-ωB97X-D/def2TZVP//SMD(fluorobenzene)-M06-2X/def2SVP	2.2
SMD(fluorobenzene)-M11/def2TZVP//SMD(fluorobenzene)-M06-2X/def2SVP	2.2

Figure S4. Computed Gibbs free energy differences (ΔΔ$G^{‡}$ [kcal mol^{-1}]) for the initial nucleophilic attack (**TS1-(*R*)** and **TS1-(*S*)**) with various functionals.

Table S1. Electronic energies (*E* in Hartrees), enthalpies (*H* in Hartrees), quasi-harmonic Gibbs free energies (*qh-G* in Hartrees) and imaginary frequencies (in *i* cm^{-1}) of all stationary points computed at SMD(fluorobenzene)/M06-2X/def2SVP level of theory. Electronic energies (E_{high} in Hartrees) computed at the SMD(fluorobenzene)/M06-2X/def2TZVP//SMD(fluorobenzene)/M06-2X/def2SVP level of theory.

structure	*E*	*H*	*qh-G*	E^{high}	*Imag. Freq.*
**SM**	-1747.972783	-1747.593091	-1747.669756	-1749.712772	-
**Ar'OH**	-385.658047	-385.487591	-385.527586	-386.091299	-
**B12-P(Ph)$_3$**	-2260.150209	-2259.312686	-2259.425415	-2262.406804	-
**RC-(*R*)**	-4393.825854	-4392.435166	-4392.620055	-4398.233493	-
**RC-(*S*)**	-4393.825460	-4392.434570	-4392.621241	-4398.231444	-
**TS1-(*R*)**	-4393.821200	-4392.431933	-4392.614819	-4398.225576	138.9*i*
**TS1-(*S*)**	-4393.817343	-4392.427350	-4392.610918	-4398.221850	159.8*i*
**INT-(*R*)**	-4393.835220	-4392.444323	-4392.629936	-4398.242413	-
**INT-(*S*)**	-4393.834389	-4392.441790	-4392.625905	-4398.234956	-
**TS2-(*R*)**	-4393.834895	-4392.443701	-4392.627096	-4398.237837	24.7*i*
**TS2-(*S*)**	-4393.828322	-4392.43701	-4392.620330	-4398.231189	118.9*i*
**Product**	-1582.969503	-1582.566677	-1582.642487	-1584.513225	-
**ArOH**	-550.672070	-550.524721	-550.565989	-551.303478	-
**TS1-(*R*)-PMe$_3$**	-3819.245953	-3818.028384	-3818.193343	-3823.037672	120.8*i*
**TS1-(*S*)-PMe$_3$**	-3819.239102	-3818.021622	-3818.188288	-3823.033081	57.2*i*
**TS1-(*R*)-Me**	-4276.029066	-4274.727553	-4274.902791	-4280.302311	145.5*i*
**TS1-(*S*)-Me**	-4276.027534	-4274.726133	-4274.903455	-4280.299130	156.2*i*

Synthesis of Catalyst B1:

(*S*)-2-(((anthracen-9-ylmethoxy)carbonyl)amino)-3,3-dimethylbutanoic acid (**S1**)

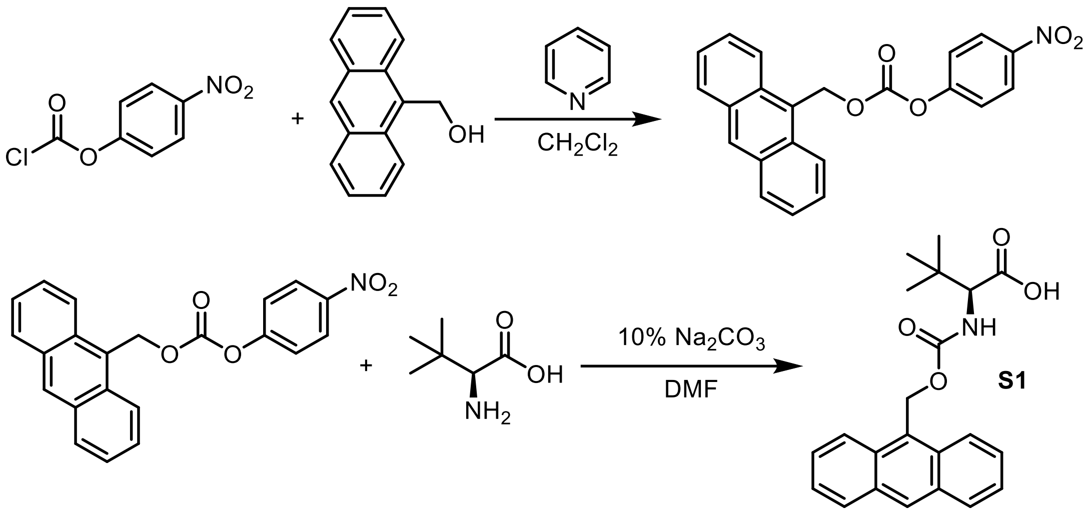

According to a literature procedure,[12] to a solution of 4-nitrophenylchloroformate (2.2 g, 11 mmol, 1.1 eq) in CH_2Cl_2 (14 mL) at rt was added pyridine (0.90 mL, 11 mmol, 1.1 eq) dropwise. The slurry was cooled to 0 °C and 9-anthracenemethanol (2.08 g, 10 mmol, 1.0 eq) was added portionwise. The reaction mixture was then allowed to warm to rt and stirring maintained overnight. The reaction mixture was diluted with CH_2Cl_2 (40 mL) and washed with 1 M HCl (20 mL), water (20 mL) and brine (20 mL) and dried ($MgSO_4$). The volatiles were removed *in vacuo* to afford a yellow solid in 60% yield (2.23g) which was used without further purification.

To a stirred solution of L-*tert*-Leucine (0.66 g, 5.0 mmol, 1.0 eq) in 10% aqueous Na_2CO_3 (13 mL) and DMF (10 mL) at 0 °C was slowly added the 4-nitrophenylcarbonate (1.87 g, 5.0 mmol, 1.0 eq) in DMF (15 mL). After stirring for one hour at this temperature, the reaction mixture was allowed to warm to rt overnight, diluted by the addition of H_2O (50 mL) and extracted with Et_2O (3 x 20 mL). The aqueous layer was cooled in an ice bath and acidified to pH 1 by the addition of concentrated HCl and then extracted with EtOAc (3 x 25 mL). The combined organics were washed (brine), dried ($MgSO_4$) and the volatiles removed *in vacuo*. Purification by flash column chromatography [Petroleum ether to EtOAc] afforded the title compound as a pale-yellow solid in 60% yield (1.08 g).

Mp 153 – 154 °C; **^{1}H NMR** (400 MHz, $CDCl_3$) δ ppm 1.00 (s, 9 H), 4.27 (d, *J* = 10.0 Hz, 1 H), 5.31 (d, *J* = 10.0 Hz, 1 H), 6.13 (d, *J* = 12.5 Hz, 1 H), 6.20 (d, *J* = 12.5 Hz, 1 H), 7.44 - 7.61 (m, 4 H), 8.01 (d, *J* = 8.5 Hz, 2 H), 8.37 (d, *J* = 8.5 Hz, 2 H), 8.49 (s, 1 H); **^{13}C NMR** (100 MHz, $CDCl_3$) δ ppm 26.6, 34.7, 59.8, 62.4, 124.1, 125.2, 126.4, 126.8, 129.2, 129.3, 131.2, 131.5, 156.6, 176.4. Data was consistent with the literature.

---

[12] Diosdado, S.; Etxabe, J.; Izquierdo, J.; Landa, A.; Mielgo, A.; Olaizola, I.; López, R.; Palomo, C. *Angew. Chem. Int. Ed.* **2013**, *52*, 11846-11851.

Anthracen-9-ylmethyl [(1*S*)-1-({[(2*S*)-1-azido-3,3-dimethylbutan-2-yl]carbamoyl}amino)-2,2-dimethylpropyl]carbamate (**B1**)

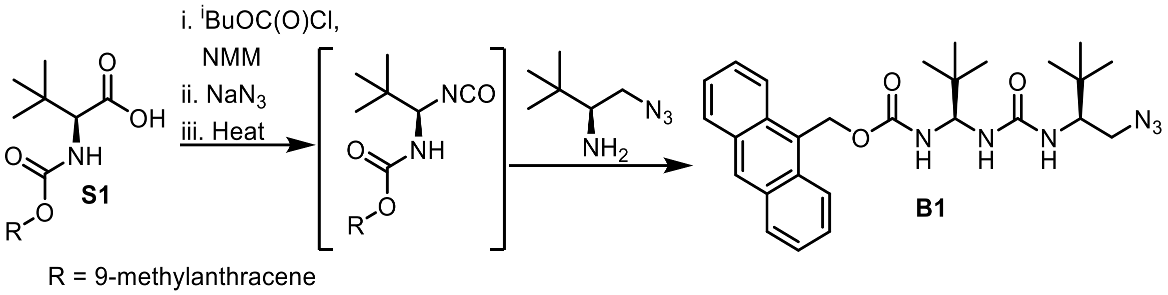

According to a modified literature procedure,[1] to a solution of **S1** (365 mg, 1.0 mmol, 1.0 eq.) in THF (4 mL) at −20 °C were added sequentially isobutyl chloroformate (130 µL, 1.0 mmol, 1.0 eq.) and *N*-methylmorpholine (110 µL, 1.0 mmol, 1.0 eq.) and stirring was maintained for 20 min. To the reaction mixture was added sodium azide (98 mg, 1.5 mmol, 1.5 eq) in H_2O (1.0 mL) and stirring was maintained for 30 min at −20 °C. The organic layer was then separated, the volatiles removed *in vacuo*, dissolved in CH_2Cl_2 (15 mL) and washed with water (10 mL). The organic layer was dried ($MgSO_4$) and concentrated *in vacuo* to approximately 5 mL. The reaction mixture was heated to reflux for 2 h and then the reaction mixture was cooled to rt and the crude amino azide[13] (156 mg, 1.00 mmol, 1.00 eq) as a solution in Et_2O (4 mL) was added dropwise. The reaction mixture was stirred at rt overnight and then the volatiles removed *in vacuo*. The crude was triturated in toluene at 100 °C, cooled to rt, filtered and washed with pentane to obtain the title compound as a colourless solid in 40% yield (200 mg).

[α]$_D$^{25} = +20.8 (c = 0.91, $CHCl_3$); **Mp** 268 – 270 °C; **^{1}H NMR** (400 MHz, DMSO-d_6) δ 8.67 (s, 1H), 8.39 (d, *J* = 8.8 Hz, 2H), 8.13 (d, *J* = 8.3 Hz, 2H), 7.57 (dt, *J* = 21.4, 7.1 Hz, 4H), 7.36 (d, *J* = 9.2 Hz, 1H), 6.08 (dq, *J* = 34.3, 12.3, 10.8 Hz, 4H), 5.26 (t, *J* = 9.5 Hz, 1H), 3.60 (dt, *J* = 9.7, 4.8 Hz, 1H), 3.43 (dd, *J* = 12.8, 3.6 Hz, 1H), 3.17 (dd, *J* = 12.7, 8.8 Hz, 1H), 0.84 (d, *J* = 5.6 Hz, 18H); **^{13}C NMR** (101 MHz, DMSO-d_6) δ 156.9, 155.7, 130.9, 130.5, 128.9, 128.5, 127.4, 126.5, 125.2, 124.2, 64.8, 57.8, 56.7, 51.9, 36.2, 34.3, 26.4, 25.3; **IR** ν_{max}/cm^{-1}: 3658, 3376, 2977, 2887, 2172, 1667, 1385, 1259, 1004; **HRMS** (ESI+): calcd. for $C_{28}H_{36}N_6NaO_3$ $[M+Na]^+$ 527.2741, found 527.2742.

[13] M. G. Núñez, A. J. M. Farley, D. J. Dixon, *J. Am. Chem. Soc.* **2013**, *135*, 16348–16351.

Synthesis of Catalyst B12:

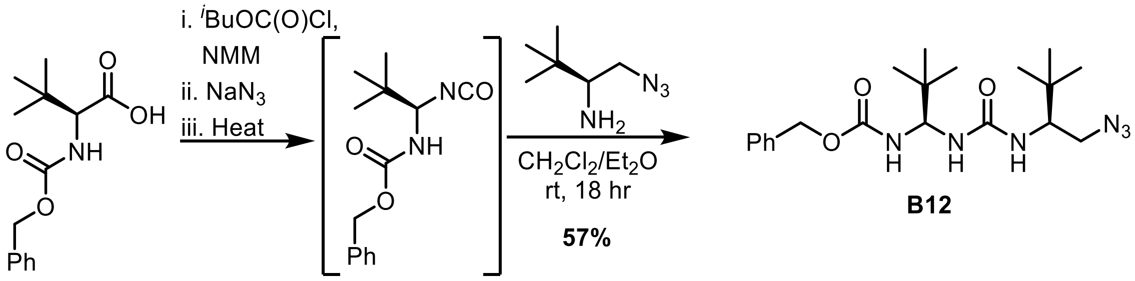

To a solution of **Cbz-L-(S)-*tert*-Leucine** (265 mg, 1 mmol, 1 eq.) in THF (4 mL) at −20 °C were added sequentially isobutyl chloroformate (130 µL, 1 mmol, 1 eq.) and *N*-methylmorpholine (110 µL, 1 mmol, 1 eq) and stirring was maintained for 20 min. To the reaction mixture was added sodium azide (98.0 mg, 1.5 mmol, 1.5 eq) in H_2O (1 mL) and stirring was maintained for 30 min at −20 °C. The organic layer was then separated, the volatiles removed *in vacuo*, dissolved in CH_2Cl_2 (15 mL) and washed with water (10 mL). The organic layer was dried ($MgSO_4$) and concentrated *in vacuo* to 5 mL. The reaction mixture was heated to reflux for 2 hr and then the reaction mixture was cooled to room temperature and the crude amino azide (142 mg, 1 mmol, 1.0 eq) as a solution in Et_2O (4 mL) was added dropwise. The reaction mixture was stirred at room temperature for 18 hr and then the volatiles removed *in vacuo*. The crude material was triturated from hot toluene (5 mL), cooled, filtered, and washed with pentane (5 mL) and diethyl ether (5 mL) to obtain **B12** as a white solid in 57% yield (230 mg).

$[\alpha]_D^{25}$ = -11.4 (c = 0.27, DMSO); **Mp** 278 °C (toluene); **^{1}H NMR** (400 MHz, DMSO-*d*$_6$); δ 7.44 (d, *J* = 9.2 Hz, 1H), 7.38 – 7.27 (m, 5H), 6.12 (appt, *J* = 9.6 Hz, 2H), 5.15 (t, *J* = 9.4 Hz, 1H), 5.07 – 4.97 (m, 2H), 3.57 (td, *J* = 9.2, 3.6 Hz, 1H), 3.43 (dd, *J* = 12.7, 3.6 Hz, 1H), 3.17 (dd, *J* = 12.7, 9.0 Hz, 1H), 0.84 (d, *J* = 1.6 Hz, 18H); **^{13}C NMR** (101 MHz, DMSO-*d*$_6$) δ 156.9, 155.4, 137.2, 128.3, 127.7(2x), 65.1, 64.7, 56.7, 51.9, 36.1, 34.3, 26.4, 25.3; **IR** ν_{max}/cm^{-1}: 3314, 2969, 2095, 1638(C=O), 1550(C=O), 1244, 729, 697; **HRMS** (ESI+): calcd. for $C_{20}H_{33}N_6O_3$ $[M+H]^+$ 405.2609, found 405.2611.

Synthesis of Catalyst B13:

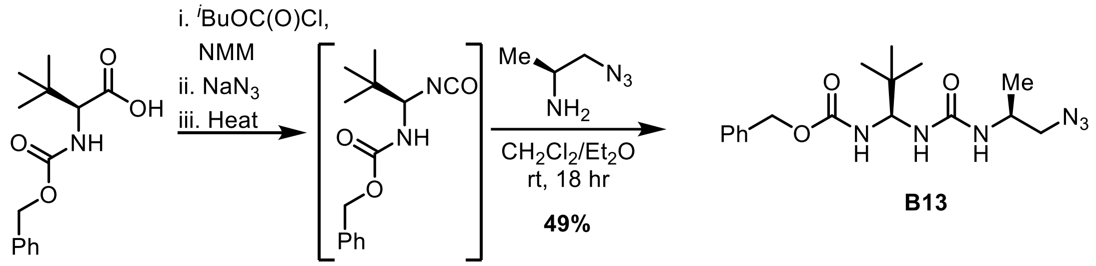

To a solution of **Cbz-L-(S)-*tert*-Leucine** (265 mg, 1 mmol, 1 eq) in THF (4 mL) at −20 °C were added sequentially isobutyl chloroformate (130 µL, 1 mmol, 1 eq) and *N*-methylmorpholine (110 µL, 1 mmol, 1 eq) and stirring was maintained for 20 min. To the reaction mixture was added sodium azide (98.0 mg, 1.5 mmol, 1.5 eq) in H_2O (1 mL) and stirring was maintained for 30 min at −20 °C. The organic layer was then separated, the volatiles removed *in vacuo*, dissolved in CH_2Cl_2 (15 mL) and washed with water (10 mL). The organic layer was dried ($MgSO_4$) and concentrated *in vacuo* to 5 mL. The reaction mixture was heated to reflux for 2 hr and then the reaction mixture was cooled to room temperature and the crude amino azide (100 mg, 1 mmol, 1.0 eq) as a solution in Et_2O (4 mL) was added dropwise. The reaction mixture was stirred at room temperature for 18 hr and then the volatiles removed *in vacuo*. The crude material was purified by column chromatography (gradient pentane/ethyl acetate) to obtain **B13** as a white foam in 49% yield (178 mg).

$[\alpha]_D^{25}$ = −25.2 (c = 1.0, DMSO); **^{1}H NMR** (400 MHz, DMSO-*d*$_6$) δ 7.41 – 7.33 (m, 5H), 6.15 (d, *J* = 7.7 Hz, 1H), 6.01 (d, *J* = 9.4 Hz, 1H), 5.10 (t, *J* = 9.2 Hz, 1H), 5.06 – 4.95 (m, 2H), 3.75 (ddt, *J* = 7.7, 6.7, 5.2 Hz, 1H), 3.35 – 3.29 (m, 2H), 1.04 (d, *J* = 6.7 Hz, 3H), 0.84 (s, 9H); **^{13}C NMR** (151 MHz, DMSO-*d*$_6$) δ 156.3, 155.4, 137.3, 137.2, 128.3, 127.7, 65.1, 64.7, 55.7, 44.8, 35.8, 25.3, 18.3; **IR** ν_{max}/cm^{-1}: 3366, 3034, 2966, 2101, 1693(C=O), 1531(C=O), 1267, 1048, 696; **HRMS** (ESI+): calcd. for $C_{17}H_{27}N_6O_3$ $[M+H]^+$ 363.2139, found 363.2153.

Substrate Synthesis:

General Procedure I: Synthesis of P(V) desymmetrisation substrates

General Procedure IA: Substrate synthesis from phosphoryl dichloride:

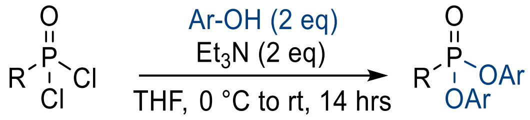

According to a modified literature procedure.[14] The corresponding phosphonic dichloride (4.00 mmol) and the corresponding nucleophile (8.00 mmol) were dissolved in THF (40 mL) and cooled to 0 °C. Et_3N (800 mg, 8 mmol, 1.10 mL) was added dropwise resulting in the formation of a white precipitate. The reaction mixture was then allowed to warm to rt and stirred for 14 hrs. The reaction mixture was filtered, and the solvent was removed *in vacuo*. The resulting crude was dissolved in CH_2Cl_2 (40 mL) and was washed sequentially with 1M aq. NaOH (15 mL), brine (15 mL), 1M HCl (15 mL) and brine (15 mL). The organic phase was then dried over $MgSO_4$, filtered and the solvent was removed under reduced pressure. The resulting crude product was purified by FCC as described in the individual experiment and subsequently triturated with pentane to afford the pure phosphonate ester.

General Procedure IB: Substrate synthesis from phosphonic acid:

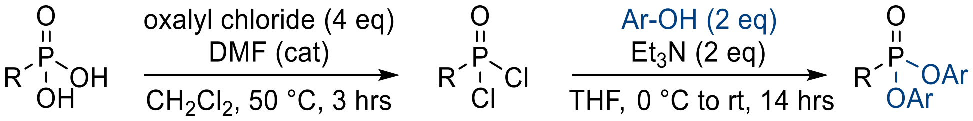

According to a modified literature procedure.[15] The corresponding phosphonic acid (4.00 mmol) was dissolved in CH_2Cl_2 (8 mL) and a catalytic amount of DMF (0.1 mL) was added. The solution was heated to 50 °C and oxalyl chloride (1.36 mL, 16 mmol) was added dropwise over 30 min and the solution was stirred for a further 3 hrs. Volatiles were removed *in vacuo* and the crude phosphoryl dichloride was converted to the desired phosphonic ester following General Procedure IA.

[14] D. J. Jones, E. M. O'Leary, T. P. O'Sullivan, *Tet. Lett.*, **2017**, *58*, 4212-4214.
[15] C.Courtens, M.Risseeuw,G.Caljon, P. Cos, S.Van Calenbergh, *ACS Med. Chem. Lett.* **2018**, 9, 986-989

General Procedure IC: Substrate synthesis from alkyl phosphonate ester:

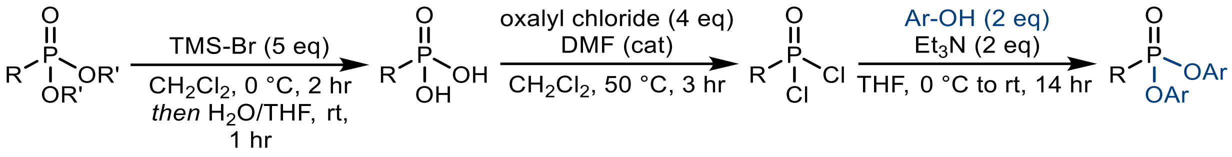

According to a modified literature procedure.[3] The corresponding alkyl phosphonate ester (4.00 mmol) was dissolved in CH_2Cl_2 (32.0 mL) and cooled to 0 °C then TMS-Br was added (5.28 mL, 20 mmol) and the solution was stirred for a further 2 hrs. Volatiles were removed *in vacuo* and the crude was stirred in THF (13.6 mL) and H_2O (0.136 mL) for 1 hr. Volatiles were removed *in vacuo* and azeotroped with toluene to remove all traces of water and dried under high vacuum for 12 hrs. The crude phosphonic acid was converted to the desired phosphonate ester following General Procedure IB.

Synthesis of Starting Materials

Bis(4-nitrophenyl) phenylphosphonate (**P-LGS1**)

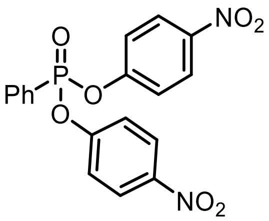

Bis(4-nitrophenyl) phenylphosphonate was synthesised following **GP IA**. Phenylphosphonic dichloride (779.9 mg, 4.00 mmol, 567 µL) was used and 4-nitrophenol (1.11g, 8.00 mmol) used as nucleophile. FCC Pentane:EtOAc 9:1 to 7:3. Colourless solid (1.15 g, 2.87 mmol, 72% yield).

Mp: 74-76 °C (from pentane); **^{1}H NMR** (400 MHz, $CDCl_3$) δ 8.24 – 8.18 (m, 4H), 8.05 – 7.91 (m, 2H), 7.74 – 7.65 (m, 1H), 7.62 – 7.52 (m, 2H), 7.42 – 7.33 (m, 4H); **^{13}C NMR** (101 MHz, $CDCl_3$) δ 154.9 (d, *J* = 7.0 Hz), 145.2, 134.5 (d, *J* = 3.2 Hz), 132.4 (d, *J* = 10.8 Hz), 129.3 (d, *J* = 16.2 Hz), 125.9, 124.0, 121.3 (d, *J* = 4.9 Hz); **^{31}P NMR** (162 MHz, $CDCl_3$) δ 12.72; **IR** (film) υ_{max}/cm^{-1}: 3082, 1614, 1590, 1520, 1488, 1345, 1200, 1161, 1131, 917, 857, 748, 690; **HRMS** (ESI+): calcd. for $C_{18}H_{13}O_7N_2NaP$ 423.0352 $[M+Na]^+$, found 423.0352.

Bis(2-nitrophenyl) phenylphosphonate (**P-LG1**)

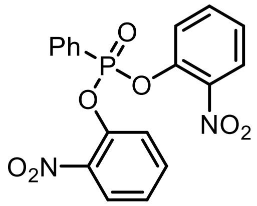

Bis(2-nitrophenyl) phenylphosphonate was synthesised following **GP IA**. Phenylphosphonic dichloride (779.9 mg, 4.00 mmol, 567 µL) was used and 2-nitrophenol (1.11 g, 8.00 mmol) used as nucleophile. FCC Pentane:EtOAc 9:1 to 7:3. Pale yellow solid (1.11 g, 2.78 mmol, 70% yield).

Mp: 57-58 °C (from pentane); **^{1}H NMR** (400 MHz, $CDCl_3$) δ 8.14 – 8.05 (m, 2H), 7.94 (dt, *J* = 8.4, 1.2 Hz, 2H), 7.71 – 7.65 (m, 1H), 7.61 – 7.48 (m, 6H), 7.33 – 7.27 (m, 2H); **^{13}C NMR** (101 MHz, $CDCl_3$) δ 143.1 (d, *J* = 7.6 Hz), 141.6, 134.5 (d, *J* = 1.4 Hz), 134.4 (d, *J* = 3.3 Hz), 132.7 (d, *J* = 11.3 Hz), 129.1 (d, *J* = 16.7 Hz), 126.1, 125.8 (d, *J* = 1.2 Hz), 124.8 (d, *J* = 196.5 Hz), 123.3 (d, *J* = 3.2 Hz); **^{31}P NMR** (162 MHz, $CDCl_3$) δ 13.90; **IR** (film) υ_{max}/cm^{-1}: 1602, 1526, 1480, 1349, 1264, 1213, 1130, 1089, 919, 846, 779, 746; **HRMS** (ESI+): calcd. for $C_{18}H_{14}O_7N_2P$ 401.0553 $[M+H]^+$, found 401.0525.

Bis(5-methyl-2-nitrophenyl) phenylphosphonate (**P-LG2**)

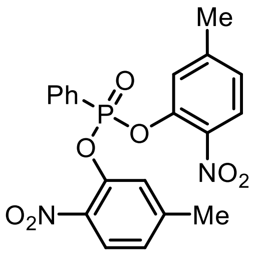

Bis(5-methyl-2-nitrophenyl) phenylphosphonate was synthesised following **GP IA**. Phenylphosphonic dichloride (779.9 mg, 4.00 mmol, 567 µL) was used and 5-methyl-2-nitrophenol (1.22 g, 8.00 mmol) used as nucleophile. FCC Pentane:EtOAc 9:1 to 7:3. Colourless solid (1.30 g, 3.06 mmol, 76% yield).

Mp: 76-77 °C; **^{1}H NMR** (400 MHz, $CDCl_3$) δ 8.15 – 8.04 (m, 2H), 7.87 (dd, *J* = 8.4, 0.9 Hz, 2H), 7.72 – 7.63 (m, 1H), 7.61 – 7.52 (m, 2H), 7.31 (td, *J* = 1.7, 0.8 Hz, 2H), 7.08 (ddt, *J* = 8.4, 1.7, 0.8 Hz, 2H), 2.37 (s, 6H); **^{13}C NMR** (101 MHz, $CDCl_3$) δ 146.5, 143.1 (d, *J* = 7.9 Hz), 139.2, 134.3 (d, *J* = 3.3 Hz), 132.8 (d, *J* = 11.3 Hz), 129.0 (d, *J* = 16.7 Hz), 126.4, 126.1, 125.0 (d, *J* = 196.6 Hz), 123.6 (d, *J* = 3.3 Hz), 21.7; **^{31}P NMR** (162 MHz, $CDCl_3$) δ 13.57; **IR** (film) υ_{max}/cm^{-1}: 1591, 1521, 1491, 1345, 1265, 1242, 1171, 1129, 1086, 853, 828, 752, 731, 711; **HRMS**: calcd. for $C_{20}H_{18}O_7N_2P$ 429.0846 $[M+H]^+$, found 429.0840.

Bis(4-methyl-2-nitrophenyl) phenylphosphonate (**P-LG3**)

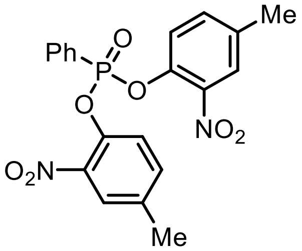

Bis(4-methyl-2-nitrophenyl) phenylphosphonate was synthesised following **GP IA**. Phenylphosphonic dichloride (779.9 mg, 4.00 mmol, 567 µL) was used and 4-methyl-2-nitrophenol (1.22 g, 8.00 mmol) used as nucleophile. FCC Pentane:EtOAc 9:1 to 7:3. Colourless solid (1.29 g, 3.02 mmol, 76% yield).

Mp: 126-128 °C; **^{1}H NMR** (400 MHz, $CDCl_3$) δ 8.12 – 7.98 (m, 2H), 7.72 (dd, *J* = 2.0, 1.0 Hz, 2H), 7.68 – 7.62 (m, 1H), 7.58 – 7.50 (m, 2H), 7.37 (dd, *J* = 8.4, 1.5 Hz, 2H), 7.34 – 7.28 (m, 2H), 2.36 (d, *J* = 0.9 Hz, 6H); **^{13}C NMR** (101 MHz, $CDCl_3$) δ 141.1 (d, *J* = 6.2 Hz), 140.8 (d, *J* = 7.8 Hz), 136.2 (d, *J* = 1.4 Hz), 135.1 (d, *J* = 1.6 Hz), 134.2 (d, *J* = 3.4 Hz), 132.7 (d, *J* = 11.2 Hz), 129.0 (d, *J* = 16.6 Hz), 126.1, 124.9 (d, *J* = 196.0 Hz), 123.0 (d, *J* = 3.2 Hz), 20.7; **^{31}P NMR** (162 MHz, $CDCl_3$) δ 13.98; **IR** (film) υ_{max}/cm^{-1}: 1531, 1496, 1351, 1232, 1205, 1130, 1086, 936, 901, 808; **HRMS** (ESI+): calcd. for $C_{20}H_{18}O_7N_2P$ 429.0846 $[M+H]^+$, found 429.0838.

Bis(2-methyl-6-nitrophenyl) phenylphosphonate (**P1**)

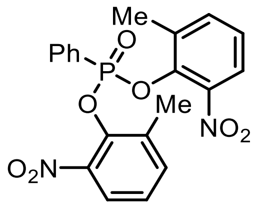

Bis(5-methyl-2-nitrophenyl) phenylphosphonate was synthesised following **GP IA**. Phenylphosphonic dichloride (780.0 mg, 4.00 mmol, 567 µL) was used and 6-methyl-2-nitrophenol (1.22 g, 8.00 mmol) used as nucleophile. FCC Pentane:EtOAc 9:1 to 7:3. Colourless solid (1.17 g, 2.73 mmol, 68% yield).

Mp: 168-169 °C; **^{1}H NMR** (400 MHz, $CDCl_3$) δ 8.05 – 7.95 (m, 2H), 7.68 (dd, *J* = 8.1, 1.7 Hz, 2H), 7.41 (ddt, *J* = 7.6, 1.6, 0.8 Hz, 2H), 7.19 (td, *J* = 7.9, 1.2 Hz, 2H), 7.07 – 6.98 (m, 2H), 2.18 (s, 6H); **^{13}C NMR** (101 MHz, $CDCl_3$) δ 143.6, 140.9 (d, *J* = 9.9 Hz), 136.0 (d, *J* = 1.8 Hz), 134.2 (d, *J* = 3.7 Hz), 134.2 (d, *J* = 3.3 Hz), 132.9 (d, *J* = 11.1 Hz), 129.0 (d, *J* = 16.4 Hz), 125.7 (d, *J* = 195.9 Hz), 125.6 (d, *J* = 1.8 Hz), 123.4 (d, *J* = 1.7 Hz), 17.2; **^{31}P NMR** (162 MHz, $CDCl_3$) δ 13.01; **IR** (film) υ_{max}/cm^{-1}: 1532, 1471, 1357, 1275, 1213,1178, 1152, 1130, 1092, 933, 803; 751, 694; **HRMS** (ESI+) calcd. for $C_{20}H_{18}O_7N_2P$ 429.0846 $[M+H]^+$, found 429.0842.

Bis(2-methyl-6-nitrophenyl) (4-methoxyphenyl)phosphonate (**P2**)

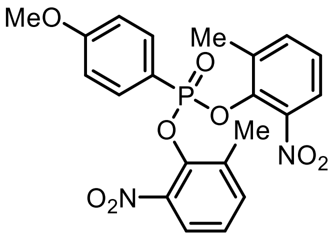

Bis(2-methyl-6-nitrophenyl) (4-methoxyphenyl)phosphonate was synthesised following **GP IA**. 4-methoxyphenyl phosphonic dichloride (900 mg, 4.00 mmol, 635 µL) was used and 5-methyl-2-nitrophenol (1.22 g, 8.00 mmol) used as nucleophile. FCC Pentane:EtOAc 9:1 to 6:4. Colourless solid (1.33 g, 2.90 mmol, 72% yield).

Mp: 127-128 °C; **^{1}H NMR** (400 MHz, $CDCl_3$) δ 8.05 – 7.95 (m, 2H), 7.68 (dd, *J* = 8.1, 1.7 Hz, 2H), 7.41 (ddt, *J* = 7.6, 1.6, 0.8 Hz, 2H), 7.19 (td, *J* = 7.9, 1.2 Hz, 2H), 7.07 – 6.98 (m, 2H), 3.89 (s, 3H), 2.18 (s, 6H); **^{13}C NMR** (101 MHz, $CDCl_3$) δ 164.3 (d, *J* = 3.6 Hz), 143.7 (d, *J* = 3.2 Hz), 141.0 (d, *J* = 9.9 Hz), 135.9 (d, *J* = 1.8 Hz), 135.1 (d, *J* = 13.0 Hz), 134.3 (d, *J* = 3.8 Hz), 125.4 (d, *J* = 1.9 Hz), 123.3 (d, *J* = 1.7 Hz), 116.5 (d, *J* = 204.5 Hz), 114.5 (d, *J* = 17.8 Hz), 55.6, 17.2; **^{31}P NMR** (162 MHz, $CDCl_3$) δ 14.12; **IR** (film) υ_{max}/cm^{-1}:1598, 1531, 1471, 1357, 1260, 1213, 1179, 1157, 1129, 1092, 906, 804, 756; **HRMS** (ESI+) calcd. for $C_{21}H_{20}N_2O_8P$ 459.0952 $[M+H]^+$, found 459.0945.

Bis(2-methyl-6-nitrophenyl) methylphosphonate (**P3**)

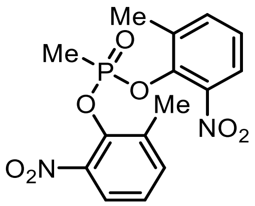

Bis(2-methyl-6-nitrophenyl) methylphosphonate was synthesised following **GP IA**. methylphosphonic dichloride (532 mg, 4.00 mmol, 362 µL) was used and 5-methyl-2-nitrophenol (1.22 g, 8.00 mmol) used as nucleophile. FCC Pentane:EtOAc 9:1 to 7:3. Colourless solid (520.0 mg, 1.42 mmol, 35% yield).

Mp: 136-138 °C; **^{1}H NMR** (400 MHz, $CDCl_3$) δ 7.77 (dd, *J* = 8.1, 1.7 Hz, 2H), 7.50 (ddt, *J* = 7.7, 1.7, 0.8 Hz, 2H), 7.24 (td, *J* = 7.9, 1.2 Hz, 2H), 2.40 (d, *J* = 0.9 Hz, 6H), 1.98 (d, *J* = 18.0 Hz, 3H); **^{13}C NMR** (101 MHz, $CDCl_3$) δ 143.0, 141.2 (d, *J* = 9.8 Hz), 136.3 (d, *J* = 1.8 Hz), 134.0 (d, *J* = 3.5 Hz), 125.5 (d, *J* = 1.8 Hz), 123.6 (d, *J* = 1.6 Hz), 17.2, 13.1 (d, *J* = 143.8 Hz); **^{31}P NMR** (162 MHz, $CDCl_3$) δ 26.75; **IR** (film) υ_{max}/cm^{-1}: 1598, 1531, 1471, 1357, 1314, 1269, 1214, 1180, 1158, 1093, 925, 803, 752; **HRMS** (ESI+) calcd. for $C_{15}H_{16}N_2O_7P$ 367.0690 $[M+H]^+$, found 367.0702.

Bis(2-methyl-6-nitrophenyl) ethylphosphonate (**P4**)

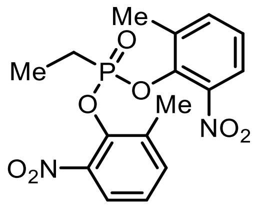

Bis(2-methyl-6-nitrophenyl) ethylphosphonate was synthesised following **GP IA**. ethylphosphonic dichloride (588 mg, 4.00 mmol, 427 µL) was used and 5-methyl-2-nitrophenol (1.22 g, 8.00 mmol) used as nucleophile. FCC Pentane:EtOAc 9:1 to 7:3. Colourless solid (1.08 g, 2.84 mmol, 71% yield).

Mp: 126-127 °C; **^{1}H NMR** (400 MHz, $CDCl_3$) δ 7.71 (dd, *J* = 8.2, 1.7 Hz, 2H), 7.45 (ddt, *J* = 7.6, 1.7, 0.8 Hz, 2H), 7.20 (td, *J* = 7.9, 1.2 Hz, 2H), 2.35 (d, *J* = 0.9 Hz, 6H), 2.21 (dq, *J* = 18.4, 7.6 Hz, 2H), 1.39 (dt, *J* = 22.4, 7.6 Hz, 3H); **^{13}C NMR** (101 MHz, $CDCl_3$) δ 143.3, 141.1 (d, *J* = 10.5 Hz), 136.1 (d, *J* = 1.6 Hz), 134.0 (d, *J* = 3.6 Hz), 125.4 (d, *J* = 1.8 Hz), 123.5 (d, *J* = 1.5 Hz), 21.0 (d, *J* = 140.1 Hz), 17.1, 6.7 (d, *J* = 7.6 Hz); **^{31}P NMR** (162 MHz, $CDCl_3$) δ 29.73; **IR** (film) υ_{max}/cm^{-1}: 2981, 1532, 1472, 1356, 1266, 1213, 1179, 924, 909, 804, 773, 747; **HRMS** (ESI+) calcd. for $C_{16}H_{18}N_2O_7P$ 381.0846 $[M+H]^+$, found 381.0838.

bis(2-methyl-6-nitrophenyl) isopropylphosphonate (**P5**)

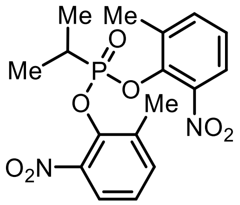

P5 was synthesized following **GP IA**. Isopropylphosphonic dichloride (643 mg, 4.00 mmol, 501 µL) was used and 5-methyl-2-nitrophenol (1.22 g, 8.00 mmol) was used as nucleophile. FCC Pentane:EtOAc 9:1 to 7:3. Colourless solid (741 mg, 1.88 mmol, 47% yield).

Mp 138 °C (pentane); **^{1}H NMR** (400 MHz, $CDCl_3$) δ 7.66 (dd, *J* = 8.1, 1.7 Hz, 2H), 7.44 (ddt, *J* = 7.7, 1.7, 0.8 Hz, 2H), 7.19 (td, *J* = 7.9, 1.1 Hz, 2H), 2.42 – 2.35 (m, 1H), 2.34 – 2.33 (m, 6H), 1.42 (dd, *J* = 20.8, 7.1 Hz, 6H); **^{13}C NMR** (101 MHz, $CDCl_3$) δ 143.6, 141.2 (d, *J* = 11.2 Hz), 135.9 (d, *J* = 1.6 Hz), 133.8 (d, *J* = 3.6 Hz), 125.3 (d, *J* = 1.7 Hz), 123.3 (d, *J* = 1.5 Hz), 28.2 (d, *J* = 138.1 Hz), 17.2, 16.4 (d, *J* = 5.5 Hz); **^{31}P NMR** (162 MHz, $CDCl_3$) δ 31.54; **IR** υ_{max}/cm^{-1}: 3318, 2970, 2095, 1640, 1532, 1355, 1262 , 1179, 1092, 934, 906, 756, 696; **HRMS** (ESI+) calcd. for $C_{17}H_{20}N_2O_7P$ 395.1003 $[M+H]^+$, found 395.1008.

Bis(2-methyl-6-nitrophenyl) hexylphosphonate (**P6**)

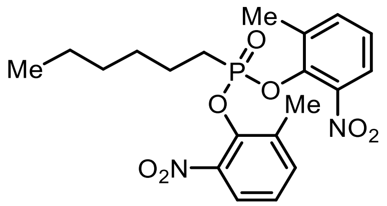

Bis(2-methyl-6-nitrophenyl) hexylphosphonate was synthesised following **GP IA**. Hexylphosphonic dichloride (812.0 mg, 4.00 mmol, 685 µL) was used and 5-methyl-2-nitrophenol (1.22 g, 8.00 mmol) used as nucleophile. FCC Pentane:EtOAc 9:1 to 7:3. Colourless solid (955 mg, 2.19 mmol, 55% yield).

Mp: 80-81 °C; **^{1}H NMR** (400 MHz, $CDCl_3$) δ 7.70 (dd, *J* = 8.1, 1.7 Hz, 2H), 7.45 (ddt, *J* = 7.7, 1.7, 0.8 Hz, 2H), 7.20 (td, *J* = 7.9, 1.1 Hz, 2H), 2.34 (s, 6H), 2.25 – 2.11 (m, 2H), 1.87 – 1.72 (m, 2H), 1.46 (p, *J* = 7.2 Hz, 2H), 1.36 – 1.27 (m, 4H), 0.92 – 0.85 (m, 3H); **^{13}C NMR** (101 MHz, $CDCl_3$) δ 143.3 (d, *J* = 3.4 Hz), 141.1 (d, *J* = 10.5 Hz), 136.0 (d, *J* = 1.9 Hz), 133.9 (d, *J* = 3.6 Hz), 125.4 (d, *J* = 1.8 Hz), 123.4 (d, *J* = 1.5 Hz), 31.3 (d, *J* = 1.4 Hz), 30.4 (d, *J* = 18.4 Hz), 27.7 (d, *J* = 137.0 Hz), 22.5, 22.4 (d, *J* = 6.1 Hz), 17.1, 14.1; **^{31}P NMR** (162 MHz, $CDCl_3$) δ 28.68; **IR** (film) υ_{max}/cm^{-1}: 2926, 1532, 1470, 1356, 1275, 1214, 1179, 1093, 1092, 906, 802, 752; **HRMS** (ESI+) calcd. for $C_{20}H_{26}N_2O_7P$ 437.1472 $[M+H]^+$, found 437.1461.

Bis(2-methyl-6-nitrophenyl) (cyclopropylmethyl)phosphonate (**P7**)

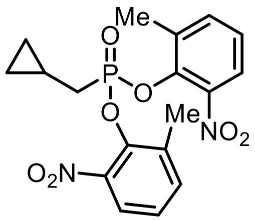

Bis(2-methyl-6-nitrophenyl) (cyclopropylmethyl)phosphonate was synthesised following **GP IC**. Diethyl (cyclopropylmethyl)phosphonate (754 µL, 4.00 mmol) was used and 5-methyl-2-nitrophenol (1.22 g, 8.00 mmol) used as nucleophile. FCC Pentane:EtOAc 9:1 to 2:1. Colourless solid (339 mg, 0.835 mmol, 21% yield).

Mp: 95-97 °C; **^{1}H NMR** (400 MHz, $CDCl_3$) 7.64 (dd, *J* = 8.2, 1.7 Hz, 2H), 7.39 (ddt, *J* = 7.6, 1.7, 0.7 Hz, 2H), 7.13 (td, *J* = 7.9, 1.1 Hz, 2H), 2.29 (d, *J* = 0.9 Hz, 6H), 2.08 (dd, *J* = 17.5, 7.0 Hz, 2H), 2.06 (d, *J* = 7.1 Hz, 1H), 0.68 – 0.53 (m, 2H), 0.39 – 0.22 (m, 2H); **^{13}C NMR** (101 MHz, $CDCl_3$) δ 143.2, 141.0 (d, *J* = 10.4 Hz), 135.9, 133.9 (d, *J* = 3.4 Hz), 125.2, 123.3, 32.6 (d, *J* = 136.9 Hz), 17.1, 5.7 (d, *J* = 10.4 Hz), 4.2 (d, *J* = 5.9 Hz); **^{31}P NMR** (162 MHz, $CDCl_3$) δ 26.54; **IR** (film) υ_{max}/cm^{-1}: 1533, 1471, 1357, 1281, 1213, 1179, 1158,1092, 924, 803; **HRMS** (ESI+) calcd. for $C_{18}H_{19}N_2NaO_7P$ 429.0828 $[M+Na]^+$, found 429.0820.

Bis(2-methyl-6-nitrophenyl) benzylphosphonate (**P8**)

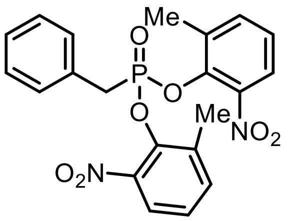

Bis(2-methyl-6-nitrophenyl) benzylphosphonate was synthesised following **GP IB**. Benzylphosphonic acid (441 mg, 2.56 mmol) was used and 5-methyl-2-nitrophenol (784 mg, 5.12 mmol) used as nucleophile. FCC Pentane:EtOAc 9:1 to 1:1. Colourless solid (144 mg, 0.320 mmol, 13% yield).

Mp: 125-126 °C; **^{1}H NMR** (400 MHz, $CDCl_3$) δ 7.73 (dd, *J* = 8.2, 1.7 Hz, 2H), 7.41 (dddd, *J* = 7.4, 4.7, 2.1, 1.1 Hz, 4H), 7.33 – 7.25 (m, 3H), 7.20 (td, *J* = 7.9, 1.1 Hz, 2H), 3.68 (s, 1H), 3.62 (s, 1H), 2.21 (s, 6H); **^{13}C NMR** (101 MHz, $CDCl_3$) δ 143.1, 141.2 (d, *J* = 10.7 Hz), 136.1 (d, *J* = 1.6 Hz), 134.0 (d, *J* = 3.5 Hz), 130.2 (d, *J* = 7.7 Hz), 129.5 (d, *J* = 9.9 Hz), 128.8 (d, *J* = 3.2 Hz), 127.6 (d, *J* = 3.9 Hz), 125.5 (d, *J* = 1.6 Hz), 123.4 (d, *J* = 1.5 Hz), 35.1 (d, *J* = 136.7 Hz), 17.0; **^{31}P NMR** (162 MHz, $CDCl_3$) δ 21.20; **IR** (film) υ_{max}/cm^{-1}: 1532, 1356, 1277, 1213, 1158, 926; **HRMS** (ESI+) calcd. for $C_{21}H_{19}N_2NaO_7P$ 465.0828 $[M+Na]^+$, found 465.0822.

Bis(2-methyl-6-nitrophenyl) (2-methylbenzyl)phosphonate (**P9**)

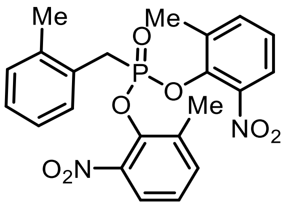

Bis(2-methyl-6-nitrophenyl) (2-methylbenzyl)phosphonate was synthesised following **GP IB**. 2-methylbenzyl phosphonic acid (745.0 mg, 4.00 mmol) was used and 5-methyl-2-nitrophenol (1.22 g, 8.00 mmol) used as nucleophile. FCC Pentane:EtOAc 9:1 to 7:3. Colourless solid (1.17 g, 2.56 mmol, 64% yield).

Mp: 126-127 °C; **^{1}H NMR** (400 MHz, $CDCl_3$) δ 7.72 (dd, *J* = 8.2, 1.7 Hz, 2H), 7.45 – 7.38 (m, 2H), 7.23 – 7.17 (m, 4H), 7.17 – 7.11 (m, 1H), 3.63 (d, *J* = 22.1 Hz, 2H), 2.45 (d, *J* = 1.9 Hz, 3H), 2.16 (s, 6H); **^{13}C NMR** (101 MHz, $CDCl_3$) δ 143.2, 141.1 (d, *J* = 10.9 Hz), 137.6 (d, *J* = 8.1 Hz), 136.2 (d, *J* = 1.7 Hz), 134.1 (d, *J* = 3.6 Hz), 131.1 (d, *J* = 6.1 Hz), 130.8 (d, *J* = 3.5 Hz), 128.2 (d, *J* = 10.3 Hz), 127.8 (d, *J* = 4.1 Hz), 126.3 (d, *J* = 3.8 Hz), 125.5 (d, *J* = 1.7 Hz), 123.4 (d, *J* = 1.5 Hz), 32.3 (d, *J* = 136.9 Hz), 20.1, 16.9; **^{31}P NMR** (162 MHz, $CDCl_3$) δ 21.72; **IR** (film) υ_{max}/cm^{-1}: 1531, 1470, 1356, 1277, 1213, 1179, 1158, 1092, 924, 803, 750; **HRMS** (ESI+) calcd. for $C_{22}H_{22}N_2O_7P$ 457.1159 $[M+H]^+$, found 457.1162.

Bis(2-methyl-6-nitrophenyl) (2-(trifluoromethyl)benzyl)phosphonate (**P10**)

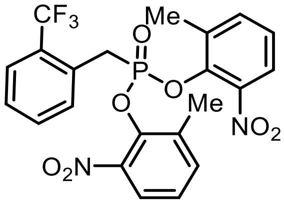

Bis(2-methyl-6-nitrophenyl) (2-(trifluoromethyl)benzyl)phosphonate was synthesised following **GP IC**. diethyl (2-(trifluoromethyl)benzyl)phosphonate (1.18 g, 4.00 mmol, 0.971 mL) was used and 5-methyl-2-nitrophenol (1.22 g, 8.00 mmol) used as nucleophile. FCC Pentane:EtOAc 9:1 to 7:3. Colourless solid (1.00 g, 1.97 mmol, 49% yield).

Mp: 114-115 °C; **^{1}H NMR** (400 MHz, $CDCl_3$) δ 7.74 (dd, *J* = 8.2, 1.7 Hz, 2H), 7.66 – 7.59 (m, 2H), 7.57 – 7.51 (m, 1H), 7.48 – 7.37 (m, 3H), 7.21 (td, *J* = 7.9, 1.1 Hz, 2H), 3.72 (d, *J* = 22.3 Hz, 2H), 2.20 (s, 6H); **^{13}C NMR** (101 MHz, $CDCl_3$) δ 143.0, 141.1 (d, *J* = 10.7 Hz), 136.3 (d, *J* = 1.8 Hz), 133.9 (d, *J* = 3.6 Hz), 133.7 (d, *J* = 7.2 Hz), 131.9 – 130.6 (m), 130.8 (d, *J* = 9.6 Hz), 129.3 (d, *J* = 3.1 Hz), 126.8 (dd, *J* = 7.9, 3.9 Hz), 125.7 (d, *J* = 1.7 Hz), 124.6 (t, *J* = 3.8 Hz), 124.0 (d, *J* = 272.5 Hz), 123.5 (d, *J* = 1.3 Hz), 34.9 (d, *J* = 137.7 Hz), 17.0; **^{19}F NMR** (377 MHz, CDCl3) δ -62.66; **^{31}P NMR** (162 MHz, $CDCl_3$) δ 19.83; **IR** (film) υ_{max}/cm^{-1}: 2981, 1532, 1356, 1330, 1278, 1226, 1213, 1159, 1125, 1092, 1075, 926, 887, 803, 766, 751, 702; **HRMS** (ESI+) calcd. for $C_{22}H_{19}F_3N_2O_7P$ 511.0876 $[M+H]^+$, found 511.0872.

Bis(2-methyl-6-nitrophenyl) thiophen-3-ylphosphonate (**P11**)

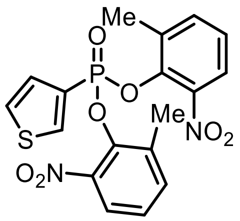

Bis(2-methyl-6-nitrophenyl) thiophen-3-ylphosphonate was synthesised following **GP IC**. Diethyl (thiophen-3-yl)phosphonate[16] (848 mg, 3.28 mmol) was used and 5-methyl-2-nitrophenol (918 mg, 6.00 mmol) used as nucleophile. FCC Pentane:EtOAc 9:1 to 1:1. Colourless solid (845 mg, 1.94 mmol, 44% yield).

Mp: 178-179 °C; **^{1}H NMR** (400 MHz, $CDCl_3$) δ 8.19 (ddd, *J* = 8.9, 2.9, 1.2 Hz, 1H), 7.67 – 7.53 (m, 3H), 7.46 (ddd, *J* = 5.1, 4.0, 2.9 Hz, 1H), 7.37 (ddt, *J* = 7.6, 1.7, 0.8 Hz, 2H), 7.14 (td, *J* = 7.9, 1.3 Hz, 2H), 2.14 (s, 6H). ; **^{13}C NMR** (101 MHz, $CDCl_3$) δ 143.41 (d, *J* = 2.8 Hz), 140.8 (d, *J* = 9.6 Hz), 138.9 (d, *J* = 20.7 Hz), 135.9, 134.1 (d, *J* = 3.4 Hz), 129.7 (d, *J* = 18.4 Hz), 127.9 (d, *J* = 21.8 Hz), 126.2 (d, *J* = 209 Hz), 125.5, 123.3 (d, *J* = 1.3 Hz), 16.9; **^{31}P NMR** (162 MHz, $CDCl_3$) δ 6.09; **IR** (film) υ_{max}/cm^{-1}: 1532, 1357, 1276, 1178, 1090, 943,924, 770; **HRMS** (ESI+) calcd. for $C_{18}H_{16}N_2O_7PS$ 435.0416 $[M+H]^+$, found 435.010.

[16] M. Lilley, B. Mambwe, R. F. W. Jackson, R. Muimo *Chem. Comm.,* **2014**, *50,* 9343-9345.

Bis(2-methyl-6-nitrophenyl) ((methylthio)methyl)phosphonate (**P12**)

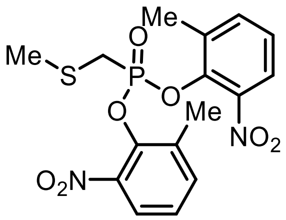

Bis(2-methyl-6-nitrophenyl) ((methylthio)methyl)phosphonate was synthesised following **GP IC**. diethyl ((methylthio)methyl)phosphonate (792 mg, 4.00 mmol, 0.701 mL) was used and 5-methyl-2-nitrophenol (1.22 g, 8.00 mmol) used as nucleophile. FCC Pentane:EtOAc 9:1 to 7:3. Colourless solid (627 mg, 1.52 mmol, 38% yield).

Mp: 122-123 °C; **^{1}H NMR** (400 MHz, $CDCl_3$) δ 7.75 (dd, *J* = 8.1, 1.7 Hz, 2H), 7.48 (ddt, *J* = 7.7, 1.7, 0.8 Hz, 2H), 7.21 (td, *J* = 7.9, 1.1 Hz, 2H), 3.21 (d, *J* = 12.0 Hz, 2H), 2.43 – 2.37 (m, 6H), 2.31 (d, *J* = 1.5 Hz, 3H); **^{13}C NMR** (101 MHz, $CDCl_3$) δ 142.7, 141.6 (d, *J* = 10.9 Hz), 136.3 (d, *J* = 1.6 Hz), 134.0 (d, *J* = 3.7 Hz), 125.5 (d, *J* = 1.6 Hz), 123.6 (d, *J* = 1.5 Hz), 29.1 (d, *J* = 150.6 Hz), 17.6 (d, *J* = 1.5 Hz), 17.2; **^{31}P NMR** (162 MHz, $CDCl_3$) δ 17.81; **IR** (film) υ_{max}/cm^{-1}: 1530, 1470, 1354, 1277, 1213, 1178, 1158, 1092, 926, 804, 749; **HRMS** (ESI+) calcd. for $C_{16}H_{18}N_2O_7PS$ 413.0567 $[M+H]^+$, found 413.0581.

Bis(2-methyl-6-nitrophenyl) ((benzyloxy)methyl)phosphonate (**P13**)

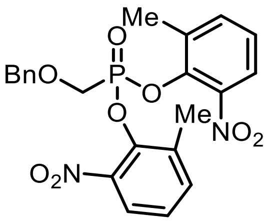

Bis(2-methyl-6-nitrophenyl) ((benzyloxy)methyl)phosphonate was synthesised following **GP IC**. Diethyl ((benzyloxy)methyl)phosphonate[17] (848 mg, 3.28 mmol) was used and 5-methyl-2-nitrophenol (918 mg, 6.00 mmol) used as nucleophile. FCC Pentane:EtOAc 9:1 to 1:1. Colourless solid (325 mg, 0.687 mmol, 21% yield).

Mp: 119-120 °C; **^{1}H NMR** (400 MHz, $CDCl_3$) δ 7.75 (dd, *J* = 8.1, 1.7 Hz, 2H), 7.46 (ddt, *J* = 7.7, 1.7, 0.8 Hz, 2H), 7.38 – 7.27 (m, 3H), 7.25 – 7.15 (m, 4H), 4.64 (s, 2H), 4.25 (d, *J* = 7.0 Hz, 2H), 2.41 (s, 6H); **^{13}C NMR** (101 MHz, $CDCl_3$) δ 142.6, 141.2 (d, *J* = 10.4 Hz), 136.3, 134.0 (d, *J* = 3.3 Hz), 128.4, 128.2, 128.1, 125.4, 123.4, 75.2 (d, *J* = 11.3 Hz), 64.9 (d, *J* = 167.7 Hz), 15.4; **^{31}P NMR** (162 MHz, $CDCl_3$) δ 21.72; **IR** (film) υ_{max}/cm^{-1}: 1532, 1461, 1354, 1321, 1244,1213, 1093, 926, 743; **HRMS** (ESI+) calcd. for $C_{22}H_{22}N_2O_8P$ 473.114 $[M+H]^+$, found 473.1108.

[17] Wei et al. *J. Med. Chem.* **2017**, *60*, 8580-8590.

bis(2-methyl-6-nitrophenyl) (((tert-butyldiphenylsilyl)oxy)methyl)phosphonate (**P14**)

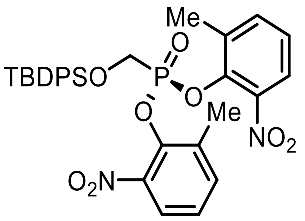

P14 was synthesized following **GP IC**. Diethyl (((tert-butyldiphenylsilyl)oxy)methyl)phosphonate (1.62, 4.00 mmol) was used and 5-methyl-2-nitrophenol (1.22 g, 8.00 mmol) used as nucleophile. FCC Pentane:EtOAc 100:0 to 4:1. Pale yellow solid (426 mg, 0.686 mmol, 17% yield).

Mp 146-150 °C; **^{1}H NMR** (400 MHz, $CDCl_3$) δ 7.76 (dd, *J* = 8.0, 1.7 Hz, 2H), 7.70 – 7.64 (m, 4H), 7.50 – 7.36 (m, 8H), 7.25 – 7.18 (m, 2H), 4.40 (d, *J* = 7.7 Hz, 2H), 2.49 – 2.41 (m, 6H), 0.97 (s, 9H).; **^{13}C NMR** (101 MHz, $CDCl_3$) δ 142.8 (d, *J* = 3.3 Hz), 141.4 (d, *J* = 10.5 Hz), 136.2, 135.8, 134.1 (d, *J* = 3.8 Hz), 131.8, 130.3, 128.1, 125.4, 123.6, 60.4 (d, *J* = 174.7 Hz), 26.7, 19.3, 17.2.; **^{31}P NMR** (162 MHz, $CDCl_3$) δ 16.14.; **IR** (film) $υ_{max}$/cm^{-1}: 2981, 2888, 1610, 1587, 1536, 1462, 1428, 1382, 1355, 1264, 1212, 1177, 1157, 1114, 1092, 940, 804, 771, 744, 703, 611. **HRMS** (ESI+): calcd. for $C_{31}H_{33}N_2O_8PSiNa$ 643.1636 $[M+Na]^+$, found 643.1633.

General Procedure II: Enantioselective Desymmetrisation of Phosphonate esters

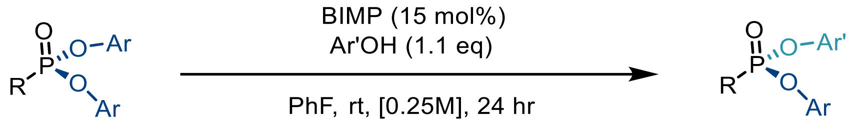

To the corresponding organoazide (0.015 mmol) and tris-*para*-tolylphosphine (0.015 mmol) under argon atmosphere was added THF (0.40 mL) and the reaction mixture was stirred for 24 h at room temperature. The formation of the organocatalysts was monitored by TLC. Upon completion volatiles were removed under a stream of N_2 yielding the expected iminiphosphorane which was used without further purification.

To the corresponding phosphonate (0.10 mmol) and BIMP catalyst (0.015 mmol) under argon atmosphere was added PhF (0.40 mL) and phenol (0.11 mmol, 1.1 equivalents) then the reaction was stirred at room temperature for 24 h. The reaction mixture was loaded directly onto silica gel and purified by flash column chromatography as specified in the individual experiment to afford pure desymmetrised phosphonate ester. The two enantiomers were separated by chiral HPLC using conditions specified in the individual experiment.

General Procedure III: Racemic Desymmetrisation of Phosphonate esters

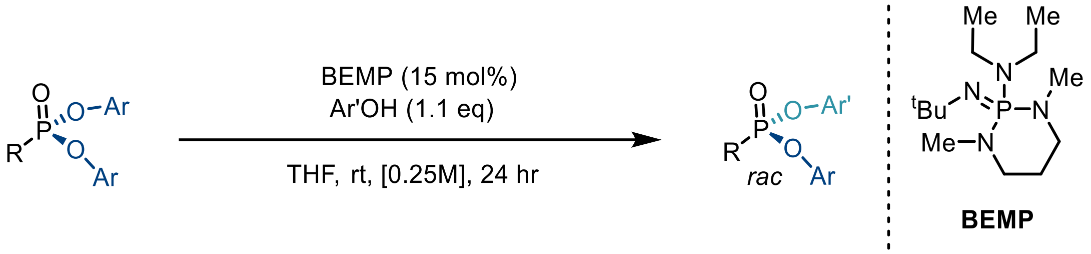

To the corresponding phosphonate (0.10 mmol) and 2-tert-Butylimino-2-diethylamino-1,3-dimethylperhydro-1,3,2-diazaphosphorine (BEMP) (4.5 µL, 0.015 mmol) under argon was added THF (0.40 mL) and the corresponding phenol (0.11 mmol) and the reaction was stirred at 22 °C for 24 h. Volatiles were removed under a stream of N_2 and the crude product was purified by flash column chromatography as specified in the individual experiment to afford the racemic desymmetrised phosphonate ester product. The two enantiomers were separated by chiral HPLC using conditions specified in the individual experiment.

Products: Leaving Group Variation

2,4-Dimethylphenyl (4-nitrophenyl) *(R)*-phenylphosphonate (**LGS1**)

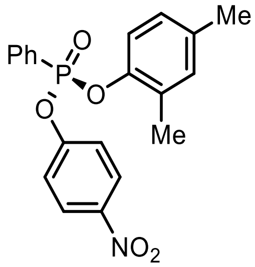

LGS1 was synthesised following **GP II**. **P-LGS1** (40.0 mg, 0.10 mmol) was used as phosphonate, 2,4-dimethylphenol (12.4 µL, 0.11 mmol) used as nucleophile and BIMP **B2** ($P(PMP)_3$ derived) (8.25 mg, 0.01 mmol) as catalyst in THF (0.2 mL). Pentane:EtOAc 7:3. Colourless oil (15.4mg, 0.041 mmol, 41% yield, 0% e.e.).

HPLC Conditions: CHIRALPAK IB, hexane/isopropanol = 90/10, 1 mL/min, λ = 220 nm, t (major) = 11.76 min, t (minor) = 12.78 min

^{1}H NMR (400 MHz, $CDCl_3$) δ 8.21 – 8.13 (m, 2H), 8.01 – 7.94 (m, 2H), 7.69 – 7.61 (m, 1H), 7.54 (ddd, *J* = 8.7, 6.9, 4.8 Hz, 2H), 7.35 – 7.30 (m, 2H), 7.13 (dd, *J* = 8.3, 1.5 Hz, 1H), 7.00 (t, *J* = 1.6 Hz, 1H), 6.92 (dd, *J* = 8.3, 2.3 Hz, 1H), 2.27 (s, 3H), 2.22 (s, 3H); **^{13}C NMR** (101 MHz, $CDCl_3$) δ 155.5 (d, *J* = 7.1 Hz), 146.6 (d, *J* = 8.6 Hz), 144.8, 135.2 (d, *J* = 1.6 Hz), 133.8 (d, *J* = 3.2 Hz), 132.3, 132.3 (d, *J* = 10.6 Hz), 129.1, 129.0, 127.7, 126.5 (d, *J* = 193.8 Hz), 125.7, 121.3 (d, *J* = 4.8 Hz), 120.2 (d, *J* = 2.8 Hz), 20.8, 16.6; **^{31}P NMR** (162 MHz, $CDCl_3$) δ 12.03; **IR** (film) υ_{max}/cm^{-1}: 2923, 1591, 1521 (NO_2), 1491, 1346 (NO_2), 1251, 1223 (P=O), 1193, 1130 ,1115, 953, 918, 749; **HRMS** (ESI+): calcd. for $C_{20}H_{19}O_5NP$ 384.09954 $[M+H]^+$, found 384.09943.

2,4-Dimethylphenyl (2-nitrophenyl) *(R)*-phenylphosphonate (**LG1**)

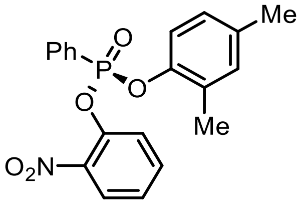

LG1 was synthesised following **GP II**. **P-LG1** (40.0 mg, 0.10 mmol) was used as phosphonate, 2,4-dimethylphenol (12.4 µL, 0.11 mmol) used as nucleophile and BIMP **B1** ($P(Ph)_3$ derived) (7.8 mg, 0.01 mmol) as catalyst in PhF (0.40 mL) at 0 °C. Pentane:EtOAc 7:3. Colourless oil (15.7 mg, 0.041 mmol, 41% yield, 86% e.e.).

HPLC Conditions: CHIRALPAK AD-H, hexane/isopropanol = 80/20, 1 mL/min, λ = 240 nm, t (major) = 19.87 min, t (minor) = 24.11 min

$[\alpha]_D^{25}$ = +20.9 (c = 0.56, $CHCl_3$); **^{1}H NMR** (400 MHz, $CDCl_3$) δ 8.05 (ddt, *J* = 14.3, 6.9, 1.4 Hz, 2H), 7.90 (dt, *J* = 8.2, 1.2 Hz, 1H), 7.65 (tt, *J* = 7.4, 1.5 Hz, 2H), 7.57 – 7.48 (m, 3H), 7.31 – 7.22 (m, 1H), 7.10 (dd, *J* = 8.2, 1.5 Hz, 1H), 6.97 (d, *J* = 2.2 Hz, 1H), 6.89 (dd, *J* = 8.3, 2.2 Hz, 1H), 2.25 (s, 3H), 2.16 (s, 3H) **^{13}C NMR** (101 MHz, $CDCl_3$) δ 146.7 (d, *J* = 8.5 Hz), 143.6 (d, *J* = 7.2 Hz), 135.1 (d, *J* = 1.6 Hz), 134.4, 133.8 (d, *J* = 3.3 Hz), 132.6 (d, *J* = 10.8 Hz), 132.6, 129.3, 128.9 (d, *J* = 16.1 Hz), 128.2 (d, *J* = 220.6 Hz), 127.6 (d, *J* = 1.6 Hz), 125.8, 125.3, 125.2, 123.5 (d, *J* = 3.1 Hz), 120.2 (d, *J* = 2.7 Hz), 20.8, 16.5; **^{31}P NMR** (162 MHz, $CDCl_3$) δ 12.88; **IR** (film) υ_{max}/cm^{-1}: 2924, 1663, 1531 (NO_2), 1495, 1352 (NO_2), 1276, 1231, 1194 (P=O), 1131, 1117, 951, 921, 750, 694; **HRMS** (ESI+): calcd. for $C_{20}H_{19}O_5NP$ 384.09954 $[M+H]^+$, found 384.09949.

2,4-Dimethylphenyl (5-methyl-2-nitrophenyl) *(R)*-phenylphosphonate (**LG2**)

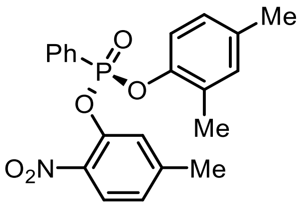

LG2 was synthesised following **GP II**. **P-LG2** (42.6 mg, 0.10 mmol) was used as phosphonate, 2,4-dimethylphenol (12.4 µL, 0.11 mmol) used as nucleophile and BIMP **B1** ($P(Ph)_3$ derived) (7.8 mg, 0.01 mmol) as catalyst in PhF (0.40 mL) at 0 °C. Pentane:EtOAc 7:3. Colourless oil (14.7 mg, 0.034 mmol, 34% yield, 67% e.e.).

SFC Conditions: CHIRALPAK ID, 1500 psi, 30 °C, flow: 1.5 mL/min, from 1% to 30% MeOH in 5 mins, λ = 220 nm, t (minor) = 3.99 min, t (major) = 4.14 min

$[\alpha]_D^{25}$ = +28.8 (c = 0.16, $CHCl_3$); **^{1}H NMR** (400 MHz, $CDCl_3$) δ 8.11 – 8.01 (m, 2H), 7.83 (dd, *J* = 8.4, 0.9 Hz, 1H), 7.67 – 7.58 (m, 1H), 7.52 (tdd, *J* = 8.3, 4.9, 0.8 Hz, 2H), 7.42 (td, *J* = 1.7, 0.9 Hz, 1H), 7.09 (dd, *J* = 8.3, 1.5 Hz, 1H), 7.04 (ddt, *J* = 8.3, 1.7, 0.8 Hz, 1H), 6.97 – 6.93 (m, 1H), 6.88 (dd, *J* = 8.2, 2.3 Hz, 1H), 2.37 (s, 3H), 2.24 (d, *J* = 0.9 Hz, 3H), 2.15 (s, 3H). **^{13}C NMR** (101 MHz, $CDCl_3$) δ 146.7 (d, *J* = 8.4 Hz), 146.4, 143.5 (d, *J* = 7.6 Hz), 139.2, 135.0 (d, *J* = 1.6 Hz), 133.7 (d, *J* = 3.2 Hz), 132.7, 132.5, 132.2, 129.3 (d, *J* = 5.4 Hz), 128.9, 128.8, 127.5 (d, *J* = 1.7 Hz), 126.2 (d, *J* = 195.2 Hz), 123.9 (d, *J* = 3.0 Hz), 120.2 (d, *J* = 2.8 Hz), 21.7, 20.8, 16.5.**^{31}P NMR** (162 MHz, $CDCl_3$) δ 12.80; **IR** (film) υ_{max}/cm^{-1}: 1596, 1524, 1497, 1348, 1263, 1198, 1131, 1118, 592; **HRMS** (ESI+): calcd. for $C_{21}H_{21}NO_5P$ 398.1152 $[M+H]^+$, found 398.1148.

2,4-Dimethylphenyl (4-methyl-2-nitrophenyl *(R)*-phenylphosphonate (**LG3**)

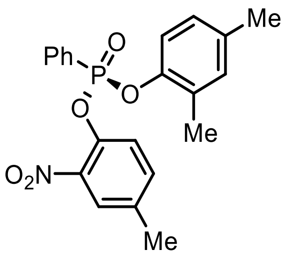

LG3 was synthesised following **GP II**. **P-LG3** (42.6 mg, 0.10 mmol) was used as phosphonate, 2,4-dimethylphenol (12.4 µL, 0.11 mmol) used as nucleophile and BIMP **B1** ($P(Ph)_3$ derived) (7.8 mg, 0.01 mmol) as catalyst in PhF (0.40 mL) at 0 °C. Pentane:EtOAc 7:3. Colourless oil (24.2 mg, 0.061 mmol, 61% yield, 89% e.e.).

HPLC Conditions: CHIRALPAK AS-H, hexane/isopropanol = gradient 98/2 to 70/30 over 40 min, 1 mL/min, λ = 220 nm, t (major) = 17.79 min, t (minor) = 16.50 min

$[\alpha]_D^{25}$ = +26.0 (c = 0.66, $CHCl_3$); **^{1}H NMR** (400 MHz, $CDCl_3$) δ 8.08 – 7.98 (m, 2H), 7.69 (dt, *J* = 2.1, 0.9 Hz, 1H), 7.66 – 7.59 (m, 1H), 7.55 – 7.47 (m, 3H), 7.31 (ddd, *J* = 8.5, 2.3, 0.8 Hz, 1H), 7.09 (dd, *J* = 8.2, 1.5 Hz, 1H), 6.98 – 6.93 (m, 1H), 6.88 (dd, *J* = 8.3, 2.3 Hz, 1H), 2.36 (d, *J* = 0.9 Hz, 3H), 2.24 (d, *J* = 1.0 Hz, 3H), 2.15 (s, 3H); **^{13}C NMR** (101 MHz, $CDCl_3$) δ 146.7 (d, *J* = 8.4 Hz), 141.3 (d, *J* = 7.2 Hz), 135.7, 135.0, 133.7 (d, *J* = 3.2 Hz), 132.6, 132.5, 132.2, 129.3 (d, *J* = 5.6 Hz), 128.9, 128.8, 127.6 (d, *J* = 1.6 Hz), 126.2 (d, *J* = 194.5 Hz), 125.9, 123.2 (d, *J* = 3.1 Hz), 120.2 (d, *J* = 2.8 Hz), 20.8, 20.7, 16.5.**^{31}P NMR** (162 MHz, $CDCl_3$) δ 12.91; **IR** (film) υ_{max}/cm^{-1}: 2924, 1534, 1496, 1353, 1276, 1240, 1194, 1131, 1118, 937, 899 **HRMS** (ESI+): calcd. for $C_{21}H_{21}NO_5P$ 398.1152 $[M+H]^+$, found 398.1145.

2,4-Dimethylphenyl (2-methyl-6-nitrophenyl) *(R)*-phenylphosphonate (**1**)

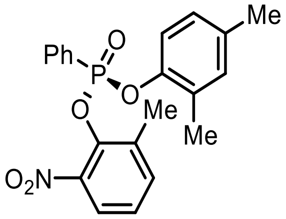

1 was synthesised following **GP II**. **P1** (42.6 mg, 0.10 mmol) was used as phosphonate, 2,4-dimethylphenol (12.4 µL, 0.11 mmol) used as nucleophile and BIMP **B1** - P(*p*-tol)$_3$ (11.7 mg, 0.015 mmol) as catalyst in PhF (0.40 mL) at 23 °C. Pentane:EtOAc 7:3. Colourless oil (33.0 mg, 0.084 mmol, 84% yield, 91% e.e.).

3 mmol scale: **P1** (1.28 g, 3 mmol) with BIMP **B1** - P(*p*-tol)$_3$ (351 mg, 0.45 mmol) in PhF (12.0 mL). Colourless solid (1.05 g, 2.64 mmol, 88% yield, 92% e.e.).

HPLC Conditions: CHIRALPAK AD-H, hexane/isopropanol = 80/20, 1 mL/min, λ = 220 nm, t (major) = 17.17 min, t (minor) = 28.28 min

Mp: 62 °C; **$[\alpha]_D^{25}$** = –15.9 (c = 1.00, $CHCl_3$); **^{1}H NMR** (400 MHz, $CDCl_3$) δ 8.07 – 7.98 (m, 2H), 7.71 (dd, *J* = 8.1, 1.7 Hz, 1H), 7.68 – 7.61 (m, 1H), 7.56 – 7.50 (m, 2H), 7.47 (ddt, *J* = 7.7, 1.6, 0.8 Hz, 1H), 7.22 (td, *J* = 7.9, 1.3 Hz, 1H), 6.93 – 6.85 (m, 2H), 6.79 (dd, *J* = 8.4, 2.2 Hz, 1H), 2.44 (d, *J* = 0.9 Hz, 3H), 2.22 – 2.18 (m, 3H), 2.05 (s, 3H); **^{13}C NMR** (101 MHz, $CDCl_3$) δ 146.8 (d, *J* = 7.2 Hz), 143.8, 141.0 (d, *J* = 10.1 Hz), 136.0 (d, *J* = 1.9 Hz), 134.8 (d, *J* = 1.5 Hz), 134.6 (d, *J* = 3.3 Hz), 133.6 (d, *J* = 3.3 Hz), 132.6 (d, *J* = 10.8 Hz), 132.2, 129.1 (d, *J* = 5.6 Hz), 128.8 (d, *J* = 16.2 Hz), 127.4 (d, *J* = 1.6 Hz), 126.6 (d, *J* = 194.3 Hz), 125.4 (d, *J* = 1.9 Hz), 123.3 (d, *J* = 1.8 Hz), 119.9 (d, *J* = 2.7 Hz), 20.7, 17.5, 16.4.**^{31}P NMR** (162 MHz, $CDCl_3$) δ 13.30; **IR** (film) υ_{max}/cm^{-1}: 1533, 1497, 1356, 1272, 1220, 1197, 1181, 1130, 1117, 910, 803, 768, 749, 694; **HRMS** (ESI+): calcd. for $C_{21}H_{21}NO_5P$ 398.1152 $[M+H]^+$, found 398.1140.

Products: Phenol Scope

2-Benzylphenyl (2-methyl-6-nitrophenyl) *(R)*-phenylphosphonate (**2**)

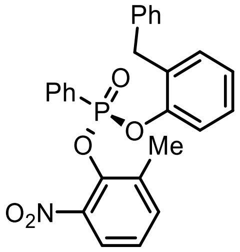

2 was synthesised following **GP II**. **P1** (42.6 mg, 0.10 mmol) was used as phosphonate, 2-benzylphenol (20.1 mg, 0.11 mmol) used as nucleophile and BIMP **B1**- P(*p*-tol)$_3$ (11.7 mg, 0.015 mmol) as catalyst in PhF (0.40 mL) at 23 °C. Pentane:EtOAc 7:3. Colourless oil (37.5 mg, 0.080 mmol, 80% yield, 88% e.e.).

HPLC Conditions: CHIRALPAK AD-H, hexane/isopropanol = 85/15, 1 mL/min, λ = 220 nm, t (major) = 22.55 min, t (minor) = 19.94 min.

[α]$_D$^{25} = –10.9 (c = 0.67, $CHCl_3$); **^{1}H NMR** (400 MHz, $CDCl_3$) δ 7.94 – 7.84 (m, 2H), 7.69 (dd, *J* = 8.2, 1.7 Hz, 1H), 7.65 – 7.58 (m, 1H), 7.50 – 7.40 (m, 3H), 7.25 – 7.16 (m, 4H), 7.15 – 7.10 (m, 1H), 7.08 – 6.98 (m, 5H), 3.93 – 3.85 (m, 2H), 2.34 (d, *J* = 1.0 Hz, 3H);**^{13}C NMR** (101 MHz, $CDCl_3$) δ 148.7 (d, *J* = 7.1 Hz), 143.6, 141.0 (d, *J* = 9.9 Hz), 139.8, 136.0 (d, *J* = 2.0 Hz), 134.5 (d, *J* = 3.4 Hz), 133.6 (d, *J* = 3.2 Hz), 132.4 (d, *J* = 11.0 Hz), 132.1 (d, *J* = 6.1 Hz), 131.4, 129.1, 128.8 (d, *J* = 16.3 Hz), 128.5, 127.6, 126.3 (d, *J* = 194.7 Hz), 126.2, 125.4 (d, *J* = 1.9 Hz), 125.4, 123.4 (d, *J* = 1.7 Hz), 120.2 (d, *J* = 2.8 Hz), 35.7, 17.4; **^{31}P NMR** (162 MHz, $CDCl_3$) δ 13.24; **IR** (film) υ_{max}/cm^{-1}: 1534, 1490, 1452, 1357, 1273, 1215, 1171, 1130, 1093, 926, 751; **HRMS** (ESI+): calcd. for $C_{26}H_{23}NO_5P$ 460.1308 $[M+H]^+$, found 460.1314.

2-Allylphenyl (2-methyl-6-nitrophenyl) *(R)*-phenylphosphonate (**3**)

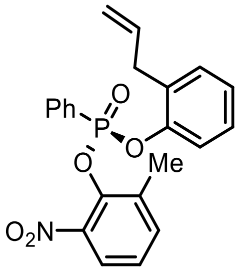

3 was synthesised following **GP II**. **P1** (42.6 mg, 0.10 mmol) was used as phosphonate, 2-allylphenol (14.6 mg, 14.4 µL, 0.11 mmol) used as nucleophile and BIMP **B1**- P(*p*-tol)$_3$ (11.7 mg, 0.015 mmol) as catalyst in PhF (0.40 mL) at 23 °C. Pentane:EtOAc 7:3. Colourless oil (29.8 mg, 0.071 mmol, 71% yield, 86% e.e.).

SFC Conditions: CHIRALPAK IC, 1500 psi, 30 °C, flow : 1.5 mL/min, from 1% to 30% MeOH in 5 mins, λ = 220 nm, t (major) = 4.03 min, t (minor) = 4.16 min

$[\alpha]_D^{25}$ = –8.4 (c = 0.63, $CHCl_3$); **^{1}H NMR** (400 MHz, $CDCl_3$) δ 8.07 – 7.98 (m, 2H), 7.72 (dd, *J* = 8.1, 1.7 Hz, 1H), 7.69 – 7.63 (m, 1H), 7.58 – 7.50 (m, 2H), 7.47 (ddt, *J* = 7.7, 1.6, 0.8 Hz, 1H), 7.23 (td, *J* = 7.9, 1.3 Hz, 1H), 7.17 – 7.10 (m, 1H), 7.08 – 7.02 (m, 3H), 5.79 (ddt, *J* = 16.7, 10.1, 6.5 Hz, 1H), 4.99 (dq, *J* = 10.1, 1.5 Hz, 1H,), 4.93 (dq, *J* = 17.0, 1.7 Hz, 1H), 3.27 (dt, *J* = 6.5, 1.6 Hz, 2H), 2.42 (d, *J* = 0.9 Hz, 3H);**^{13}C NMR** (101 MHz, $CDCl_3$) δ 148.5 (d, *J* = 7.4 Hz), 143.7, 141.1 (d, *J* = 10.1 Hz), 136.1 (d, *J* = 1.9 Hz), 135.9, 134.6 (d, *J* = 3.5 Hz), 133.7 (d, *J* = 3.3 Hz), 132.5 (d, *J* = 10.9 Hz), 131.3 (d, *J* = 6.0 Hz), 130.7, 128.9 (d, *J* = 16.3 Hz), 127.5, 126.5 (d, *J* = 182.9 Hz), 125.5 (d, *J* = 1.8 Hz), 125.4, 123.4 (d, *J* = 1.7 Hz), 120.4 (d, *J* = 2.7 Hz), 116.4, 34.0, 17.5; **^{31}P NMR** (162 MHz, $CDCl_3$) δ 13.16; **IR** (film) υ_{max}/cm^{-1}: 1535, 1489, 1357, 1272, 1212, 1170, 1130, 925, 750; **HRMS** (ESI+): calcd. for $C_{22}H_{21}NO_5P$ 410.1152 [M+H]$^+$, found 410.1144.

2-Ethylphenyl (2-methyl-6-nitrophenyl) *(R)*-phenylphosphonate (**4**)

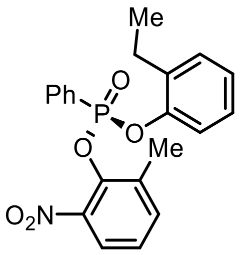

4 was synthesised following **GP II**. **P1** (42.6 mg, 0.10 mmol) was used as phosphonate, 2-ethylphenol (13.4 mg, 13.0 µL, 0.11 mmol) used as nucleophile and BIMP **B1** - P(*p*-tol)$_3$ (11.7 mg, 0.015 mmol) as catalyst in PhF (0.40 mL) at 23 °C. Pentane:EtOAc 7:3. Colourless oil (29.9 mg, 0.070 mmol, 70% yield, 91% e.e.).

HPLC Conditions: CHIRALPAK AD-H, hexane/isopropanol = 85/15, 1 mL/min, λ = 220 nm, t (major) = 13.02 min, t (minor) = 13.88 min.

$[\alpha]_D^{25}$ = –5.9 (c = 0.72, $CHCl_3$); **^{1}H NMR** (400 MHz, $CDCl_3$) δ 8.10 – 7.98 (m, 2H), 7.73 (dt, *J* = 8.3, 1.1 Hz, 1H), 7.66 (tdd, *J* = 6.9, 2.9, 1.4 Hz, 1H), 7.59 – 7.52 (m, 2H), 7.48 (ddt, *J* = 7.6, 1.6, 0.8 Hz, 1H), 7.23 (td, *J* = 7.9, 1.3 Hz, 1H), 7.18 – 7.13 (m, 1H), 7.08 – 6.98 (m, 3H), 2.50 (qd, *J* = 7.5, 2.0 Hz, 2H), 2.43 (d, *J* = 0.9 Hz, 3H), 1.06 (t, *J* = 7.6 Hz, 3H);**^{13}C NMR** (101 MHz, $CDCl_3$) δ 148.5 (d, *J* = 7.3 Hz), 143.8, 141.1 (d, *J* = 10.0 Hz), 136.1 (d, *J* = 1.9 Hz), 135.2 (d, *J* = 5.8 Hz), 134.6 (d, *J* = 3.5 Hz), 133.7 (d, *J* = 3.2 Hz), 132.6, 132.5, 129.8, 128.9 (d, *J* = 16.3 Hz), 127.0 (d, *J* = 1.4 Hz), 126.5 (d, *J* = 195.0 Hz), 125.4 (d, *J* = 1.8 Hz), 123.4 (d, *J* = 1.7 Hz), 120.3 (d, *J* = 2.7 Hz), 23.1, 17.5, 14.2; **^{31}P NMR** (162 MHz, $CDCl_3$) δ 13.08; **IR** (film) υ_{max}/cm^{-1}: 1534, 1490, 1357, 1273, 1213, 1179, 1159, 1130, 1117, 925, 751; **HRMS** (ESI+): calcd. for $C_{21}H_{21}NO_5P$ 398.1152 $[M+H]^+$, found 398.1140.

2-Methyl-6-nitrophenyl *o*-tolyl *(R)*-phenylphosphonate (**5**)

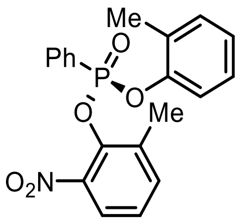

5 was synthesised following **GP II**. **P1** (42.6 mg, 0.10 mmol) was used as phosphonate, 2, methylphenol (11.9 mg, 0.11 mmol) used as nucleophile and BIMP **B1**- P(*p*-tol)$_3$ (11.7 mg, 0.015 mmol) as catalyst in PhF (0.40 mL) at 23 °C. Pentane:EtOAc 7:3. Colourless oil (31.4 mg, 0.082 mmol, 82% yield, 82% e.e.).

HPLC Conditions: CHIRALPAK AD-H, hexane/isopropanol = 80/20, 1 mL/min, λ = 220 nm, t (major) = 11.03 min, t (minor) = 14.71 min.

$[\alpha]_D^{25}$ = –3.3 (c = 0.58, $CHCl_3$); **^{1}H NMR** (400 MHz, $CDCl_3$) 8.08 – 8.00 (m, 2H), 7.72 (dd, *J* = 8.2, 1.7 Hz, 1H), 7.69 – 7.60 (m, 1H), 7.58 – 7.51 (m, 2H), 7.48 (ddt, *J* = 7.6, 1.7, 0.8 Hz, 1H), 7.23 (td, *J* = 7.9, 1.3 Hz, 1H), 7.13 – 7.08 (m, 1H), 7.04 – 6.96 (m, 3H), 2.44 (s, 3H), 2.11 (s, 3H);**^{13}C NMR** (101 MHz, $CDCl_3$) δ 149.0 (d, *J* = 7.2 Hz), 143.9, 141.0 (d, *J* = 10.3 Hz), 136.0 (d, *J* = 2.0 Hz), 134.6 (d, *J* = 3.5 Hz), 133.7 (d, *J* = 3.2 Hz), 132.6 (d, *J* = 10.9 Hz), 131.6, 129.6 (d, *J* = 5.6 Hz), 128.9, 127.0 (d, *J* = 1.5 Hz), 126.5 (d, *J* = 194.7 Hz), 125.5 (d, *J* = 1.9 Hz), 125.3, 123.4 (d, *J* = 1.8 Hz), 120.2 (d, *J* = 2.7 Hz), 17.5, 16.5; **^{31}P NMR** (162 MHz, $CDCl_3$) δ 13.18; **IR** (film) υ_{max}/cm^{-1}:1534, 1492, 1357, 1272, 1218, 1172, 1130, 1111, 926, 865, 750; **HRMS** (ESI+): calcd. for $C_{20}H_{19}NO_5P$ 384.0995 $[M+H]^+$, found 384.0983.

2-(((*tert*-Butyldimethylsilyl)oxy)methyl)phenyl (2-methyl-6-nitrophenyl) (*R*)-(4-methoxyphenyl)phosphonate (**6**)

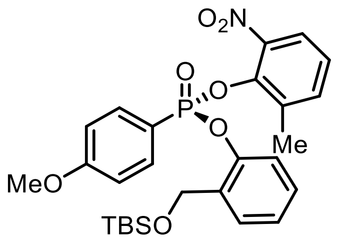

6 was synthesised following **GP II**. **P2** (45.8 mg, 0.10 mmol) was used as phosphonate, 2-(((tert-butyldimethylsilyl)oxy)methyl)phenol[18] (28.0 mg, 0.11 mmol) used as nucleophile and BIMP **B1** - P(*p*-tol)$_3$ (11.7 mg, 0.015 mmol) as catalyst in PhF (0.40 mL) at 23 °C. Pentane:EtOAc 7:3. Colourless Oil (53.4 mg, 0.098 mmol, 98% yield, 91% e.e.).

HPLC Conditions: CHIRALPAK IA, hexane/isopropanol = 90/10, 1 mL/min, λ = 220 nm, t (major) = 8.63 min, t (minor) = 9.40 min.

$[\alpha]_D^{25}$ = –7.2 (c = 1.00, $CHCl_3$); **^{1}H NMR** (400 MHz, $CDCl_3$) δ 8.02 – 7.89 (m, 2H), 7.72 (dd, *J* = 8.1, 1.7 Hz, 1H), 7.51 – 7.45 (m, 2H), 7.23 (td, *J* = 7.9, 1.3 Hz, 1H), 7.11 (tt, *J* = 7.4, 1.1 Hz, 1H), 7.07 – 6.99 (m, 3H), 6.97 (dt, *J* = 8.0, 1.3 Hz, 1H), 4.69 (d, *J* = 14.6 Hz, 1H), 4.51 (d, *J* = 14.6 Hz, 1H), 3.87 (s, 3H), 2.46 (d, *J* = 1.0 Hz, 3H), 0.92 (s, 9H), 0.05 (s, 6H); **^{13}C NMR** (101 MHz, $CDCl_3$) δ 164.0 (d, *J* = 3.6 Hz), 146.8 (d, *J* = 6.9 Hz), 143.9, 141.0 (d, *J* = 10.2 Hz), 136.0 (d, *J* = 1.9 Hz), 134.8, 134.6, 133.1 (d, *J* = 5.6 Hz), 127.6, 127.3, 125.5 (d, *J* = 1.9 Hz), 125.3, 123.3 (d, *J* = 1.8 Hz), 119.7 (d, *J* = 2.8 Hz), 117.1 (d, *J* = 202.5 Hz), 114.5 (d, *J* = 17.4 Hz), 59.8, 55.6, 26.1, 18.5, 17.6, -5.3, -5.3; **^{31}P NMR** (162 MHz $CDCl_3$) δ 14.26; **IR** (film) υ_{max}/cm^{-1}: 2929, 2856, 1599, 1535, 1456, 1357, 1257, 1213, 1173, 1129, 1077, 921, 835, 805, 764, 736; **HRMS** (ESI+): calcd. for $C_{27}H_{34}NO_7PSi$ 544.1915 $[M+H]^+$, found 544.1913.

[18] T. Yoshimura; K. Tomohara; T. Kawabata *J. Am. Chem. Soc.* **2013**, 135, 7102–7105.

2,3-Dimethylphenyl (2-methyl-6-nitrophenyl) *(R)*-phenylphosphonate (**7**)

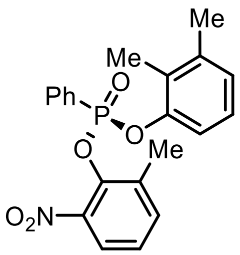

7 was synthesised following **GP II**. **P1** (42.6 mg, 0.10 mmol) was used as phosphonate, 2,3-dimethylphenol (13.4 mg, 0.11 mmol) used as nucleophile and BIMP **B1** - P(*p*-tol)$_3$ (11.7 mg, 0.015 mmol) as catalyst in PhF (0.40 mL) at 23 °C. Pentane:EtOAc 7:3. Colourless oil (36.8 mg, 0.093 mmol, 93% yield, 88% e.e.).

HPLC Conditions: CHIRALPAK IA, hexane/isopropanol = 85/15, 1 mL/min, λ = 220 nm, t (major) = 11.98 min, t (minor) = 13.86 min.

[α]$_D^{25}$ = –8.4 (c = 0.77, $CHCl_3$); **^{1}H NMR** (400 MHz, $CDCl_3$) δ 8.07 – 7.96 (m, 2H), 7.72 (dd, *J* = 8.2, 1.7 Hz, 1H), 7.68 – 7.63 (m, 1H), 7.58 – 7.50 (m, 2H), 7.48 (ddt, *J* = 7.6, 1.6, 0.7 Hz, 1H), 7.23 (td, *J* = 7.9, 1.3 Hz, 1H), 6.94 – 6.79 (m, 3H), 2.44 (d, *J* = 0.9 Hz, 3H), 2.21 (s, 3H), 2.01 (s, 3H); **^{13}C NMR** (101 MHz, $CDCl_3$) δ 148.8 (d, *J* = 7.2 Hz), 143.8, 141.1 (d, *J* = 10.2 Hz), 138.9, 136.0 (d, *J* = 2.0 Hz), 134.6 (d, *J* = 3.3 Hz), 133.6 (d, *J* = 3.2 Hz), 128.9, 128.7, 128.1 (d, *J* = 5.8 Hz), 126.8, 126.5 (d, *J* = 194.5 Hz), 126.1 (d, *J* = 1.5 Hz), 125.4 (d, *J* = 1.9 Hz), 123.3 (d, *J* = 1.8 Hz), 117.8 (d, *J* = 2.7 Hz), 20.3, 17.5, 12.5; **^{31}P NMR** (162 MHz, $CDCl_3$) δ 13.14; **IR** (film) $υ_{max}$/cm^{-1}: 1534, 1469, 1357, 1274, 1232, 1218, 1182, 1131, 1064, 926, 804, 750; **HRMS** (ESI+): calcd. for $C_{21}H_{21}NO_5P$ 398.1152 $[M+H]^+$, found 398.1143.

4-Chloro-2-cyclohexylphenyl (2-methyl-6-nitrophenyl) (*R*)-phenylphosphonate (**8**)

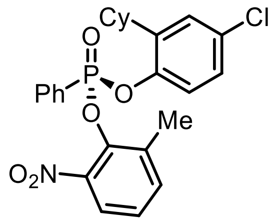

8 was synthesised following **GP II**. **P1** (43.4 mg, 0.100 mmol) was used as phosphonate, 4-chloro-2-cyclohexylphenol (23.0 mg, 0.11 mmol) used as nucleophile and BIMP **B1**- P(*p*-tol)$_3$ (11.7 mg, 0.015 mmol) as catalyst in PhF (0.40 mL) at 23 °C. Pentane:EtOAc 7:3. Colourless oil (29.8 mg, 0.061 mmol, 61% yield, 75% e.e.).

HPLC Conditions: CHIRALPAK AD-H, hexane/isopropanol = 90/10, 1 mL/min, λ = 220 nm, t (major) = 10.61 min, t (minor) = 12.47 min.

$[\alpha]_D^{25}$ = –1.57 (c = 0.38, $CHCl_3$); **^{1}H NMR** (400 MHz, $CDCl_3$) δ 8.01 – 7.90 (m, 2H), 7.69 – 7.54 (m, 2H), 7.53 – 7.39 (m, 3H), 7.23 – 7.14 (m, 1H), 7.05 (dd, *J* = 2.5, 1.0 Hz, 1H), 6.92 – 6.79 (m, 2H), 2.59 – 2.47 (m, 1H), 2.39 (s, 3H), 1.76 – 1.65 (m, 3H), 1.63 – 1.54 (m, 2H), 1.37 – 0.85 (m, 5H); **^{13}C NMR** (101 MHz, $CDCl_3$) δ 145.9 (d, *J* = 7.4 Hz), 143.7 (d, *J* = 3.1 Hz), 140.9 (d, *J* = 5.8 Hz), 140.67 (d, *J* = 10.4 Hz), 135.9, 134.5 (d, *J* = 3.3 Hz), 133.7 (d, *J* = 3.2 Hz), 132.4 (d, *J* = 11.0 Hz), 130.8, 128.8 (d, *J* = 16.1 Hz), 127.7, 126.45, 125.9 (d, *J* = 195 Hz), 125.5, 123.3, 121.8 (d, *J* = 2.5 Hz), 36.9, 33.3 (d, *J* = 8.4 Hz), 26.6, 26.0, 17.4; **^{31}P NMR** (162 MHz, $CDCl_3$) δ 13.23; **IR** (film) υ_{max}/cm^{-1}: 2927, 2159, 1535, 1481, 1357, 1277, 1213, 1167, 1130, 1105, 928; **HRMS** (ESI+): calcd. for $C_{25}H_{26}ClNO_5P$ 486.1237 $[M+H]^+$, found 486.1231.

(8aS)-1-Isopropyl-4b,8,8-trimethyl-4b,5,6,7,8,8a,9,10-octahydrophenanthren-2-yl (2-methyl-6-nitrophenyl) (*R*)-phenylphosphonate (**9**)

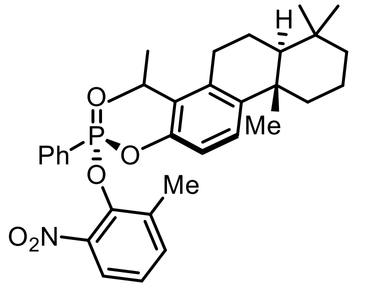

9 was synthesised following **GP II**. **P1** (43.4 mg, 0.100 mmol) was used as phosphonate, totarol (31.5 mg, 0.11 mmol) used as nucleophile and BIMP **B1**- P(*p*-tol)$_3$ (11.7 mg, 0.015 mmol) as catalyst in PhF (0.40 mL) at 23 °C. Pentane:EtOAc 7:3. Colourless oil (35.4 mg, 0.063 mmol, 63% yield, 97:3 dr).

[α]$_D^{25}$ = –20.2 (c = 0.65, $CHCl_3$); **^{1}H NMR** (400 MHz, $CDCl_3$) δ 8.00 (ddt, *J* = 14.4, 6.9, 1.4 Hz, 2H), 7.68 (dd, *J* = 8.1, 1.7 Hz, 1H), 7.62 (dq, *J* = 6.9, 1.7 Hz, 1H), 7.55 – 7.51 (m, 2H), 7.45 (s, 1H), 7.19 (td, *J* = 7.9, 1.2 Hz, 1H), 6.92 (d, *J* = 4.4 Hz, 2H), 3.29 (dq, *J* = 14.7, 7.4, 6.0 Hz, 1H), 2.93 (ddd, *J* = 17.2, 6.6, 1.7 Hz, 1H), 2.73 (ddd, *J* = 17.7, 11.2, 7.8 Hz, 1H), 2.34 (d, *J* = 3.4 Hz, 3H), 2.19 – 2.09 (m, 1H), 1.88 (dd, *J* = 13.4, 7.7 Hz, 1H), 1.77 – 1.52 (m, 3H), 1.44 (dtd, *J* = 13.2, 3.3, 1.3 Hz, 1H), 1.36 – 1.19 (m, 4H), 1.17 (d, *J* = 7.1 Hz, 3H), 1.13 (s, 3H), 0.92 (s, 3H), 0.90 (s, 3H); **^{13}C NMR** (126 MHz, $CDCl_3$) δ 147.4, 143.6, 141.4 (d, *J* = 9.1 Hz), 136.0 (d, *J* = 1.8 Hz), 135.1, 134.5 (d, *J* = 3.3 Hz), 133.4 (d, *J* = 3.3 Hz), 132.5, 132.4, 128.8, 128.7, 127.1 (d, *J* = 195.1 Hz), 125.2, 123.4 (d, *J* = 1.5 Hz), 123.3, 117.7 (d, *J* = 2.9 Hz), 53.6, 49.4, 41.6, 39.4, 38.1, 33.4, 33.3, 28.9, 25.1, 20.7, 20.7, 19.5, 19.4, 17.6; **^{31}P NMR** (162 MHz, $CDCl_3$) δ 12.51; **IR** (film) $υ_{max}$/cm^{-1}:2927, 1536, 1471, 1440, 1357, 1275, 1219, 1197, 1179, 1129, 982, 923, 804, 766, 740, 644; **HRMS** (ESI+): calcd. for $C_{33}H_{41}NO_5P$ 562.2717 $[M+H]^+$, found 562.2715.

2-Isopropyl-5-methylphenyl (2-methyl-6-nitrophenyl) *(R)*-(4-methoxyphenyl)phosphonate (**10**)

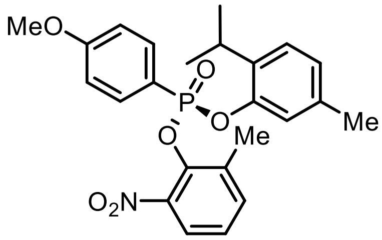

10 was synthesised following **GP II**. **P2** (45.8 mg, 0.10 mmol) was used as phosphonate, 2-isopropyl-5-methylphenol (16.52 mg, 0.11 mmol) used as nucleophile and BIMP **B1**- P(*p*-tol)$_3$ (11.7 mg, 0.015 mmol) as catalyst in PhF (0.40 mL) at 23 °C. Pentane:EtOAc 7:3. Colourless oil (29.0 mg, 0.064 mmol, 64% yield, 96% e.e.).

HPLC Conditions: CHIRALPAK AD-H, hexane/isopropanol = 85/15, 1 mL/min, λ = 220 nm, t (major) = 9.75 min, t (minor) = 11.28 min.

[α]$_D^{25}$ = –31.9 (c = 0.48, $CHCl_3$); **^{1}H NMR** (400 MHz, $CDCl_3$) δ 7.99 – 7.90 (m, 2H), 7.70 (dd, *J* = 8.1, 1.7 Hz, 1H), 7.47 (ddt, *J* = 7.7, 1.7, 0.8 Hz, 1H), 7.21 (td, *J* = 7.9, 1.3 Hz, 1H), 7.09 – 7.05 (m, 1H), 7.04 – 6.98 (m, 2H), 6.88 (t, *J* = 3.5 Hz, 2H), 3.88 (s, 3H), 3.05 (p, *J* = 6.9 Hz, 1H), 2.44 (d, *J* = 1.0 Hz, 3H), 2.17 (s, 3H), 1.05 (dd, *J* = 49.8, 6.9 Hz, 6H); **^{13}C NMR** (101 MHz, $CDCl_3$) δ 163.9 (d, *J* = 3.6 Hz), 147.6 (d, *J* = 7.3 Hz), 143.9, 141.3 (d, *J* = 10.2 Hz), 136.7, 136.5 (d, *J* = 5.9 Hz), 135.9 (d, *J* = 2.0 Hz), 134.7 (d, *J* = 2.0 Hz), 134.6, 126.6, 126.2, 125.3 (d, *J* = 1.7 Hz), 123.3 (d, *J* = 1.7 Hz), 120.8 (d, *J* = 2.6 Hz), 117.5 (d, *J* = 203.2 Hz), 114.4 (d, *J* = 17.3 Hz), 55.6, 26.4, 23.3, 23.2, 21.0, 17.6; **^{31}P NMR** (162 MHz, $CDCl_3$) δ 13.88; **IR** (film) υ_{max}/cm^{-1}: 2965, 1599, 1535, 1506, 1357, 1259, 1219, 1181, 1130, 1087, 924, 911; **HRMS** (ESI+): calcd. for $C_{24}H_{27}NO_6P$ 456.1571 $[M+H]^+$, found 456.1562.

5-Isopropyl-2-methylphenyl (2-methyl-6-nitrophenyl) *(R)*- (4-methoxyphenyl)phosphonate **(11)**

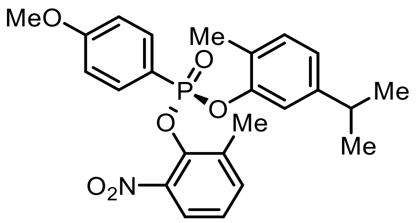

11 was synthesised following **GP II**. **P2** (45.8 mg, 0.10 mmol) was used as phosphonate, 5-isopropyl-2-methylphenol (16.9 µL, 0.11 mmol) used as nucleophile and BIMP **B1**- P(*p*-tol)$_3$ (11.7 mg, 0.015 mmol) as catalyst in PhF (0.40 mL) at 23 °C. Pentane:EtOAc 7:3. Colourless oil (22.7 mg, 0.05 mmol, 50% yield, 70% e.e.).

HPLC Conditions: CHIRALPAK AD-H, hexane/isopropanol = 85/15, 1 mL/min, λ = 220 nm, t (major) = 9.40 min, t (minor) = 10.67 min.

$[\alpha]_D^{25}$ = –14.7(c = 0.21, $CHCl_3$); **^{1}H NMR** (400 MHz, $CDCl_3$) δ 8.01 – 7.91 (m, 2H), 7.70 (dd, *J* = 8.1, 1.7 Hz, 1H), 7.47 (ddt, *J* = 7.7, 1.6, 0.7 Hz, 1H), 7.21 (td, *J* = 7.9, 1.3 Hz, 1H), 7.02 (m, 3H), 6.91 – 6.83 (m, 2H), 3.88 (s, 3H), 2.72 (p, *J* = 6.9 Hz, 1H), 2.44 (d, *J* = 1.0 Hz, 3H), 2.09 (s, 3H), 1.09 (dd, *J* = 6.9, 1.3 Hz, 6H); **^{13}C NMR** (101 MHz, $CDCl_3$) δ 163.7 (d, *J* = 3.5 Hz), 148.9 (d, *J* = 7.2 Hz), 148.1, 141.1 (d, *J* = 10.2 Hz), 135.8 (d, *J* = 1.9 Hz), 134.6, 134.5, 131.1, 126.5 (d, *J* = 5.8 Hz), 125.1 (d, *J* = 2.0 Hz), 123.2 (d, *J* = 1.7 Hz), 123.0, 118.2 (d, *J* = 2.9 Hz), 117.4 (d, *J* = 203.0 Hz), 114.3, 114.2, 55.4, 33.5, 23.8, 23.7, 17.4, 16.0; **^{31}P NMR** (162 MHz, $CDCl_3$) δ 14.18; **IR** (film) υ_{max}/cm^{-1}:1599, 1532, 1506, 1358, 1259, 1131, 924; **HRMS** (ESI+): calcd. for $C_{24}H_{27}NO_6P$ 456.1571 $[M+H]^+$, found 456.1565.

Products: R group Scope

2,4-Dimethylphenyl (2-methyl-6-nitrophenyl) *(R)*- (4-methoxyphenyl)phosphonate (**12**)

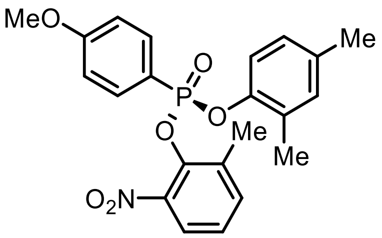

12 was synthesised following **GP II**. **P2** (45.8 mg, 0.10 mmol) was used as phosphonate, 2,4-dimethylphenol (12.4 µL, 0.11 mmol) used as nucleophile and BIMP **B1** - P(*p*-tol)$_3$ (11.7 mg, 0.015 mmol) as catalyst in PhF (0.40 mL) at 23 °C. Pentane:EtOAc 7:3. Colourless oil (29.4 mg, 0.069 mmol, 69% yield, 97% e.e.).

HPLC Conditions: CHIRALPAK AD-H, hexane/isopropanol = 70/30, 1 mL/min, λ = 220 nm, t (major) = 11.73 min, t (minor) = 21.94 min.

$[\alpha]_D^{25}$ = –15.5 (c = 0.65, $CHCl_3$); **^{1}H NMR** (400 MHz, $CDCl_3$) δ 8.00 – 7.90 (m, 2H), 7.73 – 7.68 (m, 1H), 7.47 (ddt, *J* = 7.7, 1.6, 0.7 Hz, 1H), 7.22 (td, *J* = 7.9, 1.3 Hz, 1H), 7.02 (ddd, *J* = 8.7, 4.2, 2.3 Hz, 2H), 6.92 – 6.85 (m, 2H), 6.79 (dd, *J* = 8.4, 2.3 Hz, 1H), 3.88 (s, 3H), 2.45 (d, *J* = 0.9 Hz, 3H), 2.24 – 2.14 (m, 3H), 2.05 (s, 3H);**^{13}C NMR** (101 MHz, $CDCl_3$) δ 163.9 (d, *J* = 3.5 Hz), 146.9 (d, *J* = 7.0 Hz), 144.0, 141.2 (d, *J* = 10.0 Hz), 136.0 (d, *J* = 2.0 Hz), 134.7 (d, *J* = 12.3 Hz), 134.7 (d, *J* = 3.3 Hz), 132.1, 129.2 (d, *J* = 5.6 Hz), 127.4 (d, *J* = 1.5 Hz), 125.3 (d, *J* = 1.9 Hz), 123.3, 123.3 (d, *J* = 1.8 Hz), 117.5 (d, *J* = 202.5 Hz), 114.4 (d, *J* = 17.2 Hz), 125.4, 55.5, 20.7, 17.6, 16.4; **^{31}P NMR** (162 MHz, $CDCl_3$) δ 14.32; **IR** (film) υ_{max}/cm^{-1}:1599, 1534, 1404, 1358, 1259, 1181, 1130, 923, 908, 765; **HRMS** (ESI+): calcd. for $C_{22}H_{23}NO_6P$ 428.1258 $[M+H]^+$, found 428.1255.

2,4-Dimethylphenyl (2-methyl-6-nitrophenyl) *(R)*-methylphosphonate (**13**)

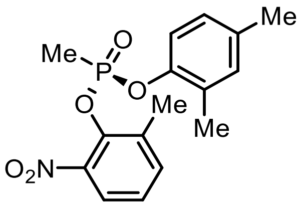

13 was synthesised following **GP II**. **P3** (36.6 mg, 0.10 mmol) was used as phosphonate, 2,4-dimethylphenol (12.4 µL, 0.11 mmol) used as nucleophile and BIMP **B1** - P(*p*-tol)$_3$ (11.7 mg, 0.015 mmol) as catalyst in PhF (0.40 mL) at 23 °C. Pentane:EtOAc 7:3. Colourless oil (23.7 mg, 0.071 mmol, 71% yield, 84% e.e.).

HPLC Conditions: CHIRALPAK AS-H, hexane/isopropanol = 90/10, 1 mL/min, λ = 220 nm, t (major) = 8.93 min, t (minor) = 11.15 min.

[α]$_D^{25}$ = –55.0 (c = 0.76, $CHCl_3$); **^{1}H NMR** (400 MHz, $CDCl_3$) δ 7.82 – 7.71 (m, 1H), 7.47 (ddt, *J* = 7.7, 1.7, 0.8 Hz, 1H), 7.21 (td, *J* = 7.9, 1.3 Hz, 1H), 7.05 – 6.98 (m, 2H), 6.89 (dd, *J* = 8.3, 2.3 Hz, 1H), 2.41 (d, *J* = 0.9 Hz, 3H), 2.26 (d, *J* = 0.9 Hz, 3H), 2.20 (s, 3H), 1.90 (d, *J* = 17.8 Hz, 3H); **^{13}C NMR** (101 MHz, $CDCl_3$) δ 146.8 (d, *J* = 7.6 Hz), 143.1, 141.6 (d, *J* = 10.3 Hz), 136.3 (d, *J* = 1.9 Hz), 135.0, 134.3 (d, *J* = 3.2 Hz), 132.3, 128.9 (d, *J* = 5.7 Hz), 127.7 (d, *J* = 1.5 Hz), 125.3 (d, *J* = 1.9 Hz), 123.5 (d, *J* = 1.6 Hz), 119.8 (d, *J* = 2.5 Hz), 20.8, 17.4, 16.4, 12.3 (d, *J* = 144.2 Hz);**^{31}P NMR** (162 MHz, $CDCl_3$) δ 26.08; **IR** (film) $υ_{max}$/cm^{-1}: 1535, 1356, 1268, 1223, 1199, 1183, 1119, 920; **HRMS** (ESI+): calcd. for $C_{16}H_{19}NO_5P$ 336.0995 [M+H]$^+$, found 336.0991.

2,4-Dimethylphenyl (2-methyl-6-nitrophenyl) *(R)*-ethylphosphonate (**14**)

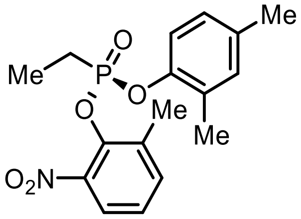

14 was synthesised following **GP II**. **P4** (38.0 mg, 0.10 mmol) was used as phosphonate, 2,4-dimethylphenol (12.4 µL, 0.11 mmol) used as nucleophile and BIMP **B1** - P(*p*-tol)$_3$ (11.7 mg, 0.015 mmol) as catalyst in PhF (0.40 mL) at 23 °C. Pentane:EtOAc 7:3. Colourless oil (22.4mg, 0.063 mmol, 63% yield, 93% e.e.).

HPLC Conditions: CHIRALPAK AS-H, hexane/isopropanol =90/10, 1 mL/min, λ = 220 nm, t (major) = 6.75 min, t (minor) = 7.39 min.

$[\alpha]_D^{25}$ = –69.2 (c = 0.52, $CHCl_3$); **^{1}H NMR** (400 MHz, $CDCl_3$) δ 7.73 (dd, *J* = 8.1, 1.7 Hz, 1H), 7.44 (ddt, *J* = 7.8, 1.7, 0.8 Hz, 1H), 7.20 (td, *J* = 7.9, 1.2 Hz, 1H), 7.01 (dd, *J* = 8.4, 1.2 Hz, 1H), 6.98 – 6.94 (m, 1H), 6.84 (dd, *J* = 8.3, 2.3 Hz, 1H), 2.38 (s, 3H), 2.27 – 2.22 (m, 4H), 2.21 – 2.13 (m, 4H), 1.37 (dt, *J* = 21.8, 7.7 Hz, 3H);**^{13}C NMR** (101 MHz, $CDCl_3$) δ 147.0 (d, *J* = 8.1 Hz), 143.5, 141.3 (d, *J* = 10.7 Hz), 136.1 (d, *J* = 1.9 Hz), 134.7, 134.4 (d, *J* = 3.2 Hz), 132.2, 128.6 (d, *J* = 6.0 Hz), 127.6, 125.2 (d, *J* = 1.9 Hz), 123.4 (d, *J* = 1.6 Hz), 119.5 (d, *J* = 2.4 Hz), 20.7, 20.1 (d, *J* = 141.3 Hz), 17.3, 16.4, 6.8 (d, *J* = 7.6 Hz);**^{31}P NMR** (162 MHz, $CDCl_3$) δ 29.03; **IR** (film) υ_{max}/cm^{-1}: 1535, 1357, 1183, 924, 909, 775; **HRMS** (ESI+): calcd. for $C_{17}H_{21}NO_5P$ 350.1152 $[M+H]^+$, found 350.1151.

2,4-Dimethylphenyl (2-methyl-6-nitrophenyl) *(R)*-isopropylphosphonate (**15**)

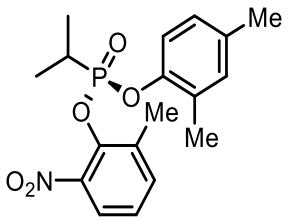

15 was synthesised following **GP II**. **P5** (39.4 mg, 0.10 mmol) was used as phosphonate, 2,4-dimethylphenol (12.4 µL, 0.11 mmol) used as nucleophile and BIMP **B1** - P(*p*-tol)$_3$ (11.7 mg, 0.015 mmol) as catalyst in PhF (0.40 mL) at 23 °C. Pentane:EtOAc 7:3. Yellow oil (2.5 mg, 0.007 mmol, 7% yield, 54% e.e.).

HPLC Conditions: CHIRALPAK AD-H, hexane/isopropanol = 85/15, 1 mL/min, λ = 220 nm, t (major) = 11.11 min, t (minor) = 12.95 min

^{1}**H NMR** (600 MHz, $CDCl_3$) δ 7.71 – 7.67 (m, 1H), 7.45 – 7.40 (m, 1H), 7.22 – 7.16 (m, 1H), 6.97 – 6.92 (m, 2H), 6.78 (dd, *J* = 8.5, 2.3 Hz, 1H), 2.48 – 2.39 (m, 1H), 2.38 (s, 3H), 2.22 (s, 3H), 2.16 (s, 3H), 1.45 (t, *J* = 7.2 Hz, 3H), 1.42 (t, *J* = 7.2 Hz, 3H); ^{13}**C NMR** (151 MHz, $CDCl_3$) δ 147.5 (d, *J* = 8.8 Hz), 143.8 (d, *J* = 3.7 Hz), 141.0 (d, *J* = 11.7 Hz), 135.9 (d, *J* = 2.0 Hz), 134.4 (d, *J* = 3.2 Hz), 134.3, 132.1, 128.3 (d, *J* = 6.4 Hz), 127.5, 125.2 (d, *J* = 1.7 Hz), 123.3 (d, *J* = 1.4 Hz), 119.2 (d, *J* = 2.2 Hz), 27.4 (d, *J* = 140.5 Hz), 20.7, 17.2, 16.4, 16.4 (d, *J* = 5.2 Hz), 16.4 (d, *J* = 5.4 Hz); ^{31}**P NMR** (162 MHz, $CDCl_3$) δ 30.72.; **IR** (film) $υ_{max}$/cm^{-1}: 2980, 1606, 1585, 1498, 1471, 1357, 1296, 1249, 1222, 1198, 1182, 1156, 1120, 1092, 1045, 948, 910, 803, 765, 743, 706, 669; **HRMS** (ESI+): calcd. for $C_{18}H_{23}NO_5P$ 364.1308 $[M+H]^+$, found 364.1308.

2,4-Dimethylphenyl (2-methyl-6-nitrophenyl) *(R)*-hexylphosphonate (**16**)

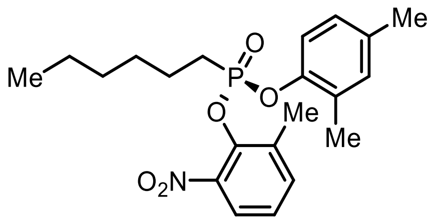

16 was synthesised following **GP II**. **P6** (43.6 mg, 0.10 mmol) was used as phosphonate, 2,4-dimethylphenol (12.4 µL, 0.11 mmol) used as nucleophile and BIMP **B1** - P(*p*-tol)$_3$ (11.7 mg, 0.015 mmol) as catalyst in PhF (0.40 mL) at 23 °C. Pentane:EtOAc 7:3. Colourless oil (24.2 mg, 0.06 mmol, 60% yield, 94% e.e.).

HPLC Conditions: CHIRALPAK AD-H, hexane/isopropanol = 85/15, 1 mL/min, λ = 220 nm, t (major) = 12.43 min, t (minor) = 15.35 min.

[α]$_D^{25}$ = –51.5 (c = 0.79, CHCl$_3$); **^{1}H NMR** (400 MHz, CDCl$_3$) δ 7.73 (dd, *J* = 8.1, 1.7 Hz, 1H), 7.44 (ddt, *J* = 7.6, 1.6, 0.7 Hz, 1H), 7.19 (td, *J* = 7.9, 1.2 Hz, 1H), 7.00 (dd, *J* = 8.3, 1.2 Hz, 1H), 6.97 – 6.94 (m, 1H), 6.84 (dd, *J* = 8.3, 2.3 Hz, 1H), 2.38 (s, 3H), 2.25 – 2.23 (m, 3H), 2.21 – 2.10 (m, 5H), 1.80 (dddd, *J* = 15.2, 11.6, 7.7, 5.9 Hz, 2H), 1.45 (p, *J* = 7.3 Hz, 2H), 1.31 (ddt, *J* = 7.2, 5.0, 2.7 Hz, 4H), 0.93 – 0.85 (m, 3H); **^{13}C NMR** (101 MHz, CDCl$_3$) δ 147.1 (d, *J* = 8.2 Hz), 143.4, 141.4 (d, *J* = 10.9 Hz), 136.1 (d, *J* = 2.0 Hz), 134.6, 134.4 (d, *J* = 3.2 Hz), 132.2, 128.6 (d, *J* = 6.1 Hz), 127.6, 125.2 (d, *J* = 1.9 Hz), 123.4 (d, *J* = 1.6 Hz), 119.5 (d, *J* = 2.4 Hz), 31.3 (d, *J* = 1.3 Hz), 30.3 (d, *J* = 18.2 Hz), 26.8 (d, *J* = 138.5 Hz), 22.5, 22.4, 20.7, 17.3, 16.4, 14.1; **^{31}P NMR** (162 MHz, CDCl$_3$) δ 28.03; **IR** (film) υ_{max}/cm^{-1}: 2928, 1536, 1356, 1199, 1120, 948, 924, 909, 804, 760; **HRMS** (ESI+): calcd. for $C_{21}H_{29}NO_5P$ 406.1778 [M+H]$^+$, found 406.1776.

2,4-Dimethylphenyl (2-methyl-6-nitrophenyl) (*R*)-(cyclopropylmethyl)phosphonate (**17**)

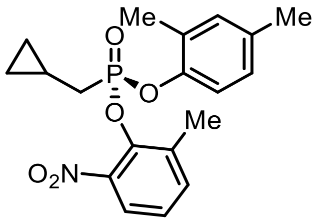

17 was synthesised following **GP II**. **P7** (40.6 mg, 0.10 mmol) was used as phosphonate, 2,4-dimethylphenol (12.4 µL, 0.11 mmol) used as nucleophile and BIMP **B1** - P(*p*-tol)$_3$ (11.7 mg, 0.015 mmol) as catalyst in PhF (0.40 mL) at 23 °C. Pentane:EtOAc 7:3. Colourless oil (17.1 mg, 0.046 mmol, 46% yield, 88% e.e.).

HPLC Conditions: CHIRALPAK AD-H, hexane/isopropanol = 80/20, 1 mL/min, λ = 220 nm, t (major) = 13.06 min, t (minor) = 14.53 min.

$[\alpha]_D^{25}$ = –76.2 (c = 0.34, $CHCl_3$); **^{1}H NMR** (400 MHz, $CDCl_3$) δ 7.74 (dd, *J* = 8.1, 1.6 Hz, 1H), 7.48 – 7.41 (m, 1H), 7.20 (td, *J* = 7.9, 1.2 Hz, 1H), 7.06 (dd, *J* = 8.2, 1.1 Hz, 1H), 6.99 – 6.94 (m, 1H), 6.84 (dd, *J* = 8.4, 2.3 Hz, 1H), 2.39 (s, 3H), 2.24 (s, 3H), 2.18 (s, 4H), 2.20 – 2.11 (m, 1H), 1.19 – 1.04 (m, 1H), 0.72 – 0.59 (m, 2H), 0.40 – 0.26 (m, 2H).;**^{13}C NMR** (101 MHz, $CDCl_3$) δ 147.1 (d, *J* = 8.0 Hz), 143.5 – 142.8 (m), 141.3 (d, *J* = 11.2 Hz), 136.0, 134.4, 134.2 (d, *J* = 3.2 Hz), 132.0, 128.4 (d, *J* = 6.6 Hz), 127.4, 125.0, 123.3, 119.2 (d, *J* = 2.9 Hz), 31.8 (d, *J* = 138.9 Hz), 20.6, 17.2, 16.3, 5.6 (dd, *J* = 10.4, 3.0 Hz), 4.3 (d, *J* = 5.6 Hz); **^{31}P NMR** (162 MHz, $CDCl_3$) δ 26.12; **IR** (film) υ_{max}/cm^{-1}:1535, 1498, 1356, 1279, 1222, 1199, 1182, 1119, 923, 799, 762; **HRMS** (ESI+): calcd. for $C_{19}H_{23}NO_5P$ 376.1314 $[M+H]^+$, found 376.1308.

2,4-dimethylphenyl (2-methyl-6-nitrophenyl) (*R*)-benzylphosphonate (**18**)

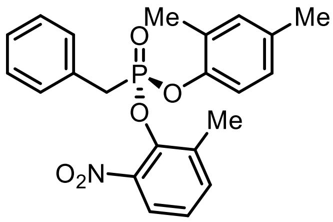

18 was synthesised following **GP II**. **P8** (44.2 mg, 0.100 mmol) was used as phosphonate, 2,4-dimethylphenol (12.4 µL, 0.11 mmol) used as nucleophile and BIMP **B1** - P(*p*-tol)$_3$ (11.7 mg, 0.015 mmol) as catalyst in PhF (0.40 mL) at 23 °C. Pentane:EtOAc 7:3. Colourless oil (30.1 mg, 0.073 mmol, 73% yield, 82% e.e.).

HPLC Conditions: CHIRALPAK AD-H, hexane/isopropanol = 70/30, 1 mL/min, λ = 220 nm, t (minor) = 12.86 min, t (major) = 15.61 min.

$[\alpha]_D^{25}$ = –84.8 (c = 0.21, $CHCl_3$); **^{1}H NMR** (400 MHz, $CDCl_3$) δ 7.76 (dd, *J* = 8.1, 1.7 Hz, 1H), 7.45 – 7.38 (m, 1H), 7.40 – 7.23 (m, 5H), 7.20 (td, *J* = 7.9, 1.2 Hz, 1H), 6.96 – 6.87 (m, 2H), 6.79 (dd, *J* = 8.3, 2.3 Hz, 1H), 3.72 – 3.54 (m, 2H), 2.23 (s, 3H), 2.21 (s, 3H), 1.93 (s, 3H).;**^{13}C NMR** (101 MHz, $CDCl_3$) δ 147.0 (d, *J* = 8.4 Hz), 143.1, 141.4 (d, *J* = 11.0 Hz), 136.1 (d, *J* = 1.8 Hz), 134.5, 134.2 (d, *J* = 3.4 Hz), 132.0, 130.2 (d, *J* = 7.2 Hz), 130.1 (d, *J* = 10.1 Hz), 128.6 (d, *J* = 3.2 Hz), 128.6, 127.3, 127.3, 125.1 (d, *J* = 1.5 Hz), 123.4 (d, *J* = 1.3 Hz), 119.0 (d, *J* = 2.4 Hz), 34.1 (d, *J* = 137.7 Hz), 20.6, 17.0, 16.0.;**^{31}P NMR** (162 MHz, $CDCl_3$) δ 21.15; **IR** (film) υ_{max}/cm^{-1}:2160, 1534, 1356, 1276, 1221, 923; **HRMS** (ESI+): calcd. for $C_{22}H_{22}NNaO_5P$ 434.1133 $[M+Na]^+$, found 434.1127.

2,4-Dimethylphenyl (2-methyl-6-nitrophenyl) *(R)*- (2-methylbenzyl)phosphonate (**19**)

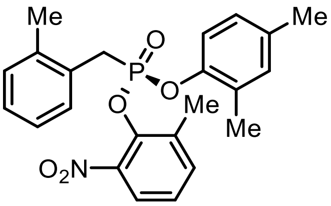

19 was synthesised following **GP II**. **P9** (45.6 mg, 0.10 mmol) was used as phosphonate, 2,4-dimethylphenol (12.4 µL, 0.11 mmol) used as nucleophile and BIMP **B1** - P(*p*-tol)$_3$ (11.7 mg, 0.015 mmol) as catalyst in PhF (0.40 mL) at 23 °C. Pentane:EtOAc 7:3. Colourless oil (17.7 mg, 0.042 mmol, 42% yield, 86% e.e.).

HPLC Conditions: CHIRALPAK AD-H, hexane/isopropanol = 80/20, 1 mL/min, λ = 220 nm, t (major) = 15.19 min, t (minor) = 22.72 min.

[α]$_D^{25}$ = –42.3 (c = 0.23, CHCl$_3$); **^{1}H NMR** (400 MHz, CDCl$_3$) δ 7.75 (dd, *J* = 8.1, 1.7 Hz, 1H), 7.44 – 7.35 (m, 2H), 7.22 – 7.10 (m, 4H), 6.91 – 6.88 (m, 1H), 6.82 (dd, *J* = 8.3, 1.2 Hz, 1H), 6.75 (dd, *J* = 8.4, 2.3 Hz, 1H), 3.66 (d, *J* = 22.3 Hz, 2H), 2.41 (d, *J* = 1.9 Hz, 3H), 2.22 – 2.20 (m, 3H), 2.20 – 2.17 (m, 3H), 1.94 (s, 3H); **^{13}C NMR** (126 MHz, CDCl$_3$) δ 147.1 (d, *J* = 9.0 Hz), 143.3 (d, *J* = 3.7 Hz), 141.4 (d, *J* = 11.4 Hz), 137.6 (d, *J* = 7.7 Hz), 136.2 (d, *J* = 1.8 Hz), 134.6, 134.4 (d, *J* = 3.2 Hz), 132.1, 131.1 (d, *J* = 6.0 Hz), 130.8 (d, *J* = 3.6 Hz), 128.8 (d, *J* = 10.1 Hz), 128.7 (d, *J* = 6.4 Hz), 127.7 (d, *J* = 4.1 Hz), 127.5, 126.3 (d, *J* = 3.8 Hz), 125.2 (d, *J* = 1.8 Hz), 123.5 (d, *J* = 1.6 Hz), 119.2 (d, *J* = 2.5 Hz), 31.6 (d, *J* = 138.5 Hz), 20.7, 20.1, 17.0, 16.1; **^{31}P NMR** (162 MHz, CDCl$_3$) δ 21.57; **IR** (film) υ_{max}/cm^{-1}: 1535, 1497, 1356, 1275, 1223, 1198, 951, 912; **HRMS** (ESI+): calcd. for $C_{23}H_{25}NO_5P$ 426.1465 [M+H]$^+$, found 426.1461.

2,4-Dimethylphenyl (2-methyl-6-nitrophenyl) *(R)*- (2-(trifluoromethyl)benzyl)phosphonate (**20**)

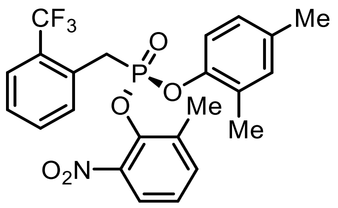

20 was synthesised following **GP II**. **P10** (51.0 mg, 0.10 mmol) was used as phosphonate, 2,4-dimethylphenol (12.4 µL, 0.11 mmol) used as nucleophile and BIMP **B1** - P(*p*-tol)$_3$ (11.7 mg, 0.015 mmol) as catalyst in PhF (0.40 mL) at 23 °C. Pentane:EtOAc 7:3. Colourless oil (20.6 mg, 0.043 mmol, 43% yield, 78% e.e.).

HPLC Conditions: CHIRALPAK AD-H, hexane/isopropanol = 80/20, 1 mL/min, λ = 220 nm, t (major) = 14.84 min, t (minor) = 12.87 min.

$[\alpha]_D^{25}$ = –61.0 (c = 0.66, $CHCl_3$); **^{1}H NMR** (400 MHz, $CDCl_3$) δ 7.79 (dd, *J* = 8.2, 1.7 Hz, 1H), 7.60 (d, *J* = 7.7 Hz, 1H), 7.57 – 7.48 (m, 2H), 7.48 – 7.39 (m, 2H), 7.21 (td, *J* = 7.9, 1.2 Hz, 1H), 6.99 (dd, *J* = 8.3, 1.2 Hz, 1H), 6.91 (dd, *J* = 2.2, 1.1 Hz, 1H), 6.81 (dd, *J* = 8.3, 2.3 Hz, 1H), 3.78 – 3.62 (m, 2H), 2.23 (s, 3H), 2.21 (s, 3H), 1.91 (s, 3H); **^{13}C NMR** (126 MHz, $CDCl_3$) δ 146.9 (d, *J* = 8.4 Hz), 143.0 (d, *J* = 3.7 Hz), 141.4 (d, *J* = 10.9 Hz), 136.4 (d, *J* = 1.8 Hz), 134.9, 134.2 (d, *J* = 3.2 Hz), 133.7 (d, *J* = 6.7 Hz), 132.3, 131.5 (d, *J* = 9.9 Hz), 131.1 (dd, *J* = 32.6, 3.1 Hz), 129.3 (d, *J* = 3.2 Hz), 128.6 (d, *J* = 6.8 Hz), 127.6, 127.0 – 126.6 (m), 125.4 (d, *J* = 1.8 Hz), 124.4 (t, *J* = 3.8 Hz), 124.0 (q, *J* = 272.4 Hz), 123.6 (d, *J* = 1.8 Hz), 118.9 (d, *J* = 2.6 Hz), 34.0 (d, *J* = 138.1 Hz), 20.7, 17.0, 16.1; **^{19}F NMR** (377 MHz, $CDCl_3$) δ -62.74; **^{31}P NMR** (162 MHz, $CDCl_3$) δ 19.65; **IR** (film) υ_{max}/cm^{-1}: 1536, 1356, 1331, 1122, 952, 915; **HRMS** (ESI+): calcd. for $C_{23}H_{21}F_3NO_5P$ 480.1181 $[M+H]^+$, found 480.1178.

2,4-Dimethylphenyl (2-methyl-6-nitrophenyl) (*R*)-thiophen-3-ylphosphonate (**21**)

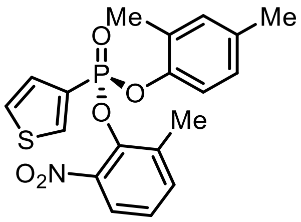

21 was synthesised following **GP II**. **P11** (43.4 mg, 0.100 mmol) was used as phosphonate, 2,4-dimethylphenol (12.4 µL, 0.11 mmol) used as nucleophile and BIMP **B1** - P(*p*-tol)$_3$ (11.7 mg, 0.015 mmol) as catalyst in PhF (0.40 mL) at 23 °C. Pentane:EtOAc 7:3. Colourless oil (30.3 mg, 0.075 mmol, 75% yield, 92% e.e.).

HPLC Conditions: CHIRALPAK AD-H, hexane/isopropanol = 70/30 1 mL/min, λ = 220 nm, t (major) = 10.70 min, t (minor) = 15.49 min.

$[\alpha]_D^{25}$ = – 1.9 (c = 0.62, CHCl$_3$); **^{1}H NMR** (400 MHz, CDCl$_3$) δ 8.20 (ddd, *J* = 8.7, 2.9, 1.2 Hz, 1H), 7.71 (dd, *J* = 8.1, 1.7 Hz, 1H), 7.55 (ddd, *J* = 5.1, 4.0, 1.2 Hz, 1H), 7.53 – 7.44 (m, 2H), 7.22 (td, *J* = 7.9, 1.3 Hz, 1H), 6.95 – 6.85 (m, 2H), 6.82 (dd, *J* = 8.4, 2.2 Hz, 1H), 2.44 (s, 3H), 2.22 (s, 3H), 2.06 (s, 3H); **^{13}C NMR** (101 MHz, CDCl$_3$) δ 146.7 (d, *J* = 7.4 Hz), 143.8, 141.0 (d, *J* = 9.8 Hz), 137.9 (d, *J* = 19.9 Hz), 136.1 (d, *J* = 2.0 Hz), 134.9 (d, *J* = 1.6 Hz), 134.6 (d, *J* = 3.5 Hz), 132.2, 129.4, 129.1 (d, *J* = 5.6 Hz), 127.7 (d, *J* = 21.2 Hz), 127.5, 127.3 (d, *J* = 205.4 Hz), 125.5 (d, *J* = 2.0 Hz), 123.3 (d, *J* = 1.8 Hz), 120.0 (d, *J* = 2.6 Hz), 20.8, 17.5, 16.4; **^{31}P NMR** (162 MHz, CDCl$_3$) δ 6.71; **IR** (film) υ_{max}/cm^{-1}:1534, 1496, 1356, 1272, 1221, 1196, 1181, 117, 1090, 931, 913, 801, 764, 736, 631; **HRMS** (ESI+): calcd. for $C_{19}H_{19}NO_5PS$ 404.0722 [M+H]$^+$, found 404.0715.

2,4-Dimethylphenyl (2-methyl-6-nitrophenyl) *(R)*- ((methylthio)methyl)phosphonate (**22**)

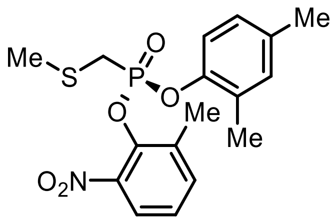

22 was synthesised following **GP II**. **P12** (41.2 mg, 0.10 mmol) was used as phosphonate, 2,4-dimethylphenol (12.4 µL, 0.11 mmol) used as nucleophile and BIMP **B1** - P(*p*-tol)$_3$ (11.7 mg, 0.015 mmol) as catalyst in PhF (0.40 mL) at 23 °C. Pentane:EtOAc 7:3. Colourless oil (29.2 mg, 0.077 mmol, 77% yield, 92% e.e.).

SFC Conditions: CHIRALPAK ID, 1500 psi, 30 °C, flow : 1.5 mL/min, from 1% to 30% MeOH in 5 mins, λ = 220 nm, t (minor) = 3.91 min, t (major) = 4.22 min

$[\alpha]_D^{25}$ = –41.0 (c = 0.73, $CHCl_3$); **^{1}H NMR** (400 MHz, $CDCl_3$) δ 7.79 – 7.73 (m, 1H), 7.46 (ddt, *J* = 7.7, 1.8, 0.8 Hz, 1H), 7.21 (td, *J* = 7.9, 1.2 Hz, 1H), 7.10 (dd, *J* = 8.3, 1.2 Hz, 1H), 6.99 – 6.95 (m, 1H), 6.90 – 6.84 (m, 1H), 3.14 (d, *J* = 12.3 Hz, 2H), 2.39 (d, *J* = 0.9 Hz, 3H), 2.33 (d, *J* = 1.5 Hz, 3H), 2.25 (d, *J* = 0.9 Hz, 3H), 2.20 (s, 3H); **^{13}C NMR** (101 MHz, $CDCl_3$) δ 147.1 (d, *J* = 8.6 Hz), 143.0, 141.6 (d, *J* = 10.9 Hz), 136.4 (d, *J* = 1.9 Hz), 134.9, 134.3 (d, *J* = 3.3 Hz), 132.2, 128.8 (d, *J* = 6.1 Hz), 127.6, 125.4 (d, *J* = 1.8 Hz), 123.6 (d, *J* = 1.5 Hz), 119.5 (d, *J* = 2.4 Hz), 28.2 (d, *J* = 150.4 Hz), 20.8, 17.6 (d, *J* = 2.2 Hz), 17.3, 16.5; **^{31}P NMR** (162 MHz, $CDCl_3$) δ 18.97; **IR** (film) υ_{max}/cm^{-1}: 1534, 1497, 1355, 1274, 1222, 1199, 1181, 1116, 951, 928, 912; **HRMS** (ESI+): $C_{17}H_{21}NO_5PS$ 382.0873 $[M+H]^+$, found 382.0881.

2,4-Dimethylphenyl (2-methyl-6-nitrophenyl) (*R*)-((benzyloxy)methyl)phosphonate (**23**)

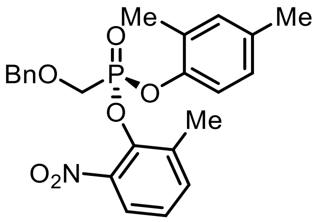

23 was synthesised following **GP II**. **P13** (47.2 mg, 0.100 mmol) was used as phosphonate, 2,4-dimethylphenol (12.4 µL, 0.11 mmol) used as nucleophile and BIMP **B1** - P(*p*-tol)$_3$ (11.7 mg, 0.015 mmol) as catalyst in PhF (0.40 mL) at 23 °C. Pentane:EtOAc 7:3. Colourless oil (31.8 mg, 0.072 mmol, 72% yield, 83% e.e.).

HPLC Conditions: CHIRALPAK AD-H, hexane/isopropanol = 80/20, 1 mL/min, λ = 220 nm, t (major) = 25.09 min, t (minor) = 35.82 min.

$[\alpha]_D^{25}$ = –21.9 (c = 1.20, $CHCl_3$); **^{1}H NMR** (400 MHz, $CDCl_3$) δ 7.70 (dd, *J* = 8.2, 1.5 Hz, 1H), 7.42 – 7.34 (m, 1H), 7.31 – 7.07 (m, 6H), 7.03 (d, *J* = 8.3 Hz, 1H), 6.89 (s, 1H), 6.79 (dd, *J* = 8.3, 2.1 Hz, 1H), 4.63 – 4.52 (m, 2H), 4.17 – 3.99 (m, 2H), 2.35 (s, 3H), 2.19 (s, 3H), 2.08 (s, 3H); **^{13}C NMR** (101 MHz, $CDCl_3$) 146.7 (d, *J* = 8.0 Hz), 142.9, 141.3 (d, *J* = 10.5 Hz), 136.5, 136.2, 134.9, 134.2 (d, *J* = 3.2 Hz), 132.1, 129.0 (d, *J* = 5.6 Hz), 128.4, 128.1, 128.1, 127.4, 125.3, 123.5, 119.7 (d, *J* = 2.4 Hz), 75.2 (d, *J* = 12.7 Hz), 63.8 (d, *J* = 166.2 Hz), 20.7, 17.3, 16.2; **^{31}P NMR** (162 MHz, $CDCl_3$) δ 16.26; **IR** (film) υ_{max}/cm^{-1}:1535, 1497, 1355, 1264, 1197, 1117, 928, 804, 753; **HRMS** (ESI+): calcd. for $C_{23}H_{24}NNaO_6P$ 464.1239 $[M+Na]^+$, found 464.1231.

2,4-dimethylphenyl (2-methyl-6-nitrophenyl) (*R*)-(((tert-butyldiphenylsilyl)oxy)methyl)phosphonate (**24**)

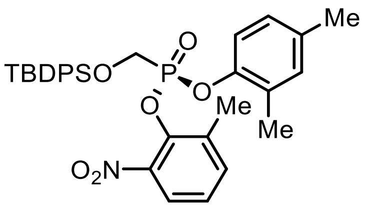

24 was synthesised following **GP II**. **P14** (62.1 mg, 0.10 mmol) was used as phosphonate, 2,4-dimethylphenol (12.4 µL, 0.11 mmol) used as nucleophile and BIMP **B1** - P(*p*-tol)$_3$ (11.7 mg, 0.015 mmol) as catalyst in PhF (0.40 mL) at 23 °C. Pentane:EtOAc 7:3. Yellow oil (13.0 mg, 0.022 mmol, 22% yield, 86% e.e.).

HPLC Conditions: CHIRALPAK IA, hexane/isopropanol = 85/15, 1 mL/min, λ = 220 nm, t (major) = 9.32 min, t (minor) = 15.98 min

$[\alpha]_D^{25}$ = +4.0 (c = 0.35, $CHCl_3$) **^{1}H NMR** (400 MHz, $CDCl_3$) δ 7.76 (dd, *J* = 8.1, 1.7 Hz, 1H), 7.72 – 7.59 (m, 4H), 7.49 – 7.41 (m, 3H), 7.41 – 7.34 (m, 4H), 7.21 (ddd, *J* = 8.5, 7.7, 1.2 Hz, 1H), 7.16 – 7.10 (m, 1H), 6.98 (d, *J* = 1.9 Hz, 1H), 6.90 – 6.83 (m, 1H), 4.39 – 4.25 (m, 2H), 2.46 – 2.41 (m, 3H), 2.27 (s, 3H), 2.19 (s, 3H), 1.04 (s, 9H).; **^{13}C NMR** (151 MHz, $CDCl_3$) δ 147.1 (d, *J* = 8.1 Hz), 143.2 (d, *J* = 3.7 Hz), 141.3 (d, *J* = 10.9 Hz), 136.2, 135.82(d, *J* = 10.6 Hz), 134.8 (d, *J* = 27.5 Hz), 134.3 (d, *J* = 3.3 Hz), 132.2, 132.0 (d, *J* = 4.9 Hz), 131.7, 130.2 (d, *J* = 5.5 Hz), 128.9 (d, *J* = 6.0 Hz), 128.0, 127.5 (d, *J* = 4.3 Hz), 125.3, 123.6, 119.7 (d, *J* = 2.5 Hz), 59.1 (d, *J* = 173.1 Hz), 26.7, 20.8, 19.4, 17.3, 16.4.; **^{31}P NMR** (162 MHz, $CDCl_3$) δ 17.32.; **IR** (film) υ_{max}/cm^{-1}:2981, 2889, 1537, 1472, 1382, 1252, 1152, 1115, 954, 821, 742, 703; **HRMS** (ESI+): calcd. for $C_{34}H_{35}NO_6PSi$ 612.1966 $[M+H]^+$, found 612.1942.

General Procedure IV: Second Nucleophilic Displacement

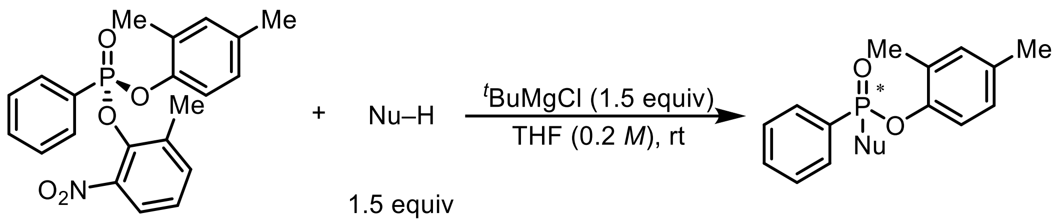

To a solution of nucleophile in THF was added *t*-BuMgCl under argon at 0 °C. After 20 min, phosphonate was added, and the reaction mixture was stirred at rt for the time specified in the individual experiment. The resulting mixture was loaded directly onto silica gel and purified by flash column chromatography as specified in the individual experiment to afford pure product. The two enantiomers were separated by chiral HPLC using conditions specified in the individual experiment.

Products: Derivatisation

Benzyl (2,4-dimethylphenyl) (*R*)-phenylphosphonate (**25**)

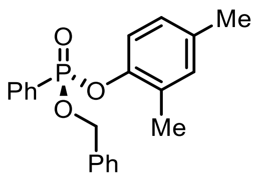

25 was synthesised following **GP IV**. **1** (39.7 mg, 0.100 mmol, 92% e.e.) was used as phosphonate, benzyl alcohol (16.0 µL, 0.150 mmol) used as nucleophile and *t*-BuMgCl (2 *M* in Et_2O, 75.0 µL, 0.150 mmol) as base in THF (0.500 mL) at 23 °C for 2 h. Pentane:EtOAc 7:3. Colourless oil (24.2 mg, 0.063 mmol, 63% yield, 92% e.e.).

HPLC Conditions: CHIRALPAK AD-H, hexane/isopropanol = 70/30, 1 mL/min, λ = 220 nm, t (major) = 11.04 min, t (minor) = 17.81 min.

$[\alpha]_D^{25}$ = –8.0 (c = 1.4, $CHCl_3$); **^{1}H NMR** (400 MHz, $CDCl_3$) δ 7.89 – 7.77 (m, 2H), 7.56 – 7.46 (m, 1H), 7.49 – 7.35 (m, 2H), 7.35 – 7.18 (m, 2H), 7.05 (dd, *J* = 8.3, 1.4 Hz, 1H), 6.92 – 6.86 (m, 1H), 6.82 (dd, *J* = 8.3, 2.2 Hz, 1H), 5.19 – 5.04 (m, 2H), 2.20 (s, 3H), 2.13 (s, 3H); **^{13}C NMR** (101 MHz, $CDCl_3$) δ 146.9 (d, *J* = 8.0 Hz), 136.0 (d, *J* = 7.1 Hz), 134.3, 132.7 (d, *J* = 3.1 Hz), 131.9 (d, *J* = 10.3 Hz), 131.9, 129.1 (d, *J* = 5.3 Hz), 128.6, 128.5, 128.34 (d, *J* = 8.9 Hz), 128.0 (d, *J* = 192 Hz), 127.9, 120.2 (d, *J* = 2.5 Hz), 68.1 (d, *J* = 5.6 Hz), 20.7, 16.5; **^{31}P NMR** (162 MHz, $CDCl_3$) δ 15.76; **IR** (film) υ_{max}/cm^{-1}:1497, 1439, 1256, 1201, 1130, 1009, 947, 907; **HRMS** (ESI+): calcd. for $C_{21}H_{21}NaO_3P$ 375.1126 $[M+Na]^+$, found 375.1121.

((2*R*,3*S*,5*R*)-5-(2-Amino-6-oxo-1,6-dihydro-9H-purin-9-yl)-3-((*tert*-butyldimethylsilyl)oxy)tetrahydrofuran-2-yl)methyl (2,4-dimethylphenyl) (*R*)-phenylphosphonate (**26**)

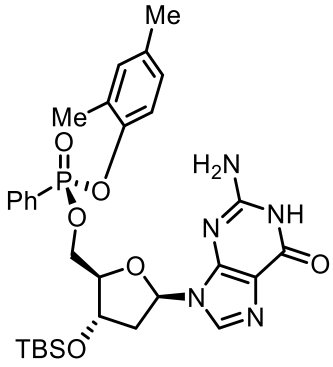

26 was synthesised following **GP IV**. **1** (39.7 mg, 0.100 mmol, 92% e.e.) was used as phosphonate, 3'-OTBS -deoxyguanosine[19] (57.2 mg, 0.150 mmol) used as nucleophile and *t*-BuMgCl (2 *M* in Et_2O, 150 µL, 0.300 mmol) as base in THF (0.500 mL) at 23 °C for 16 h. CH_2Cl_2:MeOH 9:1. Colourless oil (45.0 mg, 0.072 mmol, 72% yield, >95:5 dr).

[α]$_D^{25}$ = +34.9 (c = 0.45, DMSO); **^{1}H NMR** (500 MHz, DMSO-*d*$_6$) δ 10.66 (s, 1H), 7.83 (s, 1H), 7.79 (ddd, *J* = 13.6, 8.1, 1.4 Hz, 2H), 7.68 (td, *J* = 7.4, 1.4 Hz, 1H), 7.55 (td, *J* = 7.6, 4.4 Hz, 2H), 7.01 (d, *J* = 2.1 Hz, 1H), 6.99 (dd, *J* = 8.3, 1.2 Hz, 1H), 6.87 (dd, *J* = 8.4, 2.2 Hz, 1H), 6.48 (s, 2H), 6.12 (dd, *J* = 7.6, 6.3 Hz, 1H), 4.52 (dt, *J* = 6.1, 3.2 Hz, 1H), 4.29 (ddd, *J* = 11.6, 6.9, 4.9 Hz, 1H), 4.17 (ddd, *J* = 11.5, 6.7, 5.4 Hz, 1H), 3.98 (td, *J* = 5.1, 2.9 Hz, 1H), 2.63 (ddd, *J* = 13.4, 7.7, 5.9 Hz, 1H), 2.23 (ddd, *J* = 13.5, 6.4, 3.5 Hz, 1H), 2.19 (s, 3H), 2.11 (s, 3H), 0.84 (s, 9H), 0.05 (s, 3H), 0.03 (s, 3H); **^{13}C NMR** (126 MHz, DMSO-*d*$_6$) δ 156.7, 153.7, 150.9, 146.4 (d, *J* = 7.6 Hz), 135.2 134.0, 133.3 (d, *J* = 3.1 Hz), 131.9, 131.5 (d, *J* = 10.2 Hz), 128.9 (d, *J* = 15.2 Hz), 128.6 (d, *J* = 5.3 Hz), 127.3, 127.0(d, *J* = 189.6 Hz), 119.6 (d, *J* = 2.7 Hz), 116.7, 84.9 (d, *J* = 7.2 Hz), 82.5, 72.1, 65.5 (d, *J* = 5.7 Hz), 38.8, 25.6, 20.2, 17.6, 16.1, -4.9, -5.1; **^{31}P NMR** (202 MHz, DMSO-*d*$_6$) δ 15.57; **IR** (film) $υ_{max}$/cm^{-1}: 2863, 1592, 1498, 1423, 1267, 1213, 1128, 1112, 1058, 1022, 962, 913, 848, 744; **HRMS** (ESI+): calcd. for $C_{30}H_{41}N_5O_6PSi$ 626.2558 $[M+H]^+$, found 626.2551.

---

[19] E. Defrancq; N.Pelloux; A. Leterme; M-F. Lhomme; J. Lhomme *J. Org. Chem.* **1991**, 56, 4817-4819.

((2*R*,3*S*,5*R*)-5-(4-Amino-2-oxopyrimidin-1(2H)-yl)-3-((tert-butyldimethylsilyl)oxy)tetrahydrofuran-2-yl)methyl (2,4-dimethylphenyl) (*R*)-phenylphosphonate (**27**)

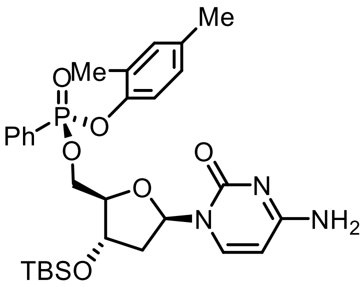

27 was synthesised following **GP IV**. **1** (39.7 mg, 0.10 mmol, 92% e.e.) was used as phosphonate, 3'-OTBS –deoxycytidine[20] (68.3 mg, 0.20 mmol) used as nucleophile and *t*-BuMgCl (2 *M* in Et_2O, 200 µL, 0.40 mmol) as base in THF (0.50 mL) at 23 °C for 48 h. CH_2Cl_2:MeOH 95:5. Colourless oil (28.1 mg, 0.048 mmol, 48% yield, >95:5 dr).

$[\alpha]_D^{25}$ = +57.0 (c = 0.45, $CHCl_3$); **^{1}H NMR** (400 MHz, $CDCl_3$) δ 7.87 (ddt, *J* = 13.8, 6.9, 1.4 Hz, 2H), 7.64 – 7.57 (m, 1H), 7.54 – 7.46 (m, 3H), 7.08 (dd, *J* = 8.3, 1.4 Hz, 1H), 6.96 – 6.94 (m, 1H), 6.86 (dd, *J* = 8.2, 2.3 Hz, 1H), 6.20 (t, *J* = 6.3 Hz, 1H), 5.58 (d, *J* = 7.5 Hz, 1H), 4.42 – 4.19 (m, 2H), 4.02 (dd, *J* = 3.8, 1.9 Hz, 1H), 2.34 (ddd, *J* = 13.5, 6.2, 4.1 Hz, 1H), 2.23 (s, 3H), 2.20 (s, 3H), 1.91 (dt, *J* = 13.3, 6.5 Hz, 1H), 0.84 (s, 9H), 0.02 (s, 3H), 0.00 (s, 3H); **^{13}C NMR** (101 MHz, $CDCl_3$) δ 165.4, 155.6, 146.8 (d, *J* = 7.5 Hz), 140.7, 134.8, 133.4 (d, *J* = 3.1 Hz), 132.2, 131.9(d, *J* = 10.1 Hz), 129.0 (d, *J* = 15.7 Hz), 129.0 (d, *J* = 5.7 Hz), 127.6, 127.3 (d, *J* = 191.8 Hz), 120.0 (d, *J* = 2.5 Hz), 94.6, 86.4, 85.4 (d, *J* = 7.9 Hz), 71.5, 65.3, 41.8, 25.8, 20.8, 18.0, 16.7, -4.6, -4.9; **^{31}P NMR** (162 MHz, $CDCl_3$) δ 15.97 (major), 15.91 (minor); **IR** (film) υ_{max}/cm^{-1}: 2928, 1648, 1496, 1251, 1200, 1118, 1024, 948, 909, 935, 781, 696; **HRMS** (ESI+): calcd. for $C_{29}H_{41}N_3O_6PSi$ 586.2497 $[M+H]^+$, found 586.2491.

---

[20] X-F. Zhu; H. J. Williams; A.I Scott *J. Chem. Soc. Perkin 1* **2000**, 2305-2306.

((2*R*,3*S*,5*R*)-5-(6-Amino-9H-purin-9-yl)-3-((tert-butyldimethylsilyl)oxy)tetrahydrofuran-2-yl)methyl (2,4-dimethylphenyl) (*R*)-phenylphosphonate (**28**)

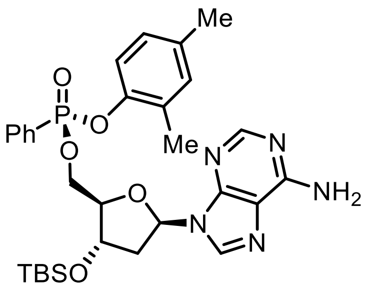

28 was synthesised following **GP IV**. **1** (39.7 mg, 0.100 mmol, 92% e.e.) was used as phosphonate, 3'-OTBS -deoxyadenosine[21] (54.8 mg, 0.150 mmol) used as nucleophile and *t*-BuMgCl (2 *M* in Et_2O, 150 µL, 0.300 mmol) as base in THF (0.500 mL) at 23 °C for 16 h. CH_2Cl_2:MeOH 95:5. Colourless oil (29.2 mg, 0.048 mmol, 49% yield, >95:5 dr).

$[\alpha]_D^{25}$ = –14.9 (c = 0.18, $CHCl_3$); **^{1}H NMR** (500 MHz, $CDCl_3$) δ 8.38 (s, 1H), 8.22 – 8.12 (m, 2H), 7.95 (s, 1H), 7.80 (s, 1H), 7.58 – 7.52 (m, 1H), 7.50 – 7.43 (m, 2H), 7.21 (dd, *J* = 8.2, 1.3 Hz, 1H), 6.97 (d, *J* = 2.0 Hz, 1H), 6.84 (dd, *J* = 8.3, 2.3 Hz, 1H), 6.23 (dd, *J* = 9.6, 5.4 Hz, 1H), 6.00 (s, 1H), 4.66 (d, *J* = 4.8 Hz, 1H), 3.98 – 3.87 (m, 1H), 3.76 – 3.66 (m, 2H), 2.92 (ddd, *J* = 13.0, 9.6, 5.0 Hz, 1H), 2.35 (s, 3H), 2.24 (s, 3H), 2.22 (s, 1H), 2.17 (dd, *J* = 13.0, 5.5 Hz, 1H), 0.92 (s, 9H), 0.10 (s, 6H); **^{13}C NMR** (126 MHz, $CDCl_3$) δ 152.1, 151.6, 149.5, 146.8 (d, *J* = 8.6 Hz), 142.3, 134.6, 132.9 (d, *J* = 3.0 Hz), 132.6 (d, *J* = 11.0 Hz), 132.3, 129.5 (d, *J* = 5.8 Hz), 129.3 (d, *J* = 182.3 Hz), 128.4 (d, *J* = 15.8 Hz), 127.5, 120.0 (d, *J* = 3.3 Hz), 90.6, 88.1, 74.2, 63.5, 58.6, 41.5, 25.9, 20.8, 18.2, 16.9, -4.6, -4.6; **^{31}P NMR** (202 MHz, $CDCl_3$) δ 12.79; **IR** (film) υ_{max}/cm^{-1}: 2927, 2856, 1606, 1584, 1462, 1439, 1252, 1220, 1123, 1101, 1063, 1025, 950, 909, 835, 734; **HRMS** (ESI+): calcd. for $C_{30}H_{41}N_5O_5PSi$ 610.2609 $[M+H]^+$, found 610.2608.

[21] R. V. Somu; D. J. Wilson; E. M. Bennett; H. I. Boshoff; L. Celia; B. J. Beck; C. E. Barry, III; C. C. Aldrich *J. Med. Chem.* **2006**, 49, 26, 7623–7635.

((2*R*,3*S*,5*R*)-3-((*tert*-Butyldimethylsilyl)oxy)-5-(5-methyl-2,4-dioxo-3,4-dihydropyrimidin-1(2H)-yl)tetrahydrofuran-2-yl)methyl (2,4-dimethylphenyl) (*R*)-phenylphosphonate (**29**)

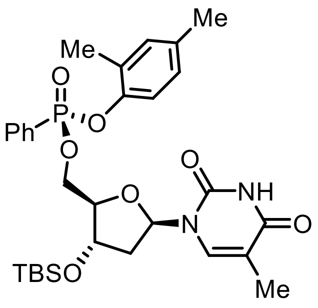

29 was synthesised following **GP IV**. **1** (39.7 mg, 0.100 mmol, 92% e.e.) was used as phosphonate, 3'-OTBS- thymidine[22] (53.5 mg, 0.150 mmol) used as nucleophile and *t*-BuMgCl (2 *M* in Et_2O, 150 µL, 0.300 mmol) as base in THF (0.500 mL) at 23 °C for 48 h. CH_2Cl_2:MeOH 95:5. Colourless oil (42.0 mg, 0.07 mmol, 70% yield, >95:5 dr).

$[\alpha]_D^{25}$ = +23.4 (c = 0.86, $CHCl_3$); **^{1}H NMR** (400 MHz, $CDCl_3$) δ 8.63 (s, 1H), 7.88 (ddt, *J* = 13.8, 6.9, 1.4 Hz, 2H), 7.66 – 7.60 (m, 1H), 7.53 – 7.47 (m, 2H), 7.34 (q, *J* = 1.2 Hz, 1H), 7.07 (dd, *J* = 8.3, 1.5 Hz, 1H), 6.99 – 6.92 (m, 1H), 6.86 (dd, *J* = 8.3, 2.3 Hz, 1H), 6.30 (dd, *J* = 7.9, 5.8 Hz, 1H), 4.42 (dt, *J* = 5.9, 2.8 Hz, 1H), 4.34 – 4.30 (m, 3H), 4.06 (td, *J* = 3.0, 2.0 Hz, 1H), 2.28 – 2.15 (m, 7H), 1.94 (ddd, *J* = 13.7, 7.9, 6.2 Hz, 1H), 1.72 (d, *J* = 1.2 Hz, 3H), 0.86 (s, 9H), 0.05 (s, 2H), 0.04 (s, 2H). **^{13}C NMR** (101 MHz, $CDCl_3$) δ 163.8, 150.4, 146.8 (d, *J* = 7.5 Hz), 135.3, 134.8, 133.4 (d, *J* = 3.1 Hz), 132.2, 131.9 (d, *J* = 10.4 Hz), 129.0, 128.9, 127.6, 127.2 (d, *J* = 190.9 Hz), 119.9 (d, *J* = 2.5 Hz), 111.3, 85.7 (d, *J* = 7.8 Hz), 85.1, 72.1, 65.6 (d, *J* = 5.6 Hz), 41.0, 25.7, 20.8, 18.0, 16.7, 12.4, -4.6, -4.8; **^{31}P NMR** (162 MHz, $CDCl_3$) δ 16.04; **IR** (film) υ_{max}/cm^{-1}: 2928, 2856, 1688, 1498, 1470, 1440, 1251, 1149, 1131, 1117, 1086, 1033, 950, 910, 834, 780, 754, 696; **HRMS** (ESI+): calcd. for $C_{30}H_{42}N_2O_7PSi$ 601.2493 $[M+H]^+$, found 601.2490.

[22] X-F. Zhu; H. J. Williams; A.I Scott *J. Chem. Soc. Perkin 1* **2000**, 2305-2306.

((2*R*,5*R*)-3-((tert-butyldimethylsilyl)oxy)-5-(2,4-dioxo-3,4-dihydropyrimidin-1(2H)-yl)-4-fluoro-4-methyltetrahydrofuran-2-yl)methyl (2,4-dimethylphenyl) (*R*)-phenylphosphonate (**30**)

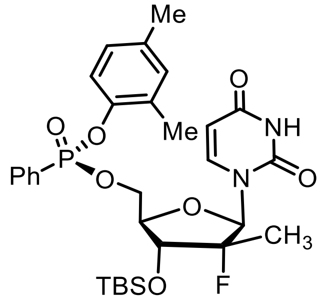

30 was synthesised following **GP IV**. **1** (39.7 mg, 0.100 mmol, 92% e.e.) was used as phosphonate, 3’-OTBS-2’-deoxy-2’-α-fluoro-2’-β-C-methyluridine[23] (56.2 mg, 0.150 mmol) used as nucleophile and *t*-BuMgCl (2 *M* in Et_2O, 150 µL, 0.300 mmol) as base in THF (0.500 mL) at 23 °C for 2 h. CH_2Cl_2:MeOH 19:1. Pale yellow oil (22.1 mg, 0.035 mmol, 35% yield, 94:6 dr).

$[\alpha]_D^{25}$ = +73.7° (c = 1.0, $CHCl_3$); **^{1}H NMR** (400 MHz, $CDCl_3$) δ 8.96 (s, 1H), 7.95 – 7.81 (m, 2H), 7.70 – 7.61 (m, 1H), 7.58 – 7.51 (m, 2H), 7.43 (d, *J* = 8.2 Hz, 1H), 7.03 (dd, *J* = 8.3, 1.4 Hz, 1H), 7.00 – 6.95 (m, 1H), 6.85 (dd, *J* = 8.3, 2.3 Hz, 1H), 6.16 (d, *J* = 18.8 Hz, 1H), 5.18 (d, *J* = 8.2 Hz, 1H), 4.70 (ddd, *J* = 11.9, 5.5, 2.0 Hz, 1H), 4.31 (ddd, *J* = 11.8, 3.7, 2.0 Hz, 1H), 4.14 (ddt, *J* = 8.3, 3.4, 1.7 Hz, 1H), 3.94 (dd, *J* = 21.2, 9.1 Hz, 1H), 2.24 (s, 3H), 2.20 (s, 3H), 1.31 (d, *J* = 21.0 Hz, 3H), 0.91 (s, 8H), 0.14 (s, 3H), 0.14 (s, 3H).; **^{13}C NMR** (151 MHz, $CDCl_3$) δ 162.6, 150.1, 146.6 (d, *J* = 7.1 Hz), 138.7, 134.8, 133.5 (d, *J* = 3.1 Hz), 132.2, 132.0 (d, *J* = 10.3 Hz), 129.0 (d, *J* = 15.4 Hz), 128.7 (d, *J* = 5.5 Hz), 127.5, 126.8 (d, *J* = 190.0 Hz), 119.7 (d, *J* = 2.7 Hz), 102.5, 99.3 (d, *J* = 186.9 Hz), 89.3 (d, *J* = 40.8 Hz), 79.7 (d, *J* = 8.5 Hz), 72.4 (d, *J* = 17.3 Hz), 62.7 (d, *J* = 5.3 Hz), 25.6, 20.6, 18.0, 17.1 (d, *J* = 25.8 Hz), 16.6, -4.2, -4.4.; **^{31}P NMR** (162 MHz, $CDCl_3$) δ 15.92, 15.86.; **^{19}F NMR** (377 MHz, $CDCl_3$) δ -161.36. **IR** (film) υ_{max}/cm^{-1}:2929, 2858, 1697, 1498, 1455, 1380, 1264, 1199, 1157, 1131, 1118, 1098, 1036, 950, 910, 859, 839, 779, 733, 695, 622; **HRMS** (ESI+): calcd. for $C_{30}H_{41}N_2O_7PFSi$ 619.2399 $[M+H]^+$, found 619.2399.

[23] U. Pradere; F. Amblard; S. J. Coats; R. F. Schinazi *Org. Lett.* **2012**, 14, 17, 4426-4429.

2,4-Dimethylphenyl (((3a*R*,4*R*,6*R*,6a*R*)-6-(2,4-dioxo-3,4-dihydropyrimidin-1(2H)-yl)-2,2-dimethyltetrahydrofuro[3,4-d][1,3]dioxol-4-yl)methyl) (*R*)-phenylphosphonate (**31**)

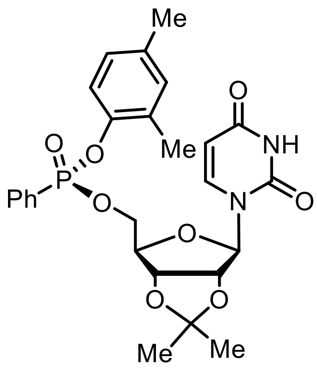

31 was synthesised following **GP IV**. **1** (39.7 mg, 0.100 mmol, 92% e.e.) was used as phosphonate, 2',3'-isopropylideneuridine[24] (31.0 mg, 0.150 mmol) used as nucleophile and *t*-BuMgCl (2 *M* in Et_2O, 150 µL, 0.300 mmol) as base in THF (0.500 mL) at 23 °C for 2 h. CH_2Cl_2:Acetone 10:1 to 5:1. Colourless oil (37.7 mg, 0.071 mmol, 71% yield, >95:5 dr).

$[\alpha]_D^{25}$ = +4.5 (c = 1.27, $CHCl_3$); **^{1}H NMR** (400 MHz, $CDCl_3$) δ 9.36 (s, 1H), 7.87 (dd, *J* = 13.8, 6.8 Hz, 2H), 7.65 – 7.55 (m, 1H), 7.54 – 7.44 (m, 2H), 7.23 (d, *J* = 8.1 Hz, 1H), 7.10 (d, *J* = 8.3 Hz, 1H), 6.95 (s, 1H), 6.87 (d, *J* = 8.3 Hz, 1H), 5.78 (d, *J* = 2.8 Hz, 1H), 5.51 (d, *J* = 8.1 Hz, 1H), 4.79 (dd, *J* = 6.5, 2.3 Hz, 1H), 4.67 (dd, *J* = 6.5, 2.8 Hz, 1H), 4.37 (m, 3H), 2.23 (s, 3H), 2.19 (s, 3H), 1.55 (s, 3H), 1.32 (s, 3H).;**^{13}C NMR** (101 MHz, $CDCl_3$) 163.3, 150.0, 146.7 (d, *J* = 7.9 Hz), 140.9, 134.7, 133.3 (d, *J* = 3.1 Hz), 132.1, 131.9 (d, *J* = 10.3 Hz), 128.8 (d, *J* = 5.7 Hz), 128.8 (d, *J* = 15.5 Hz), 127.5, 127.0 (d, *J* = 192 Hz), 119.8 (d, *J* = 2.7 Hz), 114.6, 102.5, 93.0, 84.9 (d, *J* = 7.2 Hz), 84.4, 80.5, 65.9 (d, *J* = 4.6 Hz), 27.1, 25.2, 20.7, 16.6; **^{31}P NMR** (162 MHz, $CDCl_3$) δ 16.09 (minor), 15.94 (major); **IR** (film) υ_{max}/cm^{-1}:2897, 1693, 1498, 1456, 1381, 1253, 1201, 1118, 1068, 949, 909, 754, 696; **HRMS** (ESI+): calcd. for $C_{26}H_{30}N_2O_8P$ 529.1740 $[M+H]^+$, found 529.1733.

[24] A. E. Ragab; S. Grüschow; D. R. Tromans; R. J. M. Goss *J. Am. Chem. Soc.* **2011**, 133, 39, 15288–15291.

((3a*R*,4*R*,6*R*,6a*R*)-6-(6-Amino-9H-purin-9-yl)-2,2-dimethyltetrahydrofuro[3,4-d][1,3]dioxol-4-yl)methyl (2,4-dimethylphenyl) (*R*)-phenylphosphonate (**32**)

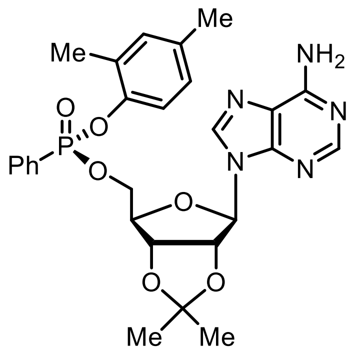

32 was synthesised following **GP IV**. **1** (39.7 mg, 0.100 mmol, 92% e.e.) was used as phosphonate, 2',3'-isopropylideneadenosine[25] (45.0 mg, 0.150 mmol) used as nucleophile and *t*-BuMgCl (2 *M* in Et_2O, 150 µL, 0.300 mmol) as base in THF (0.500 mL) at 23 °C for 16 h. CH_2Cl_2:MeOH 95:5. Colourless oil (26.4 mg, 0.048 mmol, 48% yield, >95:5 dr).

$[\alpha]_D^{25}$ = –53.6 (c = 1.00, $CHCl_3$); **^{1}H NMR** (500 MHz, $CDCl_3$) δ 8.41 (d, *J* = 12.3 Hz, 2H), 8.18 – 8.06 (m, 3H), 7.58 – 7.49 (m, 1H), 7.46 (dtd, *J* = 8.8, 4.5, 2.6 Hz, 2H), 7.19 (dd, *J* = 8.4, 1.4 Hz, 1H), 7.00 – 6.86 (m, 1H), 6.81 (dd, *J* = 8.3, 2.3 Hz, 1H), 5.90 (d, *J* = 4.4 Hz, 1H), 5.85 – 5.72 (m, 1H), 5.09 (dd, *J* = 5.9, 4.4 Hz, 1H), 5.04 (dd, *J* = 5.8, 1.3 Hz, 1H), 4.51 (q, *J* = 1.7 Hz, 1H), 3.90 (dt, *J* = 12.6, 2.0 Hz, 1H), 3.75 (ddd, *J* = 12.4, 10.0, 2.1 Hz, 1H), 2.34 (s, 3H), 2.22 (s, 3H), 1.63 (s, 3H), 1.36 (s, 3H); **^{13}C NMR** (126 MHz, $CDCl_3$) δ 152.0, 151.8146.8 (d, *J* = 8.8 Hz), 142.5, 134.5, 132.8 (d, *J* = 3.1 Hz), 132.5 (d, *J* = 11.0 Hz), 129.5 (d, *J* = 6.1 Hz), 129.4 (d, *J* = 183.2 Hz), 128.4 (d, *J* = 15.7 Hz), 127.5, 119.9 (d, *J* = 3.3 Hz), 114.2, 94.1, 83.7, 81.8, 63.3, 27.7, 25.4, 20.8, 16.9; **^{31}P NMR** (162 MHz, $CDCl_3$) δ 12.93; **IR** (film) υ_{max}/cm^{-1}: 2924, 1606, 1586, 1498, 1461, 1440, 1383, 1203, 1154, 1118, 1082, 1036, 951, 911, 706, 694, 695; **HRMS** (ESI+): calcd. for $C_{27}H_{30}N_5O_6P$ 552.2006 $[M+H]^+$, found 552.2007.

[25] F. Ishikawa; H. Kakeya *Bioorganic and Medicinal Chemistry Letters*, **2014**, 24, 865-869.

(3a*R*,5*R*,6*S*,6a*R*)-5-((*R*)-2,2-Dimethyl-1,3-dioxolan-4-yl)-2,2-dimethyltetrahydrofuro[2,3-*d*][1,3]dioxol-6-yl (2,4-dimethylphenyl) phenylphosphonate (**33**)

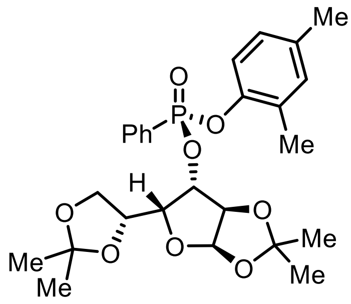

33 was synthesised following **GP IV**. 1 (39.7 mg, 0.100 mmol, 92% e.e.) was used as phosphonate, diacetone D-glucose (39.0 mg, 0.150 mmol) used as nucleophile and *t*-BuMgCl (2 *M* in Et_2O, 75.0 µL, 0.150 mmol) as base in THF (0.500 mL) at 23 °C for 24 h. Pentane:EtOAc 7:3. Colourless oil (48.9 mg, 0.096 mmol, 96% yield, >95:5 dr).

$[\alpha]_D^{25}$ = – 50.8 (c = 0.43, $CHCl_3$); **^{1}H NMR** (400 MHz, $CDCl_3$) δ 7.97 – 7.82 (m, 2H), 7.57 – 7.45 (m, 1H), 7.47 – 7.35 (m, 2H), 7.10 (dd, *J* = 8.2, 1.5 Hz, 1H), 6.93 (t, *J* = 1.5 Hz, 1H), 6.87 (dd, *J* = 8.3, 2.2 Hz, 1H), 5.62 (d, *J* = 3.5 Hz, 1H), 4.77 (dd, *J* = 7.3, 2.6 Hz, 1H), 4.32 (d, *J* = 3.5 Hz, 1H),), 4.08 – 3.97 (m, 2H), 3.94 (dd, *J* = 8.5, 5.9 Hz, 1H), 3.83 (dd, *J* = 8.5, 5.3 Hz, 1H), 2.20 (s, 3H), 2.17 (s, 3H), 1.37 (s, 3H), 1.23 (s, 3H), 1.12 (s, 3H), 1.08 (s, 3H);**^{13}C NMR** (101 MHz, $CDCl_3$) δ 146.5 (d, *J* = 8.7 Hz), 134.9 (d, *J* = 1.3 Hz), 133.0 (d, *J* = 3.2 Hz), 132.2 (d, *J* = 10.3 Hz), 132.0, 129.5 (d, *J* = 5.0 Hz), 128.4 (d, *J* = 15.6 Hz), 127.4, 127.0 (d, *J* = 193.92 Hz), 120.8 (d, *J* = 2.9 Hz), 112.2, 109.2, 105.1, 83.6, 80.7 (d, *J* = 8.5 Hz), 78.9 (d, *J* = 5.0 Hz), 77.2, 72.1, 67.43, 26.7, 26.6, 26.0, 25.0, 20.6, 16.5; **^{31}P NMR** (162 MHz, $CDCl_3$) δ 16.54 (major), 14.36 (minor); **IR** (film) υ_{max}/cm^{-1}:1498, 1440, 1373, 1262, 1201, 1164, 1131, 1075, 1021, 949, 904, 842, 751, 695; **HRMS** (ESI+): calcd. for $C_{26}H_{33}NaO_8P$ 527.1811 $[M+Na]^+$, found 527.1803.

(2*R*,3*S*,5*R*)-2-(((*tert*-Butyldimethylsilyl)oxy)methyl)-5-(5-methyl-2,4-dioxo-3,4-dihydropyrimidin-1(2H)-yl)tetrahydrofuran-3-yl (2,4-dimethylphenyl) (*R*)-phenylphosphonate (**34**)

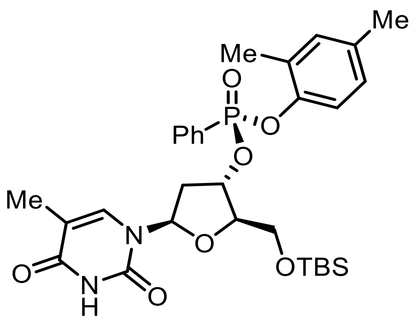

34 was synthesised following **GP IV**. **1** (39.7 mg, 0.100 mmol, 92% e.e.) was used as phosphonate, 5'-OTBS-thymidine[26] (53.5 mg, 0.150 mmol) used as nucleophile and *t*-BuMgCl (2 *M* in Et_2O, 150 µL, 0.300 mmol) as base in THF (0.500 mL) at 23 °C for 16 h. CH_2Cl_2:MeOH 95:5. Colourless oil (42.6 mg, 0.071 mmol, 71% yield, >95:5 dr).

$[\alpha]_D^{25}$ = –5.3 (c = 0.95, $CHCl_3$); **^{1}H NMR** (400 MHz, $CDCl_3$) δ 9.44 (s, 1H), 7.90 (ddt, *J* = 13.9, 6.9, 1.4 Hz, 2H), 7.63 – 7.56 (m, 1H), 7.54 – 7.47 (m, 2H), 7.45 (d, *J* = 1.4 Hz, 1H), 7.13 (dd, *J* = 8.2, 1.5 Hz, 1H), 6.97 (d, *J* = 2.4 Hz, 1H), 6.90 (dd, *J* = 8.3, 2.3 Hz, 1H), 6.37 (dd, *J* = 9.1, 5.2 Hz, 1H), 5.19 – 5.02 (m, 1H), 4.14 (q, *J* = 1.9 Hz, 1H), 3.80 (dd, *J* = 11.5, 2.0 Hz, 1H), 3.69 (dd, *J* = 11.5, 2.2 Hz, 1H), 2.49 – 2.35 (m, 1H), 2.25 (s, 3H), 2.22 (s, 3H), 2.02 (dddd, *J* = 13.7, 9.2, 5.8, 1.5 Hz, 1H), 1.89 (d, *J* = 1.3 Hz, 3H), 0.87 (s, 9H), 0.07 (s, 3H), 0.06 (s, 3H); **^{13}C NMR** (101 MHz, $CDCl_3$) δ 163.9, 150.6, 146.5 (d, *J* = 8.4 Hz), 135.0, 134.8, 133.2 (d, *J* = 3.1 Hz), 132.2, 131.9 (d, *J* = 10.3 Hz), 129.3 (d, *J* = 5.3 Hz), 128.8 (d, *J* = 15.6 Hz), 127.6 (d, *J* = 192.6 Hz), 127.5 (d, *J* = 1.7 Hz), 120.4 (d, *J* = 2.7 Hz), 111.3, 86.3 (d, *J* = 3.6 Hz), 84.6, 77.6 (d, *J* = 5.5 Hz), 63.2, 39.7, 39.6, 26.0, 20.7, 18.4, 16.6, 12.6, -5.4, -5.4; **^{31}P NMR** (162 MHz, $CDCl_3$) δ 15.93; **IR** (film) υ_{max}/cm^{-1}: 2929, 1687, 1497, 1496, 1262, 1198, 1130, 1071, 1009, 974, 950, 908, 832, 781, 733, 697, 625; **HRMS** (ESI+): calcd. for $C_{30}H_{42}N_2O_7PSi$ 601.2493 $[M+H]^+$, found 601.2488.

[26] J. Wu; R. M. Bär; L. Guo; A. Noble; V. K. Aggarwal *Angew. Chem. Int. Ed* **2019**, 58, 18830-18834.

O-(2,4-Dimethylphenyl) S-propyl (*S*)-phenylphosphonothioate (**35**)

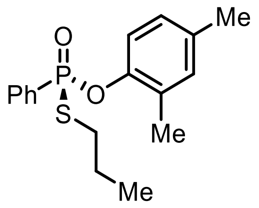

35 was synthesised following **GP IV**. **1** (39.7 mg, 0.100 mmol, 92% e.e.) was used as phosphonate, 1-propanethiol (28.0 µL, 0.300 mmol) used as nucleophile and DBU (45.0 µL, 0.300 mmol) as base in THF (0.500 mL) at 23 °C for 2 h. Pentane:EtOAc 8:2. Colourless oil (19.2 mg, 0.06 mmol, 60% yield, 90% e.e.).

HPLC Conditions: CHIRALPAK AD-H, hexane/isopropanol = 95/5, 1 mL/min, λ = 220 nm, t (major) = 23.18 min, t (minor) = 25.89 min.

$[\alpha]_D^{25}$ = – 41.8 (c = 1.0, $CHCl_3$); **^{1}H NMR** (400 MHz, $CDCl_3$) 7.98 – 7.86 (m, 2H), 7.56 – 7.46 (m, 1H), 7.50 – 7.37 (m, 2H), 7.23 (dd, *J* = 8.2, 1.6 Hz, 1H), 6.97 – 6.91 (m, 1H), 6.91 – 6.82 (m, 1H), 2.69 (dtd, *J* = 12.5, 7.2, 5.2 Hz, 2H), 2.21 (s, 3H), 2.20 (s, 3H), 1.47 (h, *J* = 7.4 Hz, 2H), 0.79 (t, *J* = 7.3 Hz, 3H); **^{13}C NMR** (101 MHz, $CDCl_3$) δ 147.1 (d, *J* = 10.1 Hz), 134.6, 133.0 (d, *J* = 153 Hz), 132.7 (d, *J* = 3.2 Hz), 132.3, 132.0, 131.3 (d, *J* = 10.7 Hz), 129.2 (d, *J* = 5.5 Hz), 128.6 (d, *J* = 15.0 Hz), 127.4, 120.7 (d, *J* = 3.3 Hz), 32.6 (d, *J* = 2.5 Hz), 24.1 (d, *J* = 5.3 Hz), 20.7, 16.8, 13.1; **^{31}P NMR** (162 MHz, $CDCl_3$) δ 42.82; **IR** (film) υ_{max}/cm^{-1}:1495, 1438, 1280, 1195, 1149, 1114, 941, 895, 816, 720, 694; **HRMS** (ESI+): calcd. for $C_{17}H_{22}O_2PS$ 321.1078 $[M+H]^+$, found 321.1073.

<u>*tert*-Butyl (*S*)-benzyl((2,4-dimethylphenoxy)(phenyl)phosphoryl)carbamate (**36**)</u>

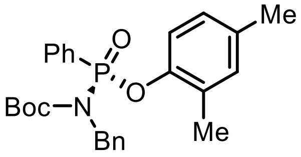

36 was synthesised following **GP IV**. **1** (39.7 mg, 0.100 mmol, 92% e.e.) was used as phosphonate, *tert*-butyl benzylcarbamate (42.6 mg, 0.150 mmol) used as nucleophile and *t*-BuMgCl (2 *M* in Et_2O, 75 µL, 0.15 mmol) as base in THF (0.500 mL) at 23 °C for 2 h. Pentane:EtOAc 8:2. Colourless oil (36.9 mg, 0.084 mmol, 84% yield, 93% e.e.).

HPLC Conditions: CHIRALPAK OD-H, hexane/isopropanol = 99/1, 1 mL/min, λ = 220 nm, t (major) = 23.61, t (minor) = 31.84 min.

$[\alpha]_D^{25}$ = – 47.1 (c = 1.0, $CHCl_3$); **^{1}H NMR** (400 MHz, $CDCl_3$) δ 7.72 – 7.60 (m, 2H), 7.49 – 7.39 (m, 1H), 7.39 – 7.28 (m, 4H), 7.23 – 7.13 (m, 3H), 6.96 – 6.88 (m, 2H), 6.77 (dd, *J* = 8.3, 2.2 Hz, 1H), 4.89 – 4.74 (m, 2H), 2.20 (s, 3H) 2.17 (s, 3H), 1.11 (s, 9H); **^{13}C NMR** (101 MHz, $CDCl_3$) δ 153.5 (d, *J* = 6.4 Hz), 146.5 (d, *J* = 8.8 Hz), 138.6, 134.4, 132.1, 132.0, 131.2 (d, *J* = 10.6 Hz), 131.0 (d, *J* = 193 Hz), 129.4 (d, *J* = 5.5 Hz), 128.9, 128.22, 128.17 (d, *J* = 15.9 Hz), 127.4, 127.3, 120.1 (d, *J* = 3.2 Hz), 83.0, 48.5 (d, *J* = 2.1 Hz), 27.8, 20.7, 16.7; **^{31}P NMR** (162 MHz, $CDCl_3$) δ 16.08; **IR** (film) υ_{max}/cm^{-1}:1717, 1497, 1439, 1368, 1292, 1249, 1199, 1157, 1125, 1092, 1062, 945, 906, 853, 814, 739, 695; **HRMS** (ESI+): calcd. for $C_{26}H_{30}NNaO_4P$ 474.1810 $[M+Na]^+$, found 474.1805.

(*R*)-2-(4-Methoxyphenyl)-4H-benzo[d][1,3,2]dioxaphosphinine 2-oxide (**37**)

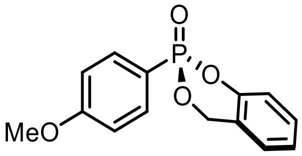

6 (55.6 mg, 0.10 mmol, 91% e.e.) was dissolved in CH_2Cl_2 (900 µL) and TFA (38.3 µL, 57.0 mg, 0.5 mmol) was added dropwise and the reaction was stirred at 23 °C for 10 min. The solution was passed through a short plug of $NaHCO_3$ and $MgSO_4$ and evaporated to dryness. The crude deprotected compound was dissolved in THF (1 mL) and cooled to 0 °C after which *t*-BuMgCl (2 *M* in Et_2O, 75 µL, 0.15 mmol) was added dropwise and the reaction was stirred at 23 °C for 20 min. Upon completion by TLC the reaction solution was loaded directly onto silica gel for silica gel chromatography. Pentane:EtOAc 1:1. Colourless oil (18.5 mg, 0.067 mmol, 67% yield, 84% e.e.).

HPLC Conditions: CHIRALPAK IA, hexane/isopropanol = 80/20, 1 mL/min, λ = 220 nm, t (major) = 17.54 min, t (minor) = 20.01 min.

$[\alpha]_D^{25}$ = – 17.5 (c = 0.82, $CHCl_3$); **^{1}H NMR** (400 MHz, $CDCl_3$) δ 7.72 – 7.52 (m, 2H), 7.26 (tdd, *J* = 8.1, 4.3, 1.9 Hz, 1H), 7.12 – 7.02 (m, 2H), 7.02 – 6.96 (m, 1H), 6.92 – 6.82 (m, 2H), 5.48 (ddd, *J* = 13.8, 9.3, 1.0 Hz, 1H), 5.08 (dd, *J* = 18.7, 13.8 Hz, 1H), 3.77 (s, 3H); **^{13}C NMR** (101 MHz, $CDCl_3$) δ 163.7 (d, *J* = 3.6 Hz), 150.7 (d, *J* = 7.6 Hz), 134.3, 134.1, 130.1, 125.8, 122.9 (d, *J* = 8.7 Hz), 118.8 (d, *J* = 7.2 Hz), 117.5 (d, *J* = 202.3 Hz), 114.4 (d, *J* = 17.1 Hz), 66.4 (d, *J* = 6.8 Hz), 55.5; **^{31}P NMR** (162 MHz, $CDCl_3$) δ 14.01; **IR** (film) υ_{max}/cm^{-1}: 2933, 1598, 1505, 1488, 1459, 1253, 1191, 1130, 1106, 1015, 1987, 911, 826, 797, 758, 729; **HRMS** (ESI+): calcd. for $C_{14}H_{13}O_4P$ 277.0624 $[M+H]^+$, found 277.0624.

NMR Spectra

Compound **B1**

^{1}H NMR (400 MHz, DMSO-d_6):

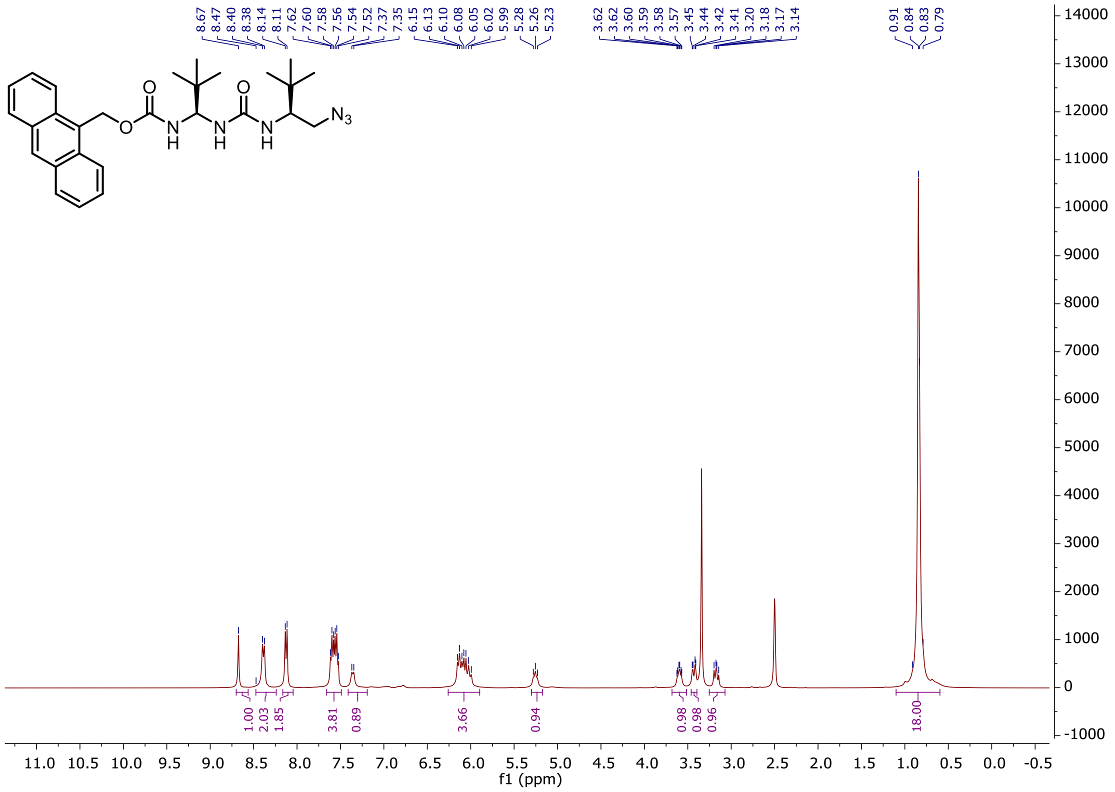

^{13}C NMR (101 MHz, DMSO-d_6):

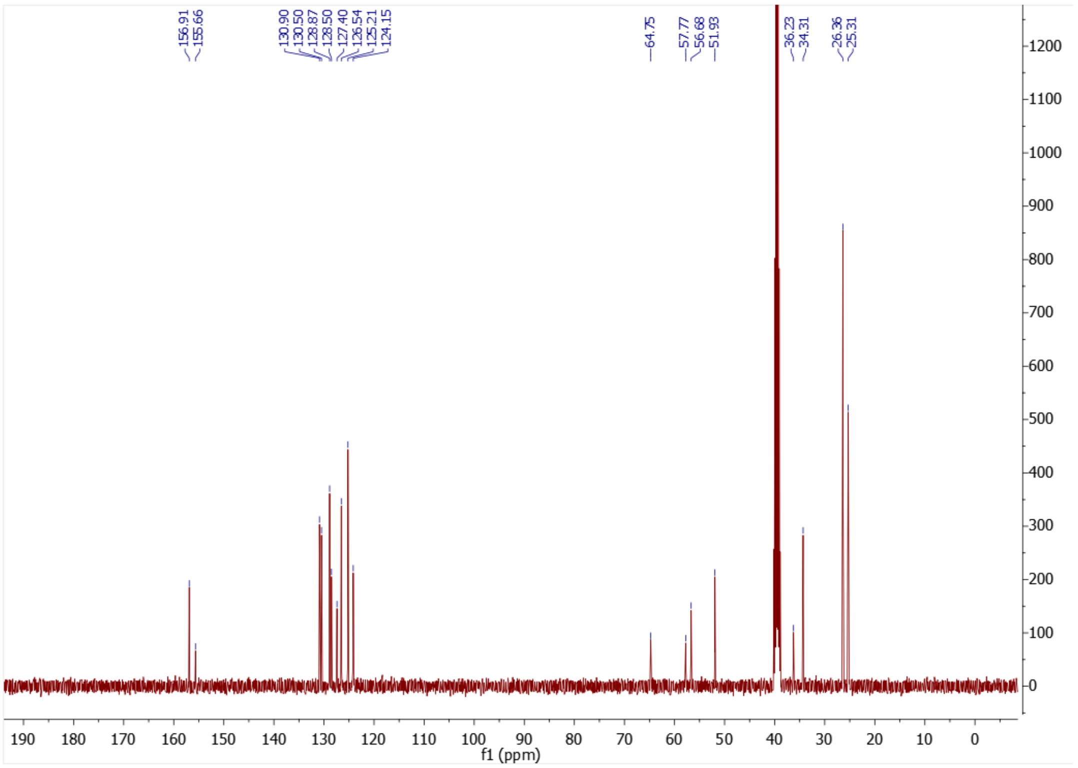

Compound **B12**

^{1}H NMR (400 MHz, DMSO-d_6):

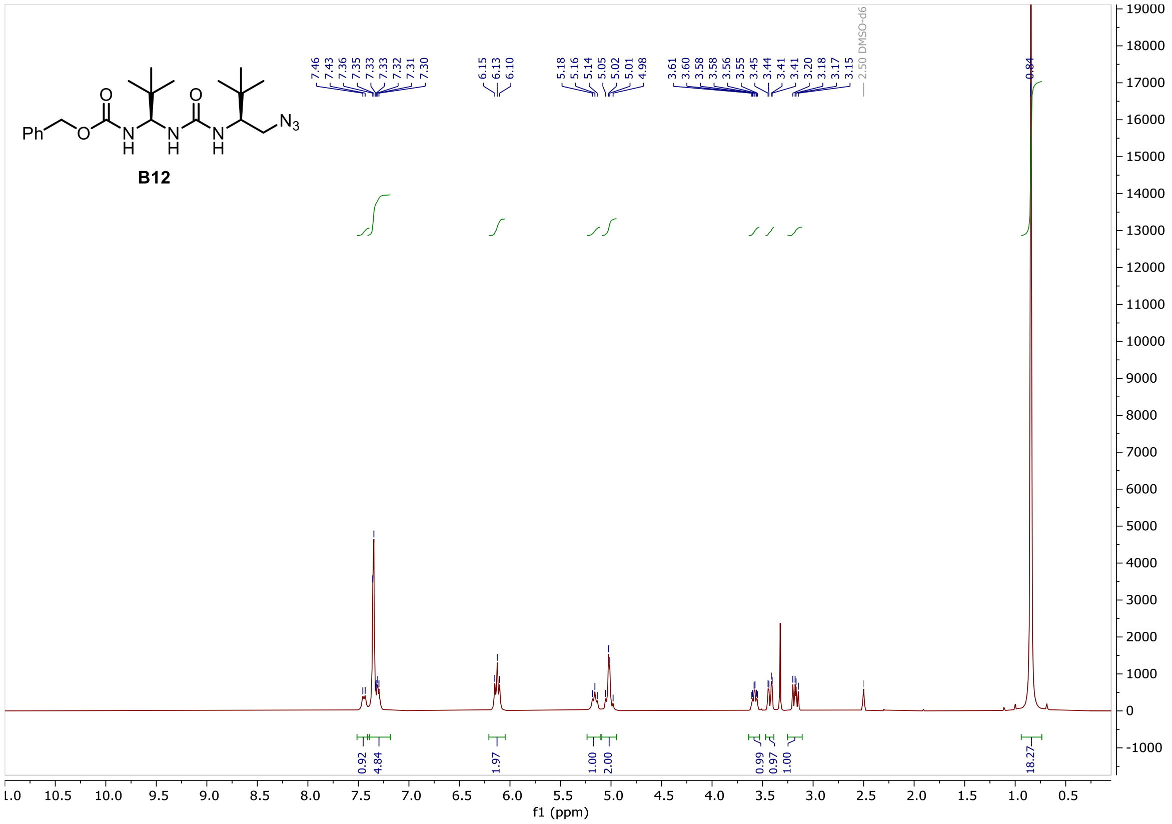

^{13}C NMR (101 MHz, DMSO-d_6):

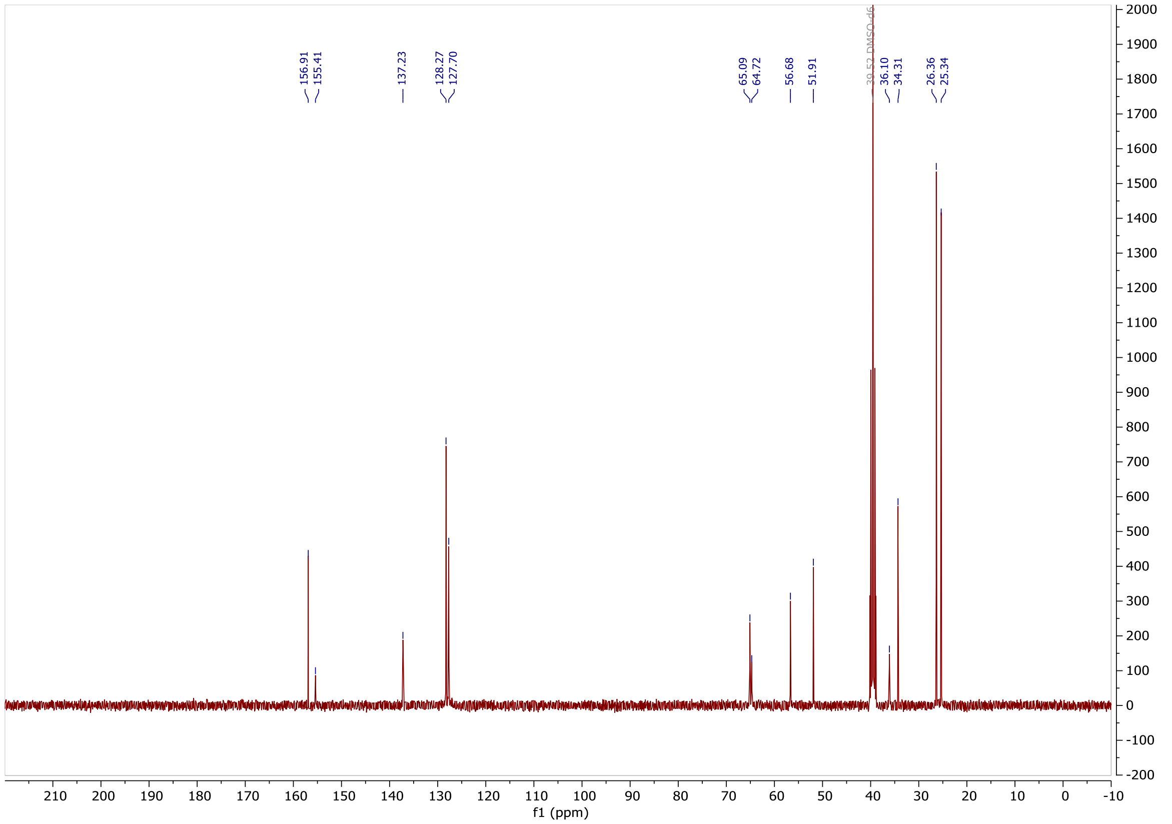

Compound **B13**

^{1}H NMR (400 MHz, DMSO-d_6):

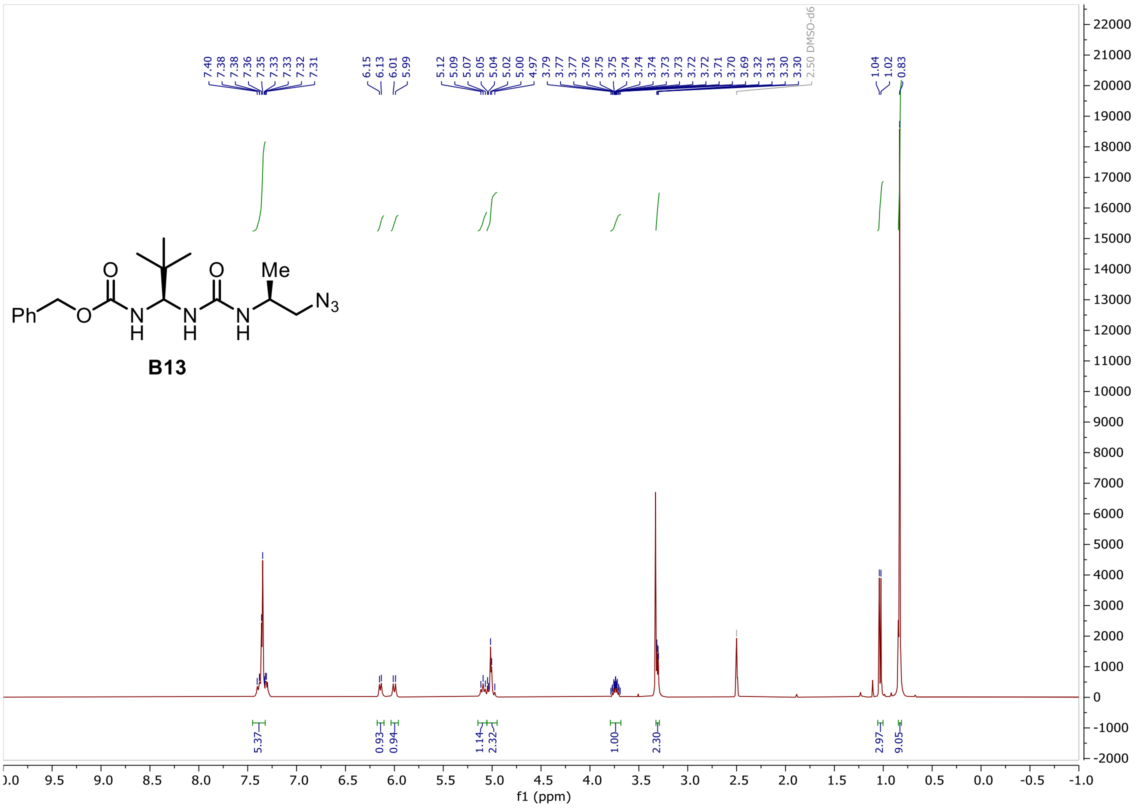

^{13}C NMR (101 MHz, DMSO-d_6):

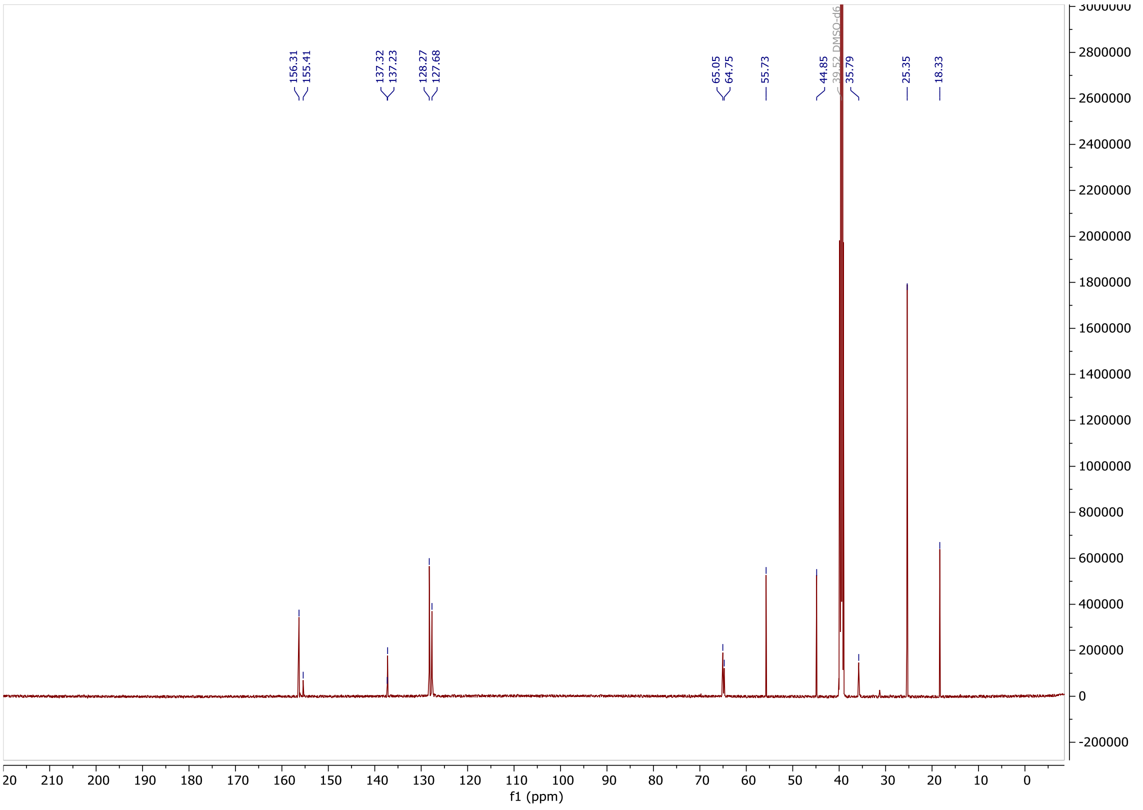

Compound **P-LGS1**

^{1}H NMR (400 MHz, $CDCl_3$):

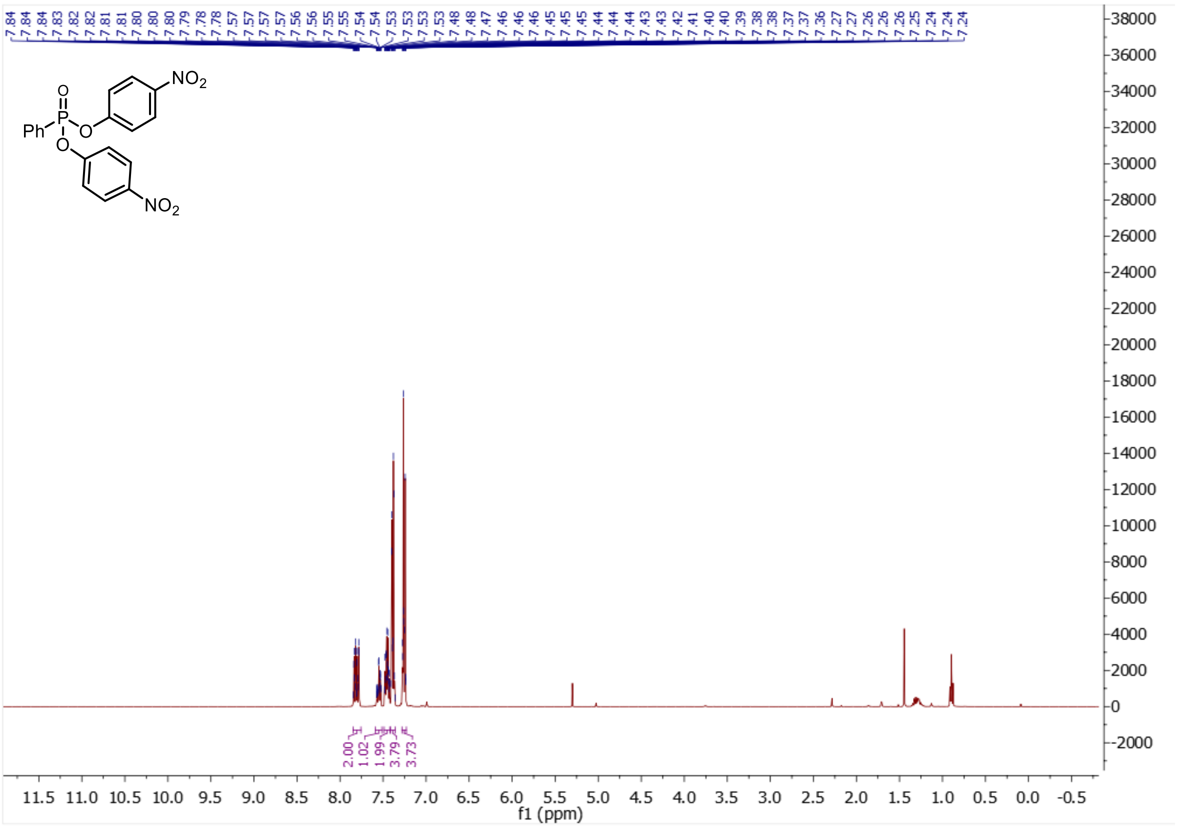

^{13}C NMR (101 MHz, $CDCl_3$):

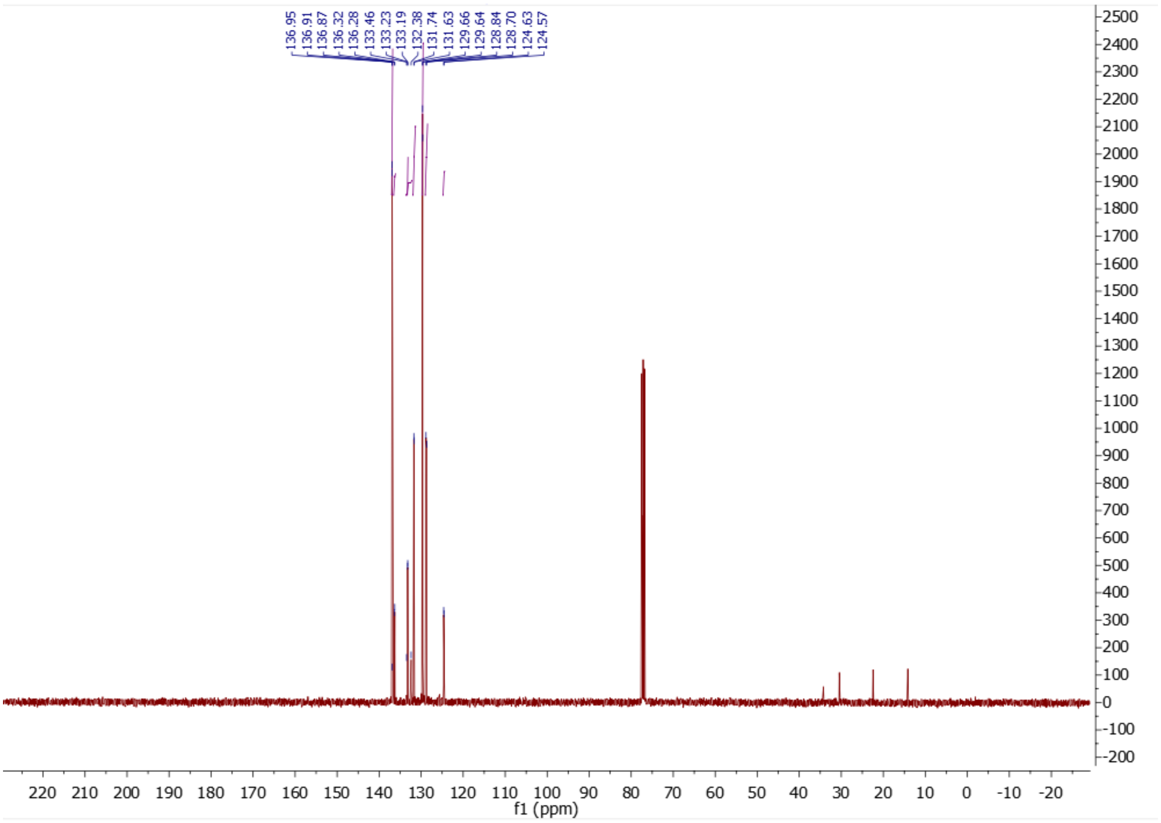

^{31}P NMR (162 MHz, $CDCl_3$):

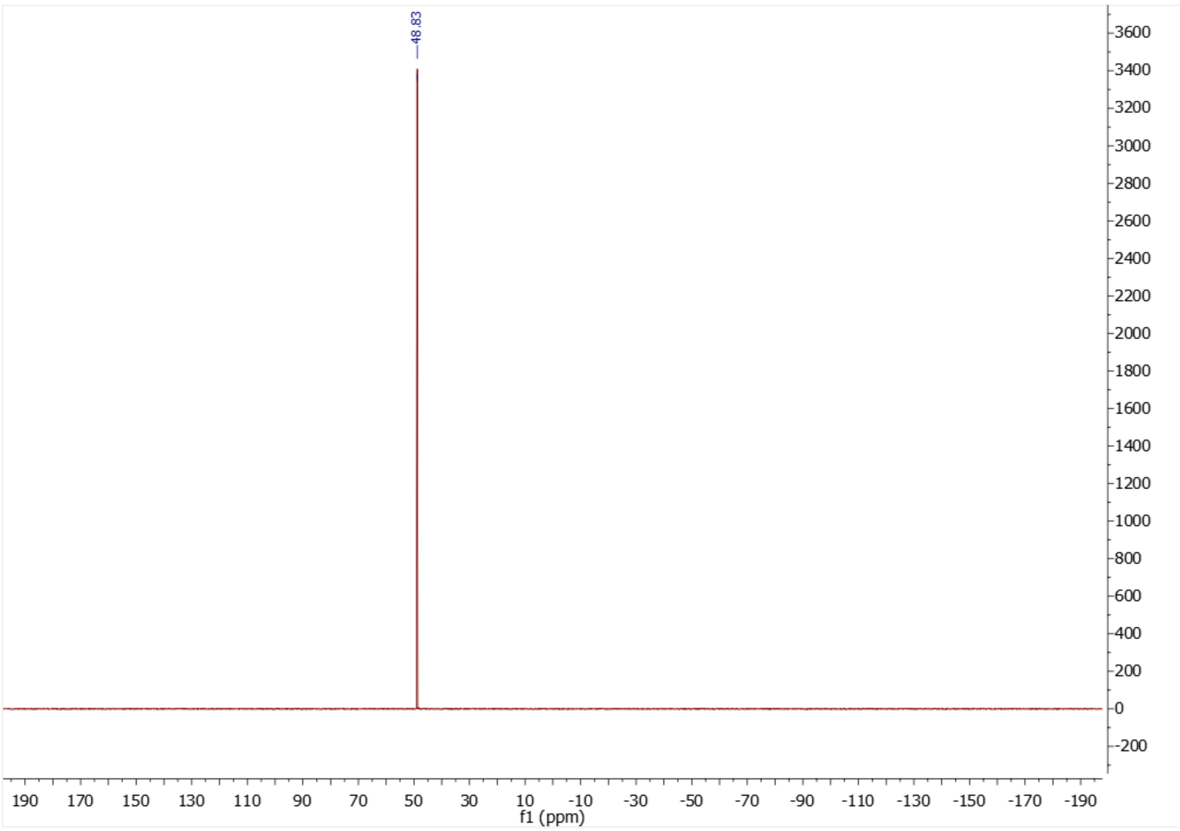

Compound **P-LG1**

^{1}H NMR (400 MHz, $CDCl_3$):

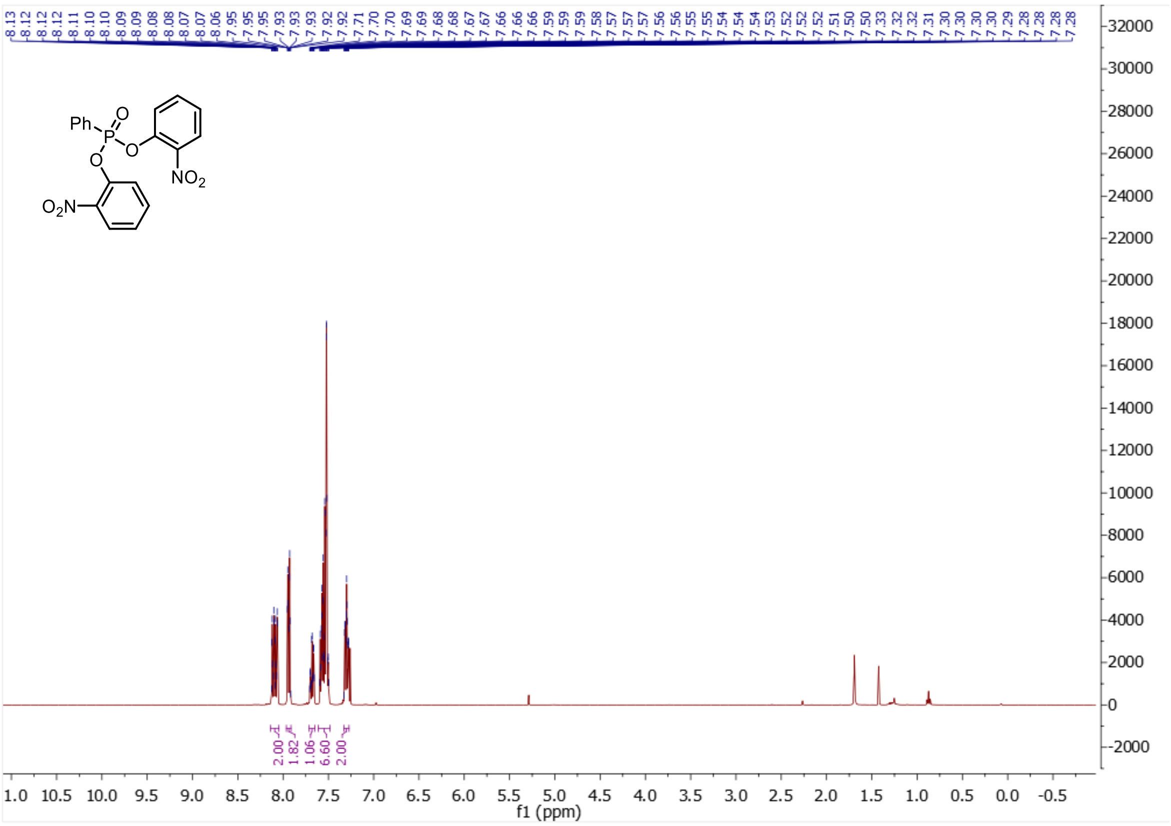

^{13}C NMR (101 MHz, $CDCl_3$):

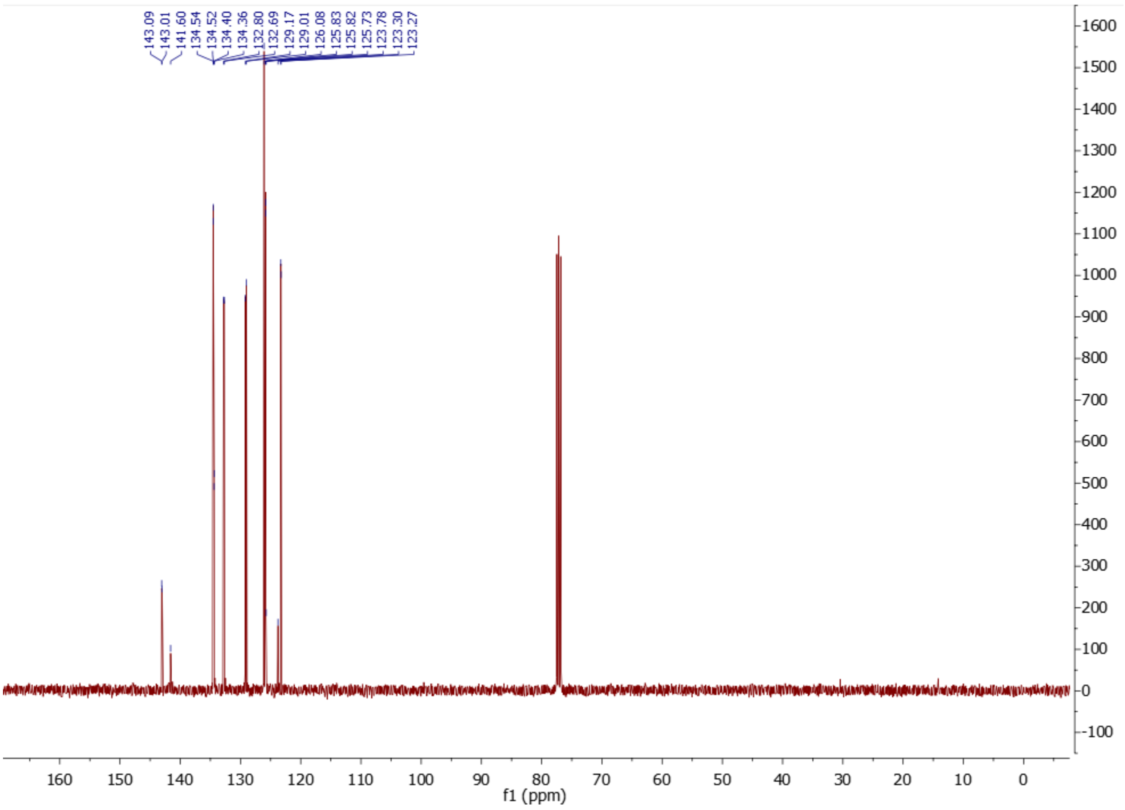

^{31}P NMR (162 MHz, $CDCl_3$):

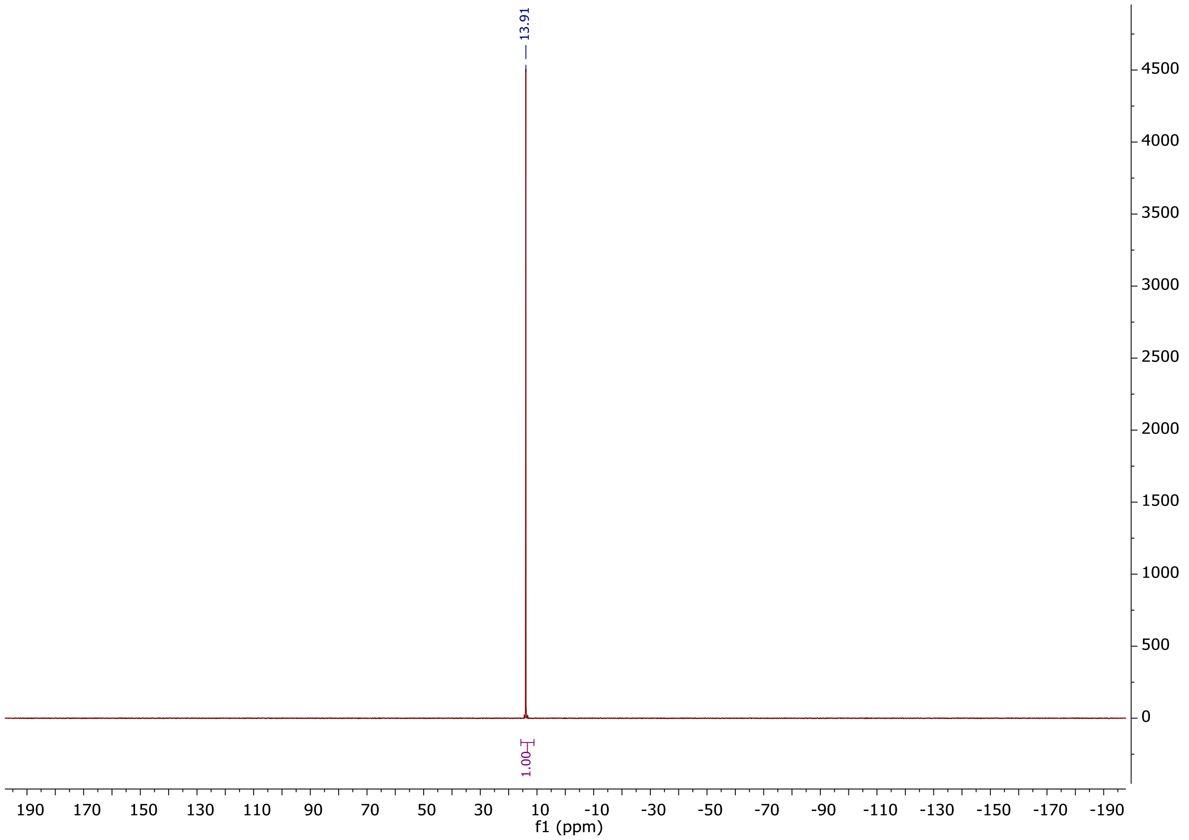

Compound **P-LG2**

^{1}H NMR (400 MHz, $CDCl_3$):

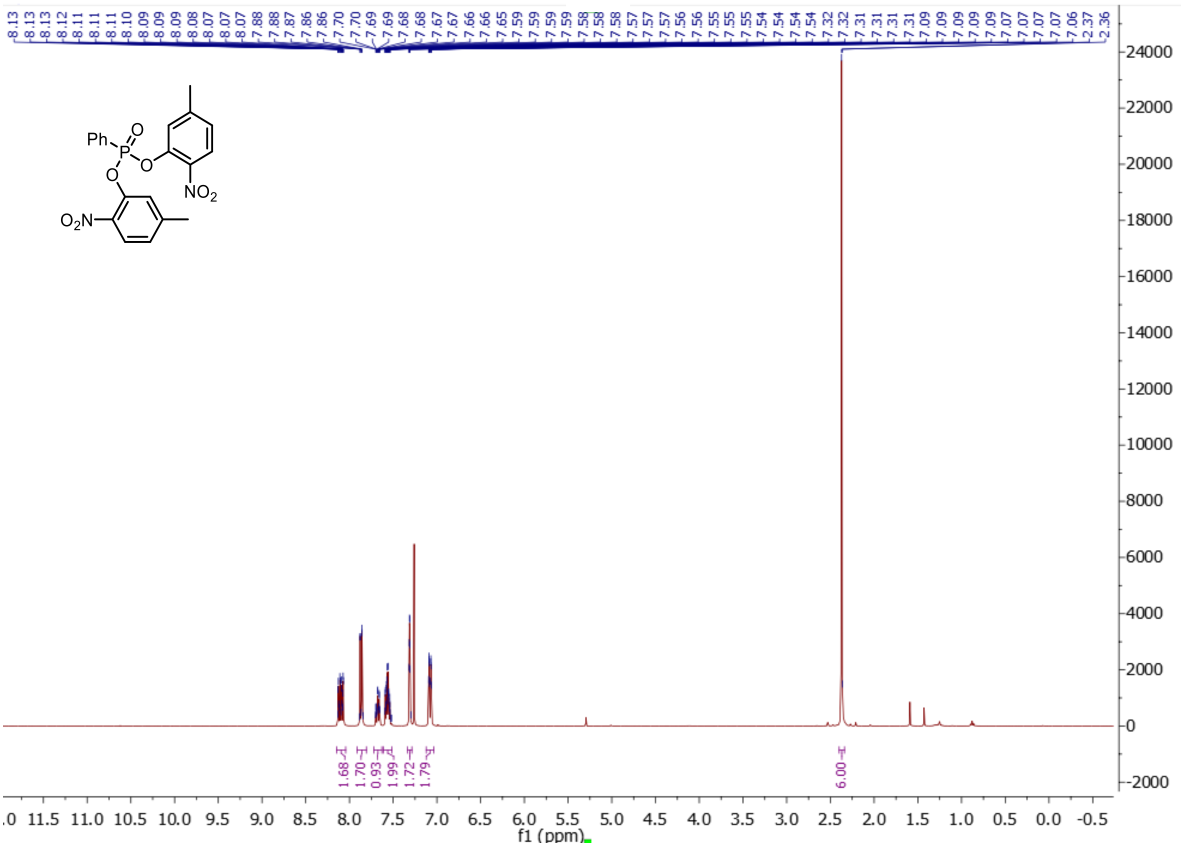

^{13}C NMR (101 MHz, $CDCl_3$):

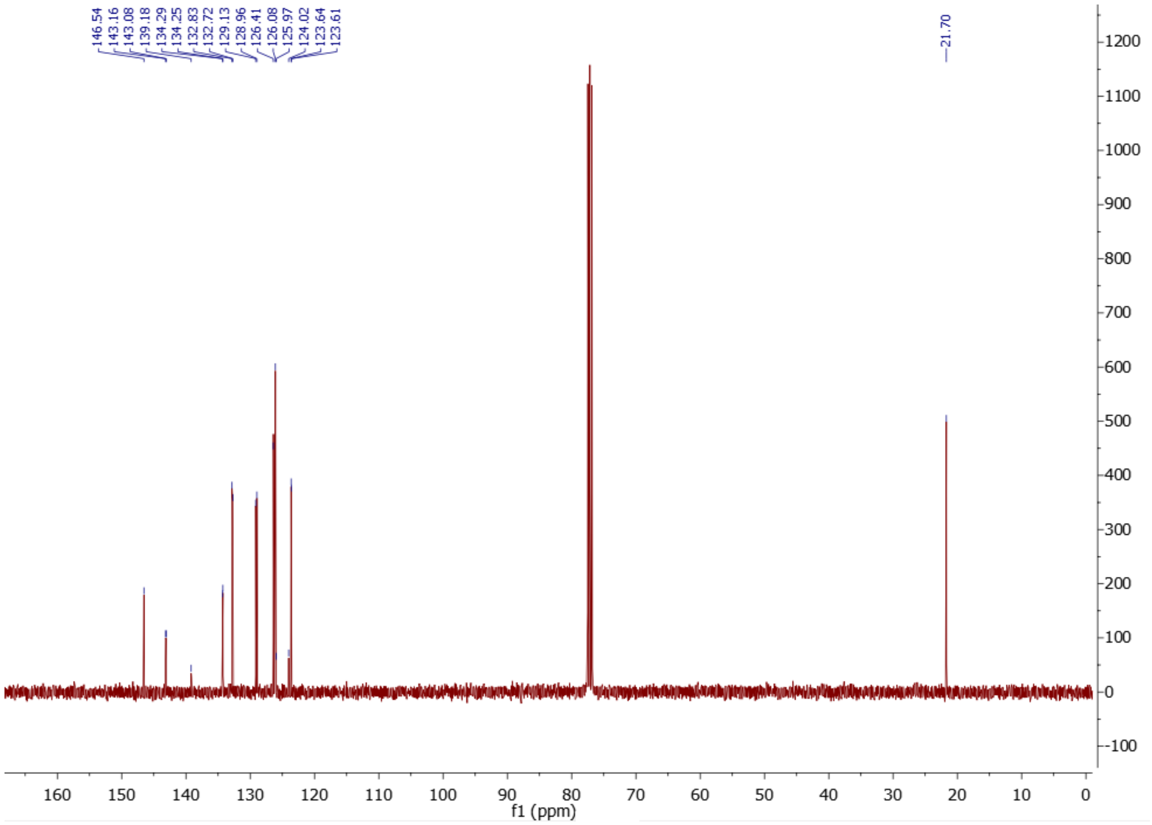

^{31}P NMR (162 MHz, $CDCl_3$):

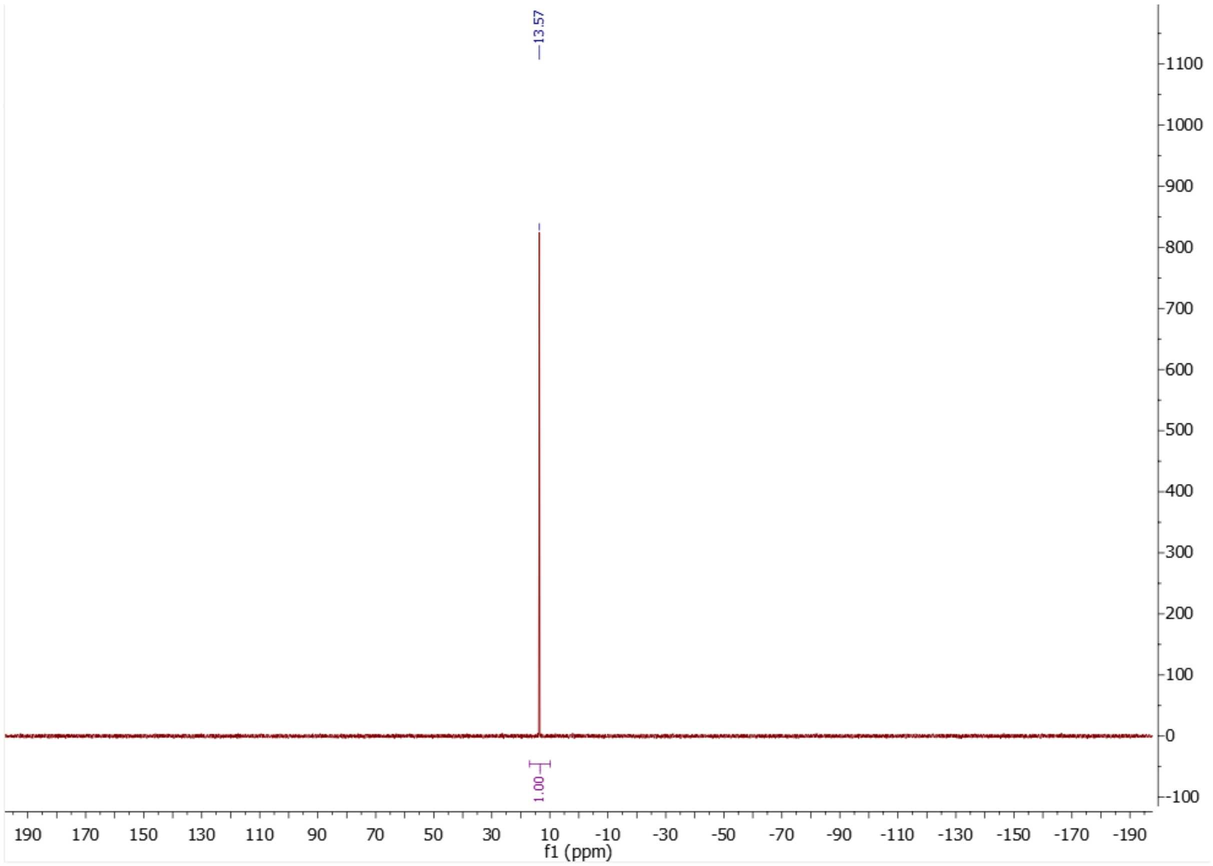

Compound **P-LG3**

^{1}H NMR (400 MHz, $CDCl_3$):

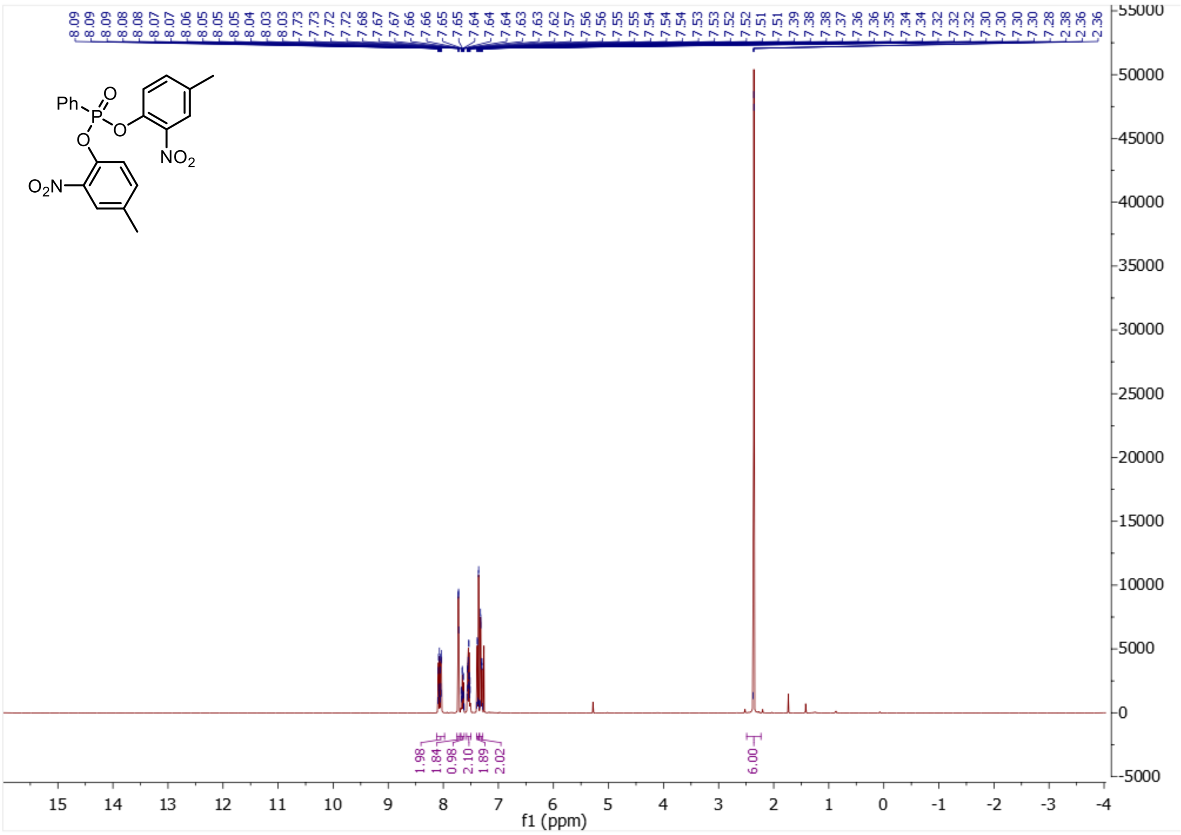

^{13}C NMR (101 MHz, $CDCl_3$):

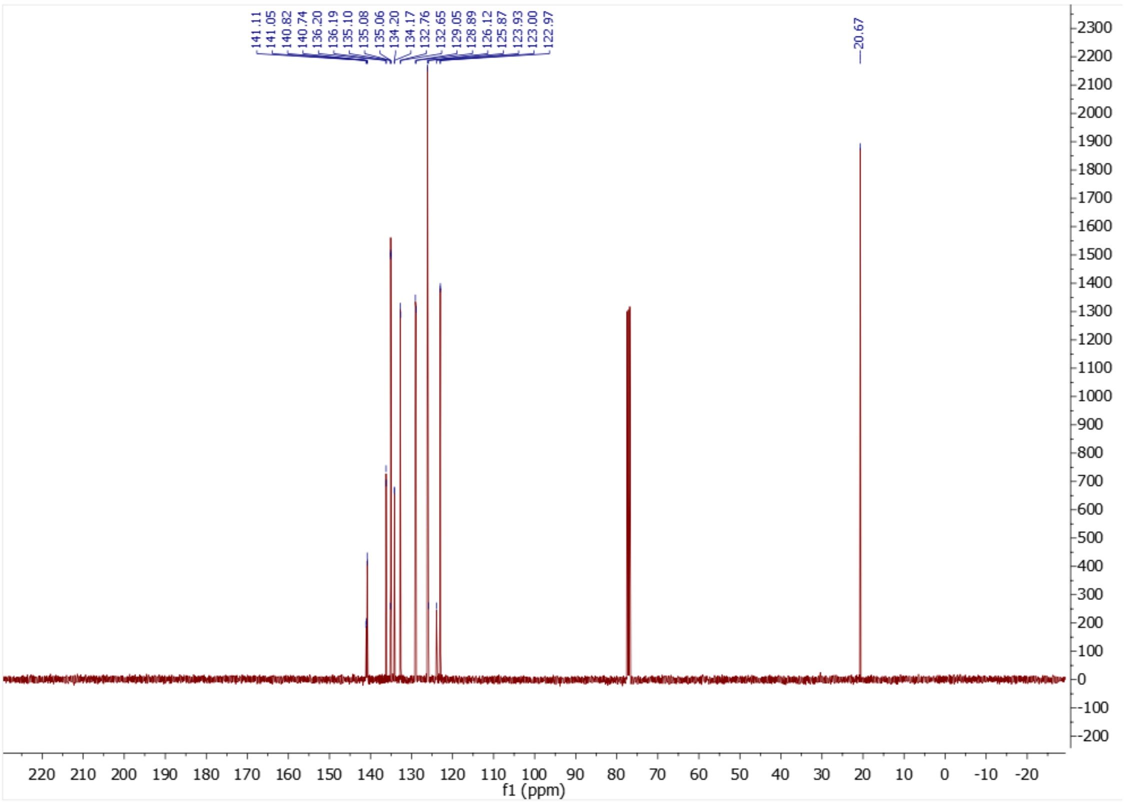

^{31}P NMR (162 MHz, CDCl$_3$):

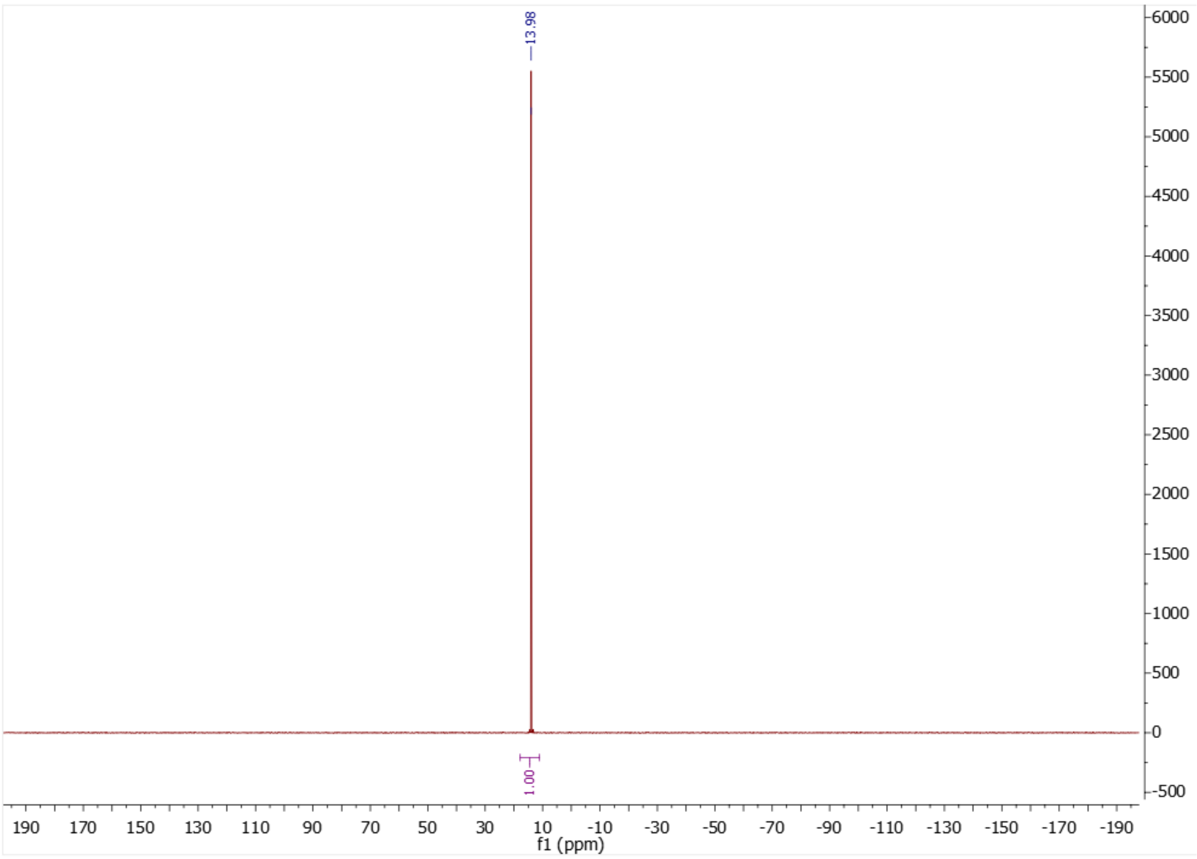

Compound **P1**

^{1}H NMR (400 MHz, $CDCl_3$):

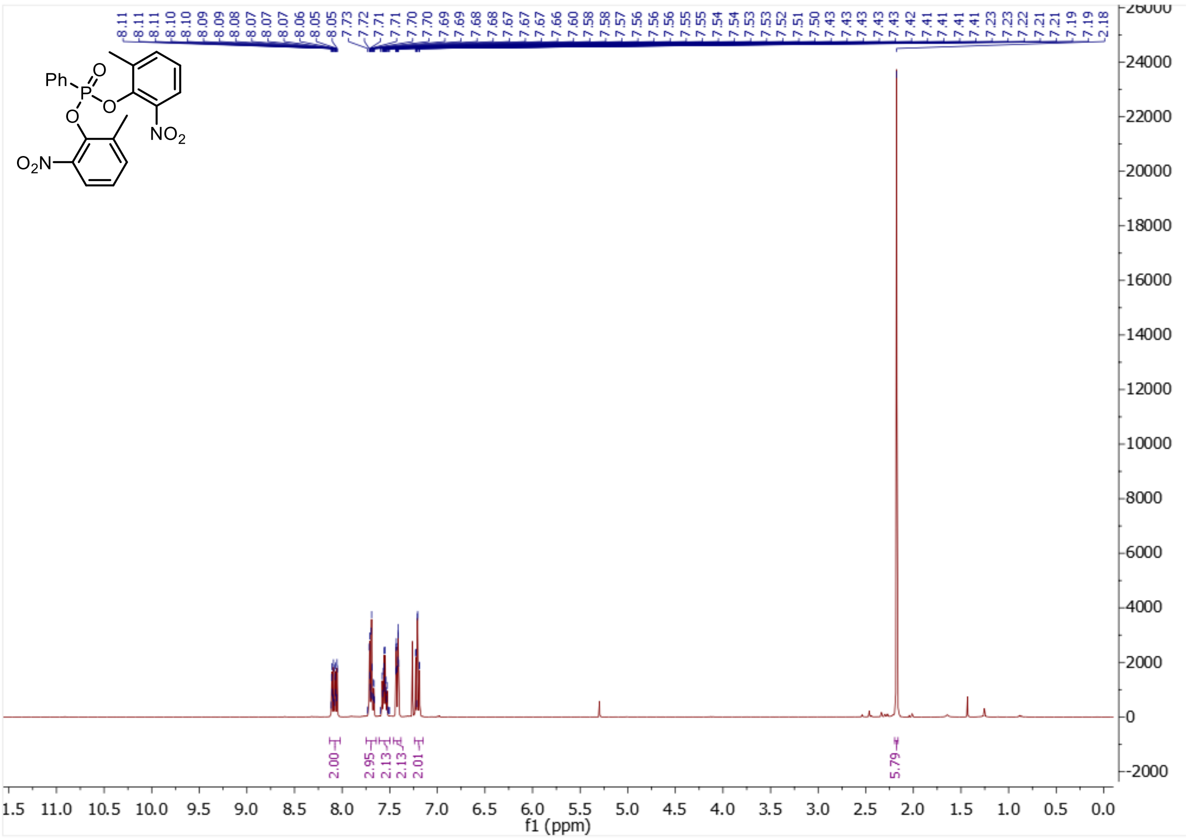

^{13}C NMR (101 MHz, $CDCl_3$):

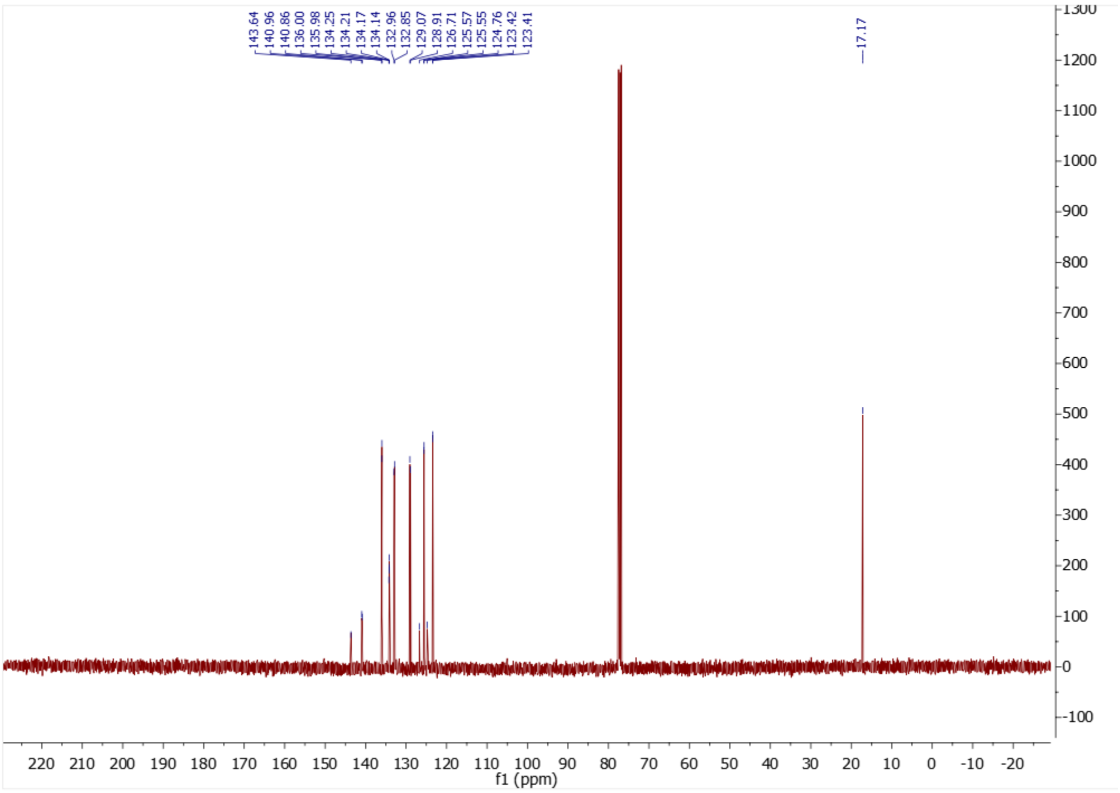

^{31}P NMR (162 MHz, $CDCl_3$):

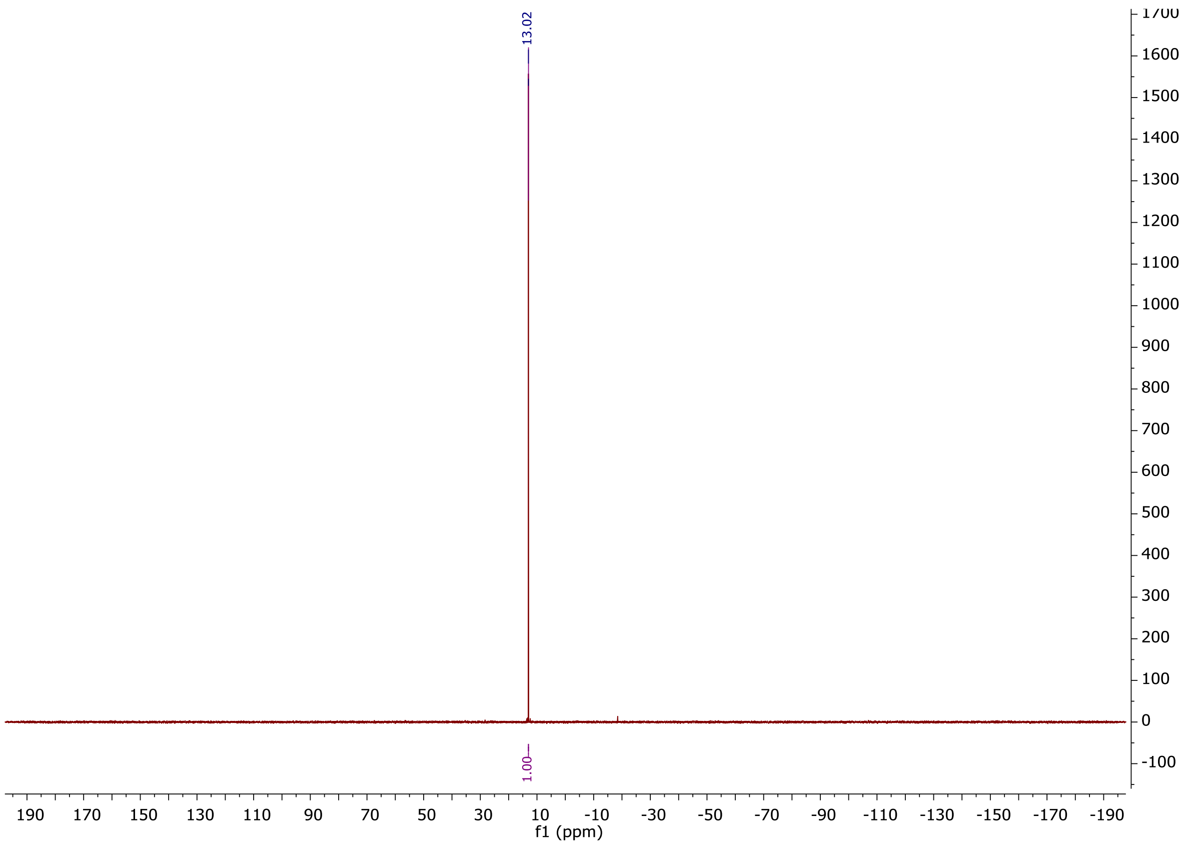

Compound **P2**

^{1}H NMR (400 MHz, $CDCl_3$):

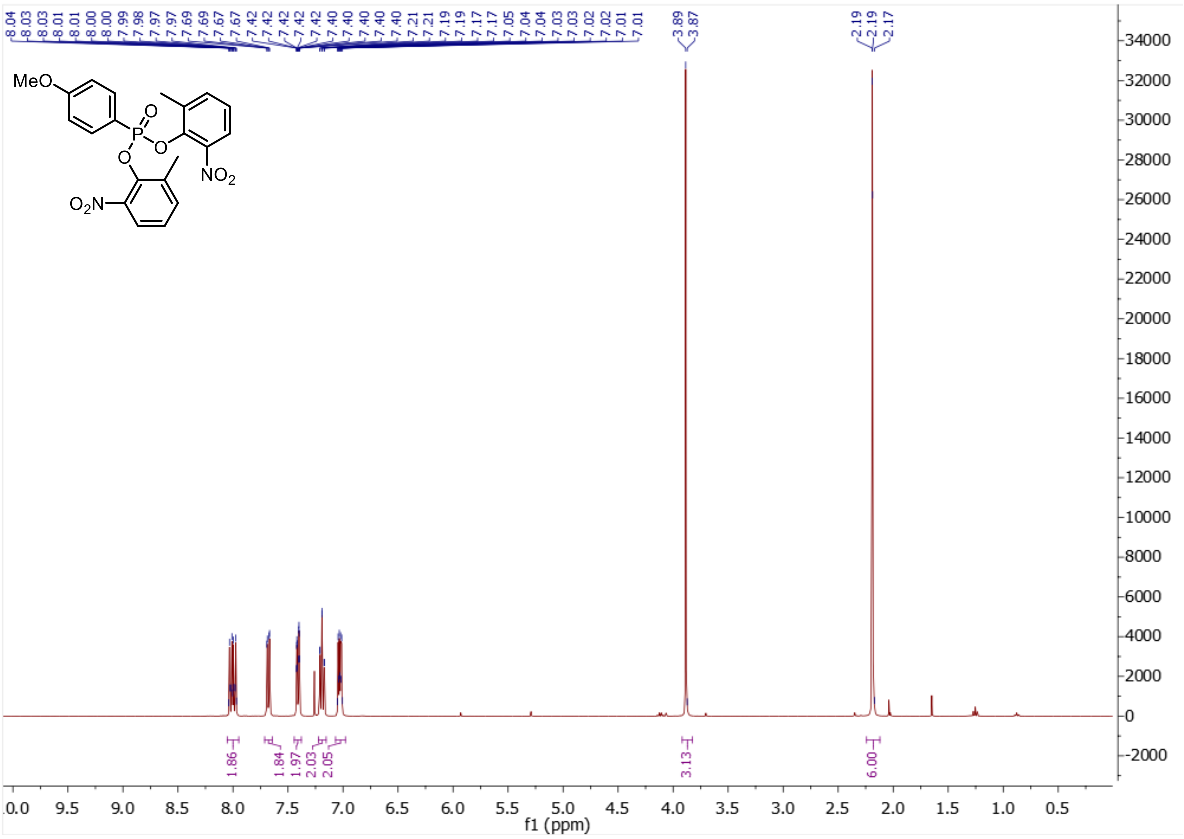

^{13}C NMR (101 MHz, $CDCl_3$):

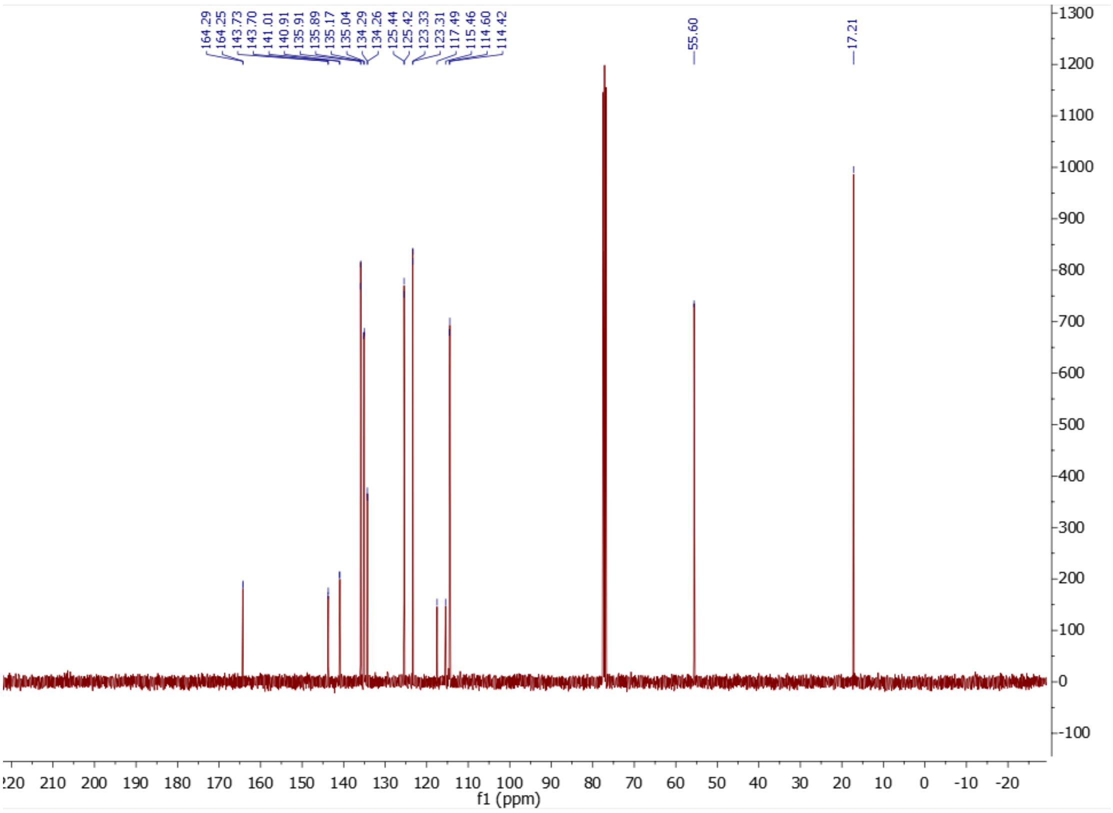

^{31}P NMR (162 MHz, $CDCl_3$):

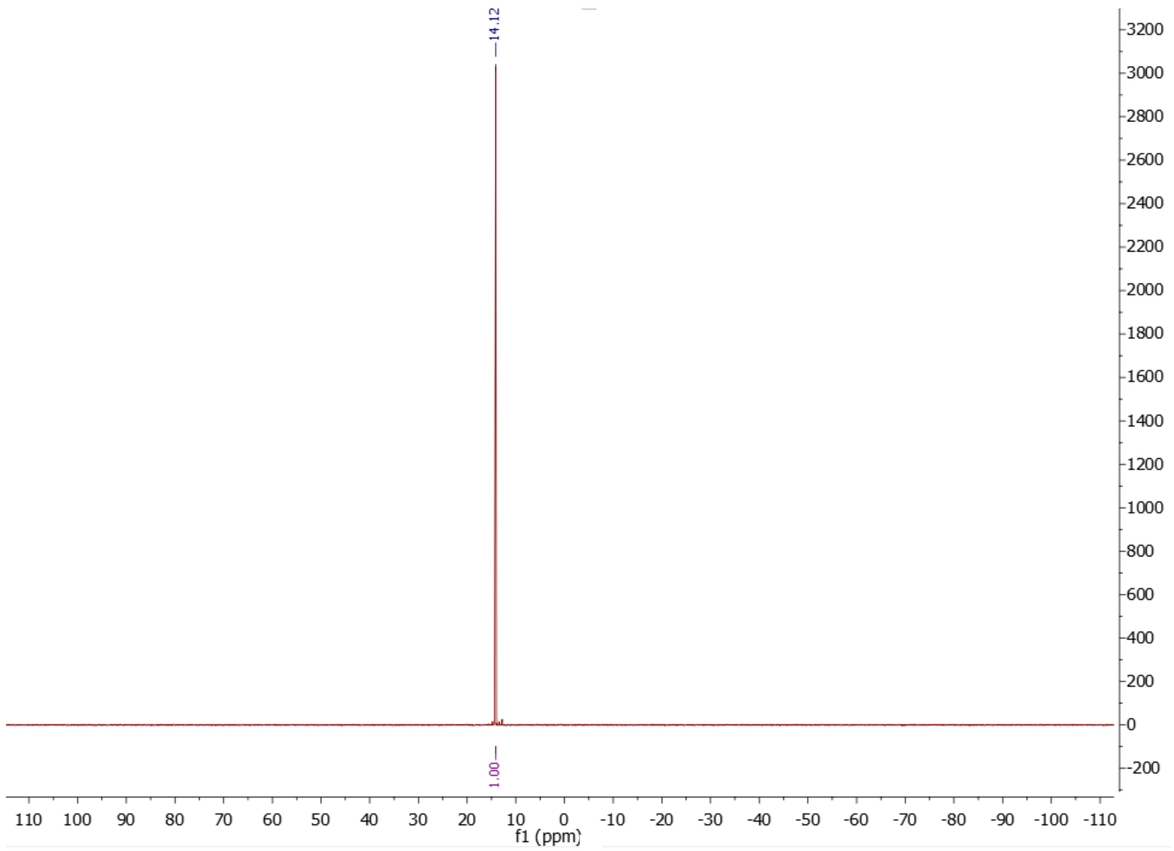

Compound **P3**

^{1}H NMR (400 MHz, $CDCl_3$):

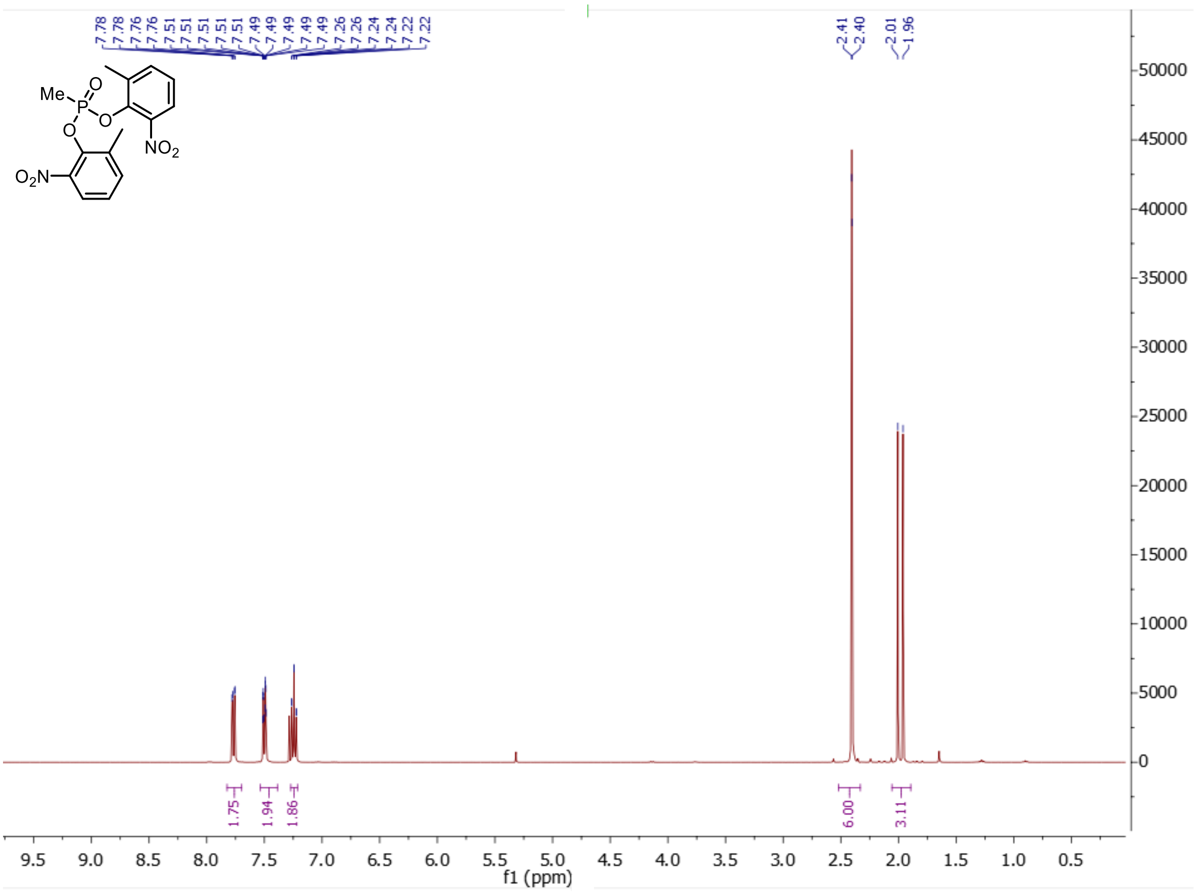

^{13}C NMR (101 MHz, $CDCl_3$):

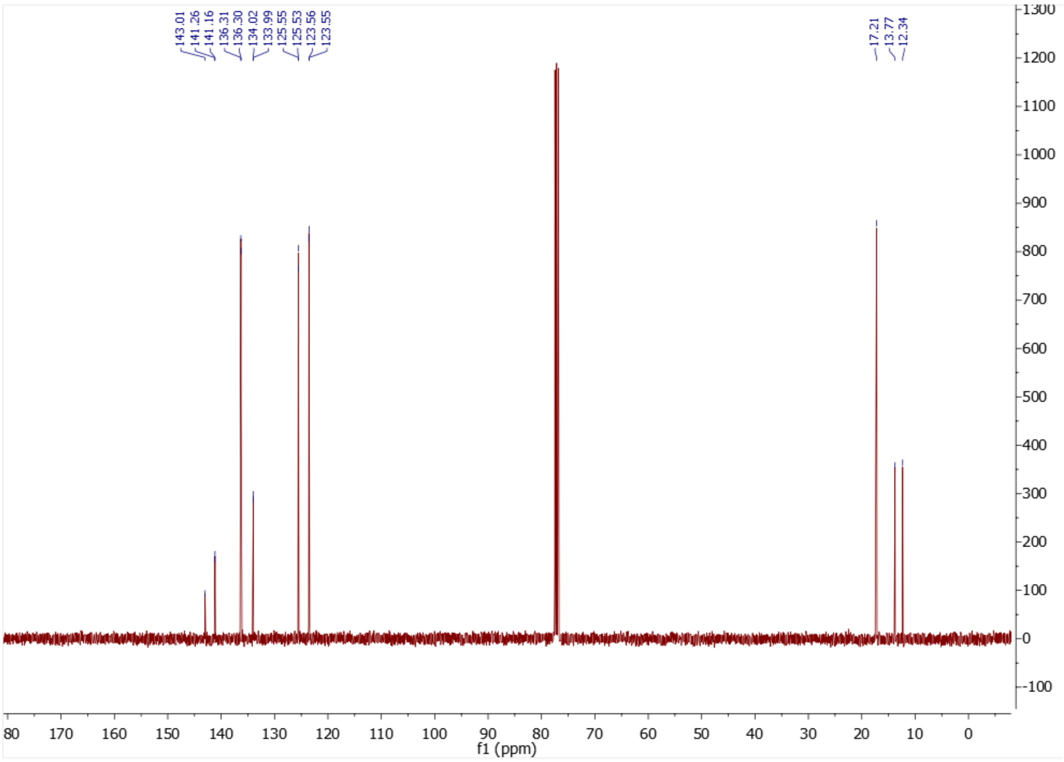

^{31}P NMR (162 MHz, $CDCl_3$):

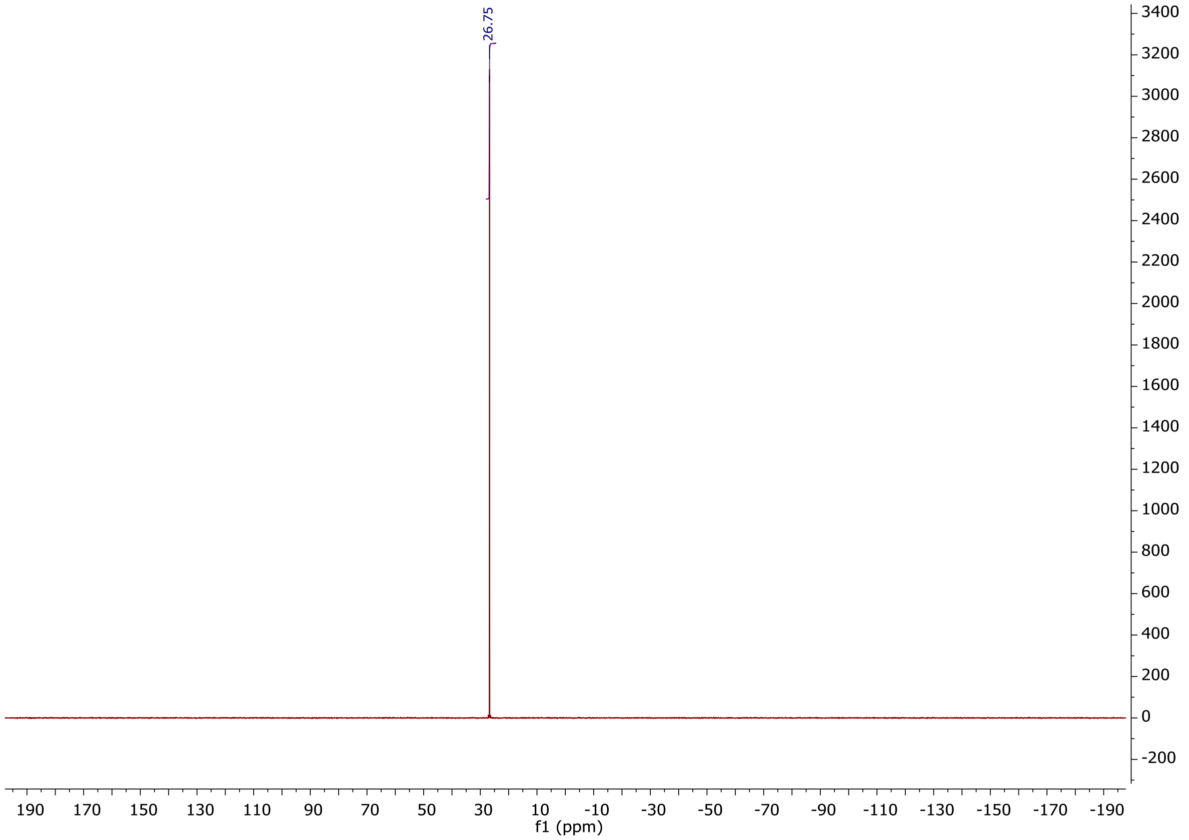

Compound **P4**

^{1}H NMR (400 MHz, $CDCl_3$):

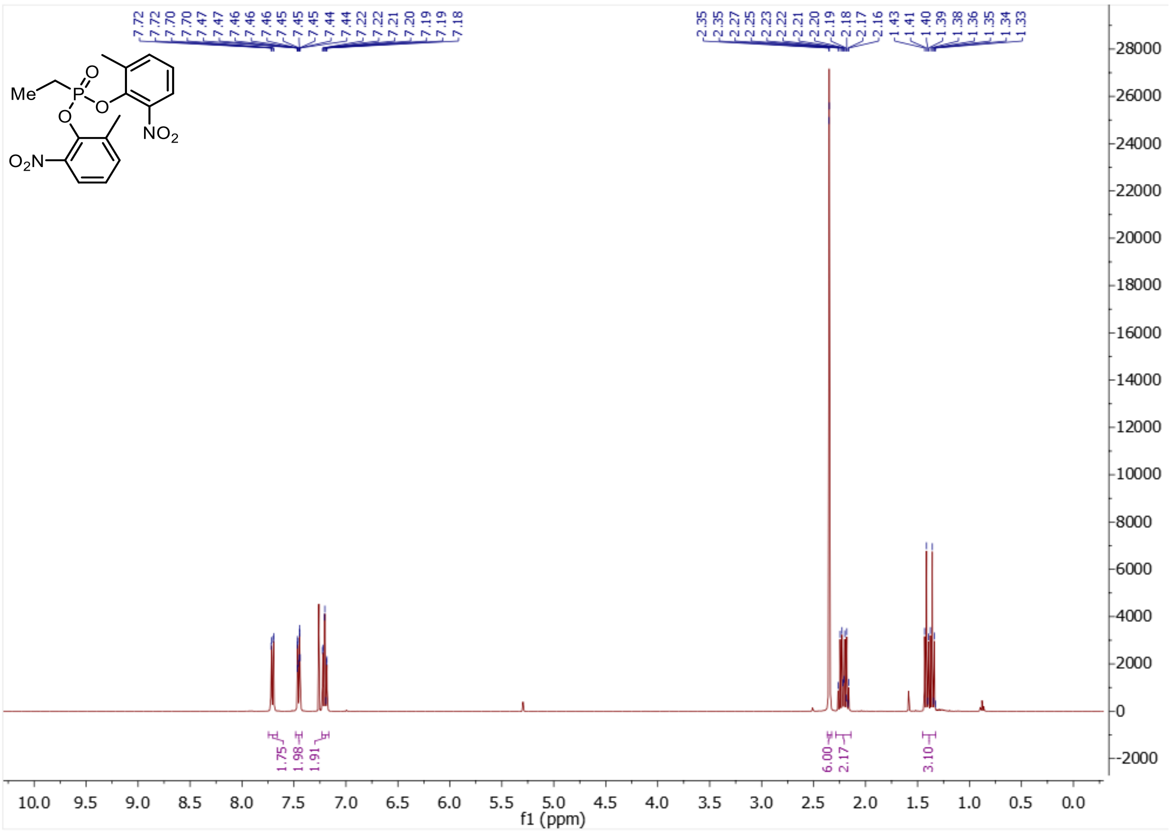

^{13}C NMR (101 MHz, $CDCl_3$):

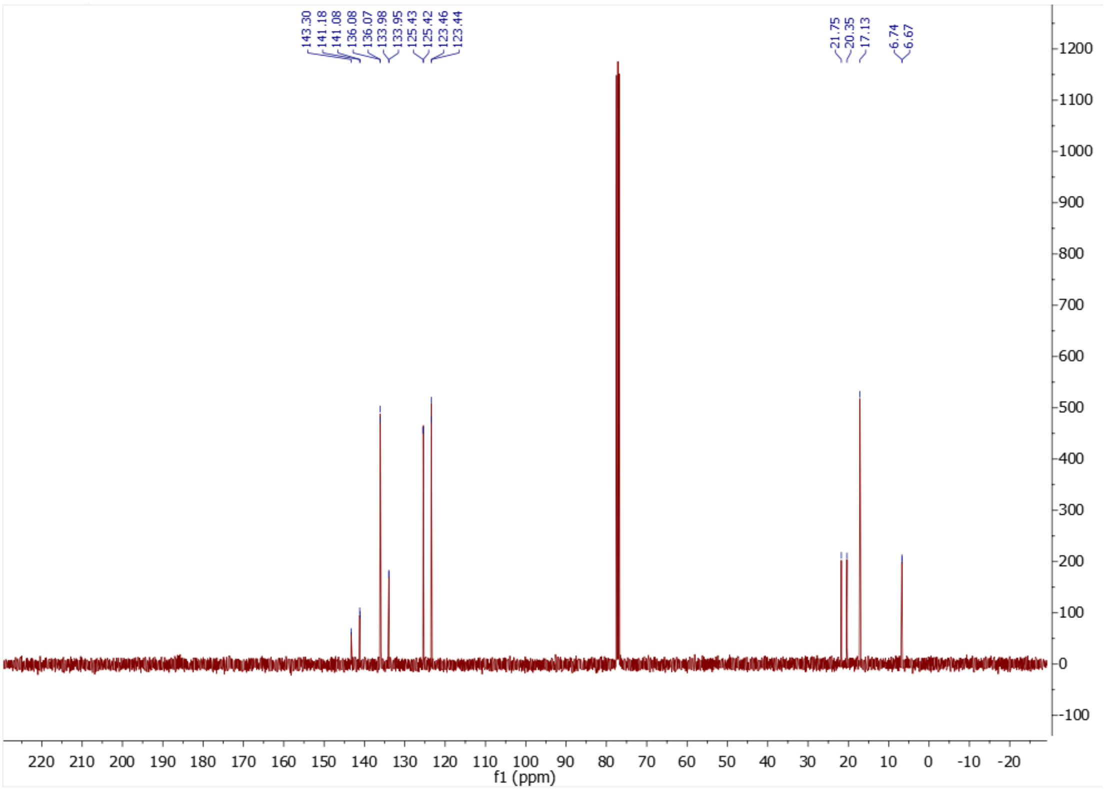

^{31}P NMR (162 MHz, CDCl$_3$):

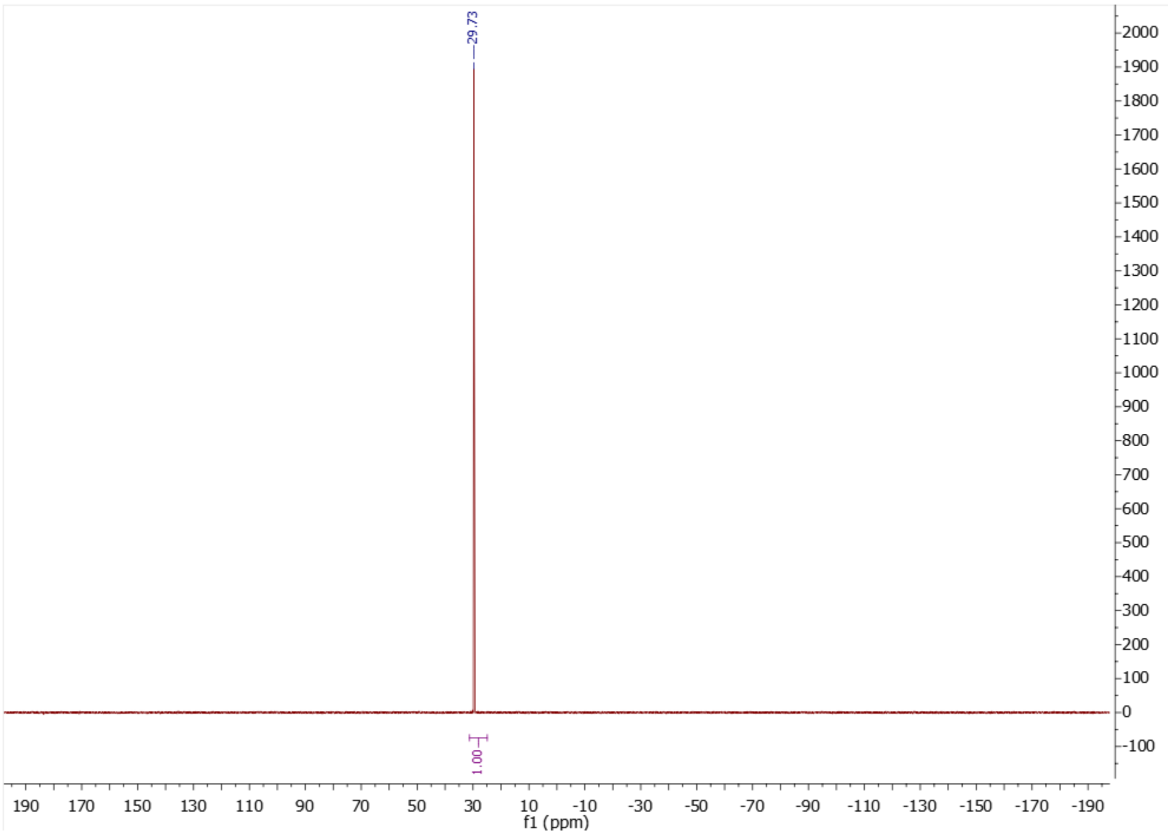

Compound **P5**

^{1}H NMR (400 MHz, CDCl$_3$):

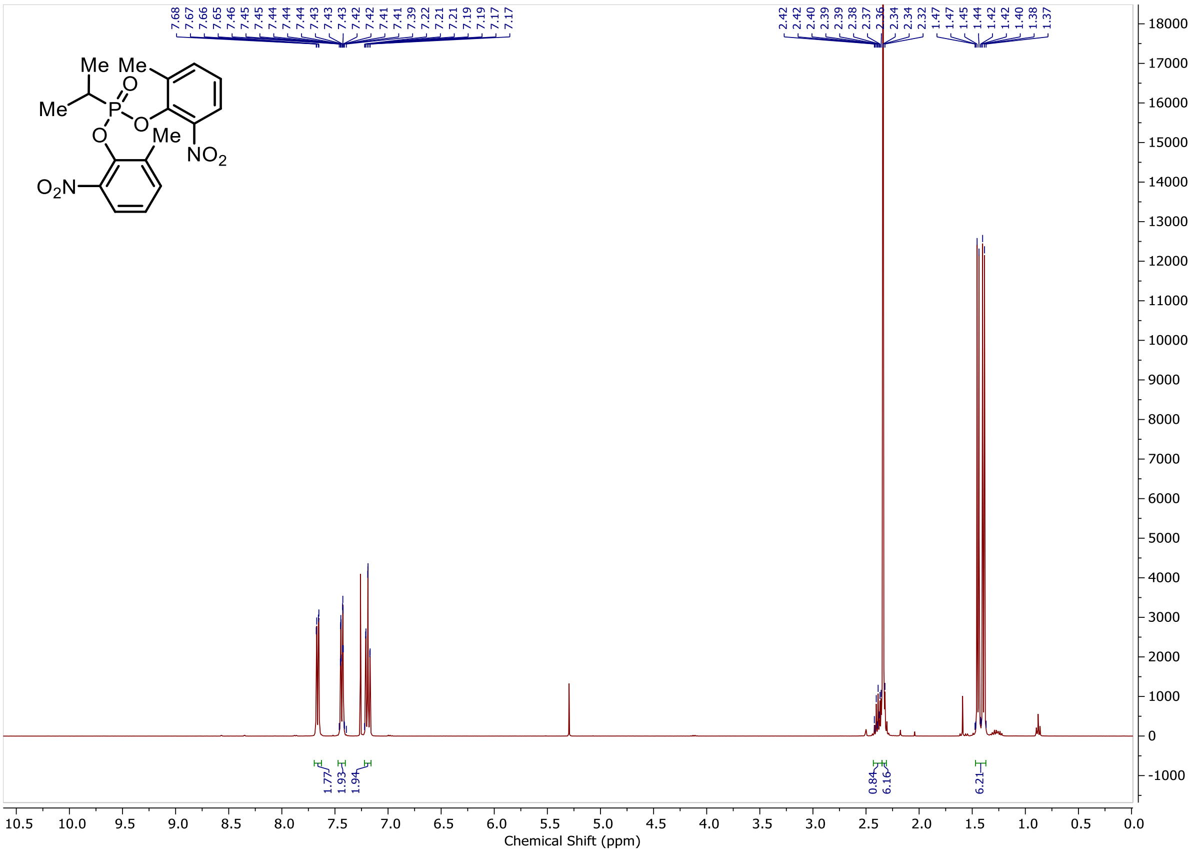

^{13}C NMR (101 MHz, CDCl$_3$):

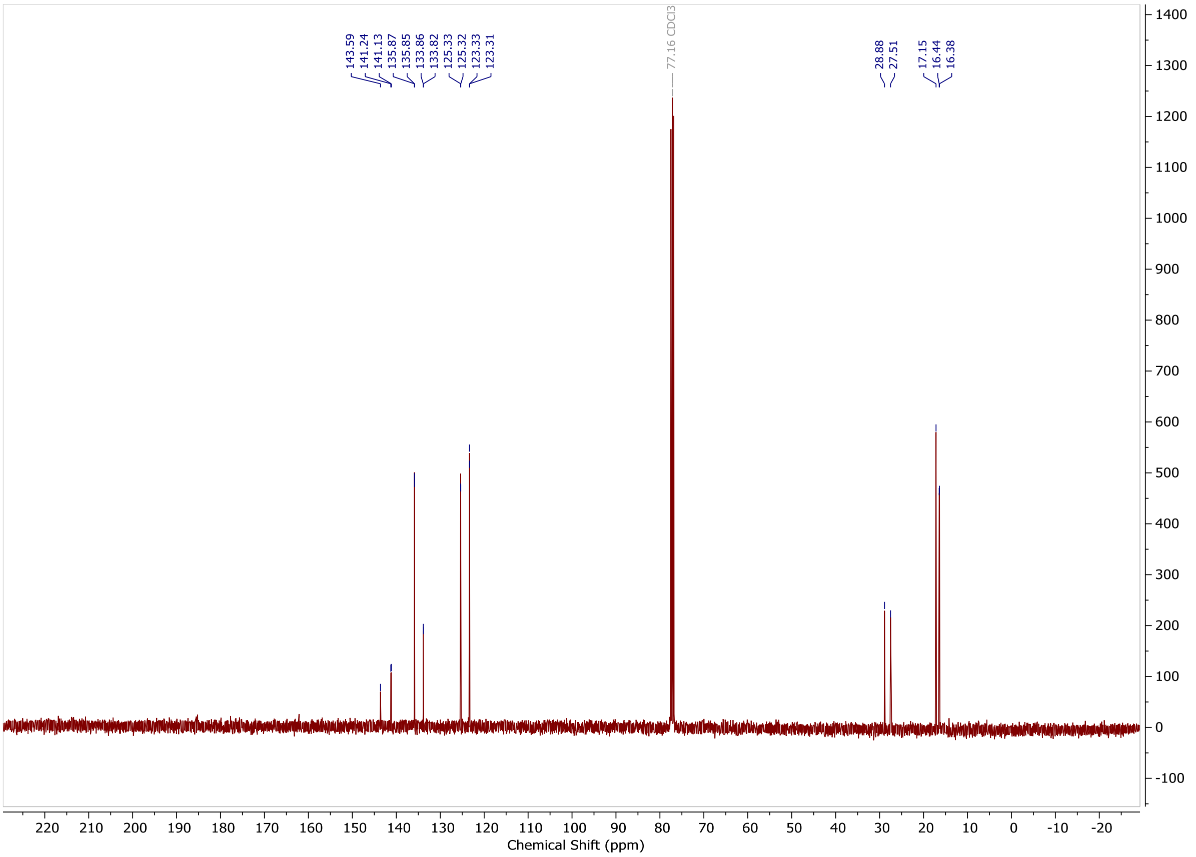

^{31}P NMR (162 MHz, $CDCl_3$):

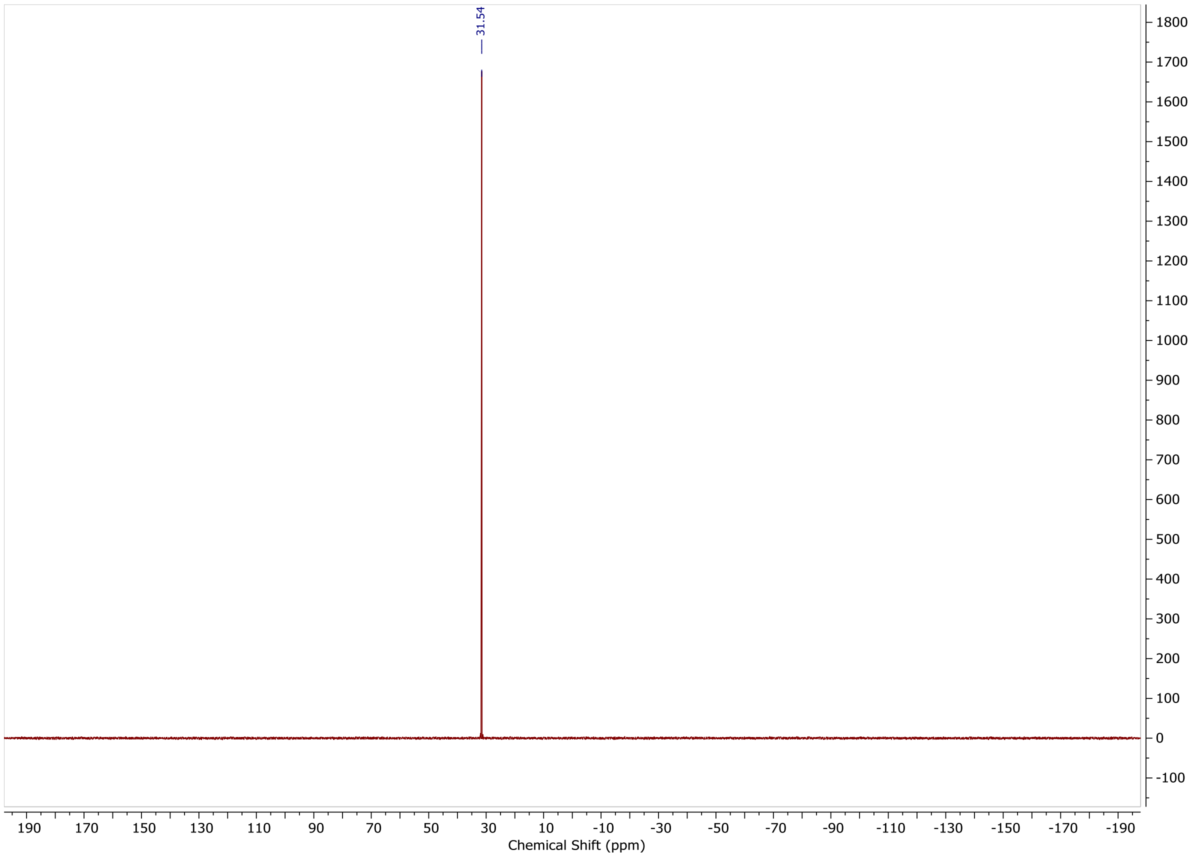

Compound **P6**

^{1}H NMR (400 MHz, $CDCl_3$):

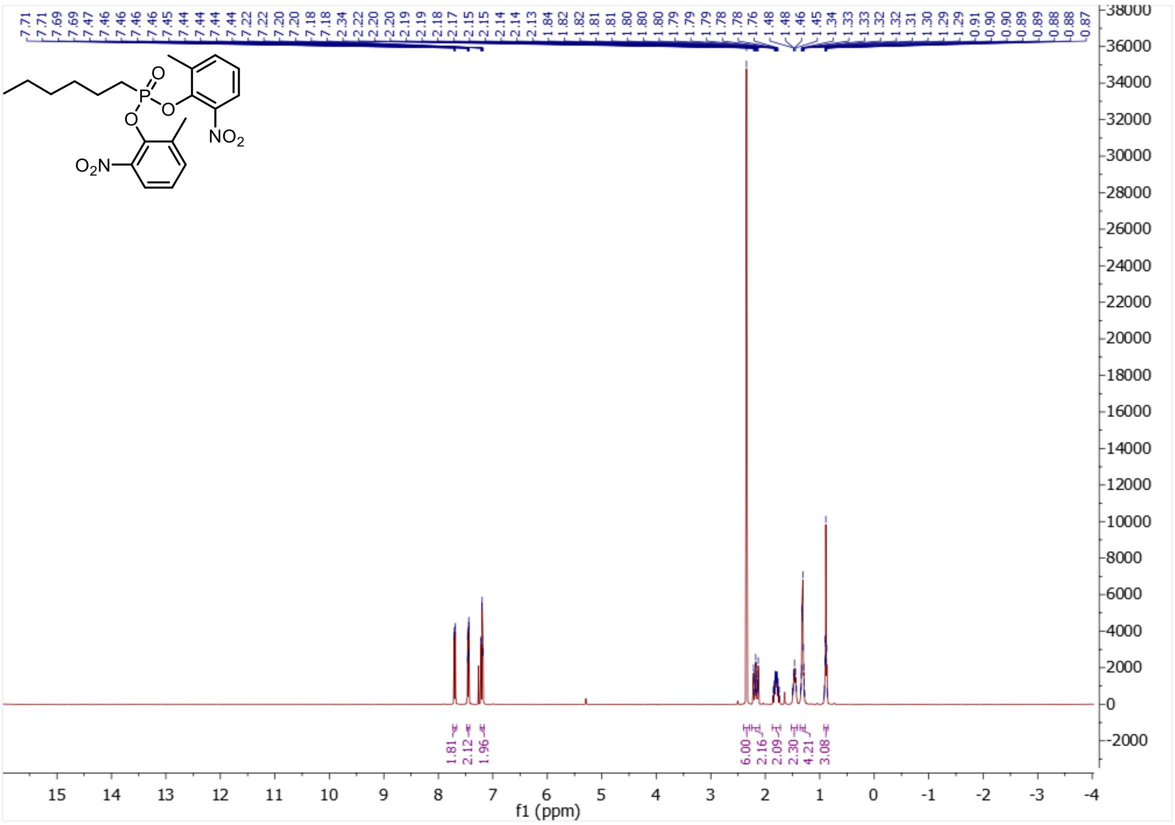

^{13}C NMR (101 MHz, $CDCl_3$):

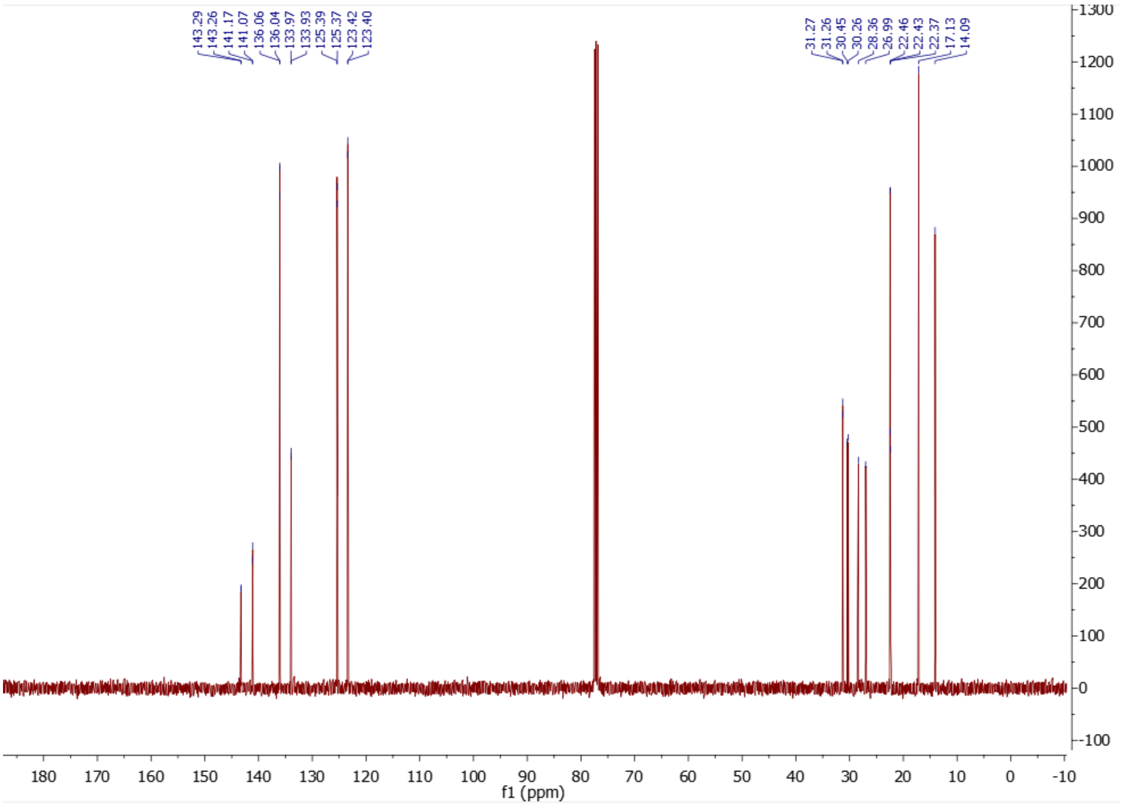

^{31}P NMR (162 MHz, CDCl$_3$):

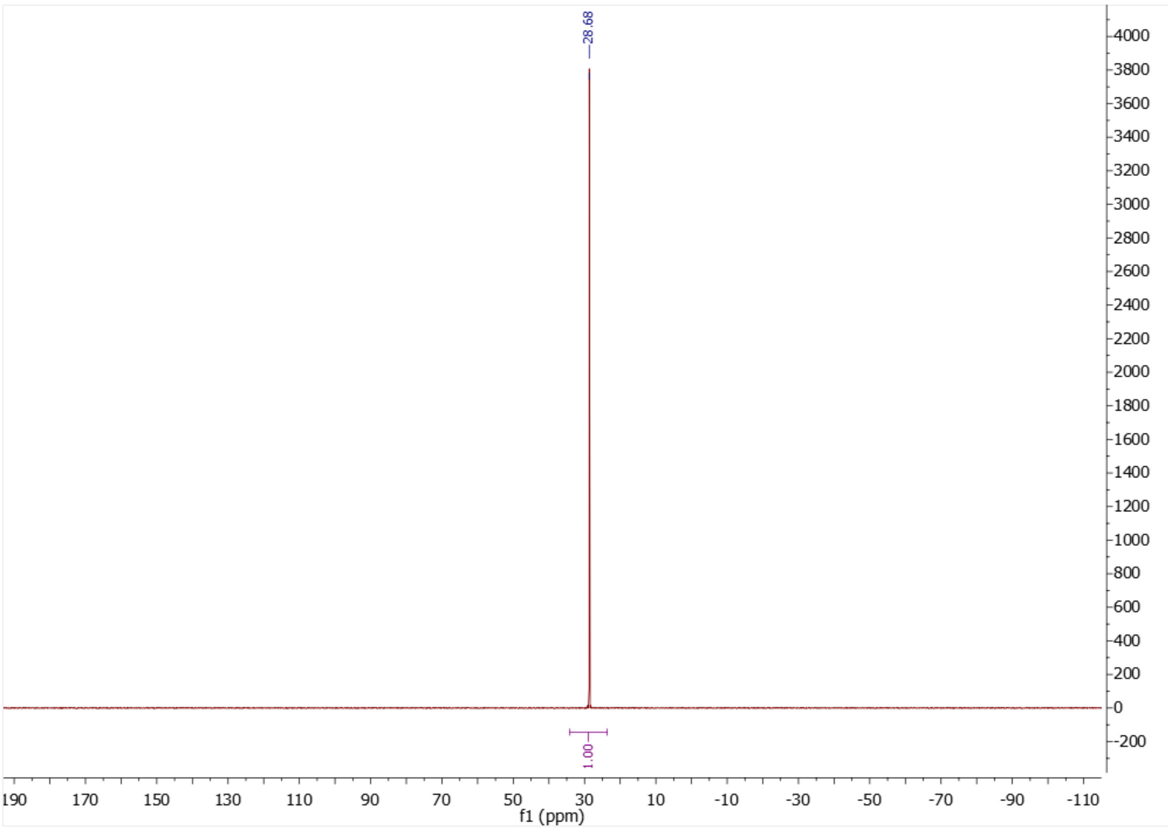

Compound **P7**

^{1}H NMR (400 MHz, $CDCl_3$):

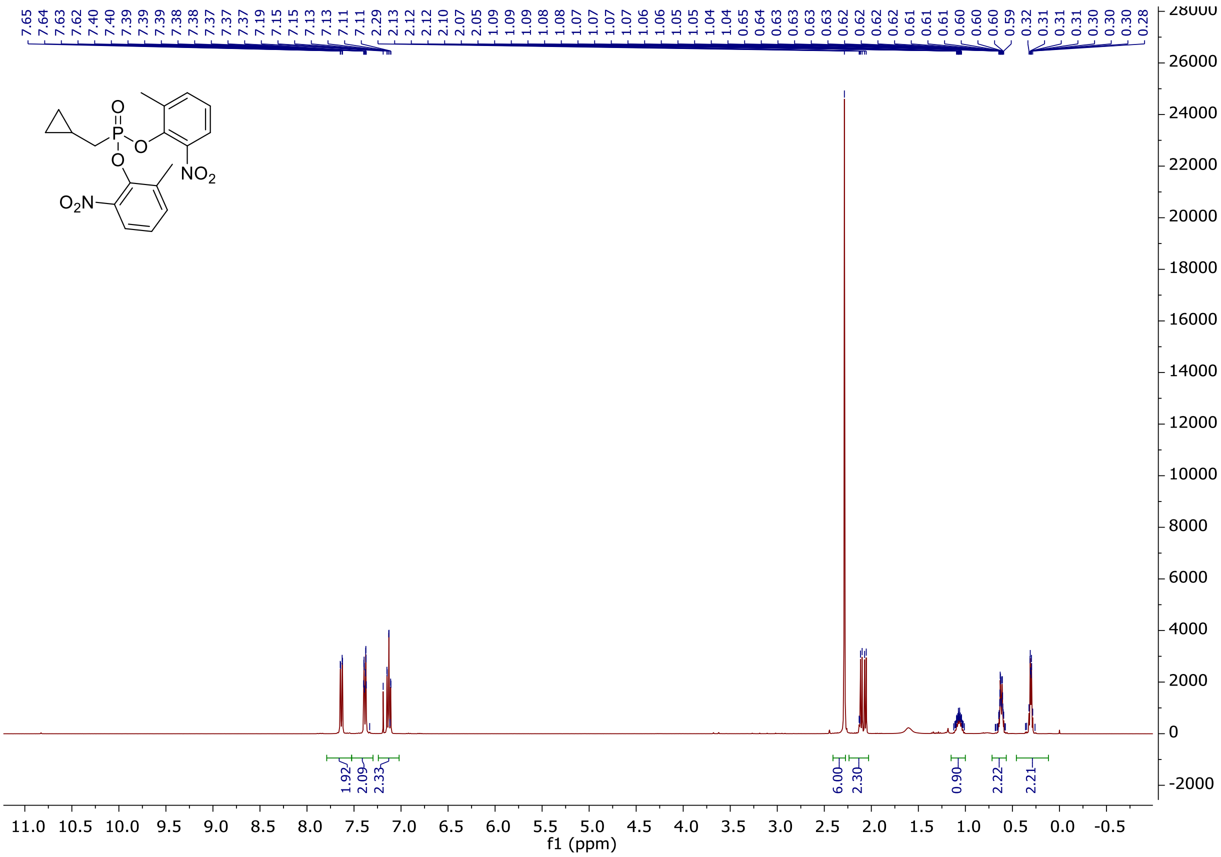

^{13}C NMR (101 MHz, $CDCl_3$):

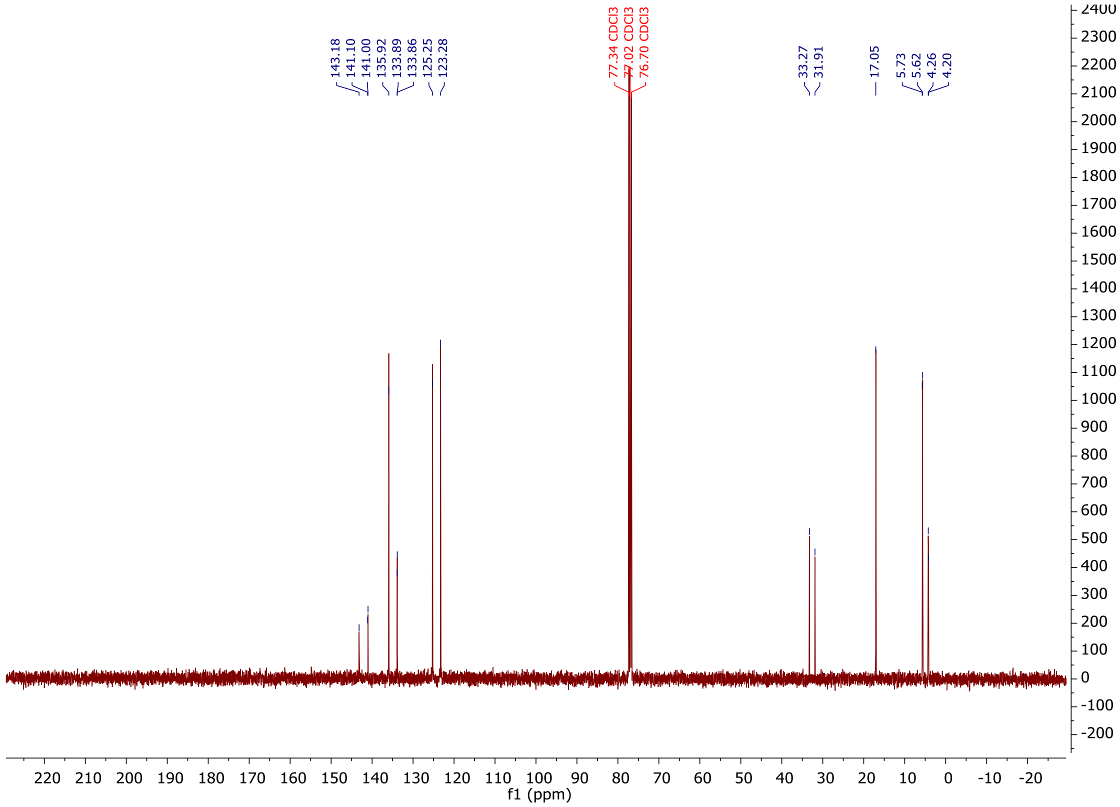

^{31}P NMR (162 MHz, $CDCl_3$):

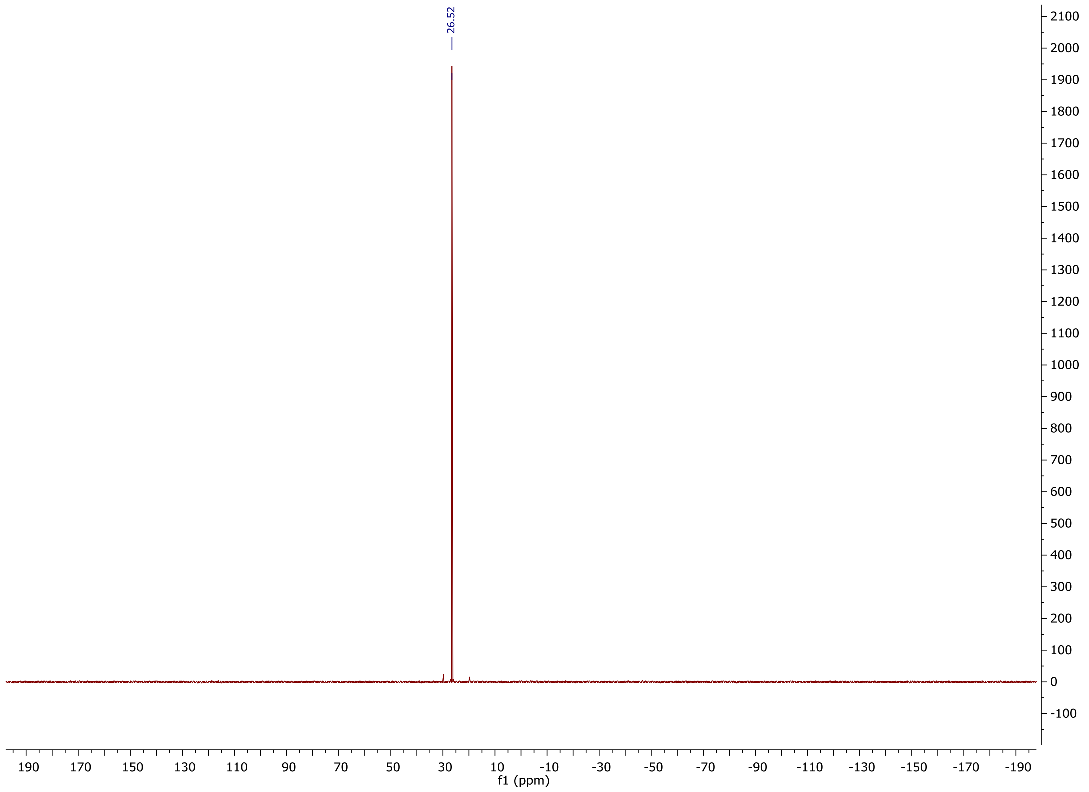

Compound **P8**

^{1}H NMR (400 MHz, $CDCl_3$):

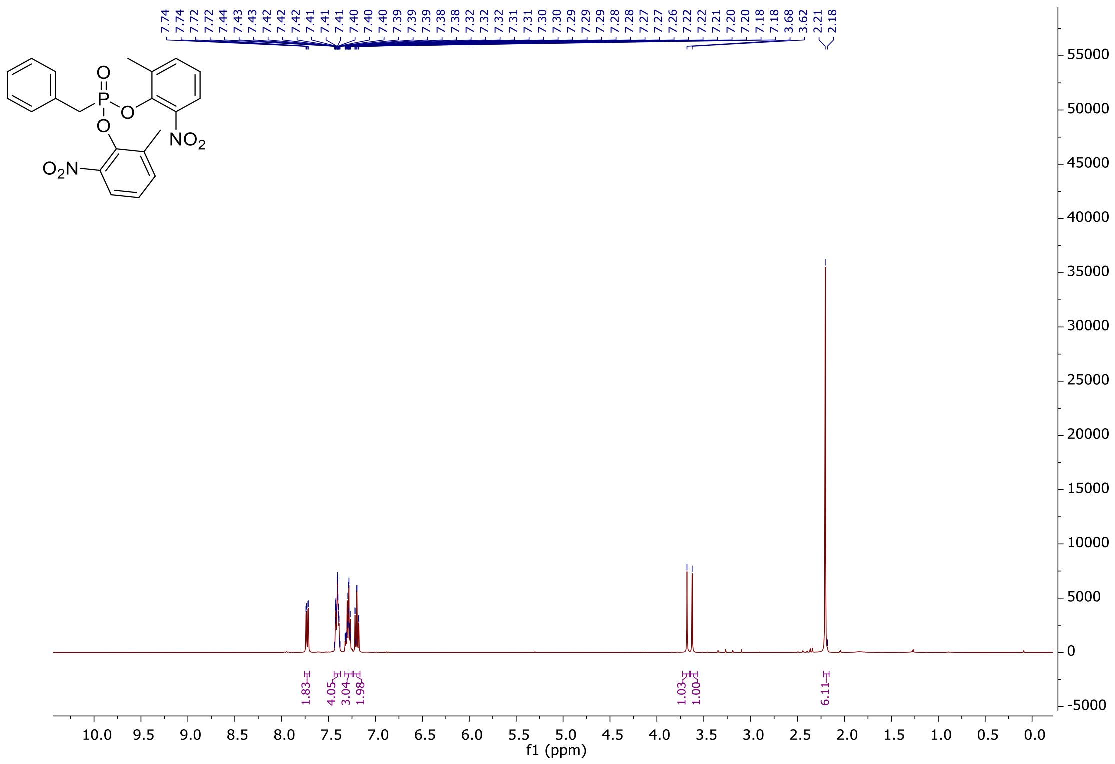

^{13}C NMR (101 MHz, $CDCl_3$):

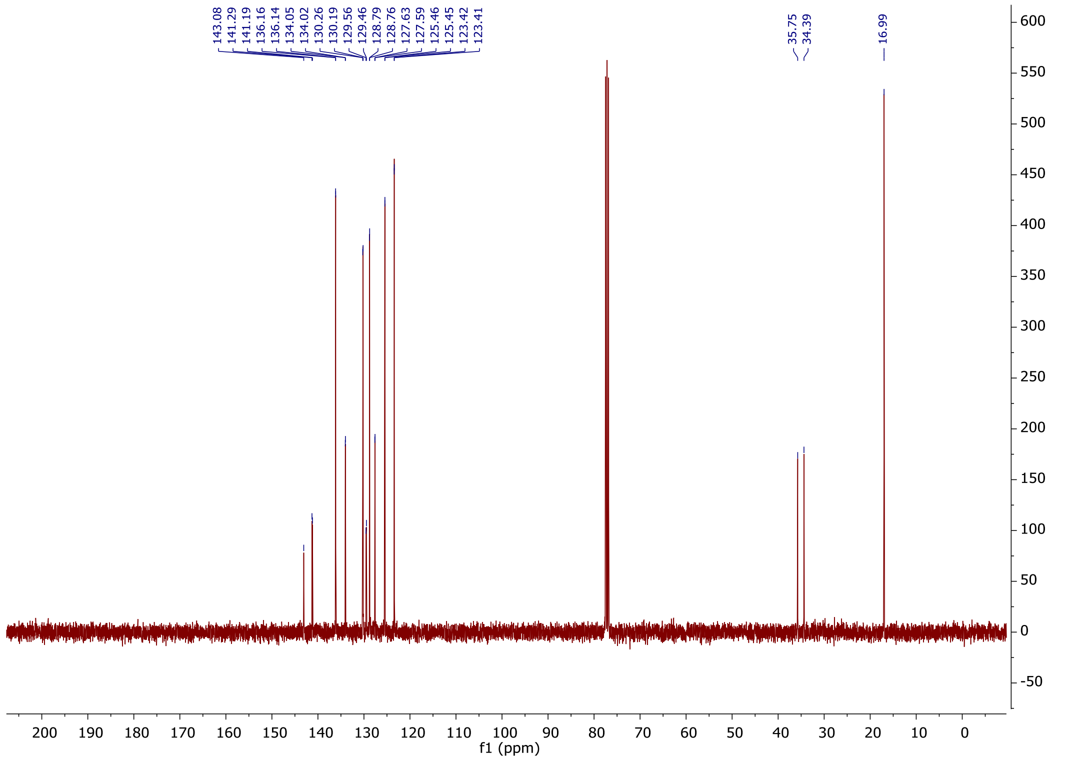

^{31}P NMR (162 MHz, $CDCl_3$):

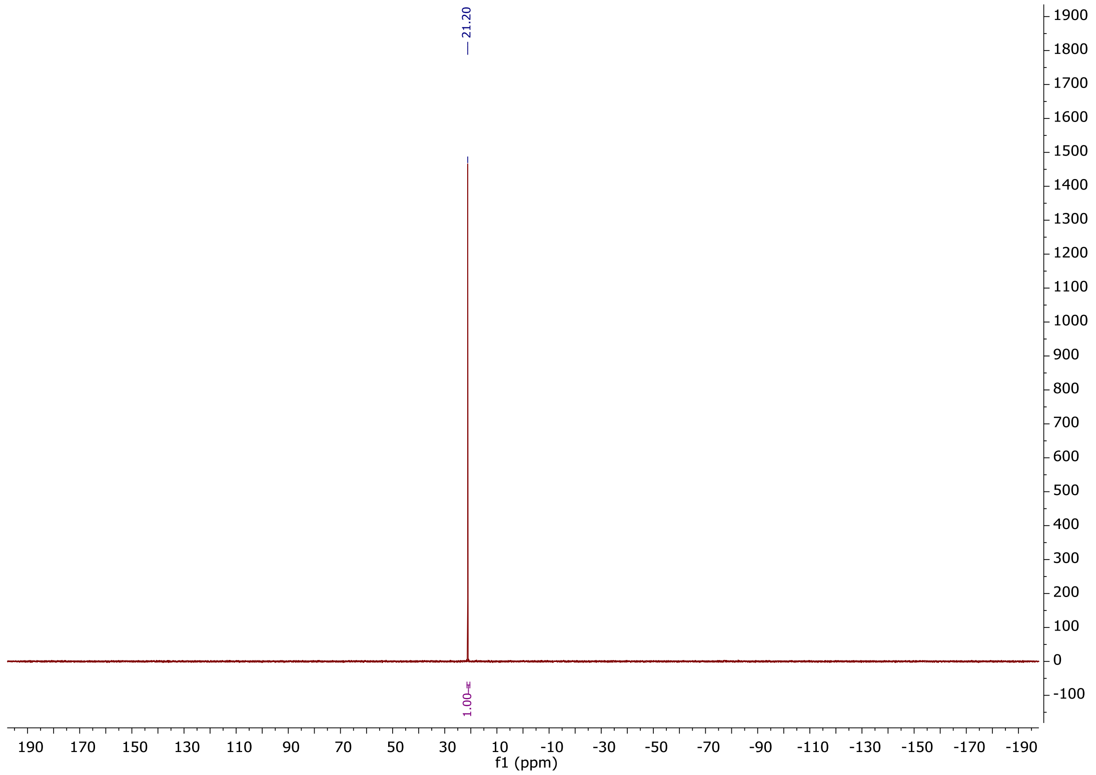

Compound **P9**

^{1}H NMR (400 MHz, $CDCl_3$):

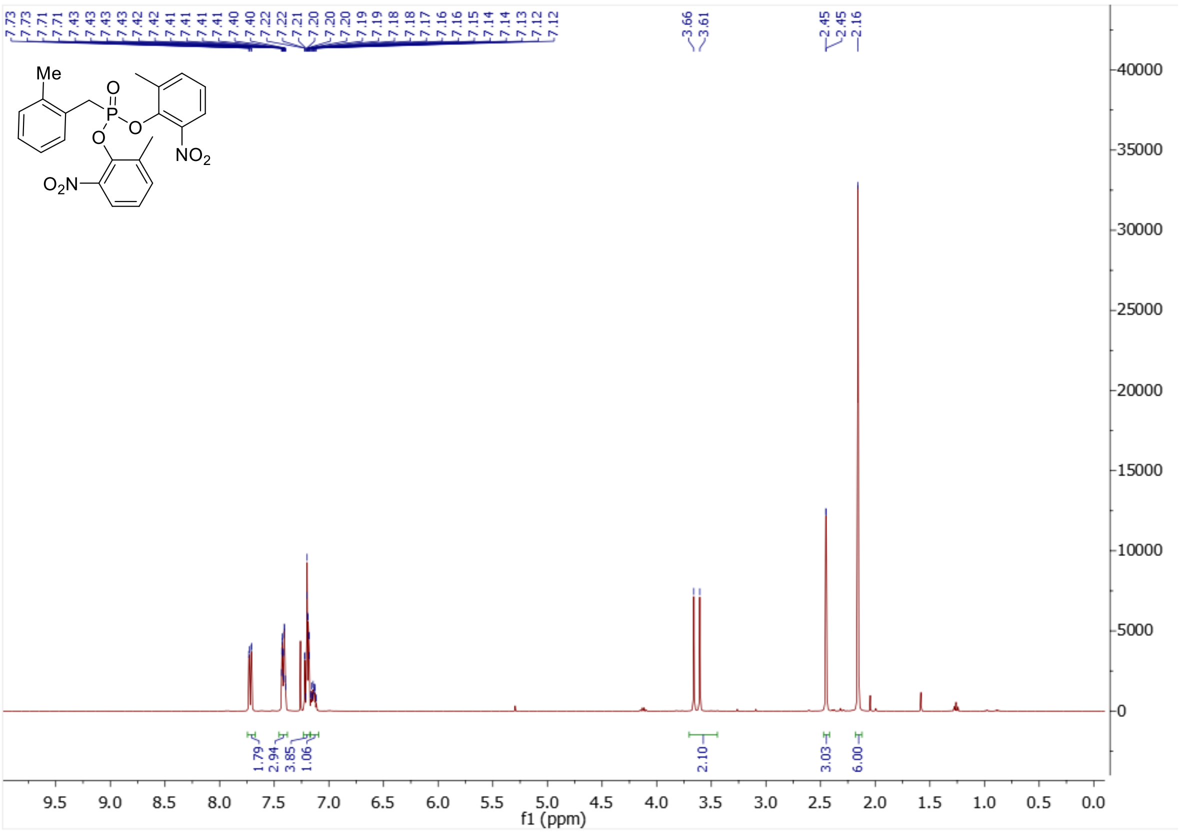

^{13}C NMR (101 MHz, $CDCl_3$):

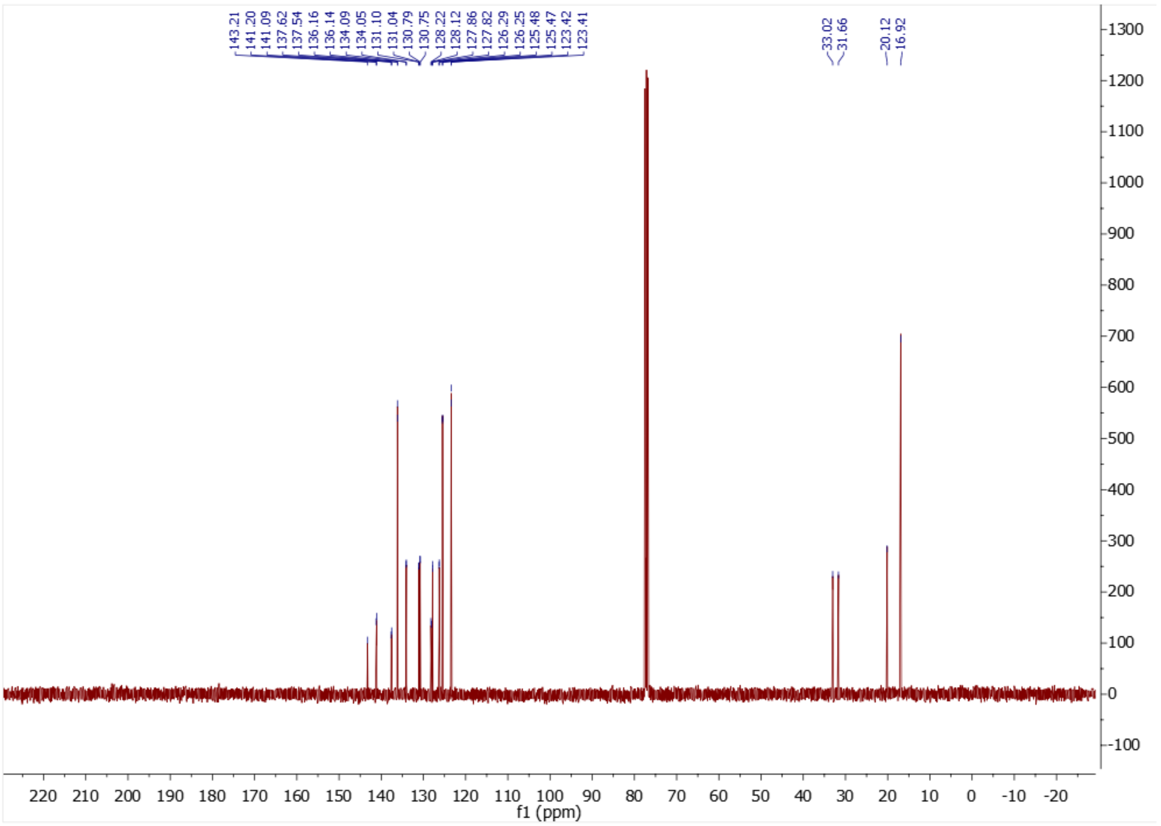

^{31}P NMR (162 MHz, CDCl$_3$):

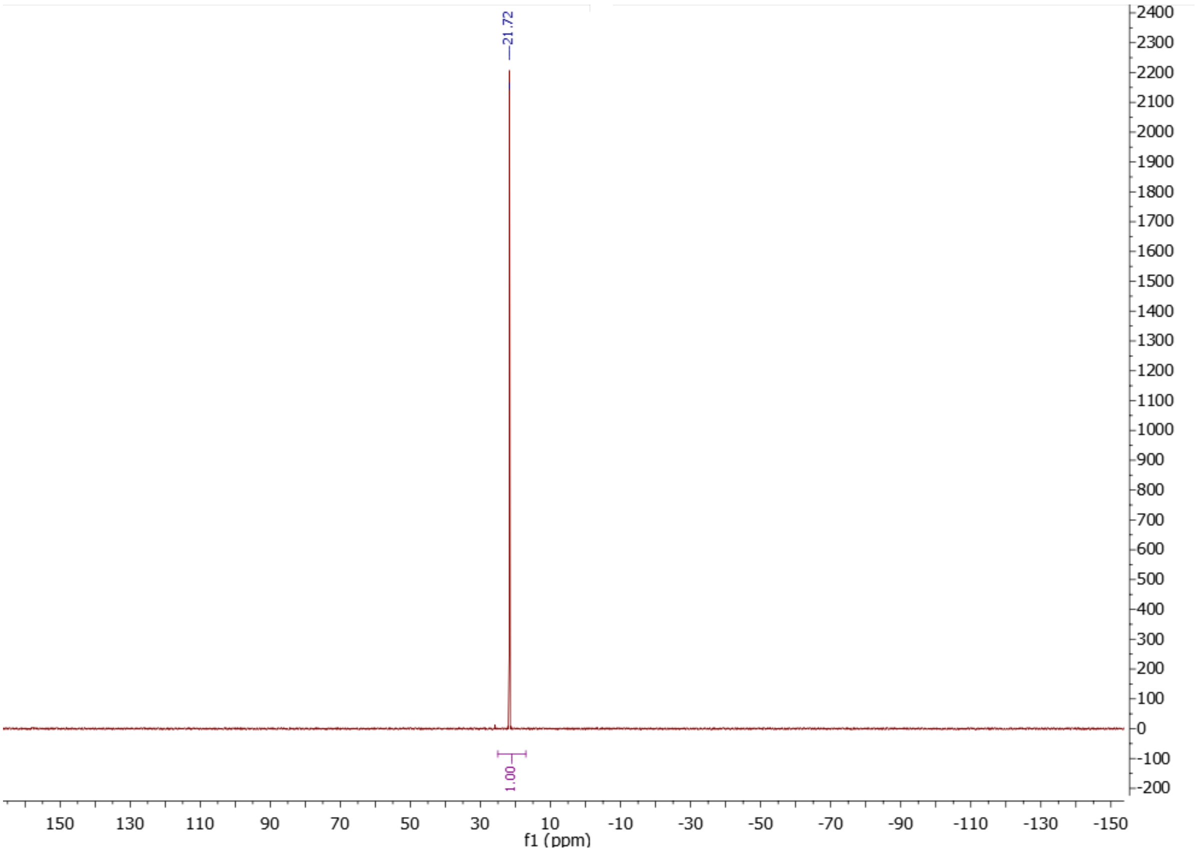

Compound **P10**

^{1}H NMR (400 MHz, $CDCl_3$):

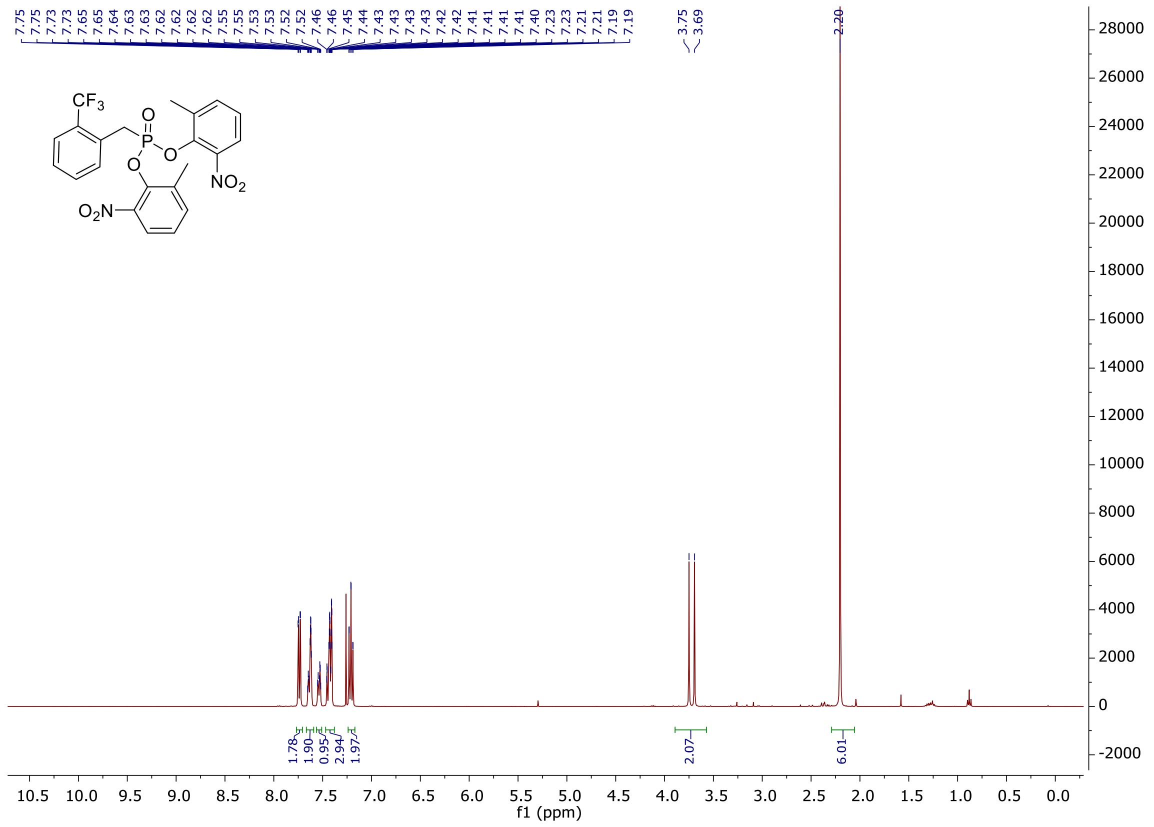

^{13}C NMR (101 MHz, $CDCl_3$):

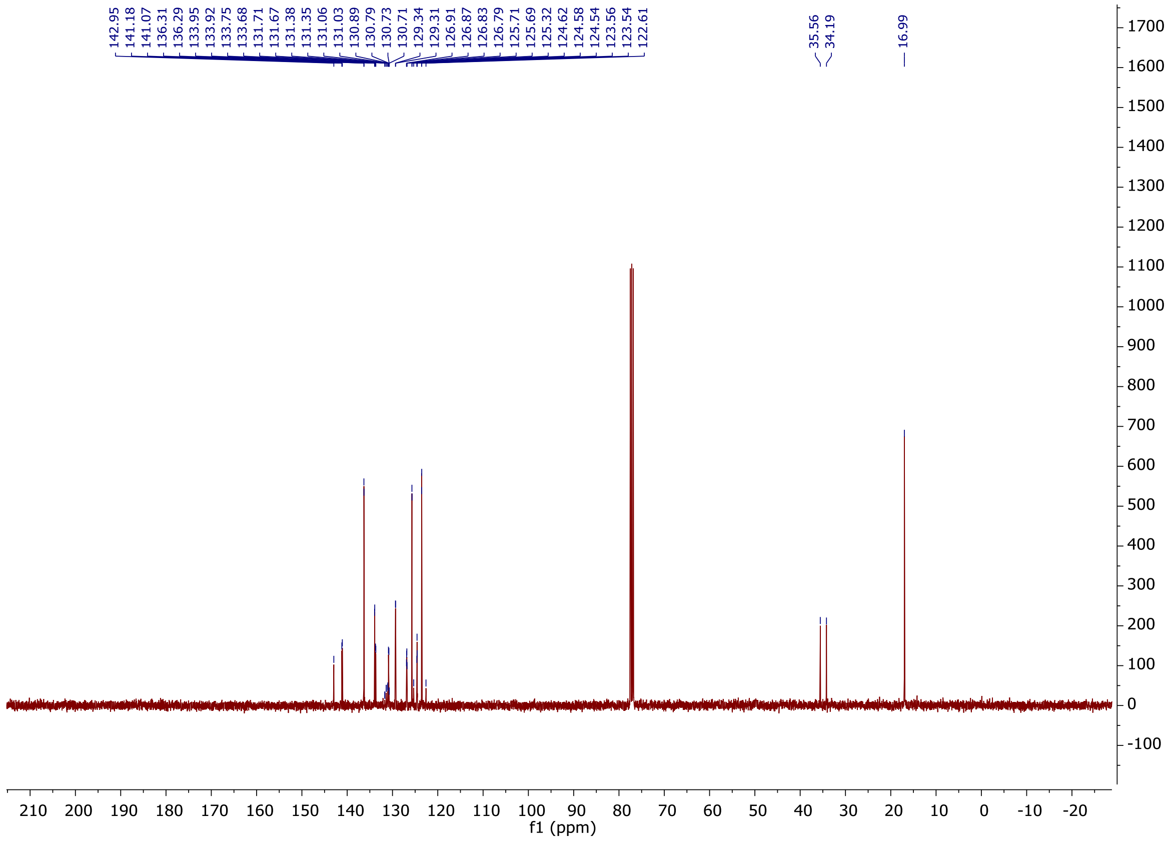

^{19}F NMR (377 MHz, CDCl$_3$)::

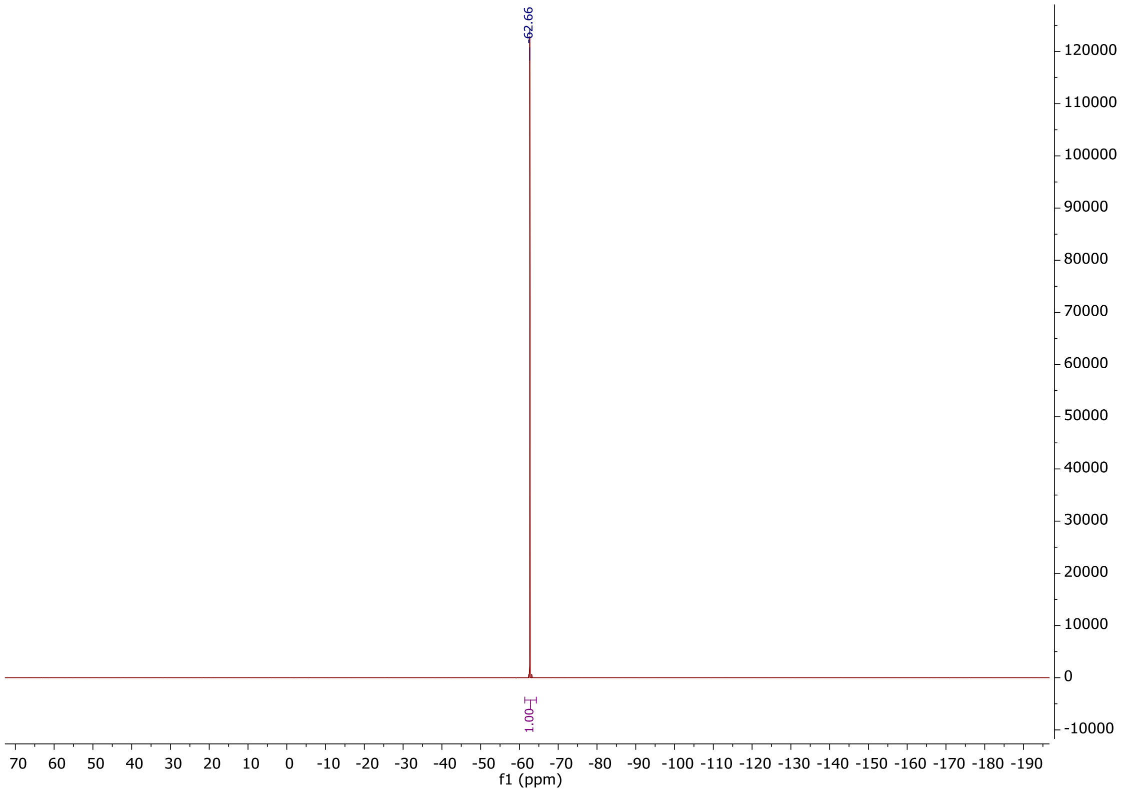

^{31}P NMR (162 MHz, CDCl$_3$):

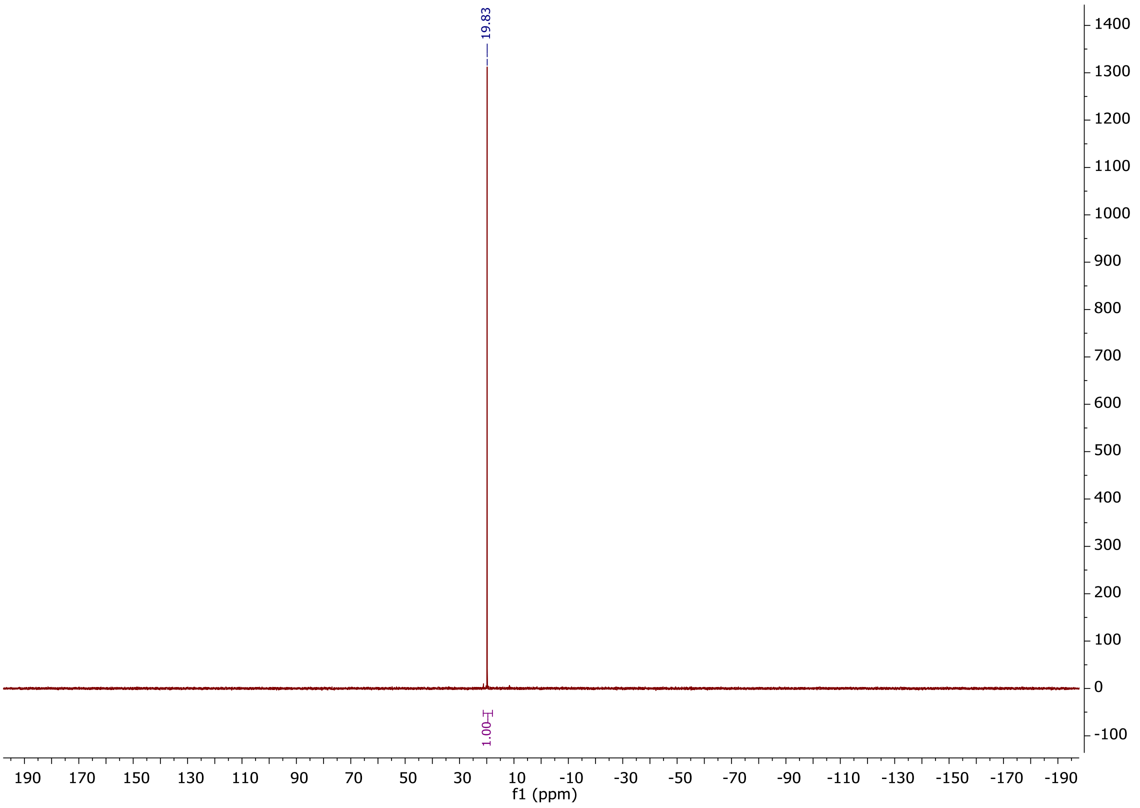

Compound **P11**

^{1}H NMR (400 MHz, $CDCl_3$):

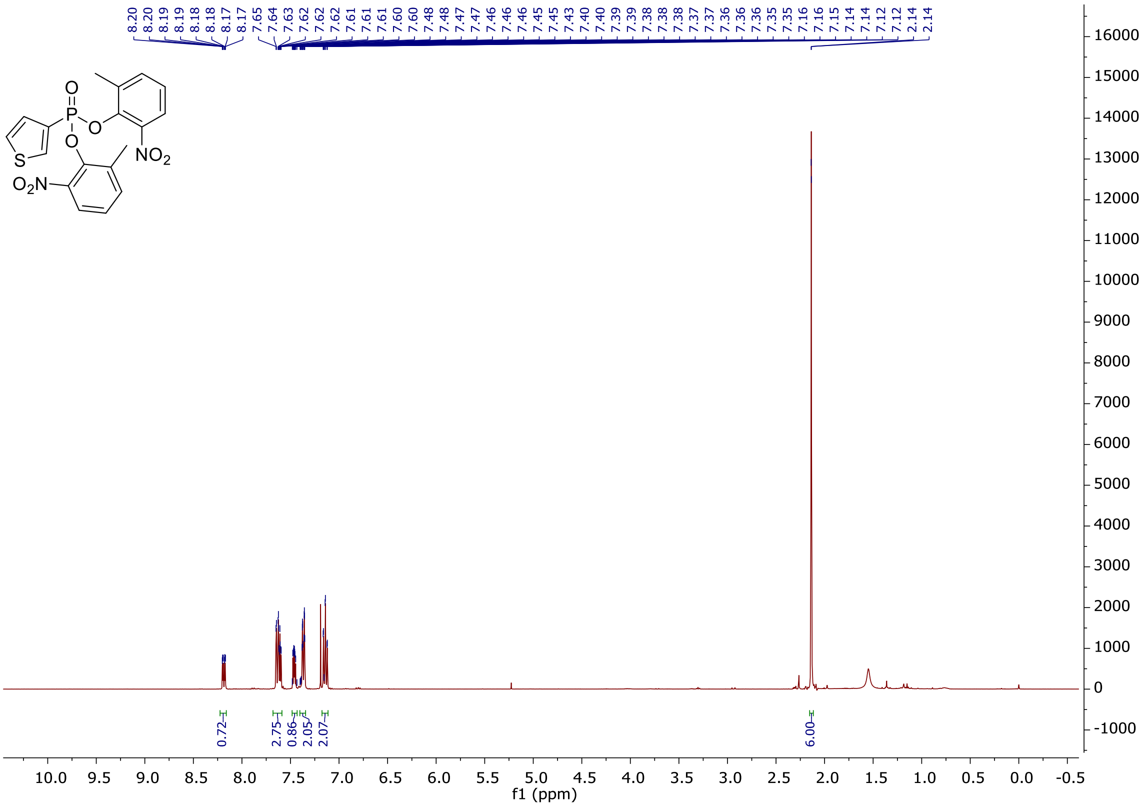

^{13}C NMR (101 MHz, $CDCl_3$):

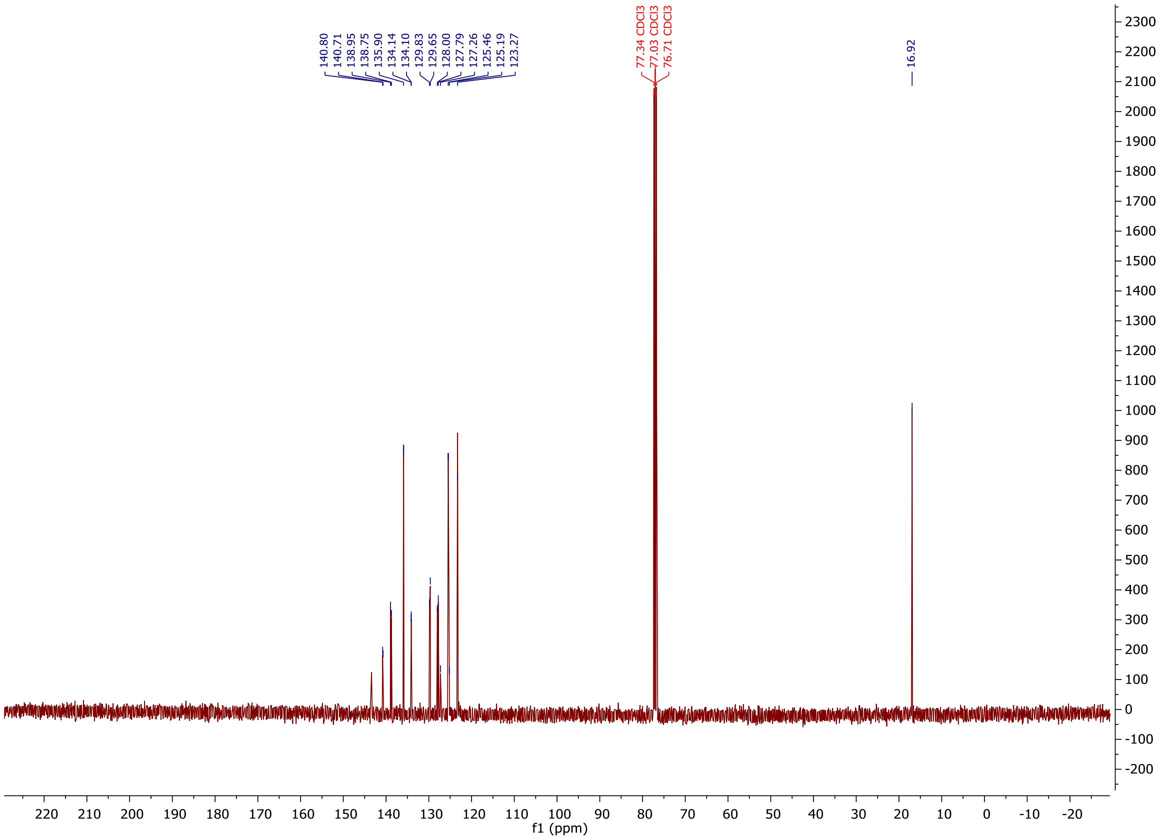

^{31}P NMR (162 MHz, $CDCl_3$):

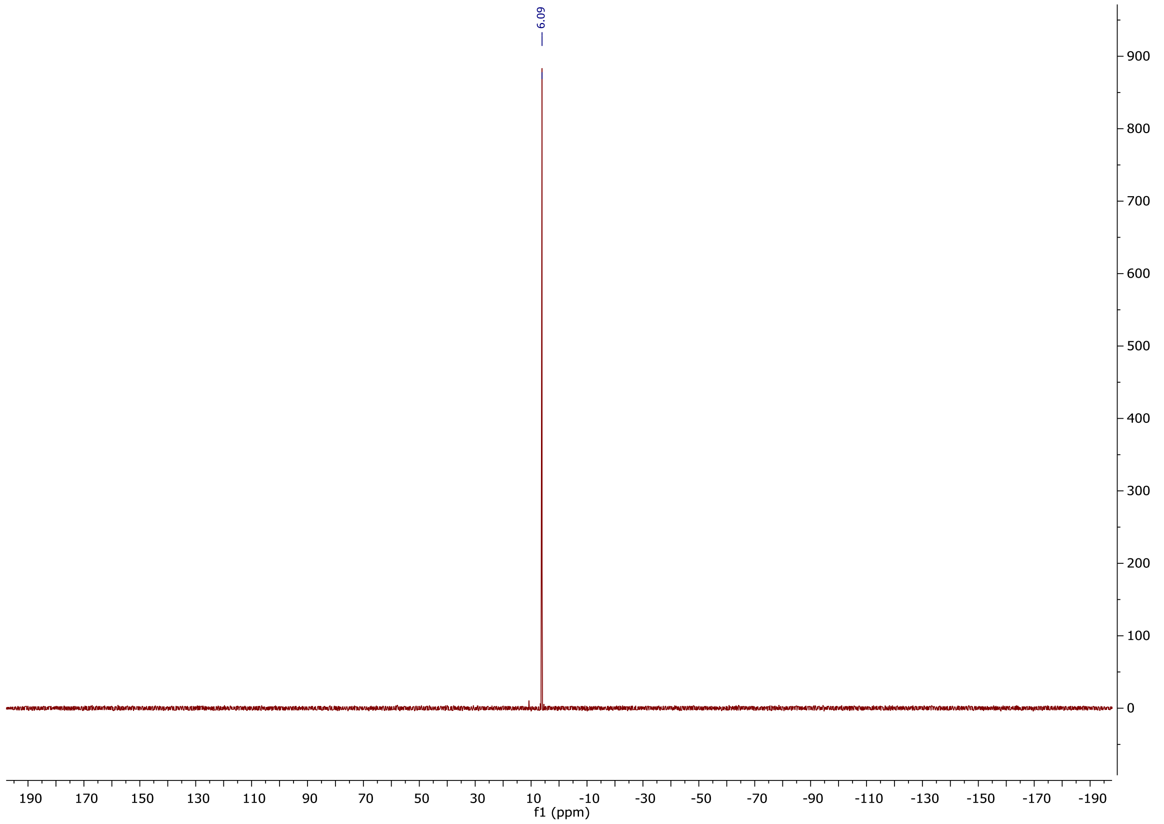

Compound **P12**

^{1}H NMR (400 MHz, $CDCl_3$):

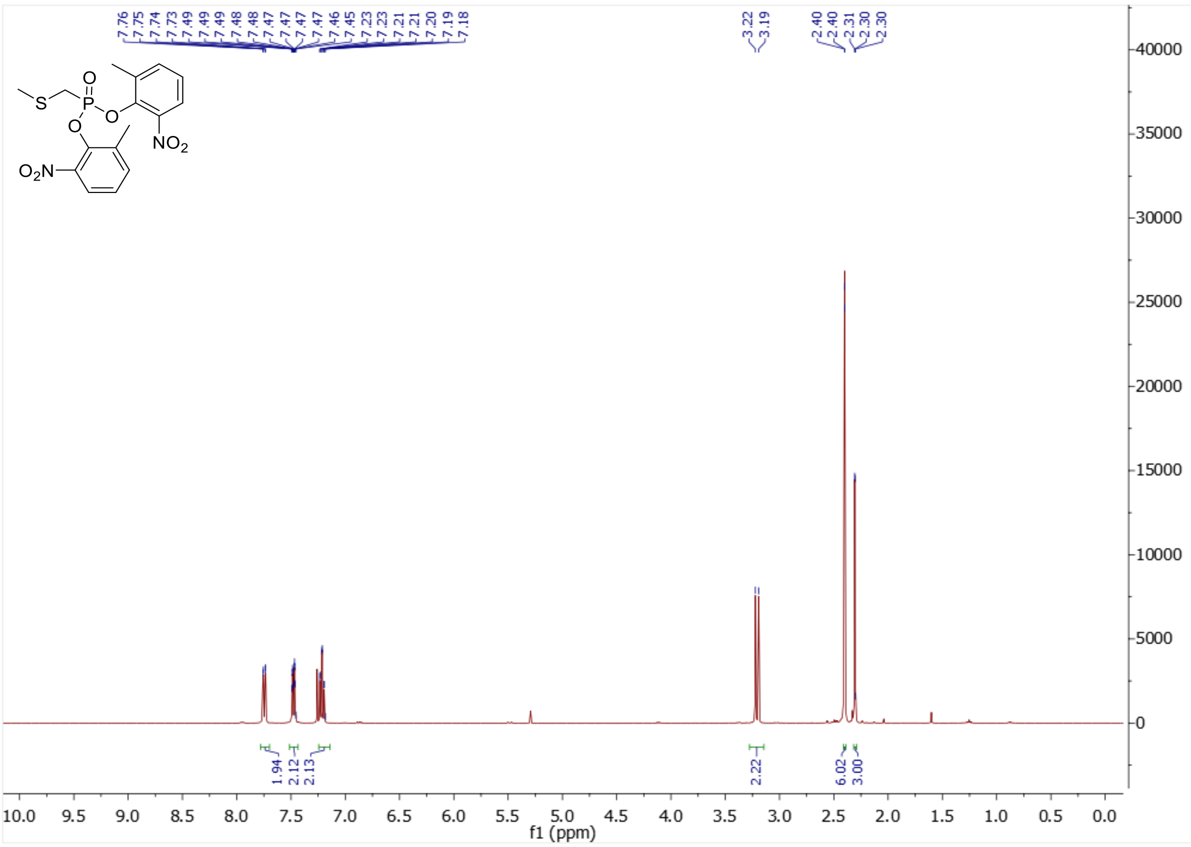

^{13}C NMR (101 MHz, $CDCl_3$):

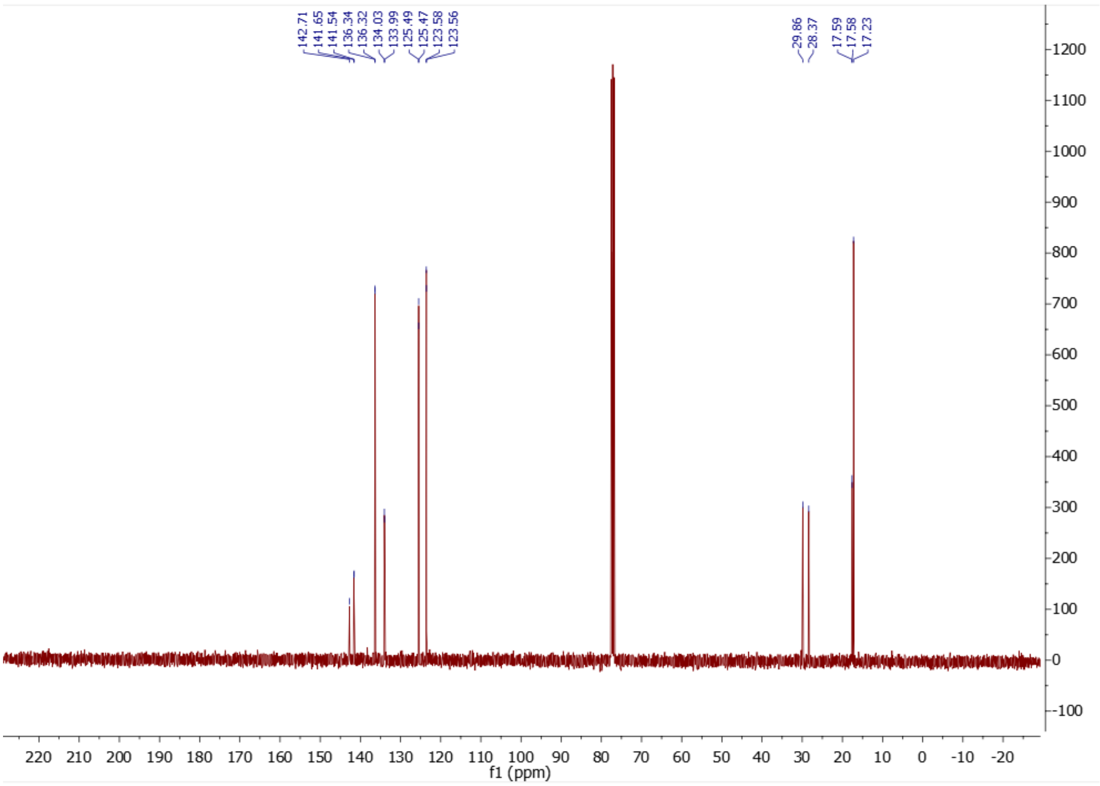

^{31}P NMR (162 MHz, $CDCl_3$):

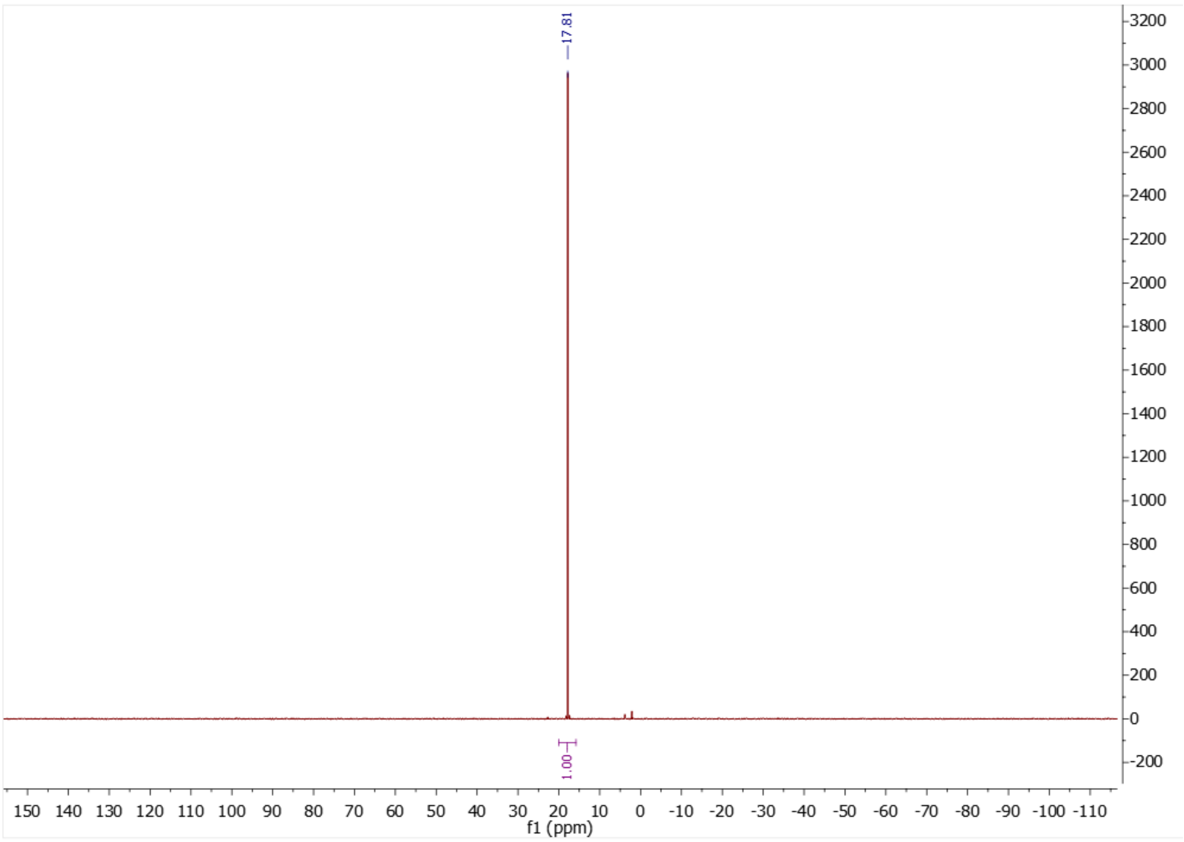

Compound **P13**

^{1}H NMR (400 MHz, $CDCl_3$):

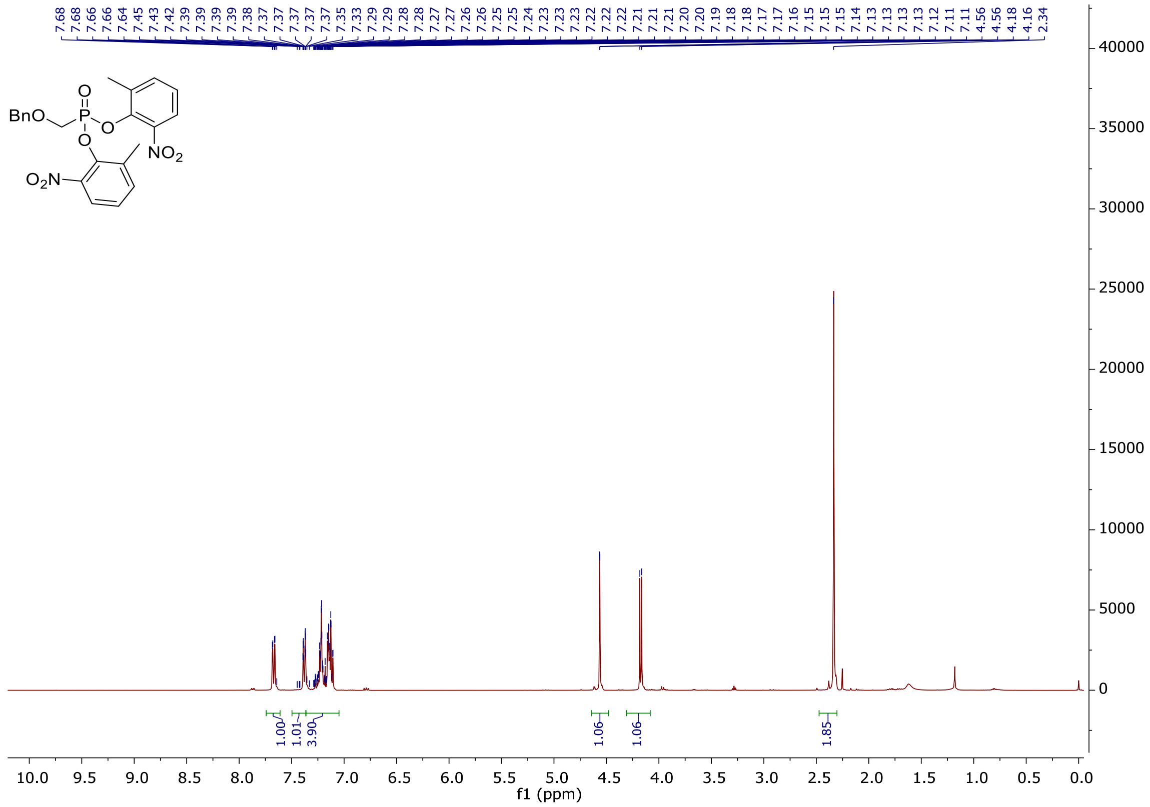

^{13}C NMR (101 MHz, $CDCl_3$):

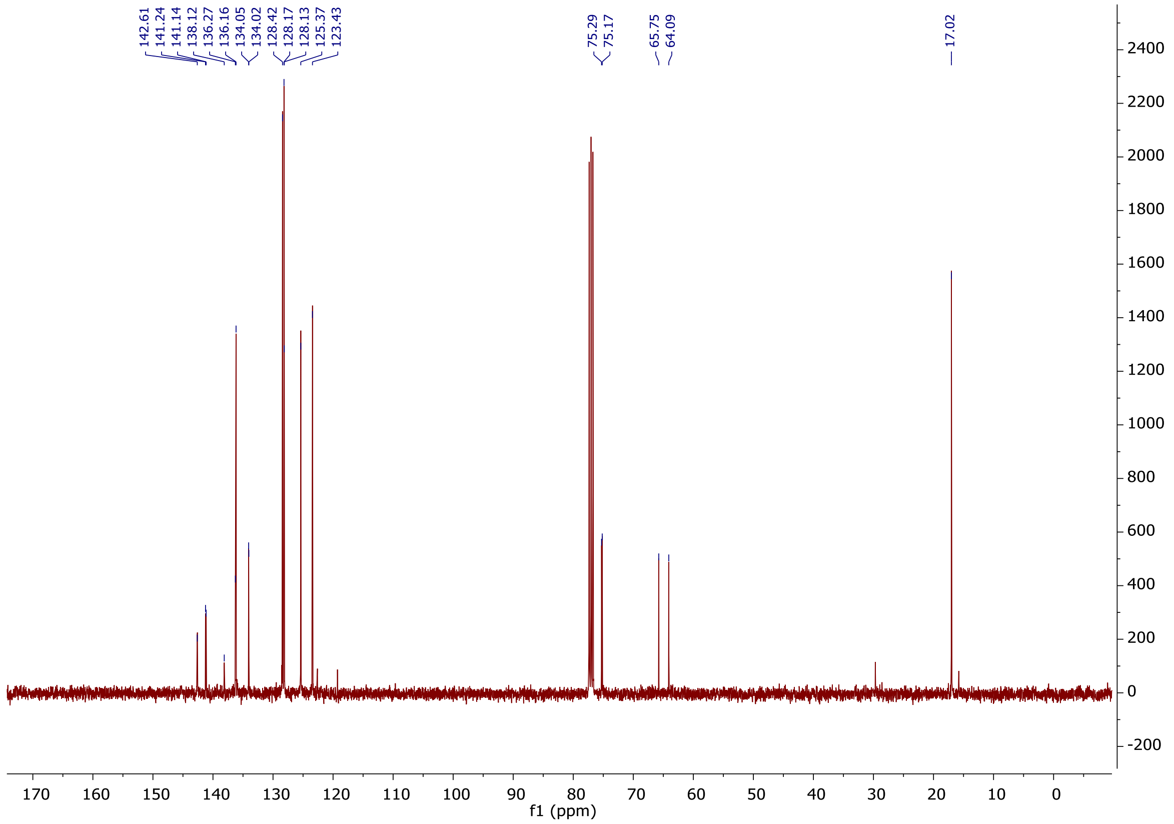

^{31}P NMR (162 MHz, $CDCl_3$):

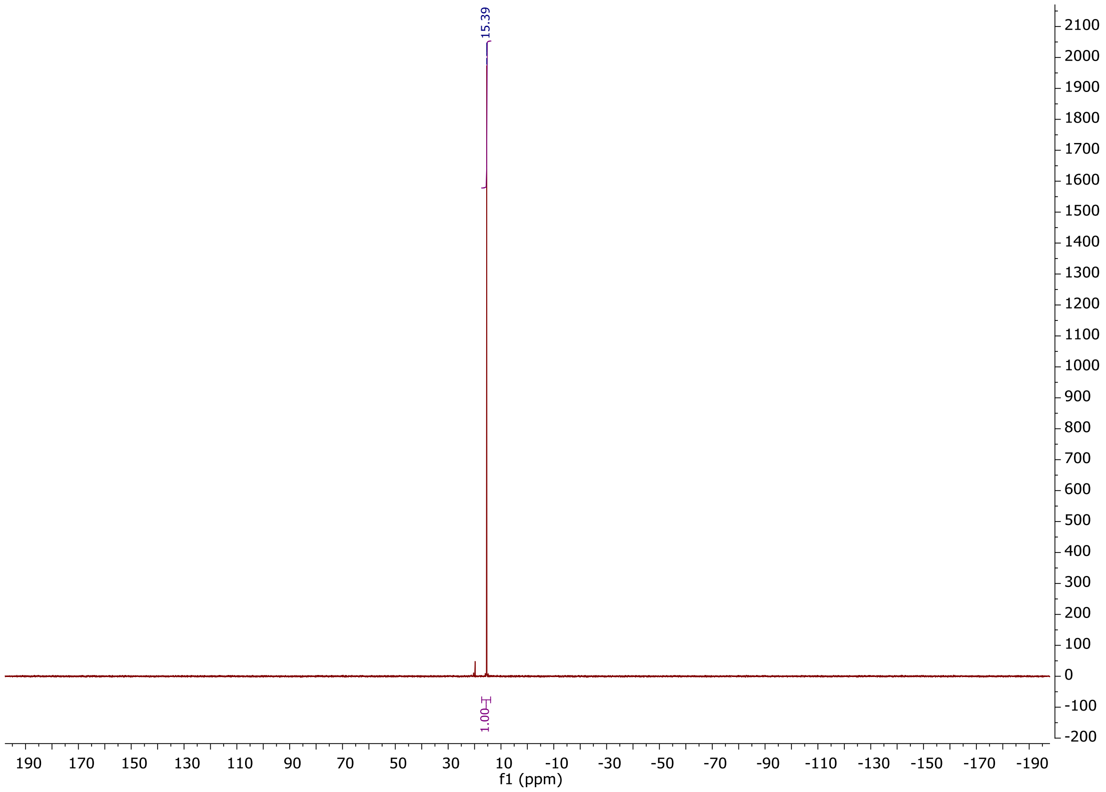

Compound **P14**

^{1}H NMR (400 MHz, $CDCl_3$):

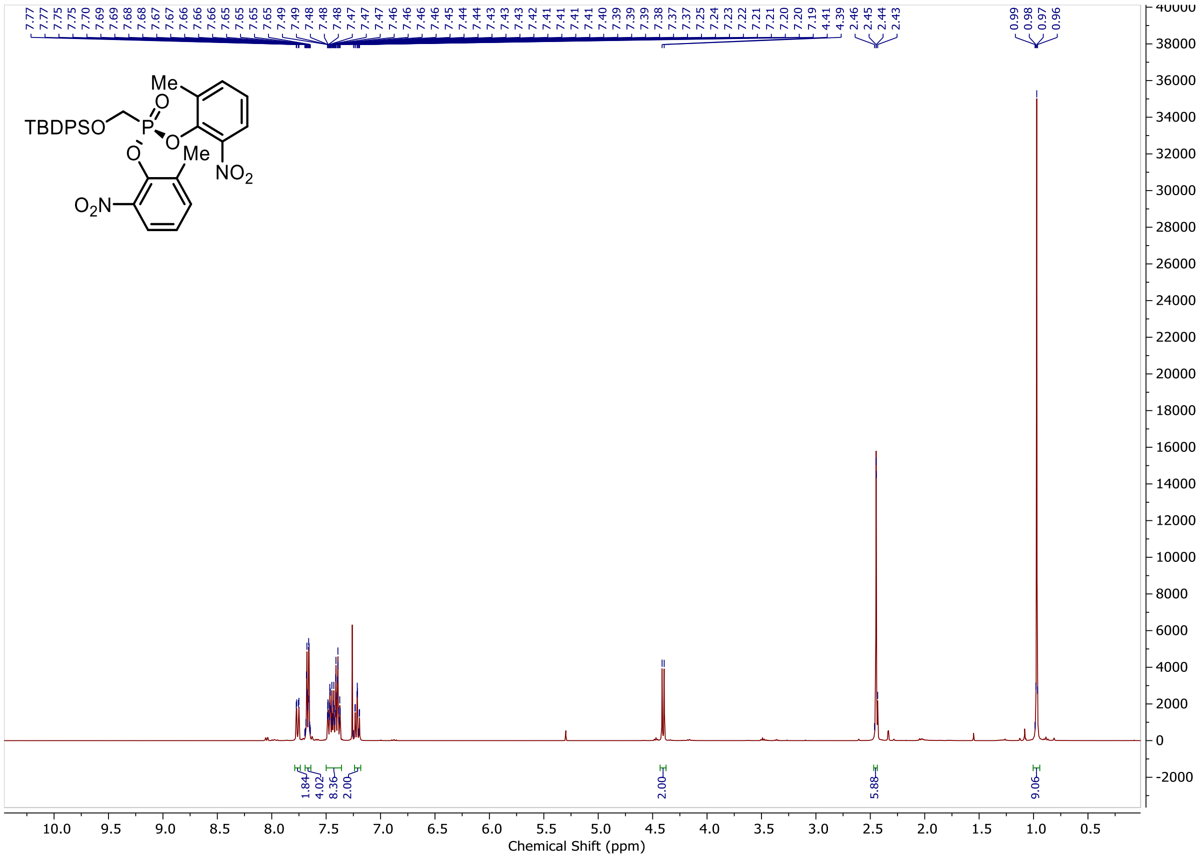

^{13}C NMR (101 MHz, $CDCl_3$):

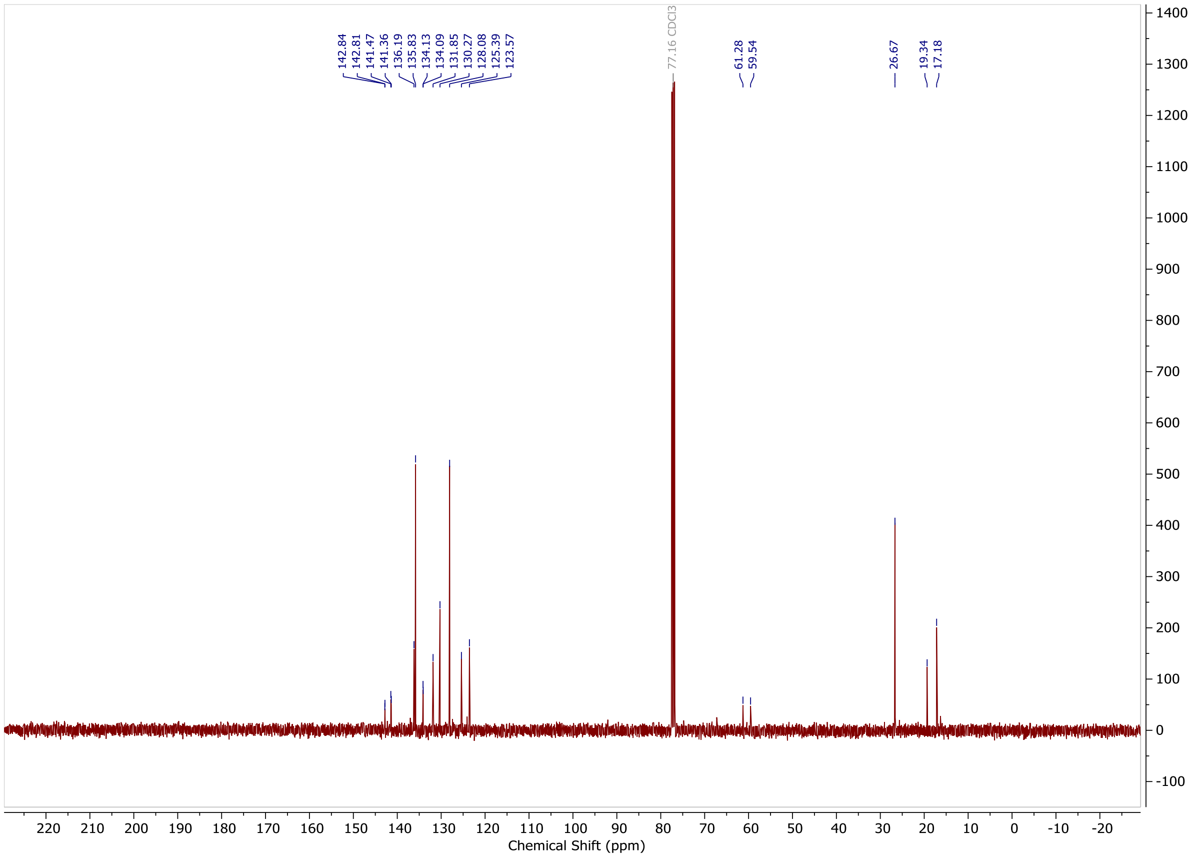

^{31}P NMR (162 MHz, $CDCl_3$):

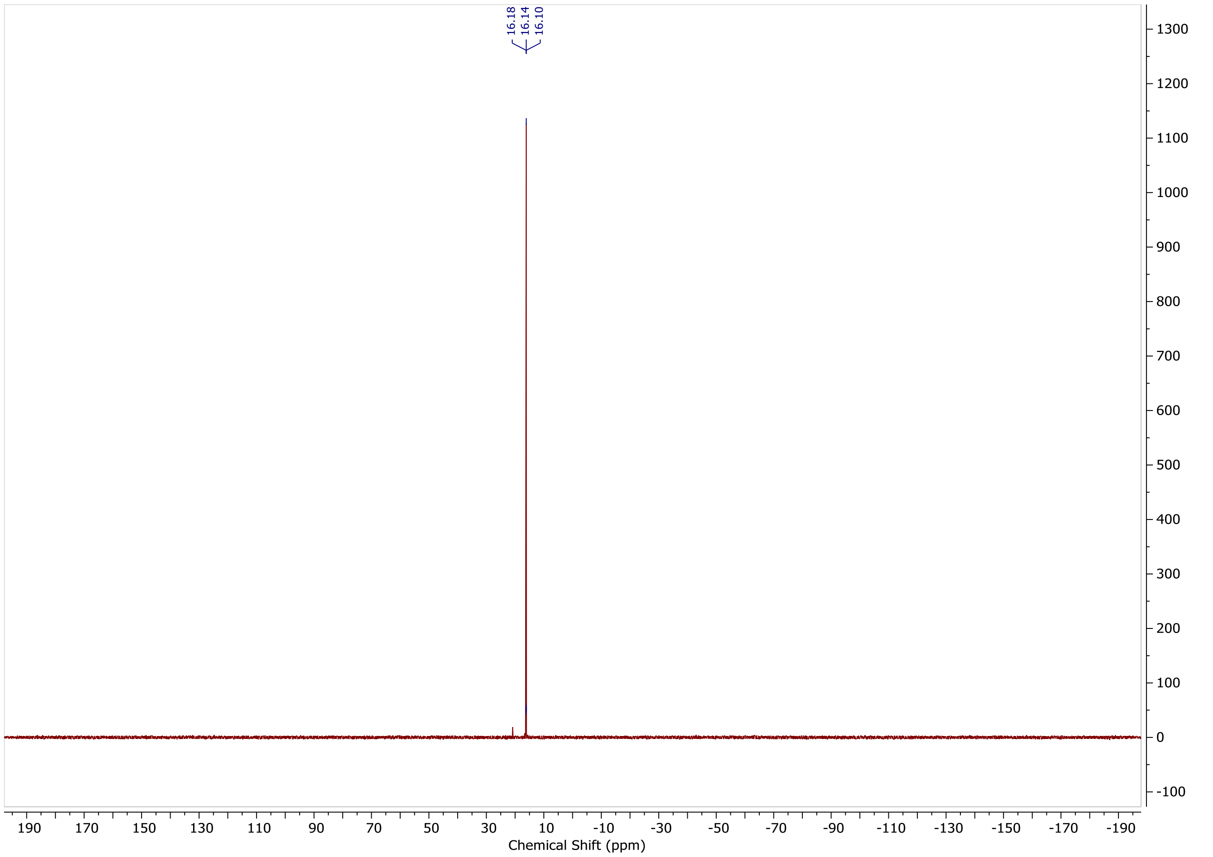

Compound **LGS1**

^{1}H NMR (400 MHz, $CDCl_3$):

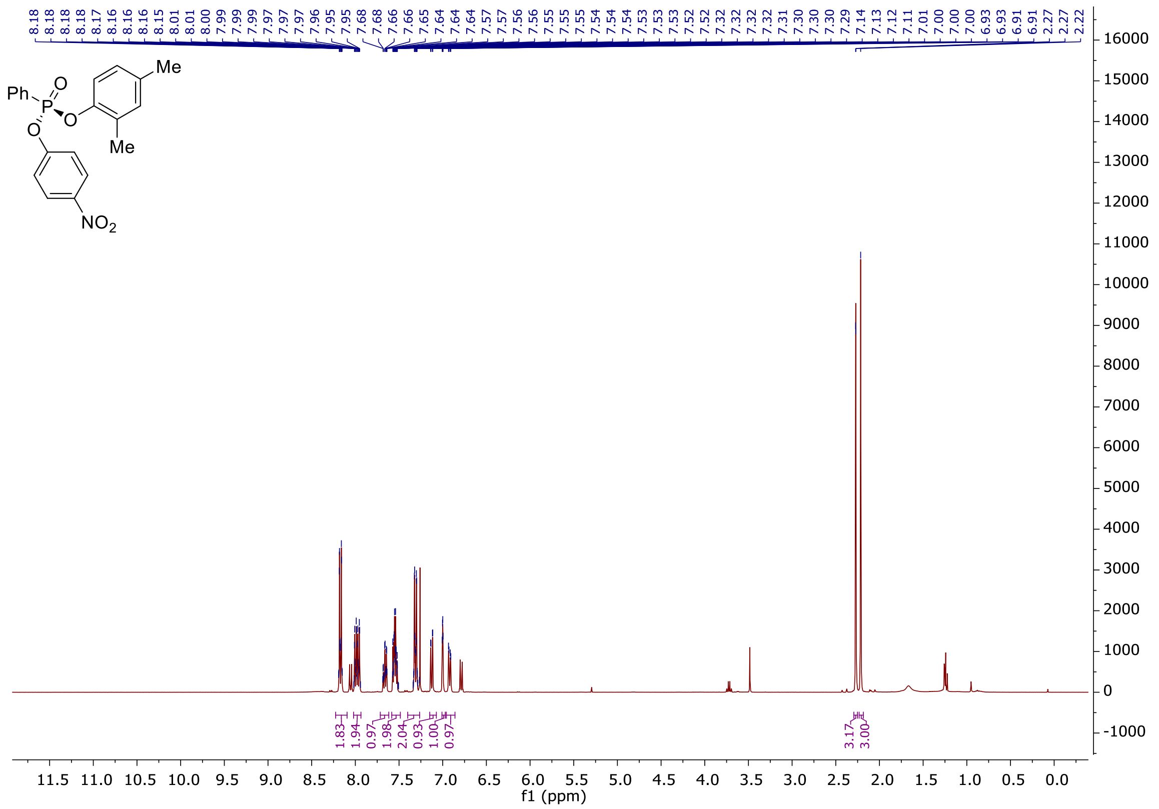

^{13}C NMR (101 MHz, $CDCl_3$):

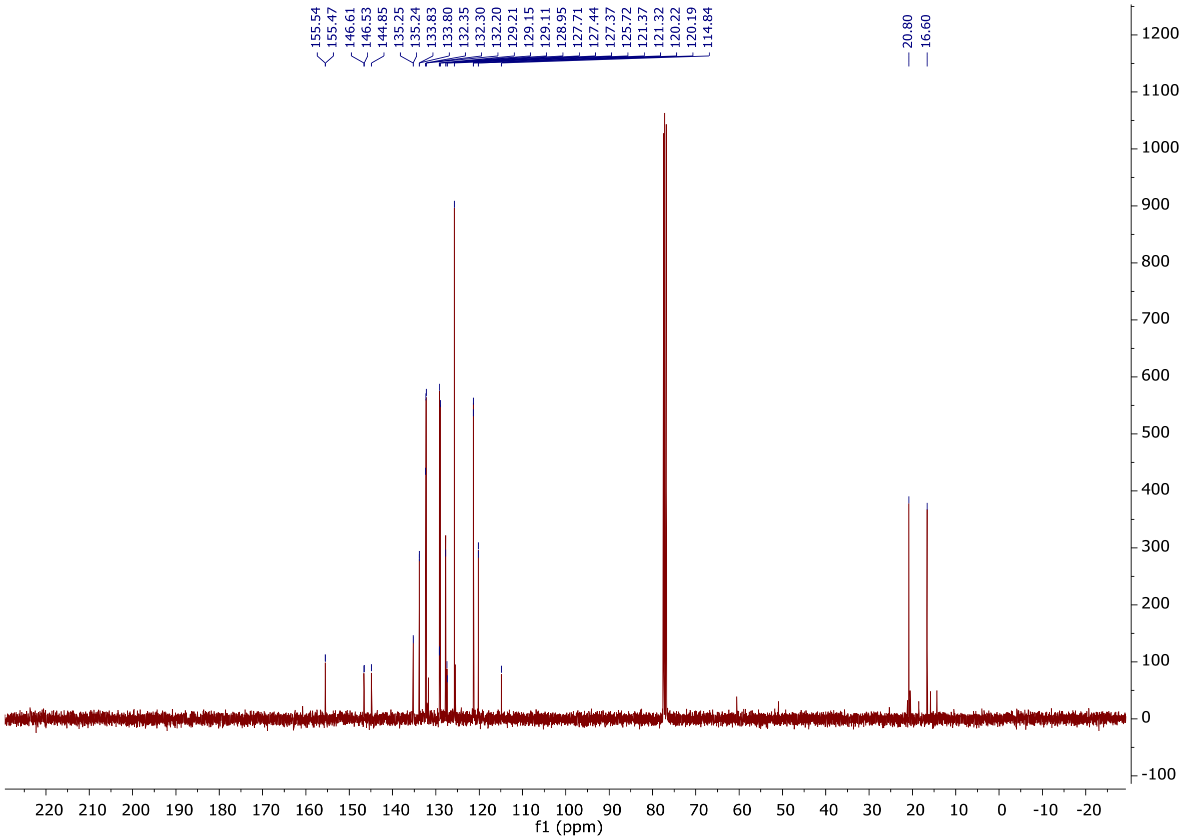

^{31}P NMR (162 MHz, CDCl$_3$):

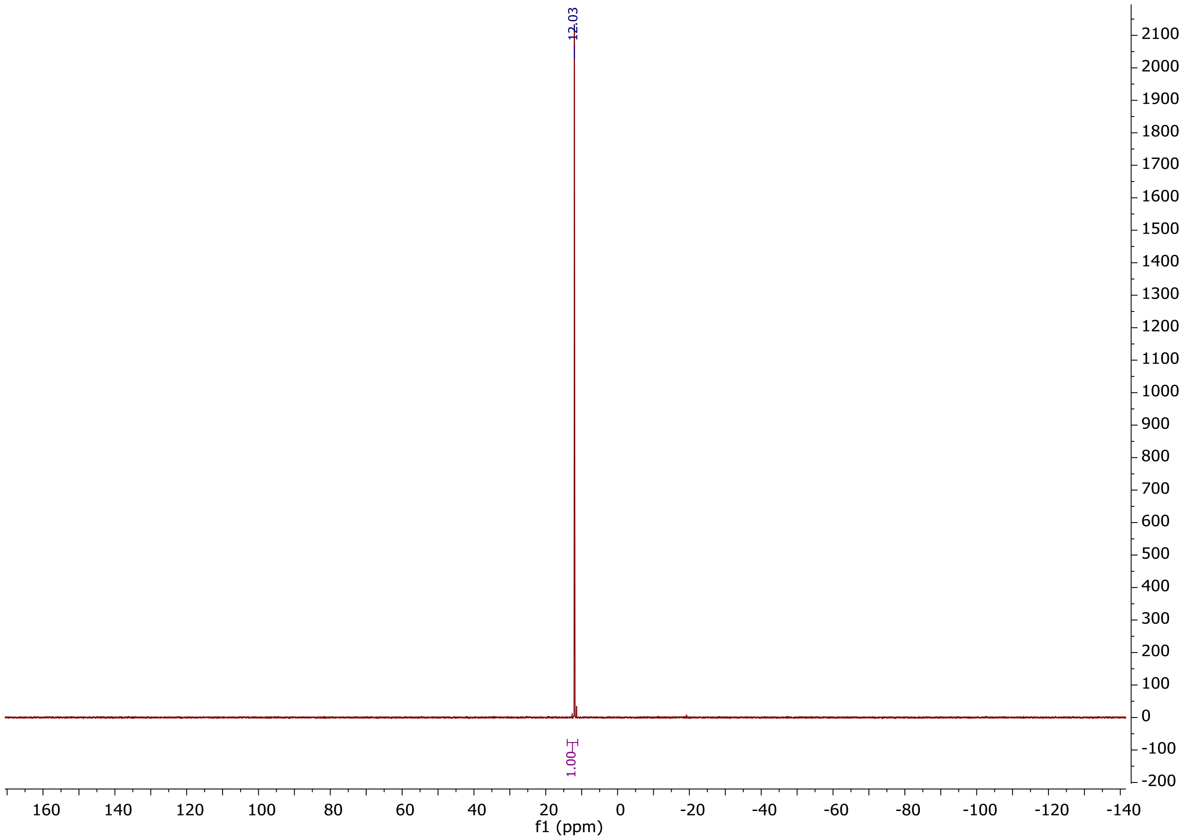

Compound **LG1**

^{1}H NMR (400 MHz, $CDCl_3$):

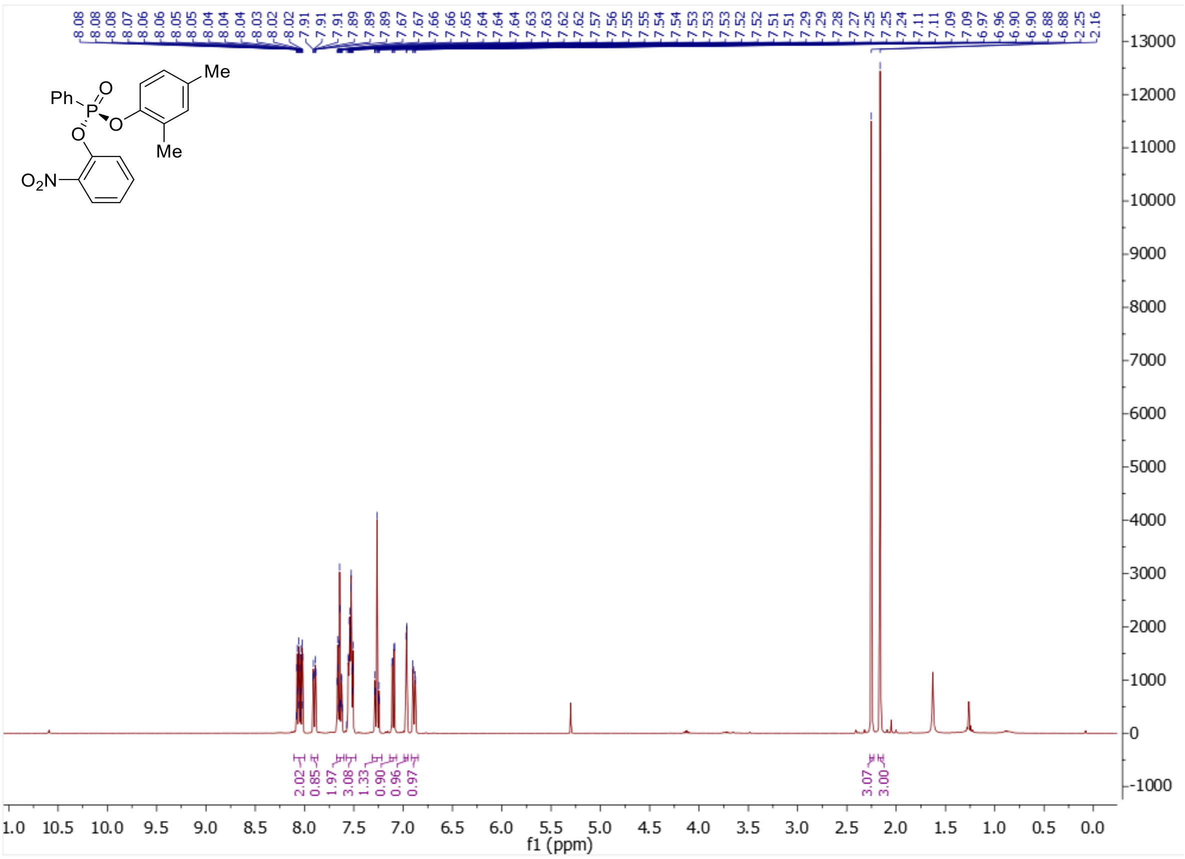

^{13}C NMR (101 MHz, $CDCl_3$):

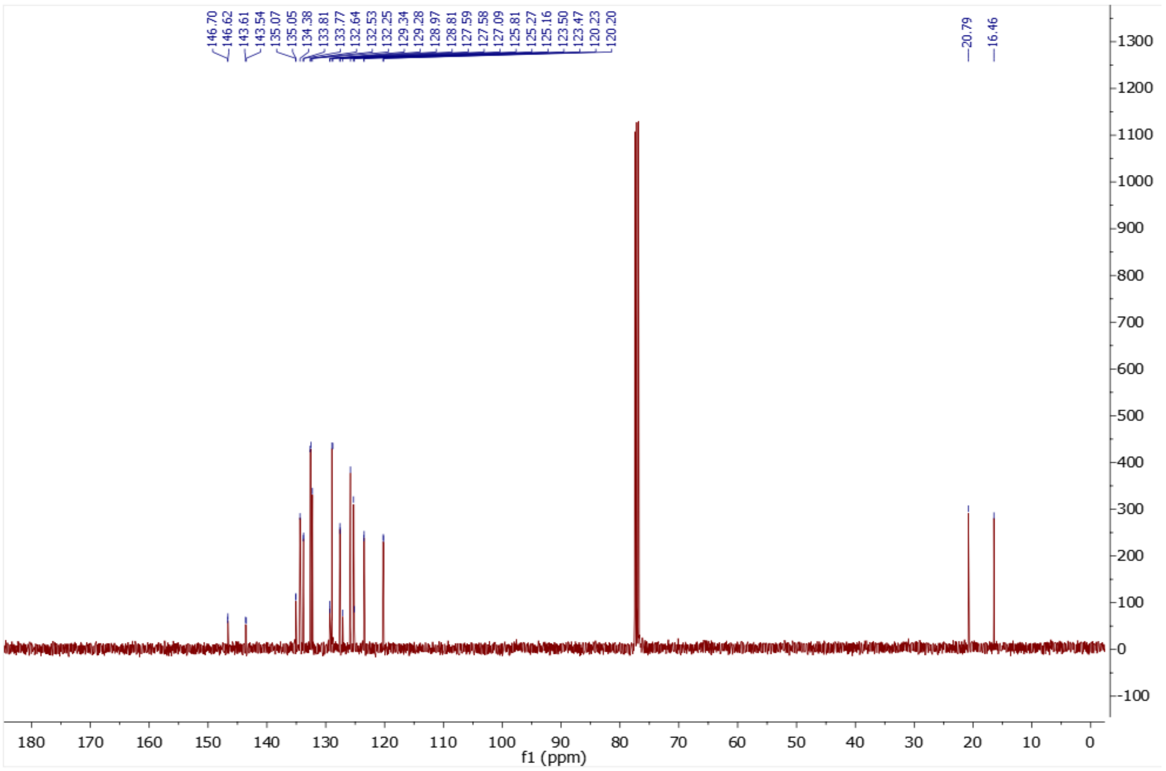

^{31}P NMR (162 MHz, $CDCl_3$):

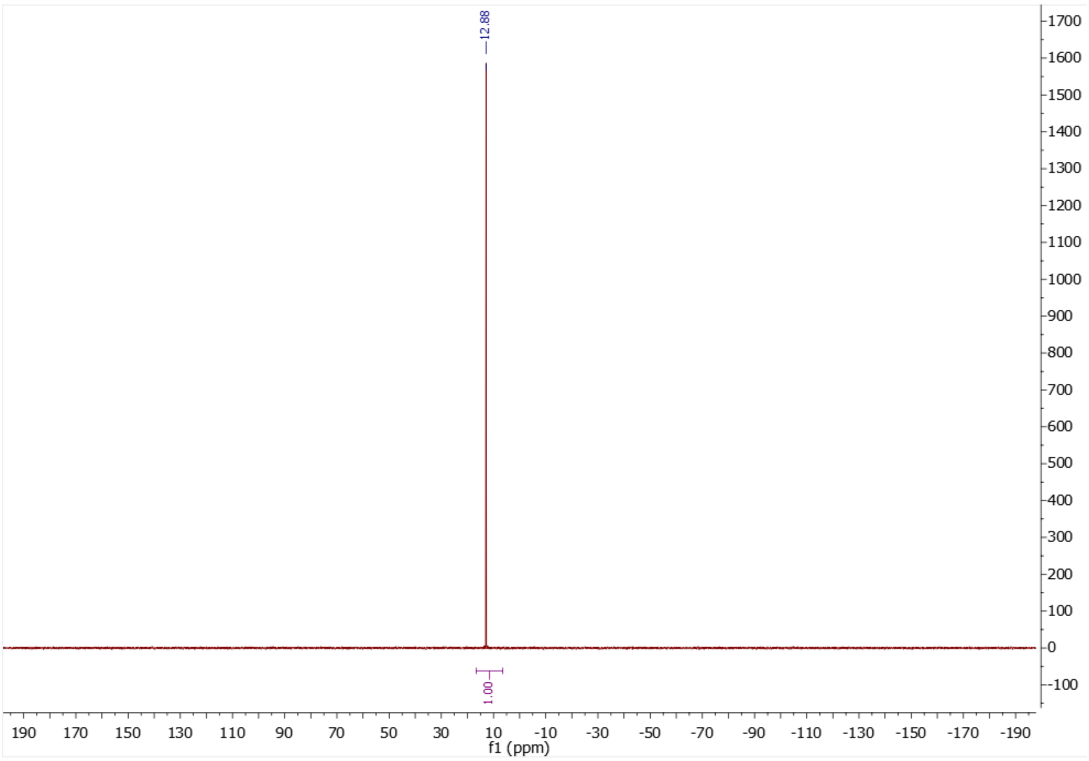

Compound **LG2**

^{1}H NMR (400 MHz, $CDCl_3$):

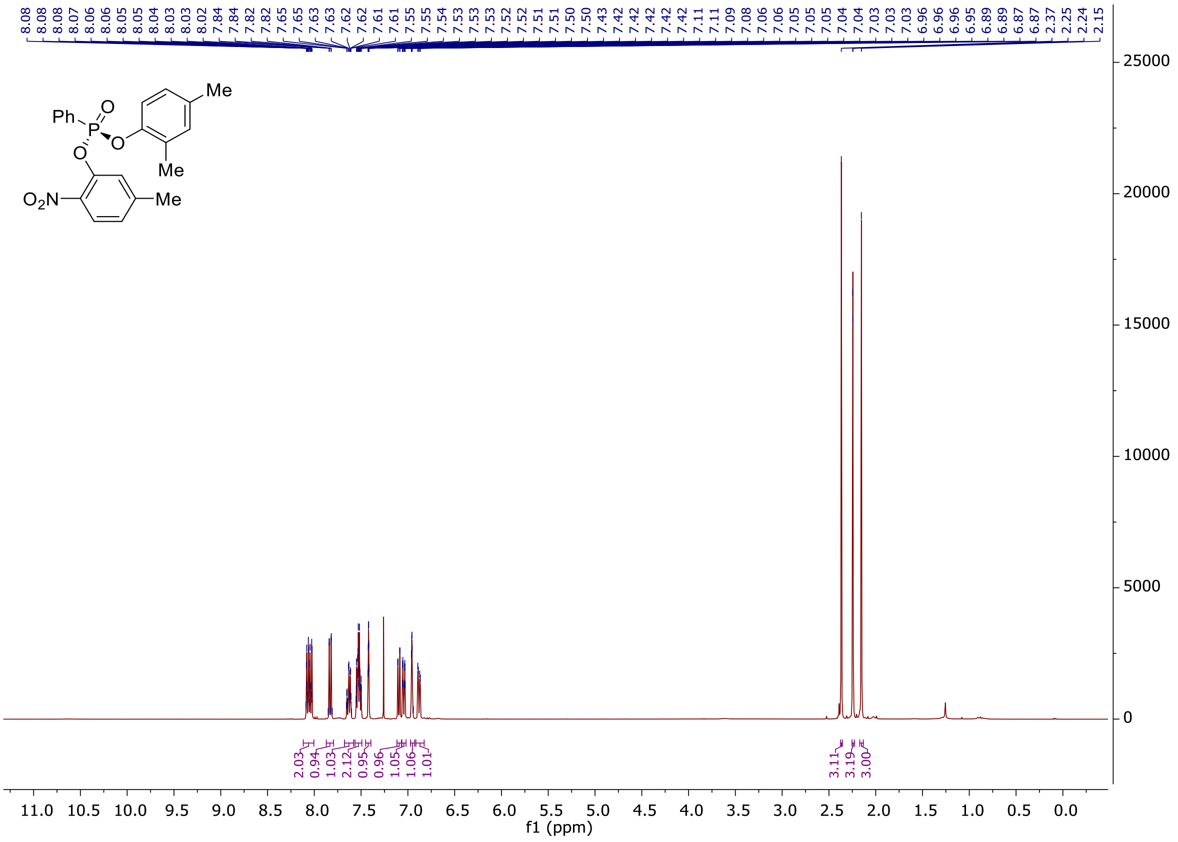

^{13}C NMR (101 MHz, $CDCl_3$):

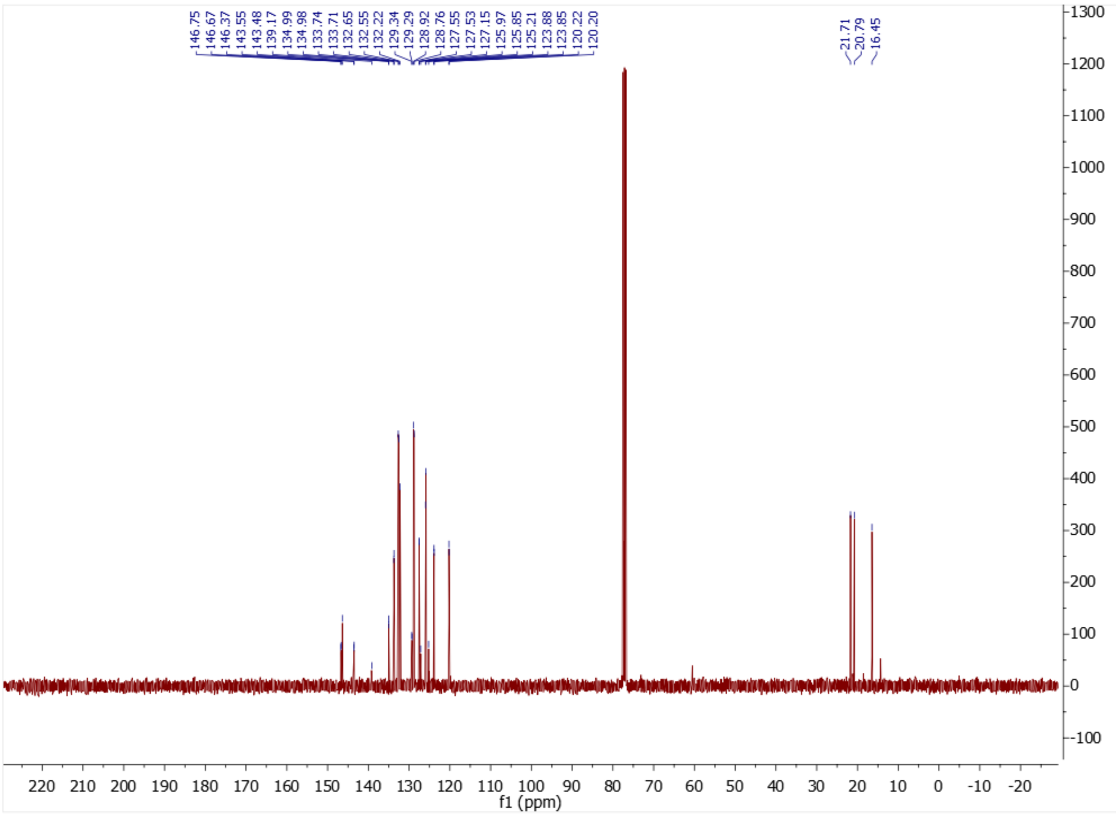

^{31}P NMR (162 MHz, CDCl$_3$):

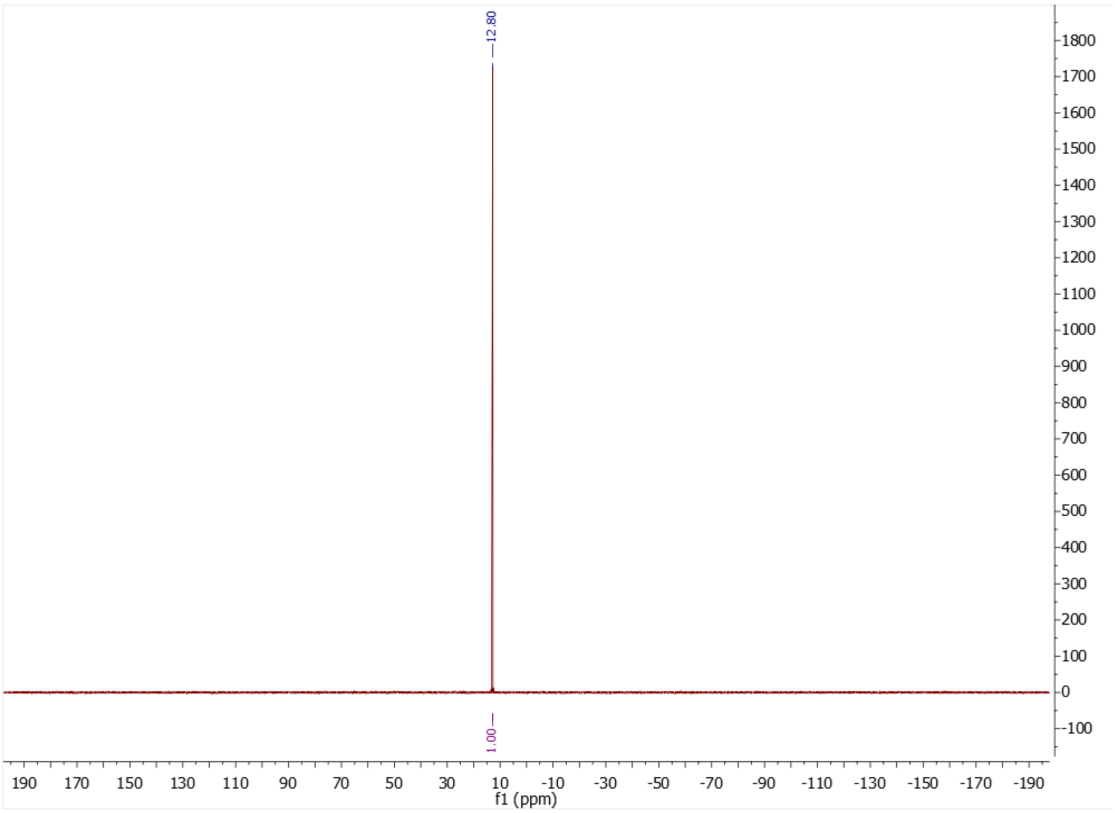

Compound **LG3**

^{1}H NMR (400 MHz, $CDCl_3$):

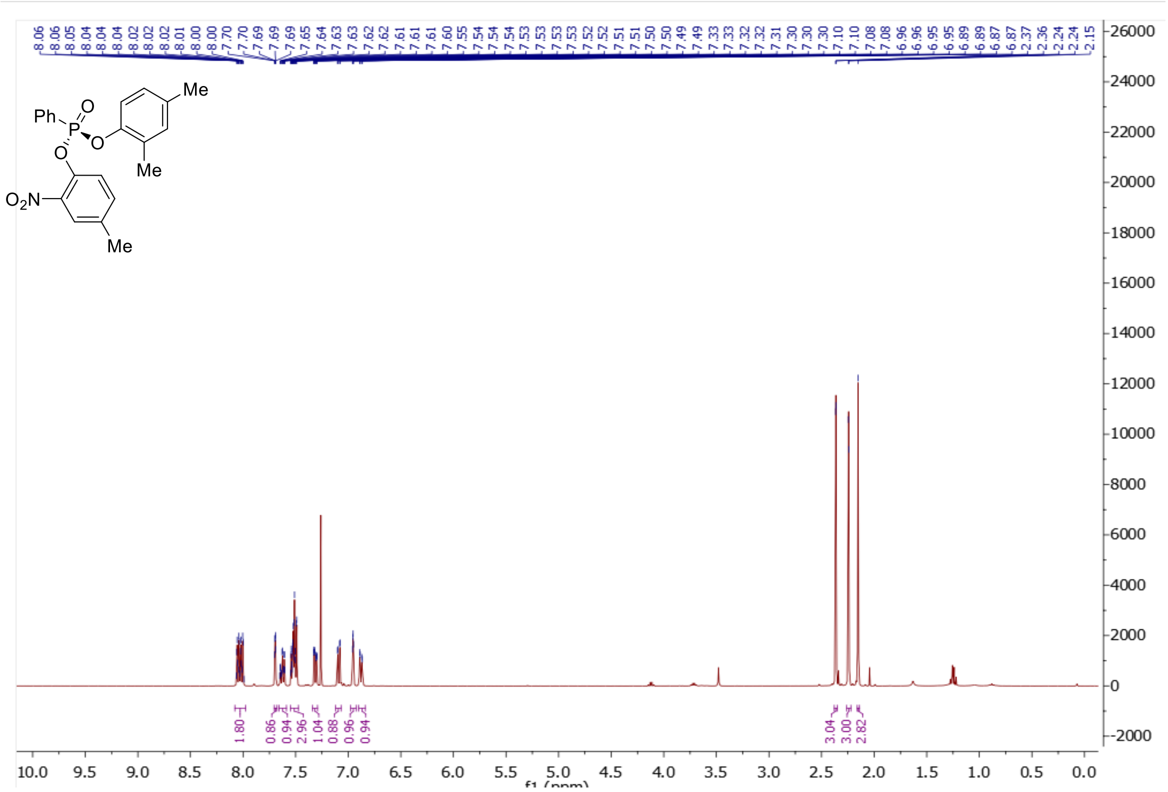

^{13}C NMR (101 MHz, $CDCl_3$):

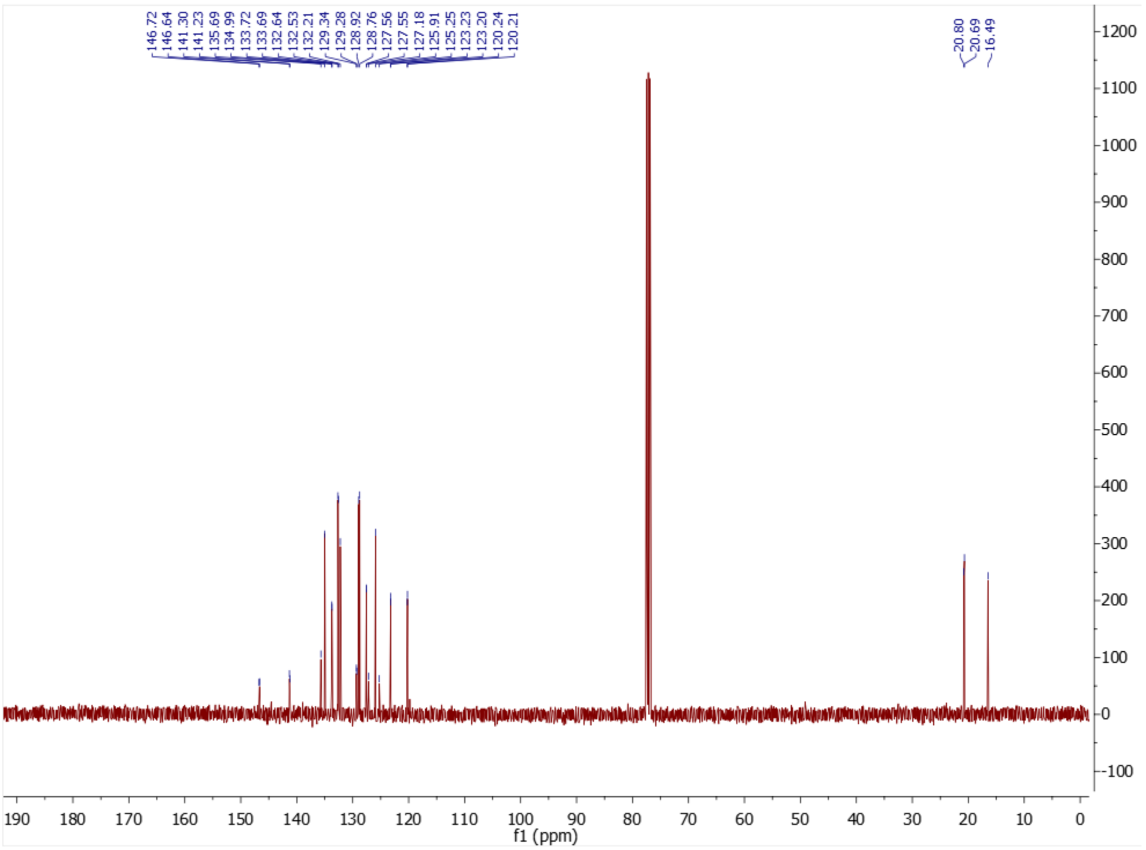

^{31}P NMR (162 MHz, $CDCl_3$):

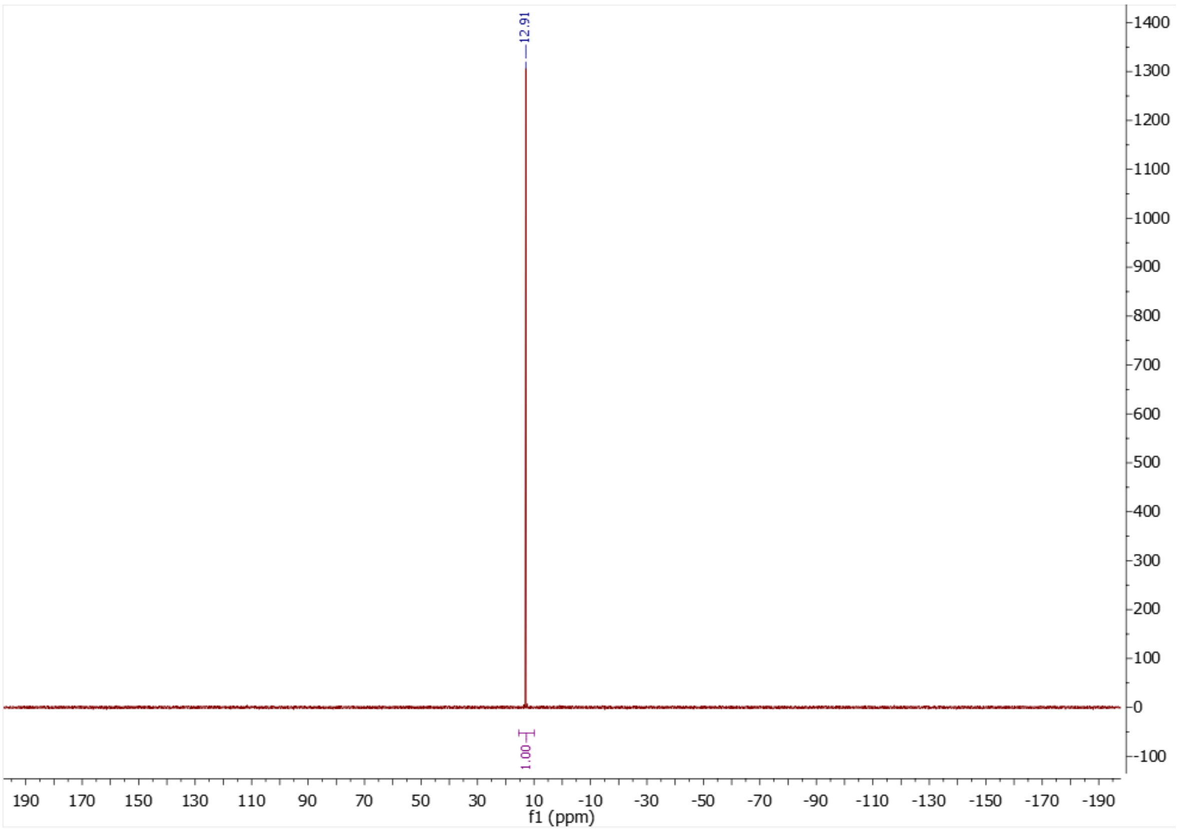

Compound **1**

^{1}H NMR (400 MHz, $CDCl_3$):

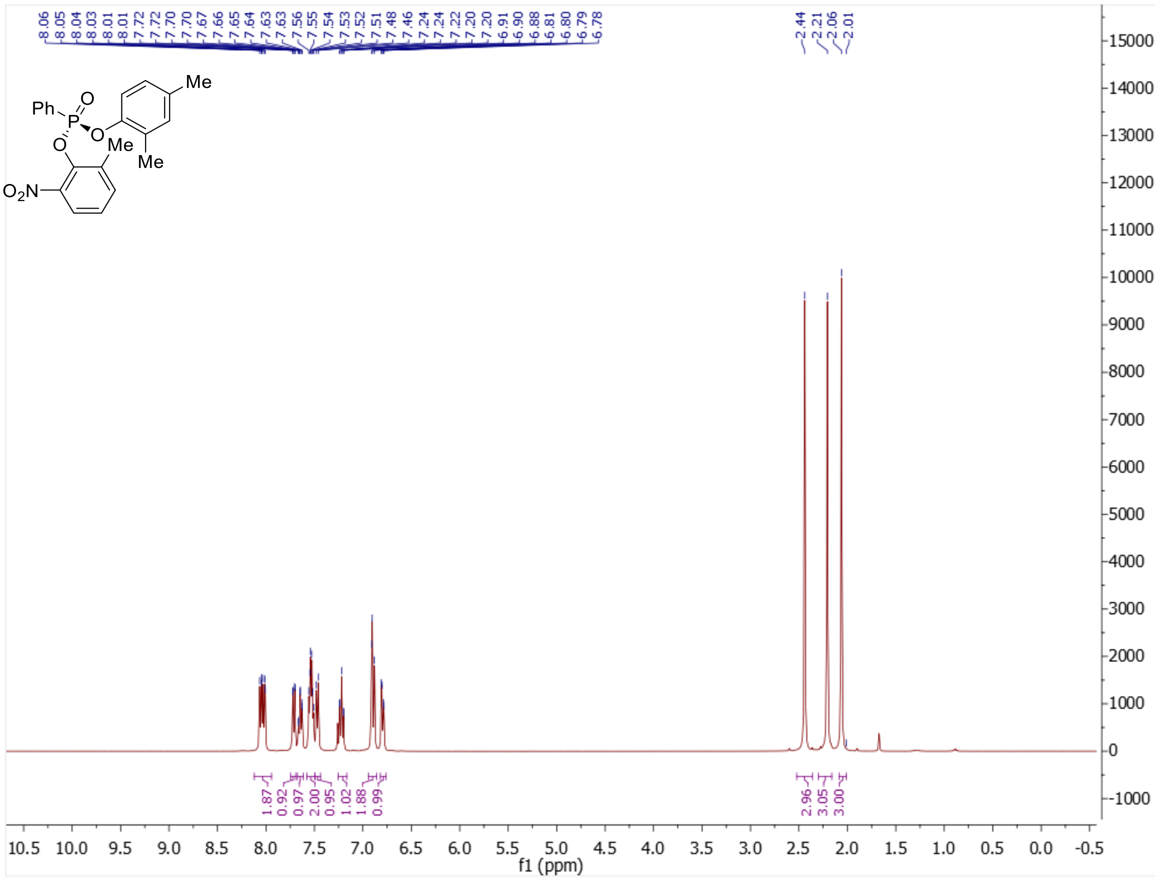

^{13}C NMR (101 MHz, $CDCl_3$):

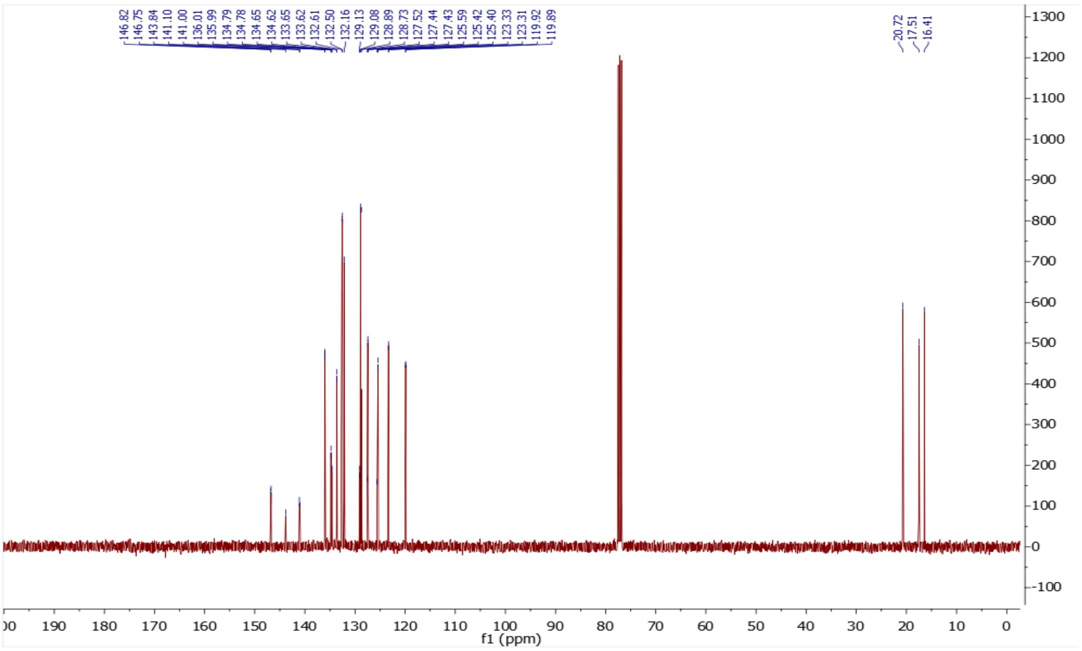

^{31}P NMR (162 MHz, $CDCl_3$):

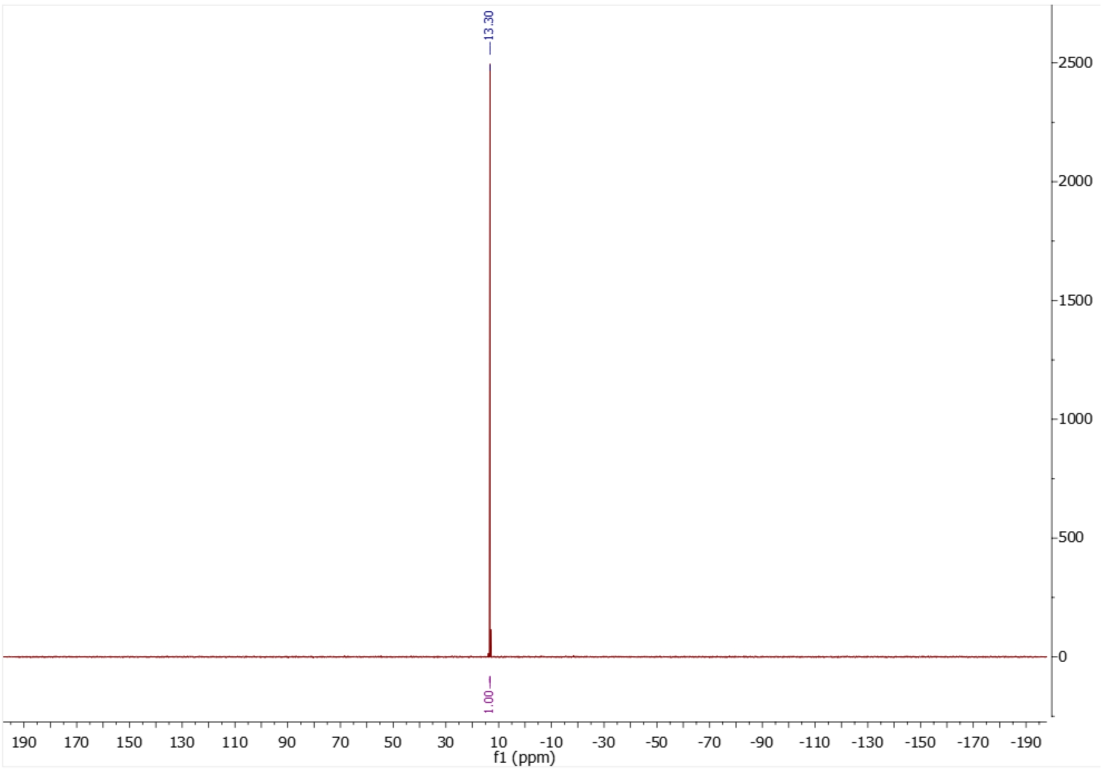

Compound **2**

^{1}H NMR (400 MHz, $CDCl_3$):

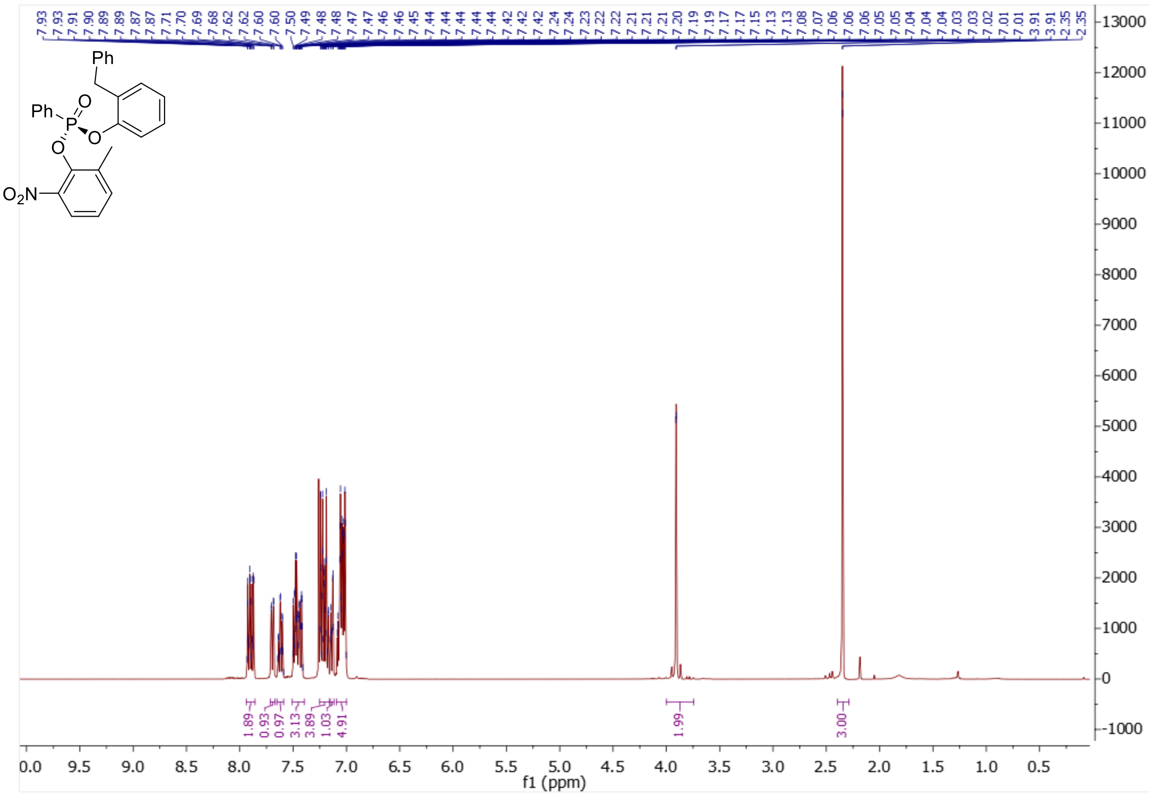

^{13}C NMR (101 MHz, $CDCl_3$):

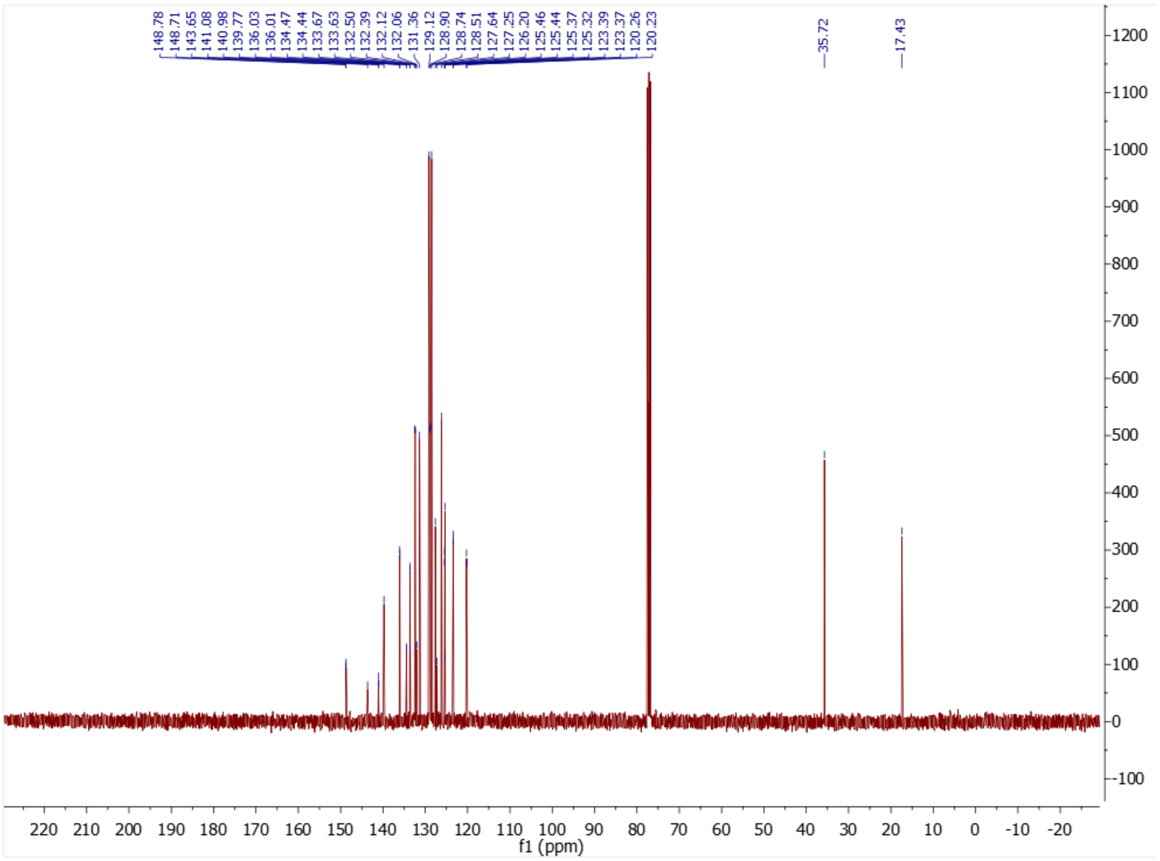

^{31}P NMR (162 MHz, $CDCl_3$):

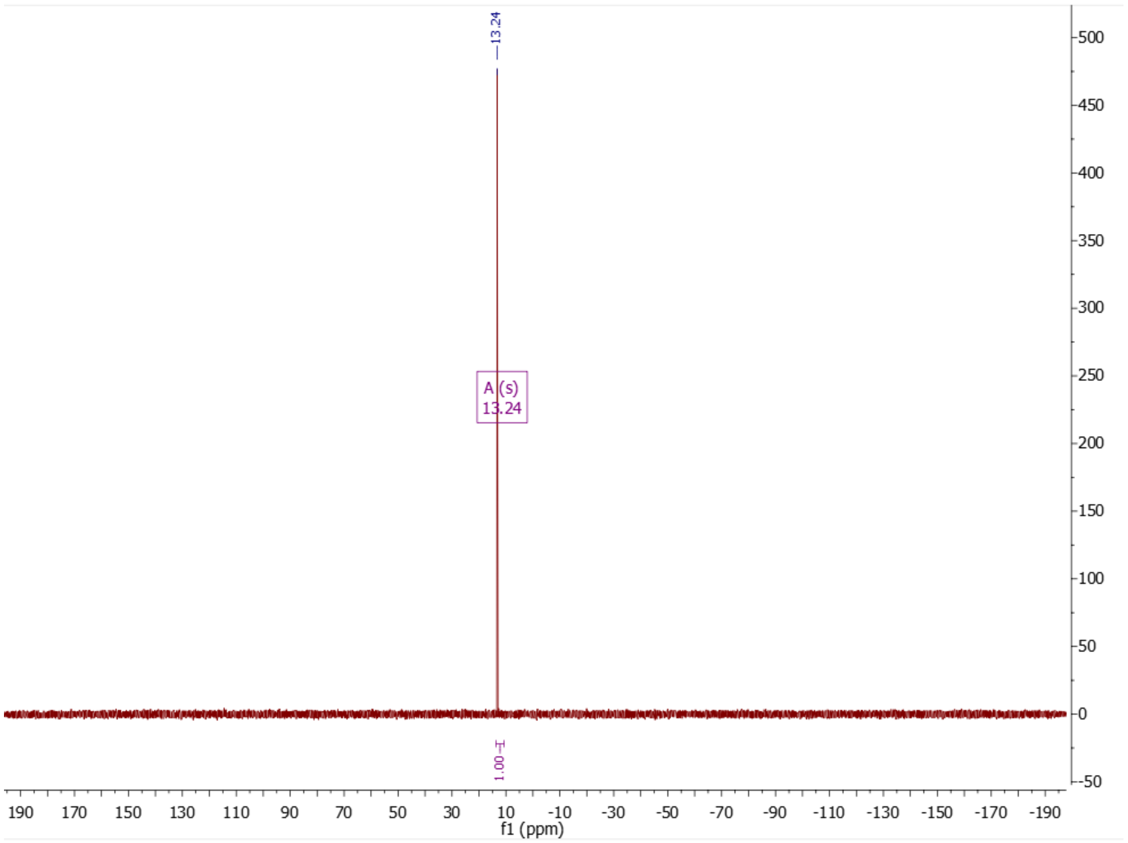

Compound **3**

^{11}H NMR (400 MHz, $CDCl_3$):

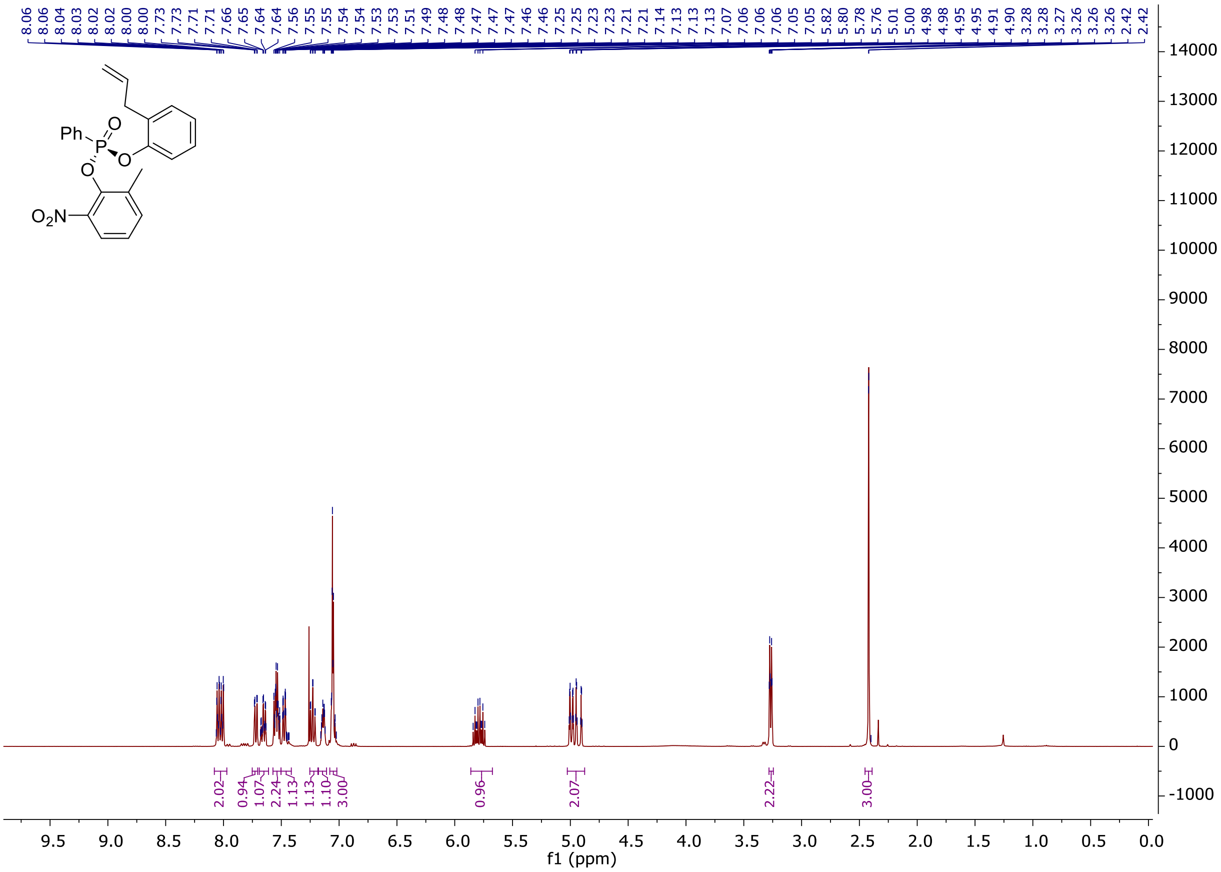

^{13}C NMR (101 MHz, $CDCl_3$):

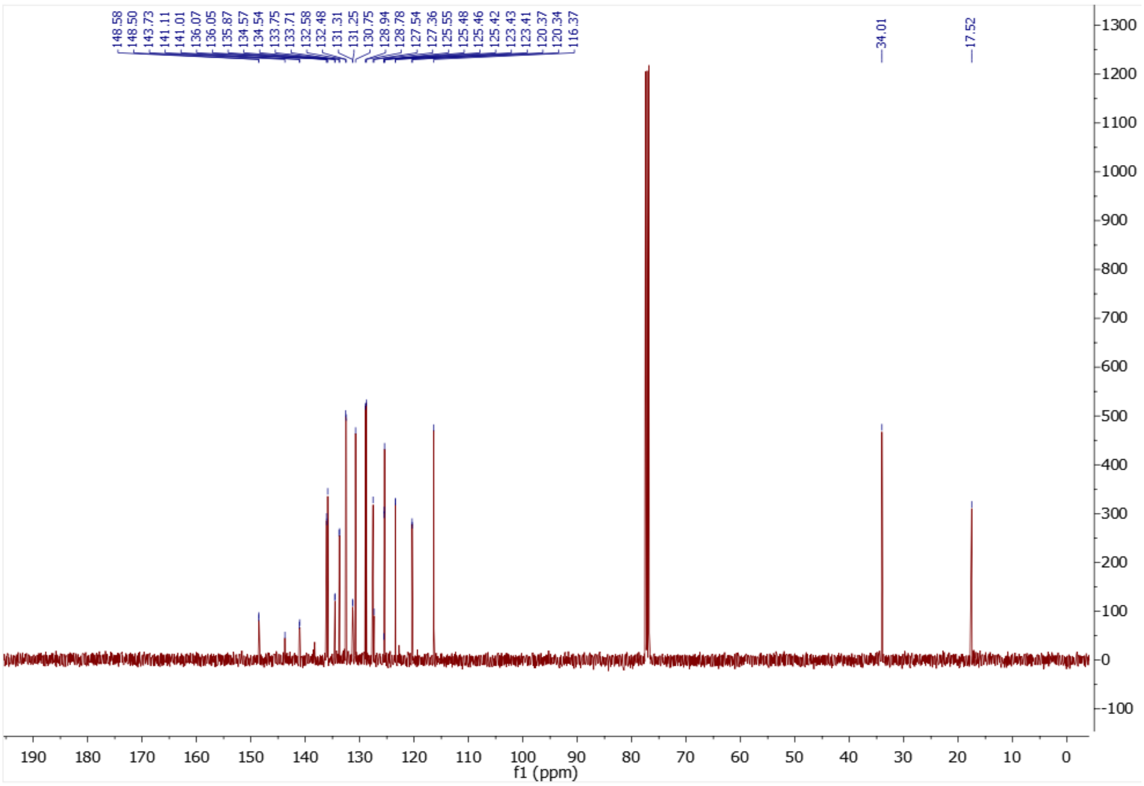

^{31}P NMR (162 MHz, CDCl$_3$):

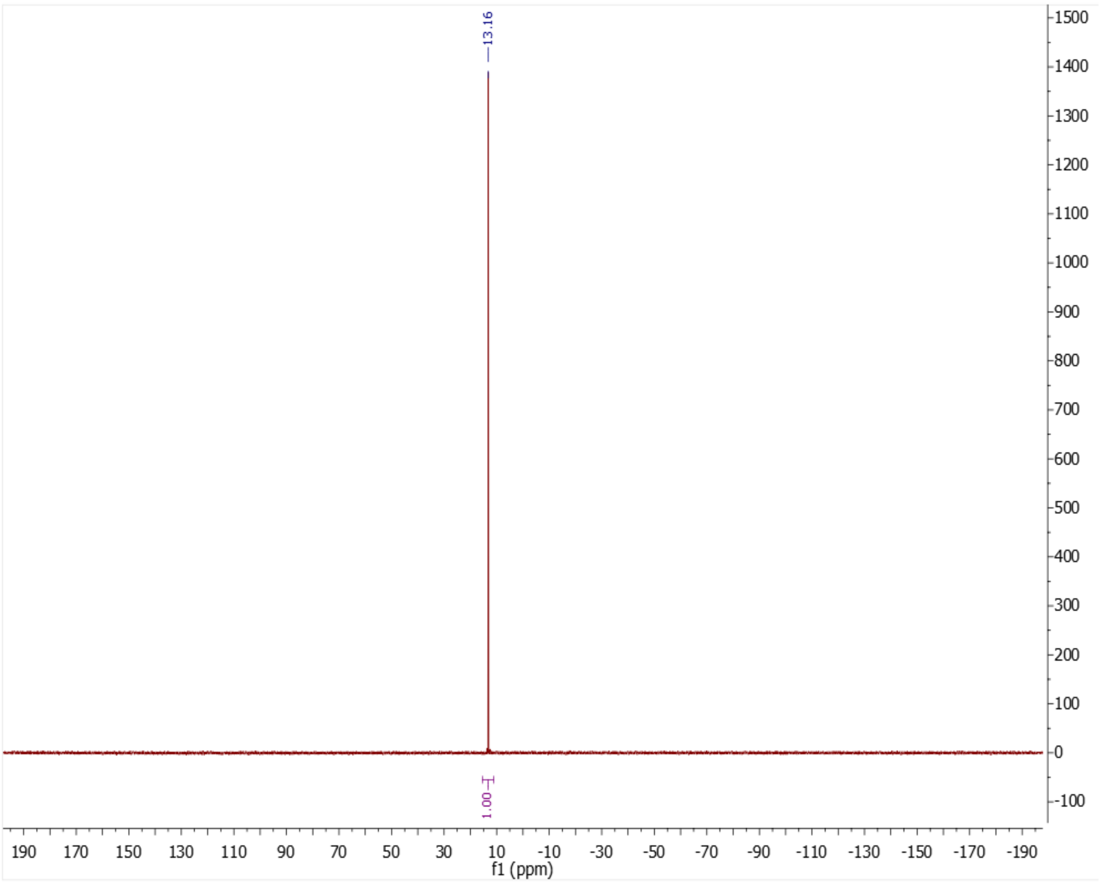

Compound **4**

^{1}H NMR (400 MHz, $CDCl_3$):

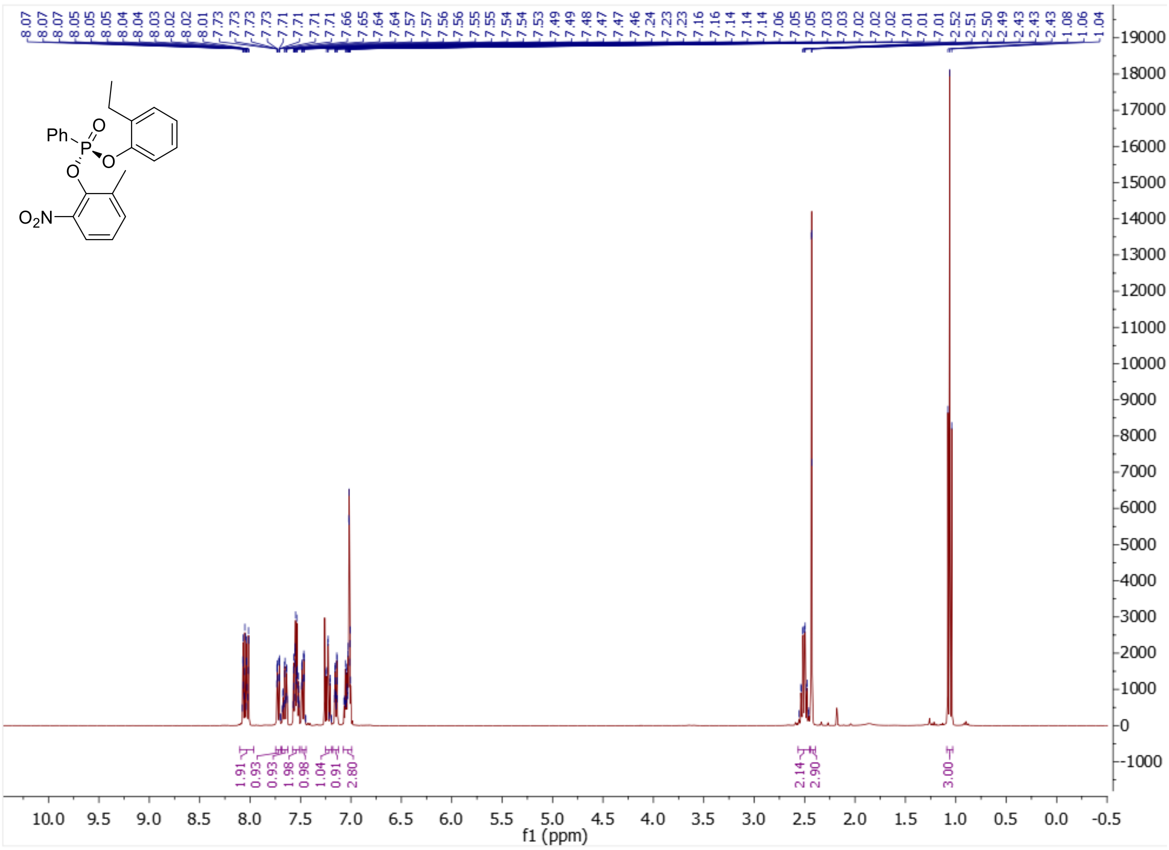

^{13}C NMR (101 MHz, $CDCl_3$):

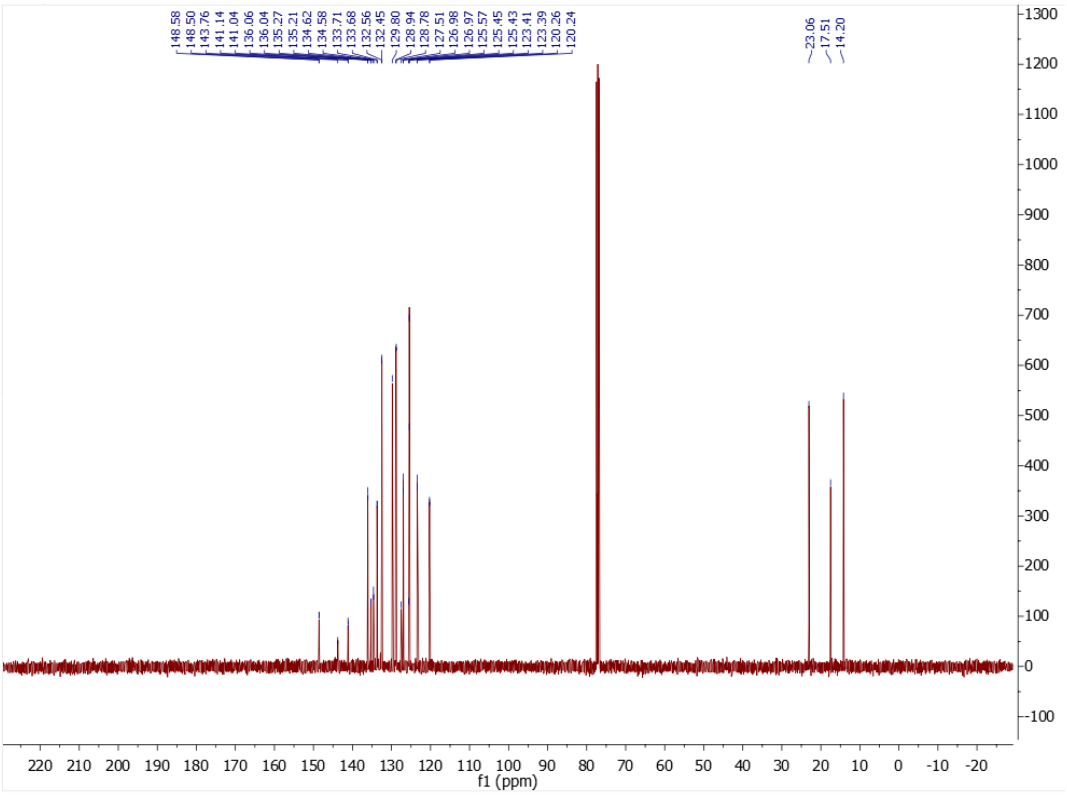

^{31}P NMR (162 MHz, $CDCl_3$):

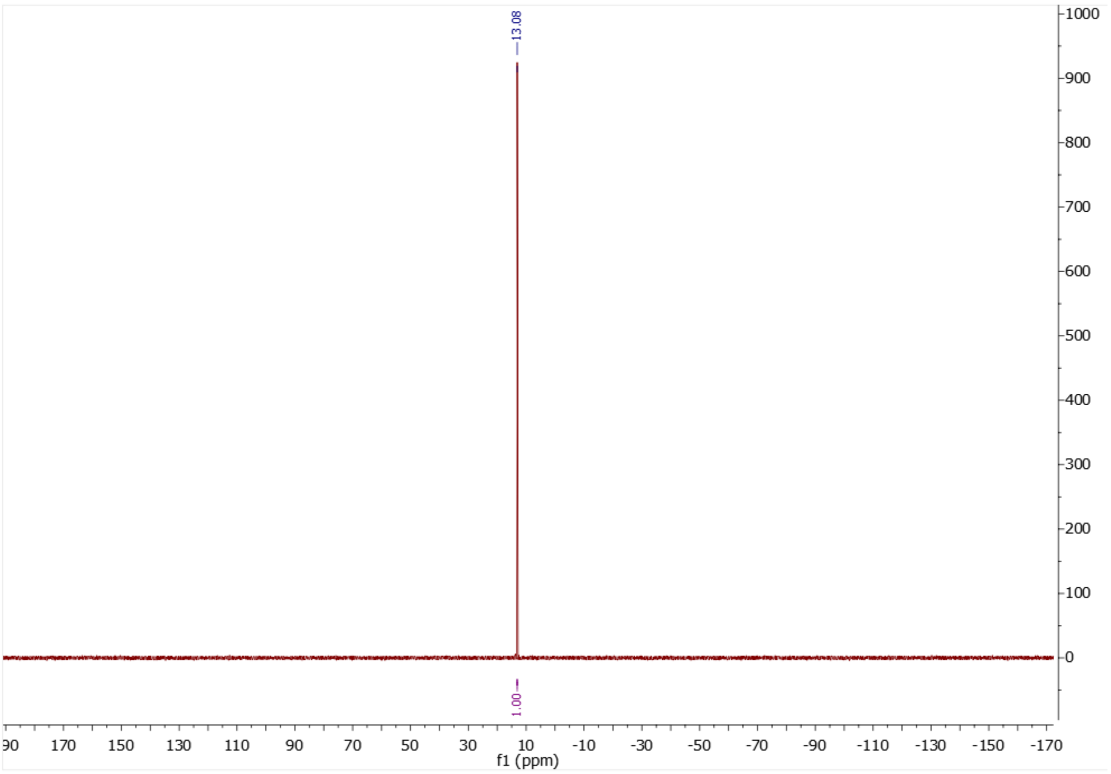

Compound **5**

^{1}H NMR (400 MHz, CDCl$_3$):

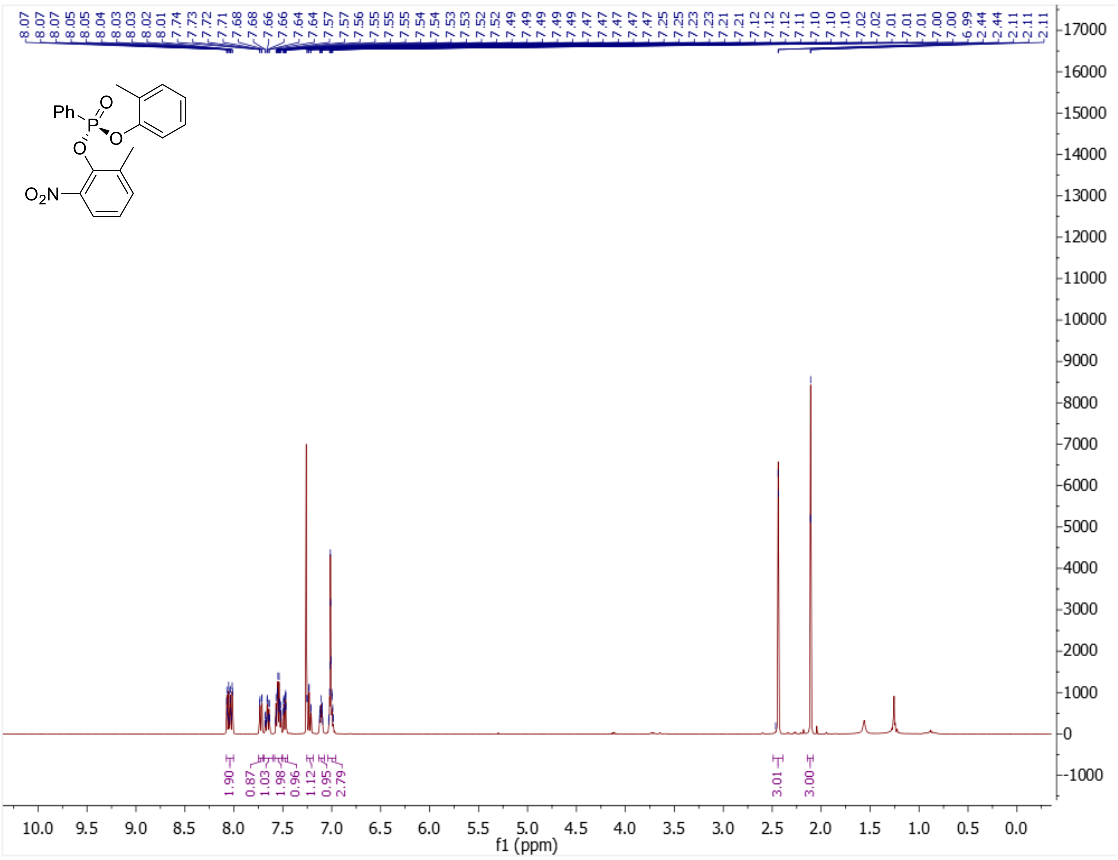

^{13}C NMR (101 MHz, CDCl$_3$):

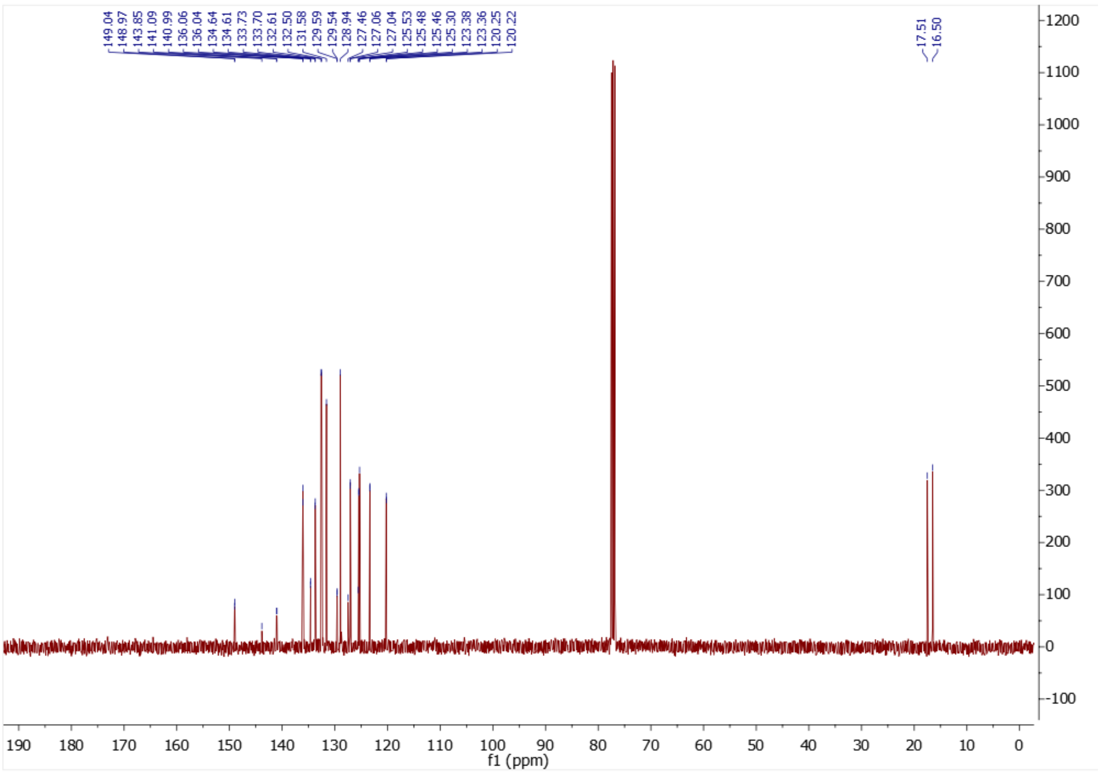

^{31}P NMR (162 MHz, $CDCl_3$):

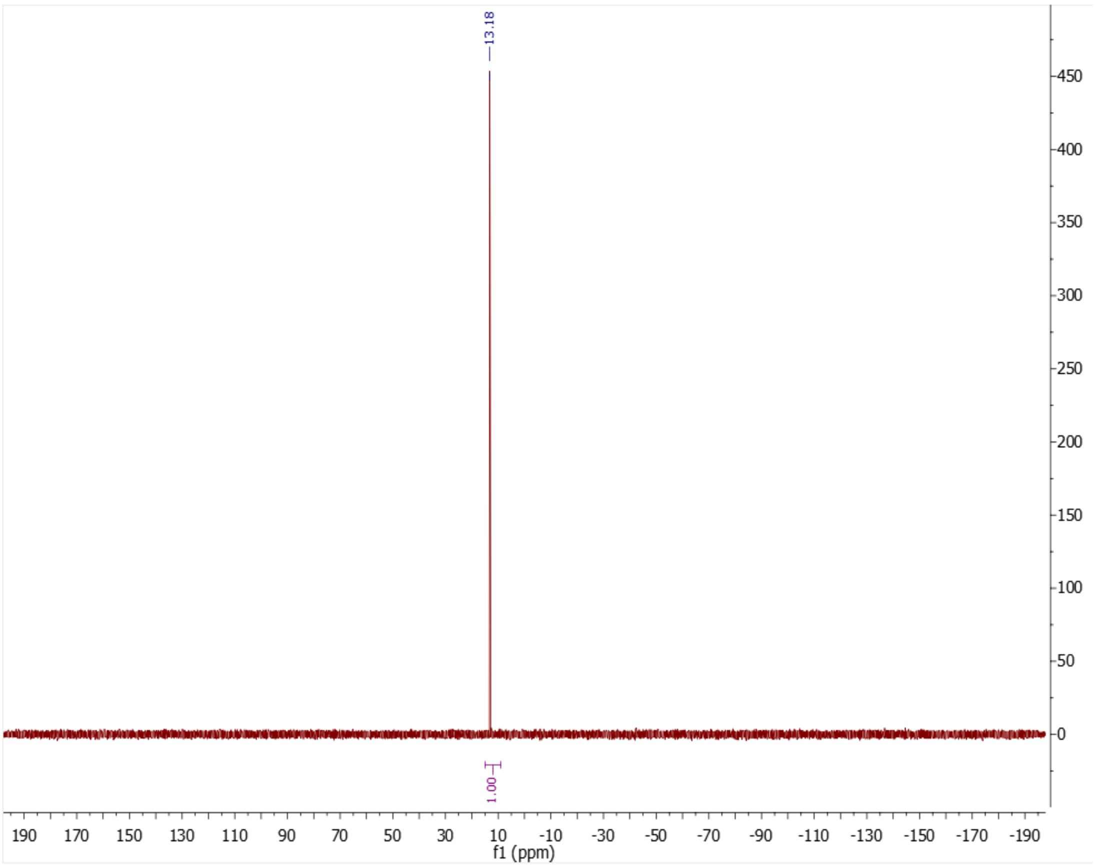

Compound **6**

^{1}H NMR (400 MHz, CDCl$_3$):

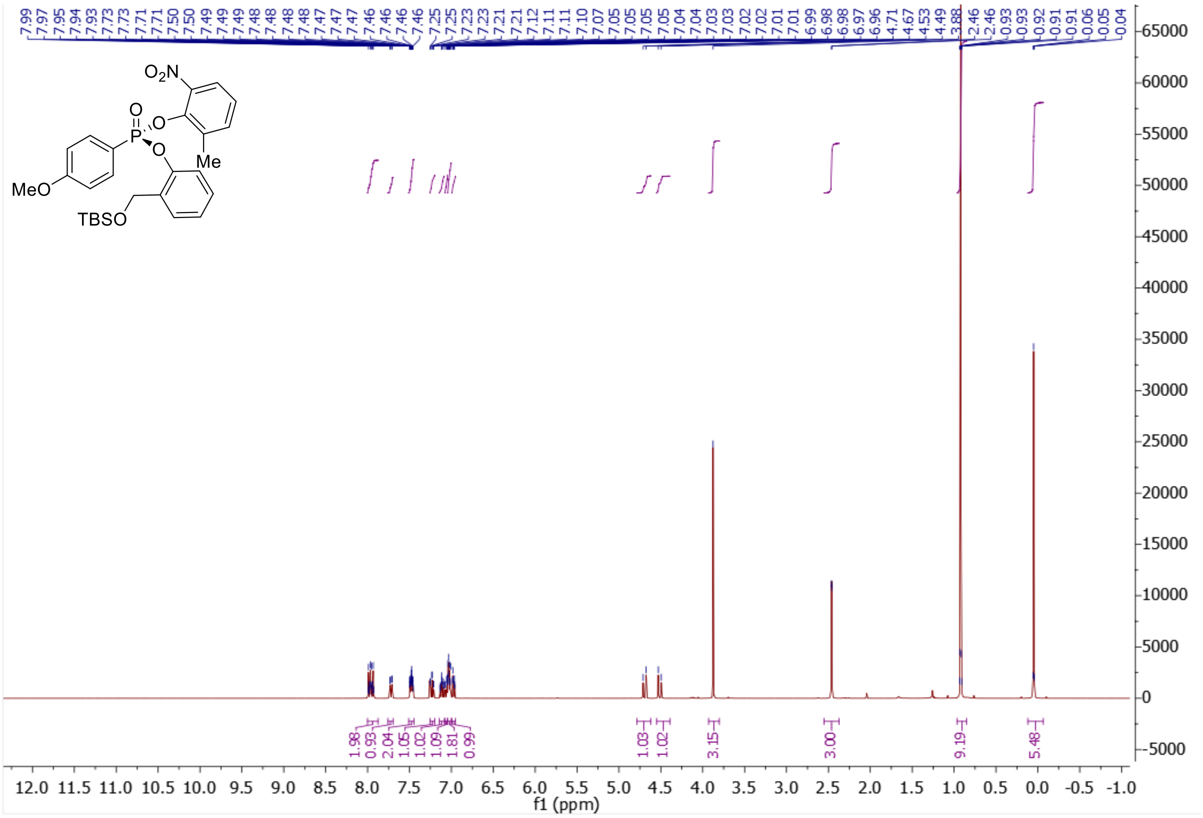

^{13}C NMR (101 MHz, CDCl$_3$):

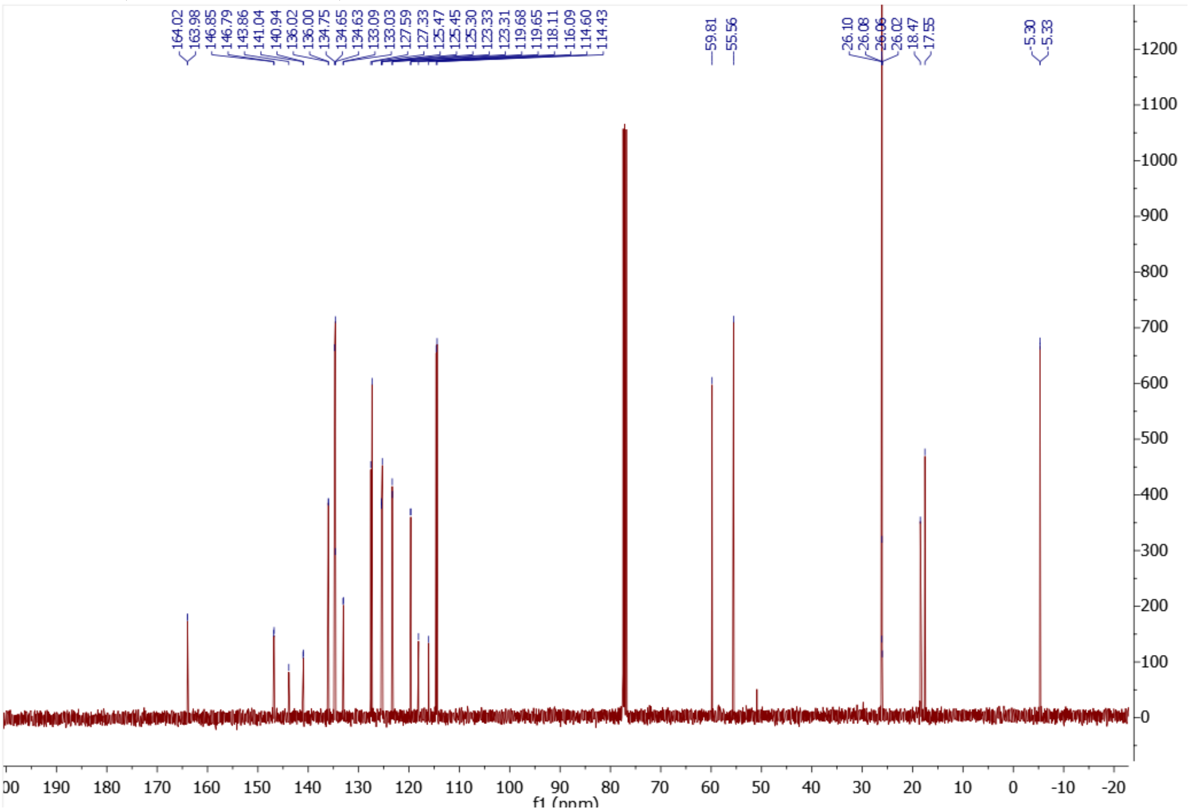

^{31}P NMR (162 MHz, CDCl$_3$):

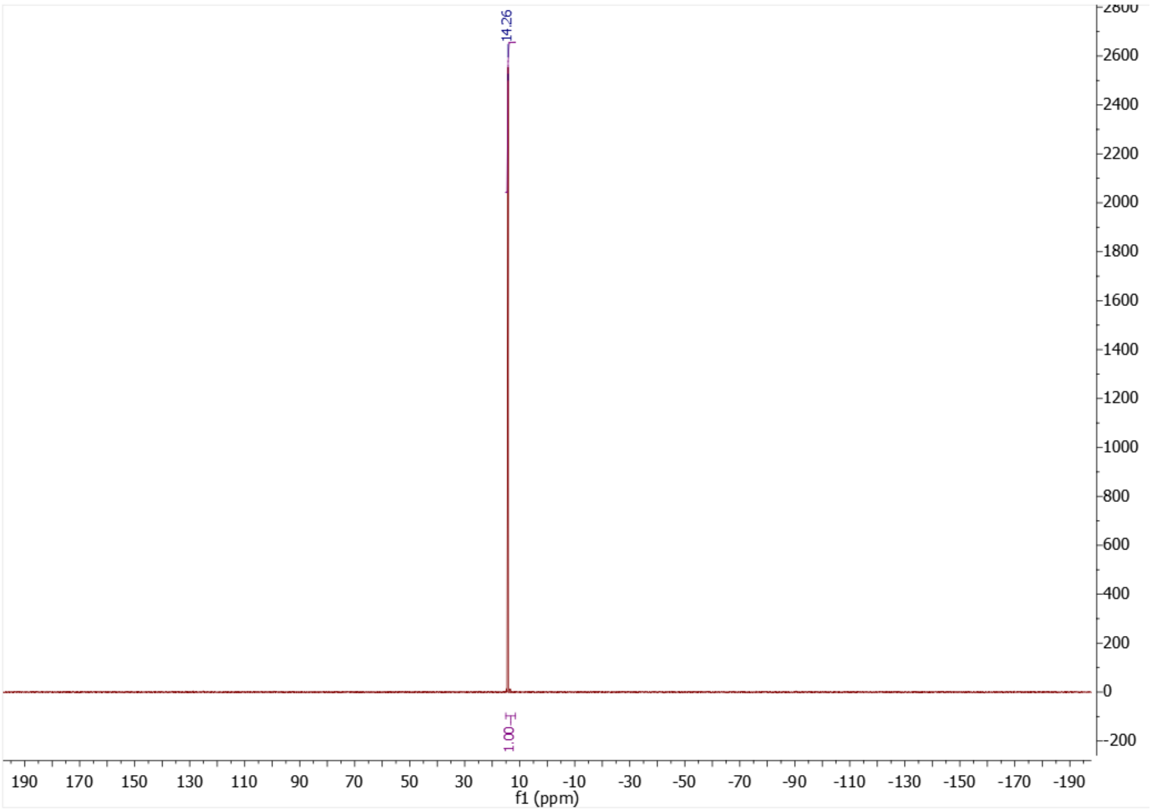

Compound **7**

^{1}H NMR (400 MHz, $CDCl_3$):

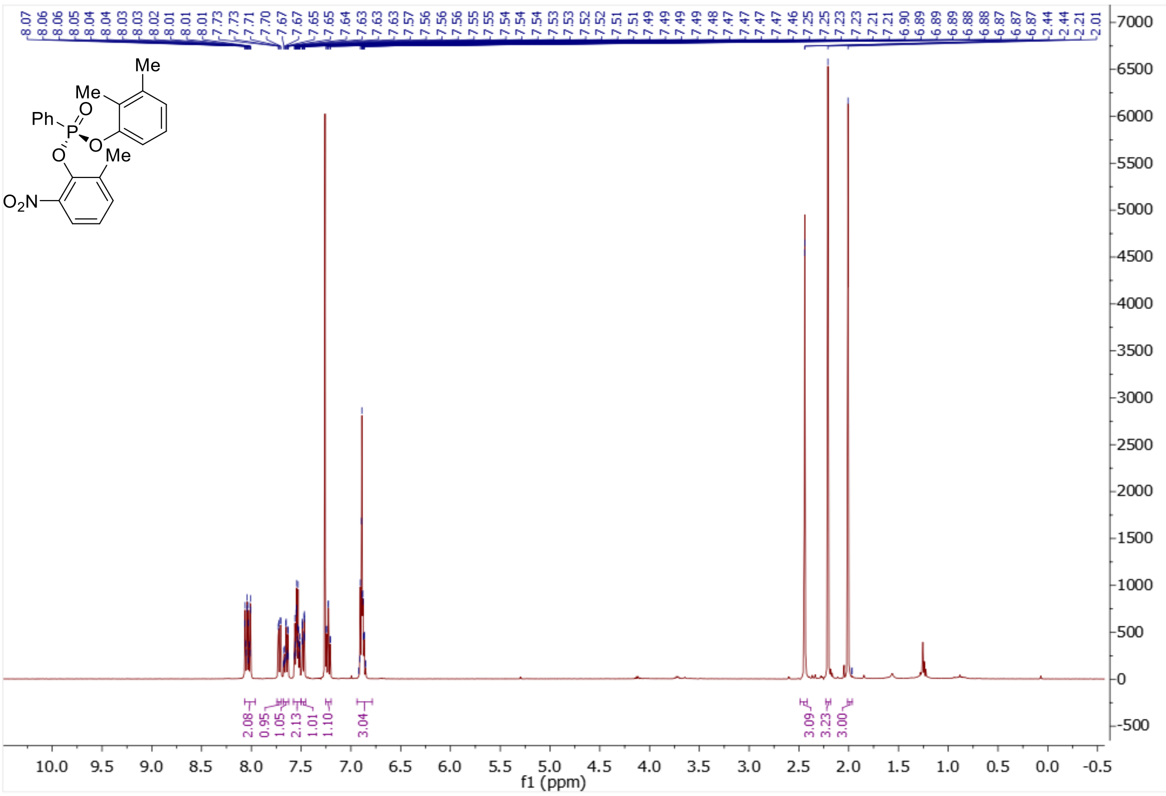

^{13}C NMR (101 MHz, $CDCl_3$):

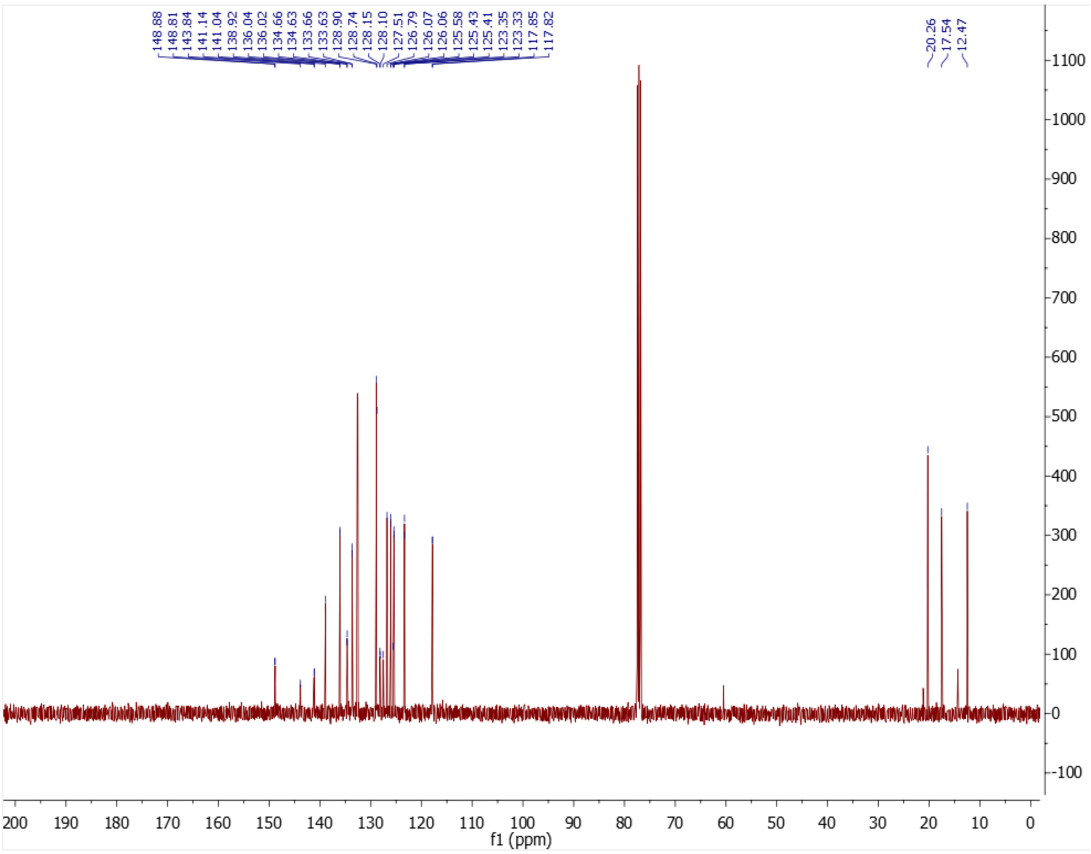

^{31}P NMR (162 MHz, $CDCl_3$):

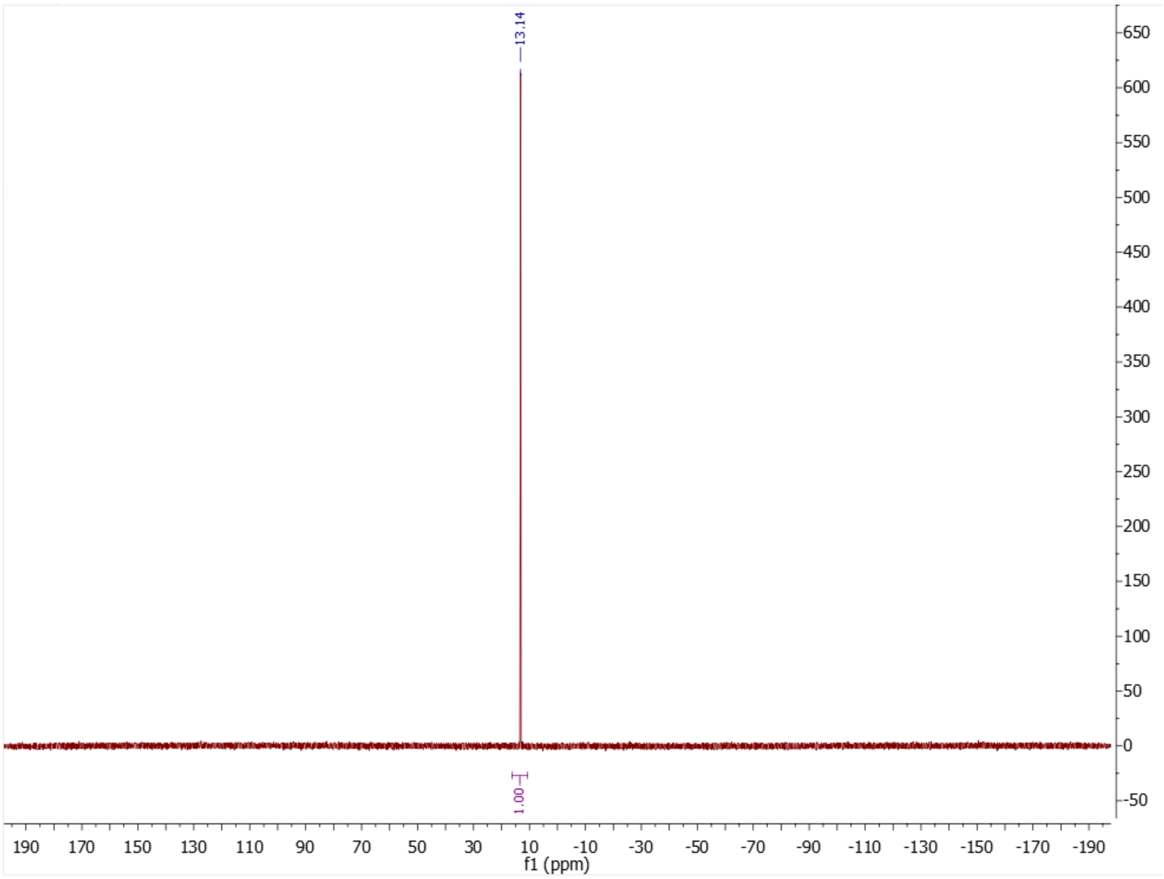

Compound **8**

^{1}H NMR (400 MHz, CDCl$_3$):

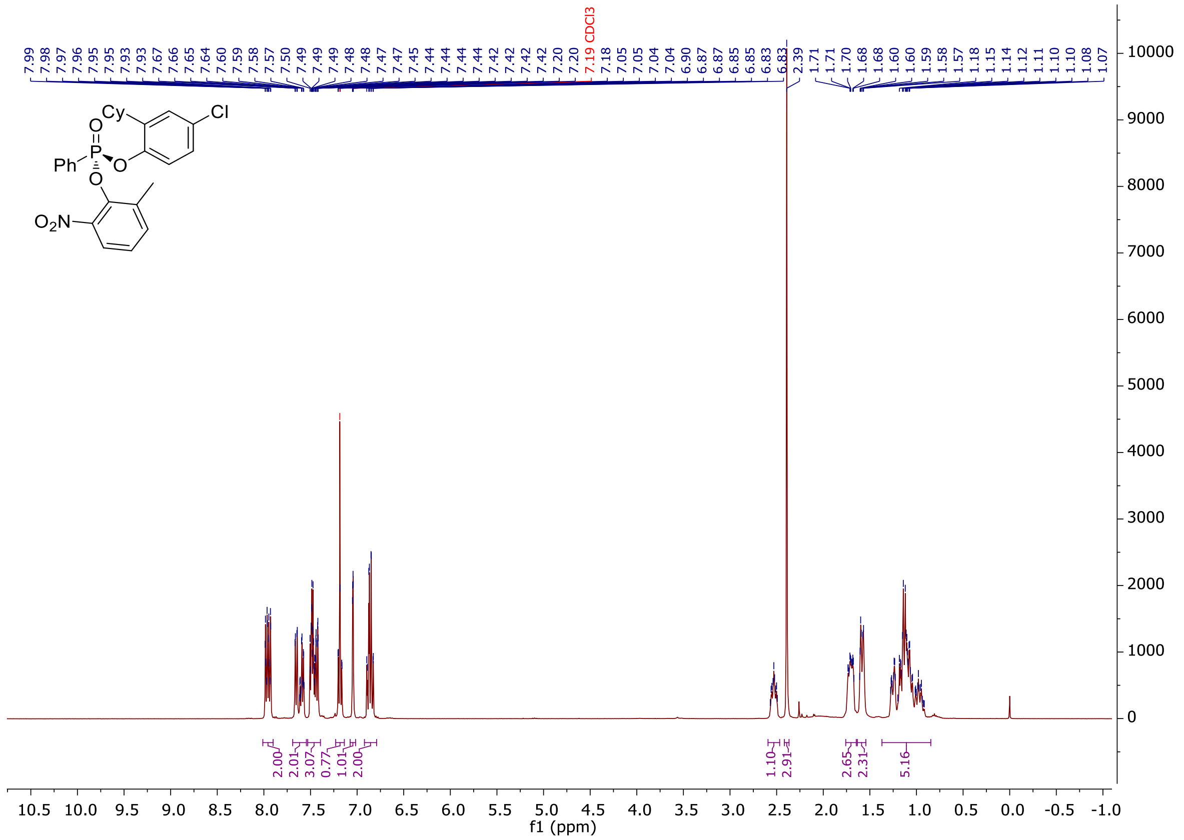

^{13}C NMR (101 MHz, CDCl$_3$):

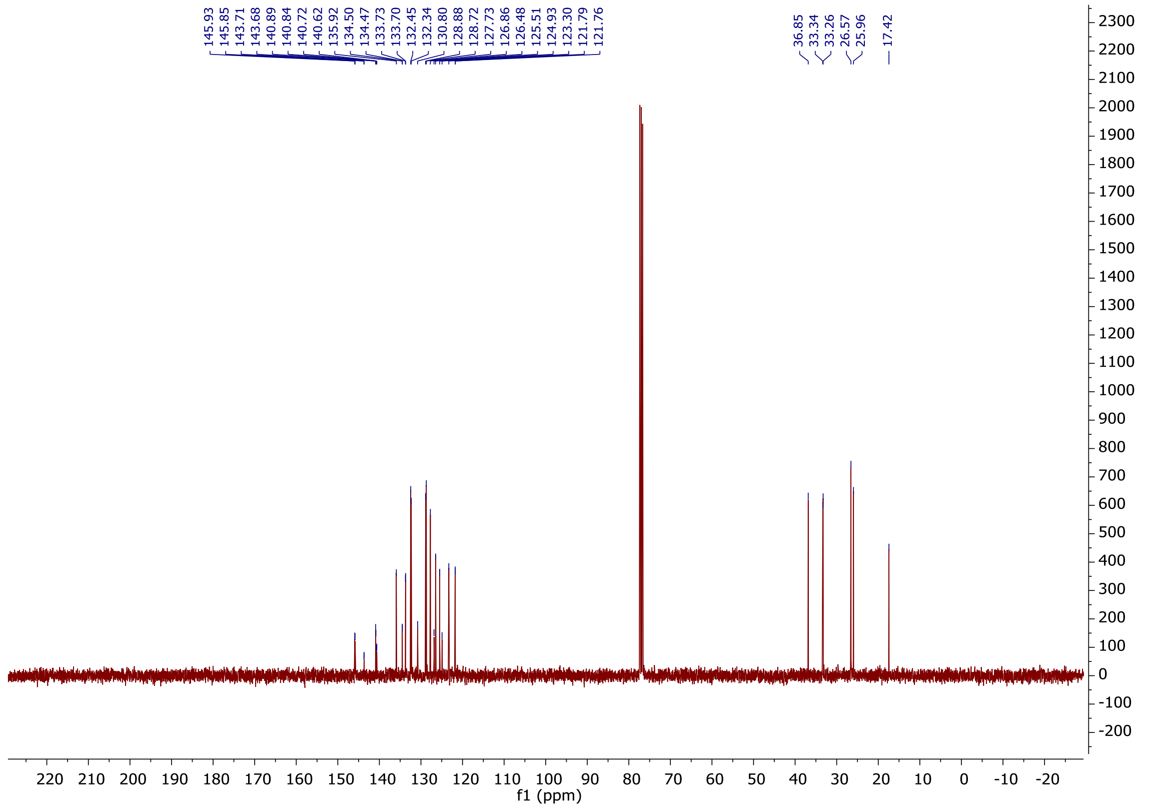

^{31}P NMR (162 MHz, $CDCl_3$):

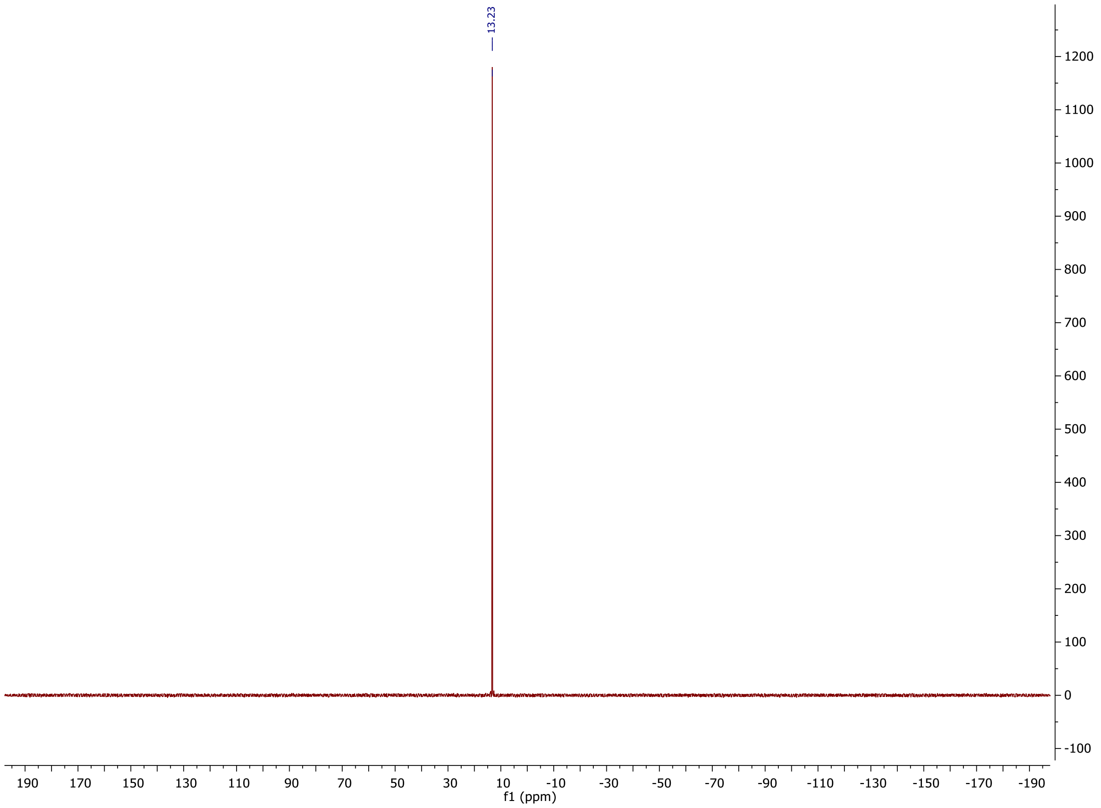

Compound **9**

^{1}H NMR (400 MHz, CDCl$_3$):

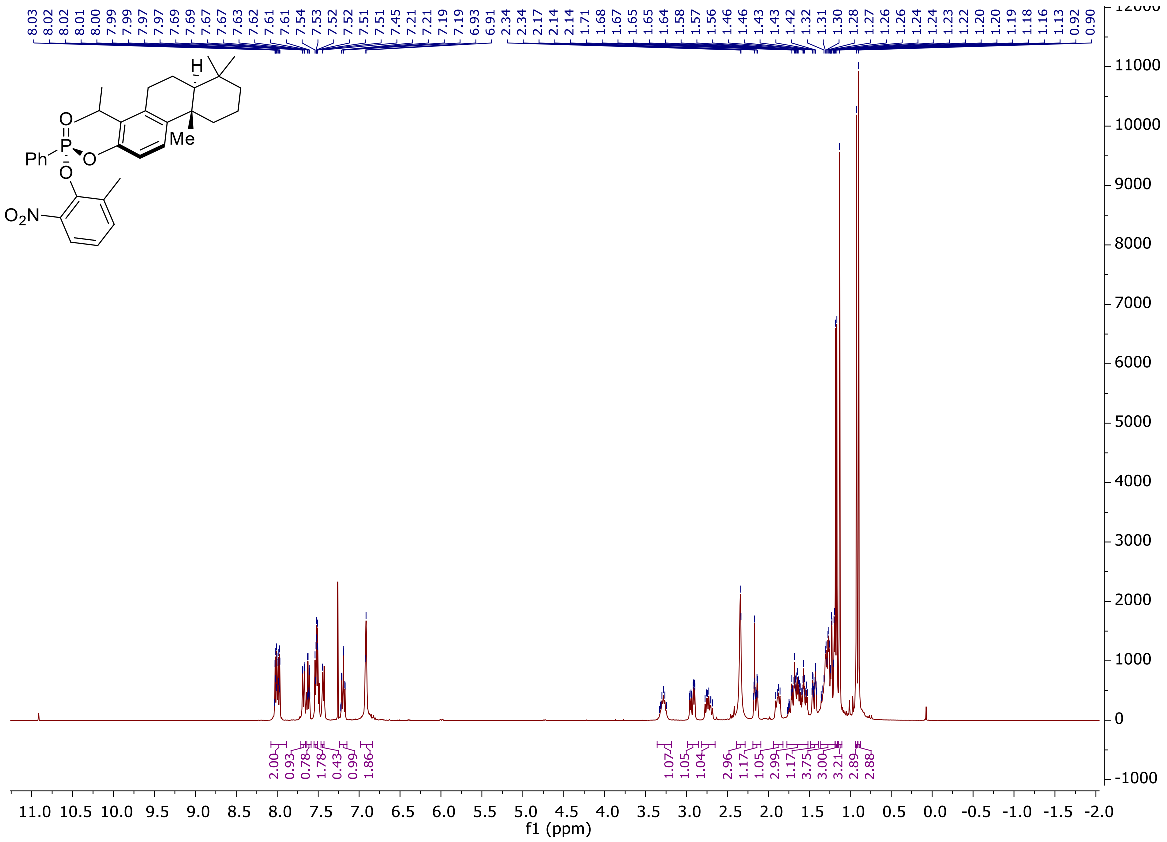

^{13}C NMR (101 MHz, CDCl$_3$):

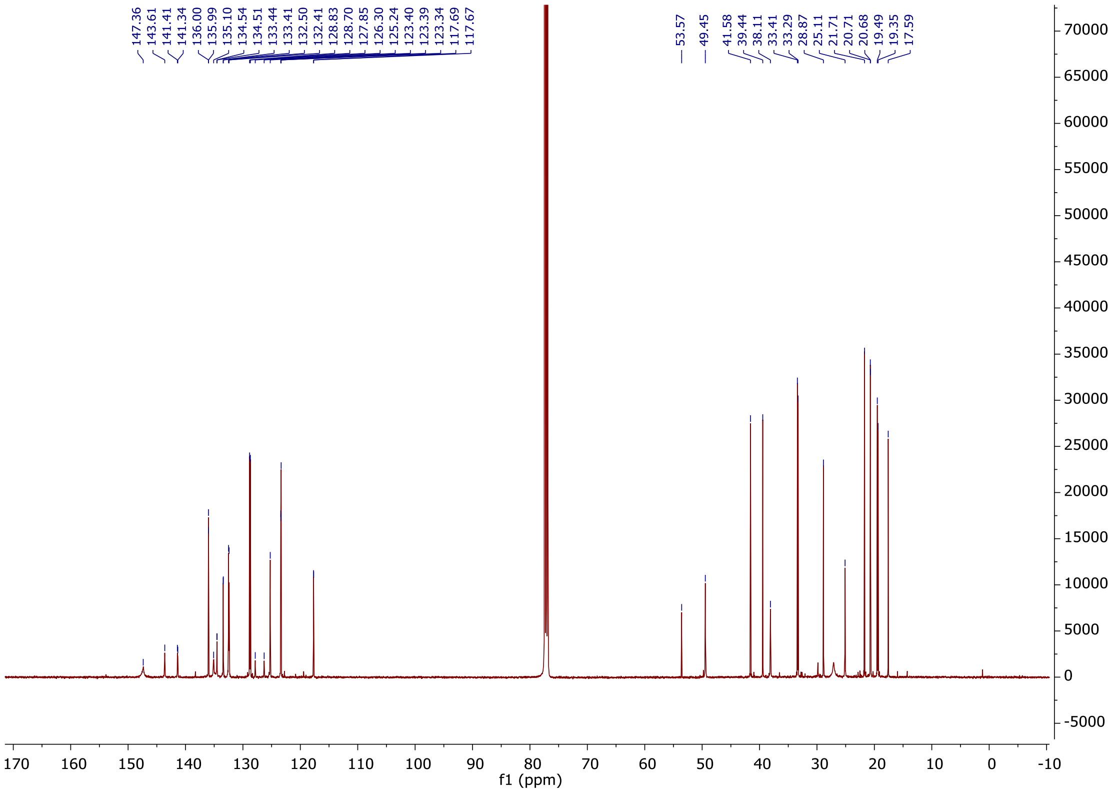

^{31}P NMR (162 MHz, $CDCl_3$) (rac):

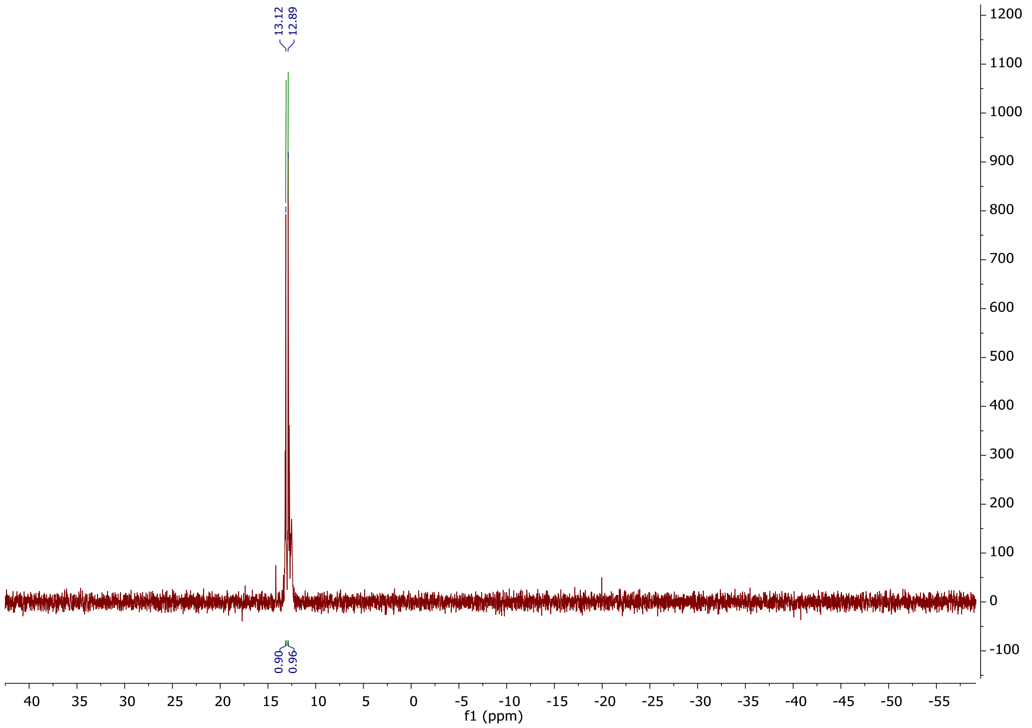

^{31}P NMR (162 MHz, $CDCl_3$) (enantioenriched):

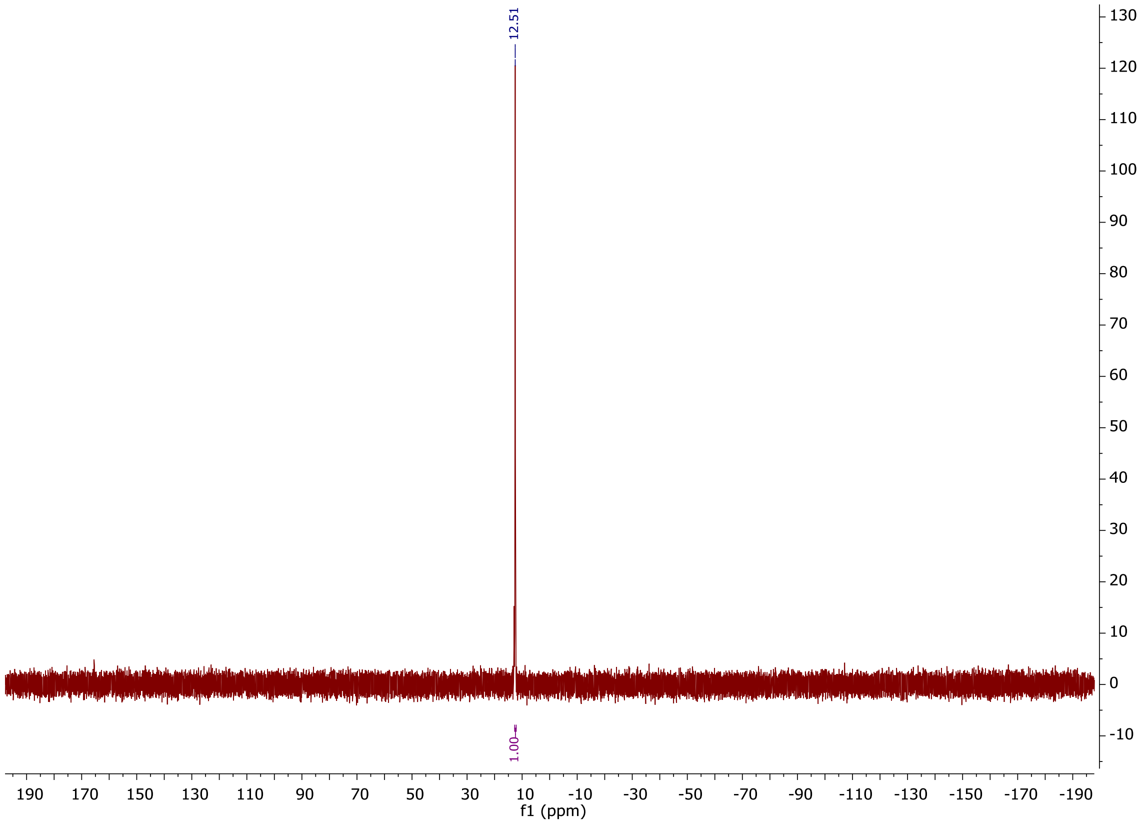

Compound **10**

^{1}H NMR (400 MHz, CDCl$_3$):

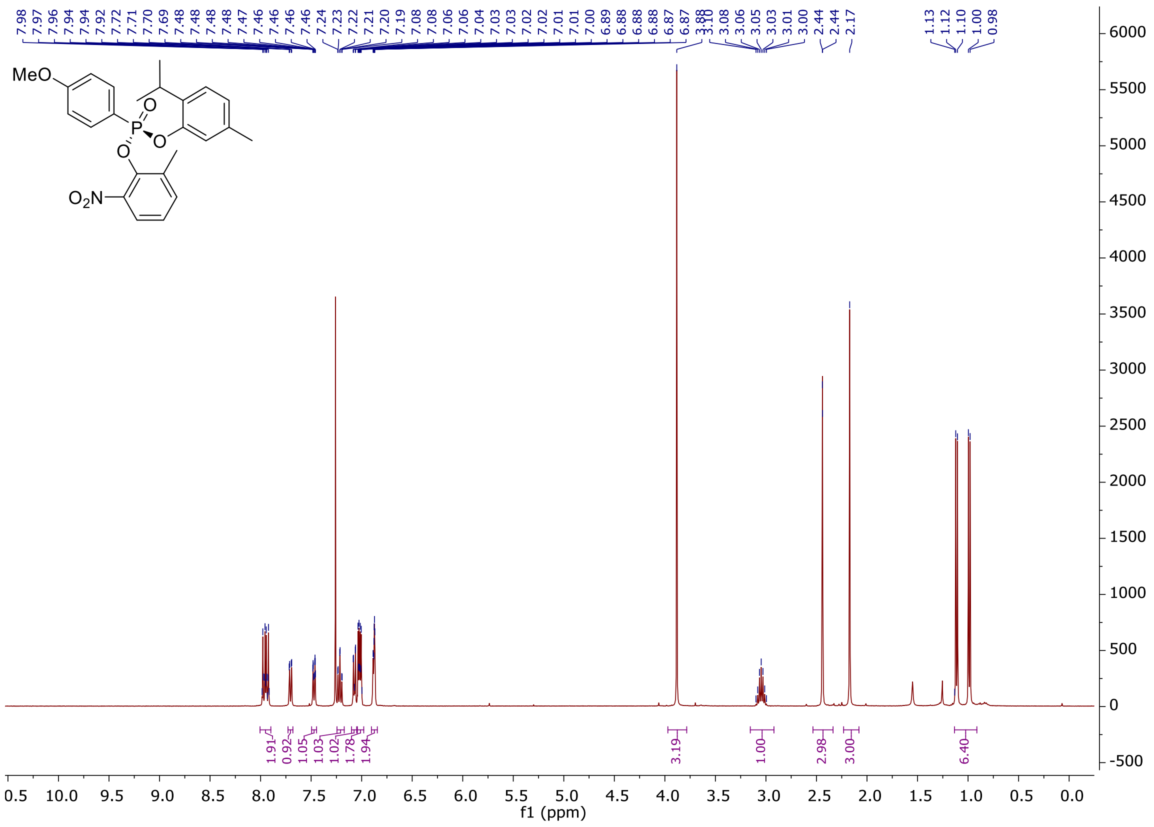

^{13}C NMR (101 MHz, CDCl$_3$):

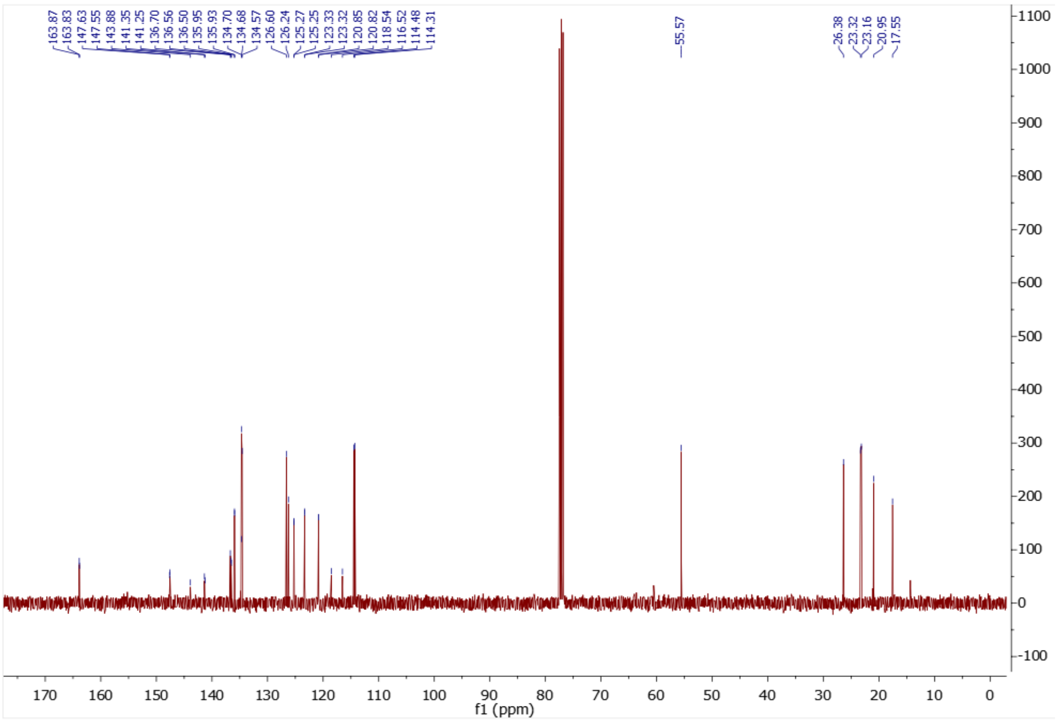

^{31}P NMR (162 MHz, CDCl$_3$):

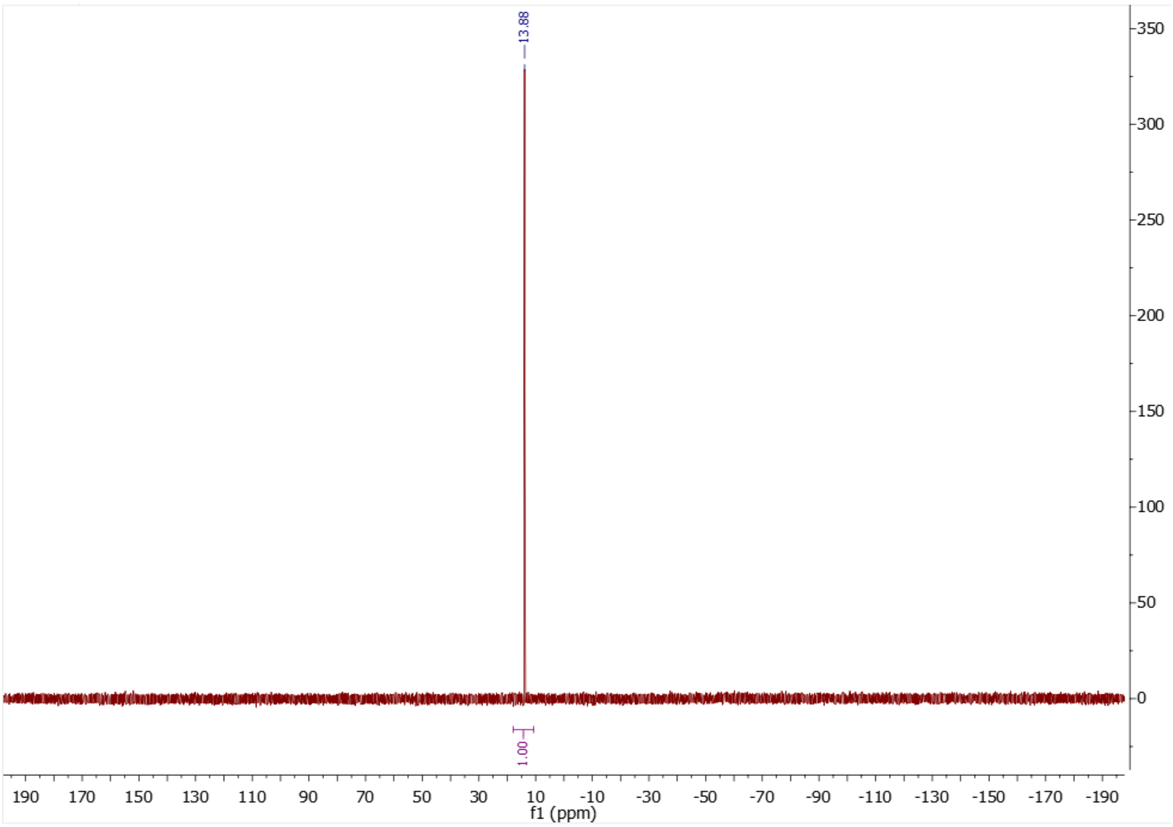

Compound **11**

^{1}H NMR (400 MHz, $CDCl_3$):

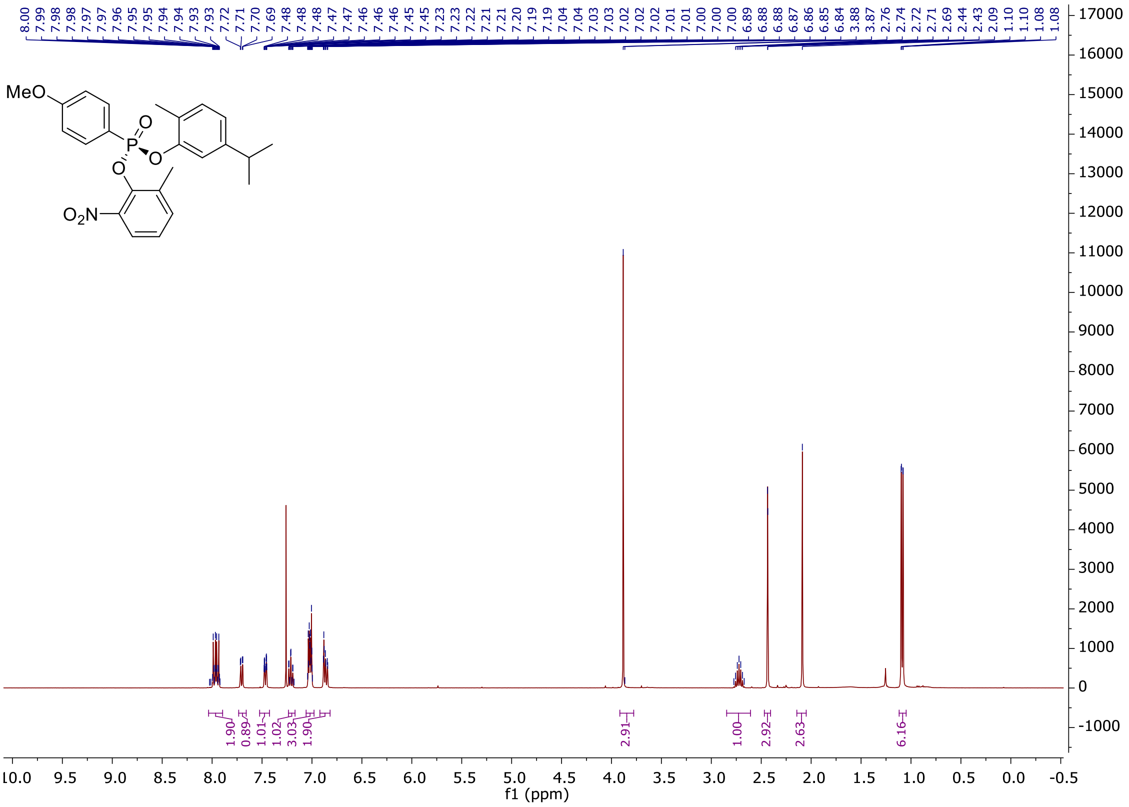

^{13}C NMR (101 MHz, $CDCl_3$):

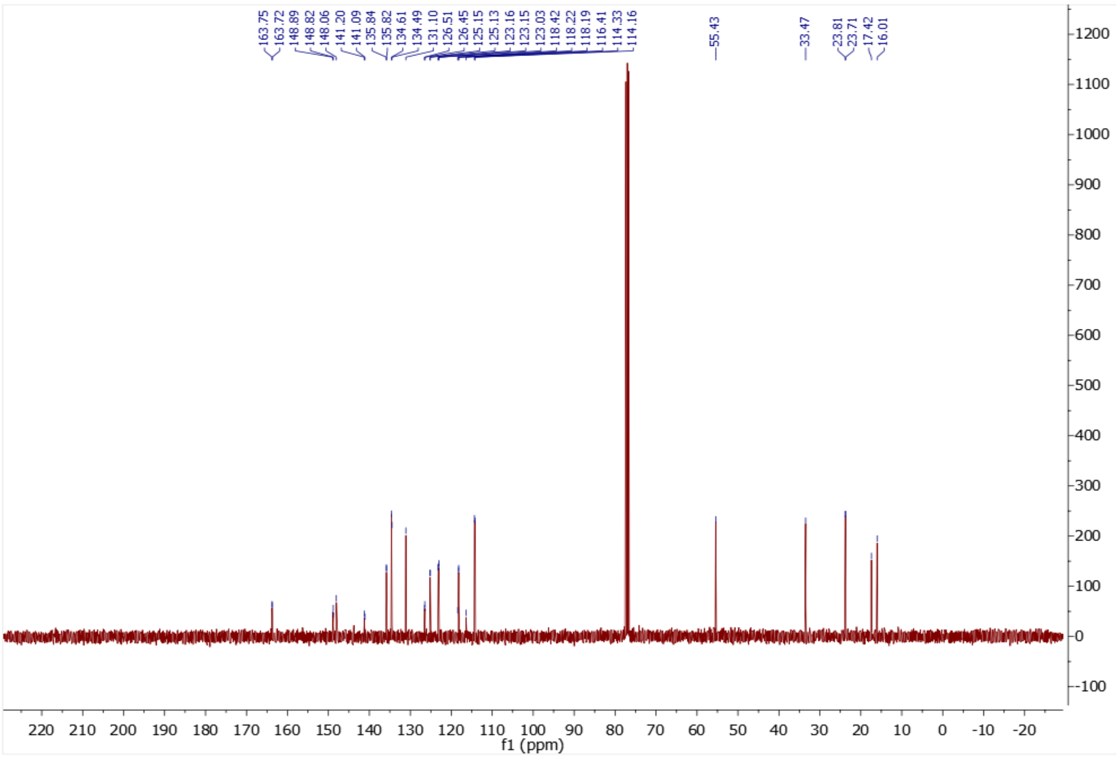

^{31}P NMR (162 MHz, $CDCl_3$):

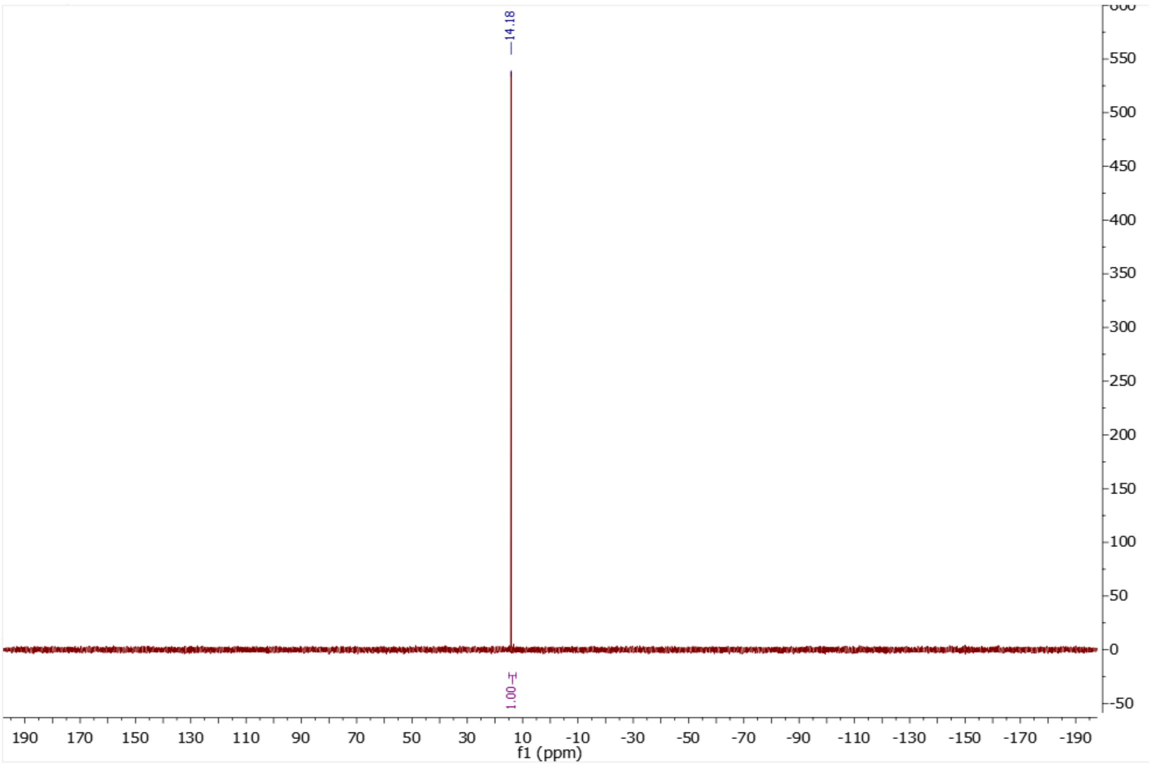

Compound **12**

^{1}H NMR (400 MHz, $CDCl_3$):

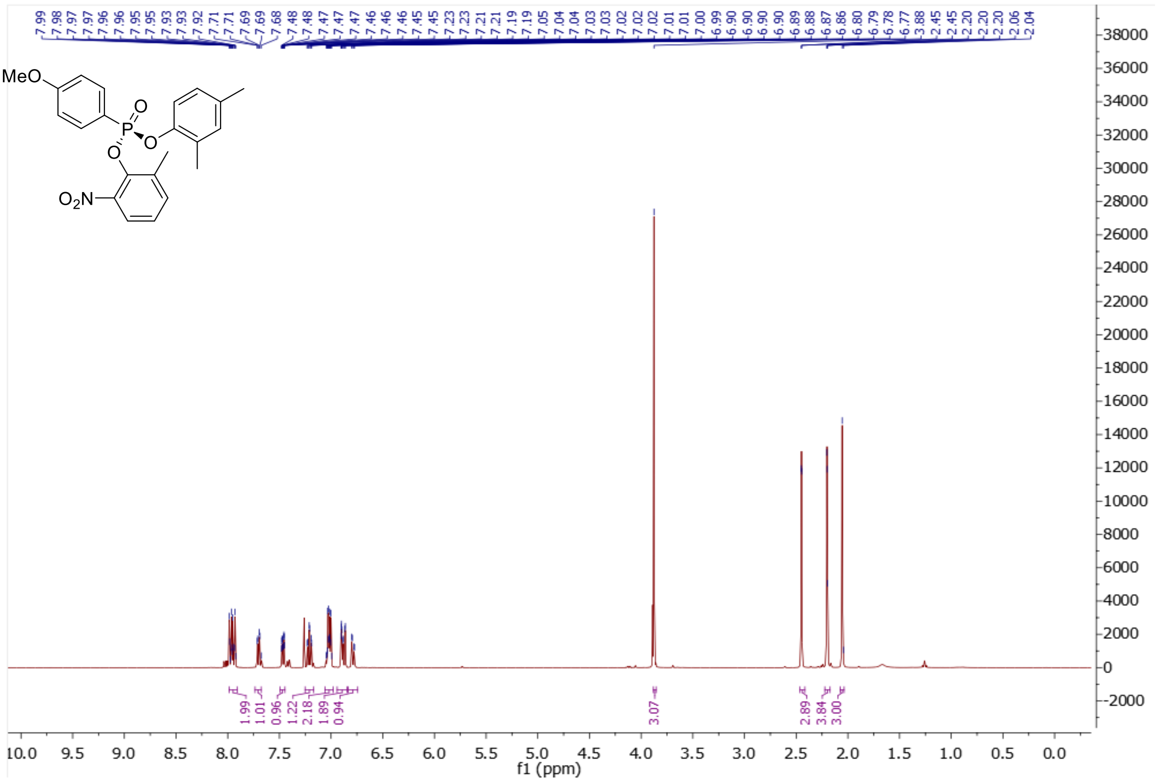

^{13}C NMR (101 MHz, $CDCl_3$):

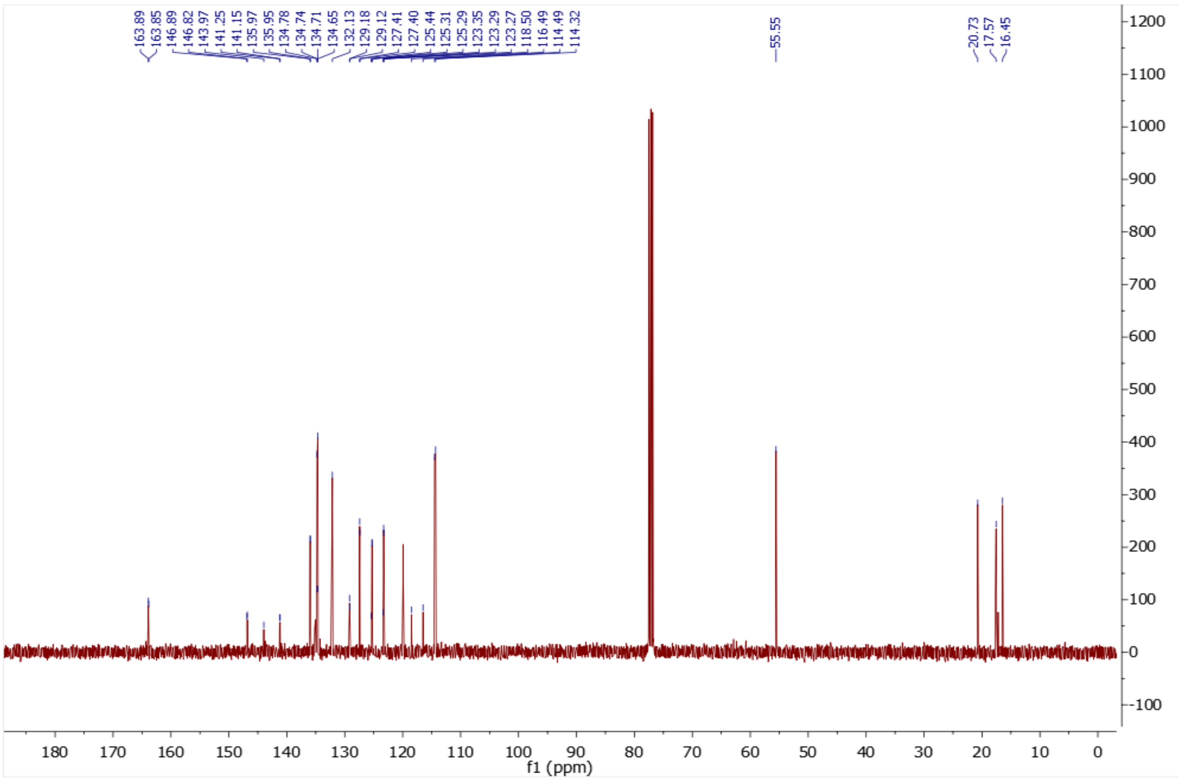

^{31}P NMR (162 MHz, $CDCl_3$):

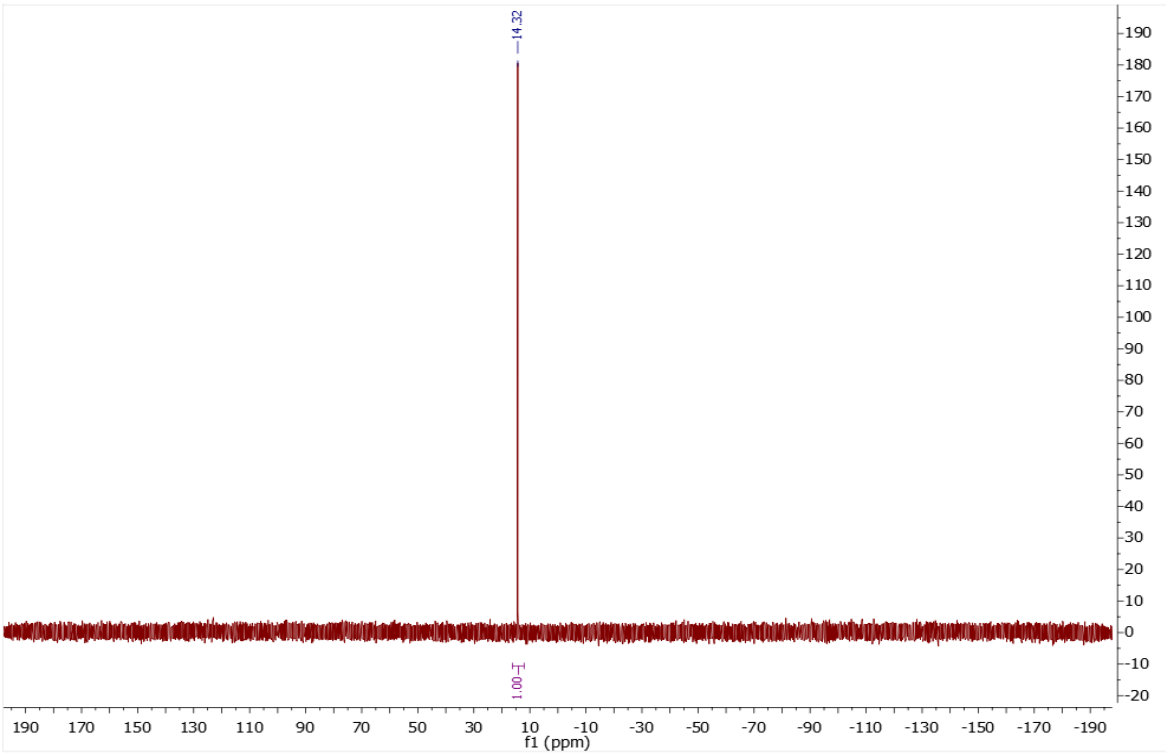

Compound **13**

^{1}H NMR (400 MHz, $CDCl_3$):

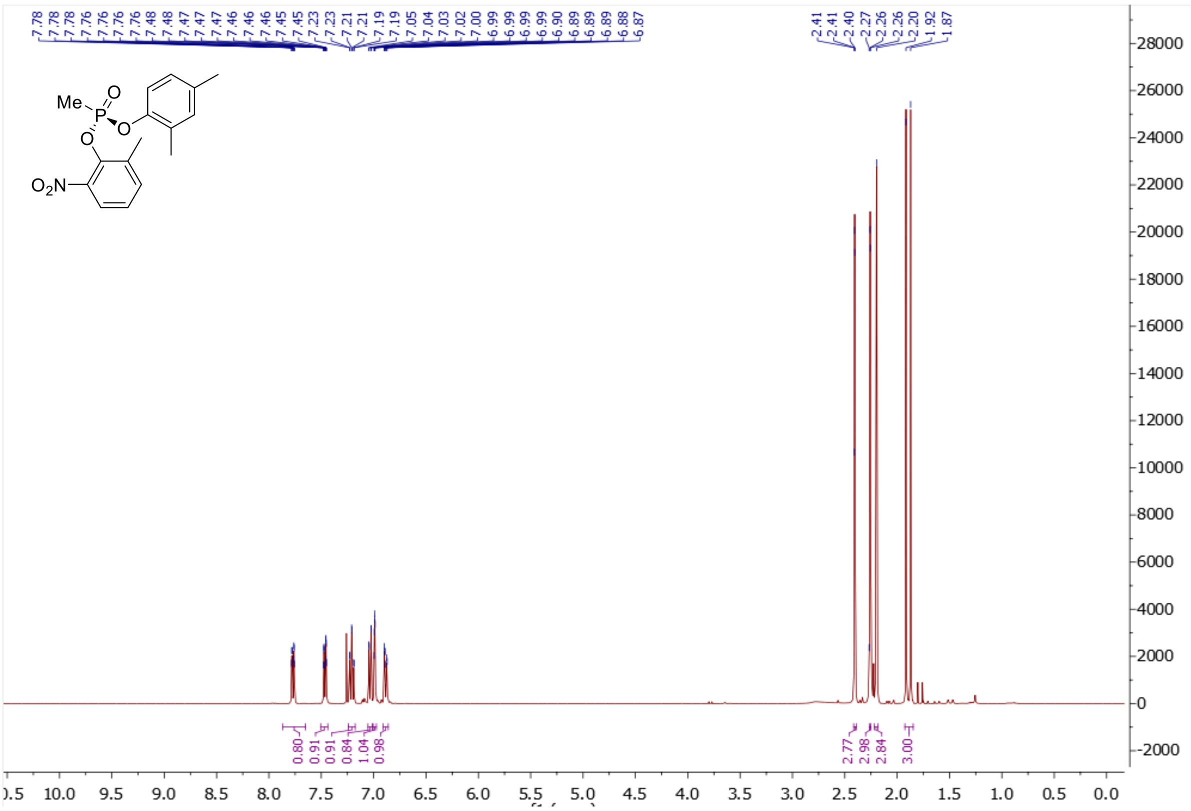

^{13}C NMR (101 MHz, $CDCl_3$):

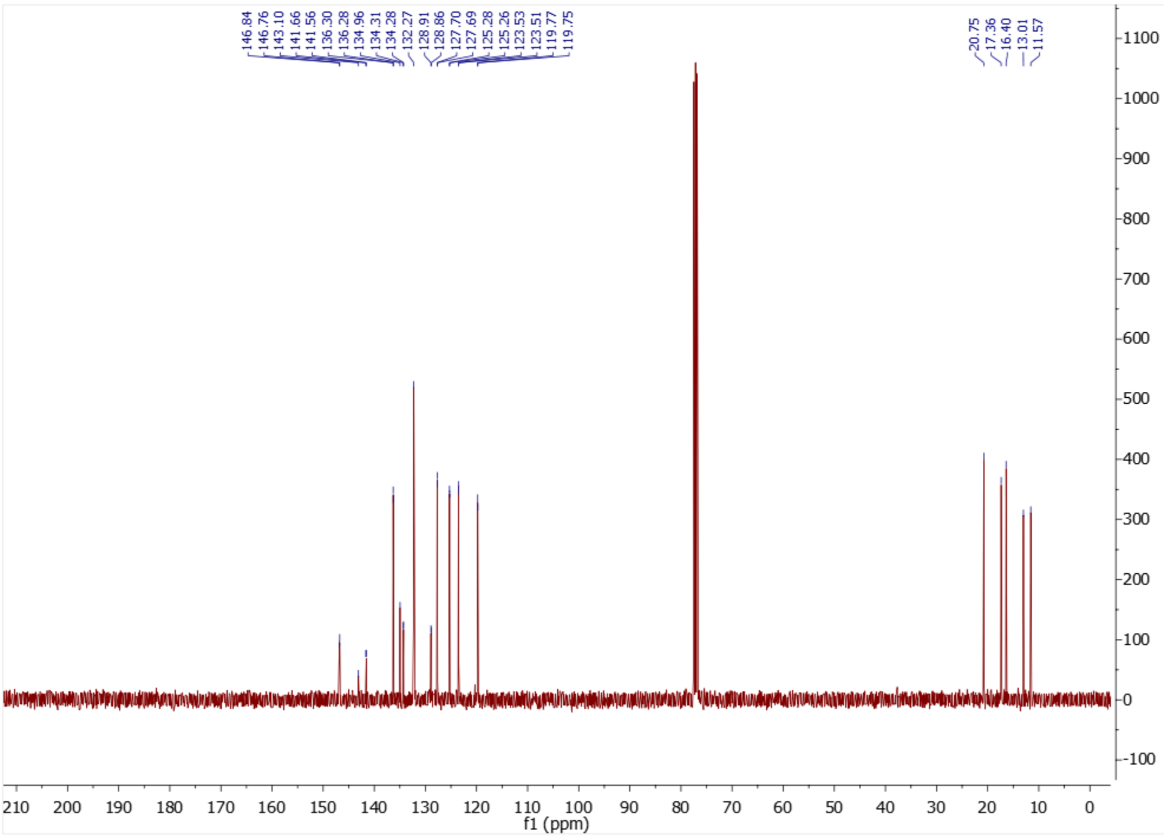

^{31}P NMR (162 MHz, CDCl$_3$):

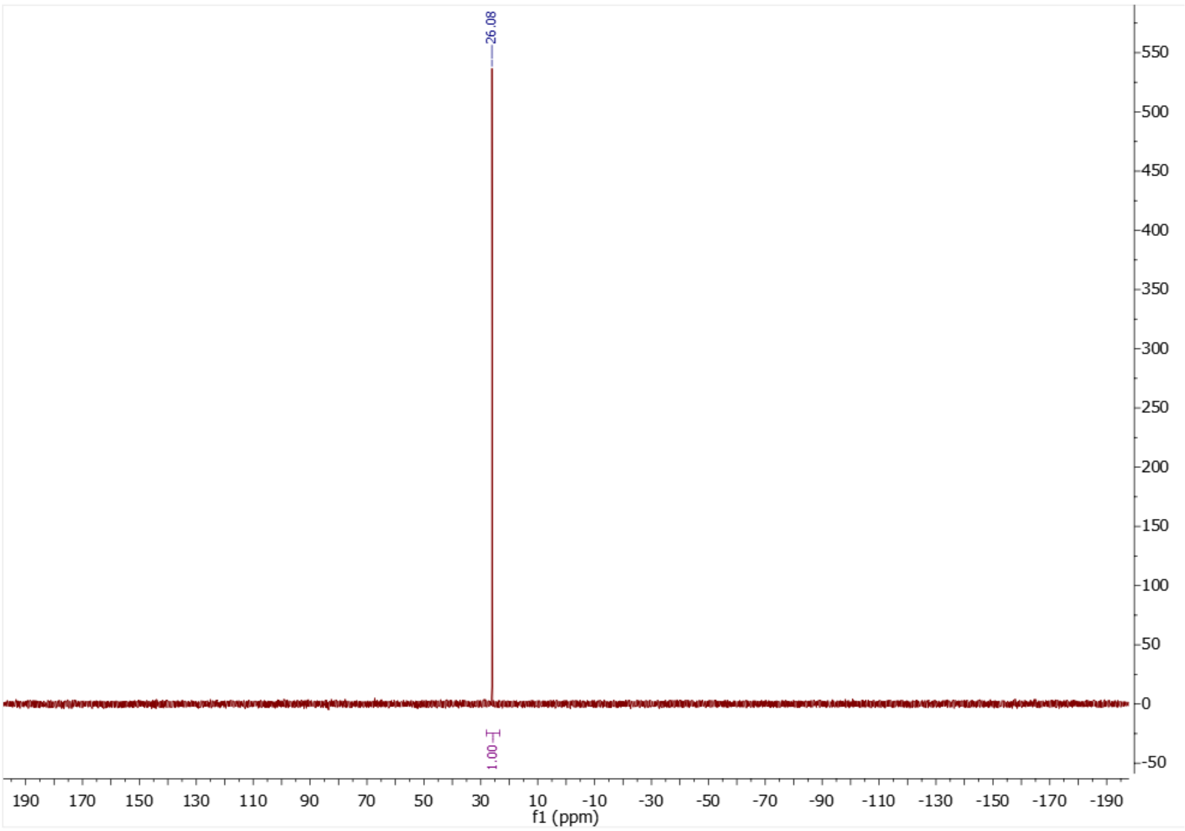

Compound **14**

^{1}H NMR (400 MHz, CDCl$_3$):

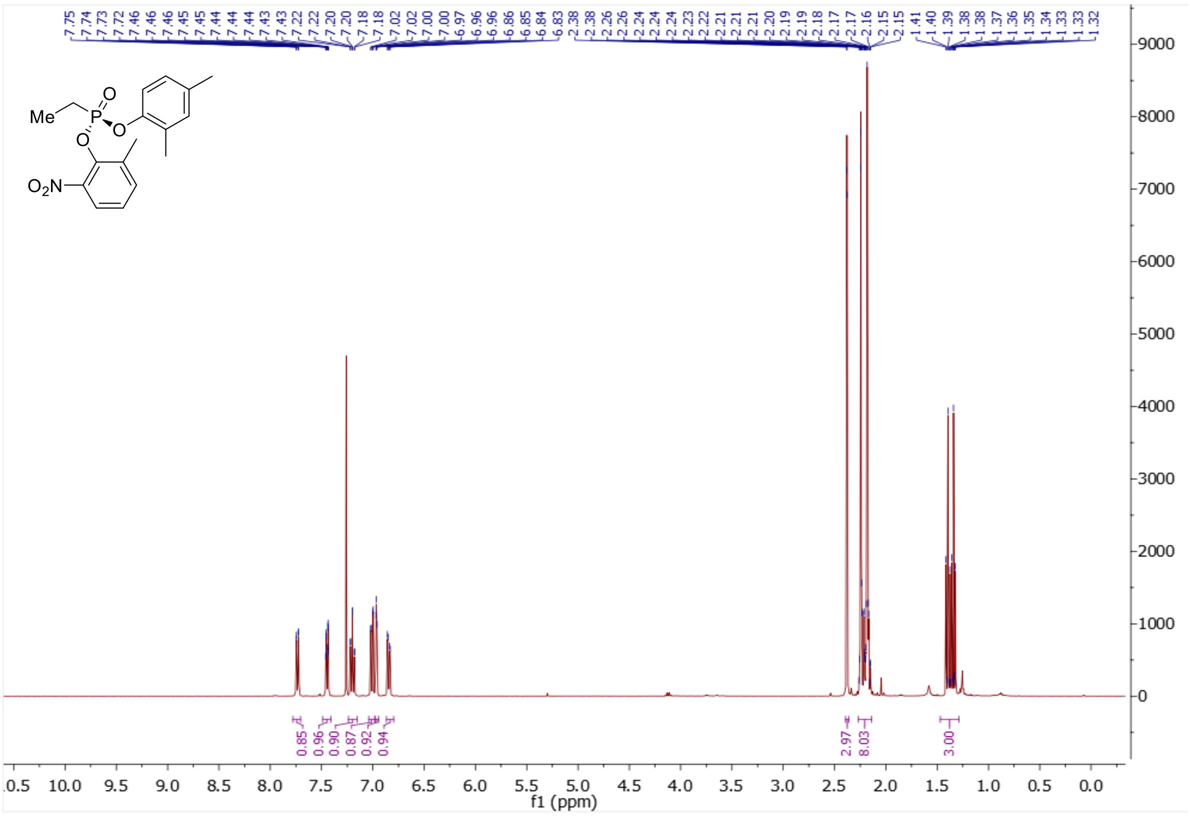

^{13}C NMR (101 MHz, CDCl$_3$):

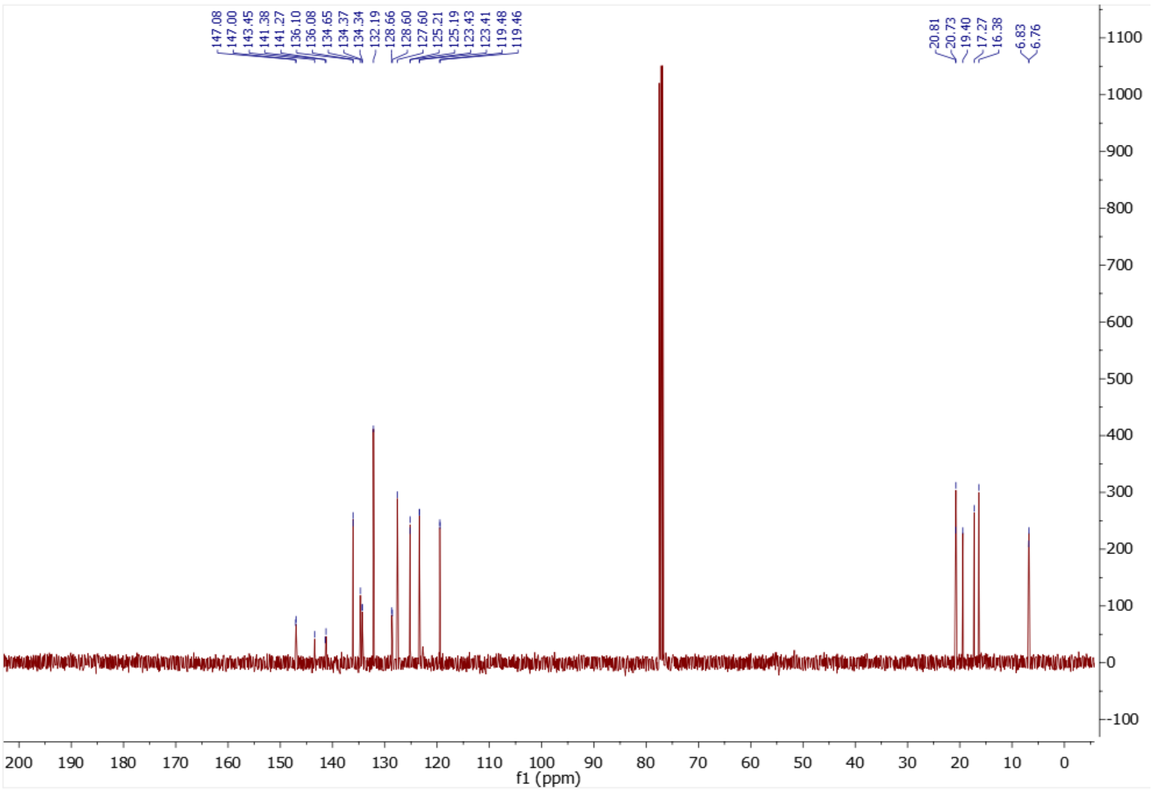

^{31}P NMR (162 MHz, $CDCl_3$):

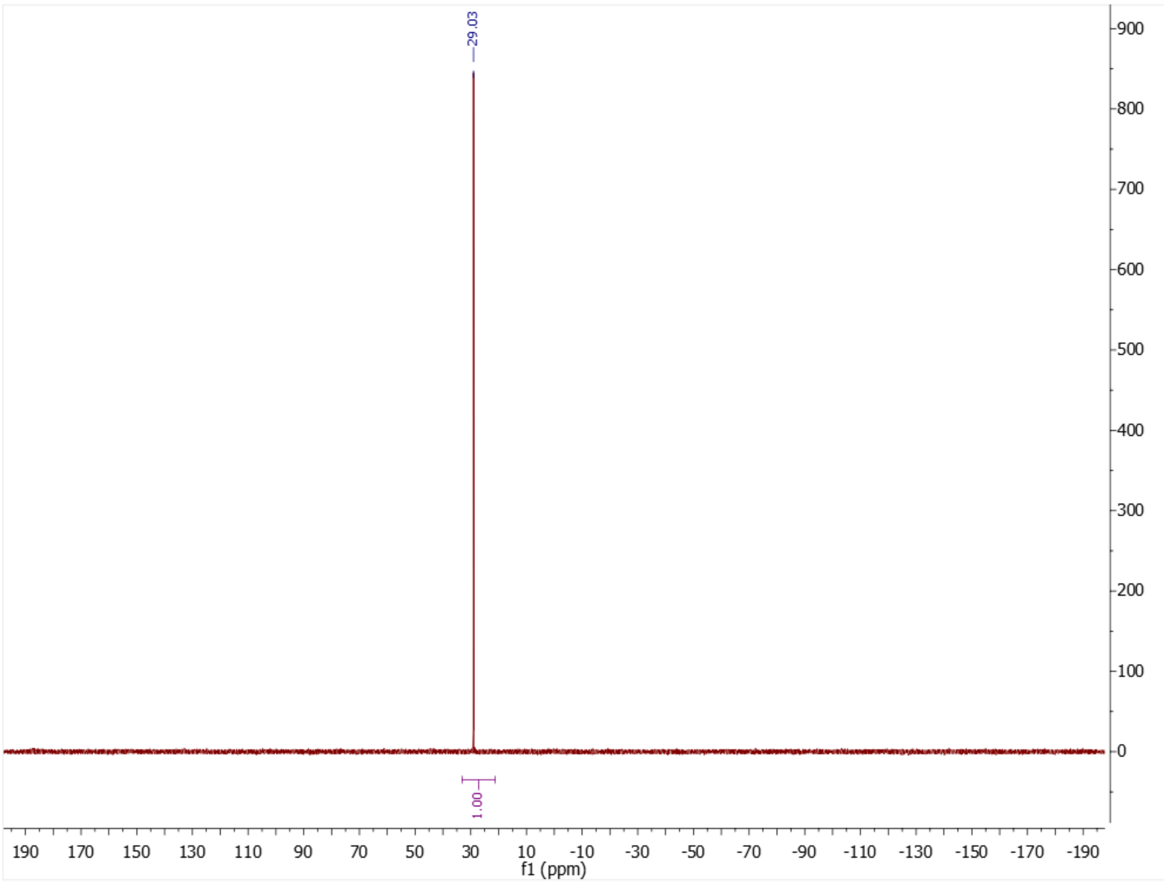

Compound **15**

^{1}H NMR (600 MHz, $CDCl_3$):

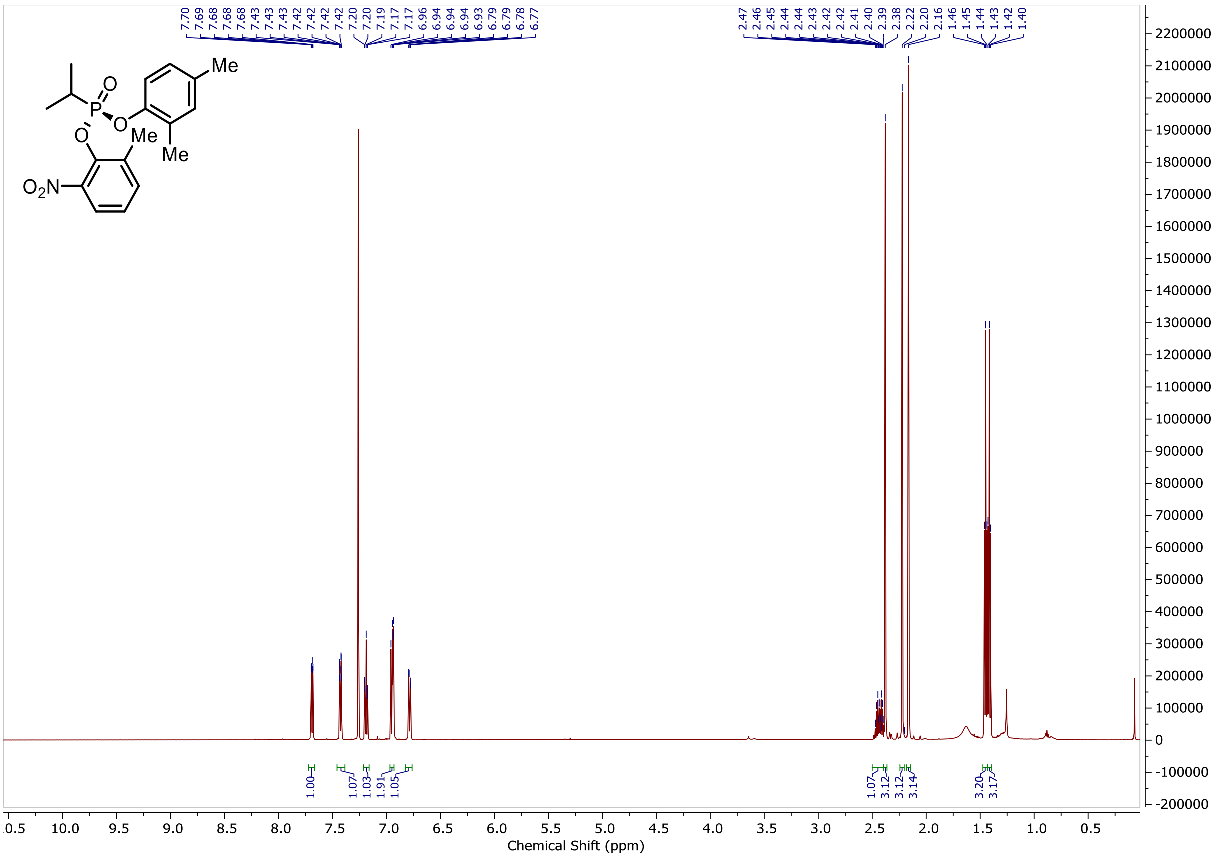

^{13}C NMR (157 MHz, $CDCl_3$):

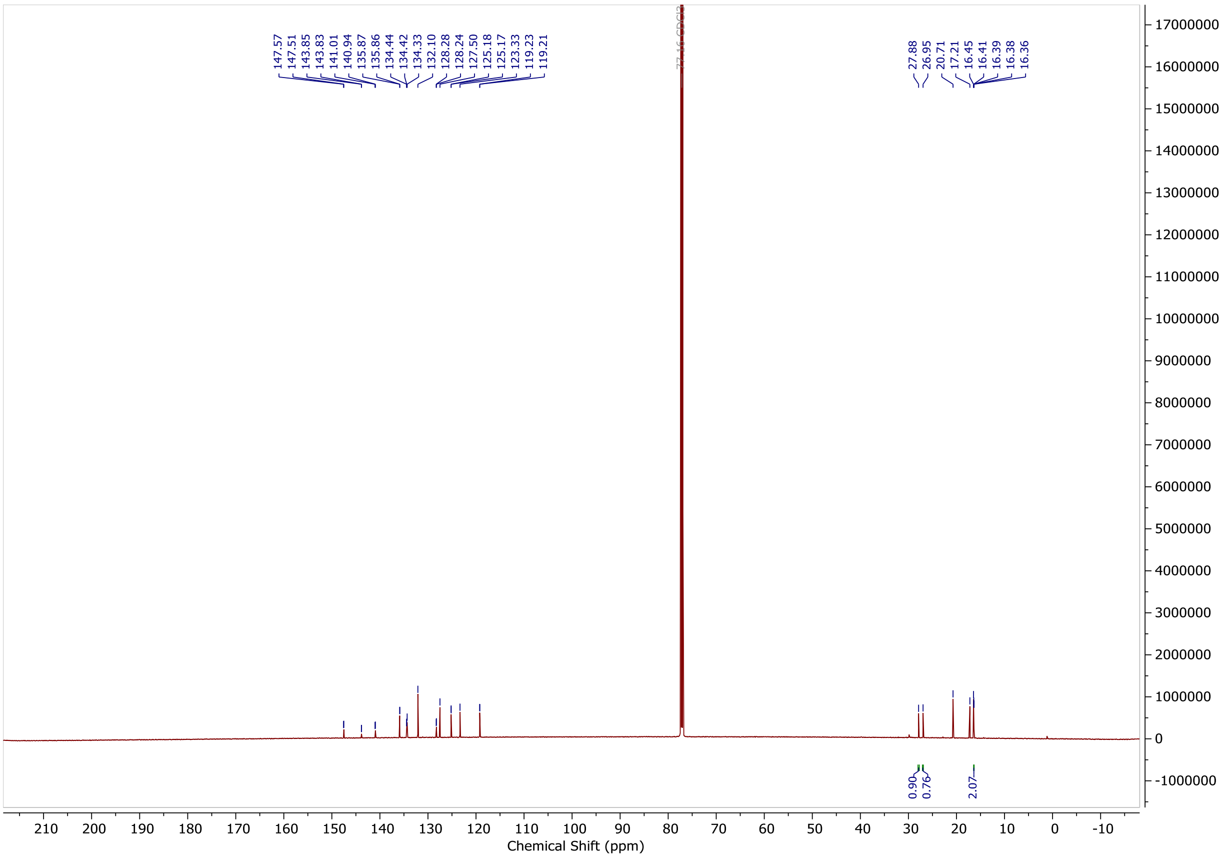

^{31}P NMR (162 MHz, CDCl$_3$):

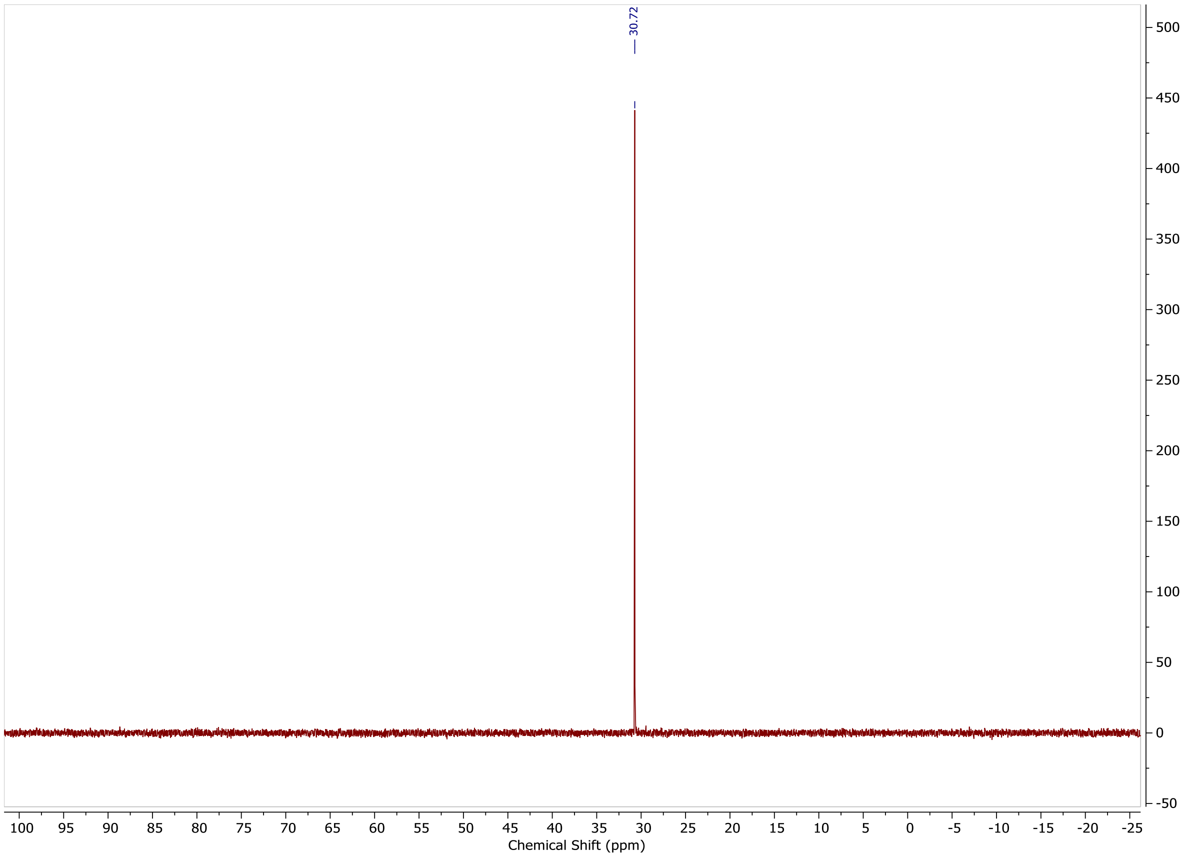

Compound **16**

^{1}H NMR (400 MHz, $CDCl_3$):

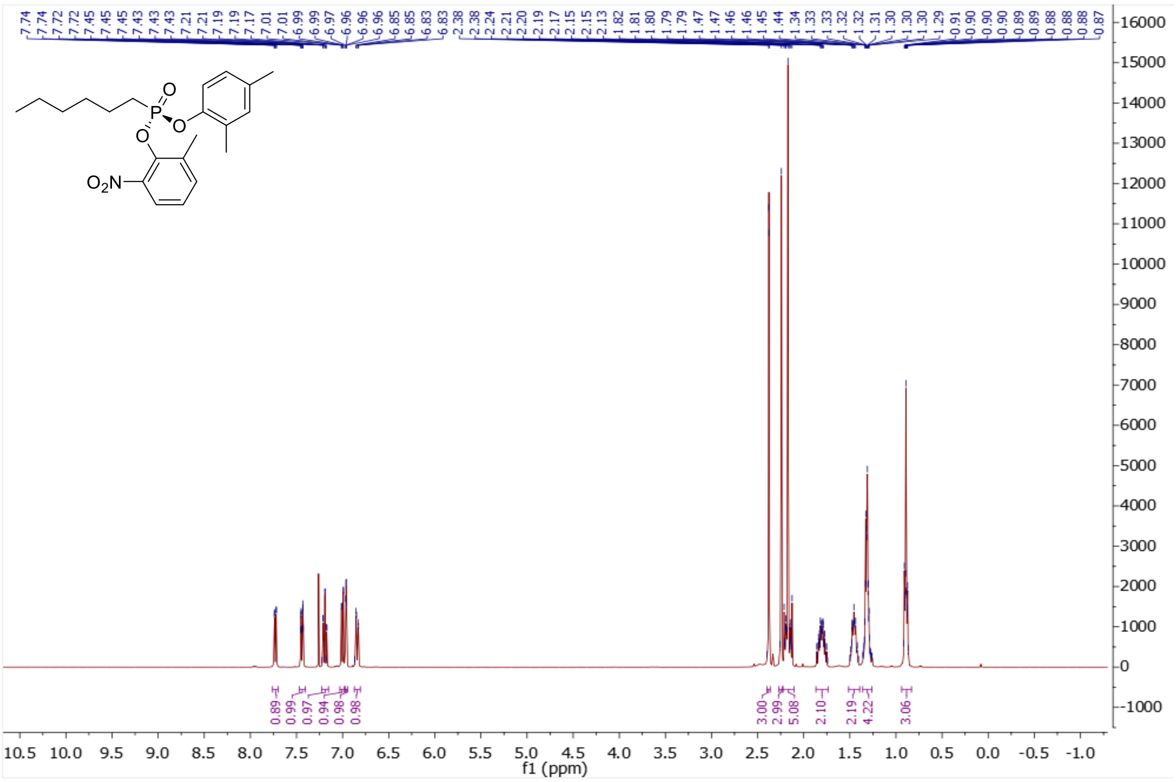

^{13}C NMR (101 MHz, $CDCl_3$):

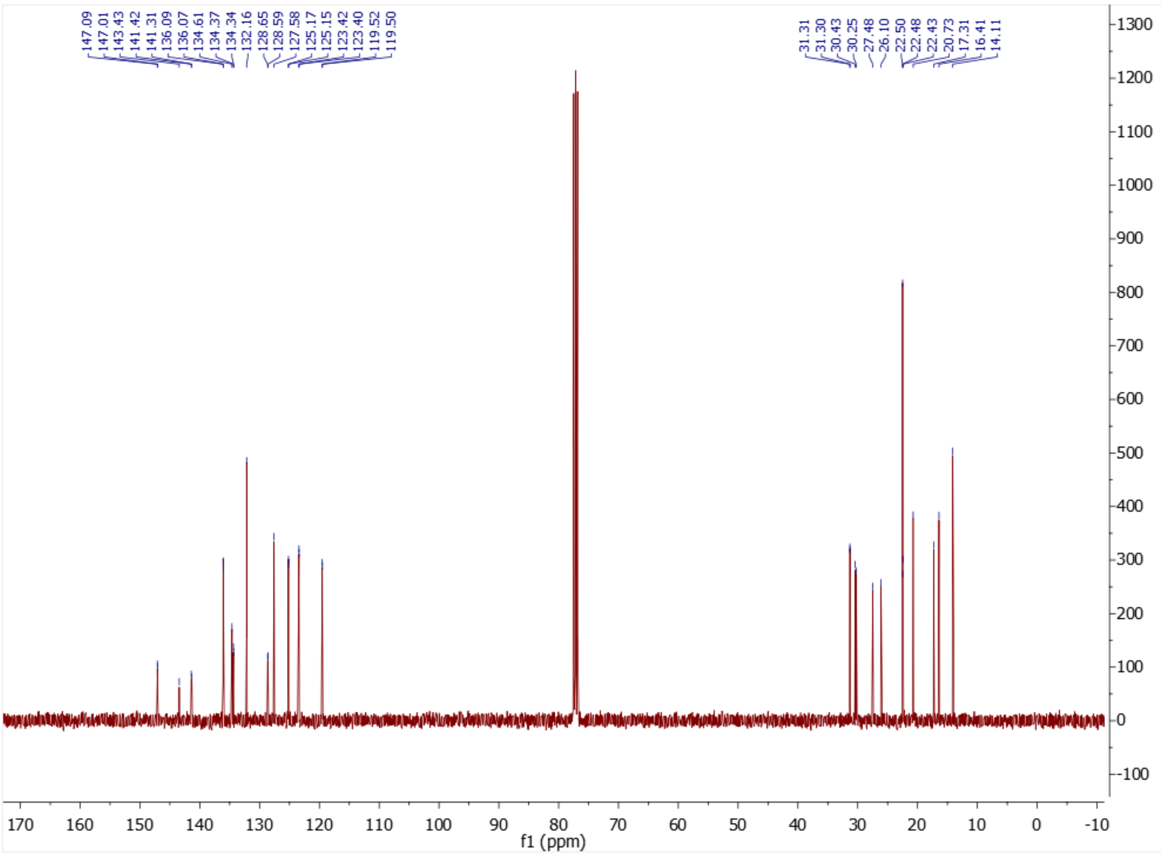

^{31}P NMR (162 MHz, CDCl$_3$):

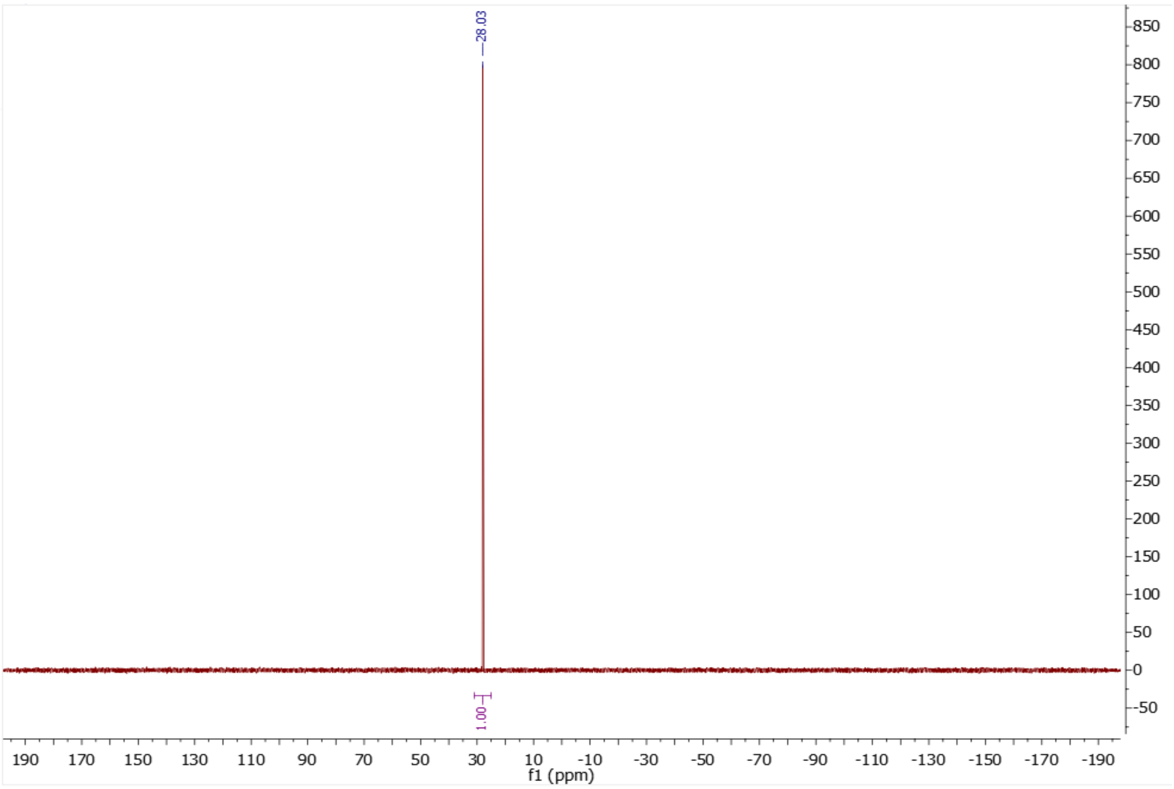

Compound **17**

^{1}H NMR (400 MHz, $CDCl_3$):

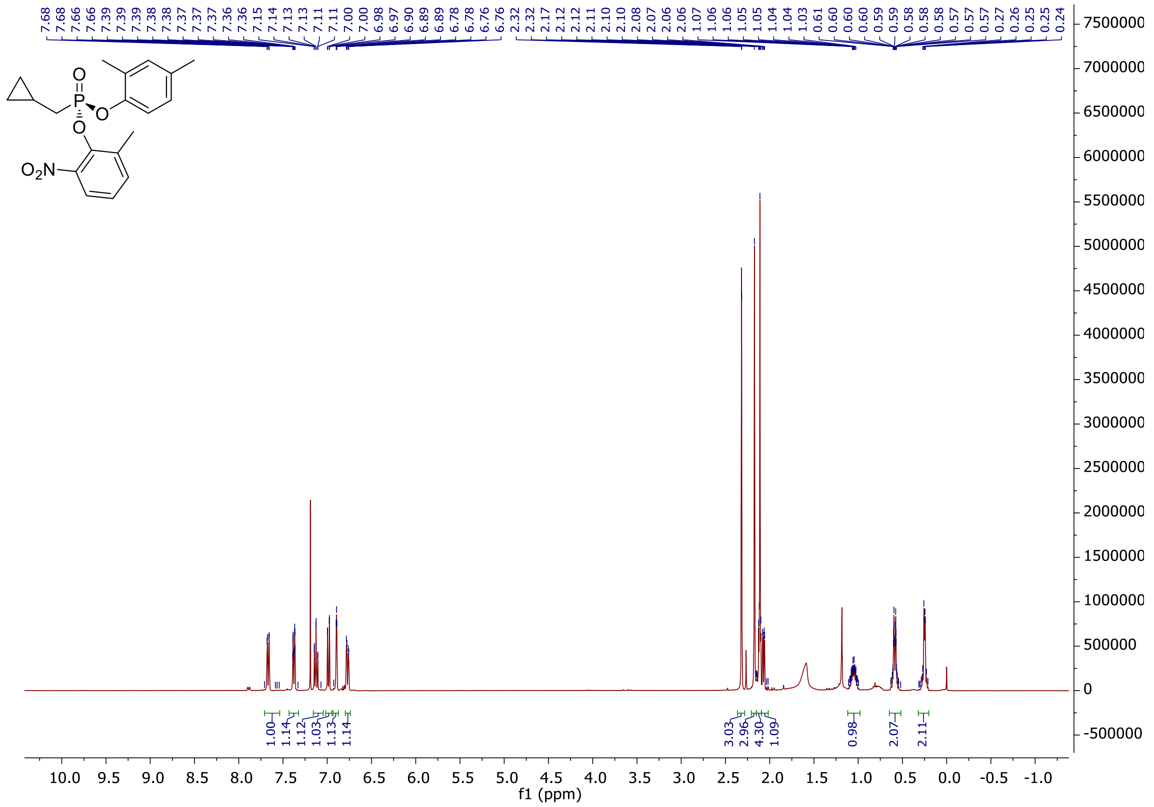

^{13}C NMR (101 MHz, $CDCl_3$):

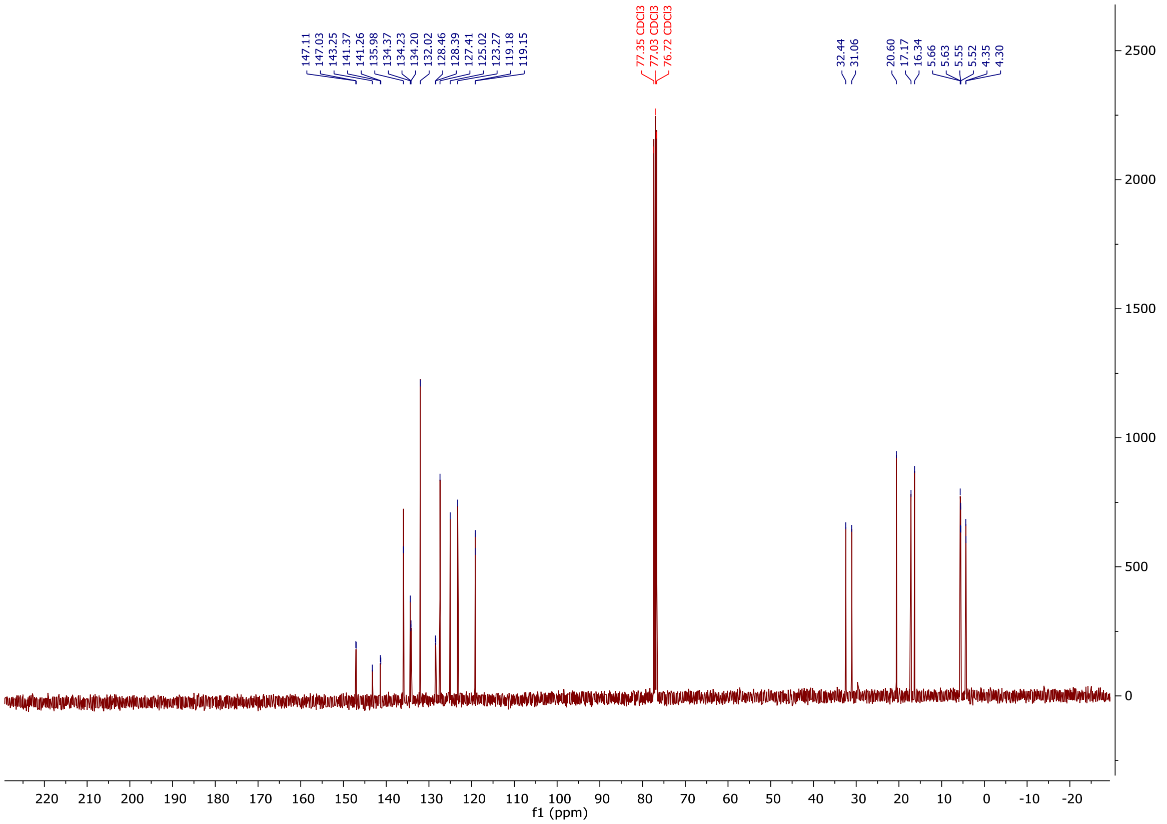

^{31}P NMR (162 MHz, CDCl$_3$):

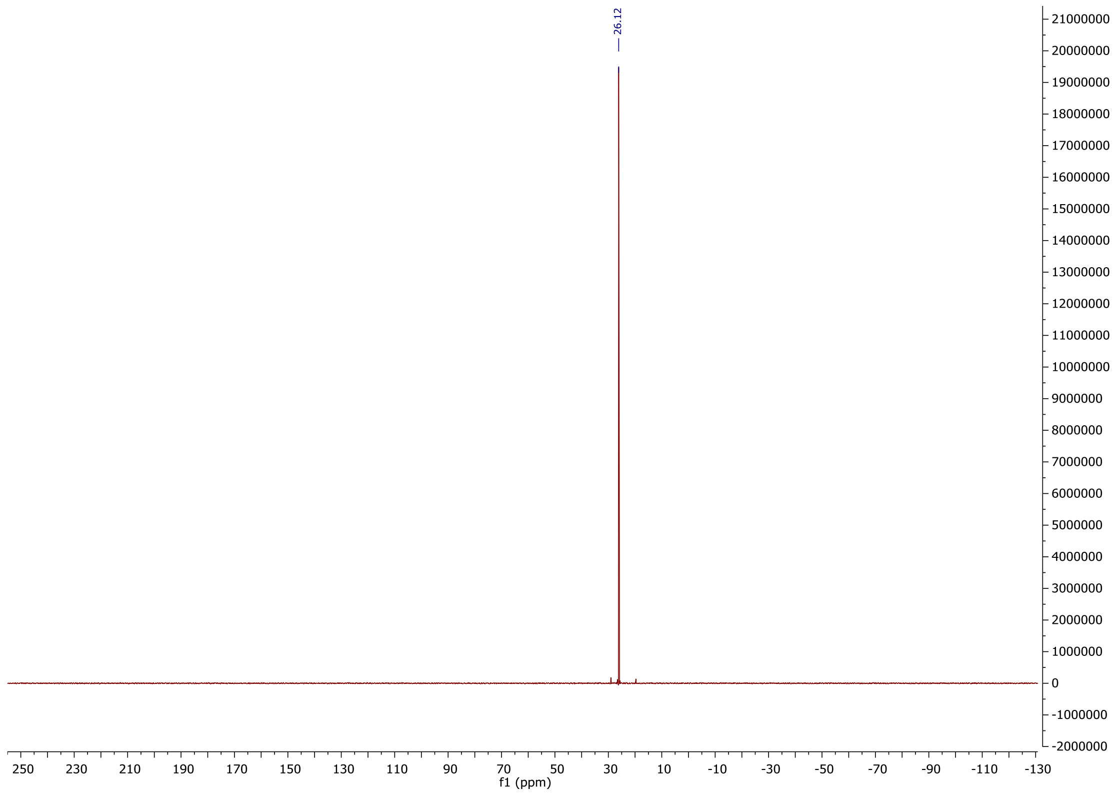

Compound **18**

^{1}H NMR (400 MHz, $CDCl_3$):

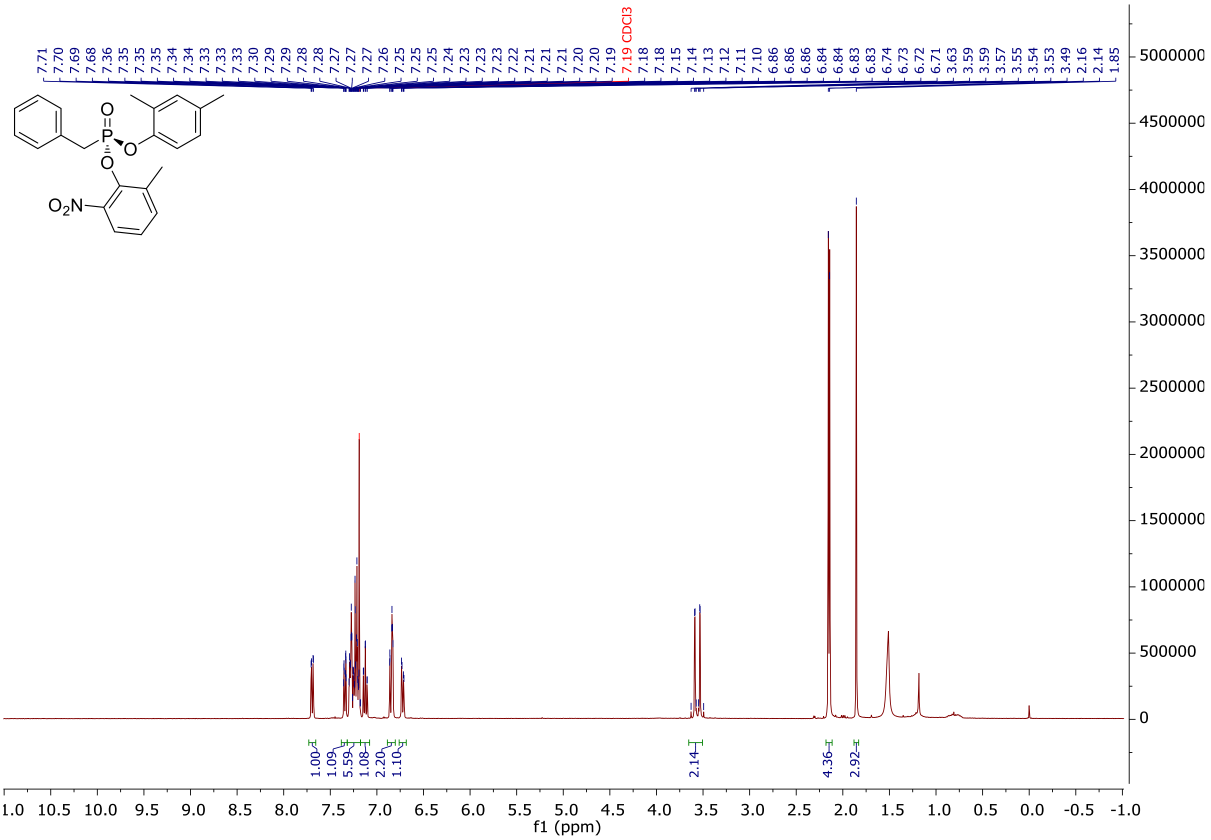

^{13}C NMR (101 MHz, $CDCl_3$):

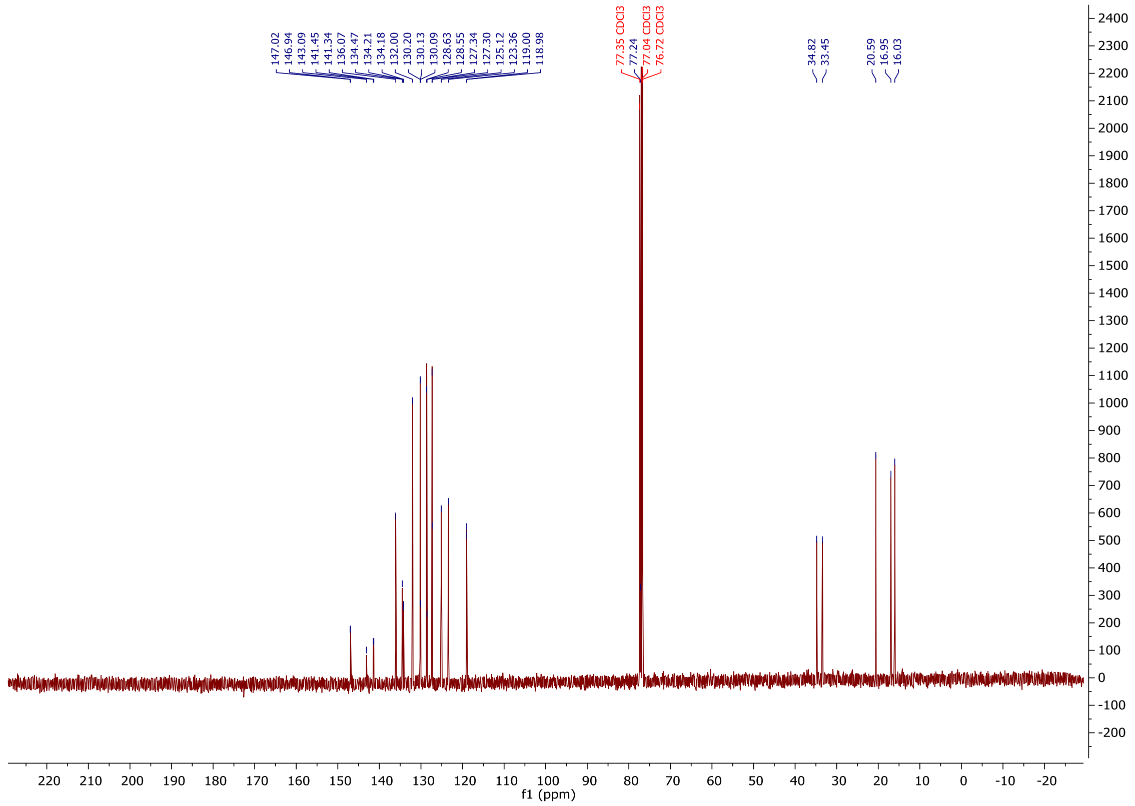

^{31}P NMR (162 MHz, $CDCl_3$):

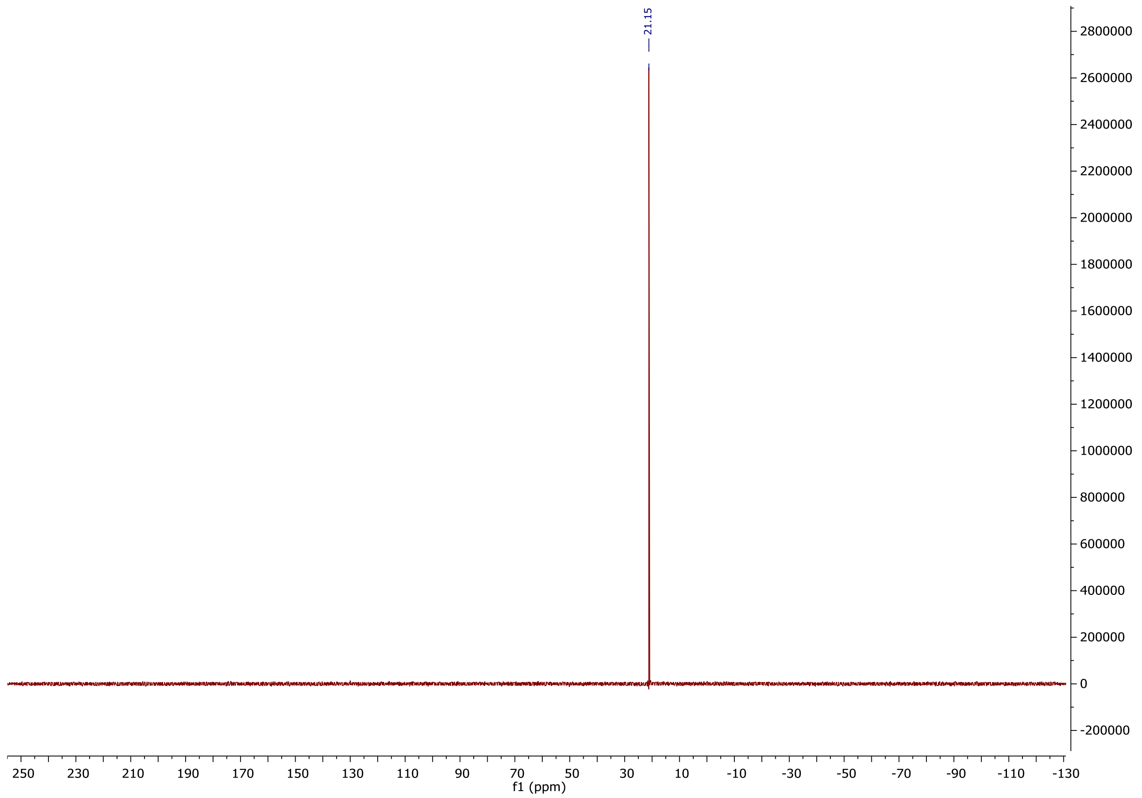

Compound **19**

^{1}H NMR (400 MHz, CDCl$_3$):

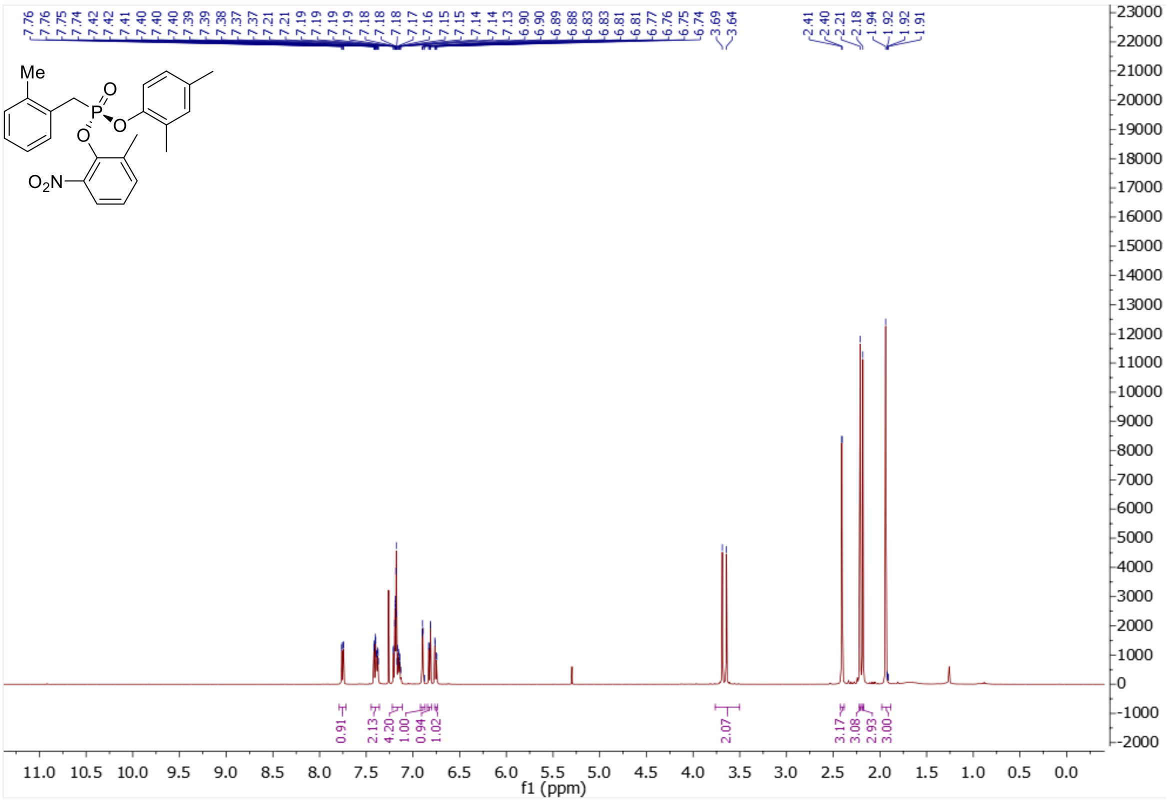

^{13}C NMR (101 MHz, CDCl$_3$):

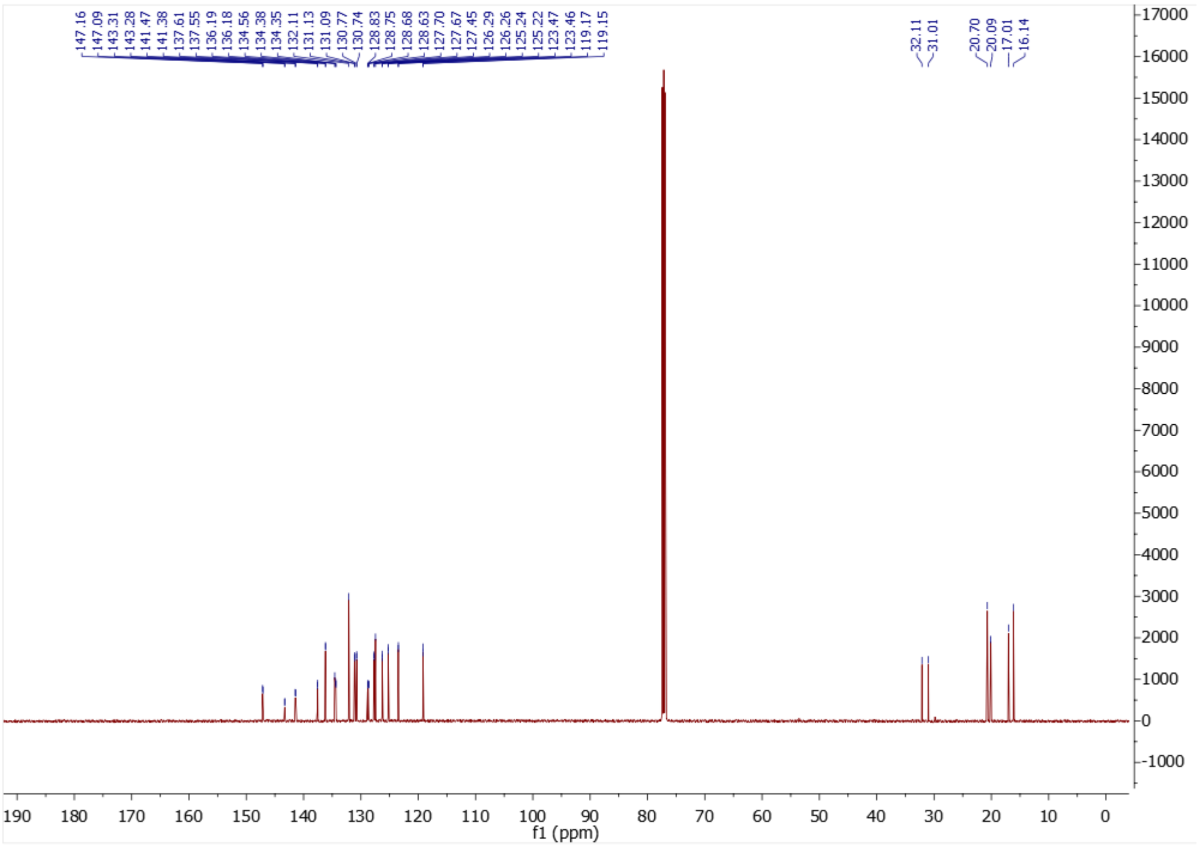

^{31}P NMR (162 MHz, CDCl$_3$):

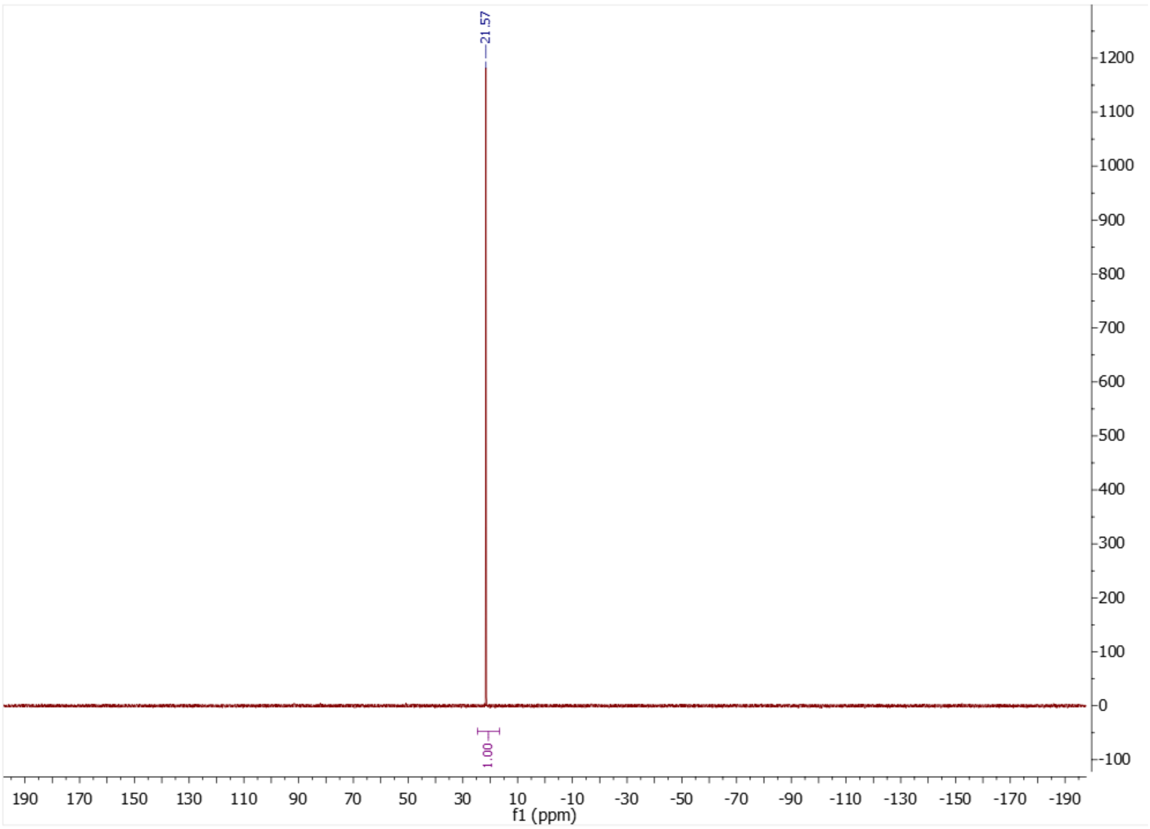

Compound **20**

^{1}H NMR (400 MHz, $CDCl_3$):

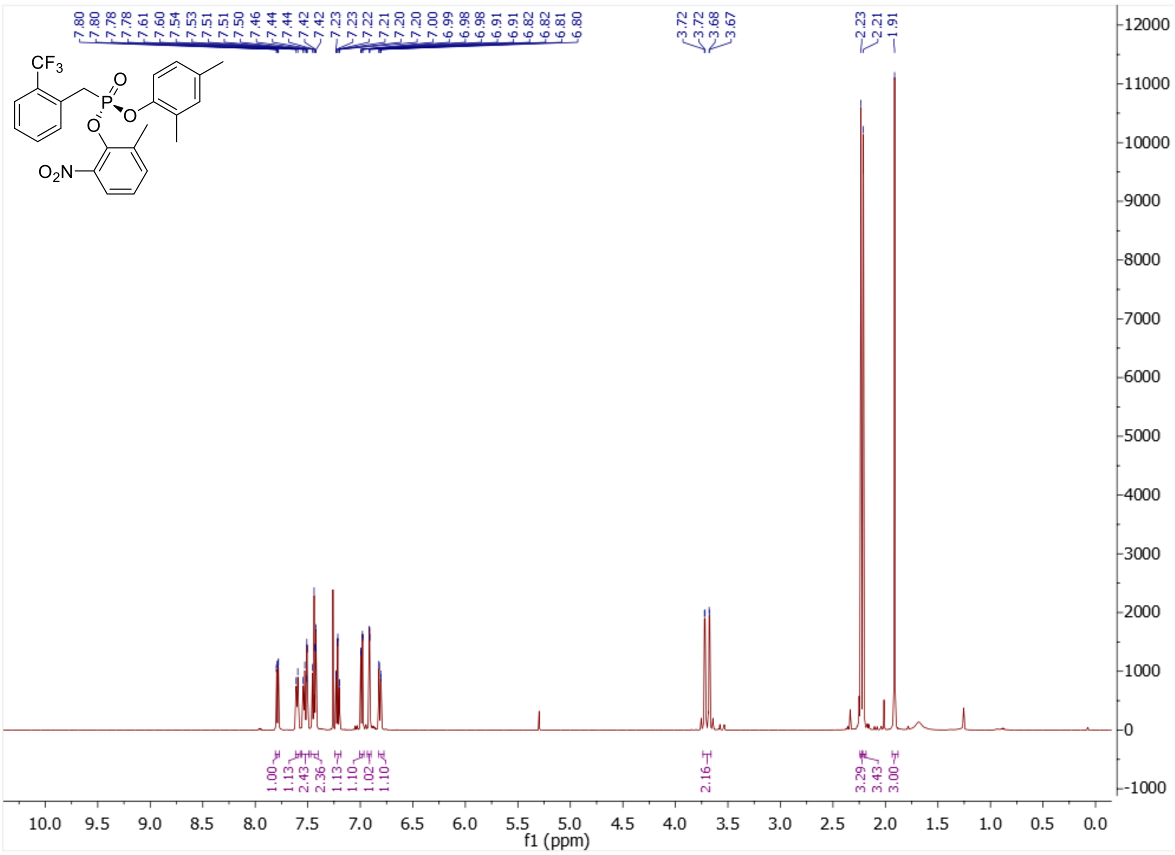

^{13}C NMR (101 MHz, $CDCl_3$):

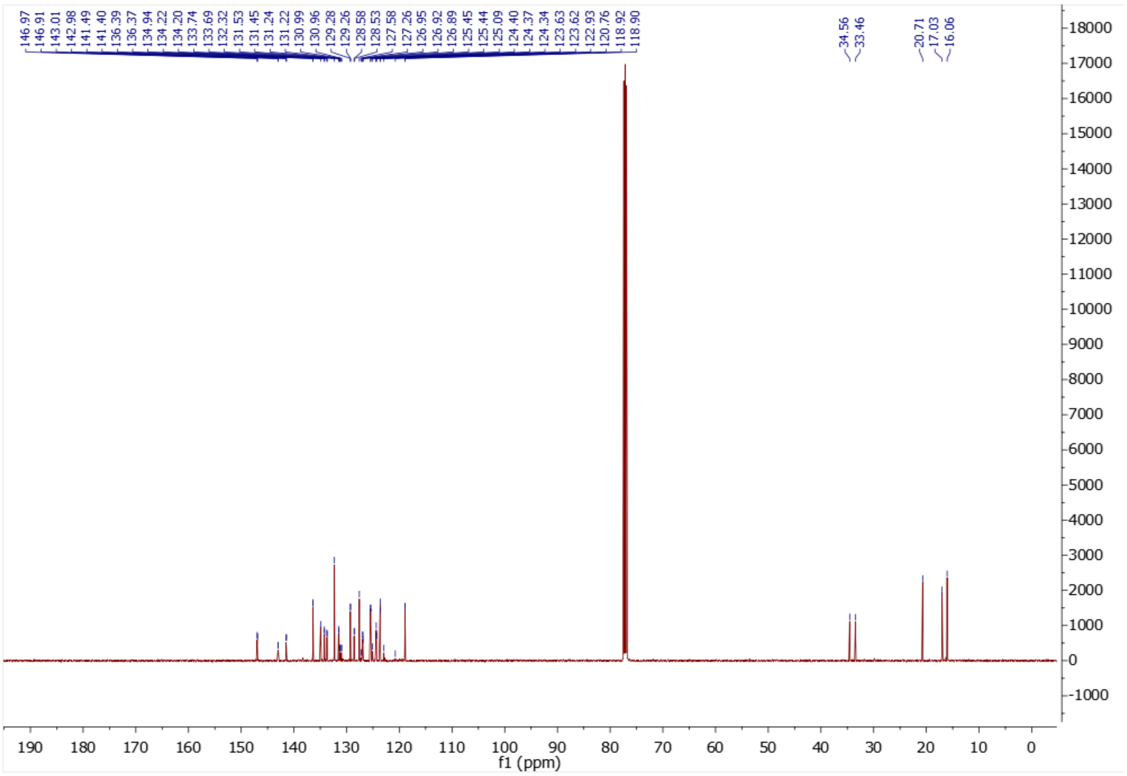

^{19}**F NMR (377 MHz, $CDCl_3$):**

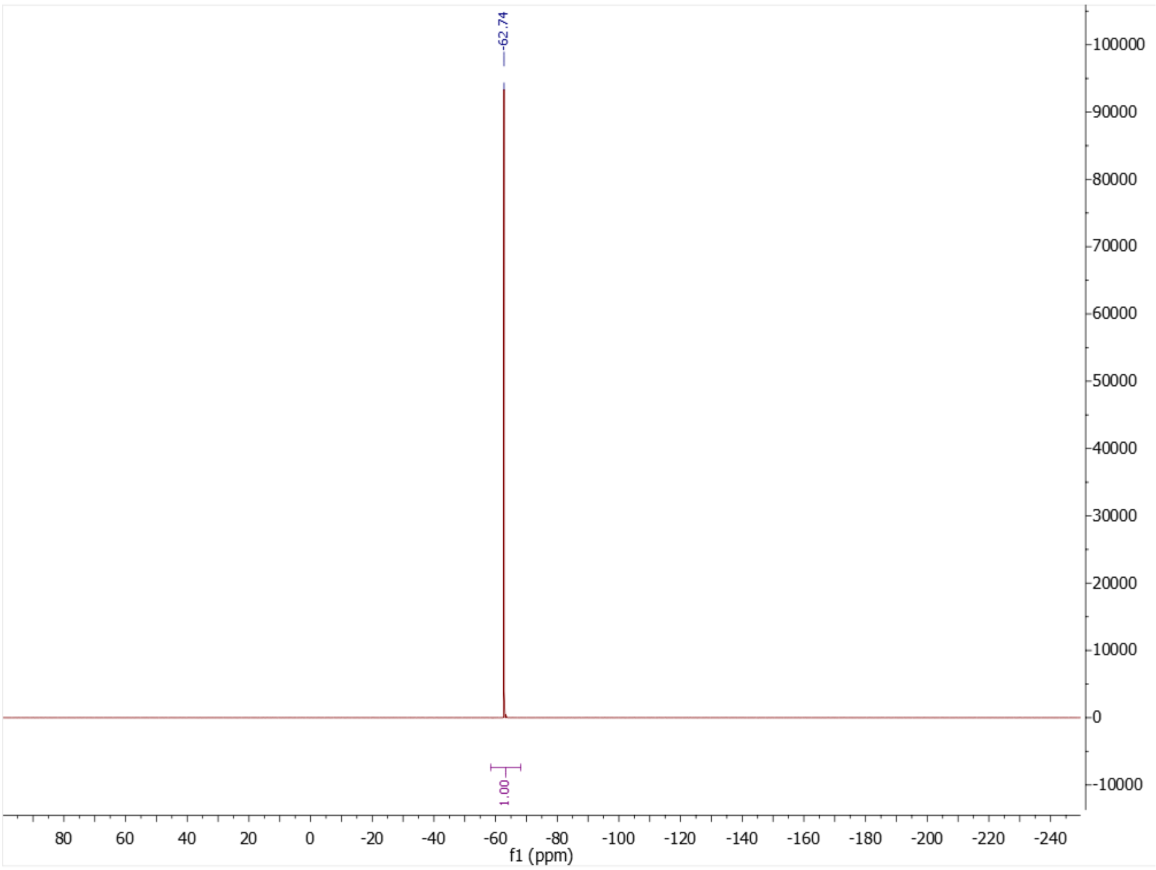

^{31}**P NMR (162 MHz, $CDCl_3$):**

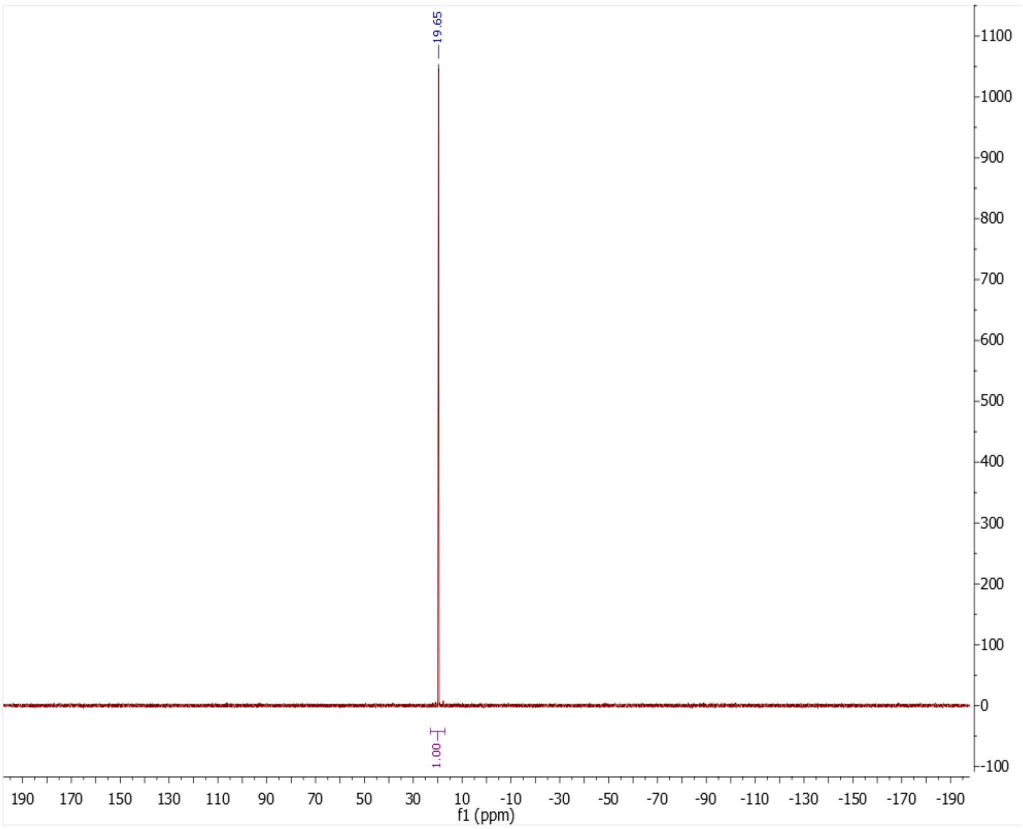

Compound **21**

^{1}H NMR (400 MHz, $CDCl_3$):

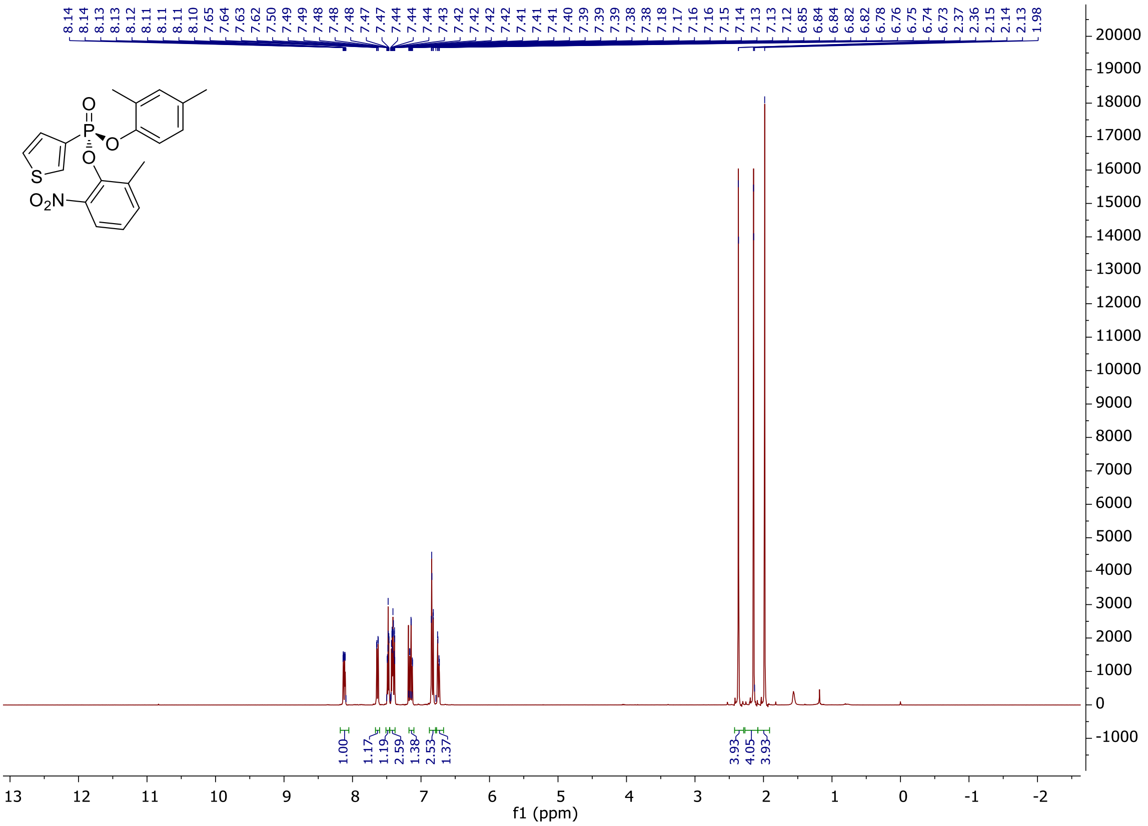

^{13}C NMR (101 MHz, $CDCl_3$):

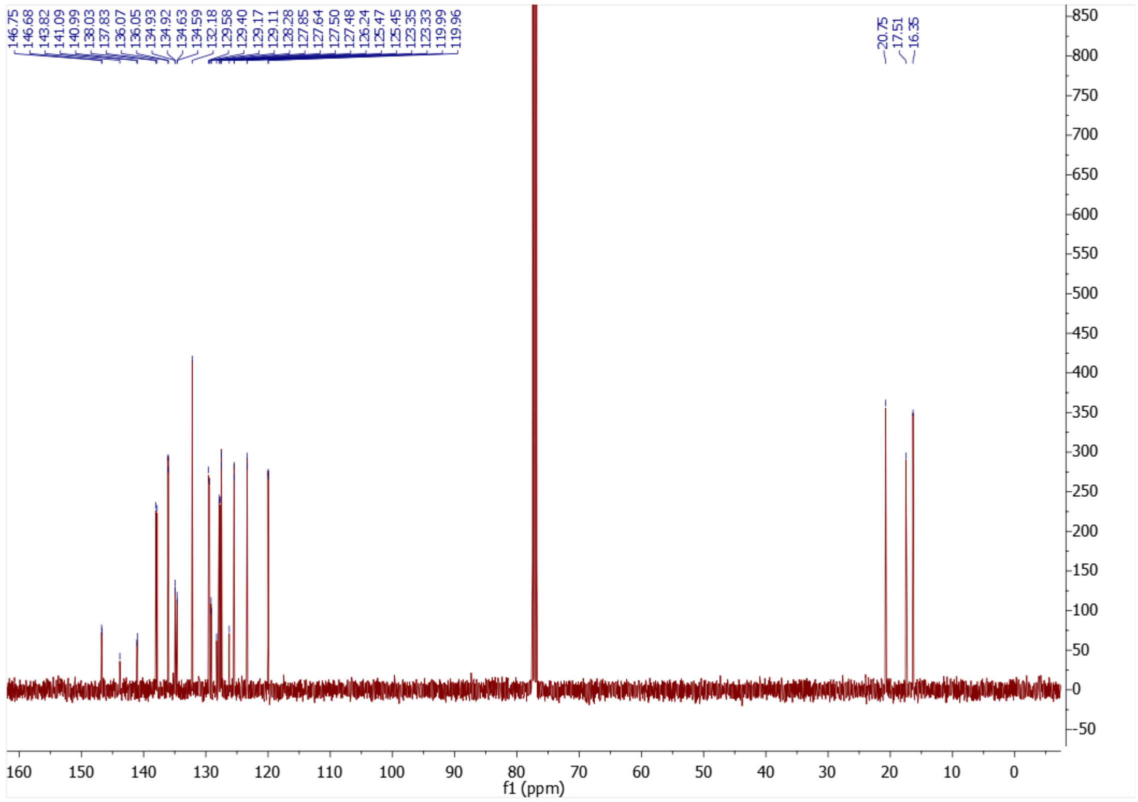

^{31}P NMR (162 MHz, $CDCl_3$):

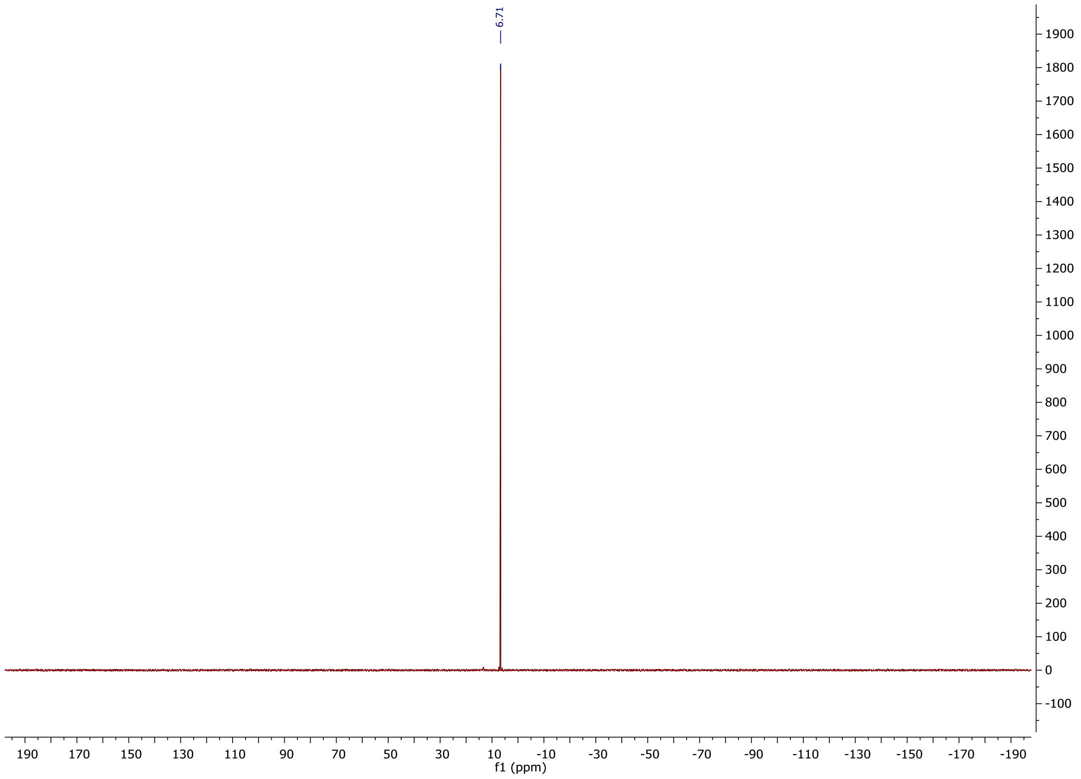

Compound **22**

^{1}H NMR (400 MHz, $CDCl_3$):

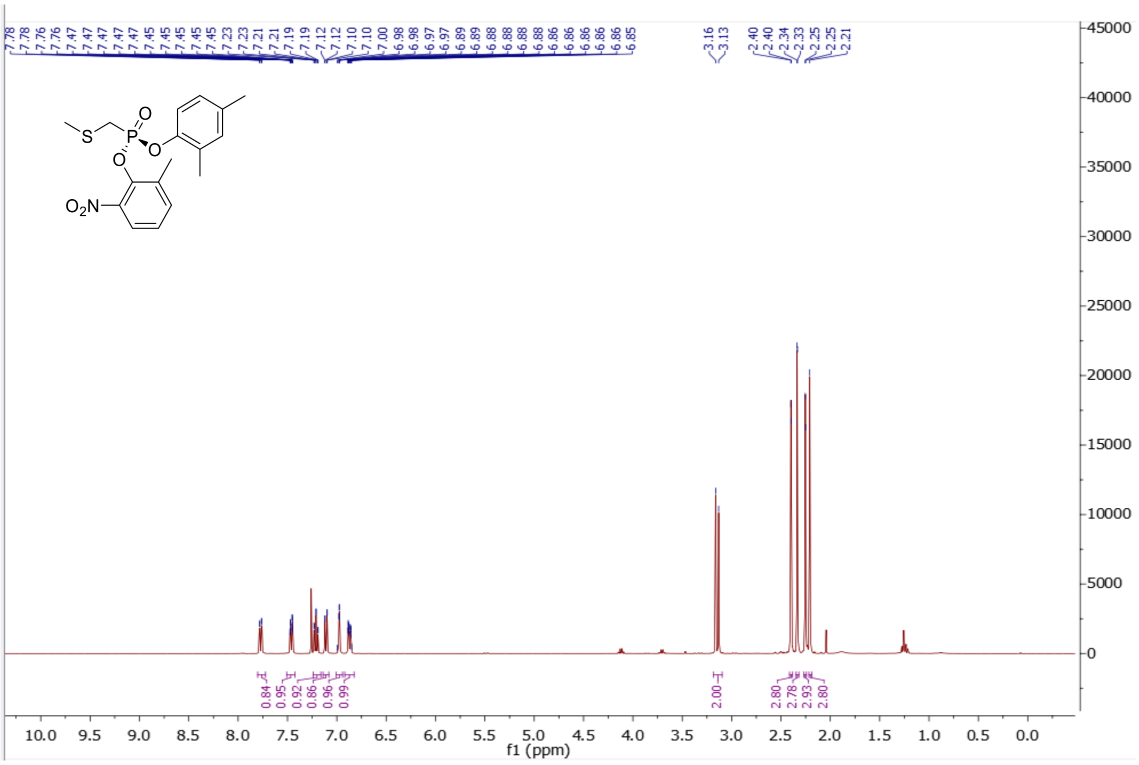

^{13}C NMR (101 MHz, $CDCl_3$):

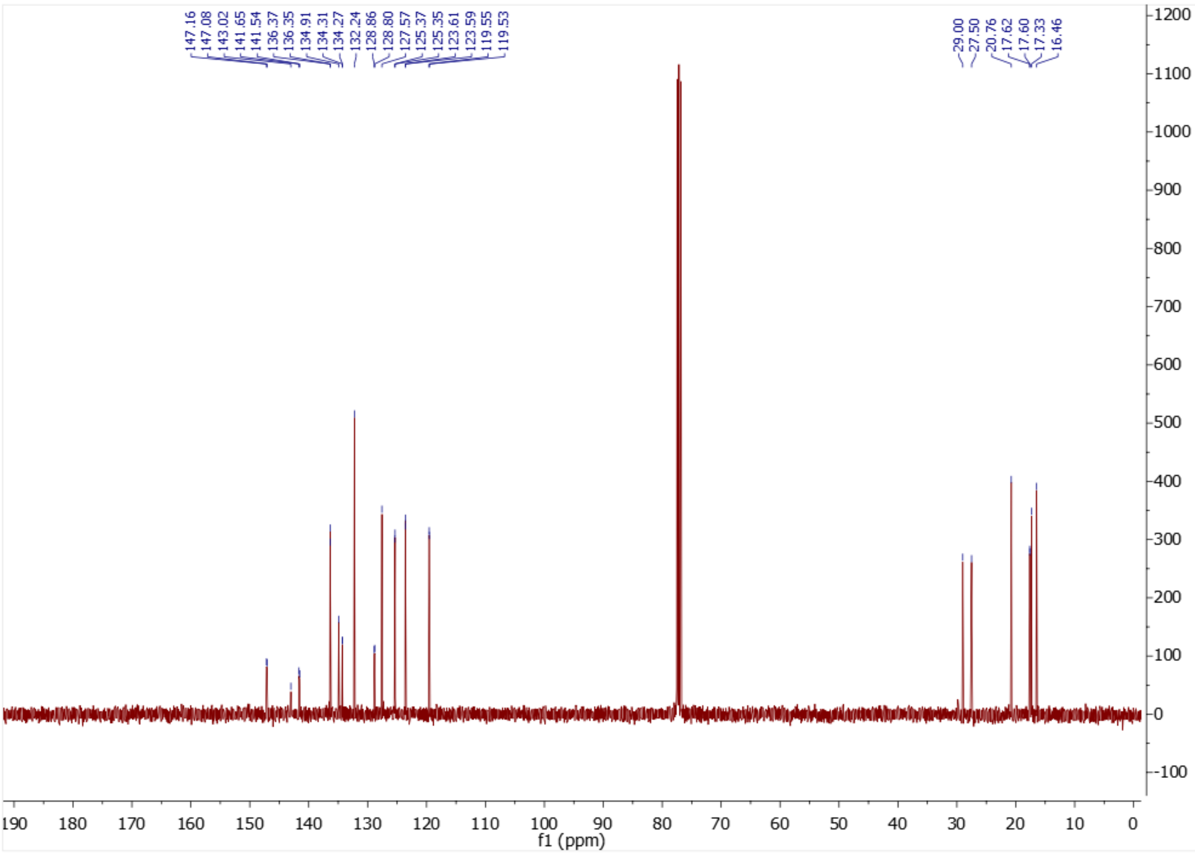

^{31}P NMR (162 MHz, $CDCl_3$):

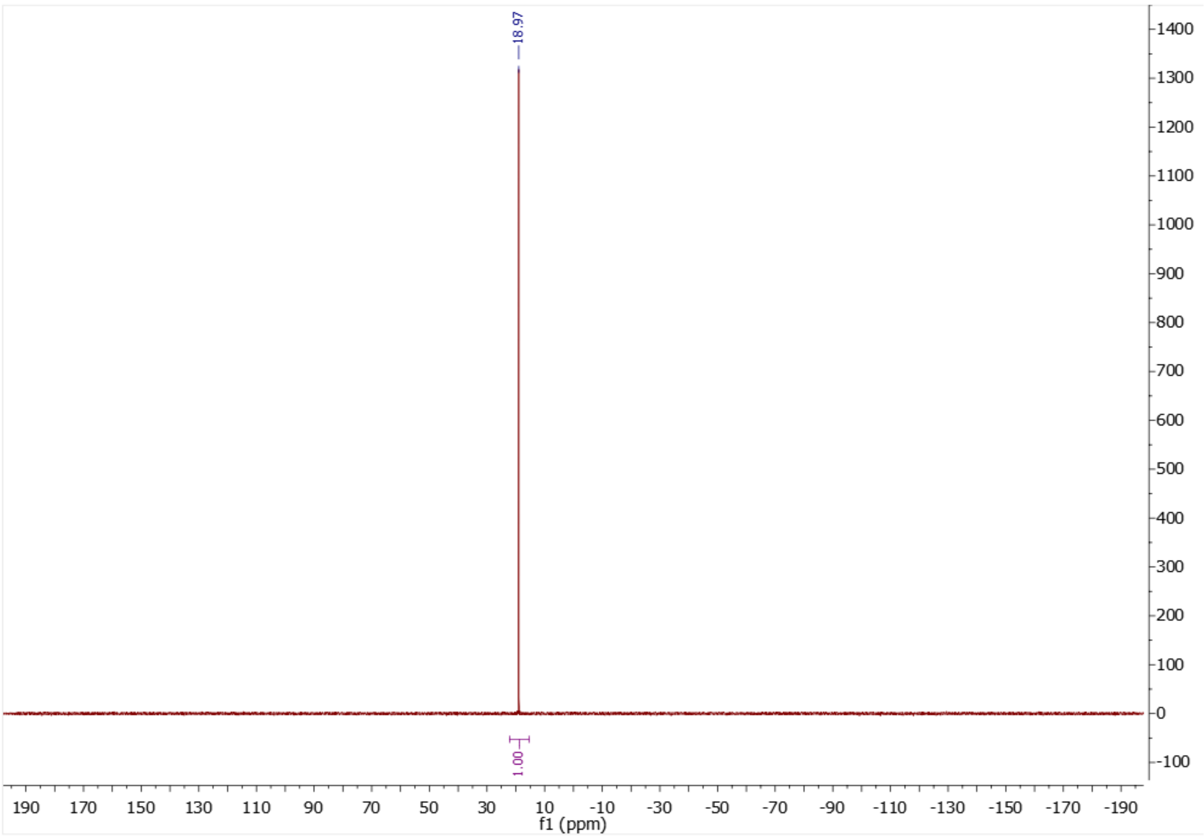

Compound **23**

^{1}H NMR (400 MHz, $CDCl_3$):

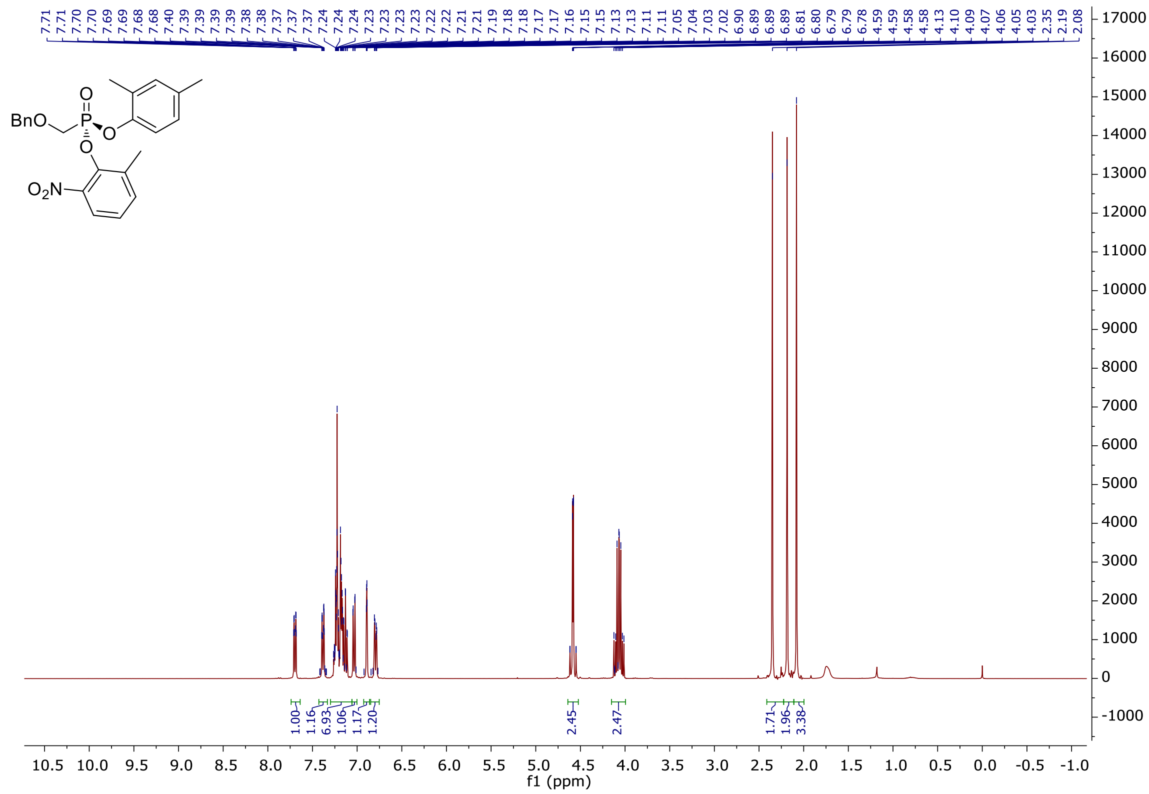

^{13}C NMR (101 MHz, $CDCl_3$):

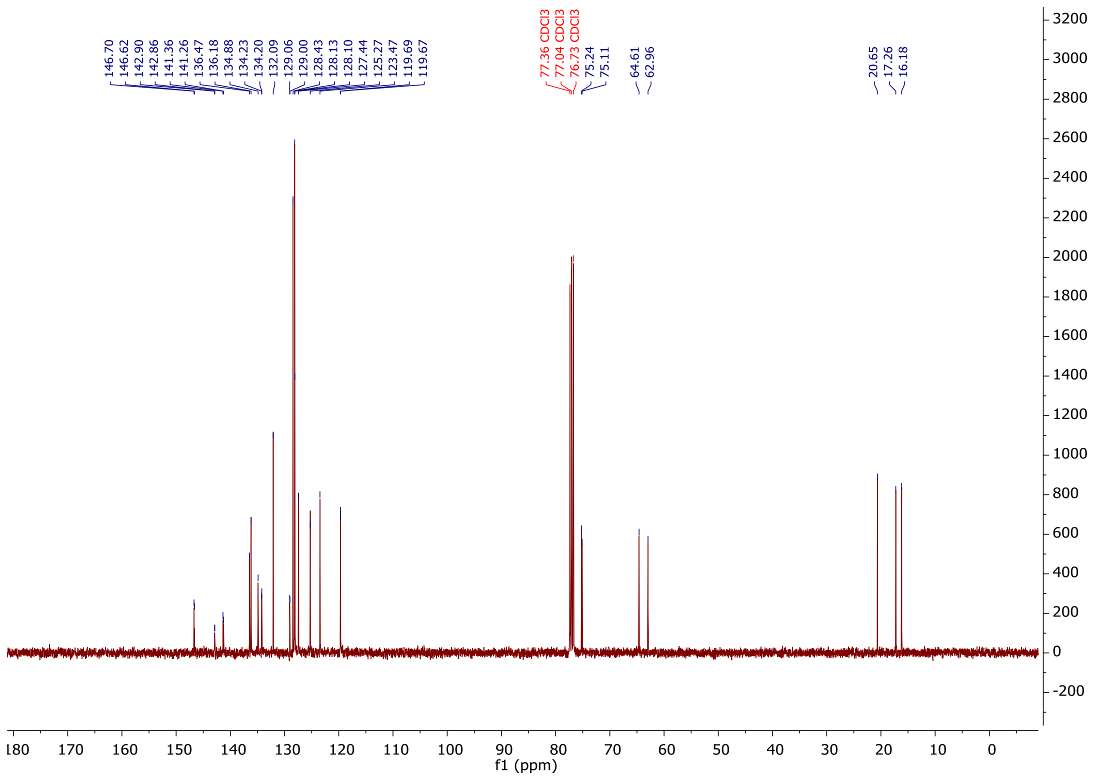

^{31}P NMR (162 MHz, CDCl$_3$):

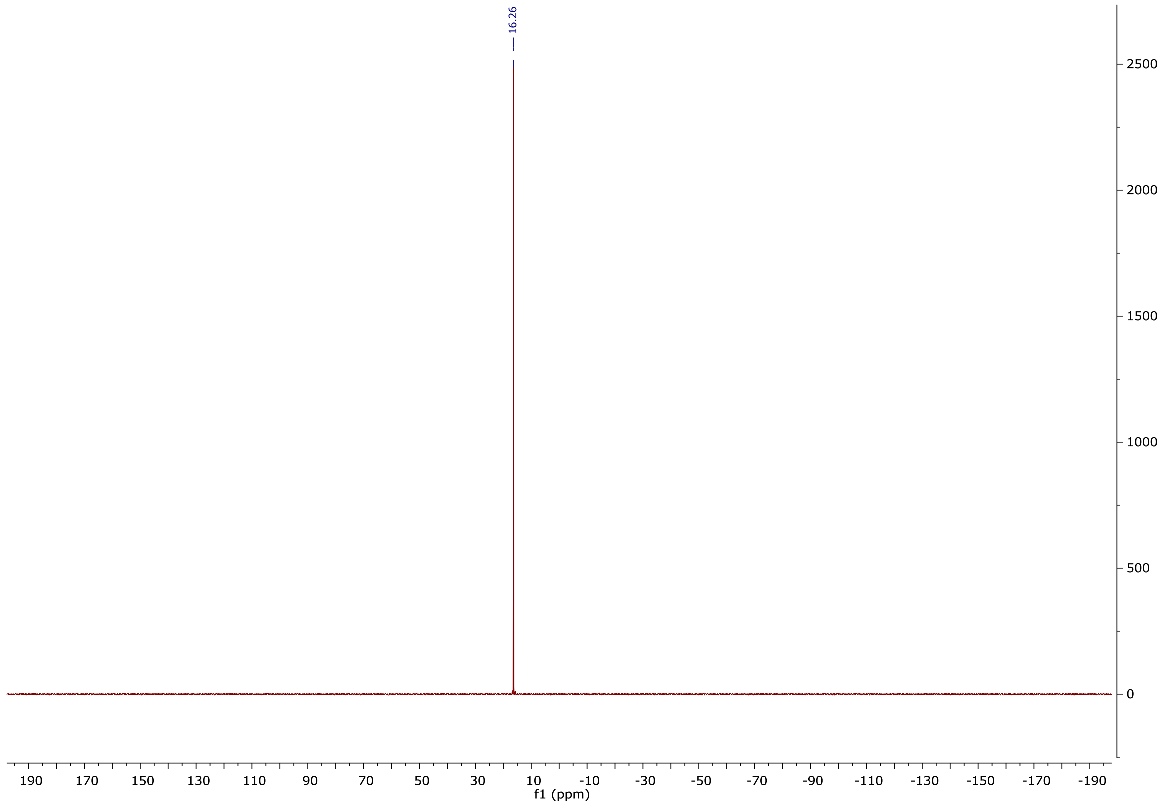

Compound **24**

^{1}H NMR (400 MHz, $CDCl_3$):

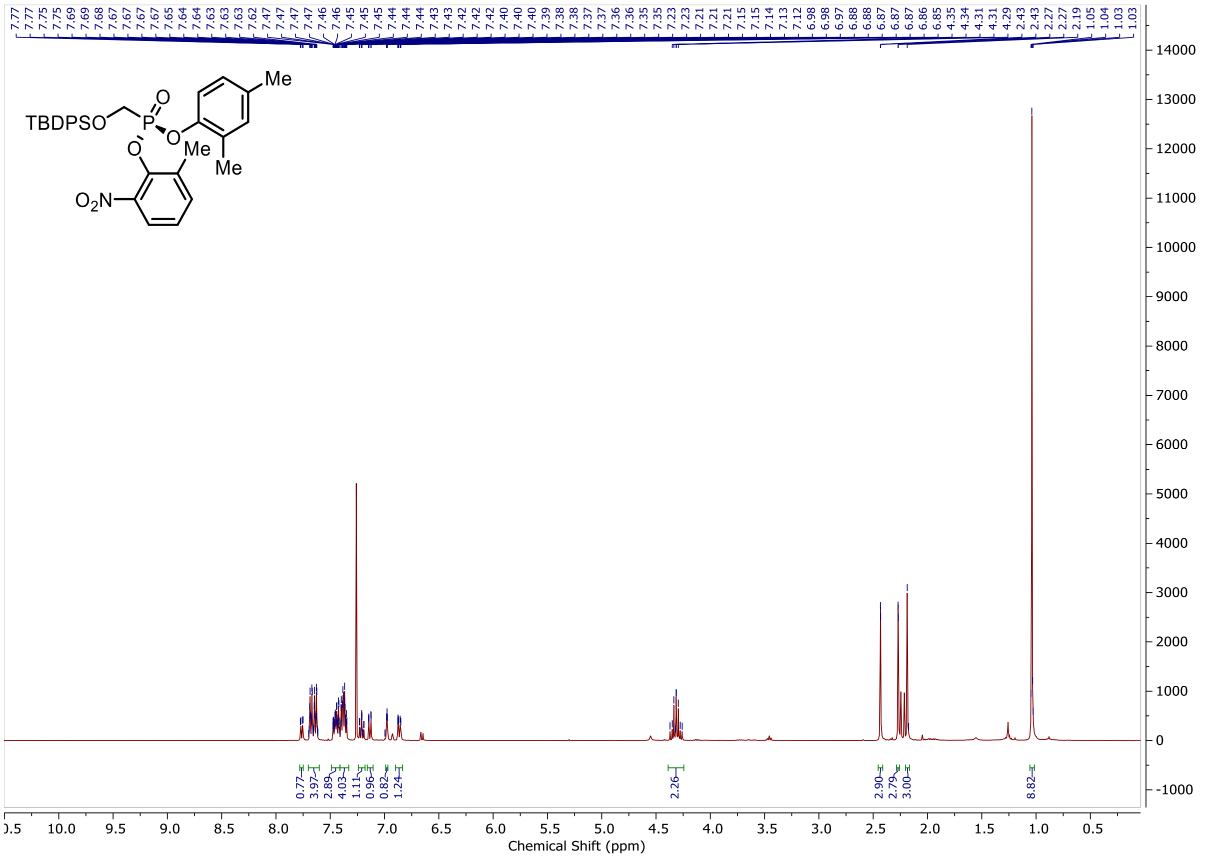

^{13}C NMR (157 MHz, $CDCl_3$):

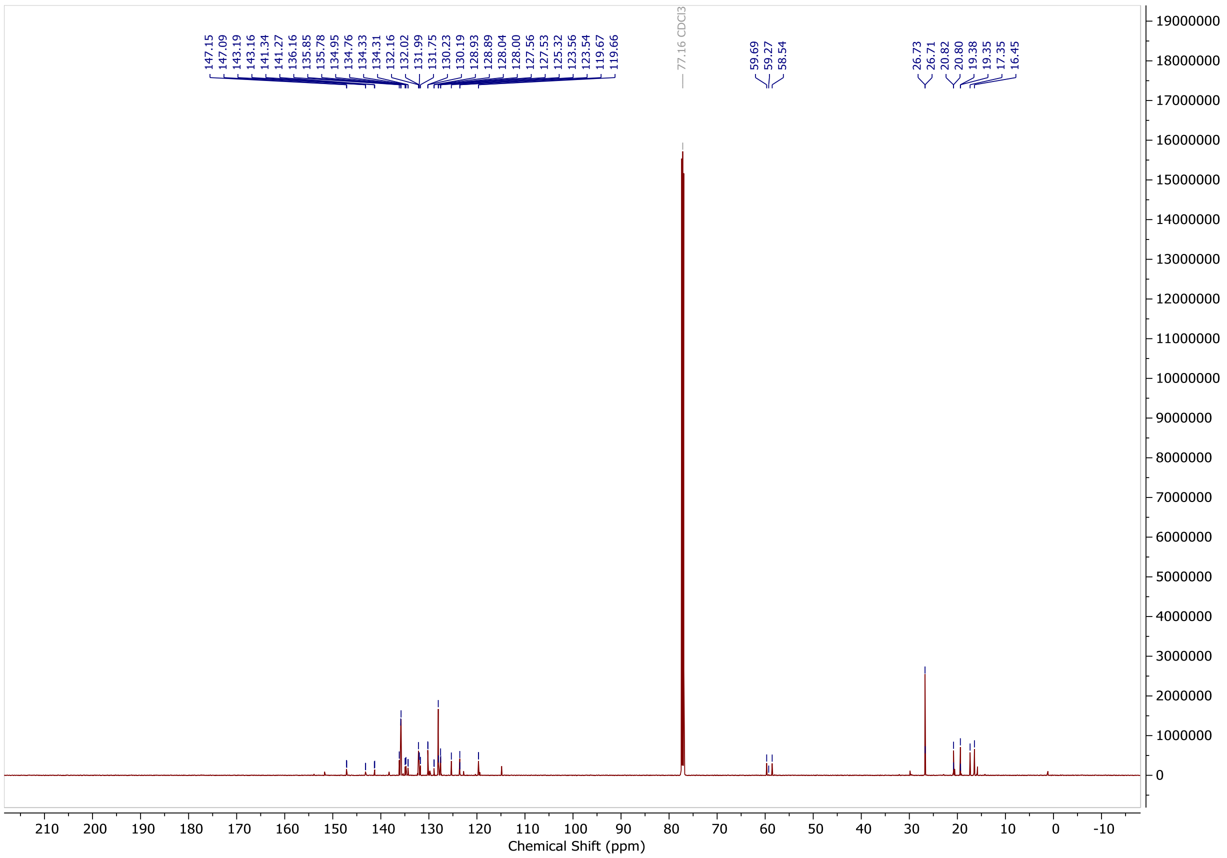

^{31}P NMR (162 MHz, $CDCl_3$):

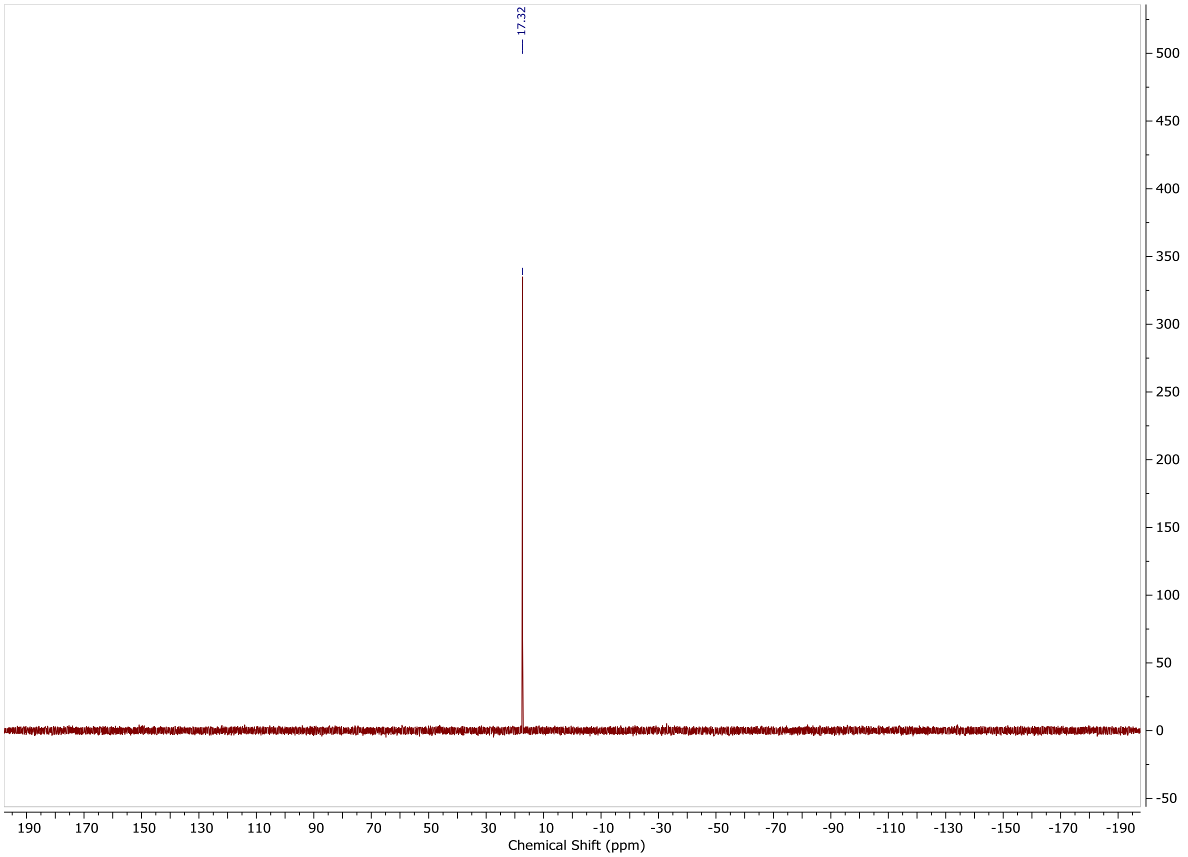

Compound **25**

^{1}H NMR (400 MHz, $CDCl_3$):

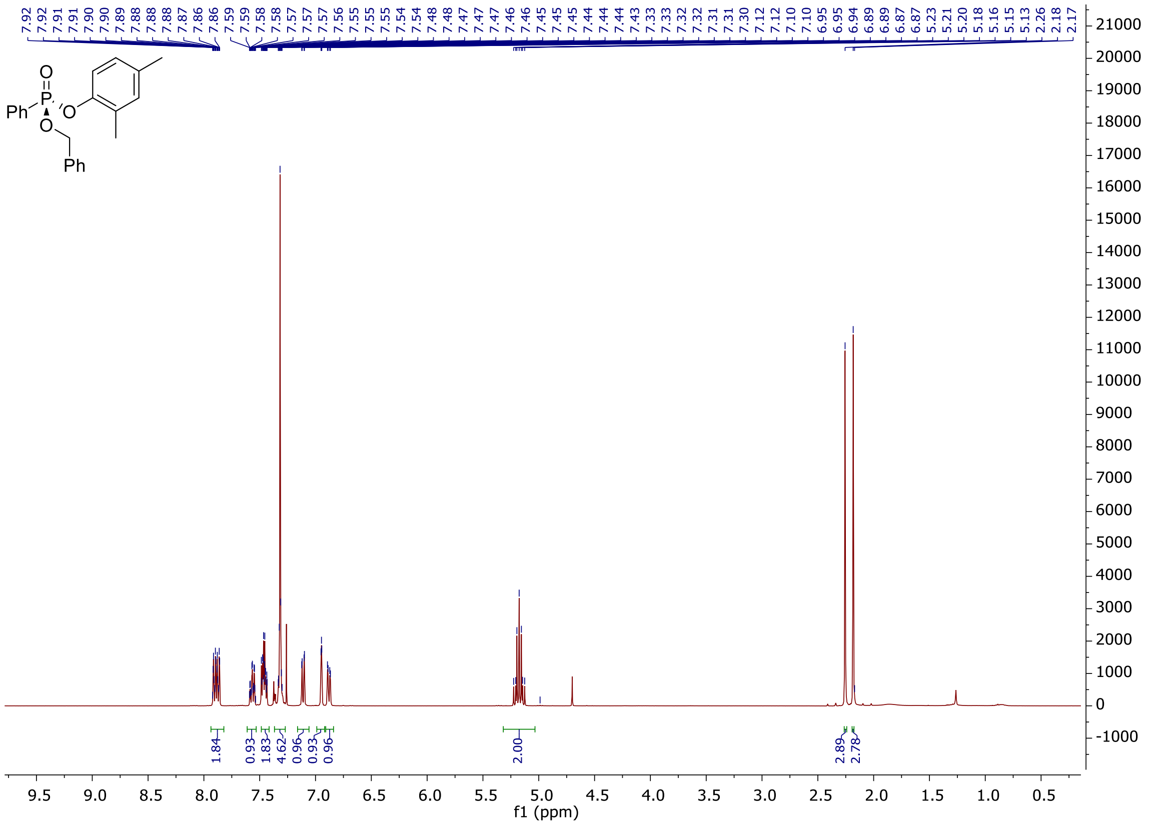

^{13}C NMR (101 MHz, $CDCl_3$):

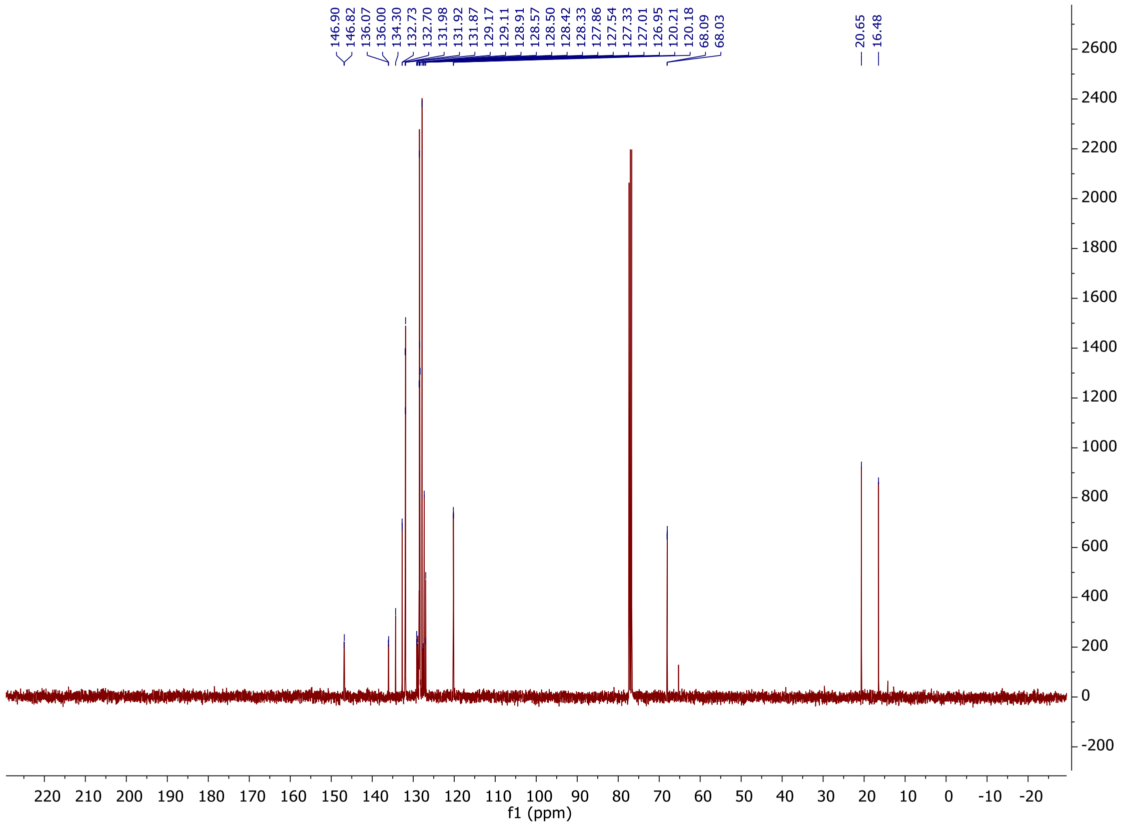

^{31}P NMR (162 MHz, CDCl$_3$):

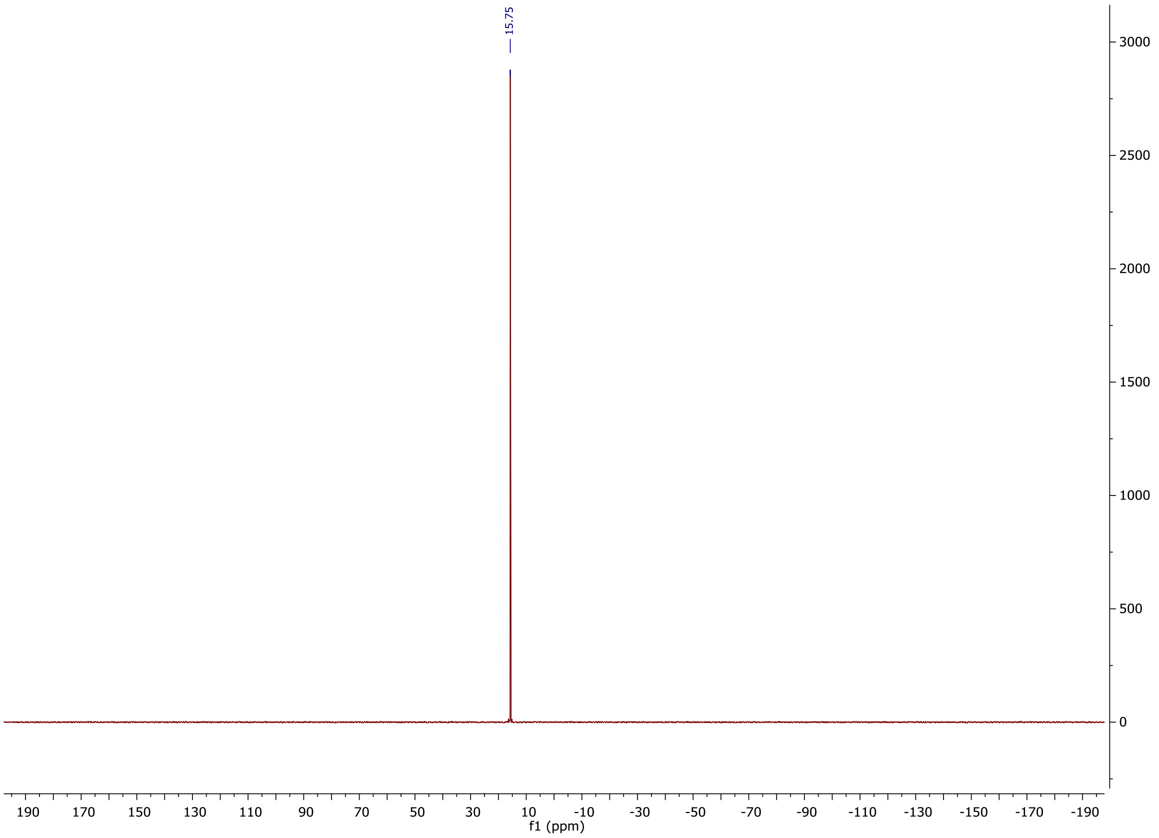

Compound **26**

^{1}H NMR (500 MHz, DMSO-d_6):

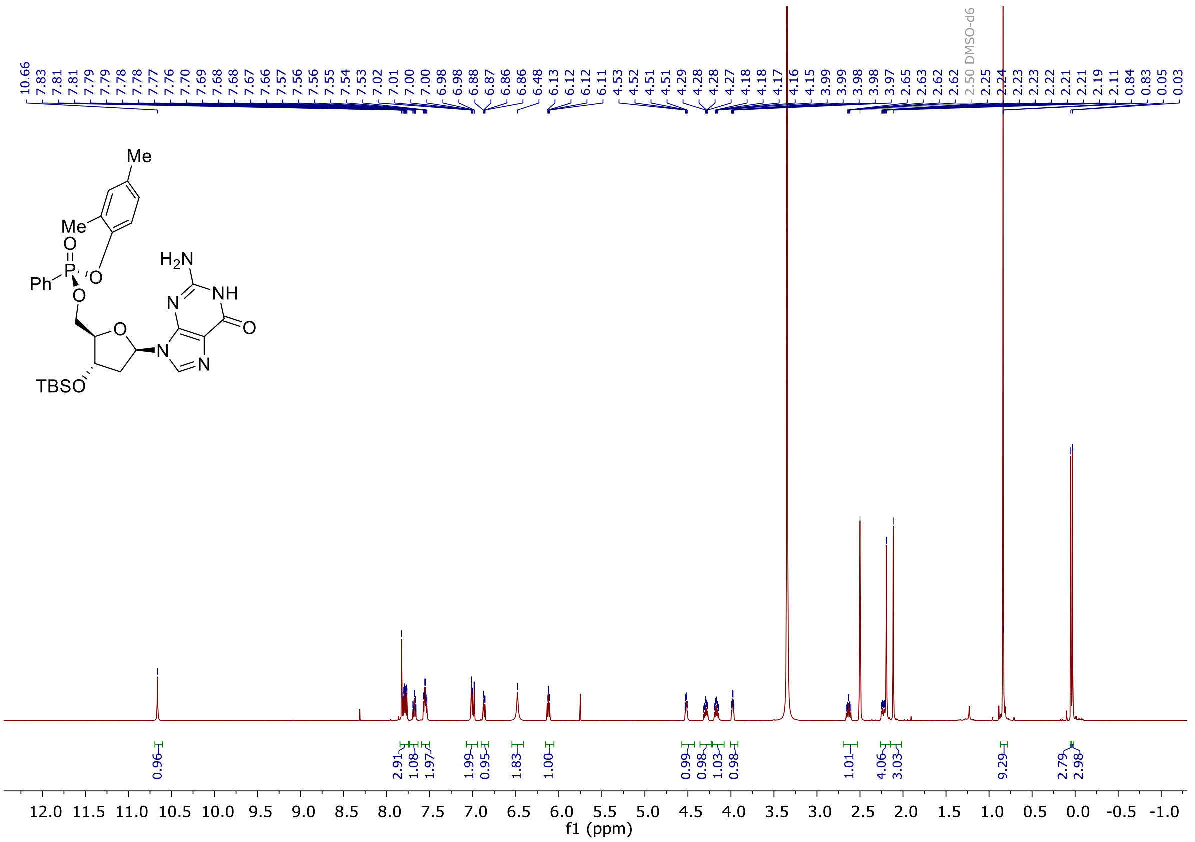

^{13}C NMR (126 MHz, DMSO-d_6):

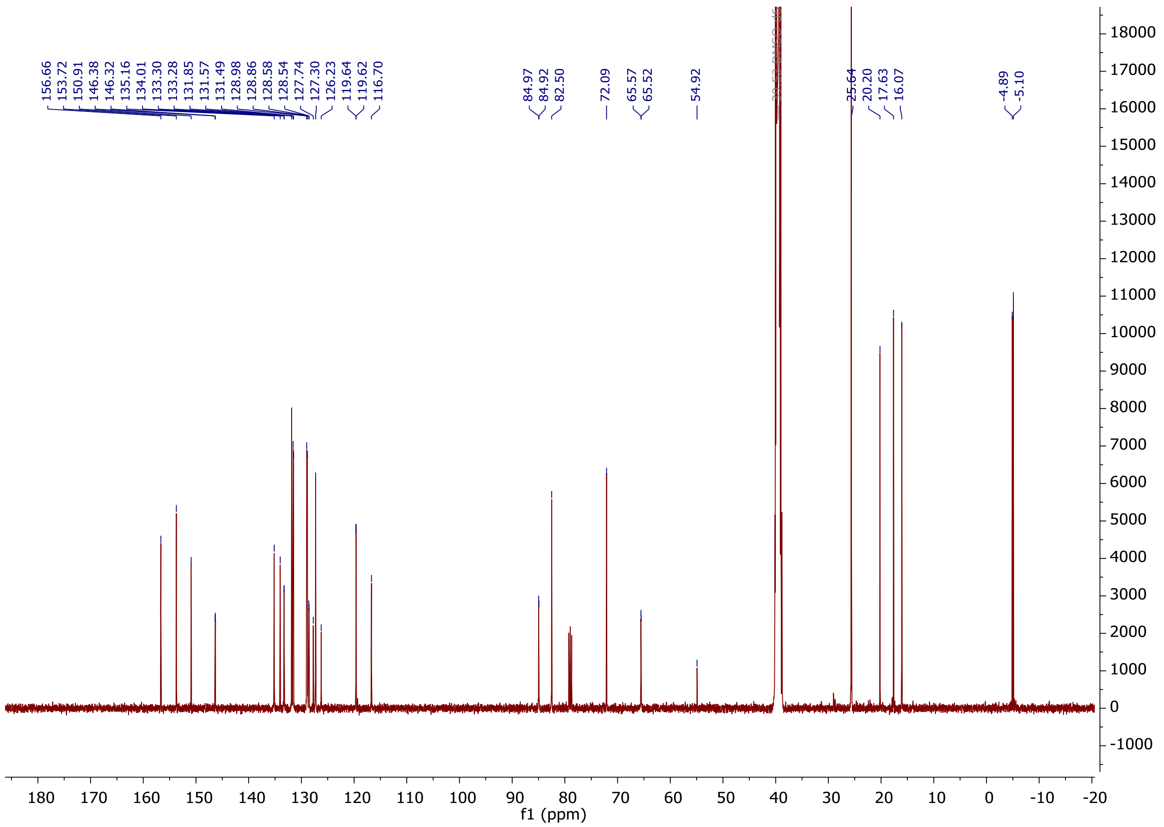

^{31}P NMR (202 MHz, DMSO-d_6):

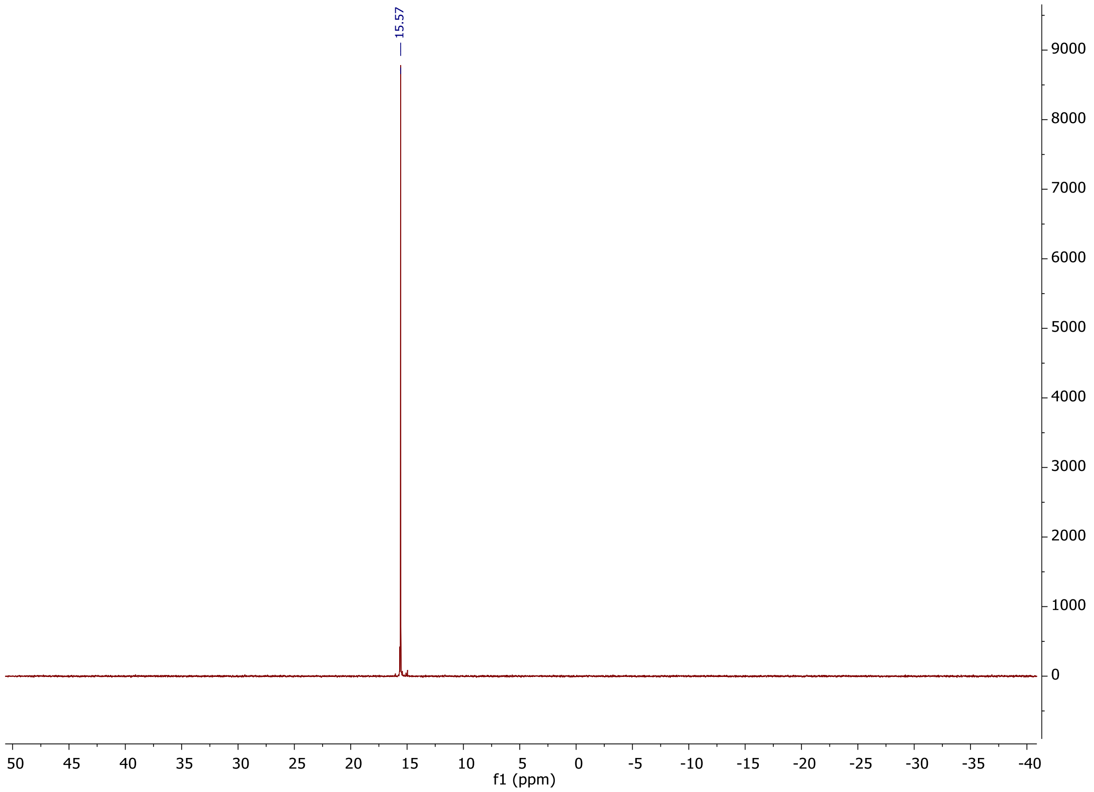

Compound **27**

^{1}H NMR (400 MHz, $CDCl_3$):

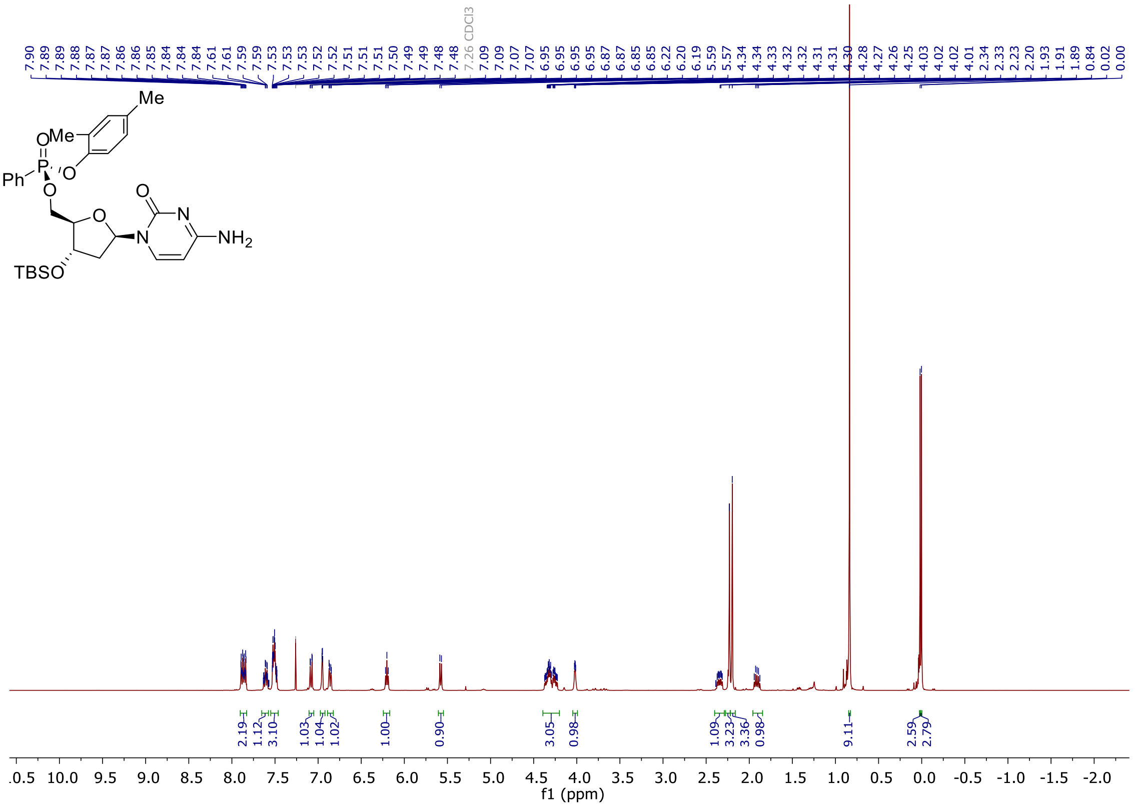

^{13}C NMR (101 MHz, $CDCl_3$):

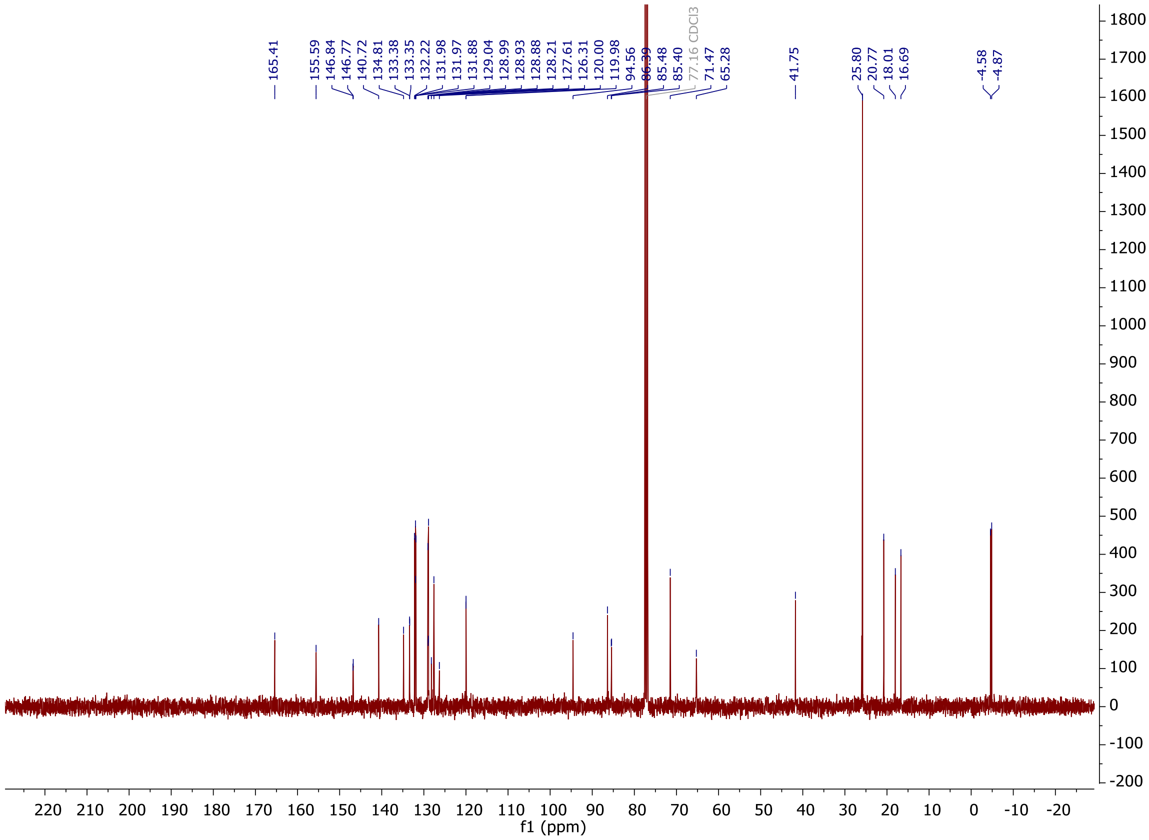

^{31}P NMR (162 MHz, $CDCl_3$):

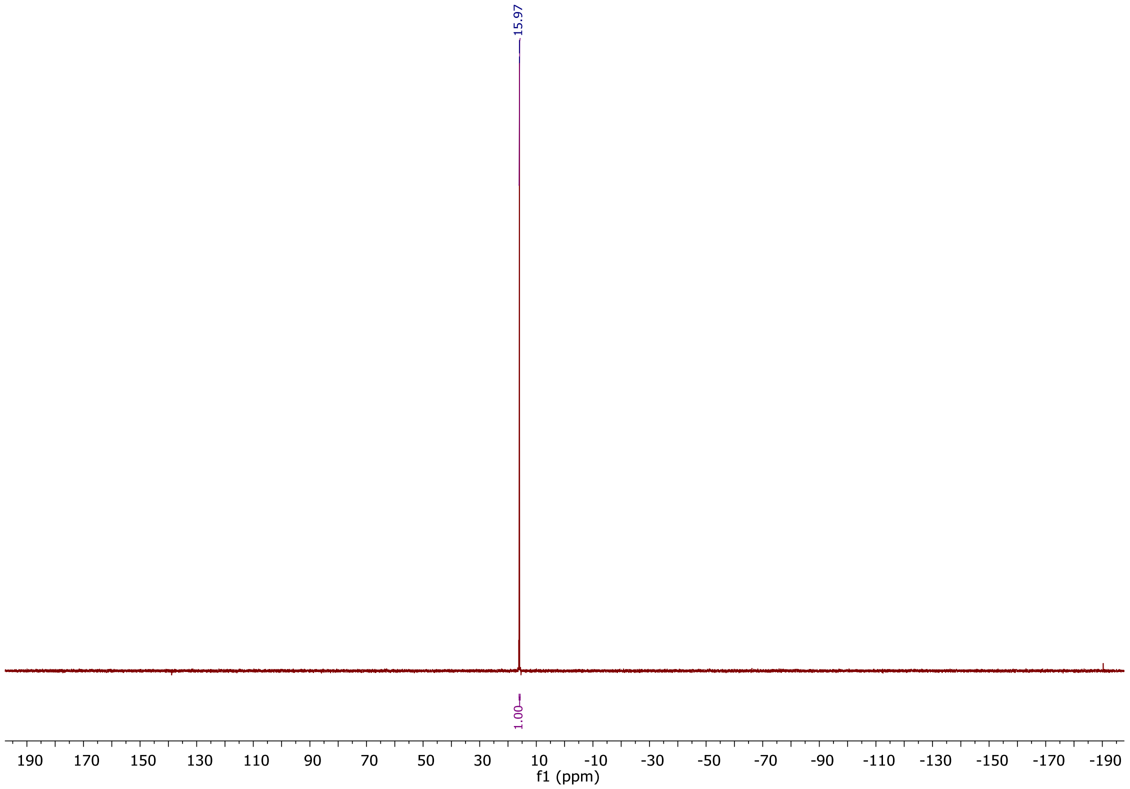

Compound **28**

^{1}H NMR (500 MHz, $CDCl_3$):

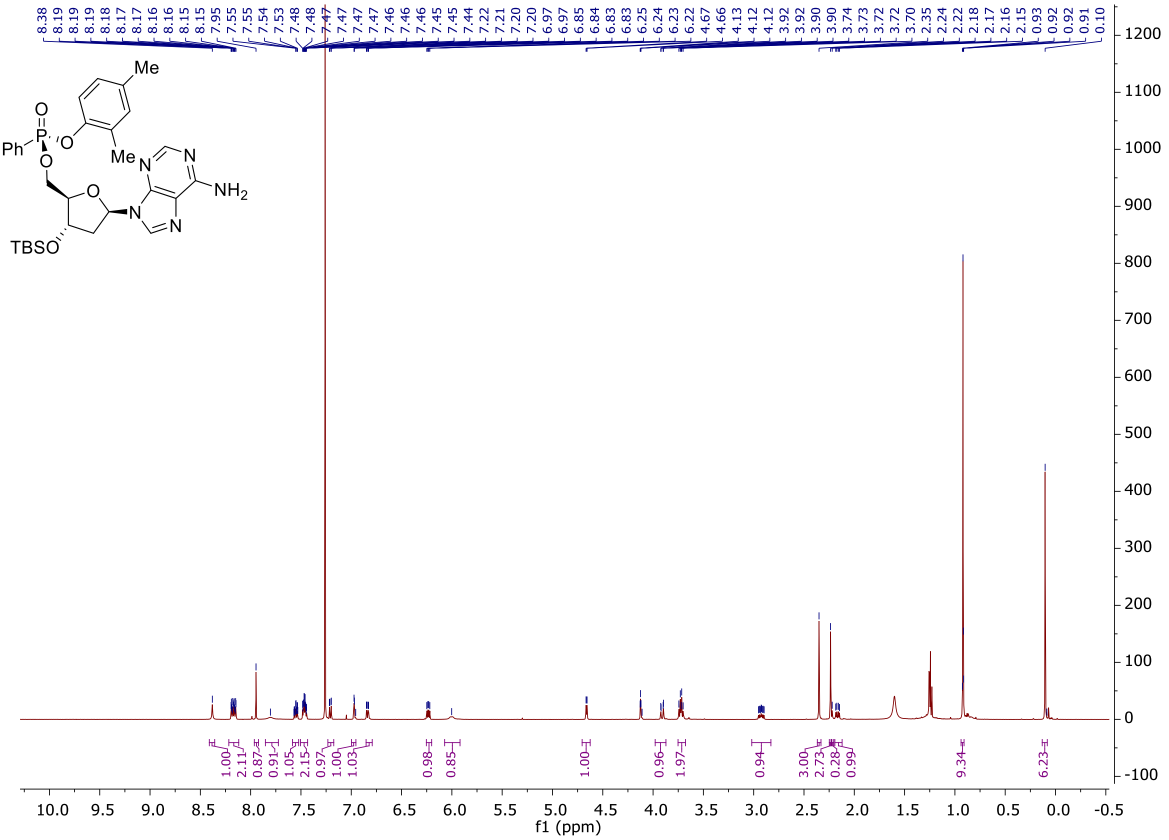

^{13}C NMR (126 MHz, $CDCl_3$):

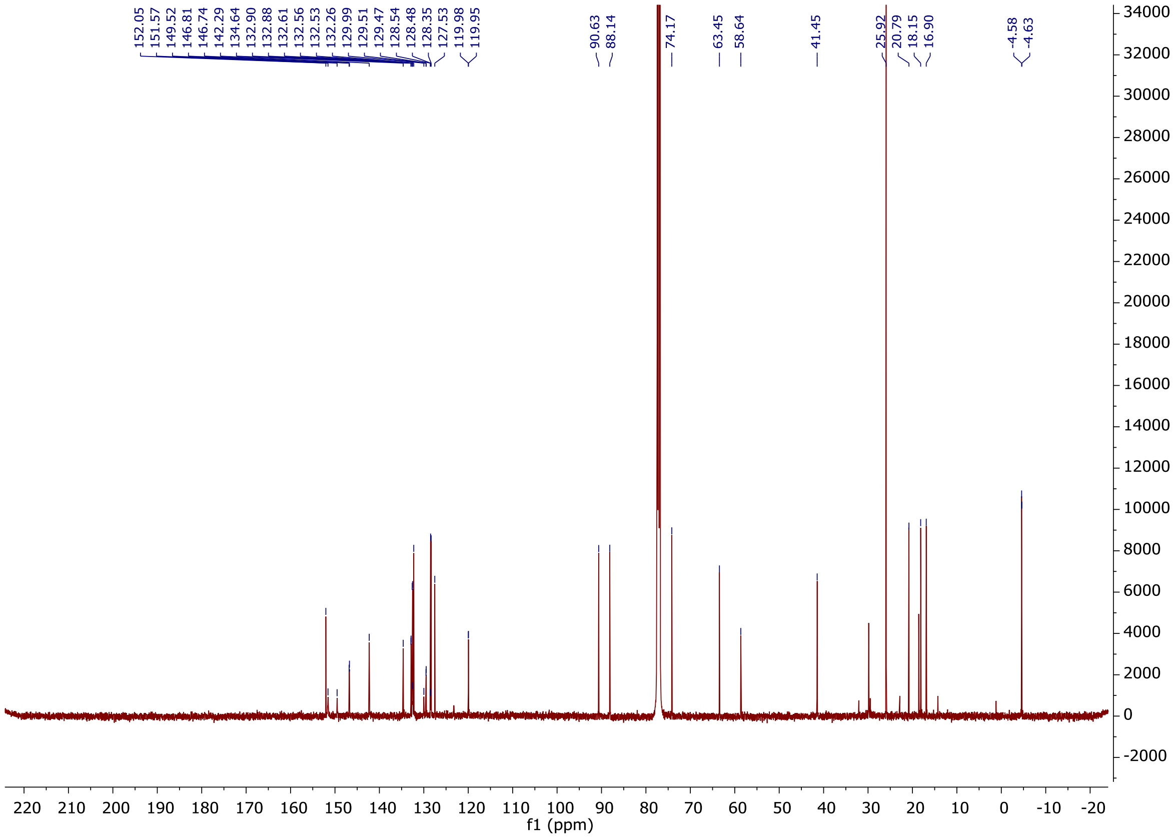

^{31}P NMR (202 MHz, $CDCl_3$):

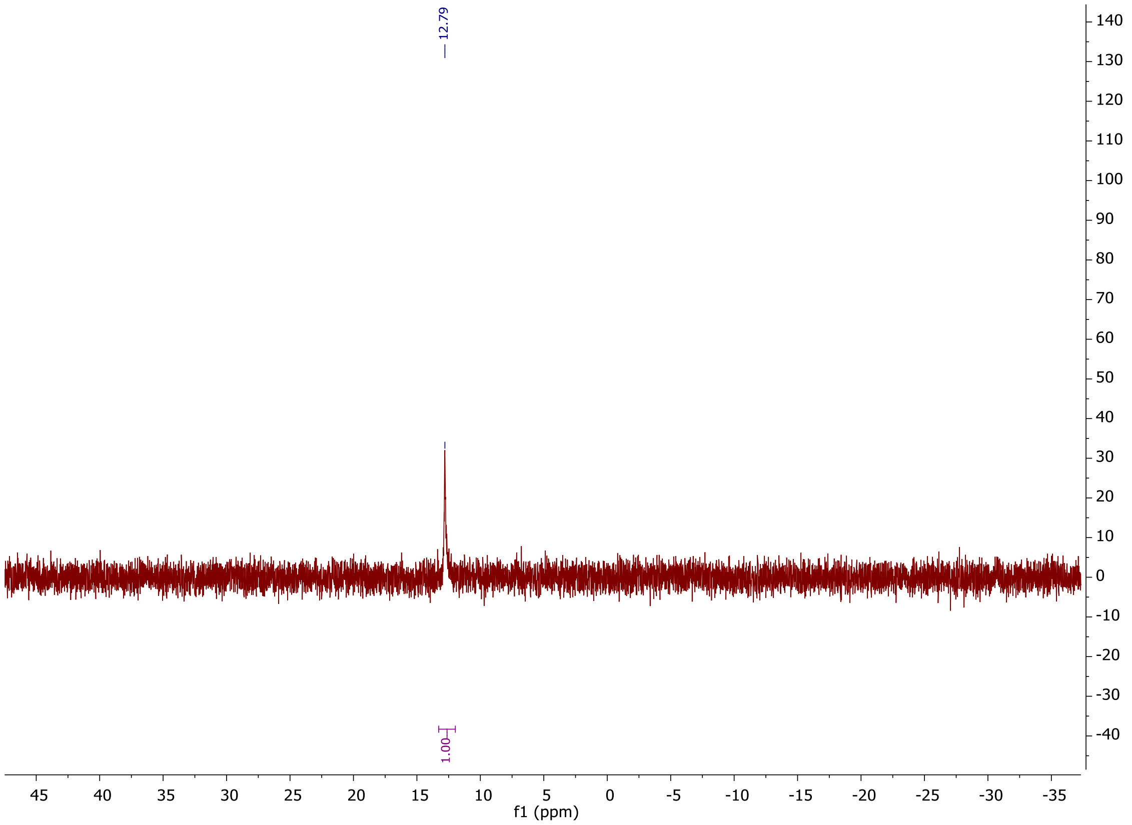

Compound **29**

^{1}H NMR (400 MHz, $CDCl_3$):

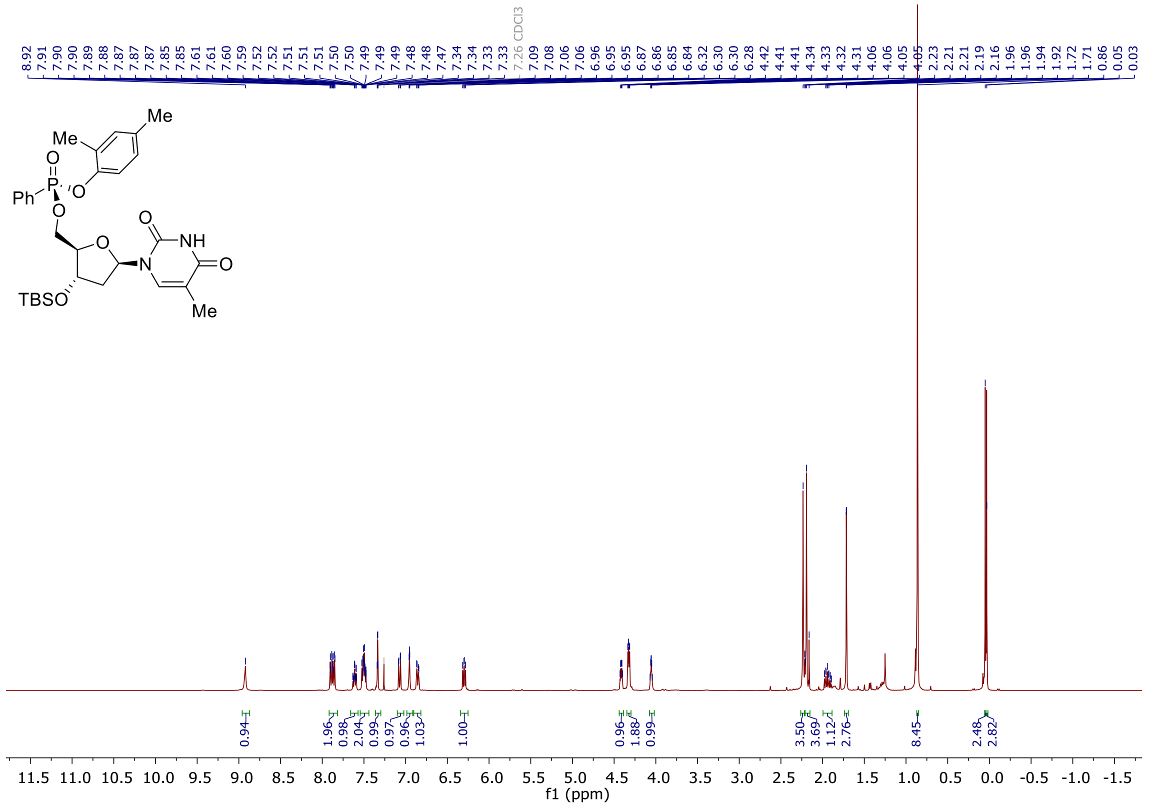

^{13}C NMR (101 MHz, $CDCl_3$):

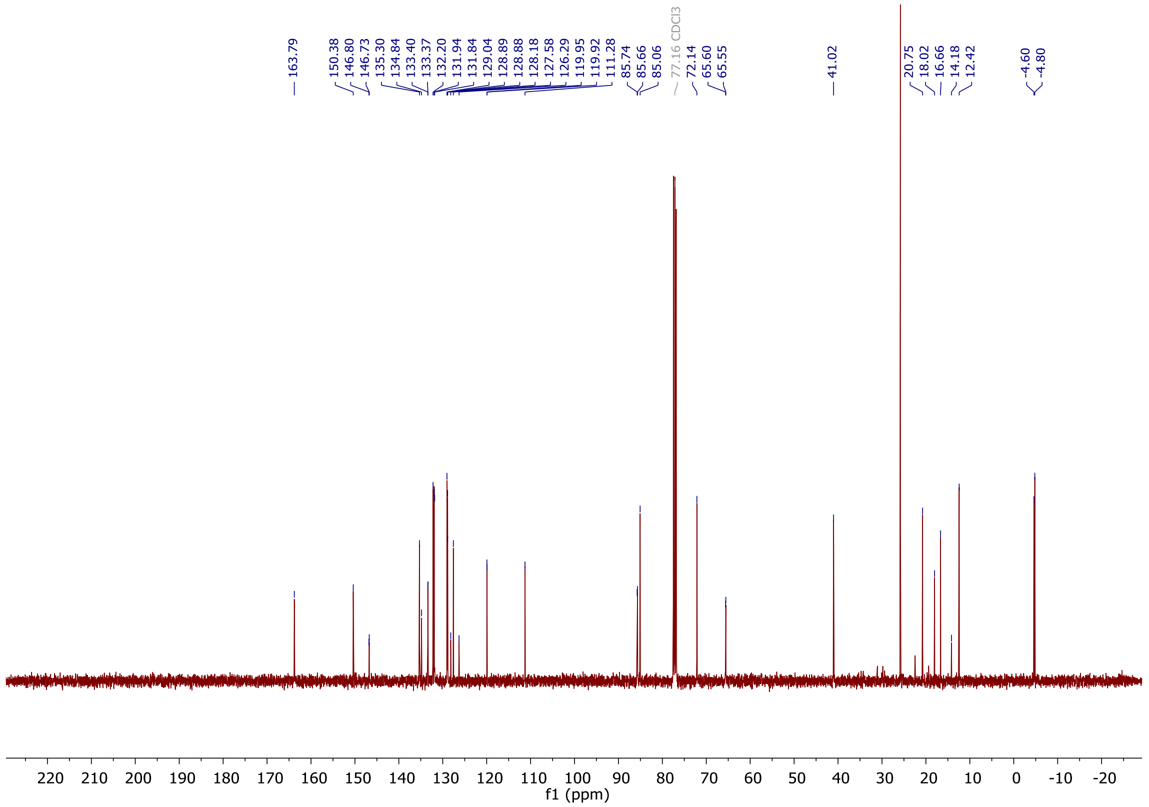

^{31}P NMR (162 MHz, $CDCl_3$):

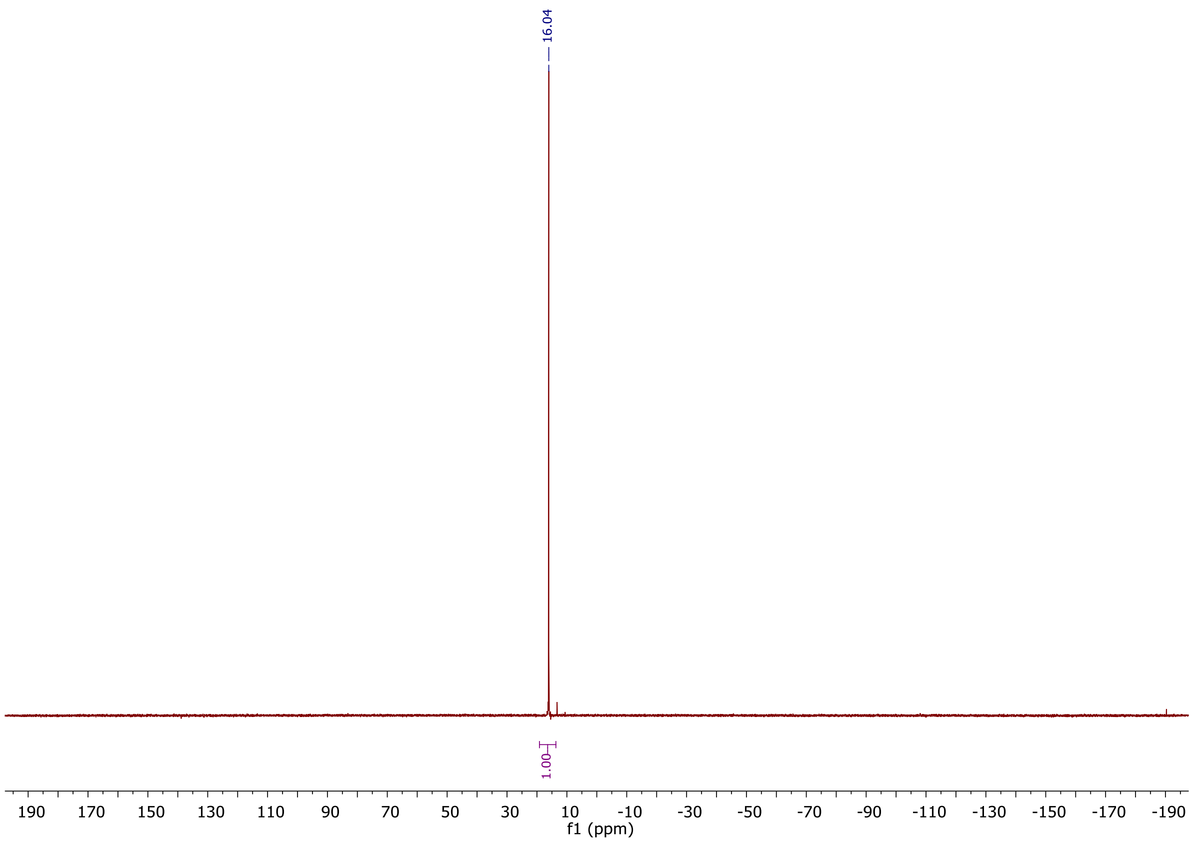

Compound **30**

^{1}H NMR (400 MHz, $CDCl_3$)

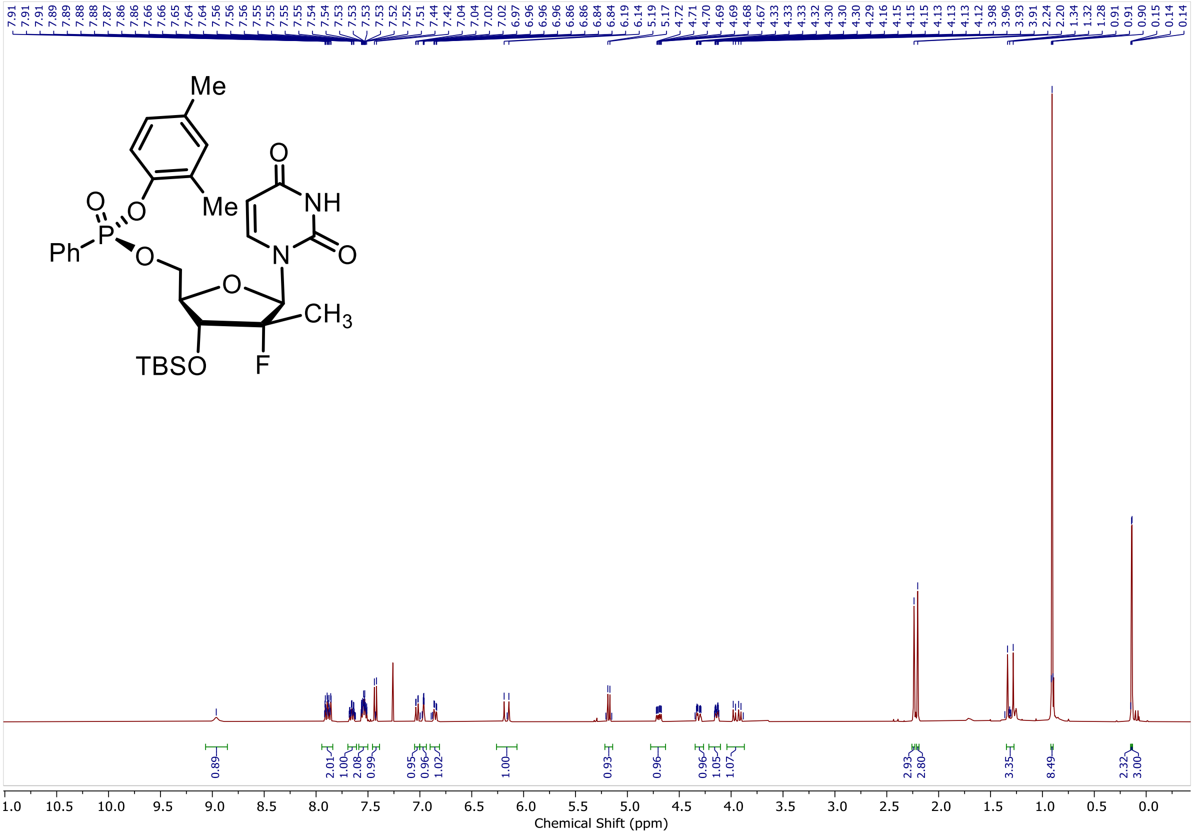

^{13}C NMR (151 MHz, $CDCl_3$)

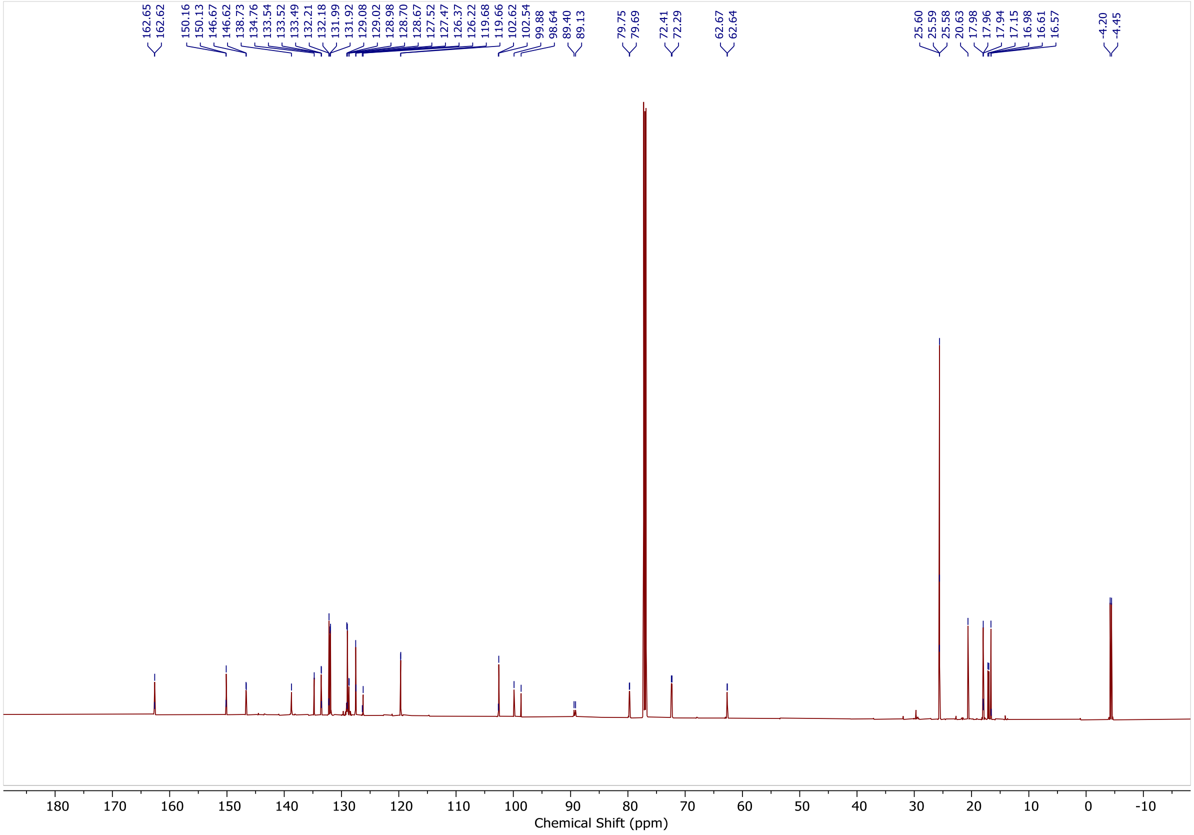

^{31}P NMR (162 MHz, $CDCl_3$)

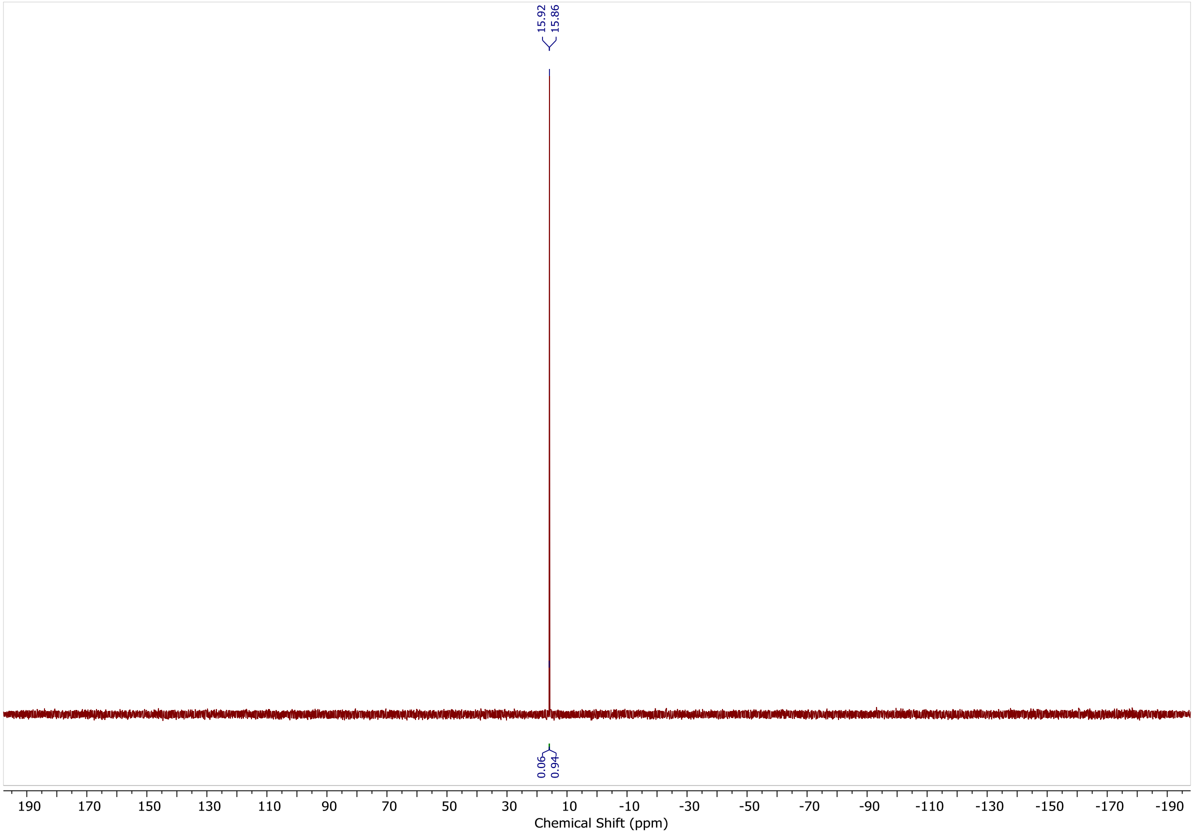

^{19}F NMR (377 MHz, $CDCl_3$)

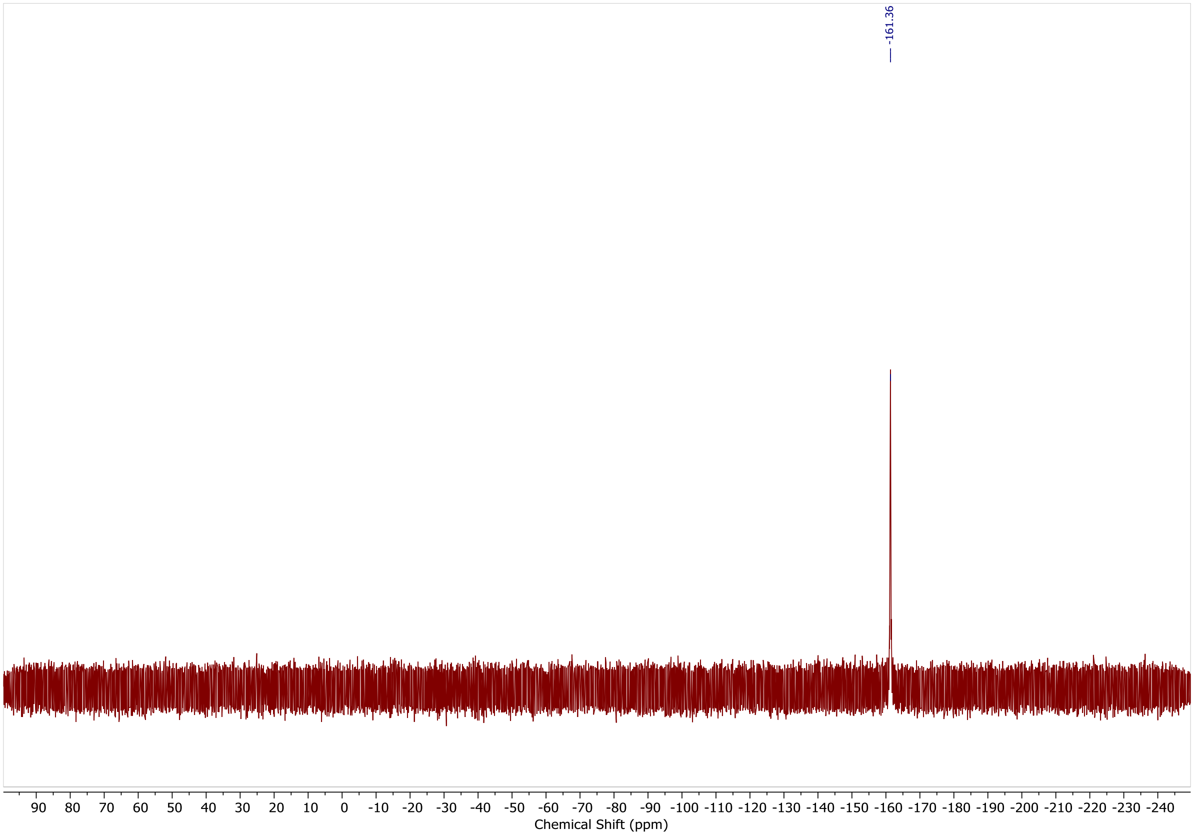

Compound **31**

^{1}H NMR (400 MHz, CDCl$_3$) (using *rac*-1 as SM):

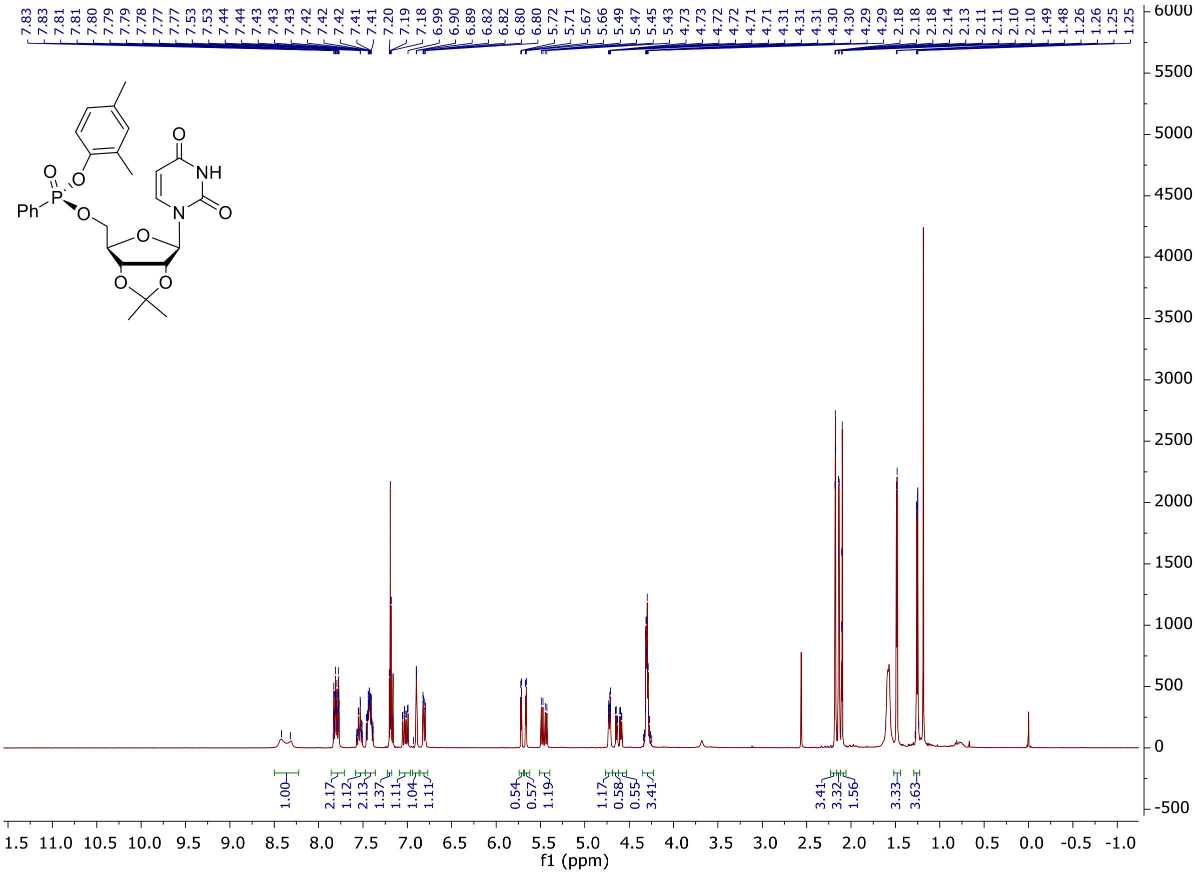

^{1}H NMR (400 MHz, CDCl$_3$) (enantioenriched):

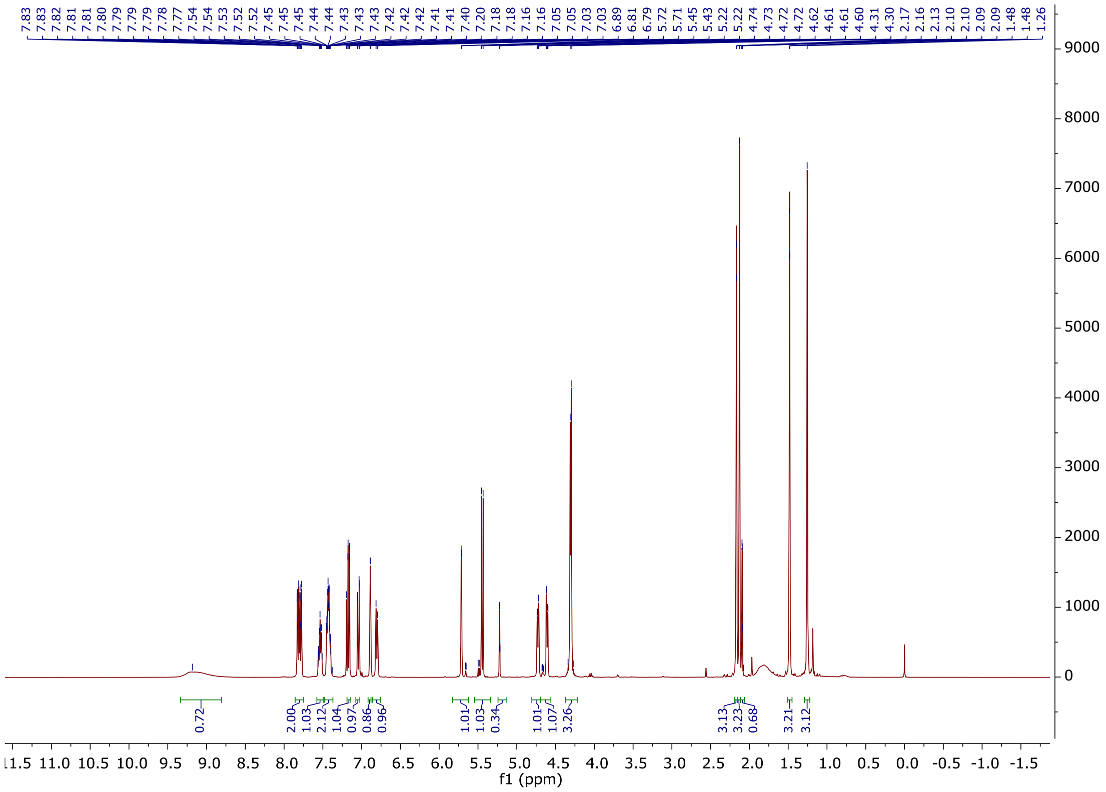

^{13}C NMR (101 MHz, $CDCl_3$) (enantioenriched):

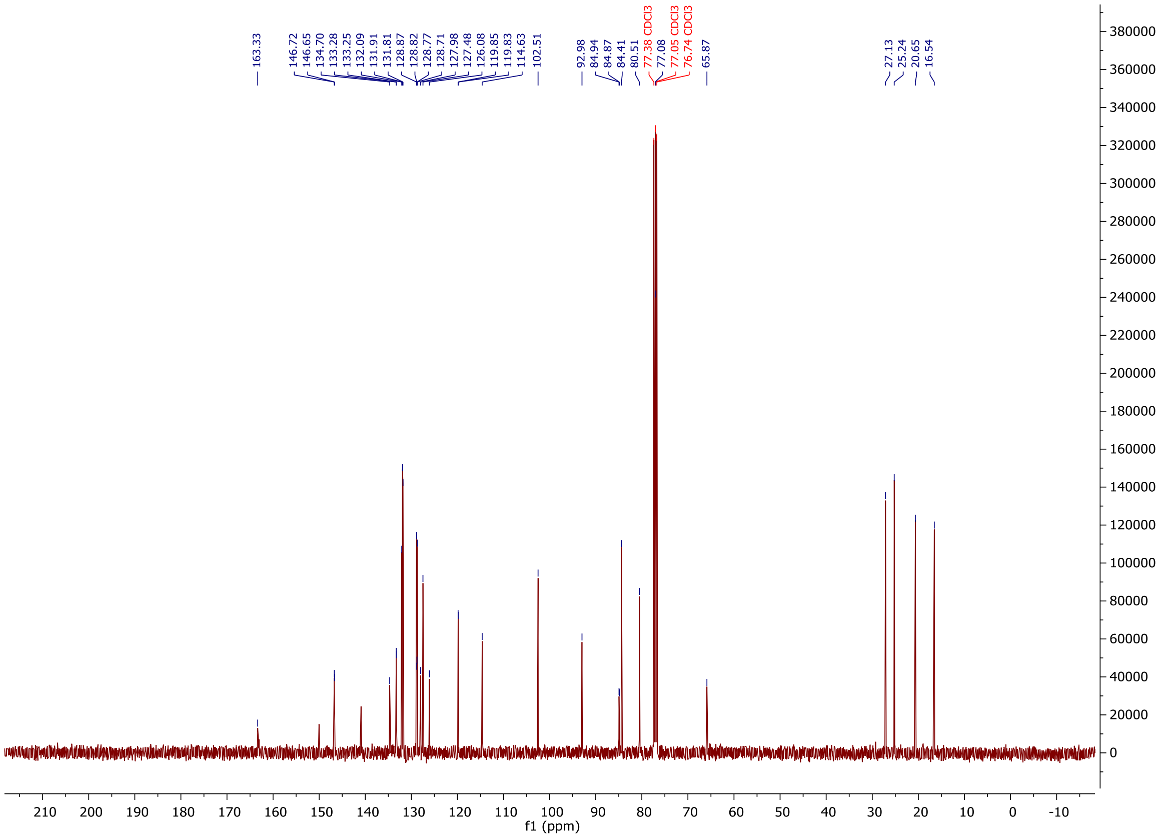

^{31}P NMR (162 MHz, $CDCl_3$) (using *rac*-1 as SM):

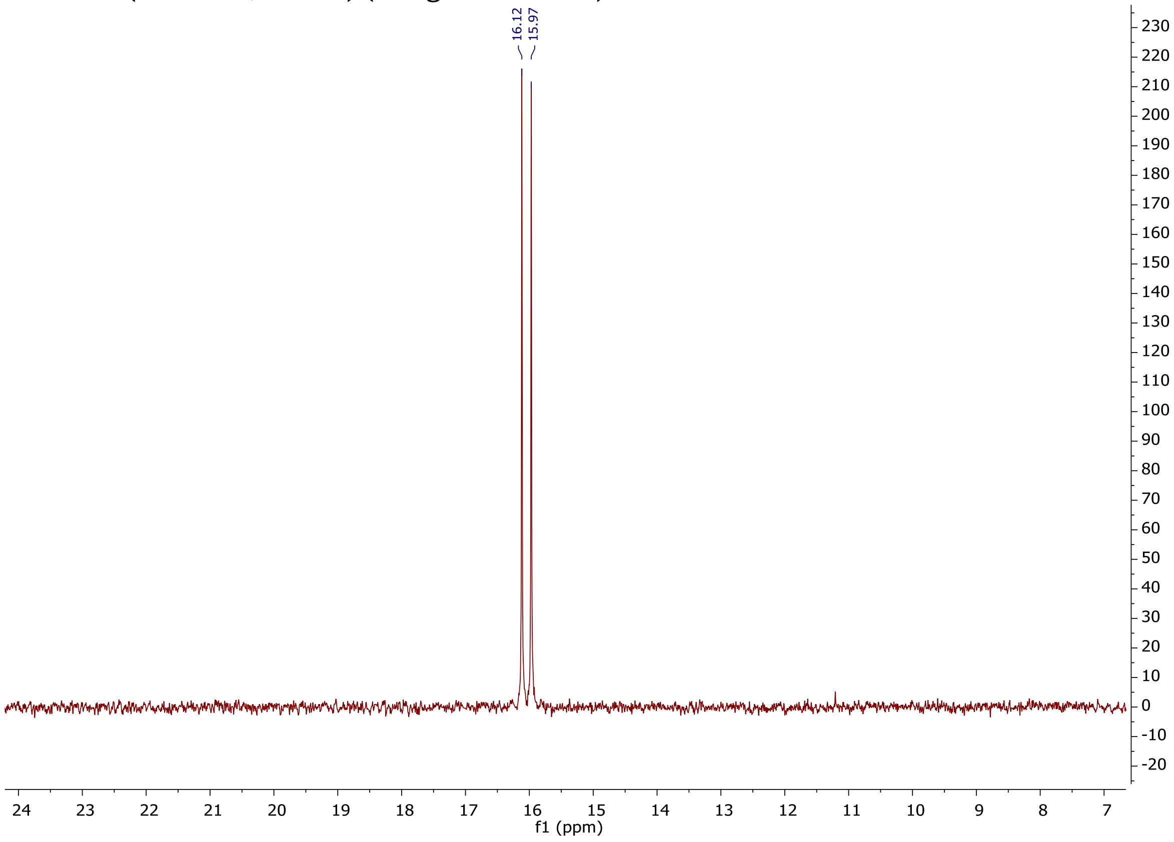

^{31}P NMR (162 MHz, CDCl$_3$) (enantioenriched):

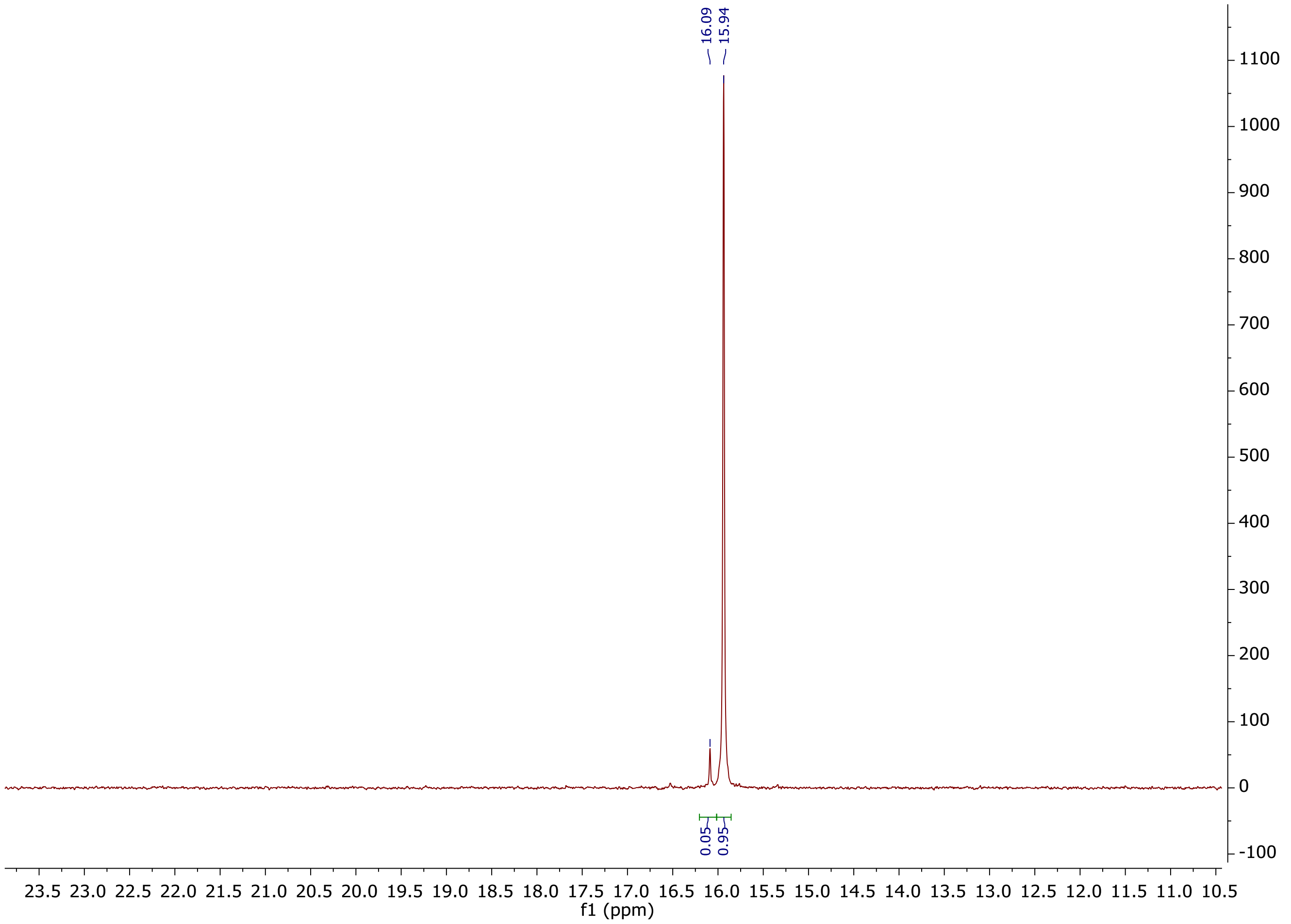

Compound **32**

^{1}H NMR (500 MHz, $CDCl_3$):

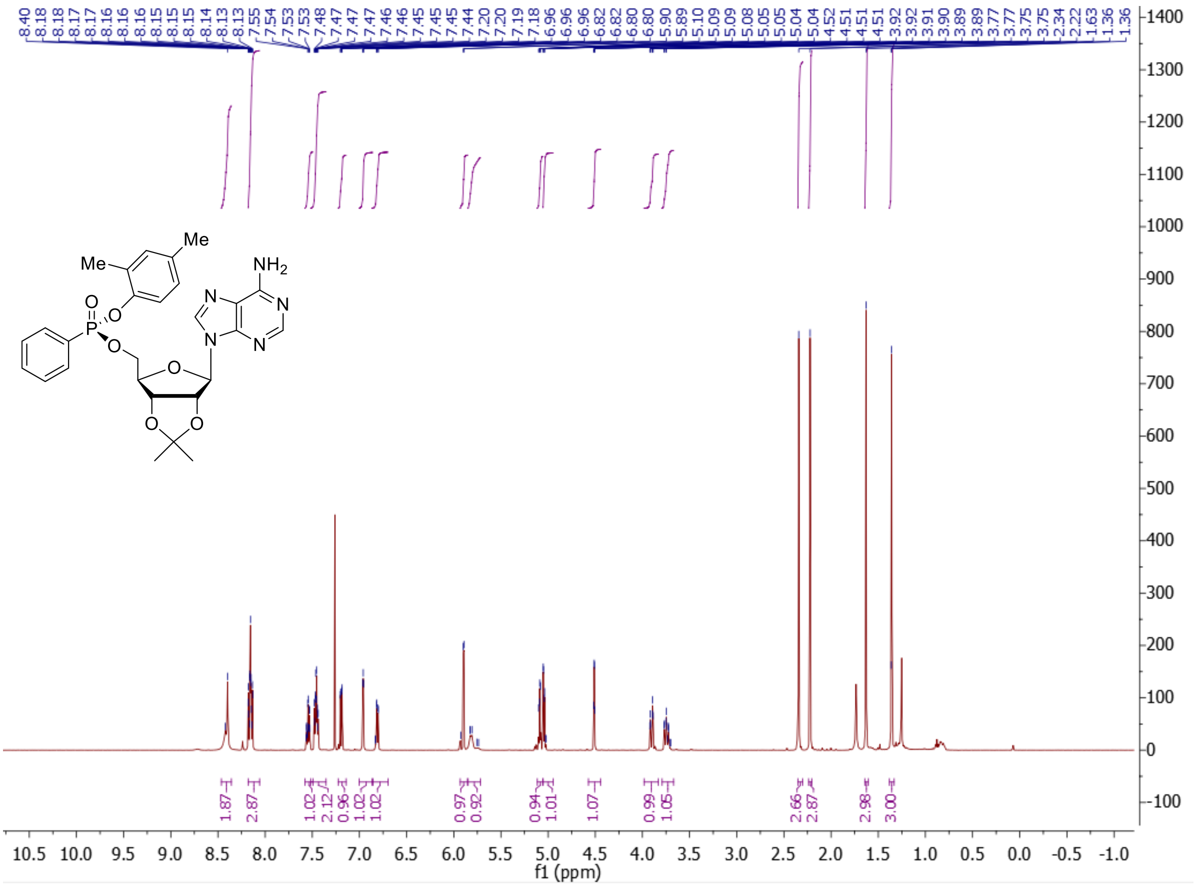

^{13}C NMR (101 MHz, $CDCl_3$) :

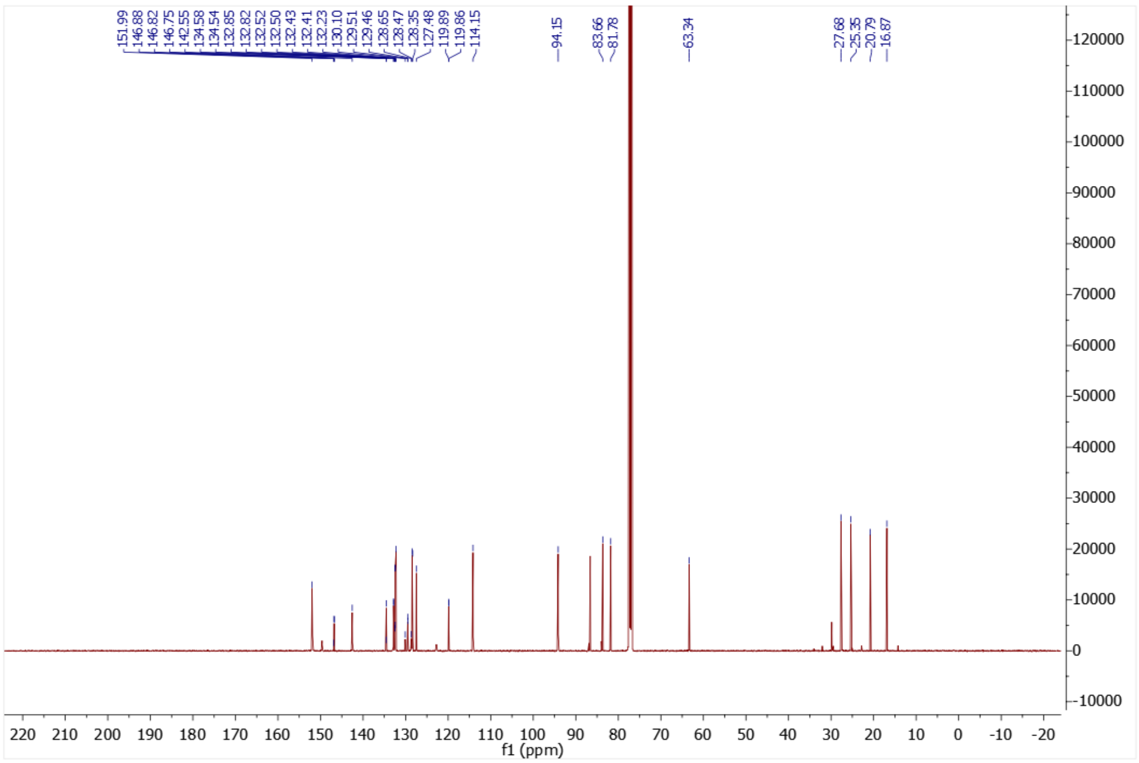

^{31}P NMR (162 MHz, CDCl$_3$):

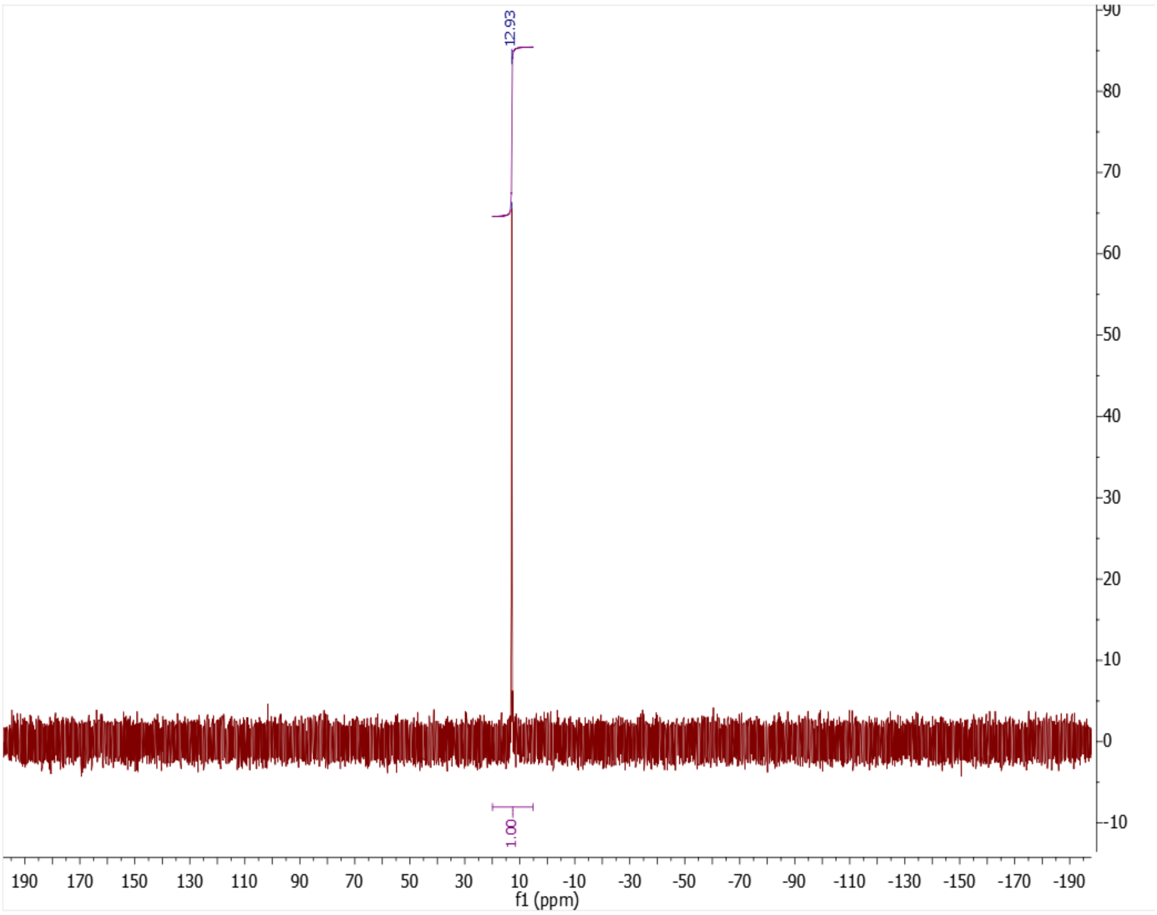

Compound **33**

^{1}H NMR (400 MHz, $CDCl_3$) (using *rac*-1 as SM):

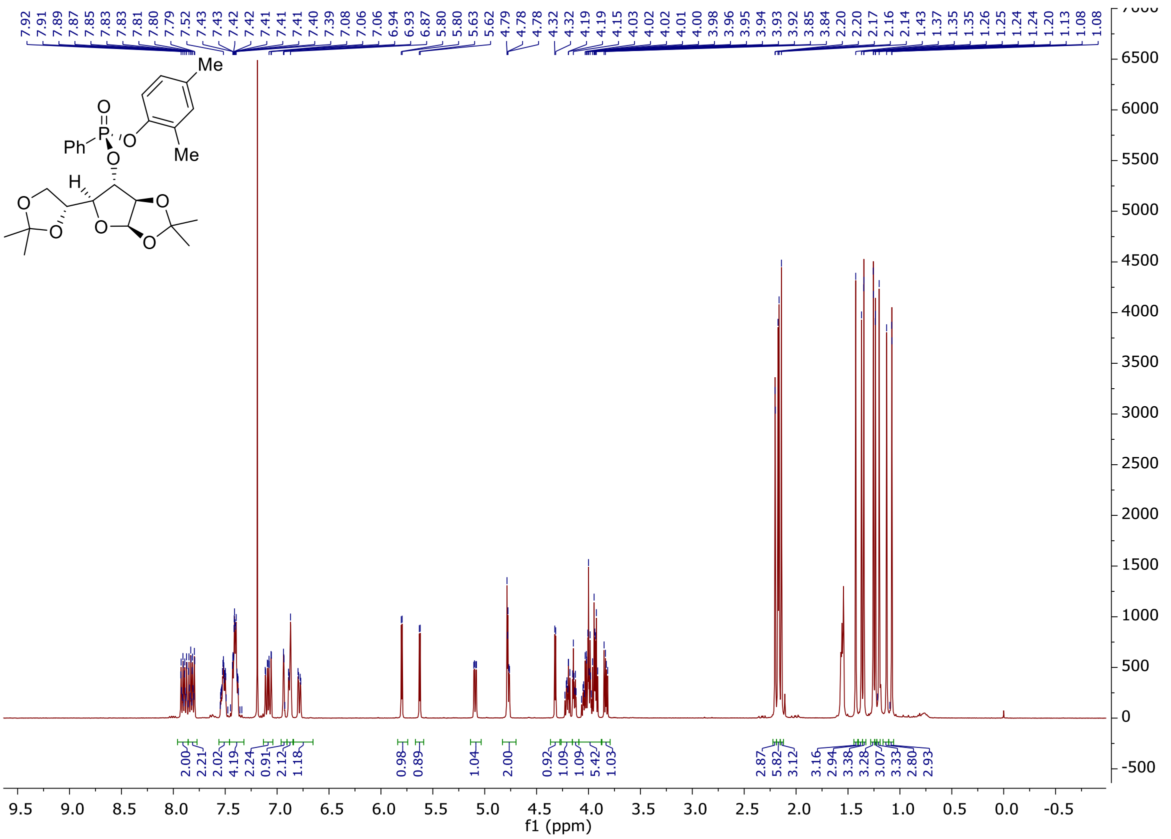

^{1}H NMR (400 MHz, $CDCl_3$) (enantioenriched):

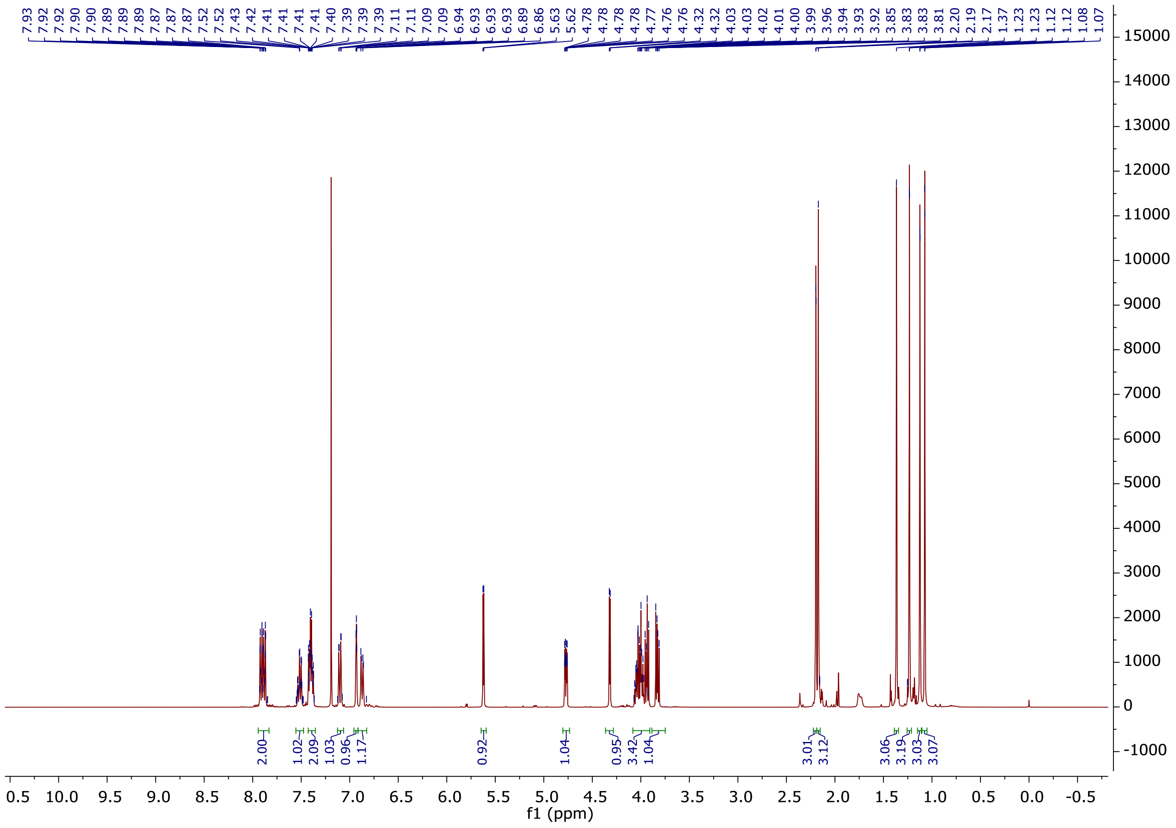

^{13}C NMR (101 MHz, $CDCl_3$) (enantioenriched):

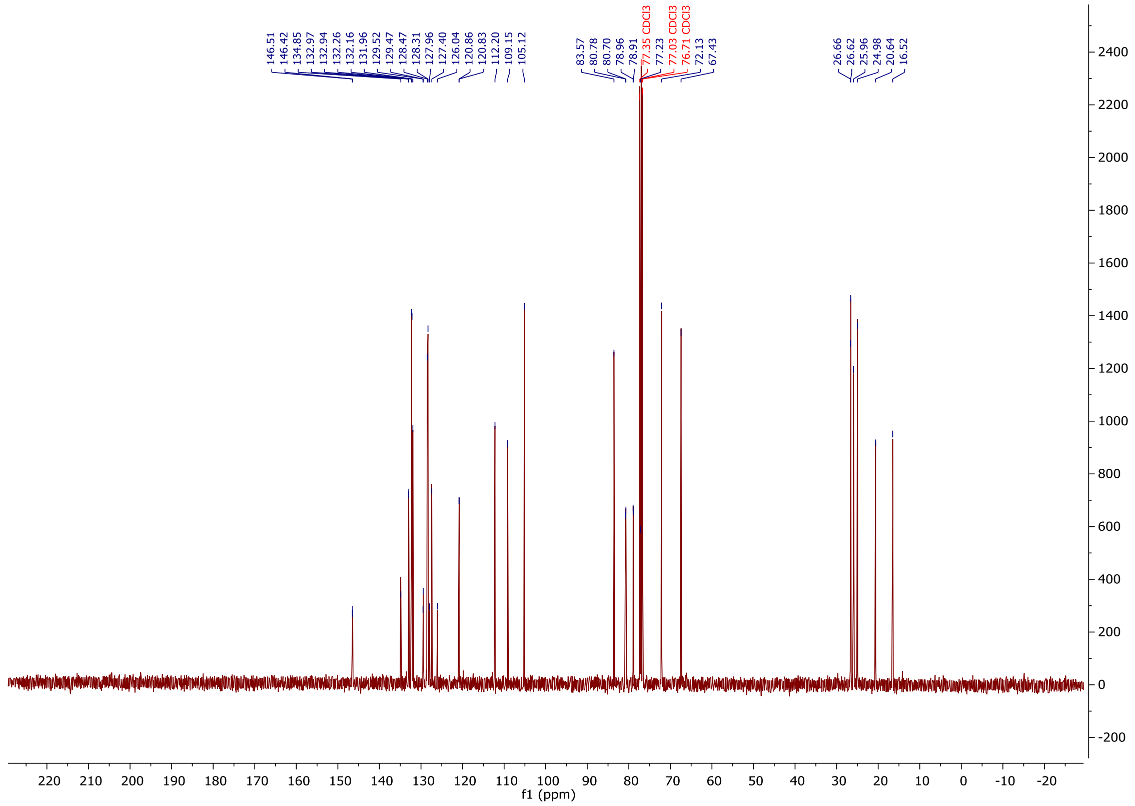

^{31}P NMR (162 MHz, $CDCl_3$) (using *rac*-1 as SM):

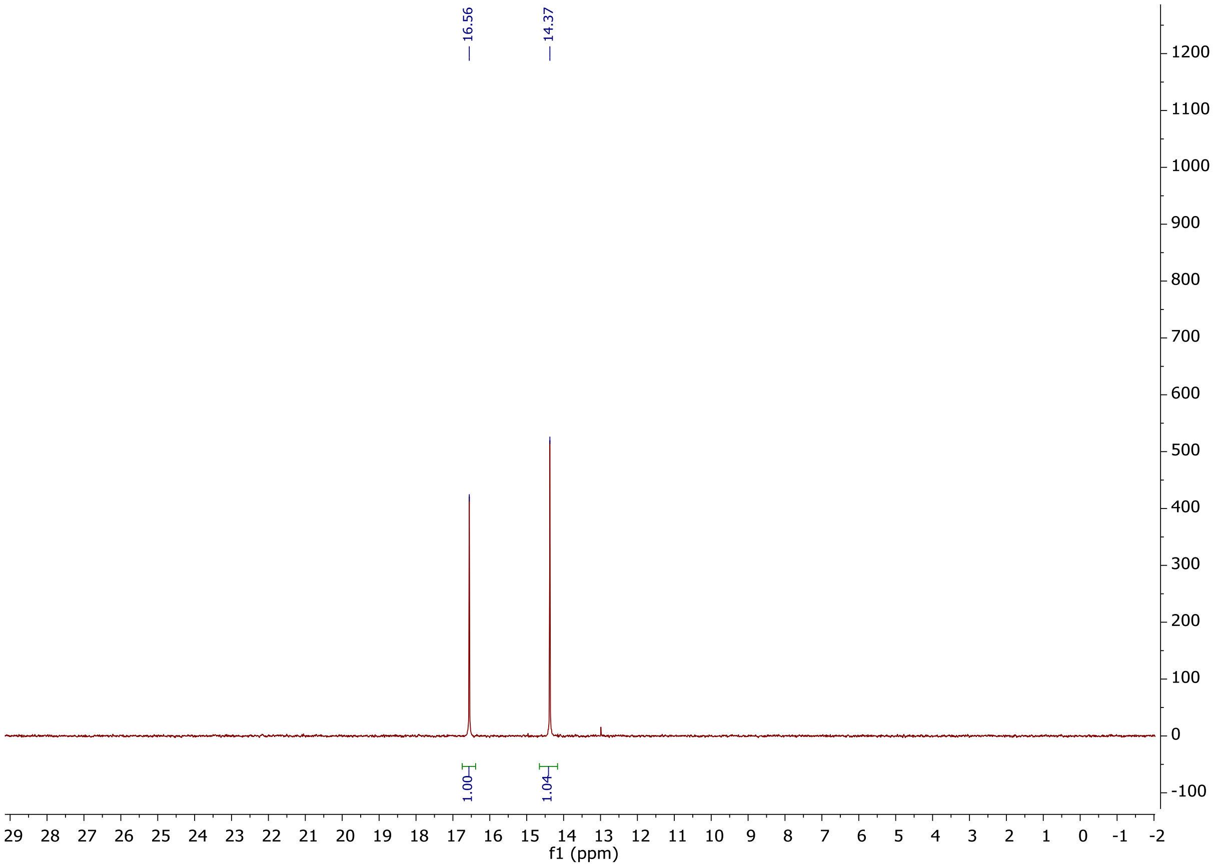

^{31}P NMR (162 MHz, $CDCl_3$) (enantioenriched):

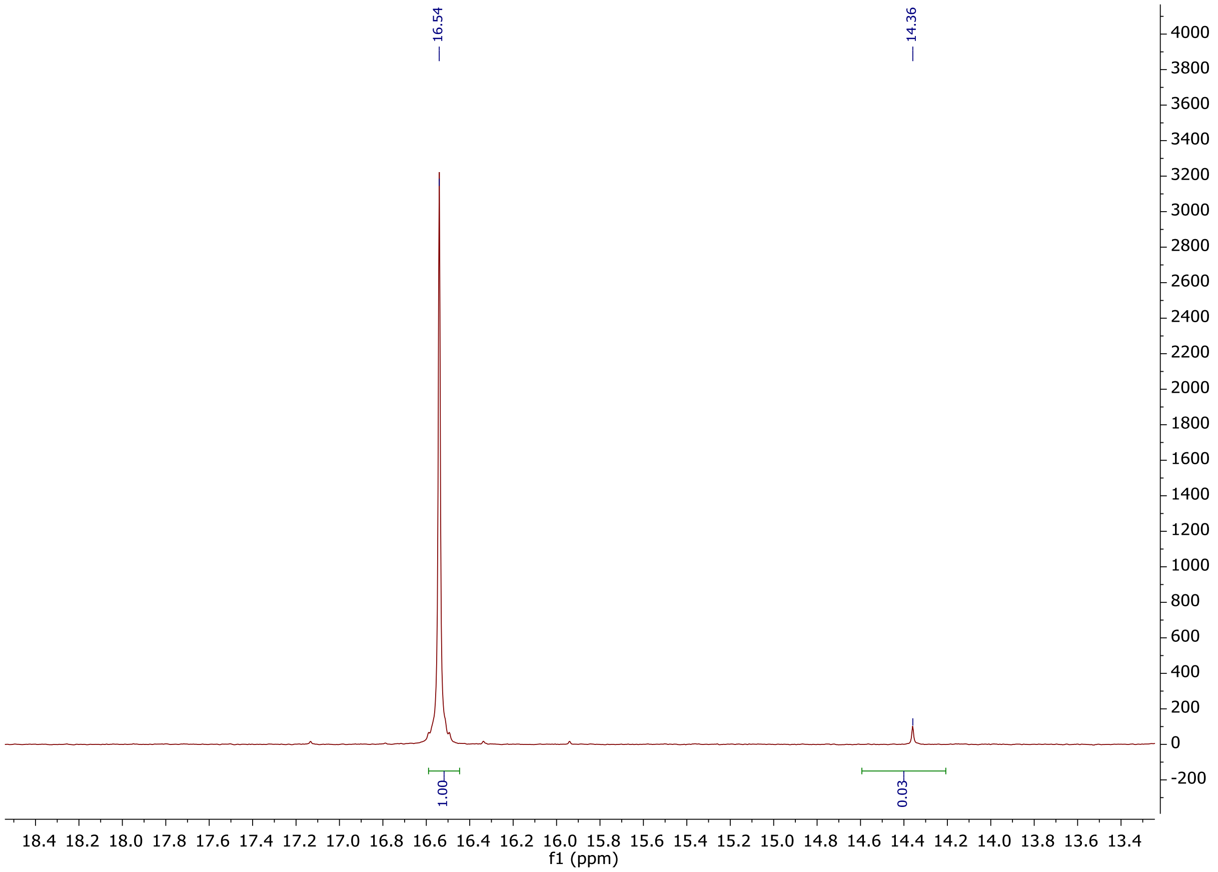

Compound **34**

^{1}H NMR (400 MHz, CDCl$_3$):

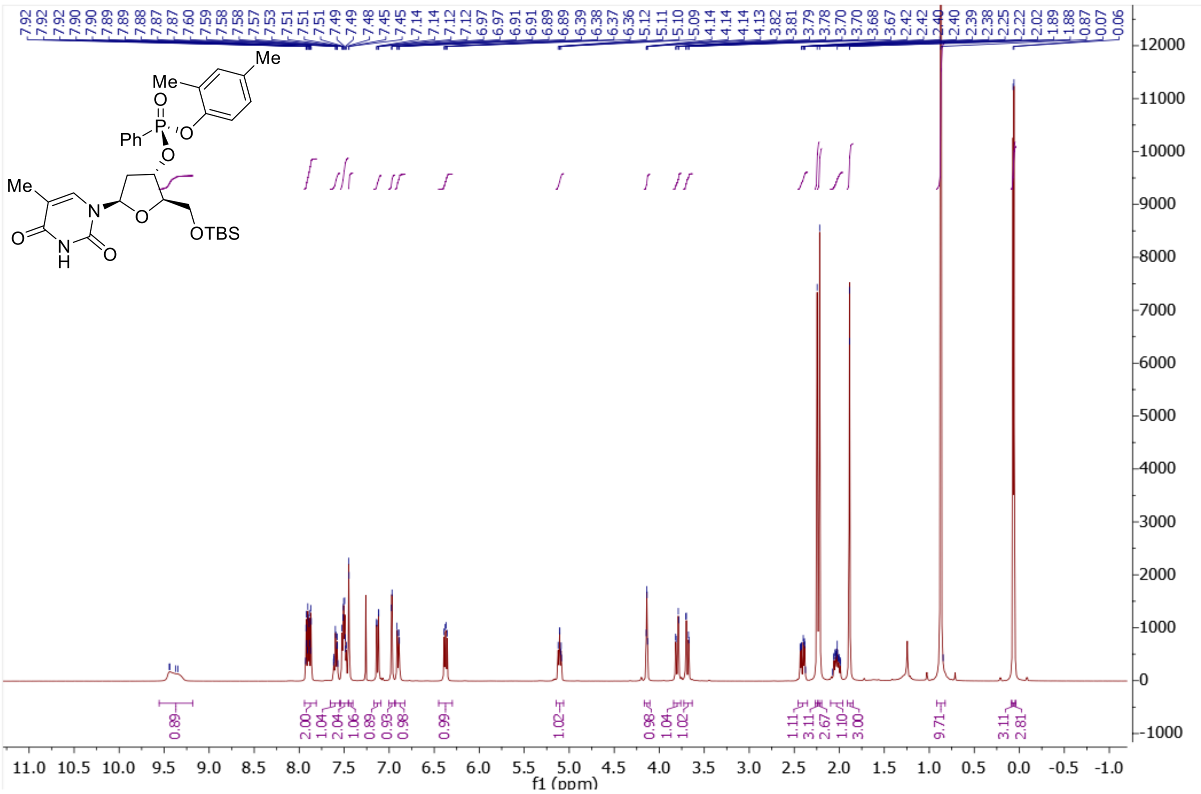

^{13}C NMR (101 MHz, CDCl$_3$):

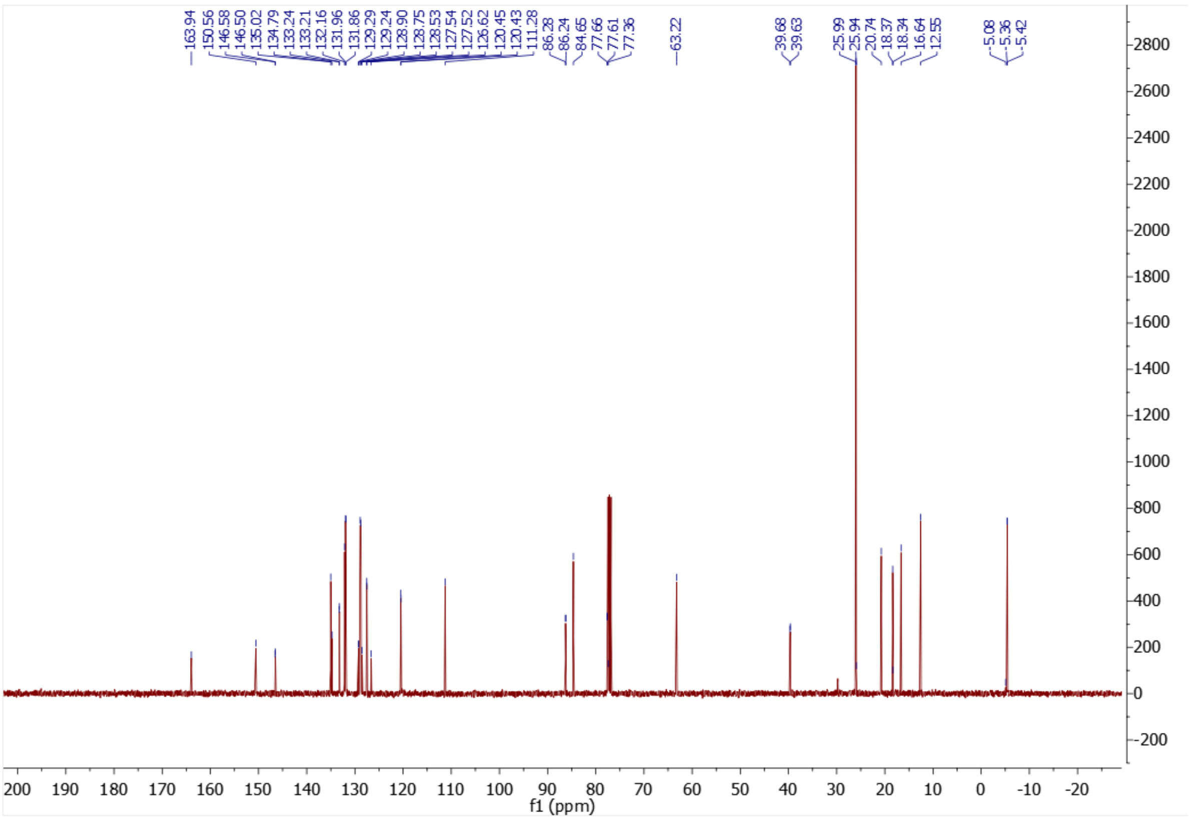

^{31}P NMR (162 MHz, $CDCl_3$):

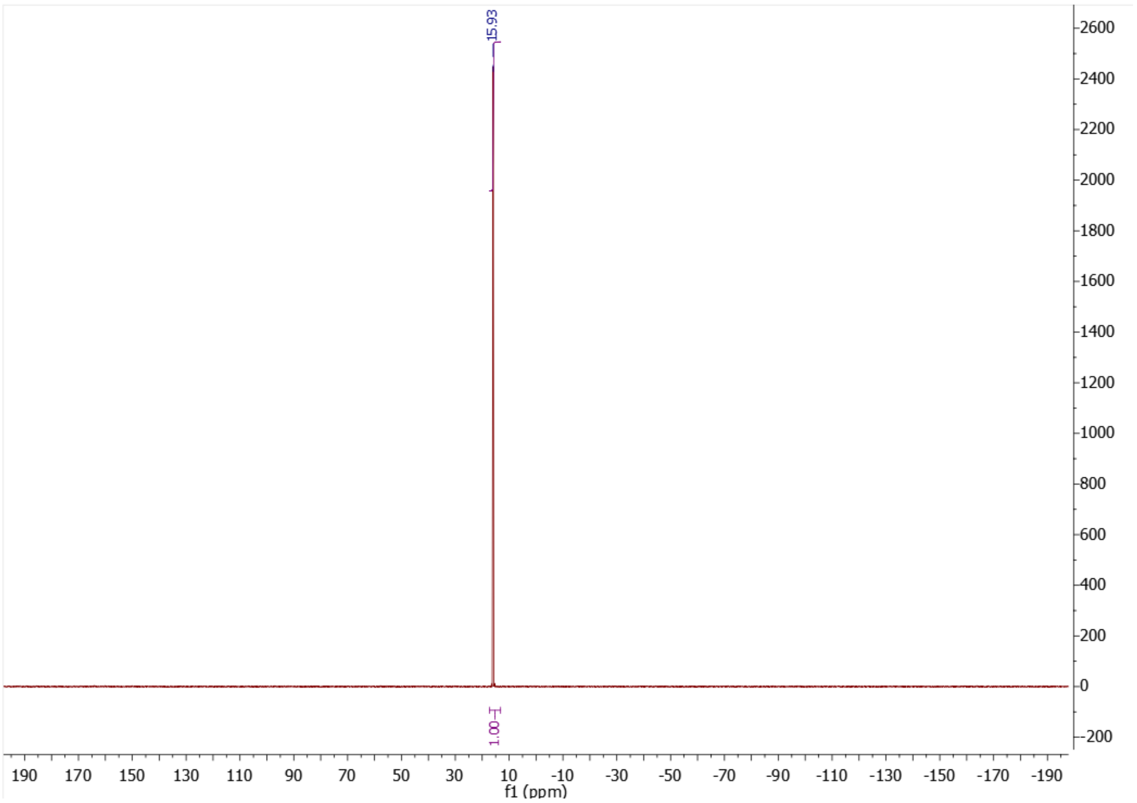

Compound **35**

^{1}H NMR (400 MHz, $CDCl_3$):

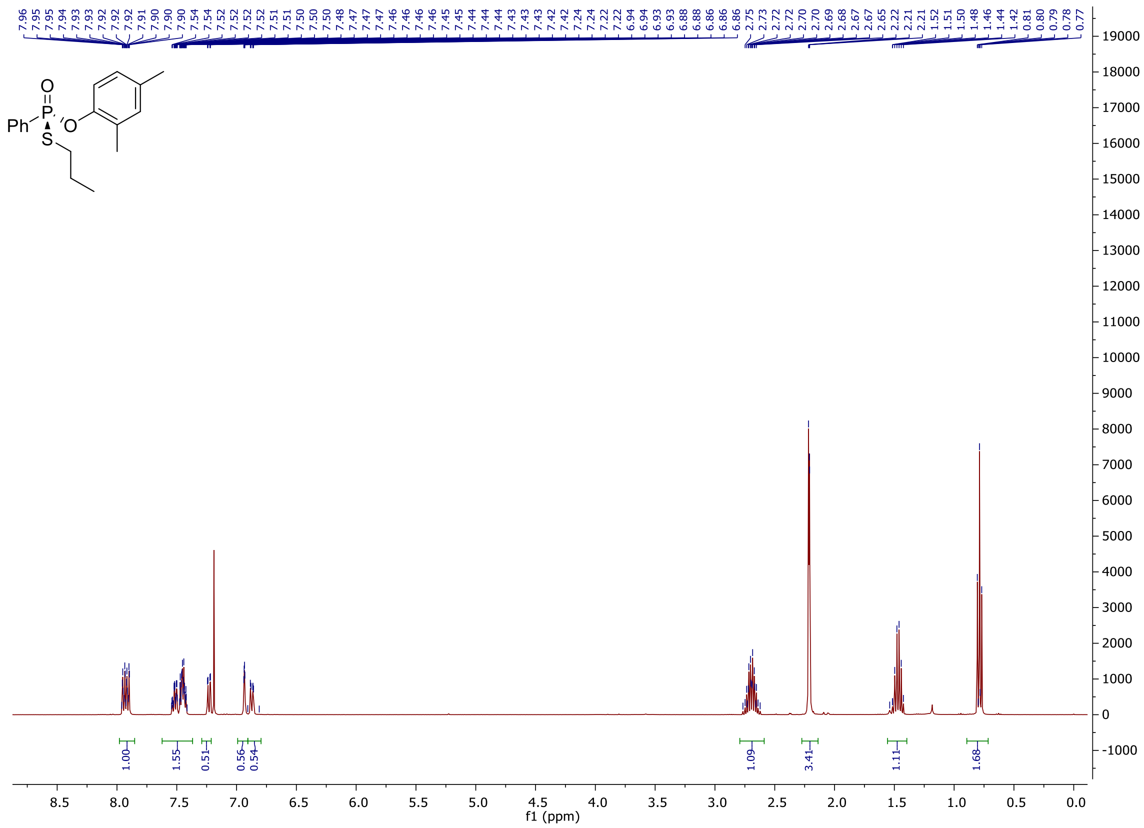

^{13}C NMR (101 MHz, $CDCl_3$):

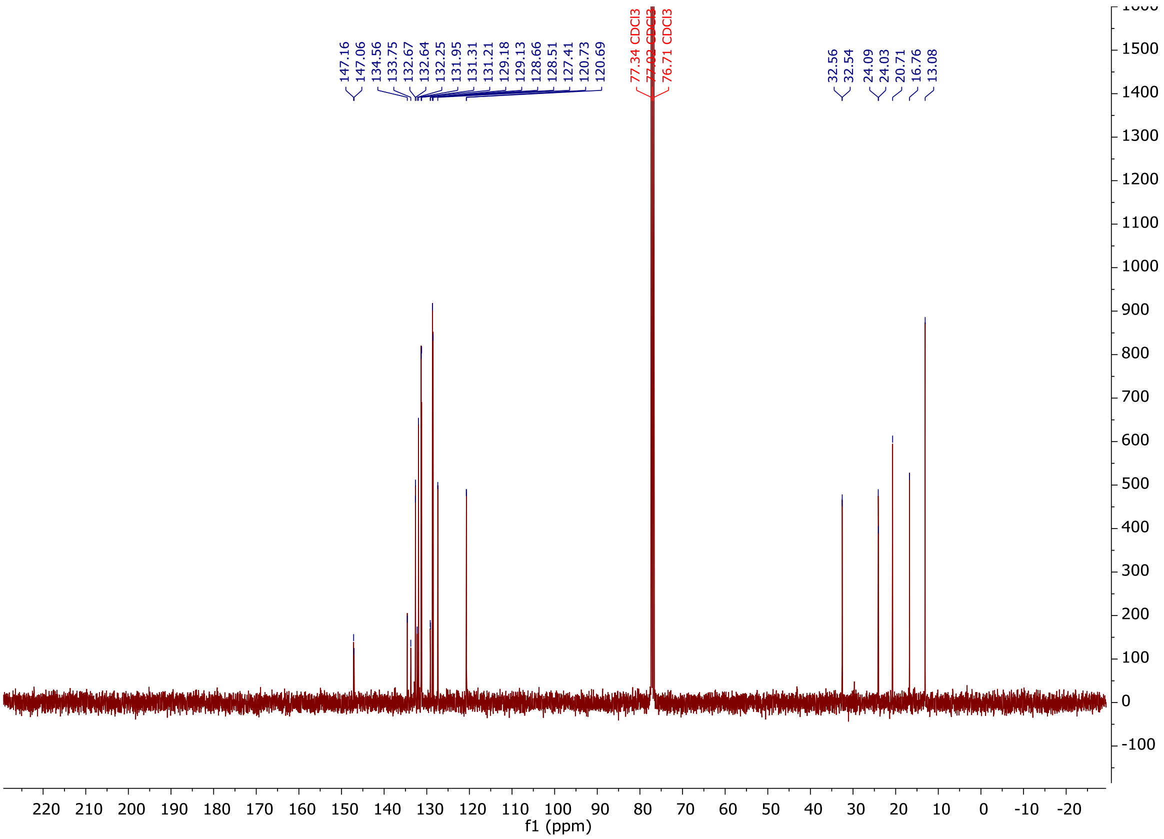

^{31}P NMR (162 MHz, $CDCl_3$):

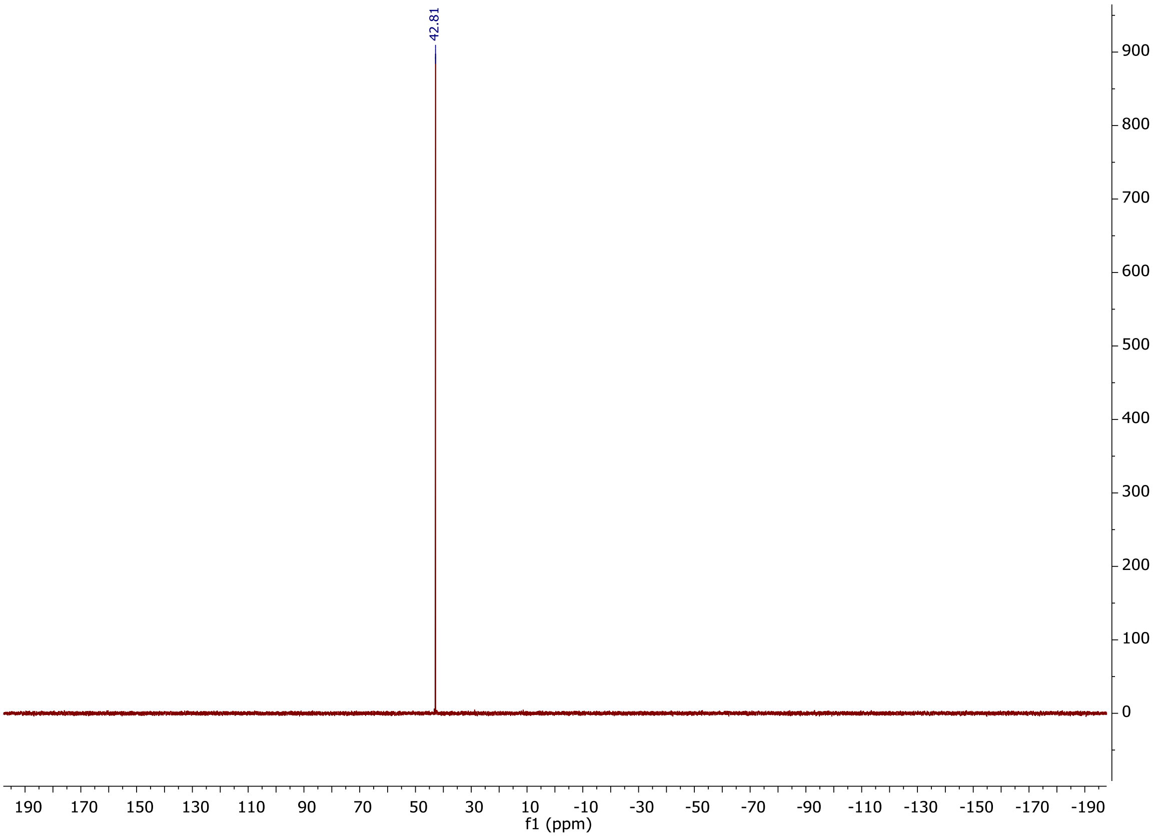

Compound **36**

^{1}H NMR (400 MHz, $CDCl_3$):

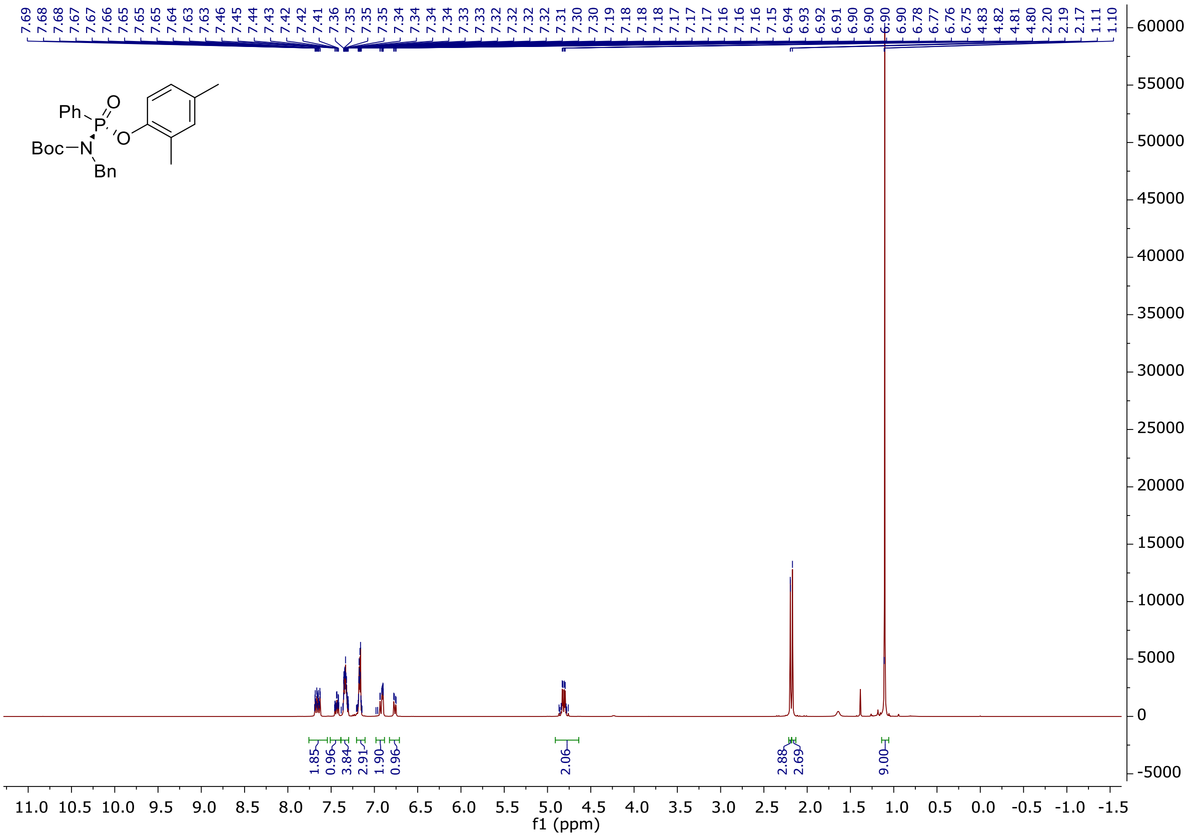

^{13}C NMR (101 MHz, $CDCl_3$):

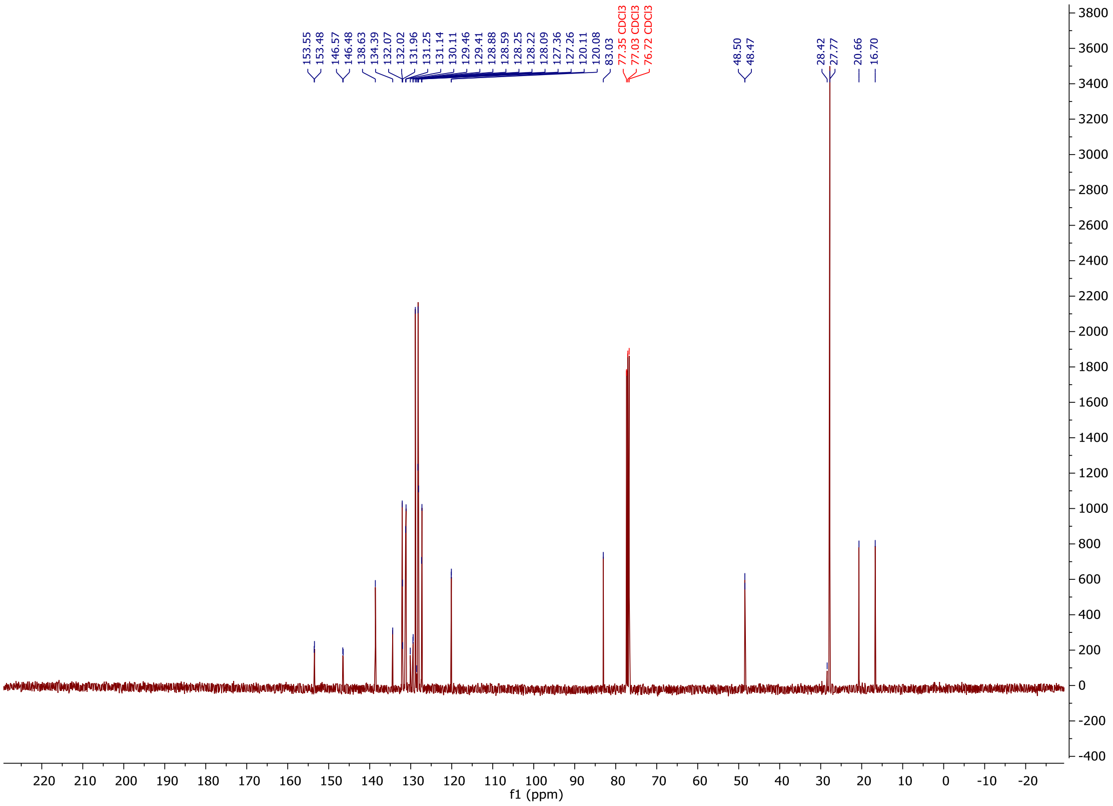

^{31}P NMR (162 MHz, $CDCl_3$):

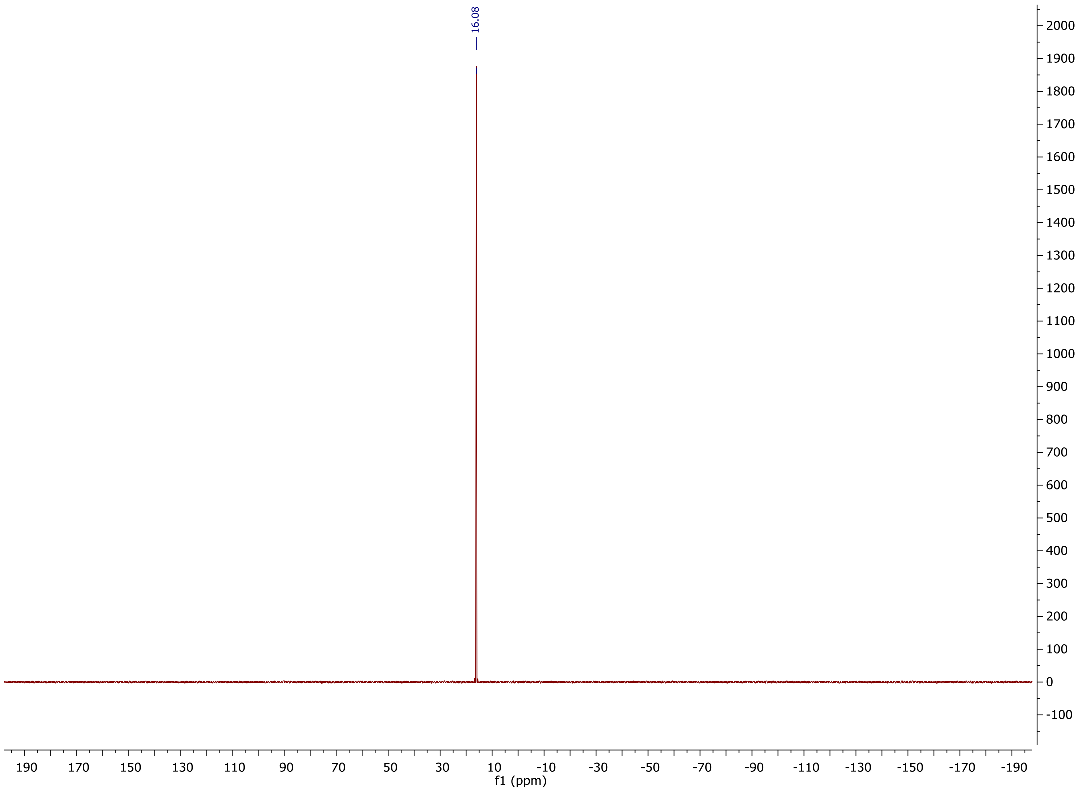

Compound **37**

^{1}H NMR (400 MHz, $CDCl_3$):

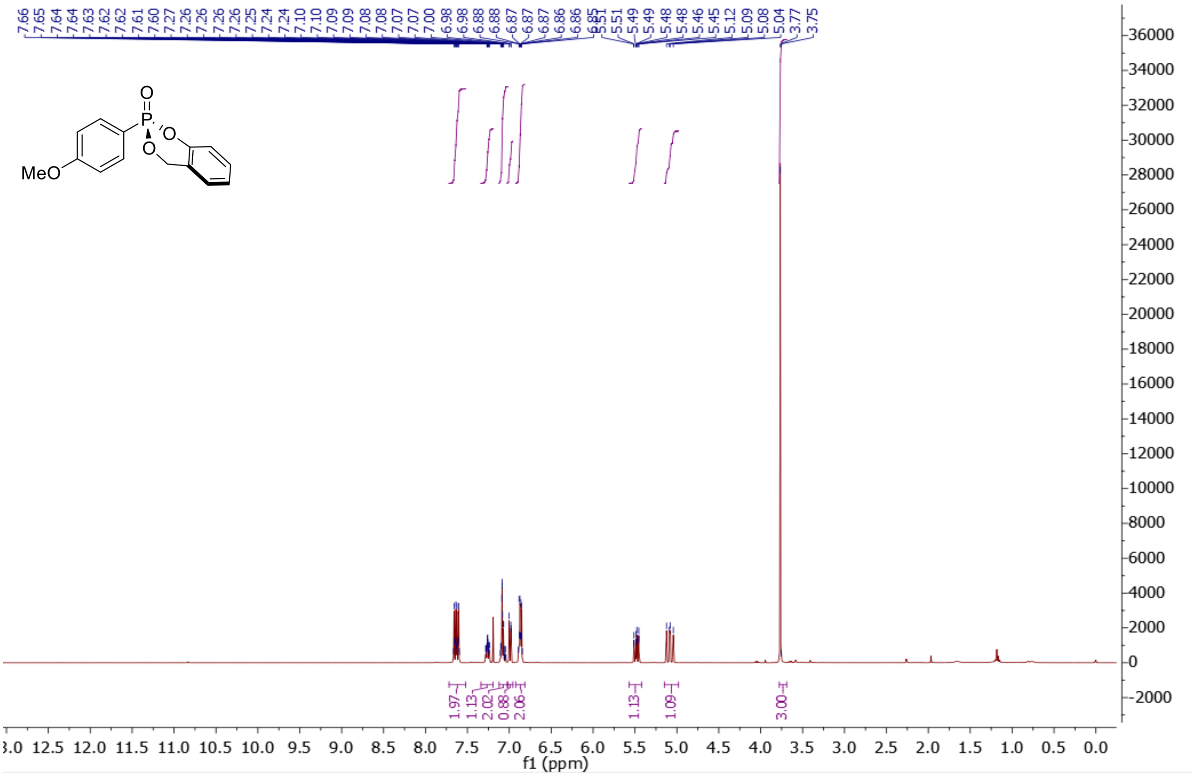

^{13}C NMR (101 MHz, $CDCl_3$):

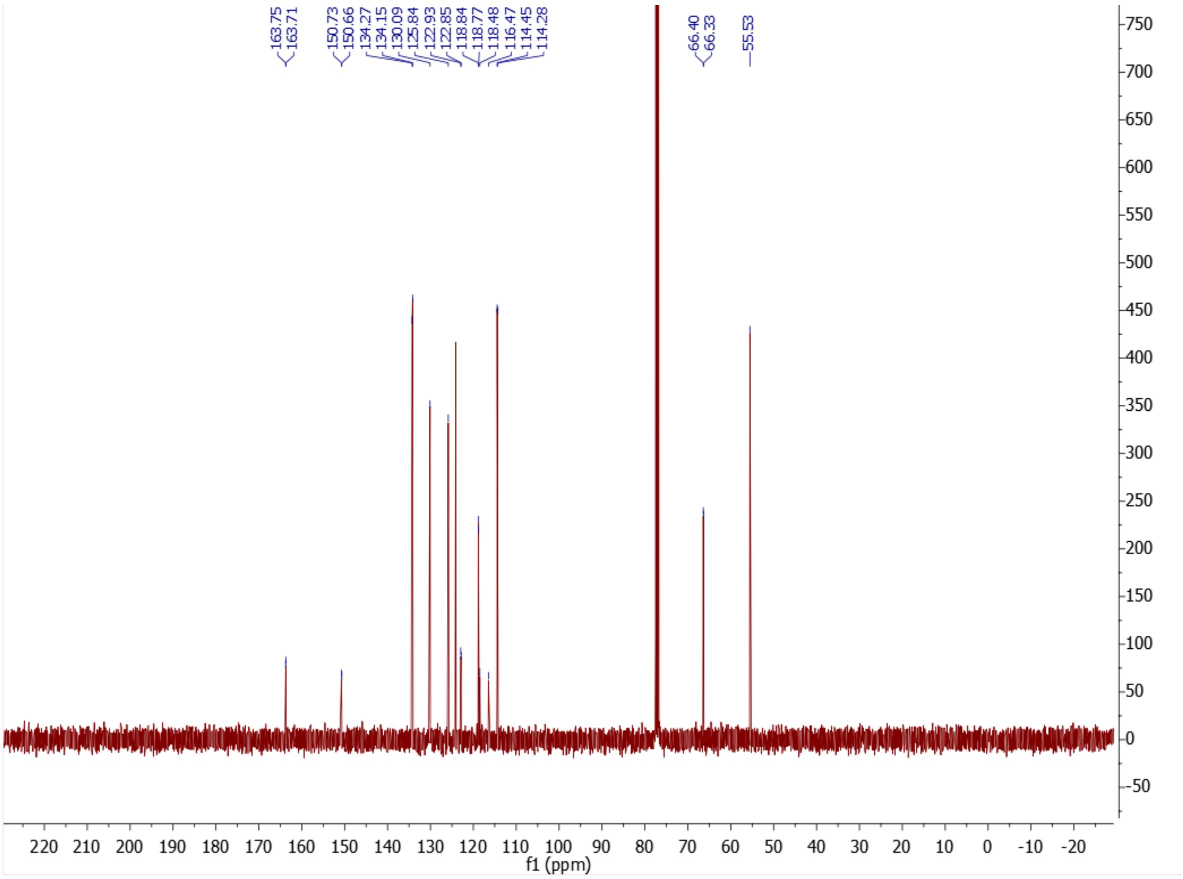

^{31}P NMR (162 MHz, $CDCl_3$):

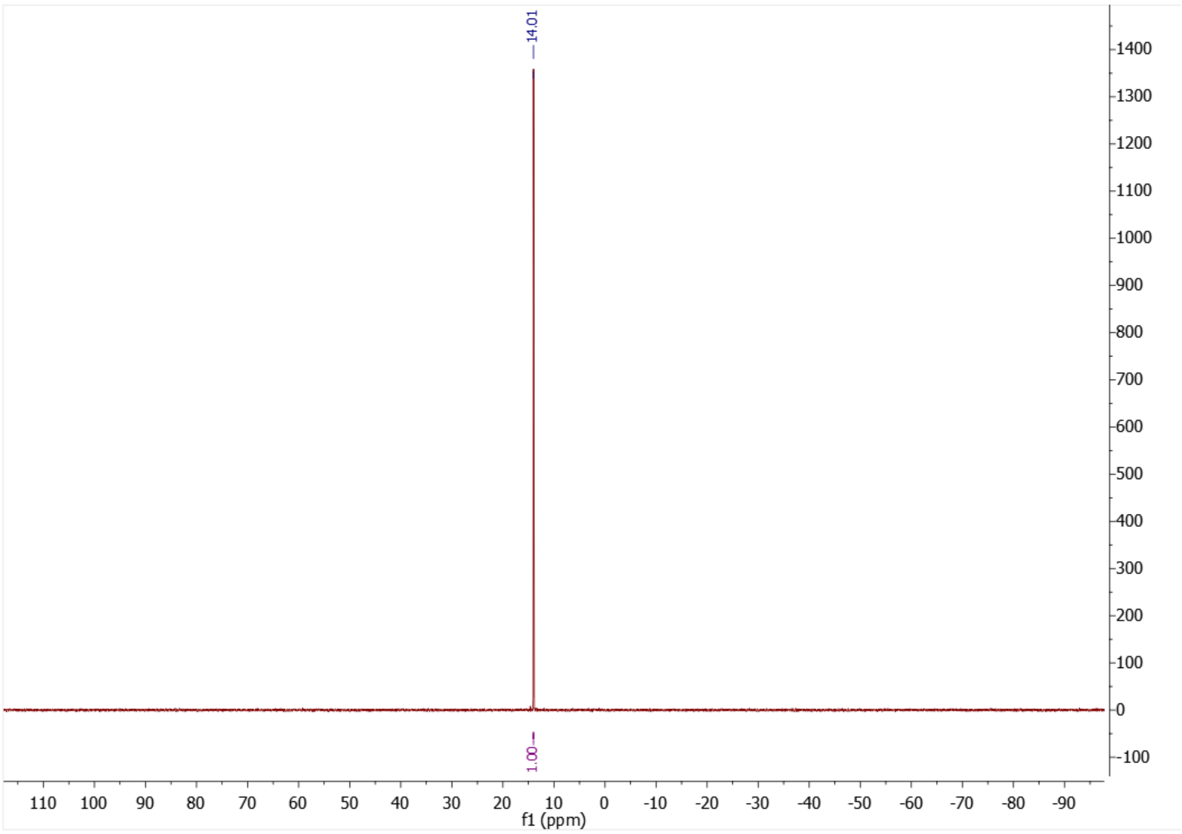

HPLC Traces:

Compound **LGS1**

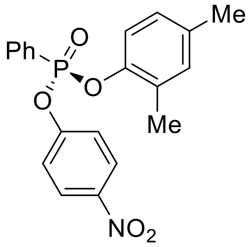

HPLC Conditions: CHIRALPAK IB, hexane/isopropanol = 90/10, 1 mL/min, λ = 220 nm

Racemic

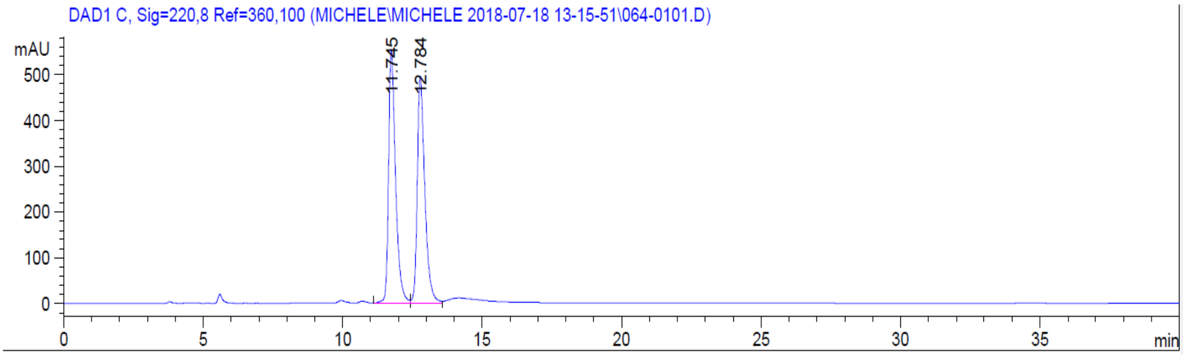

Signal 3: DAD1 C, Sig=220,8 Ref=360,100

Peak #	RetTime [min]	Type	Width [min]	Area [mAU*s]	Height [mAU]	Area %
1	11.745	VV	0.2612	9642.29980	553.73511	50.9983
2	12.784	VV	0.2804	9264.81152	494.91238	49.0017

Compound **LG1**

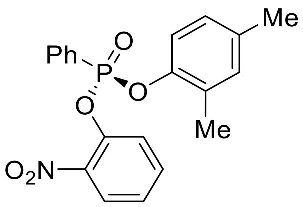

HPLC Conditions: CHIRALPAK AD-H, hexane/isopropanol = 80/20, 1 mL/min, λ = 240 nm

Racemic

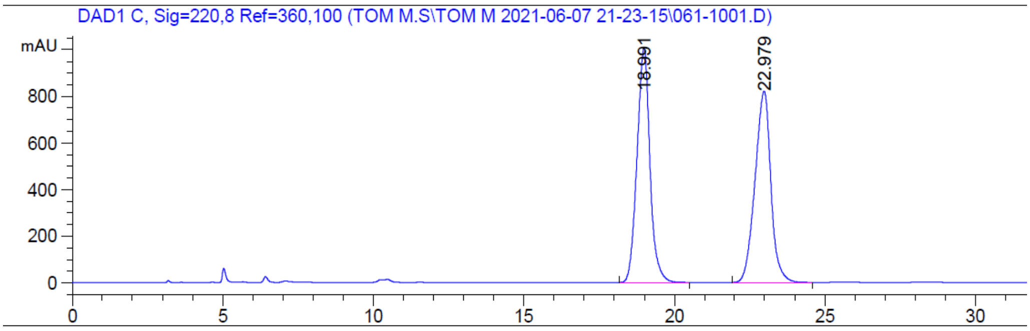

Signal 3: DAD1 C, Sig=220,8 Ref=360,100

Peak #	RetTime [min]	Type	Width [min]	Area [mAU*s]	Height [mAU]	Area %
1	18.991	BB	0.4566	3.00975e4	1007.35535	49.9699
2	22.979	BB	0.5599	3.01339e4	821.67236	50.0301
Totals :				6.02314e4	1829.02771	

Enantioenriched

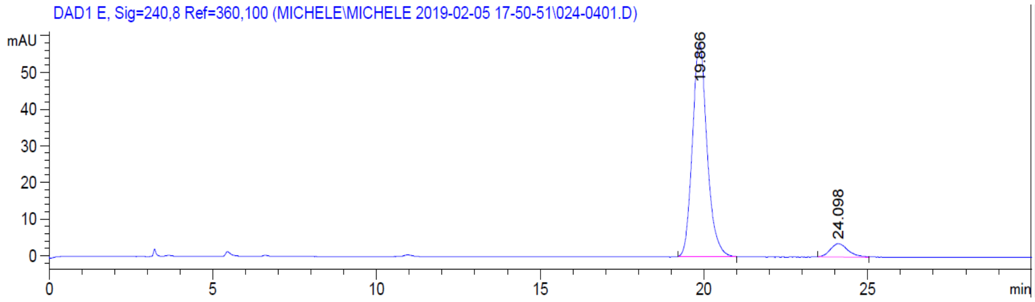

Signal 5: DAD1 E, Sig=240,8 Ref=360,100

Peak #	RetTime [min]	Type	Width [min]	Area [mAU*s]	Height [mAU]	Area %
1	19.866	BB	0.4754	1822.15442	58.49608	92.9866
2	24.098	BB	0.5465	137.43451	3.65659	7.0134

Compound **LG2**

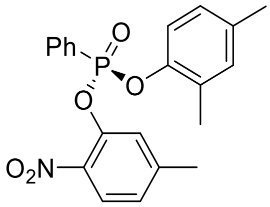

SFC Conditions: CHIRALPAK ID, 1500 psi, 30 °C, flow : 1.5 mL/min, from 1% to 30% MeOH in 5 mins, λ = 220 nm, t (minor) = 3.99 min, t (major) = 4.14 min

Racemic

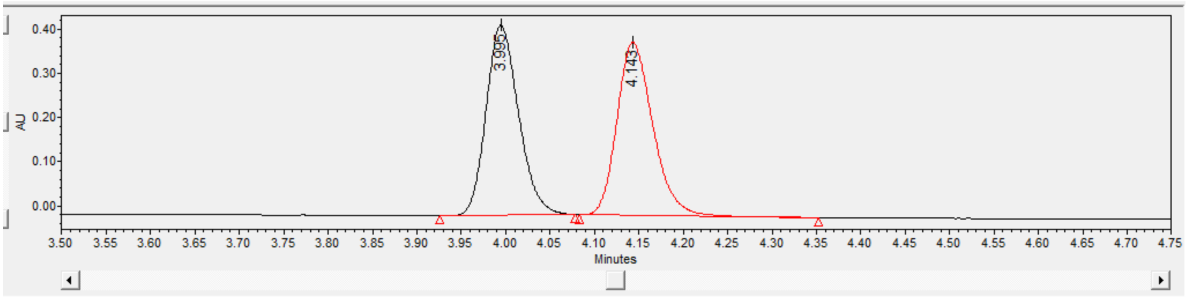

Name	Retention Time (min)	Purity1 Angle	Purity1 Threshold	PDA/FLR Match1 Spect. Name	PDA/FLR Match1 Angle	PDA/FLR Match1 Threshold	PDA/FLR Match1 Lib. Name	Area (µV*sec)	% Area	Height (µV)	Int Type	Amount	Units	Peak Type	Peak Codes
	3.995							1096750	49.88	430121	bb			Unknown	
	4.143							1102206	50.12	391835	bb			Unknown	

Enantioenriched

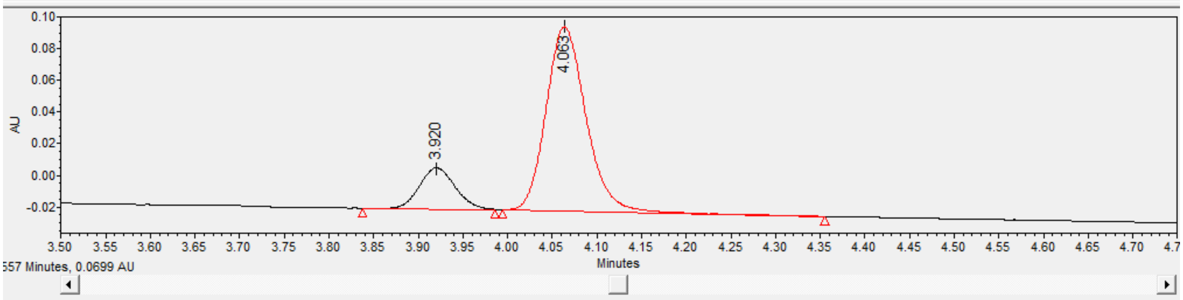

lame	Retention Time (min)	Purity1 Angle	Purity1 Threshold	PDA/FLR Match1 Spect. Name	PDA/FLR Match1 Angle	PDA/FLR Match1 Threshold	PDA/FLR Match1 Lib. Name	Area (µV*sec)	% Area	Height (µV)	Int Type	Amount	Units	Peak Type	Peak Codes
	3.920							71621	16.51	25961	bb			Unknown	
	4.063							362221	83.49	116531	bb			Unknown	

Compound **LG3**

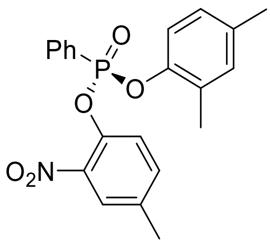

HPLC Conditions: CHIRALPAK AS-H, hexane/isopropanol = gradient 98/2 to 70/30 over 40 min, 1 mL/min, λ = 220 nm

Racemic

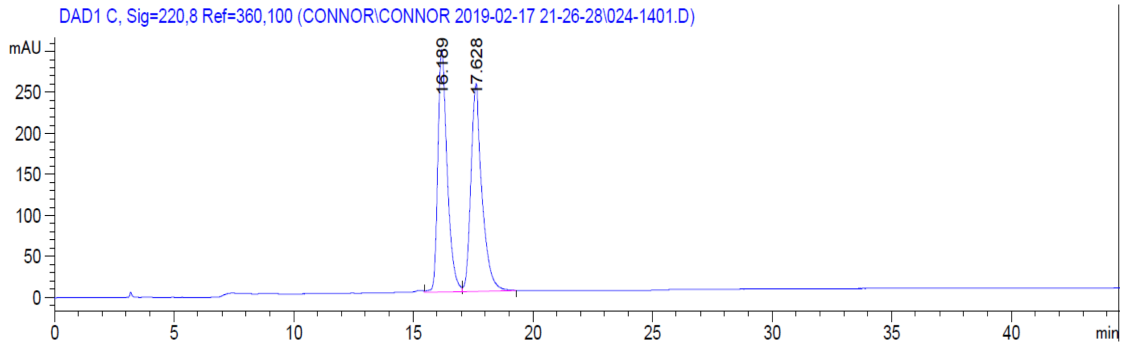

Signal 3: DAD1 C, Sig=220,8 Ref=360,100

Peak #	RetTime [min]	Type	Width [min]	Area [mAU*s]	Height [mAU]	Area %
1	16.189	VV	0.4116	8045.51904	295.51743	49.7753
2	17.628	VB	0.4593	8118.15918	253.69937	50.2247

Enantioenriched

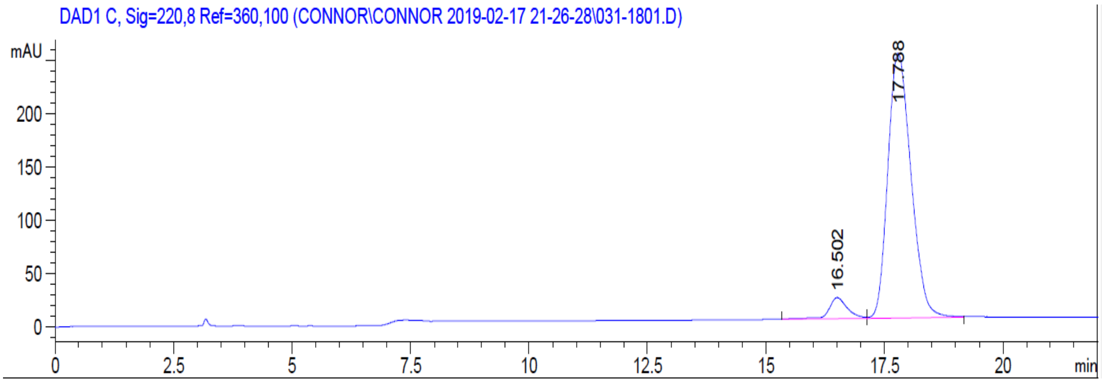

Signal 3: DAD1 C, Sig=220,8 Ref=360,100

Peak #	RetTime [min]	Type	Width [min]	Area [mAU*s]	Height [mAU]	Area %
1	16.502	BV	0.3910	522.44519	19.72477	5.8219
2	17.788	VB	0.5330	8451.36621	248.35152	94.1781

Compound **1**

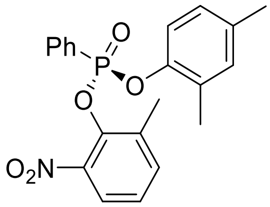

HPLC Conditions: CHIRALPAK AD-H, hexane/isopropanol = 80/20, 1 mL/min, λ = 220 nm

Racemic

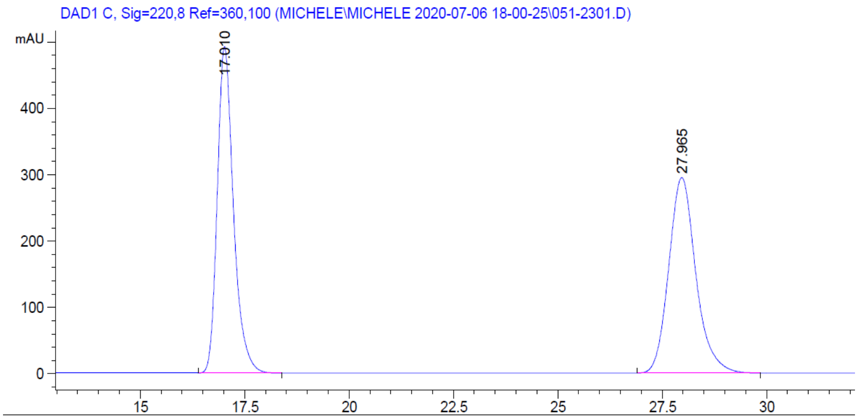

Signal 3: DAD1 C, Sig=220,8 Ref=360,100

Peak #	RetTime [min]	Type	Width [min]	Area [mAU*s]	Height [mAU]	Area %
1	17.010	BB	0.4116	1.33116e4	492.13776	49.9302
2	27.965	BB	0.6918	1.33488e4	295.05862	50.0698

Enantioenriched

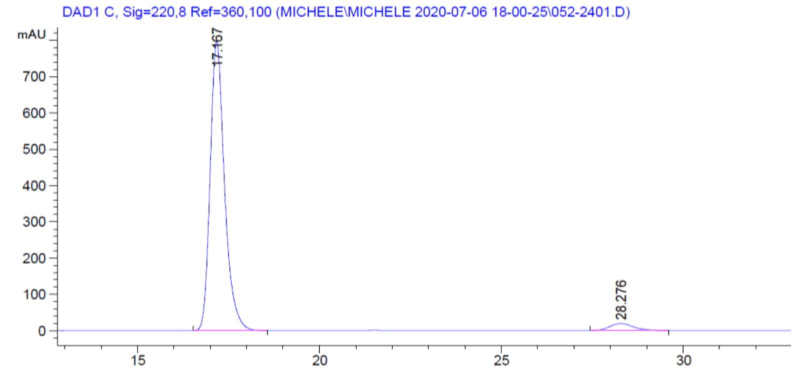

Signal 3: DAD1 C, Sig=220,8 Ref=360,100

Peak #	RetTime [min]	Type	Width [min]	Area [mAU*s]	Height [mAU]	Area %
1	17.167	BB	0.4168	2.18939e4	796.24591	96.0612
2	28.276	BB	0.6968	897.72900	19.80774	3.9388

Compound **2**

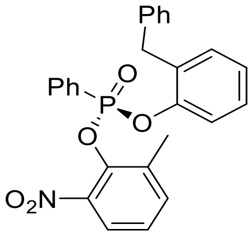

HPLC Conditions: CHIRALPAK AD-H, hexane/isopropanol = 85/15, 1 mL/min, λ = 220 nm

Racemic

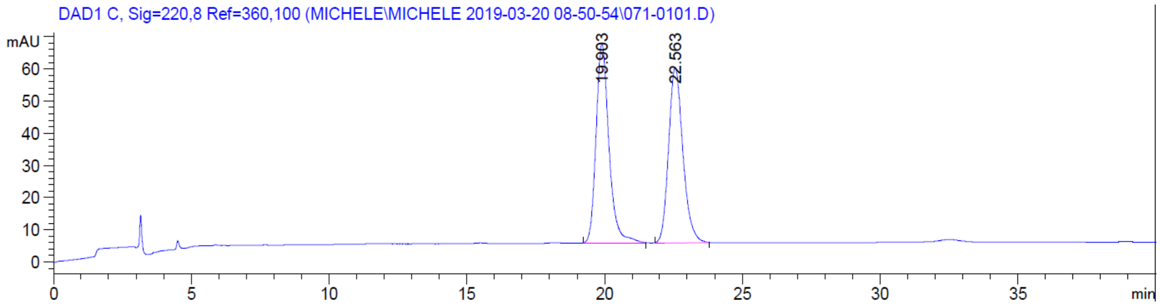

Signal 3: DAD1 C, Sig=220,8 Ref=360,100

Peak #	RetTime [min]	Type	Width [min]	Area [mAU*s]	Height [mAU]	Area %
1	19.903	BB	0.4958	2028.71521	61.99711	50.7259
2	22.563	BB	0.5536	1970.65430	54.52973	49.2741

Enantioenriched

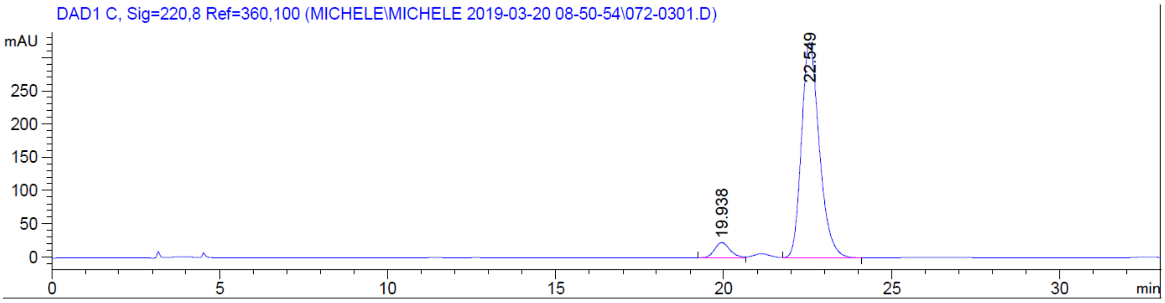

Signal 3: DAD1 C, Sig=220,8 Ref=360,100

Peak #	RetTime [min]	Type	Width [min]	Area [mAU*s]	Height [mAU]	Area %
1	19.938	BV	0.4892	744.75775	23.15643	5.8621
2	22.549	VB	0.5672	1.19598e4	323.55609	94.1379

Compound **3**

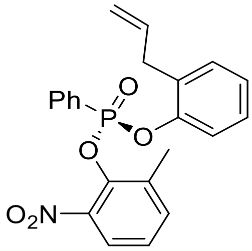

SFC Conditions: CHIRALPAK IC, 1500 psi, 30 °C, flow : 1.5 mL/min, from 1% to 30% MeOH in 5 mins, λ = 220 nm, t (major) = 4.03 min, t (minor) = 4.16 min

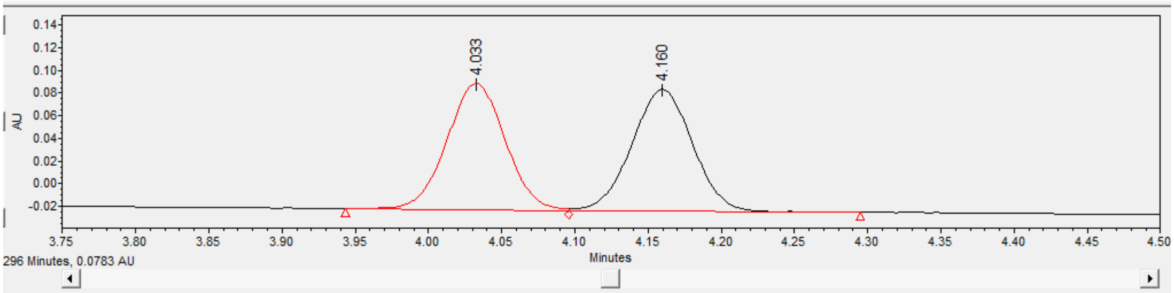

Name	Retention Time (min)	Purity1 Angle	Purity1 Threshold	PDA/FLR Match1 Spect. Name	PDA/FLR Match1 Angle	PDA/FLR Match1 Threshold	PDA/FLR Match1 Lib. Name	Area (µV*sec)	% Area	Height (µV)	Int Type	Amount	Units	Peak Type	Peak Codes
	4.033							308956	49.99	111237	bv			Unknown	
	4.160							309117	50.01	106979	vb			Unknown	

Enantioenriched

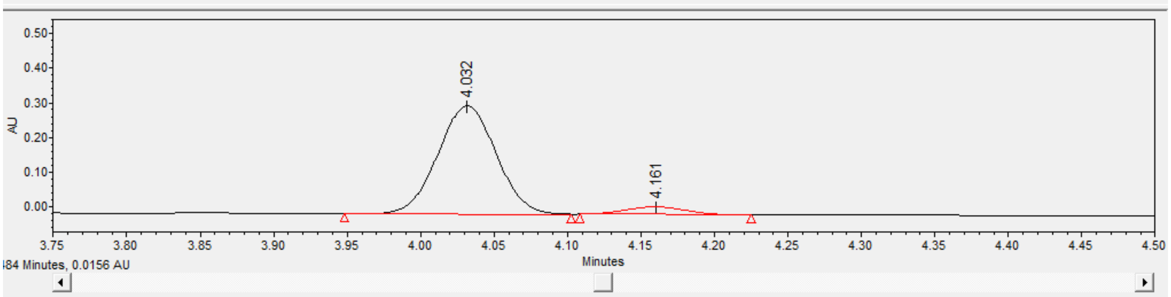

Name	Retention Time (min)	Purity1 Angle	Purity1 Threshold	PDA/FLR Match1 Spect. Name	PDA/FLR Match1 Angle	PDA/FLR Match1 Threshold	PDA/FLR Match1 Lib. Name	Area (µV*sec)	% Area	Height (µV)	Int Type	Amount	Units	Peak Type	Peak Codes
	4.032							862973	93.40	313178	bb			Unknown	
	4.161							61021	6.60	22001	bb			Unknown	

Compound **4**

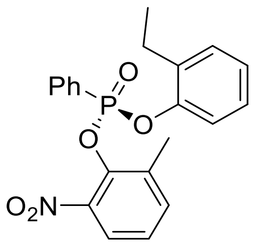

HPLC Conditions: CHIRALPAK AD-H, hexane/isopropanol = 85/15, 1 mL/min, λ = 220 nm

Racemic

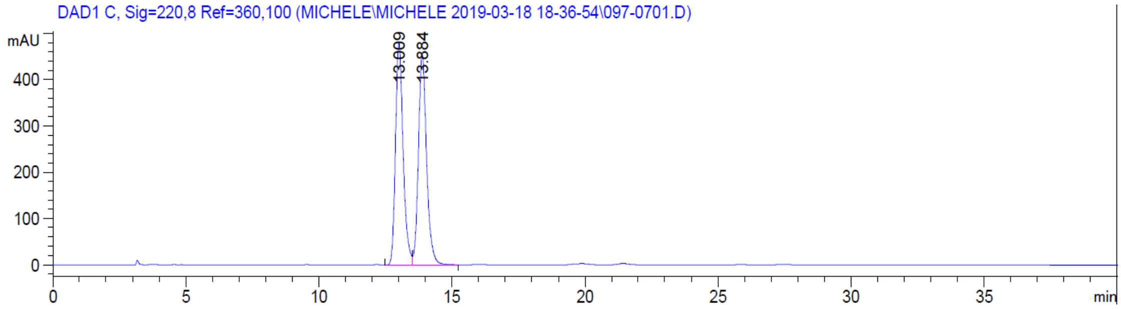

Signal 3: DAD1 C, Sig=220,8 Ref=360,100

Peak #	RetTime [min]	Type	Width [min]	Area [mAU*s]	Height [mAU]	Area %
1	13.009	VV	0.3050	9601.35059	480.67285	49.6219
2	13.884	VB	0.3265	9747.65234	453.97797	50.3781

Enantioenriched

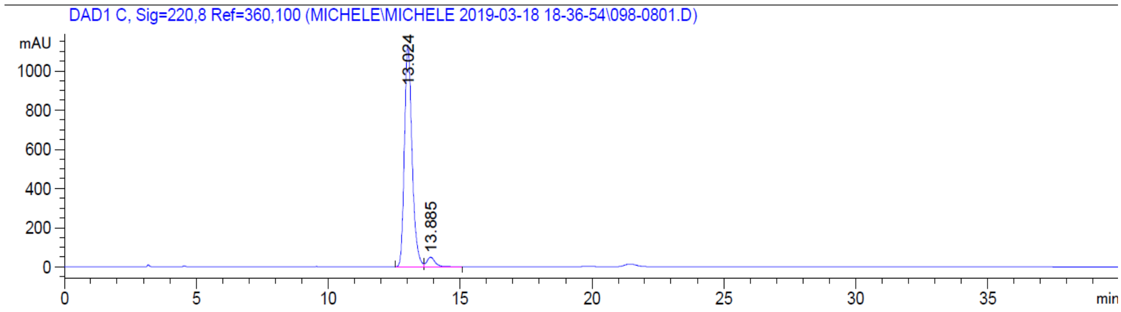

Signal 3: DAD1 C, Sig=220,8 Ref=360,100

Peak #	RetTime [min]	Type	Width [min]	Area [mAU*s]	Height [mAU]	Area %
1	13.024	BV	0.3083	2.27408e4	1132.22363	95.3855
2	13.885	VB	0.3315	1100.15039	49.45401	4.6145

Compound **5**

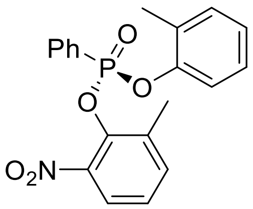

HPLC Conditions: CHIRALPAK AD-H, hexane/isopropanol = 80/20, 1 mL/min, λ = 220 nm

Racemic

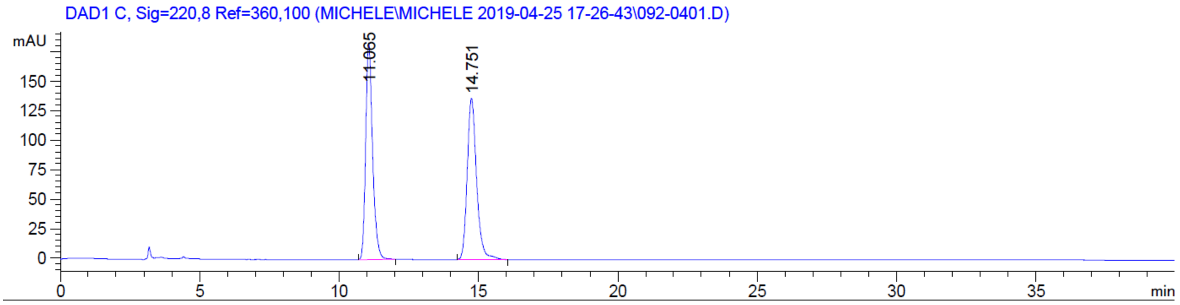

Signal 3: DAD1 C, Sig=220,8 Ref=360,100

Peak #	RetTime [min]	Type	Width [min]	Area [mAU*s]	Height [mAU]	Area %
1	11.065	BB	0.2604	3155.31763	183.65144	49.5629
2	14.751	BB	0.3558	3210.96851	136.84337	50.4371

Enantioenriched

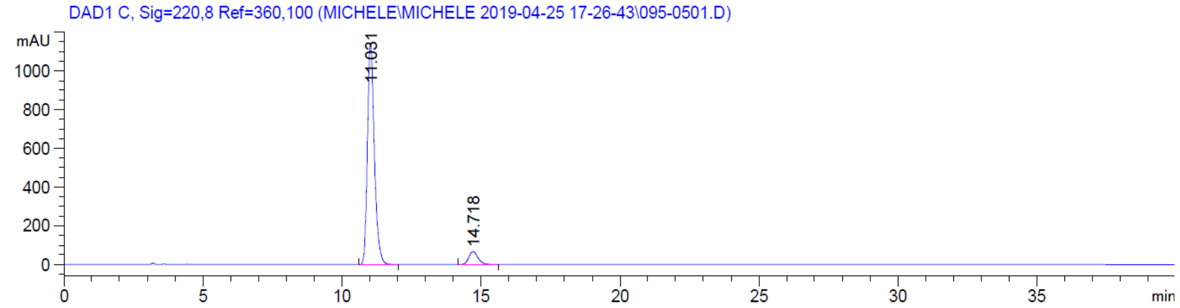

Signal 3: DAD1 C, Sig=220,8 Ref=360,100

Peak #	RetTime [min]	Type	Width [min]	Area [mAU*s]	Height [mAU]	Area %
1	11.031	BB	0.2627	1.97179e4	1146.07458	92.6404
2	14.718	BB	0.3510	1566.44568	67.94541	7.3596

Compound **6**

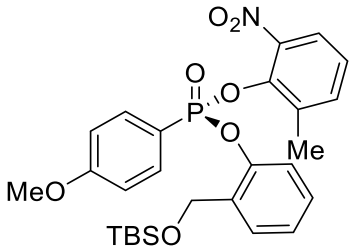

HPLC Conditions: CHIRALPAK IA, hexane/isopropanol = 90/10, 1 mL/min, λ = 220 nm

Racemic

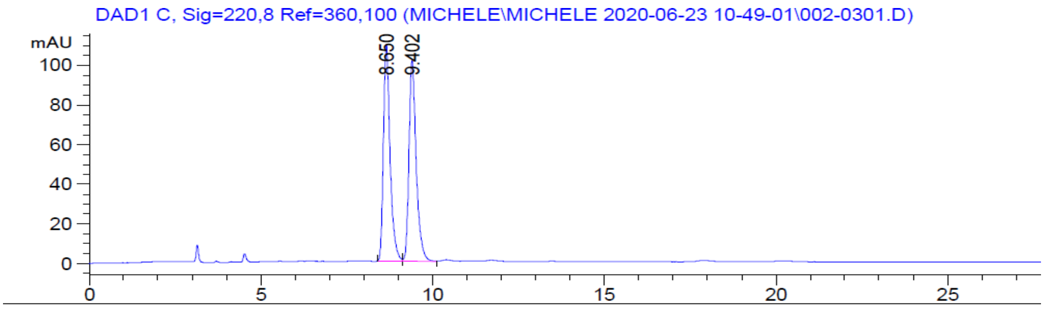

Signal 3: DAD1 C, Sig=220,8 Ref=360,100

Peak #	RetTime [min]	Type	Width [min]	Area [mAU*s]	Height [mAU]	Area %
1	8.650	BV	0.2030	1471.63525	109.32714	49.8199
2	9.402	VB	0.2208	1482.27271	101.25576	50.1801

Enantioenriched

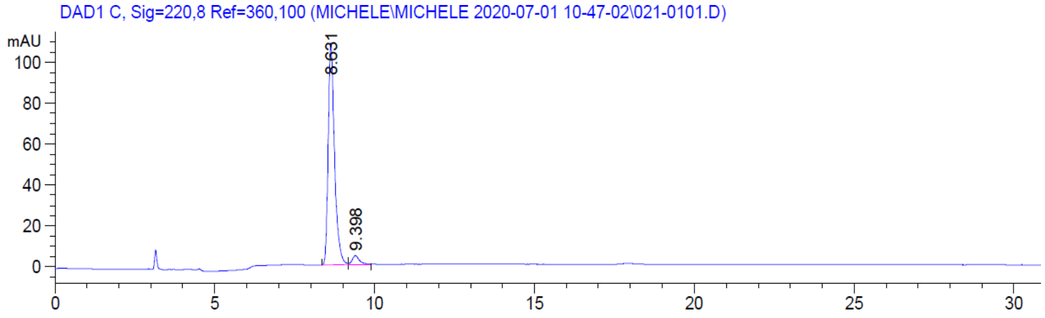

Signal 3: DAD1 C, Sig=220,8 Ref=360,100

Peak #	RetTime [min]	Type	Width [min]	Area [mAU*s]	Height [mAU]	Area %
1	8.631	BV	0.2057	1483.18665	108.37539	95.5839
2	9.398	VB	0.2304	68.52556	4.38302	4.4161

Compound **7**

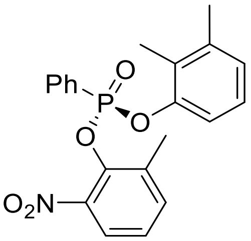

HPLC Conditions: CHIRALPAK IA, hexane/isopropanol = 85/15, 1 mL/min, λ = 220 nm

Racemic

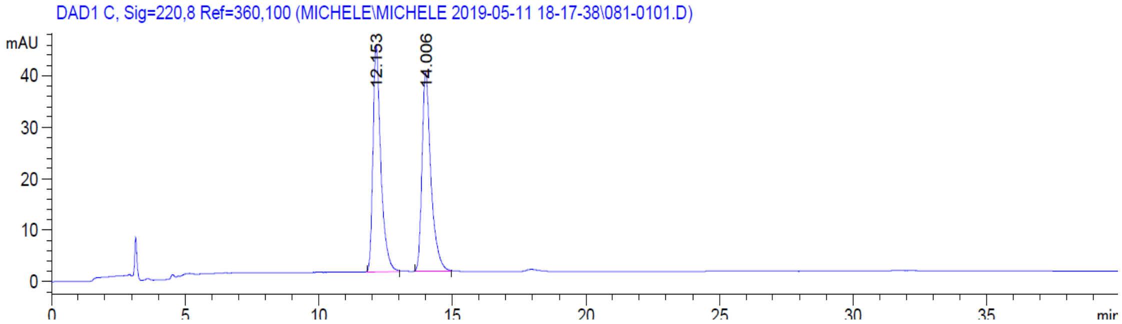

Signal 3: DAD1 C, Sig=220,8 Ref=360,100

Peak #	RetTime [min]	Type	Width [min]	Area [mAU*s]	Height [mAU]	Area %
1	12.153	BB	0.3046	900.83832	44.04750	50.0374
2	14.006	BB	0.3464	899.49158	38.51994	49.9626

Enantioenriched

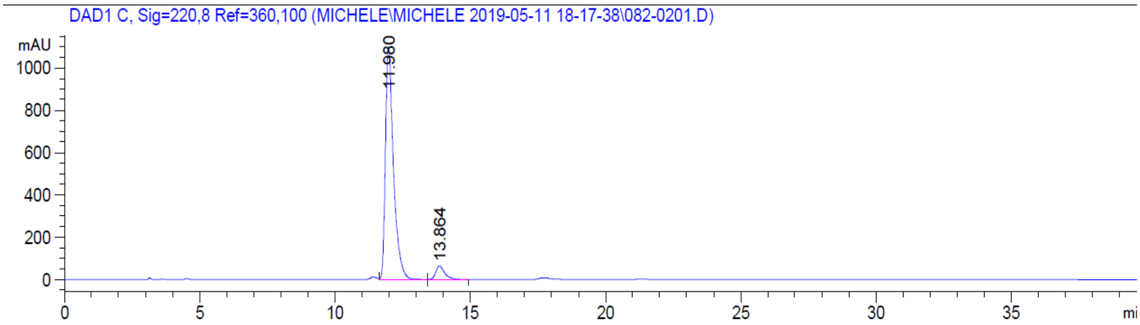

Signal 3: DAD1 C, Sig=220,8 Ref=360,100

Peak #	RetTime [min]	Type	Width [min]	Area [mAU*s]	Height [mAU]	Area %
1	11.980	VV	0.3092	2.27030e4	1098.15210	93.7270
2	13.864	VB	0.3489	1519.46631	64.95222	6.2730

Compound **8**

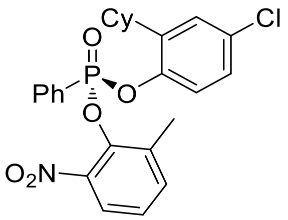

HPLC Conditions: CHIRALPAK AD-H, hexane/isopropanol = 90/10, 1 mL/min, λ = 220 nm

Racemic

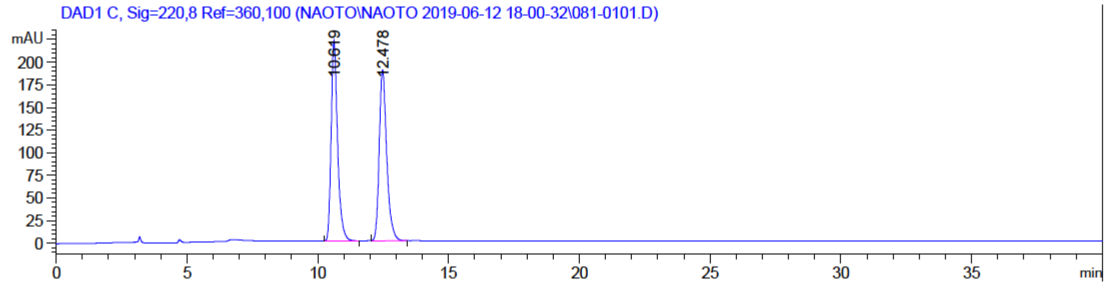

Peak #	RetTime [min]	Type	Width [min]	Area [mAU*s]	Height [mAU]	Area %
1	10.619	BB	0.2590	3775.73901	221.40659	50.1079
2	12.478	BB	0.3023	3759.48535	188.85010	49.8921

Enantioenriched

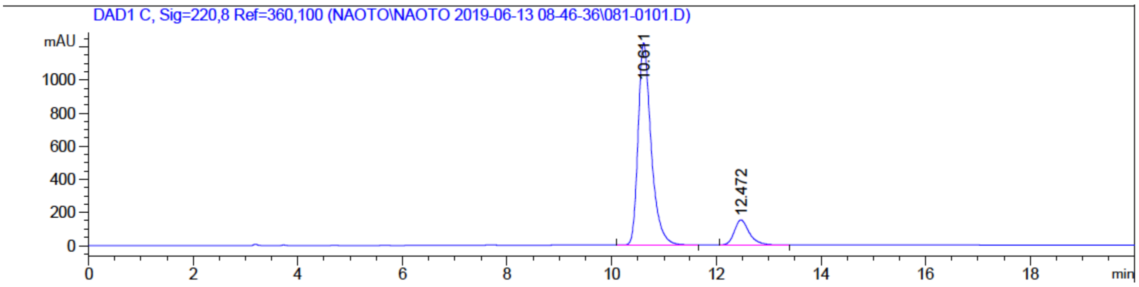

Peak #	RetTime [min]	Type	Width [min]	Area [mAU*s]	Height [mAU]	Area %
1	10.611	BV	0.2626	2.12423e4	1223.22949	87.3256
2	12.472	VB	0.3045	3083.10889	152.11086	12.6744

Compound **10**

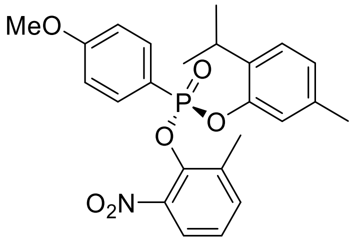

HPLC Conditions: CHIRALPAK AD-H, hexane/isopropanol = 85/15, 1 mL/min, λ = 220 nm

Racemic

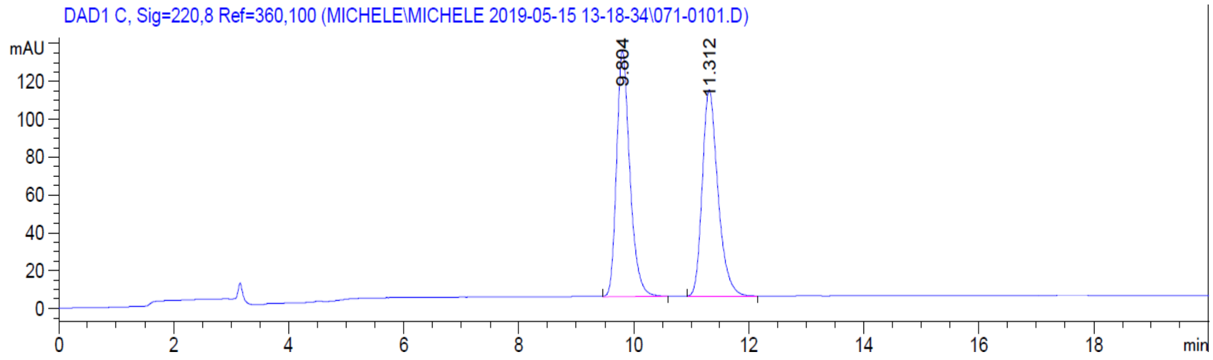

Signal 3: DAD1 C, Sig=220,8 Ref=360,100

Peak #	RetTime [min]	Type	Width [min]	Area [mAU*s]	Height [mAU]	Area %
1	9.804	BB	0.2428	2084.09766	130.13499	50.1572
2	11.312	BB	0.2891	2071.03394	109.30006	49.8428

Enantioenriched

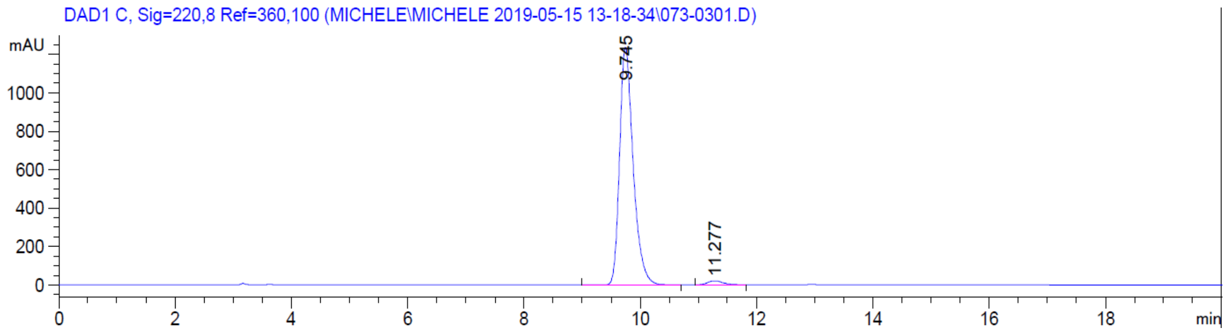

Signal 3: DAD1 C, Sig=220,8 Ref=360,100

Peak #	RetTime [min]	Type	Width [min]	Area [mAU*s]	Height [mAU]	Area %
1	9.745	BB	0.2449	2.00372e4	1237.26550	98.0044
2	11.277	BV	0.2899	408.01013	21.45067	1.9956

Compound **11**

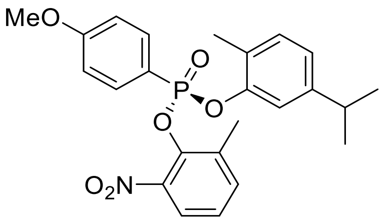

HPLC Conditions: CHIRALPAK AD-H, hexane/isopropanol = 85/15, 1 mL/min, λ = 220 nm

Racemic

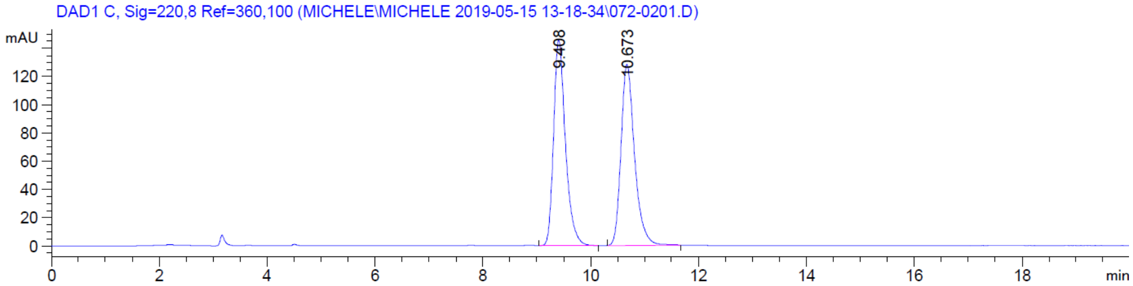

Signal 3: DAD1 C, Sig=220,8 Ref=360,100

Peak #	RetTime [min]	Type	Width [min]	Area [mAU*s]	Height [mAU]	Area %
1	9.408	VB	0.2317	2222.08130	145.88370	49.7533
2	10.673	BB	0.2640	2244.11304	128.35904	50.2467

Enantioenriched

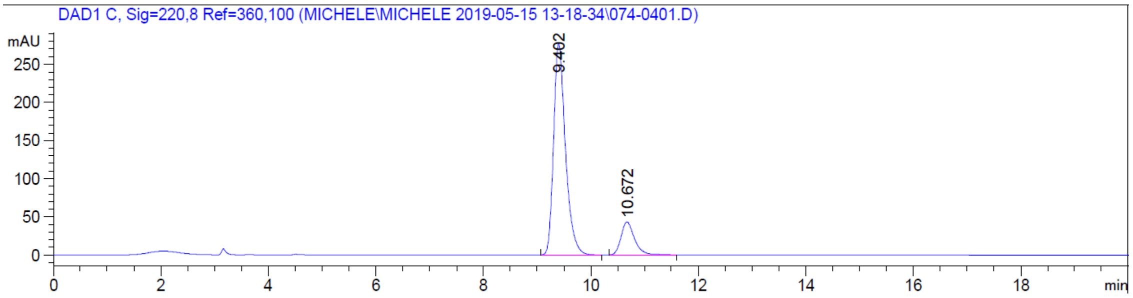

Signal 3: DAD1 C, Sig=220,8 Ref=360,100

Peak #	RetTime [min]	Type	Width [min]	Area [mAU*s]	Height [mAU]	Area %
1	9.402	BB	0.2322	4240.64258	277.58087	84.7654
2	10.672	BB	0.2651	762.15485	43.34430	15.2346

Compound **12**

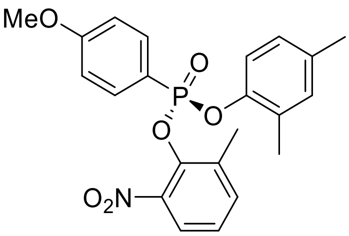

HPLC Conditions: CHIRALPAK AD-H, hexane/isopropanol = 70/30, 1 mL/min, λ = 220 nm

Racemic

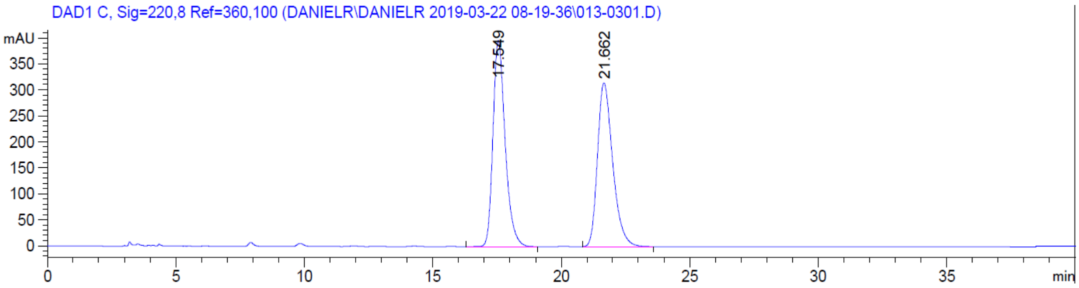

Signal 3: DAD1 C, Sig=220,8 Ref=360,100

Peak #	RetTime [min]	Type	Width [min]	Area [mAU*s]	Height [mAU]	Area %
1	17.549	VB	0.5015	1.31218e4	397.15637	50.2136
2	21.662	BB	0.6293	1.30101e4	315.35538	49.7864

Enantioenriched

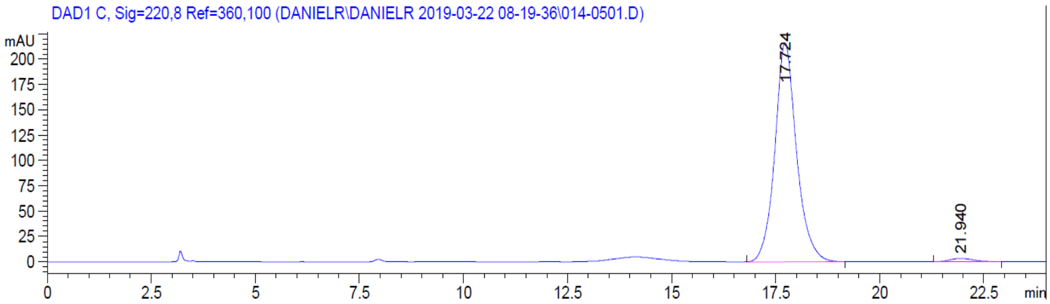

Signal 3: DAD1 C, Sig=220,8 Ref=360,100

Peak #	RetTime [min]	Type	Width [min]	Area [mAU*s]	Height [mAU]	Area %
1	17.724	BB	0.5416	7842.09229	215.98657	98.4761
2	21.940	BB	0.5821	121.35773	3.20276	1.5239

Compound **13**

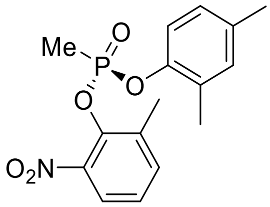

HPLC Conditions: CHIRALPAK AS-H, hexane/isopropanol = 90/10, 1 mL/min, λ = 220 nm

Racemic

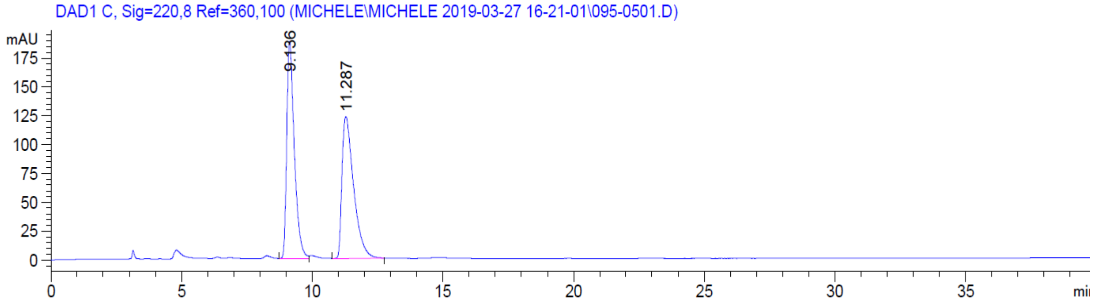

Signal 3: DAD1 C, Sig=220,8 Ref=360,100

Peak #	RetTime [min]	Type	Width [min]	Area [mAU*s]	Height [mAU]	Area %
1	9.136	VV	0.3035	3803.33862	188.38530	49.9897
2	11.287	VB	0.4689	3804.90259	123.00893	50.0103

Enantioenriched

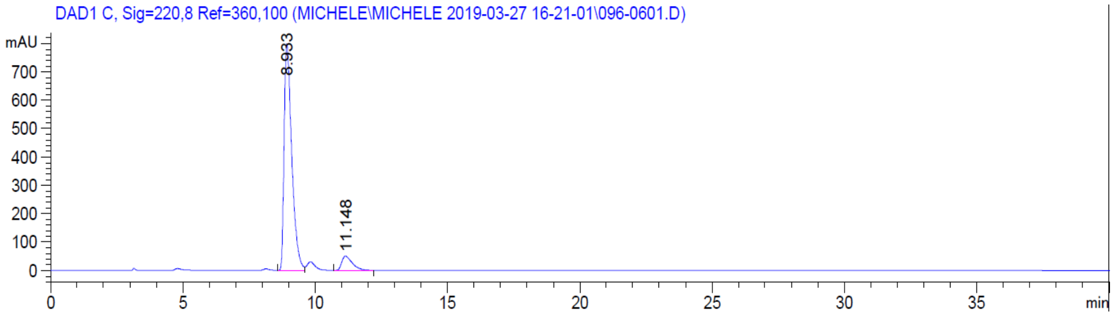

Signal 3: DAD1 C, Sig=220,8 Ref=360,100

Peak #	RetTime [min]	Type	Width [min]	Area [mAU*s]	Height [mAU]	Area %
1	8.933	VV	0.3042	1.60199e4	797.94836	91.9739
2	11.148	BB	0.4262	1397.98279	50.00701	8.0261

Compound **14**

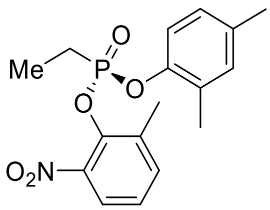

HPLC Conditions: CHIRALPAK AS-H, hexane/isopropanol =90/10, 1 mL/min, λ = 220 nm

Racemic

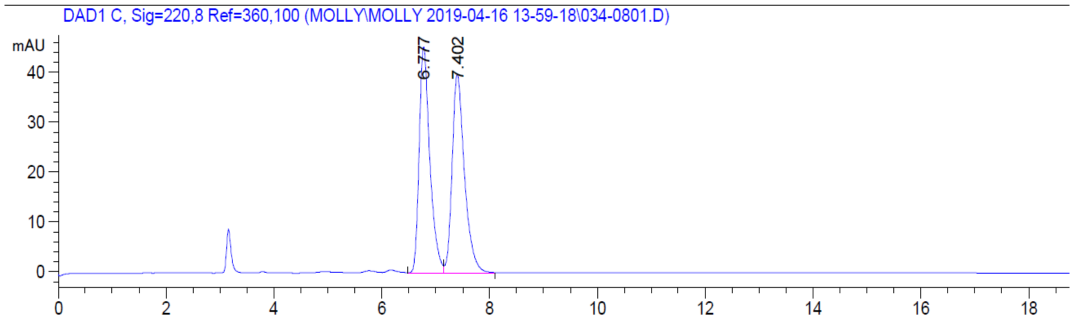

Signal 3: DAD1 C, Sig=220,8 Ref=360,100

Peak #	RetTime [min]	Type	Width [min]	Area [mAU*s]	Height [mAU]	Area %
1	6.777	VV	0.2060	621.21954	45.29765	49.4968
2	7.402	VB	0.2398	633.85175	39.80562	50.5032

Enantioenriched

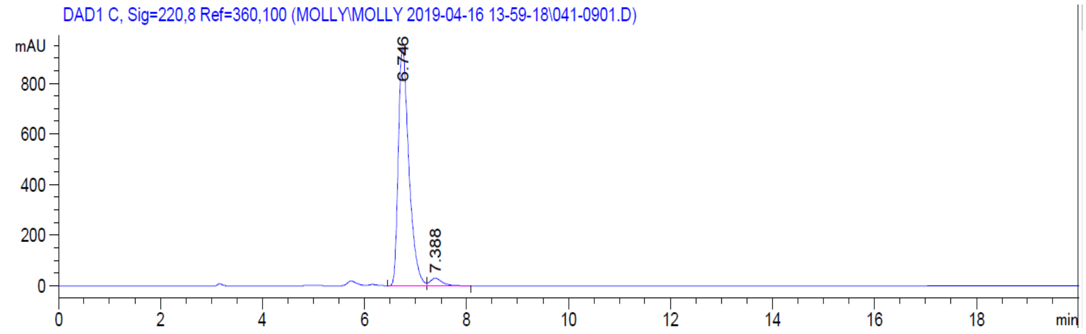

Signal 3: DAD1 C, Sig=220,8 Ref=360,100

Peak #	RetTime [min]	Type	Width [min]	Area [mAU*s]	Height [mAU]	Area %
1	6.746	VV	0.2179	1.35665e4	942.75897	96.4896
2	7.388	VB	0.2427	493.56995	30.19668	3.5104

Compound **15**

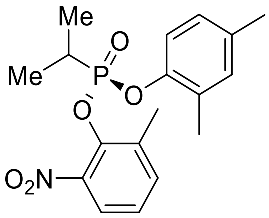

HPLC Conditions: CHIRALPAK AD-H, hexane/isopropanol = 85/15, 1 mL/min, λ = 220 nm, t (major) = 11.11 min, t (minor) = 12.95 min

Racemic

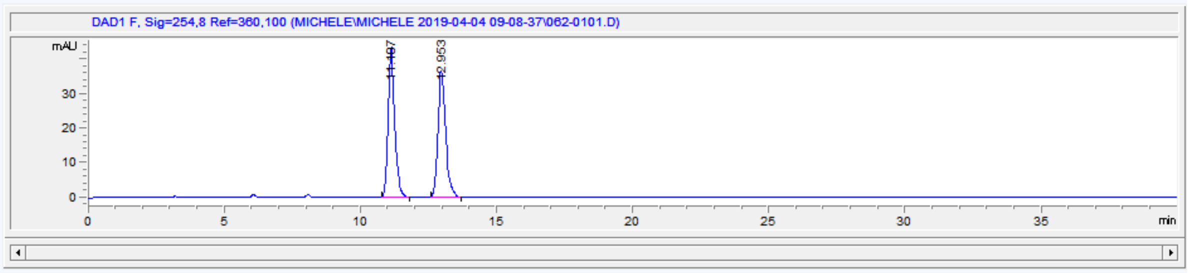

#	Time	Area	Height	Width	Area%	Symmetry
1	11.107	736.1	43.5	0.2576	50.098	0.73
2	12.953	733.2	36.6	0.3017	49.902	0.743

Enantioenriched

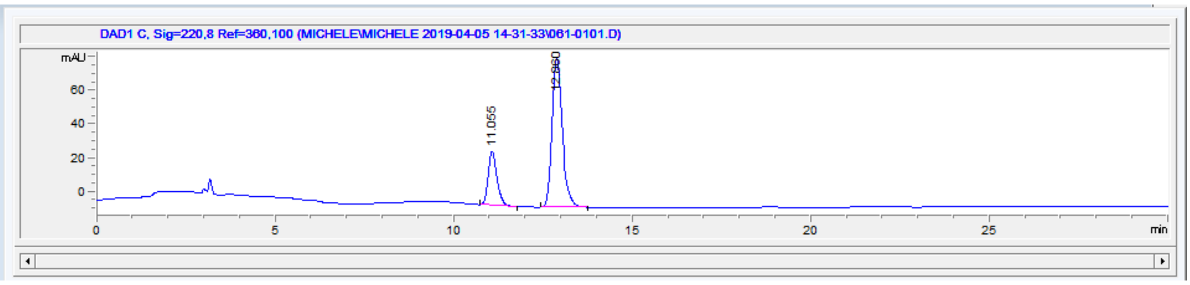

#	Time	Area	Height	Width	Area%	Symmetry
1	11.055	528.8	32	0.2509	23.032	0.749
2	12.86	1767.2	88.3	0.3035	76.968	0.737

Compound **16**

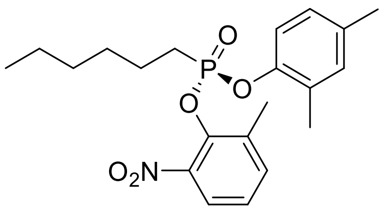

HPLC Conditions: CHIRALPAK AD-H, hexane/isopropanol = 85/15, 1 mL/min, λ = 220 nm

Racemic

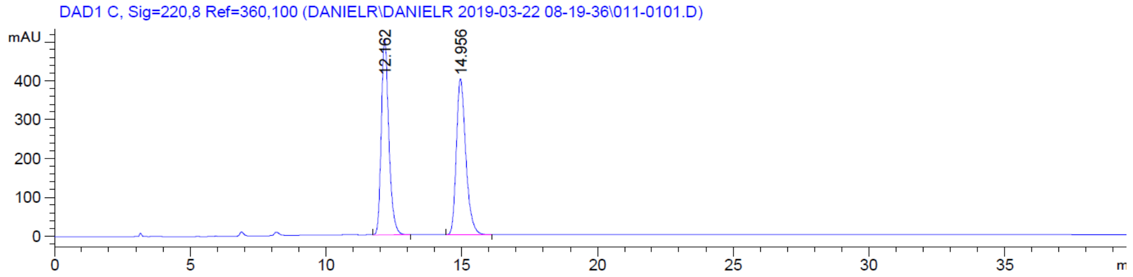

Signal 3: DAD1 C, Sig=220,8 Ref=360,100

Peak #	RetTime [min]	Type	Width [min]	Area [mAU*s]	Height [mAU]	Area %
1	12.162	BB	0.2947	9717.44043	504.46967	49.9640
2	14.956	BB	0.3706	9731.44141	401.72116	50.0360

Enantioenriched

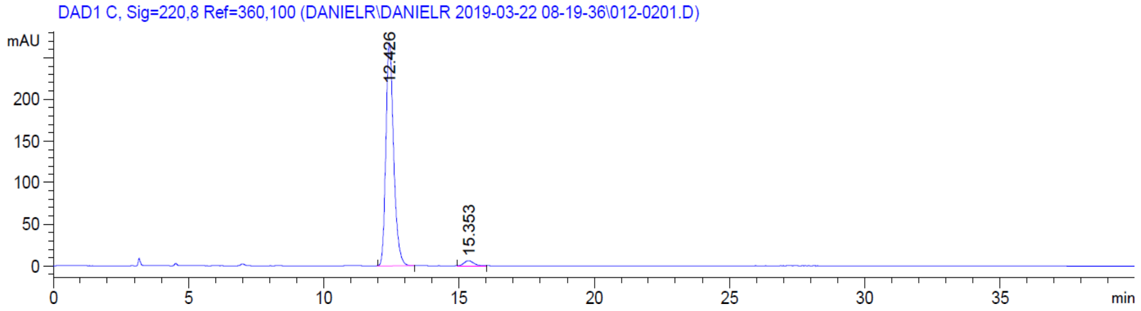

Signal 3: DAD1 C, Sig=220,8 Ref=360,100

Peak #	RetTime [min]	Type	Width [min]	Area [mAU*s]	Height [mAU]	Area %
1	12.426	BB	0.3049	5352.95215	268.15628	97.1945
2	15.353	BB	0.3802	154.51390	6.25500	2.8055

Compound **17**

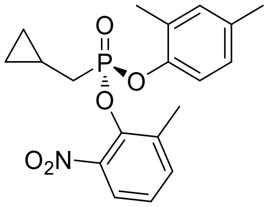

HPLC Conditions: CHIRALPAK AD-H, hexane/isopropanol = 80/20, 1 mL/min, λ = 220 nm

Racemic

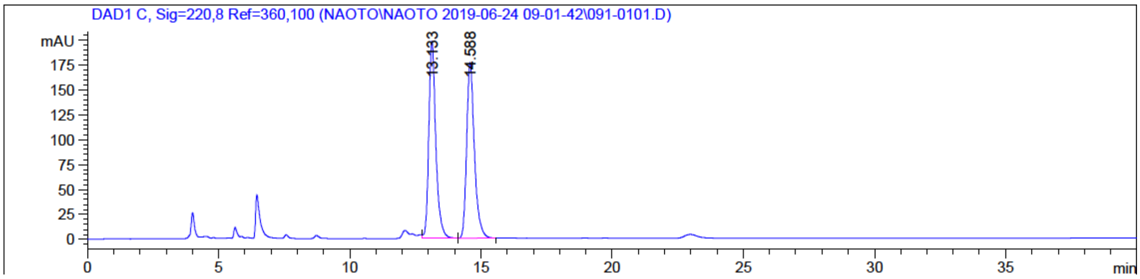

Peak #	RetTime [min]	Type	Width [min]	Area [mAU*s]	Height [mAU]	Area %
1	13.133	VV	0.2804	3663.75757	197.54814	50.2535
2	14.588	VB	0.3121	3626.80151	176.22464	49.7465

Enantioenriched

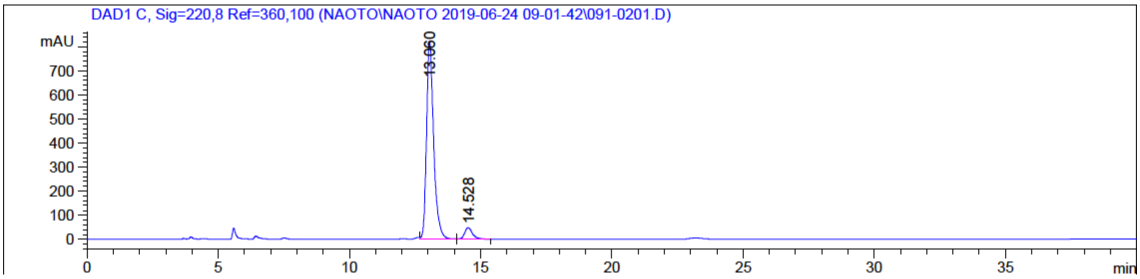

Peak #	RetTime [min]	Type	Width [min]	Area [mAU*s]	Height [mAU]	Area %
1	13.060	VV	0.2838	1.55022e4	822.68829	93.7807
2	14.528	VB	0.3181	1028.06995	48.73454	6.2193

Compound **18**

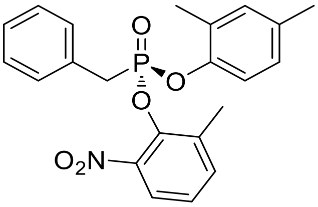

HPLC Conditions: CHIRALPAK AD-H, hexane/isopropanol = 70/30, 1 mL/min, λ = 220 nm

Racemic

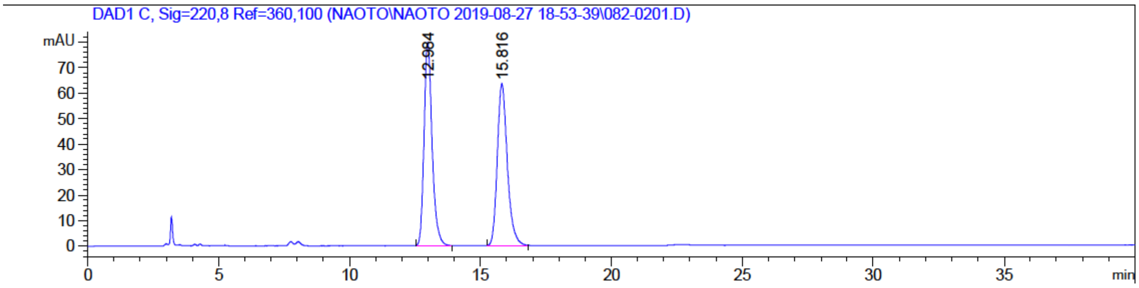

Peak #	RetTime [min]	Type	Width [min]	Area [mAU*s]	Height [mAU]	Area %
1	12.984	BB	0.3250	1702.50623	79.78445	50.1675
2	15.816	BB	0.4046	1691.14050	63.51892	49.8325

Enantioenriched

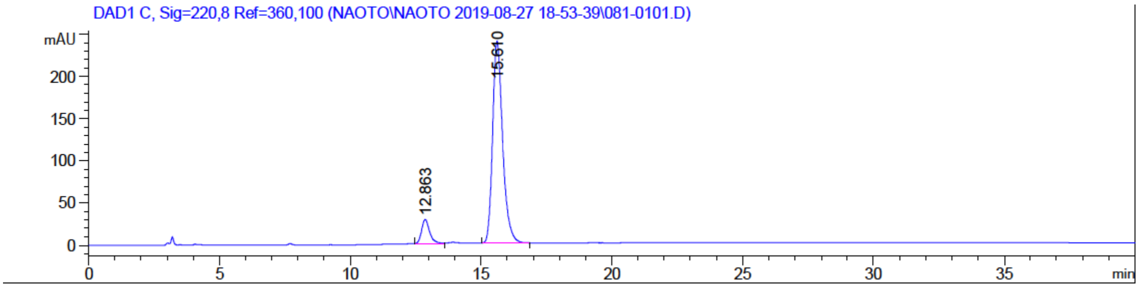

Peak #	RetTime [min]	Type	Width [min]	Area [mAU*s]	Height [mAU]	Area %
1	12.863	BB	0.3233	610.96863	28.59405	8.7585
2	15.610	BB	0.4066	6364.76172	239.06133	91.2415

Compound **19**

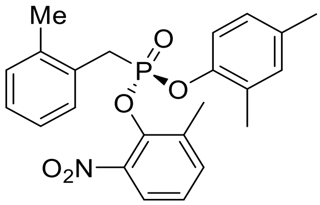

HPLC Conditions: CHIRALPAK AD-H, hexane/isopropanol = 80/20, 1 mL/min, λ = 220 nm

Racemic

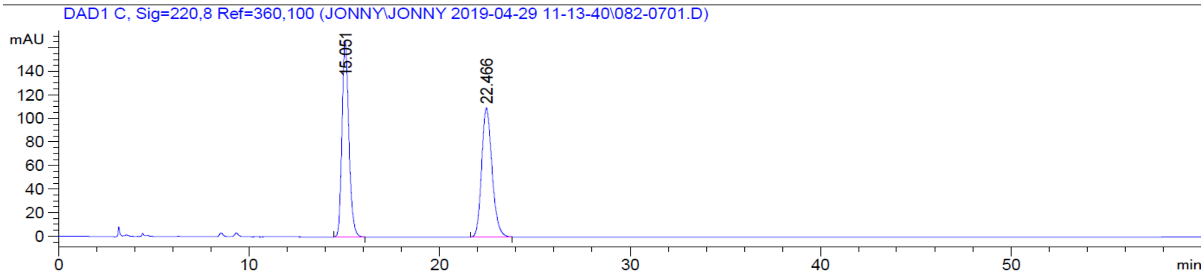

Signal 3: DAD1 C, Sig=220,8 Ref=360,100

Peak #	RetTime [min]	Type	Width [min]	Area [mAU*s]	Height [mAU]	Area %
1	15.051	BB	0.3835	4163.83008	166.64192	49.9869
2	22.466	BB	0.5836	4166.00635	109.55894	50.0131

Enantioenriched

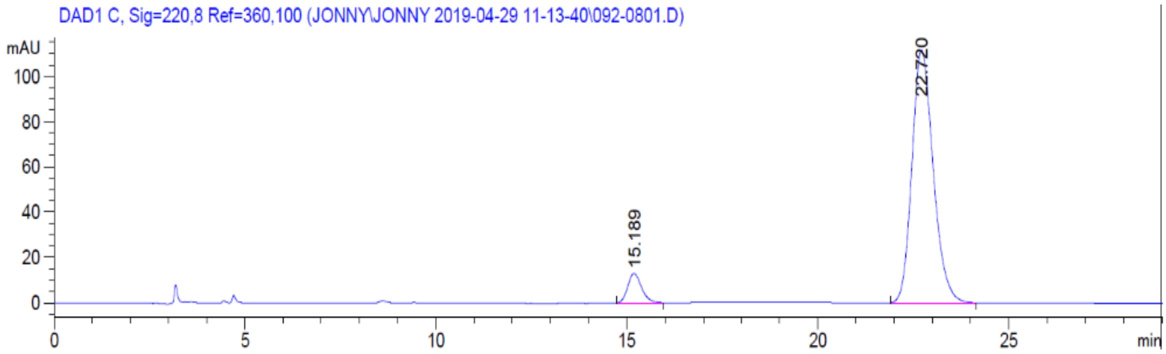

Signal 3: DAD1 C, Sig=220,8 Ref=360,100

Peak #	RetTime [min]	Type	Width [min]	Area [mAU*s]	Height [mAU]	Area %
1	15.189	BB	0.3876	331.40750	13.08106	7.1116
2	22.720	BB	0.5966	4328.71484	111.58073	92.8884

Compound **20**

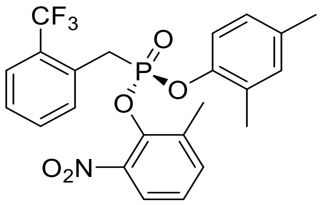

HPLC Conditions: CHIRALPAK AD-H, hexane/isopropanol = 80/20, 1 mL/min, λ = 220 nm

Racemic

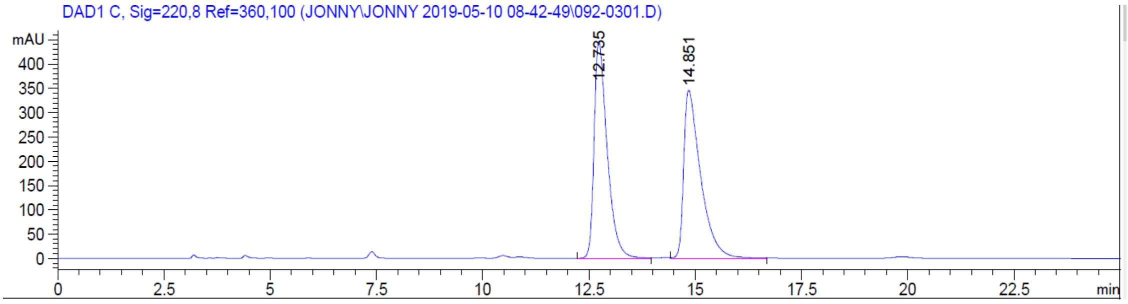

Signal 3: DAD1 C, Sig=220,8 Ref=360,100

Peak #	RetTime [min]	Type	Width [min]	Area [mAU*s]	Height [mAU]	Area %
1	12.735	BB	0.3213	9544.25488	446.66455	49.9020
2	14.851	VB	0.4069	9581.74805	346.15640	50.0980

Enantioenriched

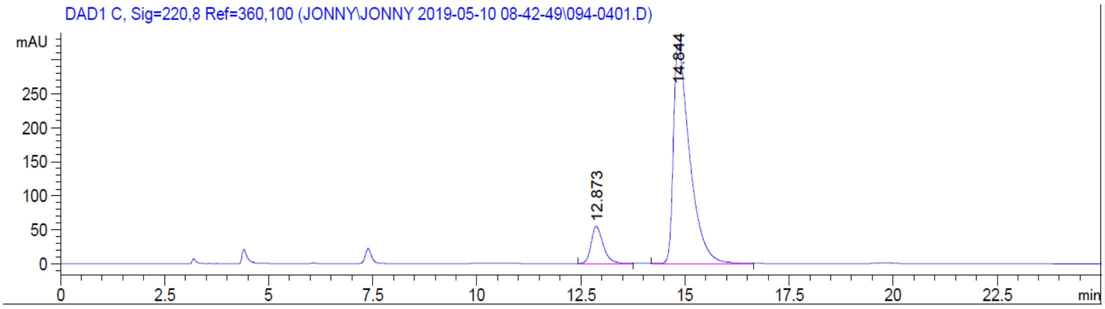

Signal 3: DAD1 C, Sig=220,8 Ref=360,100

Peak #	RetTime [min]	Type	Width [min]	Area [mAU*s]	Height [mAU]	Area %
1	12.873	BB	0.3075	1119.27466	54.98465	11.3094
2	14.844	BB	0.4011	8777.58789	322.89600	88.6906

Compound **21**

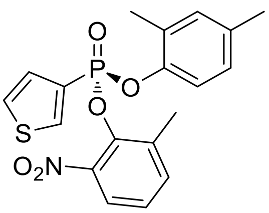

HPLC Conditions: CHIRALPAK AD-H, hexane/isopropanol = 70/30 1 mL/min, λ = 220 nm

Racemic

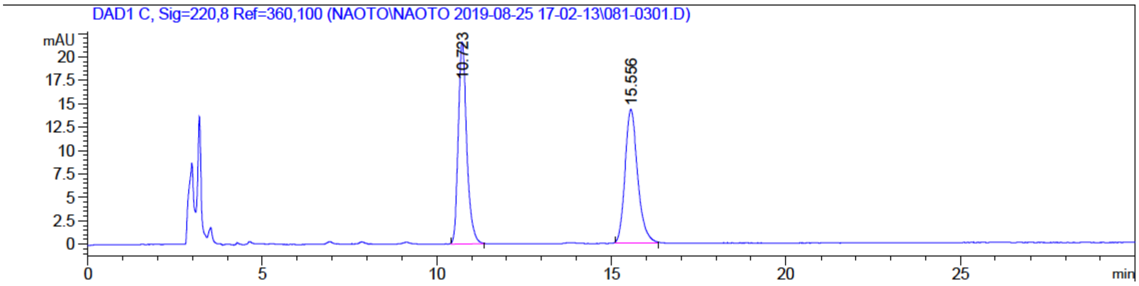

Peak #	RetTime [min]	Type	Width [min]	Area [mAU*s]	Height [mAU]	Area %
1	10.723	BB	0.2542	362.26984	21.54199	50.1680
2	15.556	BB	0.3839	359.84381	14.28391	49.8320

Enantioenriched

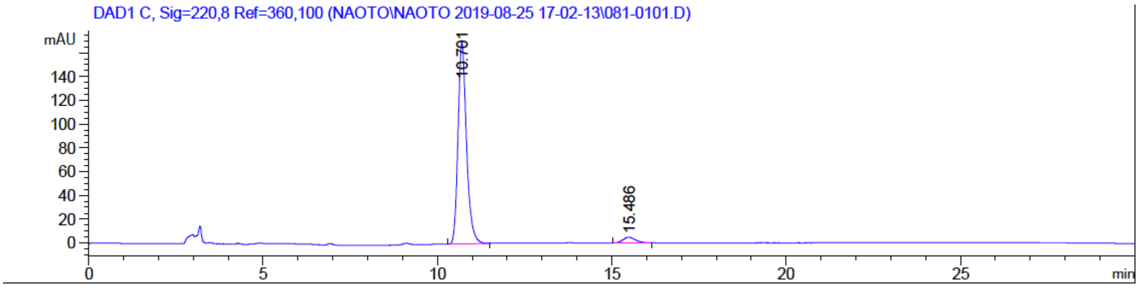

Peak #	RetTime [min]	Type	Width [min]	Area [mAU*s]	Height [mAU]	Area %
1	10.701	BB	0.2555	2857.20801	170.51819	96.0078
2	15.486	BB	0.3846	118.80994	4.77062	3.9922

Compound **22**

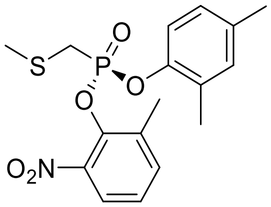

SFC Conditions: CHIRALPAK ID, 1500 psi, 30 °C, flow : 1.5 mL/min, from 1% to 30% MeOH in 5 mins, λ = 220 nm, t (minor) = 3.91 min, t (major) = 4.22 min

Racemic

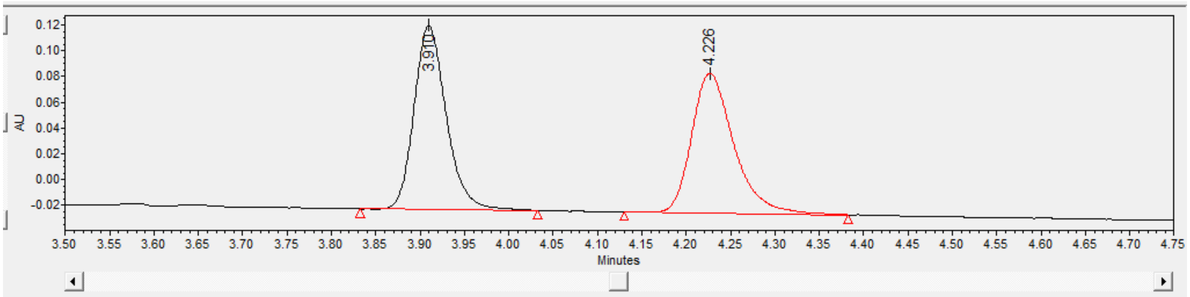

Name	Retention Time (min)	Purity1 Angle	Purity1 Threshold	PDA/FLR Match1 Spect. Name	PDA/FLR Match1 Angle	PDA/FLR Match1 Threshold	PDA/FLR Match1 Lib. Name	Area (µV*sec)	% Area	Height (µV)	Int Type	Amount	Units	Peak Type	Peak Codes
	3.910							359218	50.04	142816	bb			Unknown	
	4.226							358605	49.96	108314	bb			Unknown	

Enantioenriched

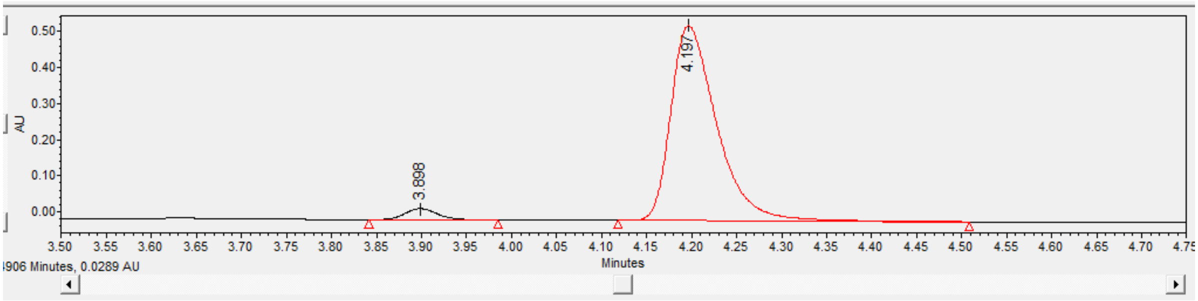

Name	Retention Time (min)	Purity1 Angle	Purity1 Threshold	PDA/FLR Match1 Spect. Name	PDA/FLR Match1 Angle	PDA/FLR Match1 Threshold	PDA/FLR Match1 Lib. Name	Area (µV*sec)	% Area	Height (µV)	Int Type	Amount	Units	Peak Type	Peak Codes
	3.898							78228	3.92	31065	bb			Unknown	
	4.197							1915438	96.08	541167	bb			Unknown	

Compound **23**

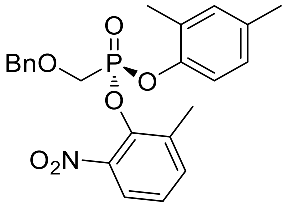

HPLC Conditions: CHIRALPAK AD-H, hexane/isopropanol = 80/20, 1 mL/min, λ = 220 nm

Racemic

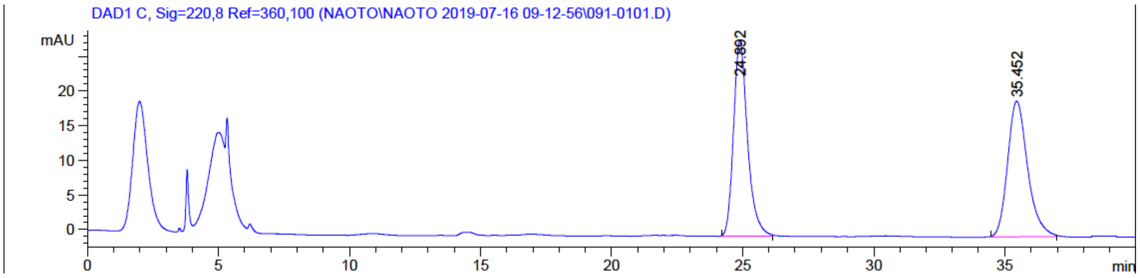

Peak #	RetTime [min]	Type	Width [min]	Area [mAU*s]	Height [mAU]	Area %
1	24.892	BB	0.5619	1047.14563	28.28589	50.1310
2	35.452	BB	0.8057	1041.67126	19.61233	49.8690

Enantioenriched

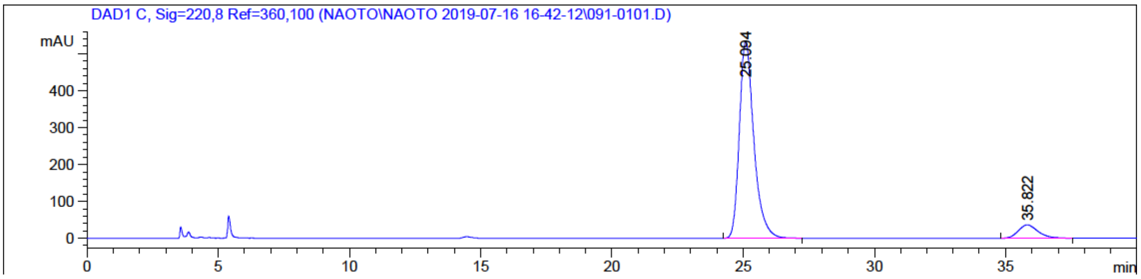

Peak #	RetTime [min]	Type	Width [min]	Area [mAU*s]	Height [mAU]	Area %
1	25.094	BB	0.5829	2.05876e4	535.04950	91.2355
2	35.822	BB	0.8233	1977.73914	36.54965	8.7645

Compound **24**

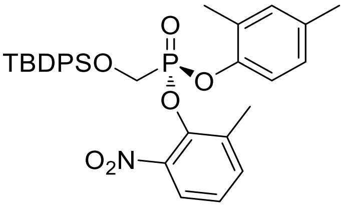

HPLC Conditions: CHIRALPAK IA, hexane/isopropanol = 85/15, 1 mL/min, λ = 220 nm, t (major) = 9.32 min, t (minor) = 15.98 min

Racemic

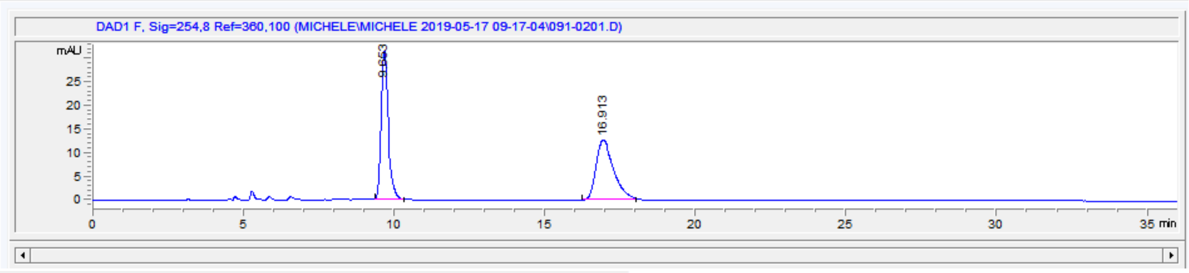

#	Time	Area	Height	Width	Area%	Symmetry
1	9.653	533.5	31.4	0.2542	50.406	0.674
2	16.913	524.9	12.7	0.6382	49.594	0.592

Enantioenriched

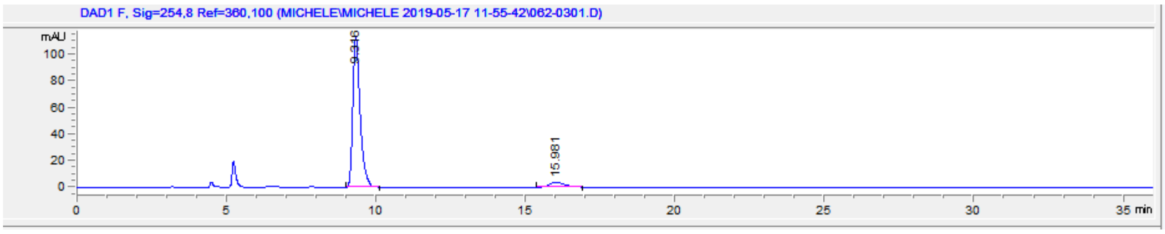

#	Time	Area	Height	Width	Area%	Symmetry
1	9.316	1845.6	112.4	0.2475	92.915	0.636
2	15.981	140.7	3.9	0.5258	7.085	0.692

Compound **25**

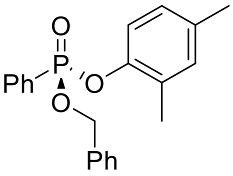

HPLC Conditions: CHIRALPAK AD-H, hexane/isopropanol = 70/30, 1 mL/min, λ = 220 nm

Racemic

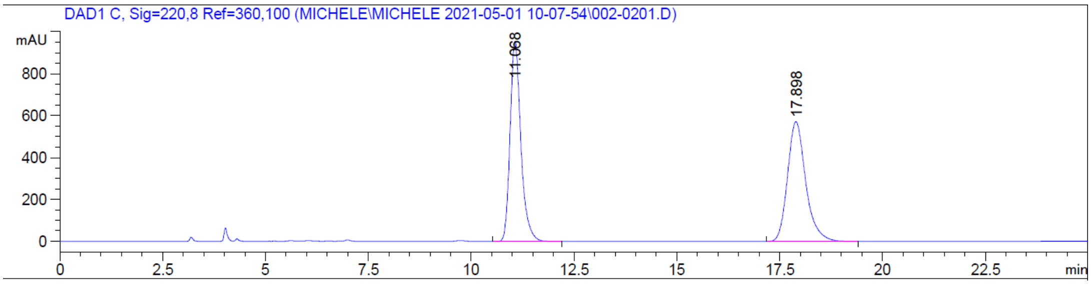

Signal 3: DAD1 C, Sig=220,8 Ref=360,100

Peak #	RetTime [min]	Type	Width [min]	Area [mAU*s]	Height [mAU]	Area %
1	11.068	VB	0.2773	1.74491e4	954.20685	49.7248
2	17.898	BB	0.4724	1.76423e4	571.12152	50.2752

Totals : 3.50914e4 1525.32837

Enantioenriched

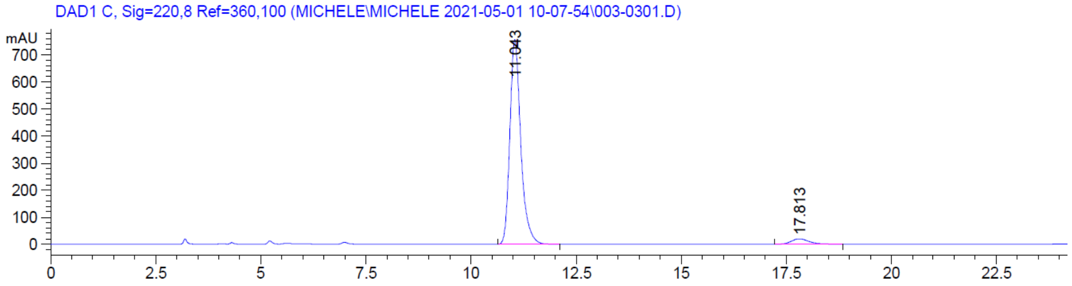

Signal 3: DAD1 C, Sig=220,8 Ref=360,100

Peak #	RetTime [min]	Type	Width [min]	Area [mAU*s]	Height [mAU]	Area %
1	11.043	BB	0.2754	1.37025e4	756.26678	95.7056
2	17.813	BB	0.4619	614.84375	20.27009	4.2944

Totals : 1.43174e4 776.53688

Compound **34**

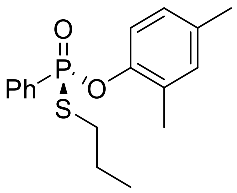

HPLC Conditions: CHIRALPAK AD-H, hexane/isopropanol = 95/5, 1 mL/min, λ = 220 nm

Racemic

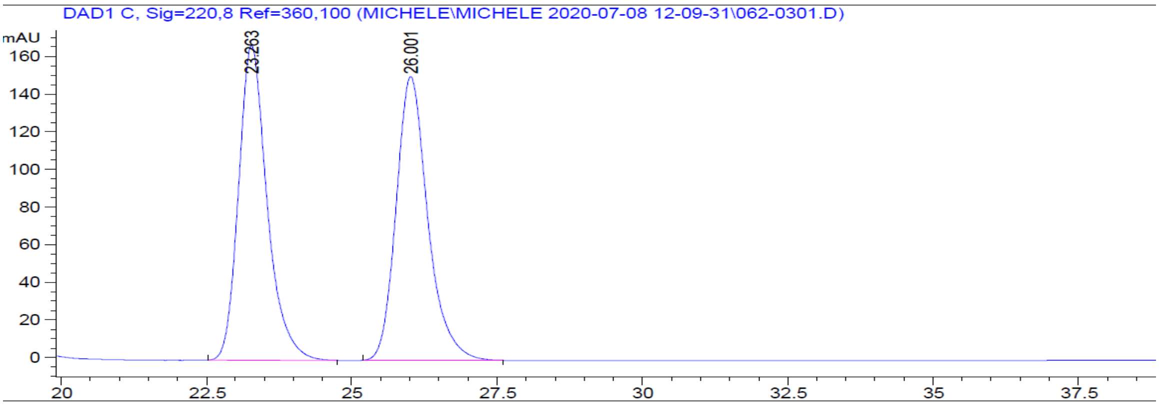

Signal 3: DAD1 C, Sig=220,8 Ref=360,100

Peak #	RetTime [min]	Type	Width [min]	Area [mAU*s]	Height [mAU]	Area %
1	23.263	BB	0.5196	5724.74658	167.14967	50.0092
2	26.001	BB	0.5788	5722.63330	150.77969	49.9908

Enantioenriched

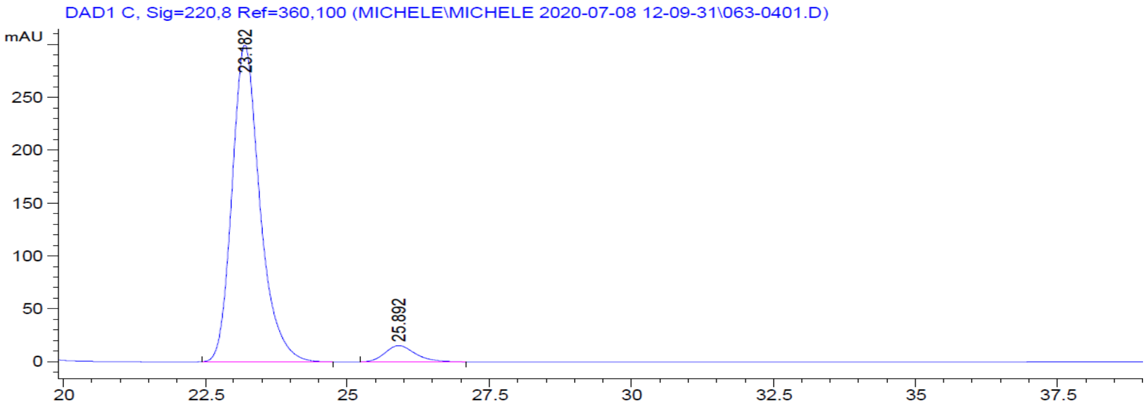

Signal 3: DAD1 C, Sig=220,8 Ref=360,100

Peak #	RetTime [min]	Type	Width [min]	Area [mAU*s]	Height [mAU]	Area %
1	23.182	BB	0.5189	1.02620e4	300.09662	94.6119
2	25.892	BB	0.5763	584.41821	15.48411	5.3881

Compound **35**

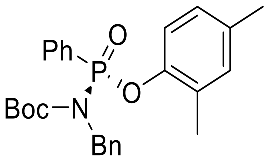

HPLC Conditions: CHIRALPAK OD-H, hexane/isopropanol = 99/1, 1 mL/min, λ = 220 nm

Racemic

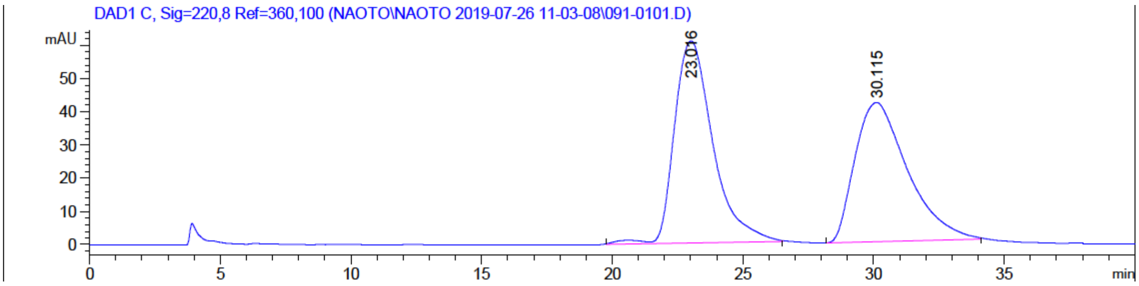

Peak #	RetTime [min]	Type	Width [min]	Area [mAU*s]	Height [mAU]	Area %
1	23.016	BB	1.5917	6264.06689	60.91538	51.5580
2	30.115	BB	2.1579	5885.48340	41.97310	48.4420

Enantioenriched

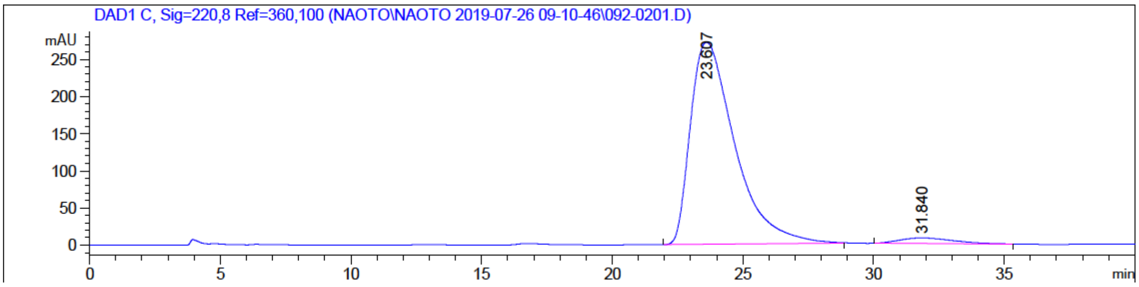

Peak #	RetTime [min]	Type	Width [min]	Area [mAU*s]	Height [mAU]	Area %
1	23.607	BB	1.8046	3.20962e4	272.02316	96.6608
2	31.840	BB	1.8691	1108.77686	7.67670	3.3392

Compound **36**

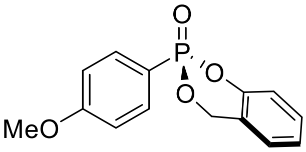

HPLC Conditions: CHIRALPAK IA, hexane/isopropanol = 80/20, 1 mL/min, λ = 220 nm

Racemic

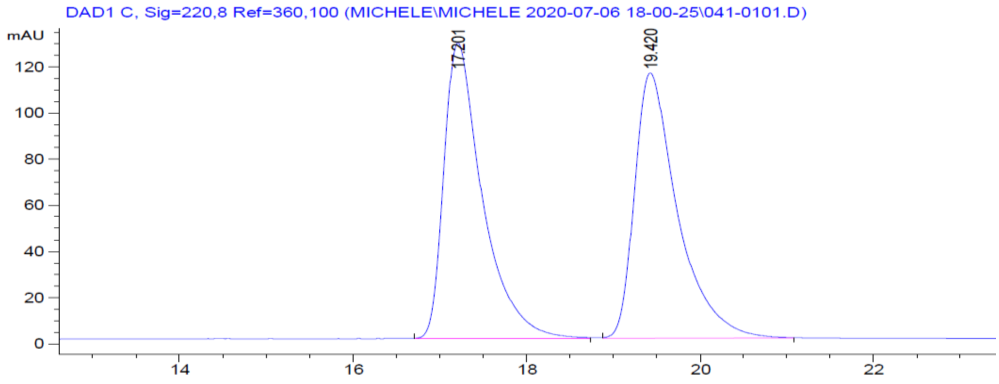

Signal 3: DAD1 C, Sig=220,8 Ref=360,100

Peak #	RetTime [min]	Type	Width [min]	Area [mAU*s]	Height [mAU]	Area %
1	17.201	BB	0.4588	3965.88916	128.23767	49.9892
2	19.420	BB	0.5082	3967.59766	115.09001	50.0108

Enantioenriched

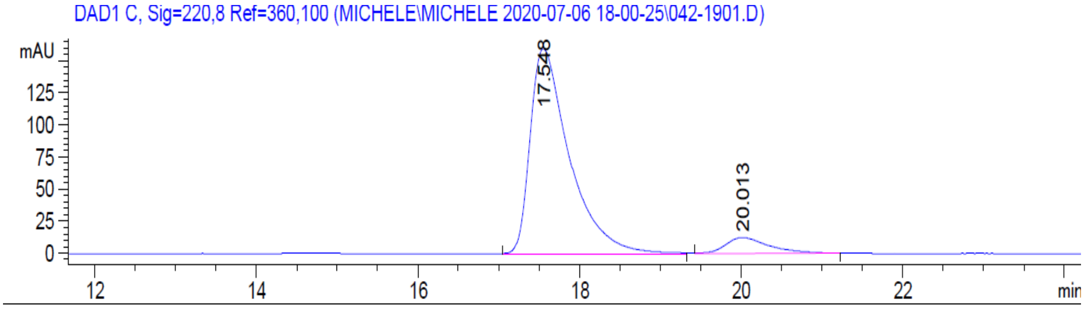

Signal 3: DAD1 C, Sig=220,8 Ref=360,100

Peak #	RetTime [min]	Type	Width [min]	Area [mAU*s]	Height [mAU]	Area %
1	17.548	BB	0.4975	5380.30664	159.52045	91.9845
2	20.013	BB	0.5693	468.83838	12.39367	8.0155

Single Crystal X-Ray Diffraction Data

Low temperature[27] single crystal X-ray diffraction data were collected using a Rigaku Oxford Diffraction SuperNova diffractometer. Raw frame data were reduced using CrysAlisPro and the structures were solved using 'Superflip'[28] before refinement with CRYSTALS[29] as per the SI (CIF). Full refinement details are given in the Supporting Information (CIF); Crystallographic data have been deposited with the Cambridge Crystallographic Data Centre (CCDC 2097043-44) and can be obtained via www. ccdc.cam.ac.uk/data_request/cif.

Compound 1 (CCDC: 2097043)

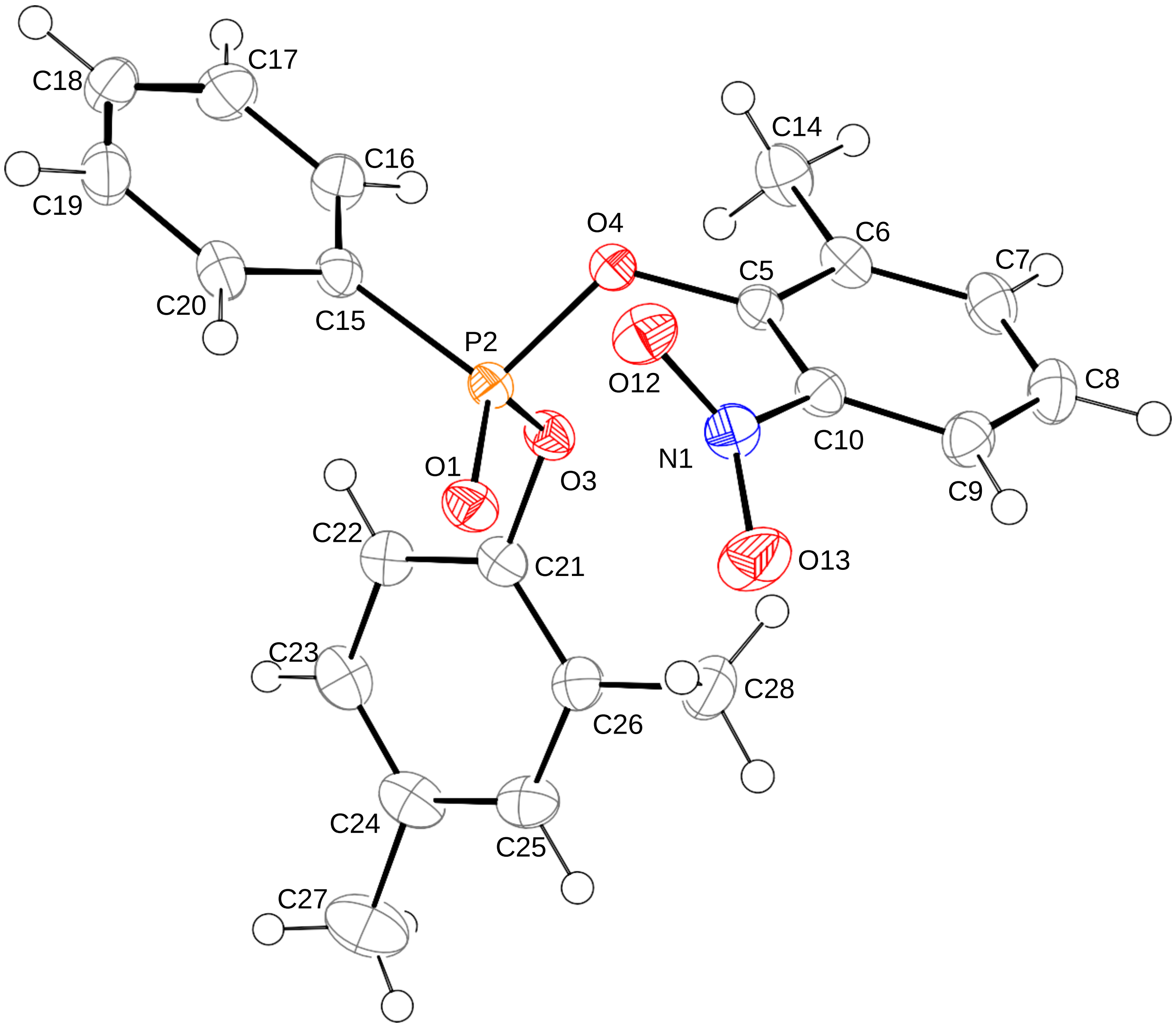

[27] Cosier J.; Glazer, A. M. *J. Appl. Cryst.*, **1986**, *19*, 105-107.

[28] Palatinus, L.; Chapuis, G. *J. Appl. Cryst.*, **2007**, *40*, 786-790.
[29] (a) Parois P.; Cooper, R. I.; Thompson, A. L. *Chem. Cent. J.*, **2015**, *9*, 30. (b) Cooper, R. I.; Thompson, A. L.; Watkin, D. J. *J. Appl. Cryst.* **2010**, *43*, 1100-1107.

Table S2. Crystal data and structure refinement for **1**.

Identification code	**1 -** 7381	
Empirical formula	C21 H20 N O5 P	
Formula weight	397.37	
Temperature	150 K	
Wavelength	1.54184 Å	
Crystal system	Monoclinic	
Space group	$P 2_1$	
Unit cell dimensions	a = 7.85800(10) Å	α= 90°.
	b = 10.65460(10) Å	β= 102.0500(14)°.
	c = 12.0478(2) Å	γ = 90°.
Volume	986.46(2) $Å^3$	
Z	2	
Density (calculated)	1.338 Mg/m^3	
Absorption coefficient	1.515 mm^{-1}	
F(000)	416	
Crystal size	0.10 x 0.05 x 0.02 mm^3	
Theta range for data collection	3.752 to 76.371°.	
Index ranges	-9<=h<=9, -13<=k<=13, -15<=l<=14	
Reflections collected	21118	
Independent reflections	4101 [R(int) = 0.042]	
Completeness to theta = 76.371°	99.7 %	
Absorption correction	Semi-empirical from equivalents	
Max. and min. transmission	0.97 and 0.86	
Refinement method	Full-matrix least-squares on F^2	
Data / restraints / parameters	4101 / 1 / 254	
Goodness-of-fit on F^2	1.0009	
Final R indices [I>2sigma(I)]	R1 = 0.0257, wR2 = 0.0668	
R indices (all data)	R1 = 0.0263, wR2 = 0.0674	
Absolute structure parameter	0.000(7)	
Largest diff. peak and hole	0.05 and -0.05 e.$Å^{-3}$	

Compound 37 (CCDC: 2097044)

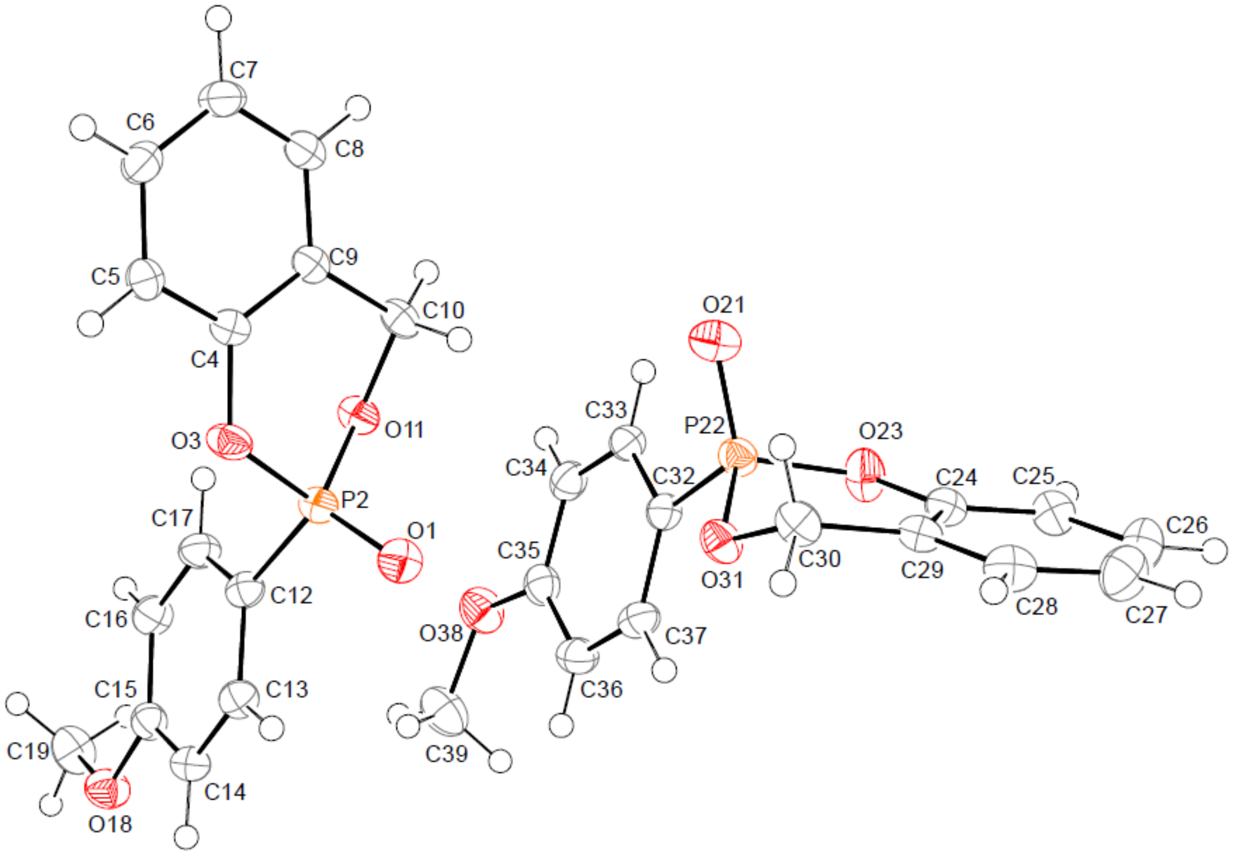

Table S3. Crystal data and structure refinement for **37**.

Identification code	**37 -** 7379	
Empirical formula	C14 H13 O4 P	
Formula weight	276.23	
Temperature	150 K	
Wavelength	1.54184 Å	
Crystal system	Monoclinic	
Space group	P 2_1	
Unit cell dimensions	a = 15.0390(3) Å	α= 90°.
	b = 5.80870(10) Å	β= 115.683(2)°.
	c = 15.9467(3) Å	γ = 90°.
Volume	1255.43(5) $Å^3$	
Z	4	
Density (calculated)	1.461 Mg/m^3	
Absorption coefficient	2.027 mm^{-1}	
F(000)	575.997	
Crystal size	0.23 x 0.20 x 0.19 mm^3	
Theta range for data collection	3.261 to 75.971°.	
Index ranges	-18<=h<=18, -7<=k<=7, -17<=l<=20	
Reflections collected	17317	
Independent reflections	5177 [R(int) = 0.024]	
Completeness to theta = 75.971°	99.5 %	
Absorption correction	Semi-empirical from equivalents	
Max. and min. transmission	0.68 and 0.64	
Refinement method	Full-matrix least-squares on F^2	
Data / restraints / parameters	5171 / 1 / 344	
Goodness-of-fit on F^2	1.0165	
Final R indices [I>2sigma(I)]	R1 = 0.0284, wR2 = 0.0781	
R indices (all data)	R1 = 0.0288, wR2 = 0.0785	
Absolute structure parameter	0	
Largest diff. peak and hole	0.23 and -0.24 e.$Å^{-3}$	
